# Benzoates as photosensitization catalysts and auxiliaries in efficient, practical, light-powered direct C(sp^3^)–H fluorinations†

**DOI:** 10.1039/d2sc05735b

**Published:** 2022-11-11

**Authors:** Shahboz Yakubov, Willibald J. Stockerl, Xianhai Tian, Ahmed Shahin, Mark John P. Mandigma, Ruth M. Gschwind, Joshua P. Barham

**Affiliations:** Fakultät für Chemie und Pharmazie, Universität Regensburg 93040 Regensburg Germany Joshua-Philip.Barham@chemie.uni-regensburg.de; Chemistry Department, Faculty of Science, Benha University 13518 Benha Egypt

## Abstract

Of the methods for direct fluorination of unactivated C(sp^3^)–H bonds, photosensitization of SelectFluor is a promising approach. Although many substrates can be activated with photosensitizing catalysts, issues remain that hamper fluorination of complex molecules. Alcohol- or amine-containing functional groups are not tolerated, fluorination regioselectivity follows factors endogenous to the substrate and cannot be influenced by the catalyst, and reactions are highly air-sensitive. We report that benzoyl groups serve as highly efficient photosensitizers which, in combination with SelectFluor, enable visible light-powered direct fluorination of unactivated C(sp^3^)–H bonds. Compared to previous photosensitizer architectures, the benzoyls have versatility to function both (i) as a photosensitizing catalyst for simple substrate fluorinations and (ii) as photosensitizing auxiliaries for complex molecule fluorinations that are easily installed and removed without compromising yield. Our auxiliary approach (i) substantially decreases the reaction's induction period, (ii) enables C(sp^3^)–H fluorination of many substrates that fail under catalytic conditions, (iii) increases kinetic reproducibility, and (iv) promotes reactions to higher yields, in shorter times, on multigram scales, and even *under air*. Observations and mechanistic studies suggest an intimate ‘assembly’ of auxiliary and SelectFluor prior/after photoexcitation. The auxiliary allows other E_n_T photochemistry *under air*. Examples show how auxiliary placement proximally directs regioselectivity, where previous methods are substrate-directed.

## Abstract 2

Benzoates serve as catalysts or auxiliaries for photochemical E_n_T radical C(sp^3^)–H fluorinations. The auxiliary markedly increases scope and efficiency, enabling reactions of free alcohols, amines, and allowing rapid gram-scale fluorinations in air.

## Graphical abstract

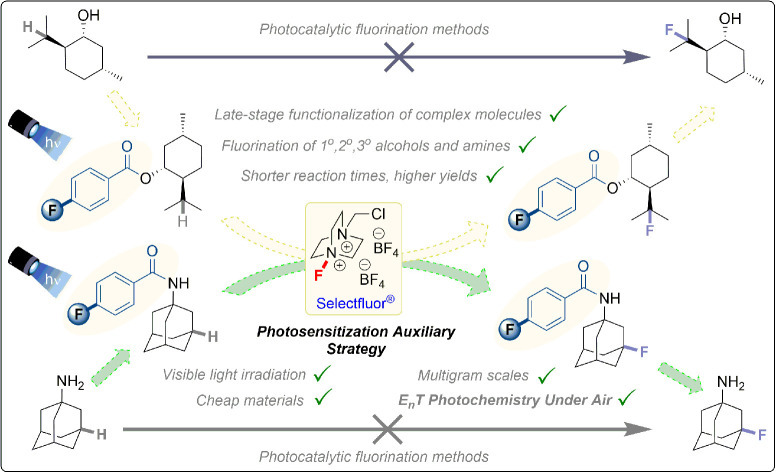

## Introduction

**Fig. 1:**
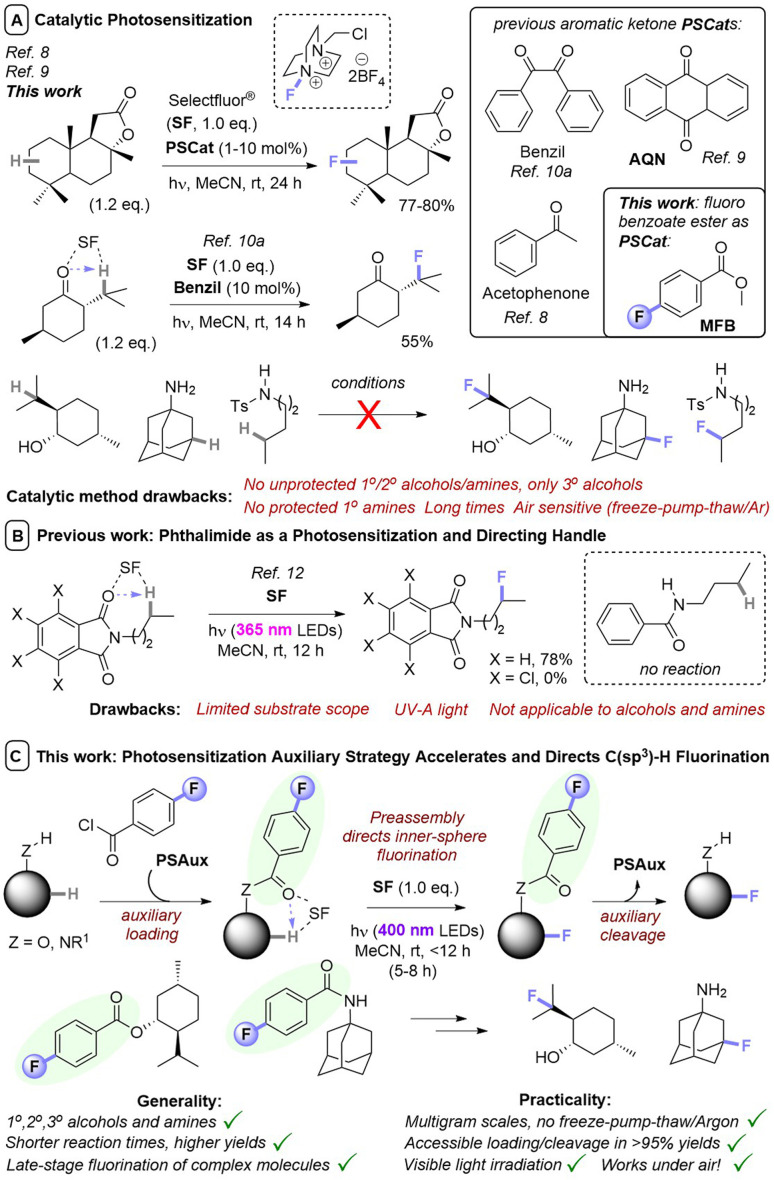
Photochemical direct fluorination of unactivated C(sp^3^)–H bonds. (A) Previous uses of catalytic aryl ketone photosensitizers and discovery herein of a catalytic benzoate photosensitizer. (B) Phthalimides as a photosensitization handle. (C) A general, efficient, selective, and rapid photosensitization auxiliary strategy using benzoyl groups.

Although numerous methods have been developed for fluorination reactions of C–H bonds, such as the fluorination of carbanions,^1^ arylpalladium complexes,^2^ and the addition of electrophilic fluorine to alkenes,^3^ few methods permit the direct fluorination of unactivated C(sp^3^)–H bonds.^4^ Such a transformation is particularly advantageous for the late-stage functionalization (LSF) of complex molecules. Especially, since F atoms as H atom bioisosteres impart vastly different – and usually favorable – properties (physical, ADME, lipophilicity) in a pharmaceutical/agrochemical context.^5^ Of these, methods that involve photosensitization of SelectFluor (SF) are particularly attractive for their mild conditions and use of light as a sustainable, uncontaminating energy source to drive reactivity.^6^ Here, SF functions as both a fluorine source and an activator of C(sp^3^)–H bonds; since quinuclidinium-type radical cations are powerful and selective hydrogen atom transfer (HAT) agents.^7^ Pioneering studies by Chen^8^ and Tan^9^ demonstrated photocatalytic, direct fluorinations of unactivated C(sp^3^)–H bonds by employing acetophenone or anthraquinone (AQN) as photosensitization catalysts (PSCats, Fig. 1A). Fluorination regioselectivity was governed by thermodynamic factors endogenous to the substrate; the most electron-rich C(sp^3^)–H bond that forms a stable 2° radical intermediate is fluorinated. Lectka used benzil as an alternative PSCat for the late-stage photocatalytic fluorination of complex molecules,^10^ including steroid derivatives, and found that ketones/enones innate to the substrate could direct regioselective C(sp^3^)–H fluorination in a proximal or distal fashion (Fig. 1A). Further examples of photosensitized C(sp^3^)–H fluorination have successfully engaged benzylic positions of simple substrates^10*a*^ or complex peptides,^10*c*^ as well as ketal substrates.^10*e*^

In most cases, PSCats belonged to the family of aryl ketones as a privileged architecture for photosensitization, whose success has been attributed to the matching of triplet energies with SF.^9*a*^ While the utilized aryl ketone catalysts alone do not absorb visible light, their assembly with SF affords a charge transfer complex with a tailing absorption >400 nm.^9*b*^ However, in all cases fluorinations of molecules containing 1°/2° alcohols or amines were unsuccessful (only 3° alcohols were tolerated), likely due to competing nucleophilic destruction of SF when the photochemical reaction is kinetically slow.^11^ This severely hampers applications to complex bioactive molecules such as pharmaceuticals. Recently, Egami and Hamashima^12^ found molecules with *N*-alkylphthalimides underwent directed photochemical C(sp^3^)–H fluorination of their alkyl chains (Fig. 1B). However, both an *N*-alkyl tetrachlorophthalimide and an *N*-alkyl benzamide derivative were unsuccessful, leading authors to suggest a key role for the phthalimide's triplet energy. The phthalimide could be considered as a ‘photosensitization auxiliary’ (PSAux) that can be cleaved to reveal a fluorinated 1° amine, though this was not realized and would typically require toxic, explosive hydrazine.

We envisioned a PSAux that is easily incorporated and removed could increase the generality and rapidity of photochemical C(sp^3^)–H fluorinations. The PSAux may form an intimate assembly with SF for inner-sphere photochemistry, increasing reaction efficiency. Moreover, an appropriately designed PSAux (*e.g.* containing a C=O group^10*a*^) may even provide an exogenous user handle to direct regioselectivity for the first time, where all previous reports follow factors/functionality endogenous to the substrate. Herein, we report the discovery of benzoyl groups as novel, versatile photosensitizer architectures. Methyl 4-fluorobenzoate serves as a photosensitizing catalyst for fluorination of simple substrates, while the 4-fluorobenzoyl group serves as a PSAux for the fluorination of complex molecules containing alcohols and amines and can be easily installed and removed without compromising product yields. The PSAux strategy (i) markedly increases the efficiency, rapidity, reliability and practicality of C(sp^3^)–H fluorination reactions; (ii) enables reactions of substrates that cannot be engaged with photocatalysis (alcohols and amines, where previous catalytic methods were generally only applicable to 3° alcohols); (iii) allows reactions to succeed under air atmosphere and (iv) offers promise to direct fluorination regioselectivity (Fig. 1C).

## Results and discussion

### Discovery

When examining the C(sp^3^)–H fluorination of amyl benzoate 1a to 2a as reported by Tan and co-workers^9*a*^ (Table 1, entries 1 and 2) with AQN and derivatives, we found that no catalyst was necessary; the reaction proceeded in its absence at 365 and 400 nm (entries 3 and 4). The PSCat-free reaction did not proceed at 451 nm or in the absence of light (entries 5 and 6). Assuming that 1a was acting as a photosensitizer for its own self-fluorination, we reasoned that arylbenzoate esters may function as exogenous PSCats in the C(sp^3^)–H fluorination of 1-adamantanol 1b to 2b and 3b. However, 1a was an ineffective PSCat (entry 7). Assuming that self-fluorination of 1a may deactivate its catalysis of the fluorination of 1b, methyl benzoate (4a) was attempted but was similarly unsuccessful (entry 8).

**Table 1:** Initial investigations and optimization of conditions^a^

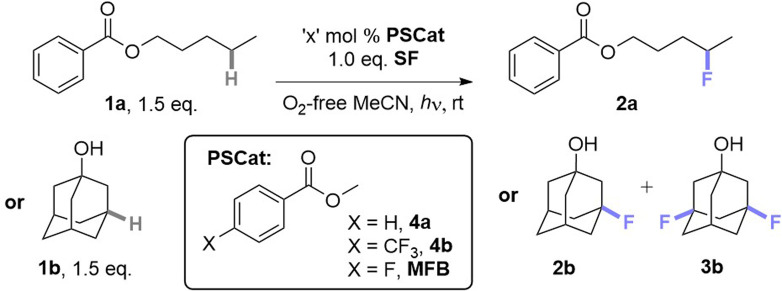					
Entry	Substrate^b^	PSCat, ‘*x*’	*λ* (nm)	*t* (h)	Product, yield^c^
1	1a	AQN, 2%	365	48	2a, 46%
2	1a	AQN, 2%	400	48	2a, 46%
3	1a	—	365	48	2a, 55%
4	1a	—	400	48	2a, 44%
5	1a	—	451	24	2a, n.d.
6	1a	—	—	24	2a, n.d.
7	1b	1a, 5%	400	24	2b, <5%; 3b, n.d.
8	1b	4a, 5%	400	24	2b, n.d.; 3b, n.d.
9	1b	4b, 5%	400	24	2b, <5%; 3b, n.d.
10	1b	MFB, 5%	400	24	2b, 43%; 3b, 3%
11	1b	—	400	48	2b, <5%; 3b, n.d.
12	1b	MFB, 5%	400	48	2b, 53%; 3b, 5%
13	1b	MFB, 10%	400	48	2b, 65%; 3b, 6%
14^d^^,^^e^	1b	MFB, 10%	400	24	2b, 75%; 3b, 6%
15	1b [0.31 M]	MFB, 1%	400	48	2b, 60%; 3b, 12%
16^d^	1b [0.31 M]	MFB, 1%	400	6	2b, 28%; 3b, 3%
17^d^^,^^f^	1b [0.31 M]	MFB, 1%	400	24	2b, 84%; 3b, 10%
18^g^	1b [0.31 M]	MFB, 1%	400	24	2b, <5%; 3b, n.d.

a nd., not detected.

b Unless otherwise specified, substrate concentration was [0.16 M] and a 0.35 W (input) 400 nm LED was employed.

c Yields determined by ^19^F NMR spectroscopy with trifluorotoluene as an internal standard and correspond to combined fluorinated regioisomers.

d A 3.8 W (input) LED was employed.

e Hereafter termed conditions A.

f Hereafter termed conditions B.

g Prepared under air.

While other *para*-substituted benzoate esters (4, where X = Cl, Br or CF_3_, entry 9) were also ineffective (also see ESI†), methyl 4-fluorobenzoate (MFB) was a surprisingly effective PSCat (entry 10), delivering a 46% yield of 2b(+3b) compared to an ineffective control reaction without PSCat (entry 11). Employing 10 mol% of MFB provided 2b+3b in an improved 71% yield (entry 13). Doubling the overall concentration of 1b to 0.31 M and decreasing catalyst loading at this higher overall concentration also improved the yield (entry 15). For both the low and high concentration conditions, a higher power LED further increased the yield of 2b+3b in a shorter reaction time (entries 14 and 17, termed ‘conditions A’ and ‘conditions B’, respectively), although 6 h was here insufficient for full conversion (entry 16). Air, protic additives and using an equimolar ratio of SelectFluor (*vs.* substrate) were all detrimental to the reaction (for full investigations, see ESI†).

### Synthetic scope of PSCat method

Under these photocatalytic conditions, the C(sp^3^)–H fluorinations of a variety of small molecules was achieved in modest to excellent (31–94%) yields (Table 2). Though electronically bearing resemblance to amyl benzoate, substrates 1c–1e did not fluorinate in the absence of MFB. Substrates 1d–1g underwent fluorination not at their benzylic nor terminal positions but at the most electron-rich C(sp^3^)–H bond that involves a 2° radical intermediate. Substrates 1h–1j underwent benzylic fluorination likely due to their benzylic C(sp^3^)–H bonds being the least electron-deficient. Aliphatic esters 1k, 1l (sclareolide), 1y (ibuprofen methyl ester) – and aliphatic ketone 1m – were all fluorinated at their most electron-rich (or benzylic) C(sp^3^)–H bonds remote from the carbonyl group. Steroidal derivatives 1o, cholest-4-en-3-one (1p) were successfully fluorinated. Bromoalkane 1q, cyanoalkane 1r and trialkylamine 1s were fluorinated at their most electron rich C(sp^3^)–H positions. When unactivated hydrocarbons 1t–1x were subjected to the reaction, C(sp^3^)–H fluorination was selective for 3° positions > 2° positions (when present). Both pyrrolidinium and imidazolium-type ionic liquids (1aa, 1ac–1ae) and a common food flavor additive (undecanoic γ-lactone, 1ab) were fluorinated in good to excellent (61–99%) yields demonstrating (i) the tolerance of heterocyclic motifs (also see 1l) and (ii) synthetic utility of the method for ‘real-world’ molecules.

**Table 2:** Scope of photocatalytic C(sp^3^)–H fluorination method^a^

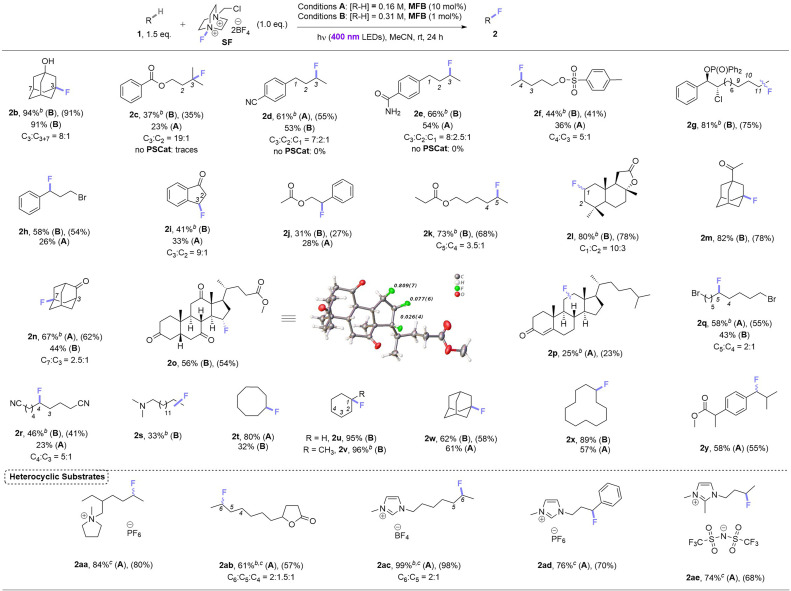

a For substrates with more than one regioisomer, the major isomer is depicted. NMR yields were determined by ^19^F NMR with trifluorotoluene as internal standard (IS). Unless explicitly defined, yields correspond to a single regioisomer. Isolated yields in parenthesis.

b Yield corresponds to the combined mixture of regioisomers.

c 2.0 eq. of SF used.

Despite a reasonably broad scope of applications of this catalytic method, we noticed that apart from 3° alcohols (*e.g.*1b), free alcohols (5a, 5b′, 5c–5e, 5g) and free amines (6a–6d) all failed under photocatalytic C(sp^3^)–H fluorinations (Table 3). We note that in the earlier reports of Chen,^8^ Tan,^9*a*^ and Lectka,^10*a–e*^ free 1° or 2° alcohols were never reported, only hindered 3° alcohols were tolerated. Neither were free 1°/2°/3° amine moieties; 1° and 2° amines always required protection. Even protected 1° amide 7 did not fluorinate in this case. One explanation could be that if the desired reactivity is too slow, nucleophilic destruction of SelectFluor (known to occur with 1° and 3° amines)^13^ may emerge as a competitive thermal reaction, arising lower yields or no reactivity. The steric hindrance of 3° alcohols – the only class of free alcohols that worked in the previous reports – may explain their tolerance. However, since nucleophilic 3° amines did proceed (1s, 1z) albeit in low (33%, 18%) yields, there may be other factors responsible for the failure of 1° and 2° alcohols/amines/amides. SF's known interactions with a wide range of functional groups in ground state chemistry – such as Lewis acid interactions with C–O bonds and fluorinations of nucleophilic (S,N) atoms^14^ – are likely detrimental to its use in photocatalytic C(sp^3^)–H fluorinations. Another explanation is that hydrogen bonding networks around alcohol/water-solvated SF prohibit its non-covalent assembly with the aromatic PSCat that is needed for successful photochemistry (*vide infra*).^9*b*^ Interestingly, although 5b has no unprotected alcohol and contained a privileged benzylic C(sp^3^)–H bond, it also reacted poorly (8% yield).

**Table 3:** Scope of photosensitization auxiliary C(sp^3^)–H fluorination method. (A) Unreactive, unprotected substrates. (B) Scope of photosensitization auxiliary method^a^

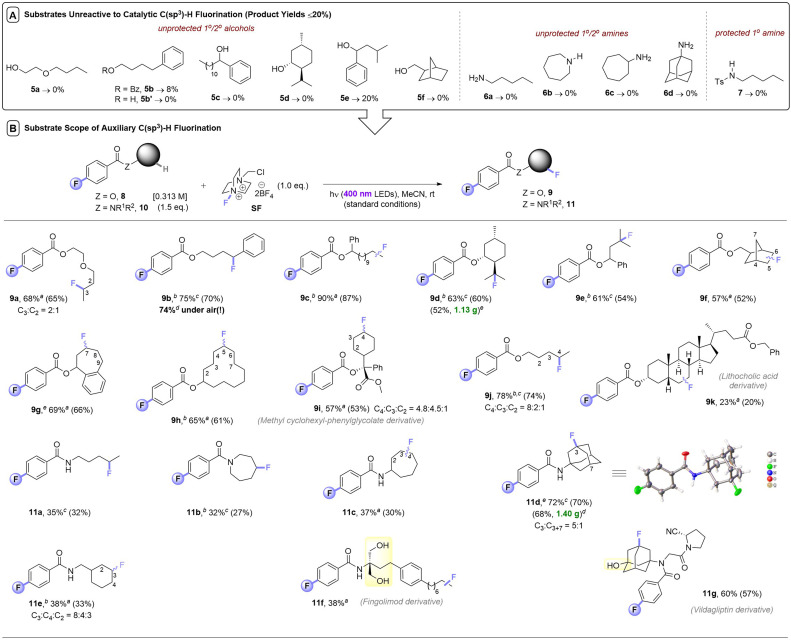

a For substrates with more than one regioisomer, the major isomer is depicted. NMR yields were determined by ^19^F NMR with trifluorotoluene as internal standard (IS). Unless explicitly defined, yields correspond to a single regioisomer. Isolated yields in parenthesis.

b Yield corresponds to a mixture of regioisomers.

c Reaction completed in 10 h.

d Yield corresponds to a single regioisomer.

e Isolated yields of gram scale reactions.

f Reaction completed in 5 h.

### Synthetic scope of PSAux method

At this juncture, we considered that a key advantage of our 4-fluorobenzoate photosensitizer compared to previously-employed aryl ketone PSCats was the ability to reversibly attach it to alcohol and amine substrates and then detach it after photochemical fluorination (both under simple conditions): a ‘photosensitization auxiliary’ (PSAux) approach.

Not only would this enable C(sp^3^)–H fluorinations that were catalytically inefficient or impossible (such as complex molecules), it would accelerate reaction kinetics and improve reproducibility (*vide infra*). Moreover, it would improve practicality by decreasing atmospheric sensitivity and simplify purification of otherwise highly polar unprotected substrates. Thus, a variety of alcohols 8 and amines 10 were loaded with the 4-fluorobenzoate PSAux in near-quantitative yields. To our delight, auxiliary-loaded alcohols 8a–i all underwent C(sp^3^)–H fluorination (Table 3), generally in good to excellent (57–90%) yields. Interestingly, where benzoate ester 5b provided only 8% yield, its 4-fluorobenzoate derivative 8b was fluorinated to afford 9b in 75% yield, highlighting the benefit of the PSAux in improving efficiency (even when unprotected alcohols/amines are absent). Moreover, while the catalytic fluorination affording 2k was completely ineffective under air, the yield of 9b (74%) was unaffected by setting up *under air*. Although the reaction of 1a without any catalyst gave a 44% yield of 2a (Table 1, entry 4), however, its PSAux derivative, amyl 4-fluorobenzoate, led to a 78% yield of 9j again confirming the benefit of the F atom in the PSAux. Though lithocholic acid derivative 8k only afforded a 23% yield of 9k, the C(sp^3^)–H fluorination of steroid derivatives was only possible previously with in-built enone/ketone/ketal protecting/directing groups.^10^

Gratifyingly, the C(sp^3^)–H fluorination of amines was also enabled *via* their auxiliary-loaded amide forms 10a–10e, affording 11a–11e in moderate to very good (32–72%) yields. The profound increase in reactivity from having the fluorine in the 4-position of the benzoate group was apparent here. Substrate 10a was successfully engaged, while the previously attempted photochemical fluorination of *N*-butylbenzamide (Fig. 1B)^12^ gave no reaction. Although phthalimides^12^ offer the potential to serve as a PSAux, the 4-fluorobenzoyl PSAux is both more effective and generally applicable since (i) phthalimides can only function in this way for 1° amines and ii) benzoates undergo straightforward deprotection (*vide infra*). To our surprise, the auxiliary loaded amide derived from Fingolimod 10f did not require any subsequent protection of its two free 1° alcohols. In summary, incorporation of a PSAux accelerates the rate of C(sp^3^)–H fluorination to a point that it (i) outcompetes the catalytic method, (ii) outcompetes degradation of SF, (iii) allows energy transfer (E_n_T) photosensitization reactions to succeed *under air*. This renders the PSAux an attractive strategy for C(sp^3^)–H fluorination of complex, bioactive molecules like active pharmaceutical ingredients, exemplified by the successful fluorination of phenylcyclohexylglycolic acid (8i), a derivative of lithocholic acid (8k), multiple sclerosis active pharmaceutical intermediate (API) Fingolimod (10f) and antidiabetic API vildagliptin (10g).

**Fig. 2:**
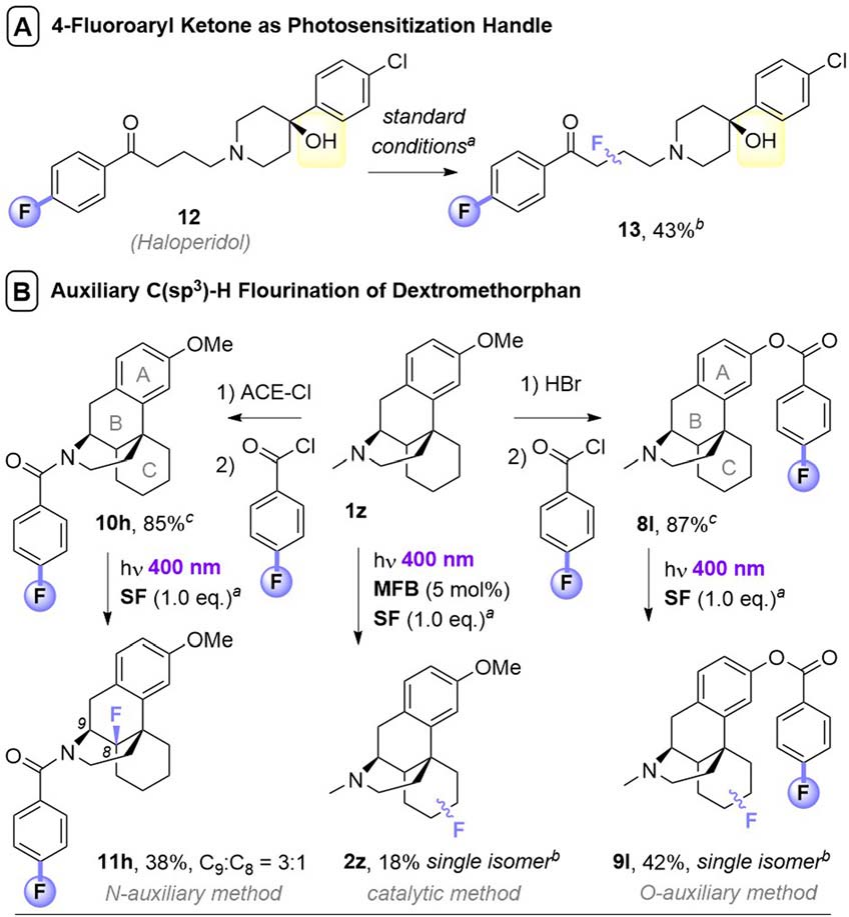
Applications of photosensitization auxiliary concept. ^a^Unless otherwise stated, standard conditions in Table 3B were used, with 1.5 eq. substrate and 1.0 eq. SF. Isolated yields given unless stated otherwise. ^b^Yield determined by ^19^F NMR with trifluorotoluene as IS. ^c^Overall yields over two steps.

**Fig. 3:**
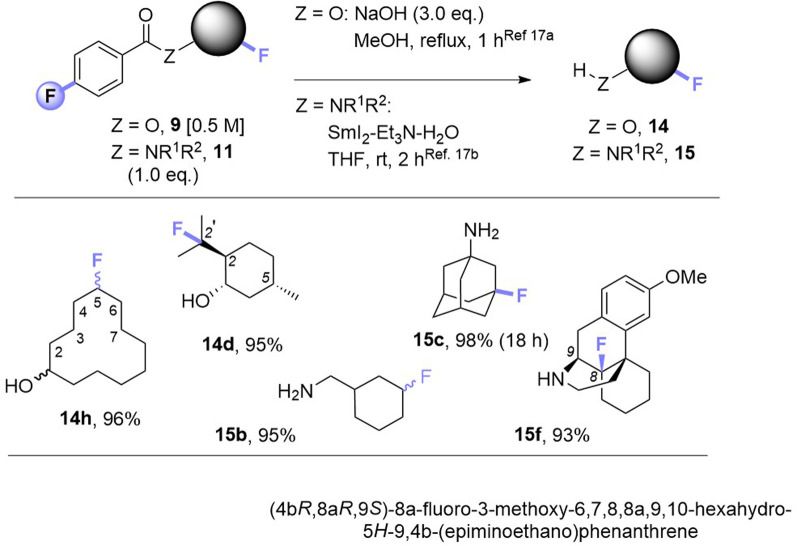
Cleavage of auxiliaries. Isolated yields are given. See Table 3 for regioselectivity.

Noticing that antipsychotic API Haloperiodol 12 contains a 4-fluoroacetophenone moiety, we reasoned its photochemical C(sp^3^)–H fluorination may occur without *any* catalyst or auxiliary. Indeed, its reaction successfully gave 13 in 43% yield (Fig. 2A). Haloperidol and Fingolimod feature in the top 200 pharmaceuticals by retail sales in 2020 ^15^ and their late-stage fluorination opens avenues to new chemical space and pharmacological activities. Finally, we investigated the impact of PSAux location on C(sp^3^)–H fluorination selectivity by the late-stage fluorination of Dextromethorphan 1z (Fig. 2B). The photocatalytic method with MFB was inefficient, affording an unsatisfactory (18%) yield of 2z. Following attachment of the PSAux to 1z by tandem *N*- or *O*-demethylation/PSAux loading – affording 10h and 8l in high yields over 2 steps (85 and 87%, respectively) – their photochemical C(sp^3^)–H fluorinations afforded 11h and 9l in satisfactory yields (42 and 48%, respectively). Interestingly, 11h was afforded as two fluorinated regioisomers on the ‘B’ ring (possibly assisted by a developing fluorine gauche effect)^16^ while 9l was a single regioisomer on the ‘C’ ring. Therefore, our PSAux auxiliary strategy offers future promise as an attachable handle to direct late-stage C(sp^3^)–H fluorination selectivity which (i) is not possible by previous catalytic methods and (ii) allows fluorination to be exogenously directed by the chemist, rather than endogenously directed by the molecule *via* its inherent functionality.^9*a*,10,12^ Of key importance and justifying the use of a PSAux strategy, the 4-fluorobenzoate auxiliaries can be cleaved by known methods^17^ to give alcohols or amines in near-quantitative (93–98%) yields (Fig. 3).

### Mechanistic studies of PSCat method

**Fig. 4:**
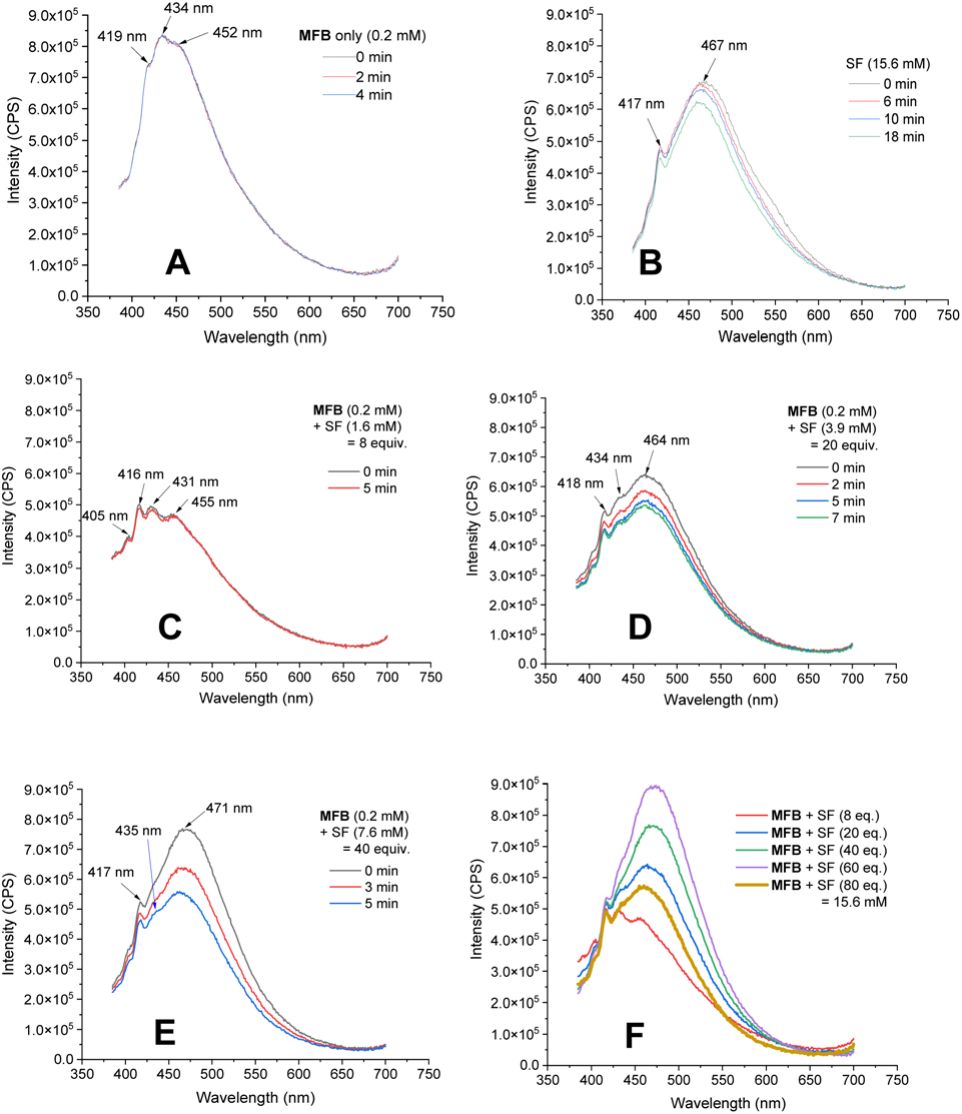
Luminescence of MFB and SF. (A) MFB only (0.2 mM), repeated measurements. (B) SF only (15.6 mM), repeated measurements. (C–E) MFB (0.2 mM) with increasing concentrations of SF, repeated measurements. (F) MFB (0.2 mM) with increasing concentration of SF.

**Fig. 5:**
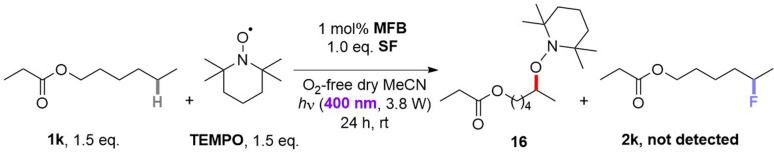
Radical trapping reaction of 1k with TEMPO.

**Fig. 6:**
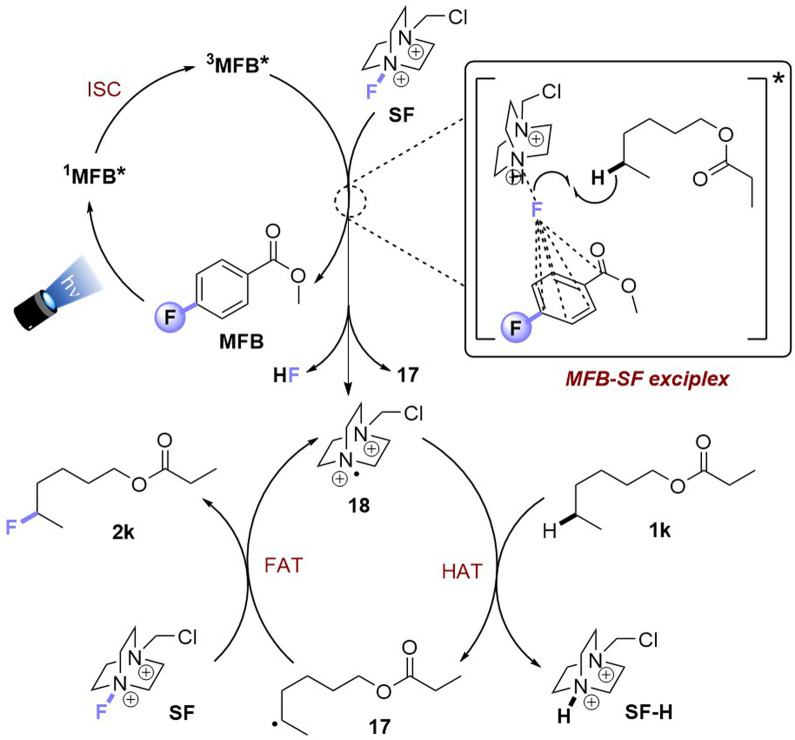
Proposed reaction mechanism for PSCat method.

Luminescence spectra of the reaction components were measured at their representative excesses (conditions B, 10× diluted to ensure SF would fully dissolve). While MFB showed no change at all after repeated excitations at *λ* = 375 nm (Fig. 4A), SF underwent a slow photodecomposition over 6–18 min (Fig. 4B; for photodecomposition rates, see ESI†). Upon mixing MFB (12.5 mol% w.r.t. SF) with SF (8 eq. w.r.t MFB), the peak at *λ* = 434 nm decreased in intensity by 42% (Fig. 4C). Its shape did not resemble SF (no peak at *λ* = 467 nm) and even after 5 min of repeated excitations the intensity barely decreased. Lu, Soo, Tan and co-workers also observed that the emission peak shape of AQN differed in the presence of SF.^9*b*^ Mixing MFB with larger excesses of SF (Fig. 4C–E) led to buildup of a peak at *λ* = 467 nm that more closely resembled SF. Interestingly, the presence of MFB led to faster photodecomposition of the SF peak compared to SF alone (Fig. 4, E vs. B). The measured lifetime of SF also decreased in the presence of MFB (see ESI†). These data corroborate an energy transfer between MFB and SF, in line with that proposed for previous arylketone catalysts.^9,10^ This suggests ^3^MFB* is the excited state involved, and its *T*_1_ energy is well matched with that of SF (see Table 4, *vide infra*). A radical trapping experiment with 1.5 eq. of TEMPO was performed. As clear evidence of the alkyl chain radical intermediate, a product was detected by LC-MS which matched the TEMPO-bound 16 and no fluorinated product (2k) was detected (Fig. 5). Therefore, the mechanism of the MFB-catalyzed reaction mirrors that previously proposed and elegantly investigated in the literature for AQN (Fig. 6).^9*b*,12^

**Table 4:** Triplet energies of reaction components

Entry	Substrate (→ surrogate of)	*T*_1_ a (kcal mol^−1^)
1	Methyl benzoate (→ 1a, 1c)	77.9
2	Benzonitrile (→ 1d)	76.8
3	Benzamide (→ 1e)	79.3
4	MFB	78.3

a Calculated using Time Dependent-Density Functional Theory (see ESI for details).

Upon photoexcitation and intersystem crossing, ^3^MFB* undergoes E_n_T with SF. An exciplex is formed involving substrate molecule (1k) which facilitates the N–F bond cleavage and HAT process.^9*b*^ Intermolecular HAT occurs between the radical dication of SF (18) and the most hydridic C(sp^3^)–H bond of the substrate (1k) that affords the more stable 2° alkyl radical 17 and SF–H. Once 17 is generated, fluorine atom transfer (FAT) with SF affords 2k. This may propagate a chain reaction, but a previously reported quantum yield of *ca.* 0.12 seems to oppose this (or suggests an inefficient chain propagation).^9*b*^

### Mechanistic studies of PSAux method

**Fig. 7:**
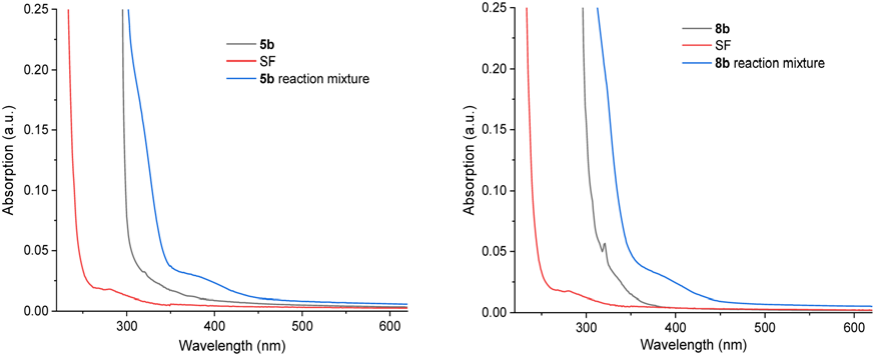
UV-visible spectroscopy of benzoate esters, SF and their reaction mixtures. Left: for benzoate 5b; right: for 4-fluorobenzoate 8b.

The majority of efforts were directed at understanding (i) the pronounced benefit of the PSAux method over the catalytic method, and (ii) the promoting role of the 4-fluorine atom in the PSAux. The first observation was that compounds 1c, 1d and 1e all gave no fluorination in the absence of MFB (Table 2), despite electronically resembling amyl benzoate (1a) and MFB. The lack of reactivity of 1e in the absence of PSCat is consistent with the previously reported lack of reactivity of *n*-butylbenzamide (Fig. 1B).^12^ The triplet energies (*T*_1_) for directly related compounds are summarized in Table 4. Time-dependent Density Functional Theory (TD-DFT) calculations were used to calculate *T*_1_ for MFB (78.3 kcal mol^−1^, see ESI†). Although the *T*_1_ values for all these compounds^18,19^ lay above that reported for SF (61.4 kcal mol^−1^),^9*a*^ no correlation existed between the *T*_1_ values and the efficiencies of the photochemical fluorinations of 1a–1d, confirming other factors underpinned successful reactivity. To probe further, we examined substrate 5b for which the catalytic method was ineffective, giving product 21 in only 8% yield (Table 5, entry 1). A control experiment without catalyst (entry 3) revealed this trace reactivity derived from photochemical self-fluorination, akin to amyl benzoate 1a but in very low efficiency. When a stoichiometric amount (150 mol%) of MFB was employed (entry 4), activity of the PSCat method was observed, affording product 21 in 47% yield. That the reaction of 8b without catalyst gave almost double (75%) the yield of 9b (entry 7) confirmed the PSAux provides a beneficial effect beyond simply higher ‘effective’ catalyst loading. It supports the notion that a preassembly co-locating substrate and SF in close proximity sets up a more efficient inner-sphere photochemical process. This is further supported by the fact that the synthetic reactions work equally well under the PSAux method without degassing (entry 7 *vs.* 8), but not under the PSCat method (entry 4 *vs.* 6) which requires strict freeze/pump/thaw cycling. NFSI as a different fluorinating source gave results that were (i) far inferior to SF (entries 8 and 9) and (ii) similar with both PSCat and PSAux methods (see ESI†), suggesting a different, unrelated mechanism operates here. UV-vis spectra of the reaction mixtures of 5b and 8b revealed, in both cases, an absorption band at *ca.* 400 nm (Fig. 7, see ESI for details†). This absorption was notably higher than any individual reaction component and was accessible at the synthetic experiments' LED wavelength, suggesting a charge-transfer complex^20^ forms between the PSAux and SF. However, the peak shape and absorption intensity was no different in the case of 5b's and 8b's reaction mixtures. Given the drastic difference in the efficiency of 5b's and 8b's reactions, the promoting role of the F atom in the PSAux's clearly does not involve chromophoric changes.

**Table 5:** Control reactions with different MFB loading

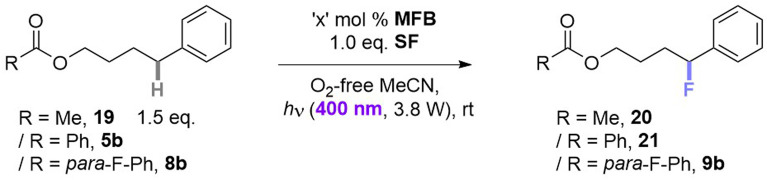			
Entry	R (Substrate)	MFB ‘*x*’ mol%	Yield^a^ (product)
1	Ph (5b)	1	8% (21)
2	Me (19)	1	19% (20)
3	Ph (5b)	0	10% (21)
4	Ph (5b)	150	47% (21)
5	Me (19)	150	30% (20)
6	Ph (5b)	150^b^	35% (21)
7	*para*-F–Ph (8b)	0	75% (9b)
8	*para*-F–Ph (8b)	0^b^	74% (9b)
9	*para*-F–Ph (8b)	0^c^	23% (9b)

a NMR yield, based on ^19^F NMR and trifluorotoluene as IS.

b Under air.

c Instead of SF, NFSI was used as a fluorine source.

**Fig. 8:**
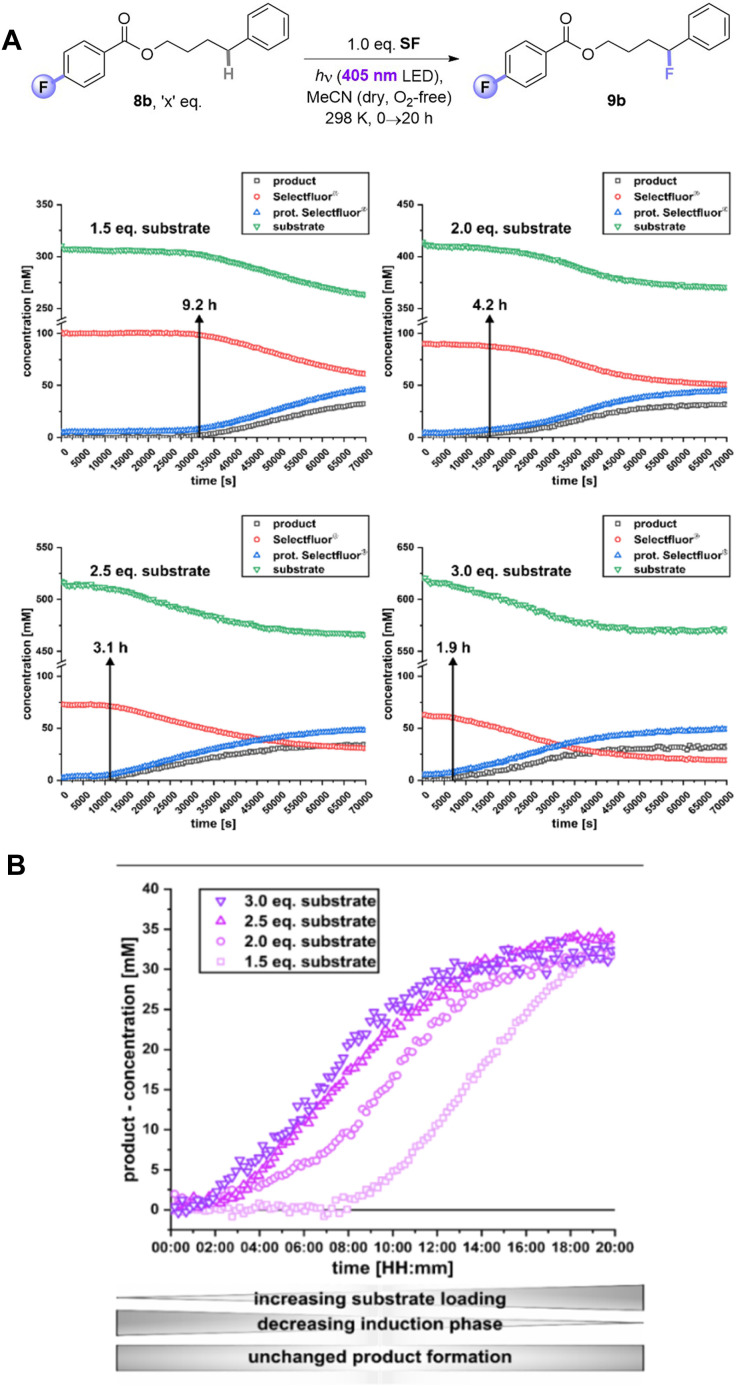
^1^H{^19^F} *In situ* illumination NMR reaction monitoring of PSAux. (A) Kinetic profiles of photosensitization auxiliary method with different amounts of 8b. (B) Detailed comparison of product formation of (9b) depending on different substrate loadings.

Given the inability to determine the origin of the promoting effect of the PSAux's F atom by optical spectroscopy, we turned to examine reaction kinetics. The kinetics of 8b's reaction were examined by a photoirradiation probe allowing on-line LED irradiation within the NMR spectrometer (see ESI for details†).^21^ Consumption of all starting materials and formation of all products could be tracked by time-resolved ^1^H{^19^F} NMR. Samples were compared at the synthetic reaction concentration (0.31 M) to be as representative as possible. SF was not fully dissolved under these conditions, meaning unagitated reactions in NMR tubes could not reach yields/conversion rates as high as the synthetic reactions. Nonetheless, this approach was deemed sufficient to observe relative effects. Interestingly, an induction phase was apparent where scarcely any product forms. When repeating the reaction with different amounts of substrate, a clear trend emerged: the induction phase shortened as the loading of 8b increased. This occurred even in spite of the decreasing (measured) solubility of SF as the overall mixture became more non-polar with increasing [8b]. While the standard reaction with 1.5 eq. of substrate 8b had an induction phase of 9.2 h, when 3 eq. 8b are used it only took 1.9 h until product formation began with a profile typical of a first-order reaction. In summary, by doubling the amount of substrate 8b, the induction phase can be shortened by 79% (Fig. 8A). The rates of product formation within the linear build-up and the overall yield were, however, independent of substrate loading (Fig. 8B). This may be due to (i) the lack of stirring (an inevitable drawback of *in situ* NMR kinetics), meaning only traces of undissolved SF are available for the ongoing reaction, and/or (ii) lower light intensity of the *in situ* irradiation setup (see Table 1, entry 15 *vs.* 17).

We hypothesized that a de-aggregation of SF may rationalize the induction period. Therefore, Diffusion ordered spectroscopy (DOSY) was performed. The volume of 8b hardly changed as a function of its concentration (431.4 Å for 0.62 M and 410.1 Å for 0.31 M; Table 6, entries 1 and 2) and was unchanged in the presence of SF (entries 4–6). In contrast, the volume of SF decreased by 13% from 952.2 Å (entry 3) to 825.3 Å (entry 7) in the presence of 1.5 eq (8b) and by 12% to 836.9 Å with 3 eq. 8b (entry 5) compared to pure SF with the same concentration (952.2 Å, entry 3). This confirmed the role of 8b as a de-aggregation agent. According to the estimated (spherical) molecular volume of SF (see ESI†), such a change suggested a change in aggregation from 3 to 2 SF molecules. We then examined the size of the SF aggregate in the presence of 5b. The volume of 5b alone (413.9 Å) was very similar to that of 8b, consistent with the fact that the fluorine atom is a known bioisostere for H.^6^ However, 5b only de-aggregated SF by 4% (entry 9) – a de-aggregating effect 73% smaller than 8b. Therefore, the F atom in the PSAux clearly plays a role in de-aggregating SF. This could be explained by a halogen–π interaction as suggested by DFT calculations (see ESI†). However, we note that (i) after this initial de-aggregation with 1.5 eq. 8b, the volume of components remain unchanged during the reaction and (ii) doubling the concentration of 8b does not further de-aggregate SF. Thus, while de-aggregation of SF can explain 8b's success *vs.*5b's failure, the substrate concentration benefit on induction period shortening (see Fig. 8) cannot be explained by de-aggregation. Further studies are ongoing in this direction.

**Table 6:** Volumes of different compounds, pure and in reaction, measured by DOSY NMR

Entry	Compound	Composition	*c* (M)	Average volume^a^ with SD (Å^3^)
1	8b	Pure	0.62	431.4 ± 5.37
2			0.31	410.1 ± 7.44
3	SF		0.21^b^	952.2 ± 6.44
4	8b	3.0 eq. (8b) reaction	0.62	425.6 ± 8.83
5	SF		0.21^b^	836.9 ± 17.95
6	8b	1.5 eq. (8b) reaction	0.31	412.5 ± 6.27
7	SF		0.21^b^	825.3 ± 16.40
8	5b	1.5 eq. (5b) reaction	0.31	413.9 ± 13.24
9	SF		0.21^b^	917.5 ± 8.86

a Volumes correspond to the mean values of several measurements carried out at different reaction states. For more details see ESI.

b Weighed in concentration.

**Fig. 9:**
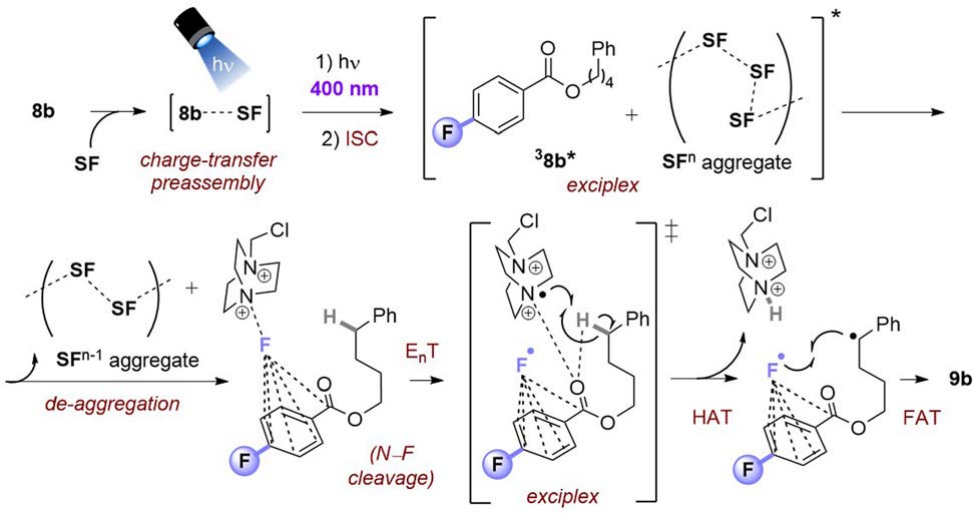
Proposed reaction mechanism for PSAux fluorination by SF de-aggregation.

Taking together the synthetic benefits of the PSAux strategy (handle to direct regioselectivity, remarkable tolerance to O_2_, ability to engage molecules with unprotected functionality), our mechanistic studies suggest that the PSAux plays two key roles. Firstly, the benzoate PSAux engages SF in an intimate charge-transfer preassembly (Fig. 9) that absorbs 400 nm (see Fig. 7). The second key role of the PSAux is to de-aggregate SF (Fig. 9), for which the 4-fluoro atom is especially effective. Moreover, the measured quantum yield of the PSAux reaction for 8b (*Φ* < 0.01) suggested against a radical chain mechanism. The downstream mechanism then resembles that of the PSCat method (Fig. 6) and that proposed in the literature,^9,12^ however we propose subsequent steps all occur within the exciplex/solvent cage. E_n_T from ^3^MFB* to SF leads to N–F bond cleavage. The benzoate then directs the dication radical to the locally most hydridic C(sp^3^)–H bond(s) for HAT that forms a stable (2°, 3°) radical to undergo fluorination. We propose that the beneficial role of de-aggregation is not related to the success of charge-transfer complex formation (see Fig. 7 and the poor reactivity of 5b*vs.*8b), but rather it increases the efficiency of the E_n_T step following photoexcitation.

We cannot rule out that the C=O of the ^3^PSAux* engages in photochemical HAT^22^ with the C(sp^3^)–H bond, but this seems inconsistent with (i) the catalytic lack of reactivities of other *para*-substituted or unsubstituted benzoyl groups (4b, 5b) in PSCat or PSAux fluorinations and (ii) the clear promotion of SF's photodecomposition as seen in the emission spectra (Fig. 4). The ability to conduct photochemical C(sp^3^)–H fluorination reactions in shorter overall reaction times is highly attractive to practitioners, and useful for achieving high yields since SF undergoes a competing (photo)decomposition process.

### Application of benzoyl auxiliary in other E_n_T photochemistry

**Fig. 10:**
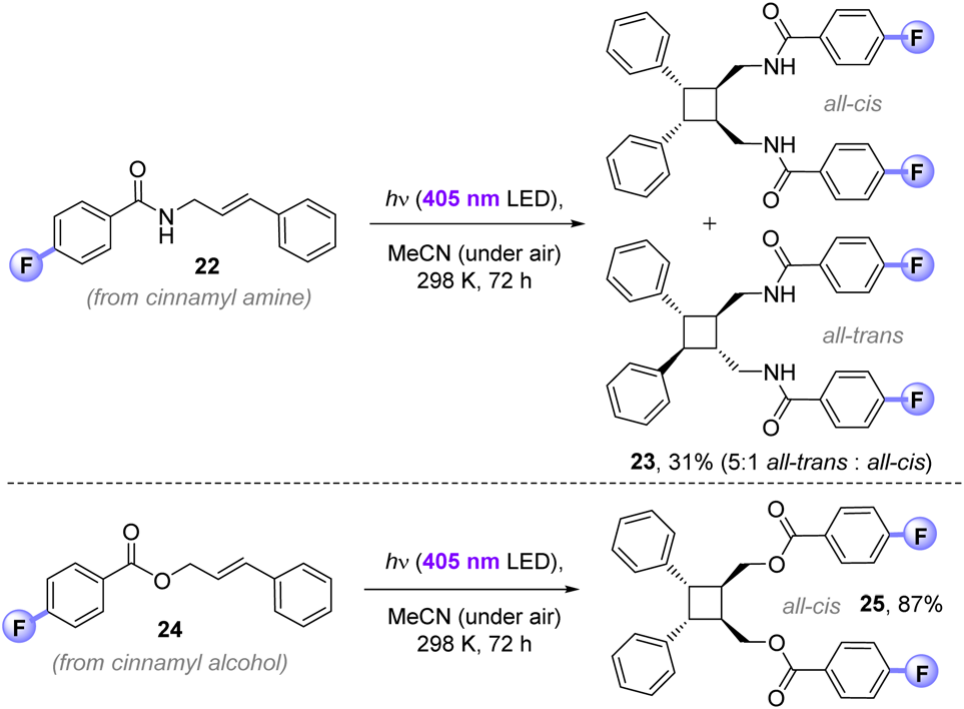
Application of 4-fluorobenzoyl photosensitizer to [2 + 2]-photocycloadditions.

While the main purpose of our study targeted applications to (and understanding of) C(sp^3^)–H fluorinations, we found that the 4-fluorobenzoyl photosensitizer could successfully deliver E_n_T [2 + 2]-photocycloadditions *in air*. The 4-fluorobenzamide derivative of cinnamyl amine (22) afforded cyclobutane 23 (31%) in exclusive head-to-head regioselectivity, but as a mixture of ‘all-*trans*’ and ‘all-*cis*’ diastereomers (5 : 1). In contrast, 4-fluorobenzoylated cinnamyl alcohol 24 underwent [2 + 2]-photocycloaddition to give exclusively the head-to-head, ‘all-trans’ diastereomer of 25 (87%). The previously reported direct UV excitation of cinnamyl alcohol (10 mM in MeCN, 450 W Hg bulb) affords a 2 : 1 mixture of *cis*-/*trans*-isomers and only traces of the cyclobutane (<5%)^23*a*^ Previously, high yield and selectivity for the cyclobutane was only possible by an iterative (2-step) covalent tethering of two cinnamyl alcohols by di-*iso*-propyldichlorosilane (compared to which, 4-fluorobenzoyl chloride is ∼2.5× cheaper) under UV-light and strict degassing.^23^ Herein, this is achieved non-covalently, presumably *via* π-stacking of the photoactive 4-fluorobenzoyl motifs under milder visible-light conditions. Where contemporary photocatalytic [2 + 2]-cycloadditions require degassing due to redox or E_n_T quenching of the photocatalyst by O_2_,^24^ the 4-fluorobenzoyl photosensitization auxiliary provides an alternative strategy than can be run *open to air* (Fig. 10).

## Conclusions

In conclusion, we report the discovery of a highly efficient visible-light harvesting photosensitizer architecture for the direct fluorination of unactivated C(sp^3^)–H bonds. Our new photosensitizing (4-fluorobenzoyl) group was successfully used both as photosensitizing catalyst and photosensitizing auxiliary. Both catalytic and auxiliary methods were successfully applied to the late-stage functionalization of complex molecules. While a reasonably broad substrate scope of fluorinated products was accessible with catalytic method, the photosensitization auxiliary strategy was found to be far superior in scope and practicality. Substrates that for which the catalytic method was inefficient or ineffective were successfully engaged in higher yields and shorter reaction times, furnishing a general platform for photosensitized fluorination that even tolerates free alcohols/amine functionality and works under air. Mechanistic studies reveal the auxiliary's promoting effects derive from two angles, (i) harnessing SF in an intimate charge transfer assembly with SF and (ii) in de-aggregating SF. The 4-fluorobenzoyl auxiliary allows other E_n_T photochemistries to occur in air, where its non-covalent π-stacking interactions alter mechanism and selectivity.

Assemblies between photocatalyst and substrate such as EDA complexes,^20^ hydrogen bonding,^22*c*,*d*^ non-covalent interactions,^25^ and ordering of solvent^26^ attract ever more attention to uncover the next generation of photocatalytic transformations and provide new frontiers in selectivity and efficiency. This study highlights the emerging importance of how changes in aggregation states of photocatalysts can profoundly influence photochemical reaction mechanisms.^27^ Further studies into the structural nature of the charge transfer assembly and the origins of the induction period in photochemical C(sp^3^)–H fluorination reactions are ongoing.

## Data availability

Experimental and computational data are located in the ESI.†

## Author contributions

S. Y. discovered and optimized the photocatalytic and photochemical reaction conditions, synthesized most substrates, synthesized and purified all products, performed synthetic experimental mechanistic studies, measured UV-visible spectra and grew crystals for XRD. W. J. S. conducted all studies on NMR kinetics and DOSY experiments and discovered the induction period and different aggregation states of SF. W. J. S. assisted with 2D NMR structural assignments of complex fluorinated products. X. T. synthesized several substrates and scientifically guided and supervised S. Y. throughout the project. A. S. measured luminescence spectroscopy of SF and MFB. M. J. P. M. performed DFG and TD-DFT calculations and calculated triplet energies. J. P. B. conceptualized the project, guided and supervised S. Y., X. T., M. J. P. M. and A. S. in their contributions. R. M. G. guided and supervised W. J. S., who together wrote the NMR spectroscopic parts of the manuscript and ESI file.† S. Y. and J. P. B. wrote all other sections of the manuscript and ESI file† and addressed peer review additions and corrections. All authors contributed to proofreading the manuscript.

## Conflicts of interest

There are no conflicts to declare.

## Supplementary Material

SC-013-D2SC05735B-s001

SC-013-D2SC05735B-s002
