# Supplementary material for: Benzoates as photosensitization catalysts and auxiliaries in efficient, practical, light-powered direct C(sp3)–H fluorinations
Source: Chem Sci. 2022 Nov 11;13(47):14041–51. doi: 10.1039/d2sc05735b (PMC9728569; doi:10.1039/d2sc05735b)
Supplement: SC-013-D2SC05735B-s001 [file SC-013-D2SC05735B-s001.pdf]

Electronic Supplementary Material (ESI) for Chemical Science.
This journal is © The Royal Society of Chemistry 2022

SUPPLEMENTARY INFORMATION FILE

Benzoates as Photosensitization Catalysts and Auxiliaries in Efficient, Practical, Visible Light-Powered Direct C(sp^3)-H Fluorinations

S. Yakubov,[a] W. J. Stockerl,[a] X. Tian,[a] A. Shahin,[a,b] M. J. P. Mandigma,[a] R. M. Gschwind,[a] J. P. Barham*[a]
(*correspondence: Joshua-Philip.Barham@chemie.uni-regensburg.de)

[a]Fakultät für Chemie und Pharmazie, Universität Regensburg, 93040 Regensburg, Germany.
[b]Chemistry Department, Faculty of Science, Benha University, 13518 Benha, Egypt.

TABLE OF CONTENTS

1 Experimental 2
1.1 General Comments 2
1.2 Optimization reactions 4
2 Synthesis of Substrates 8
2.1 Synthesis of Starting Materials 8
2.2 Synthesis of Fluorinated products via PS Fluorination 26
3 Mechanistic Studies 58
3.1 Radical Trapping 58
3.2 UV-visible Spectroscopy Studies 59
3.3 Luminescence Measurements 63
3.4 Advanced NMR-spectroscopic Investigations 68
3.4.1 General 68
3.4.2 Chemicals 68
3.4.3 Method for quantitative reaction monitoring by *in situ* illumination-NMR spectroscopy 68
3.4.4 Reaction monitoring of photosensitization auxiliary method 69
3.4.4.1 NMR sample preparation 69
3.4.4.2 Reaction monitoring of photosensitization auxiliary method applying different substrate equivalents 69
3.4.5 Reaction monitoring of photocatalytic C(sp^3)-H fluorination 78
3.4.5.1 NMR sample preparation 78
3.4.5.2 Reaction monitoring of photocatalytic C(sp^3)-H fluorination with different catalyst and substrate loadings 79
3.4.6 Method for aggregate-investigations by diffusion ordered spectroscopy (DOSY) in photosensitization auxiliary fluorination 82
3.4.6.1 NMR sample preparation 82
3.4.6.2 DOSY studies of photosensitization auxiliary fluorination using pure compounds at different concentrations and reaction mixtures with different substrate loadings and substrates 83
3.4.6.3 DOSY plots 87
3.4.7 Structural elucidation of **2o** in $CDCl_3$ at 25 °C 99
3.5 Density Functional Theory Computational Details and Cartesian Coordinates 104
3.6 Quantum Yield Measurement 111
4 ^{1}H NMR, ^{13}C NMR and ^{19}F NMR Spectra 115
5 X-Ray Crystallography 263
6 References 273

1 Experimental

1.1 General Comments

All reactions were performed using dried, deoxygenated solvents. Purifications were conducted by column chromatography with silica gel 60 (Macherey Nagel 0.063–0.2 mm) and the solvents were used without further purification. Starting materials that were commercially available were used as received. Syntheses of products were confirmed by comparisons with the literature data where possible and by ^{1}H and ^{19}F NMR spectra. The reactions were followed and pure fractions from column chromatography were detected by Thin Layer Chromatography using silica gel pre-coated aluminum sheets (Macherey Nagel: Alugram Xtra SIL G UV254 Nr. 818333, thickness 0.2 mm). TLC plates were analyzed under a UV-light (254 nm) and by potassium permanganate stain.

NMR-spectra were recorded in $CDCl_3$ or CD_3CN on a Bruker Avance 400 (400 MHz for ^{1}H, 101 MHz for ^{13}C, 376 MHz for ^{19}F, 162 MHz for ^{31}P). For ^{1}H, ^{13}C and ^{19}F chemical shifts are presented in δ-scale as ppm (parts per million) with residual chloroform peak as the internal standard (7.26 ppm for 1H and 77.00 ppm for 13C). For ^{19}F NMR, trifluorotoluene was used as the reference (-63.38 ppm), if not stated, no reference is used. NMR yields were calculated based on ^{19}F NMR using either trifluorotoluene or pentafluorobenzene as an Internal Standard. MestReNova v6.0.2-5475 was used to process NMR spectra. The description of multiplicity that were used is as follows: s = singlet, d = doublet, dd=doublet of doublet, ddd=doublet of doublet of doublet, t = triplet, q = quartet, p=pentet, m = multiplet.

UV-vis absorption spectra were recorded on an Agilent Cary 100 UV/Vis spectrometer (the range of wavelength is 200 nm to 800 nm) and 0.10 mm thick 10 mm × 10 mm quartz cuvettes were used at 25 °C.

HR-MS were recorded at the Central Analytical Department of our University and the spectra were measured on an JOEL AccuTOF GCx instrument for electron ionization (EI), Agilent Q-TOF 6540 UHD instrument for electrospray ionization (ESI) and atmospheric-pressure chemical ionization (APCI).

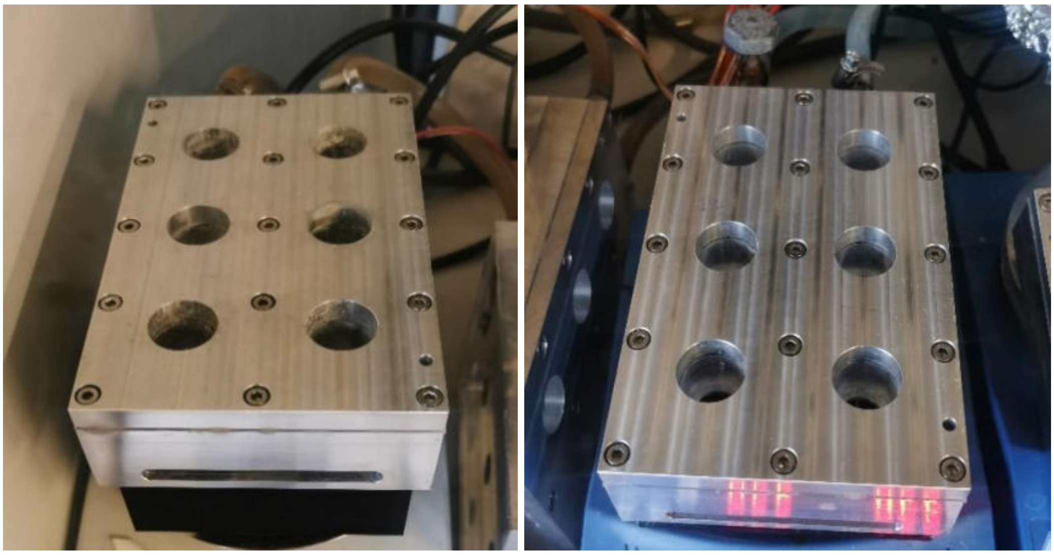

Figure S1. Typical set-up for photochemical reactions using purple LEDs of input power 3.8 W (left) and 0.35 W (right).

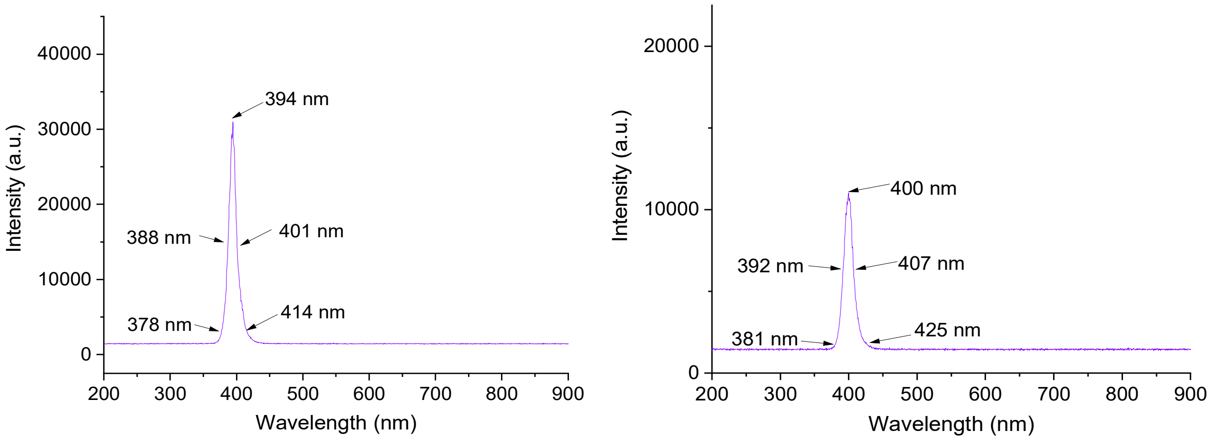

Figure S2. Relative LED intensities of 400 nm LEDs measured at a 30 cm distance directly above the LED. Input power of the higher intensity LED (left) = 3.8 W [LED Engine LZ4-40UB00-00U4 LEDs (λ = 395 nm, 14.8 V, 700 mA)], input power of lower intensity LED (right) = 350 mW. [Edison EDEV-SLC1-03 LEDs (λ = 400 nm, 3.7 V, 700 mA)].

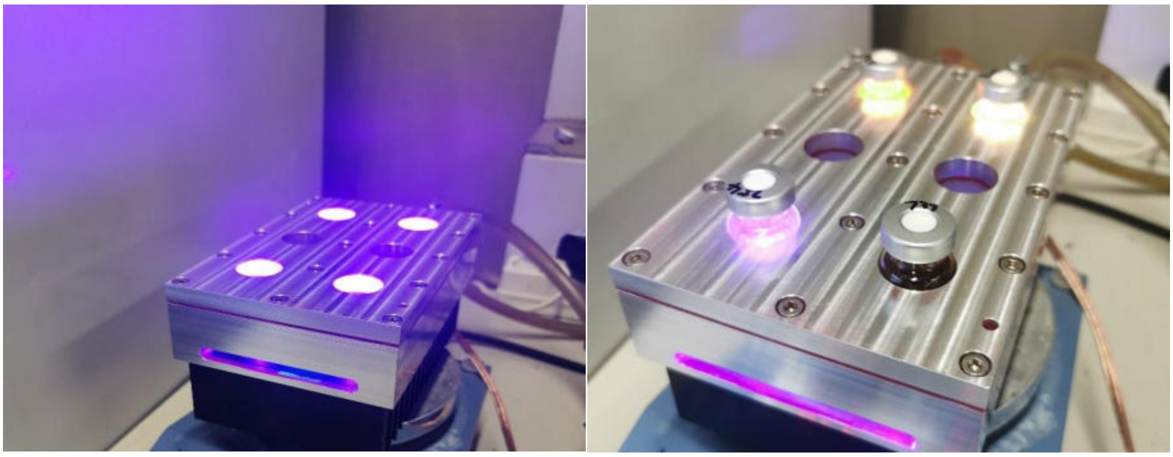

Figure S3. Fluorination reactions were conducted in sealed glass vials by irradiation with the higher intensity 3.8 W input power LED.

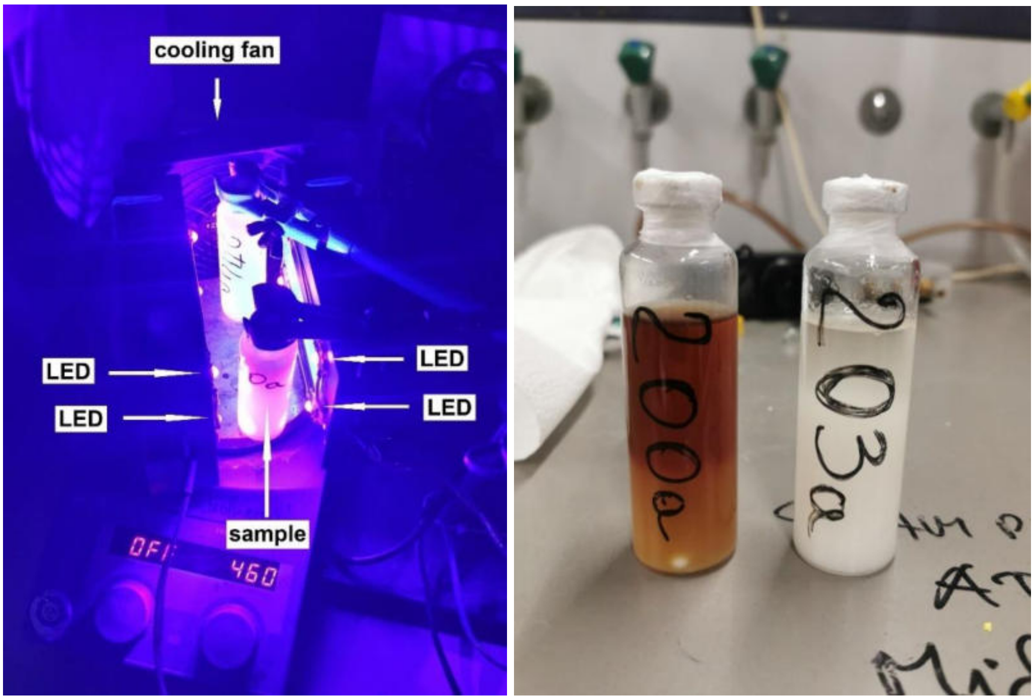

Figure S4. Large scale set-up for photochemical reactions using purple LEDs 3.8 W (left). Gram-scale reaction mixtures of two different substrates after 24 h irradiation (right).

1.2 Optimization reactions

Table S1: Solvent screen.

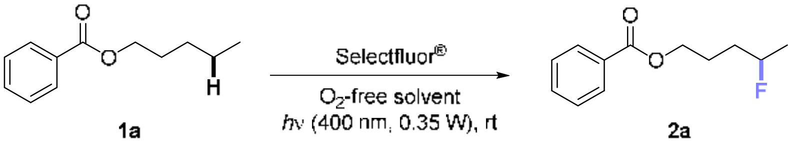

Entry	Solvent	Concentrations of Amyl Benzoate and Selectfluor	Ratio of amyl benzoate and Selectfluor	Duration (h)	NMR yield (%)
1	MeCN+H_2O (4:1)	0.313 M : 0.209 M	1.5 : 1	24	1
2	MeCN+TFA+H_2O (4:1:1)	0.313 M : 0.209 M	1.5 : 1	24	3
3	Dry MeCN+TFA (4:1)	0.313 M : 0.209 M	1.5 : 1	24	29
4	MeCN+H_2O (40:1)	0.313 M : 0.209 M	1.5 : 1	24	2
5	Dry DMA	0.313 M : 0.209 M	1.5 : 1	24	0
6	HFIP	0.313 M : 0.209 M	1.5 : 1	24	0

7	Dry $MeNO_2$	0.313 M : 0.209 M	1.5 : 1	24	0
8	Dry MeCN[a]	0.313 M : 0.209 M	1.5 : 1	48	0
9	Dry MeCN[b]	0.313 M : 0.209 M	1.5 : 1	48	0
10	Dry MeCN	0.313 M : 0.209 M	1.5 : 1	48	42
11	Dry MeCN	0.157 M : 0.105 M	1.5 : 1	48	48
12	Dry MeCN	0.091 M : 0.063 M	1.5 : 1	48	1
13	Dry MeCN	0.209 M : 0.209 M	1 : 1	24	0
14	Dry MeCN	0.105 M : 0.105 M	1 : 1	24	0

[a] Under air with no F/P/T, [b] no light.

Table S2: Comparison of **PScat** and **PSAux**.

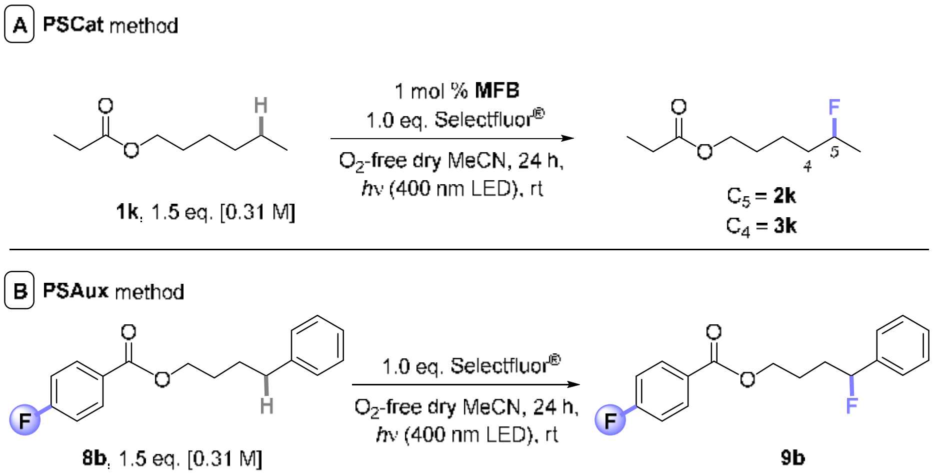

Entry	Deviations from standard conditions	NMR yield[a] of **2k** + **3k** (**PSCat**) (%)	NMR yield[a] of **9b** (**PSAux**) (%)
1	none	57 + 16	75
2	Bubbled with N_2 for 15 min[b]	53 + 11	75
3	Under air	<5	74

[a] NMR yield determined by ^{19}F NMR with trifluorotoluene as IS. [b] Instead of doing three cycles of freeze-pump-thaw.

Table S3: Applicability of the Photosensitization Auxiliary Approach

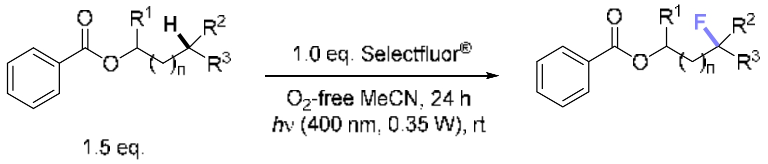

Entry	Substrate	Duration (h)	NMR yield[a] (%)
1	Isoamyl benzoate (**1c**)	24	Traces (**2c**)
2	*n*-butylphenyl benzoate (**5b**)	60	21 (**17**)
3	1-Adamantyl benzoate (**22**)	48	43 (**23**)

[a] NMR yield determined by ^{19}F NMR with trifluorotoluene as IS.

Table S4: Solubility of Selectfluor® (**SF**).

Entry	Solvent	Solubility
1	Toluene	Not soluble
2	*p*-Xylene	Not soluble
3	THF	Not soluble
4	Ethanol	Slightly soluble
5	Acetone	Slightly soluble (less than 0.05 M)
6	DMA	Slightly soluble
7	HFIP	Fairly soluble
8	MeCN	Fairly soluble (up to 0.1 M)
9	$MeNO_2$	Fairly soluble
10	DMF	Fully soluble
11	H_2O	Fully soluble (over 1.0 M)

Table S5: Catalyst optimization reactions.

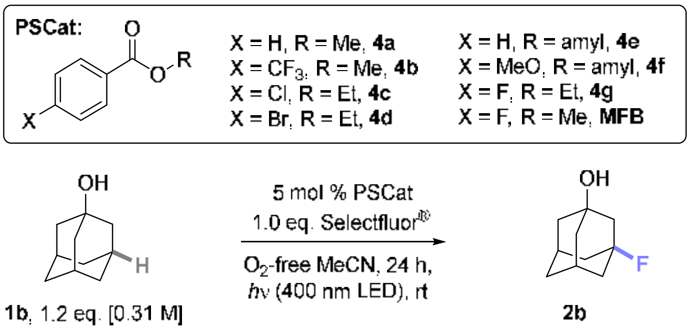

Entry	PSCat	Product (**2b**): yield[a]
1	**4a**	n.d.

2	**4b**	traces
3	**4c**	traces
4	**4d**	traces
5	**4e**	traces
6	**4f**	traces
7	**4g**	44%
8	**MFB**	46%
9	**benzamide**	n.d.
10	**benzonitrile**	n.d.

[a] NMR yield determined by ^{19}F NMR with trifluorotoluene as IS.

Table S6: Control reactions with different **MFB** loading

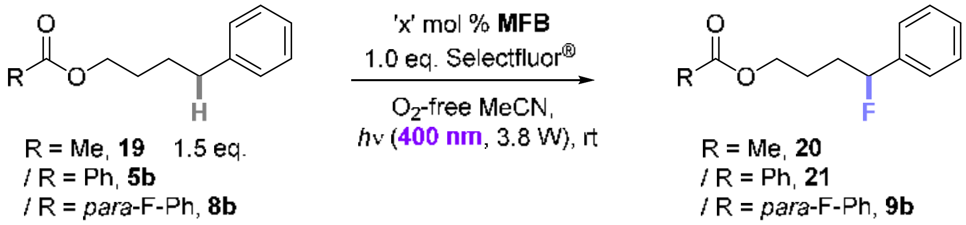

Entry	**R** (Substrate)	**MFB** 'x' mol%	Yield[a] (**Product**)
1	Ph (**5b**)	1	8% (**17**)
2	Me (**19**)	1	19% (**20**)
3	Ph (**5b**)	0	10% (**17**)
4	Me (**19**)	0	14% (**20**)
5	Ph (**5b**)	150	47% (**17**)
6	Me (**19**)	150	30% (**20**)
7	Ph (**5b**)	150[b]	35% (**17**)
8	Me (**19**)	150[b]	26% (**20**)
9	*para*-F-Ph (**8b**)	0	75% (**9b**)
10	*para*-F-Ph (**8b**)	0 [b]	74% (**9b**)
11	Ph (**5b**)	150[c]	19% (**17**)
12	Me (**19**)	150[c]	31% (**20**)
13	*para*-F-Ph (**8b**)	0[c]	23% (**9b**)

[a]NMR yield, based on ^{19}F NMR and trifluorotoluene as IS. [b]under air. [c]Instead of **SF**, NFSI was used as a fluorine source.

2 Synthesis of Substrates

2.1 Synthesis of Starting Materials

General Procedure 1: Esterification

To a solution of Et_3N (1.5 eq.) in DCM (0.2 M), DMAP (0.1 eq.) was added followed by the addition of the alcohol (1.0 eq.). At 0°C, benzoyl chloride (1.2 eq.) was added dropwise to the reaction mixture. The reaction was stirred overnight at room temperature (rt). The solution was quenched with water and extracted 2 times with DCM (20 mL). Combined organic layers were dried over $MgSO_4$ and the filtrate was concentrated *in vacuo*. The residue was purified by column chromatography to yield the desired ester.

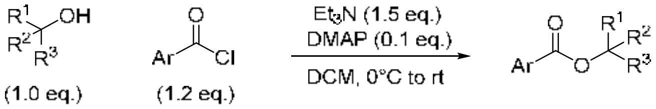

General Procedure 2: Ketone reduction

Sodium borohydride (0.5 eq.) was added to a solution of the ketone in methanol at 0 °C. The reaction was stirred at 0 °C for 3 h. The solvent was removed *in vacuo* and the residue was dissolved in DCM. The solution was washed with a saturated solution of $NaHCO_3$, brine, and the organic phase was dried with $MgSO_4$. After evaporation of the solvent *in vacuo* the residue was purified on silica gel to yield the desired alcohol.

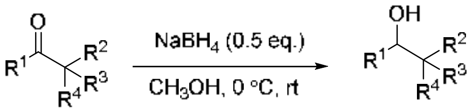

General Procedure 3: Amidation

To a solution of the amine (1.0 eq.) in DCM (0.1 M), 4-fluorobenzoyl chloride (1.1 eq.) and Et_3N (1.1 eq.) were added. The reaction mixture was stirred at rt for 1 hour and washed with water and brine. The organic phase was separated and dried over $MgSO_4$. After filtration and evaporation of the solvent *in vacuo*, the residue was purified over silica gel to obtain the desired amide.

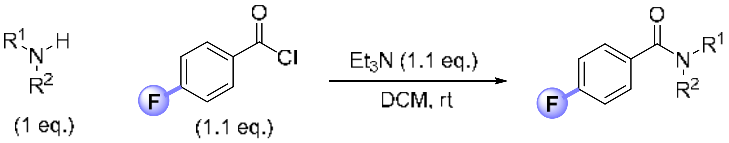

General Procedure 4:

To a solution of the amine (1.0 eq.) in MeCN (1 M), alkyl bromide (2.0 eq.) was added. The reaction mixture was refluxed for 24 h. The solvent was removed under reduced pressure and the residue was dissolved in

water. Solution of KPF_6 (1.1 eq.) in water was added to the mixture and stirred for 30 min. The product was extracted with EtOAc, dried over $MgSO_4$, filtered, and dried *in vacuo* to afford the desired product.

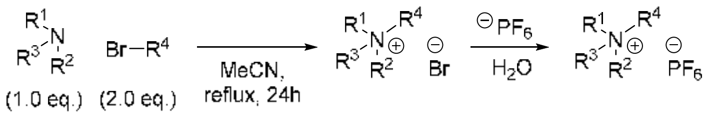

Methyl 4-fluorobenzoate (MFB)

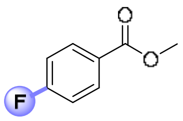

According to **General Procedure 1**. Yield: 2.16 g, 94%; colorless oil; ^{1}H NMR (400 MHz, $CDCl_3$) δ 8.03 (dd, *J* = 8.9, 5.5 Hz, 2H), 7.08 (t, *J* = 8.7 Hz, 2H), 3.89 (s, 3H) ppm; ^{13}C NMR (101 MHz, $CDCl_3$) δ 167.0, 166.1, 164.4, 132.1 (d, *J* = 9.3 Hz), 126.4 (d, *J* = 3.0 Hz), 115.5, 115.3, 52.1 ppm; ^{19}F NMR (376 MHz, CDCl3) δ -106.3 ppm; HRMS (EI) (*m/z*) $[M]^+$: exact mass calc. for $C_8H_7FO_2$: 154.0430, found: 154.0428.
Data are consistent with the literature.[1]

(3*s*,5*s*,7*s*)-Adamantan-1-yl benzoate (26)

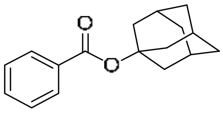

According to **General Procedure 1**. Column chromatography was conducted using 5% EtOAc in *n*-pentane. Yield: 1.80 g, 87%; white solid; ^{1}H NMR (400 MHz, $CDCl_3$) δ 8.20 – 8.13 (m, 2H), 7.73 – 7.63 (m, 1H), 7.53 (dd, *J* = 10.7, 4.9 Hz, 2H), 2.14 (s, 3H), 1.71 (d, *J* = 2.7 Hz, 6H), 1.66 – 1.56 (m, 6H) ppm; ^{13}C NMR (101 MHz, $CDCl_3$) δ 162.3, 134.5, 130.6, 128.9, 68.2, 45.3, 36.1, 30.7 ppm; HRMS (EI) (*m/z*) $[M]^+$: exact mass calc. for $C_{17}H_{20}O_2$: 256.1463, found: 256.1451.
Data are consistent with the literature.[2]

4-Methyl-*N*-pentylbenzenesulfonamide (7)

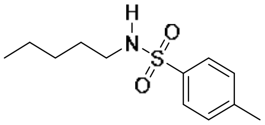

To a solution of amylamine (435.0 mg, 5.00 mmol, 1.0 eq.) in DCM, p-toluenesulfonyl chloride (1.045 g, 5.50 mmol, 1.1 eq.) and Et_3N (555.0 mg, 5.50 mmol, 1.1 eq.) were added at 0 °C. The mixture was stirred for 5 minutes at 0 °C and diluted with water and extracted with DCM. The organic phase was washed with water and brine and dried over $MgSO_4$. The mixture was filtered and concentrated *in vacuo* to obtain 4-methyl-*N*-pentylbenzenesulfonamide. Purification was conducted by column chromatography using 20% EtOAc in *n*-pentane.

Yield: 1.11 g, 92%; slightly yellow oil; ^{1}H NMR (400 MHz, $CDCl_3$) δ 7.75 (d, J = 8.3 Hz, 2H), 7.30 (d, J = 8.0 Hz, 2H), 4.67 (s, 1H), 2.91 (dd, J = 12.7, 6.8 Hz, 2H), 2.42 (s, 3H), 1.52 – 1.34 (m, 2H), 1.27 – 1.16 (m, 4H), 0.81 (t, J = 6.9 Hz, 3H) ppm; ^{13}C NMR (101 MHz, $CDCl_3$) δ 143.2, 137.0, 130.2, 129.6, 127.1, 127.0, 43.2, 29.1, 28.6, 22.1, 21.5, 13.8 ppm; HRMS (ESI) (*m/z*) $[M+H]^+$: exact mass calc. for $C_{12}H_{19}NO_2S$: 242.1209, found: 242.1213.
Data are consistent with the literature.[3]

Pentyl 4-methylbenzenesulfonate (1f)

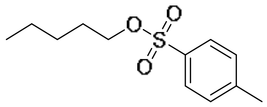

To a stirred solution of alkyl alcohol (5.00 mmol, 1.0 eq.) and Et_3N (7.50 mmol, 1.5 eq.) in DCM (25 mL), p-toluenesulfonyl chloride (6.00 mmol, 1.2 eq.) was added dropwise at 0 °C. The mixture was slowly warmed to rt with continue stirring for 10 hours. The reaction mixture was diluted with saturated sodium bicarbonate (10 mL) and extracted with DCM (2 x 20 mL). The combined organic layer was washed with brine (2 x 10 mL), dried over $MgSO_4$, and concentrated *in vacuo*. Purification was conducted by column chromatography using 5% EtOAc in *n*-pentane.

Yield: 1.06 g, 88%; colorless oil; ^{1}H NMR (400 MHz, $CDCl_3$) δ 7.77 (d, J = 8.3 Hz, 2H), 7.33 (d, J = 8.0 Hz, 2H), 4.01 (t, J = 6.5 Hz, 2H), 2.43 (s, 3H), 1.70 – 1.53 (p, J = 7.3 Hz, 2H), 1.35 – 1.11 (m, 4H), 0.84 (t, J = 7.1 Hz, 3H) ppm; ^{13}C NMR (101 MHz, $CDCl_3$) δ 144.6, 133.2, 129.8, 127.8, 70.7, 28.5, 27.4, 22.0, 21.6, 13.8 ppm; HRMS (ESI) (*m/z*) $[M+NH_4]^+$: exact mass calc. for $C_{12}H_{18}O_3S$: 260.1320, found: 260.1320.
Data are consistent with the literature.[4]

Isopentyl benzoate (1c)

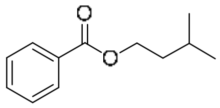

According to **General Procedure 1**. Yield: 1.77 g, 92%; colorless oil; ^{1}H NMR (400 MHz, $CDCl_3$) δ 8.08 – 8.01 (m, 2H), 7.59-7.50 (m, 1H), 7.43 (t, J = 7.6 Hz, 2H), 4.36 (t, J = 6.8 Hz, 2H), 1.80 (h, J = 13.5 Hz, 1H), 1.67 (q, J = 6.8 Hz, 2H), 0.98 (d, J = 6.6 Hz, 6H) ppm; ^{13}C NMR (101 MHz, $CDCl_3$) δ 166.7, 132.8, 130.5, 129.5, 128.3, 63.6, 37.4, 25.2, 22.5 ppm; HRMS (ESI) (*m/z*) $[M+H]^+$: exact mass calc. for $C_{12}H_{16}O_2$: 193.1223, found: 193.1227.
Data are consistent with the literature.[5]

Pentyl 4-methoxybenzoate (27)

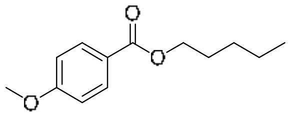

According to **General Procedure 1**. Yield: 2.00 g, 90%; colorless oil; ^{1}H NMR (400 MHz, $CDCl_3$) δ 7.99 (d, *J* = 8.9 Hz, 2H), 6.91 (d, *J* = 8.9 Hz, 2H), 4.28 (t, *J* = 6.7 Hz, 2H), 3.85 (s, 3H), 1.80-1.70 (m, 2H), 1.46 – 1.32 (m, 4H), 0.92 (t, *J* = 7.1 Hz, 3H) ppm; ^{13}C NMR (101 MHz, $CDCl_3$) δ 166.4, 163.2, 131.5, 123.0, 113.5, 64.8, 55.4, 28.5, 28.2, 22.4, 14.0 ppm; HRMS (EI) (*m/z*) $[M]^+$: exact mass calc. for $C_{13}H_{18}O_3$: 222.1256, found: 222.1252.
Data are consistent with the literature.[6]

4-Phenylbutyl benzoate (5b)

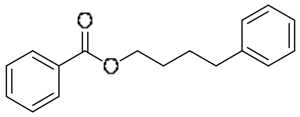

According to **General Procedure 1**. Yield: 2.30 g, 91%; colorless oil; ^{1}H NMR (400 MHz, $CDCl_3$) δ 8.06 (dd, *J* = 8.1, 1.0 Hz, 2H), 7.62 – 7.53 (m, 1H), 7.45 (t, *J* = 7.6 Hz, 2H), 7.35 – 7.27 (m, 2H), 7.25 – 7.14 (m, 3H), 4.36 (t, *J* = 6.2 Hz, 2H), 2.71 (t, *J* = 7.1 Hz, 2H), 1.93 – 1.73 (m, 4H) ppm; ^{13}C NMR (101 MHz, $CDCl_3$) δ 166.6, 142.0, 132.8, 130.4, 129.5, 128.4, 128.4, 128.3, 128.3, 125.9, 64.8, 35.5, 28.3, 27.8 ppm; HRMS (ESI) (*m/z*) $[M+NH_4]^+$: exact mass calc. for $C_{17}H_{18}O_2$: 272.1645, found: 272.1654.
Data are consistent with the literature.[7]

2-Chloro-1-phenyldodecyl diphenylphosphinate (1g)

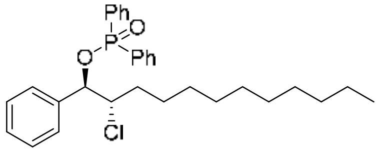

The chlorinated ketone was prepared using NCS according to literature procedures.[8] To a solution of α-chlorinated ketone (3.00 mmol, 1.0 eq.) in 20 mL MeOH, $NaBH_4$ (57.0 mg, 0.5 eq.) was added in 2 portions, at 0 °C. The reaction mixture was stirred at rt for 2 h. Solvent was removed under vacuum and 10 mL H_2O was added to the residue. The resulting mixture was then extracted with DCM (10 mL × 3). The organic layers were combined, dried over $MgSO_4$, and concentrated *in vacuo*. The residue was treated with Et_3N (4.50 mmol, 1.5 eq.), 4-dimethylamino pyridine (0.30 mmol, 0.1 eq.), 20 mL DCM and diphenylphosphinic chloride (3.60 mmol, 1.2 eq.), at 0 °C. The reaction mixture was stirred at rt for 18 h. Solvent was removed *in vacuo* and the residue was purified by column chromatography using 50% EtOAc in pentane to give the desired product.

Yield: 1.09 g, 73%; colorless oil; ^{1}H NMR (400 MHz, $CDCl_3$) δ 7.91-7.82 (m, 2H), 7.62-7.50 (m, 3H), 7.49-7.42 (m, 2H), 7.40-7.33 (m, 1H), 7.30-7.20 (m, 2H), 7.46 (dd, J = 9.6, 6.0 Hz, 1H), 4.28-4.20 (m, 1H), 1.78-1.65 (m, 1H), 1.55-1.42 (m, 2H), 1.34-1.11 (m, 15H), 0.87 (t, J = 6.8, 3H) ppm; ^{13}C NMR (100 MHz, $CDCl_3$) (the major isomer) δ 137.1 (d, J = 2.5 Hz), 132.2 (d, J = 2.7 Hz), 132.0 (d, J = 2.7 Hz), 131.86 (d, J = 10.4 Hz), 131.76 (d, J = 138.9 Hz), 131.67 (d, J = 10.3 Hz), 131.2 (d, J = 133.7 Hz), 128.54, 128.49 (d, J = 13.2 Hz), 128.139, 128.136 (d, J = 13.2 Hz), 127.6, 79.7 (d, J = 5.8 Hz), 65.4 (d, J = 5.4 Hz), 33.5, 31.9, 29.57, 29.51, 29.38, 29.32, 28.9, 26.3, 22.7, 14.1 ppm; ^{31}P NMR (162 MHz, $CDCl_3$) δ 33.0 ppm; HRMS (ESI) (*m/z*) $[M+H]^+$: exact mass calc. for $C_{30}H_{39}ClO_2P$: 497.2371, found 497.2372.

Data are consistent with the literature.[9]

Methyl (*R*)-4-((5*S*,8*R*,9*S*,10*S*,13*R*,14*S*,17*R*)-10,13-dimethyl-3,7,12-trioxohexadecahydro-1*H*-cyclopenta[a]phenanthren-17-yl)pentanoate (1o)

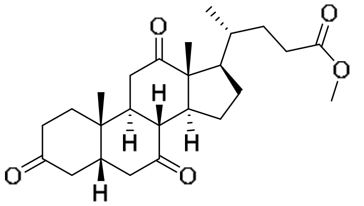

Prepared according to the literature procedure.[10] To a previously stirred mixture of dehydrocholic acid 1c (2.00 g, 5.00 mmol, 1.0 eq.) and Cs_2CO_3 (2.02 g, 6.20 mmol 1.2 eq.) in 10 mL DMF, methyl Iodide (4.05 g, 28.50 mmol, 5.7 eq.) was added. The mixture was stirred at rt for 24 h. The precipitate obtained after the addition of water (40 mL) was filtered and dried. Yield: 1.90 g, 92%; white solid; ^{1}H NMR (400 MHz, $CDCl_3$) δ 3.65 (d, J = 1.1 Hz, 3H), 2.96 – 2.75 (m, 3H), 2.44 – 2.16 (m, 8H), 2.12 (dd, J = 12.8, 5.9 Hz, 2H), 2.05 – 1.91 (m, 4H), 1.88 – 1.77 (m, 2H), 1.59 (tt, J = 11.6, 5.9 Hz, 1H), 1.41 – 1.21 (m, 7H), 1.05 (s, 3H), 0.83 (d, J = 6.5 Hz, 3H) ppm; ^{13}C NMR (101 MHz, $CDCl_3$) δ 211.9, 209.0, 208.7, 174.5, 56.9, 51.7, 51.5, 49.0, 46.8, 45.6, 45.5, 44.9, 42.8, 38.6, 36.4, 36.0, 35.5, 35.2, 31.2, 30.4, 27.6, 25.1, 21.9, 18.6, 11.8 ppm; HRMS (EI) (*m/z*) $[M]^+$: exact mass calc. for $C_{25}H_{36}O_5$: 416.2563, found: 416.2554.

Data are consistent with the literature.[11]

1-Cyclohexyl-2-methoxy-2-oxo-1-phenylethyl 4-fluorobenzoate (8i)

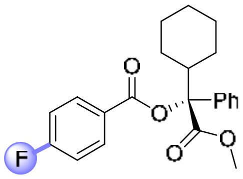

Prepared from 2-cyclohexyl-2-hydroxy-2-phenylacetic acid in two steps. Step 1: To a mixture of 2-cyclohexyl-2-hydroxy-2-phenylacetic acid (1.24 g, 5.30 mmol, 1.0 eq.) and potassium carbonate (1.83 g, 13.25 mmol, 2.5 eq.) in 10 mL DMF, methyl iodide (2.27 g, 16.00 mmol, 3.0 eq.) was added at rt. The mixture was stirred for 2 h and poured into water and extracted with hexane three times. The organic phase was

dried over $MgSO_4$ and concentrated *in vacuo* to give a crude product. The crude product was used for the next step without further purification.

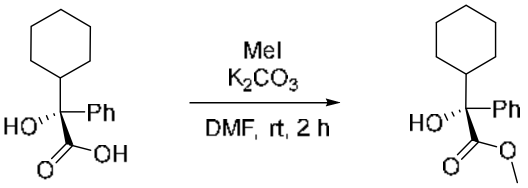

Step 2: To the mixture of the crude product of the first step, sodium hydride (317.0 mg (60% NaH), 7.92 mmol, 1.5 eq.) in 10 mL DMF, 4-fluorobenzoyl chloride (0.75 mL, 6.36 mmol, 1.2 eq.) was added slowly at rt. The mixture was stirred for 18 h and poured into water and extracted with hexane three times. The organic phase was dried over $MgSO_4$ and the solvent was concentrated *in vacuo*. Purification by column chromatography with 5% EtOAc in *n*-pentane provided the desired product.

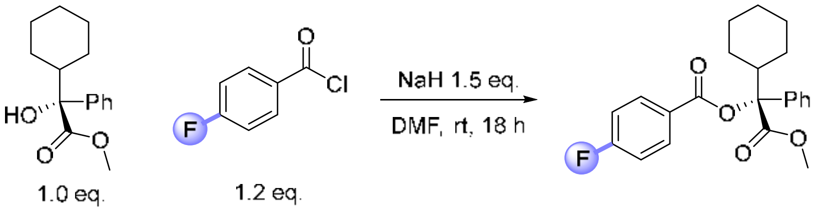

Yield: 1.52 g, 82%; white solid; IR (neat) ν (cm^{-1}): 2937, 2855, 1729, 1602, 1505, 1449, 1412, 1282, 1237, 1207, 1088, 1025, 853, 767, 704; ^1H NMR (400 MHz, $CDCl_3$) δ 8.19 – 8.10 (m, 2H), 7.58 – 7.52 (m, 2H), 7.39 – 7.27 (m, 3H), 7.21 – 7.14 (m, 2H), 3.75 (s, 3H), 2.38 (tt, J = 12.0, 2.8 Hz, 1H), 1.87 (d, J = 12.6 Hz, 1H), 1.74 (dd, J = 9.5, 3.4 Hz, 3H), 1.63 (d, J = 12.8 Hz, 1H), 1.23 (m, 2H), 1.08 – 0.96 (m, 2H), 0.95 – 0.84 (m, 1H) ppm; ^{13}C NMR (101 MHz, $CDCl_3$) δ 170.5 (s), 167.2 (s), 164.7 (s), 164.0 (s), 137.0 (s), 132.4 (d, J = 9.3 Hz), 127.8 (s), 127.6 (s), 126.3 (d, J = 3.0 Hz), 126.2 (s), 115.7 (d, J = 22.0 Hz), 86.9 (s), 52.3 (s), 46.8 (s), 27.8 (d, J = 76.7 Hz), 26.4 (d, J = 4.8 Hz), 26.1 (s) ppm; ^{19}F NMR (377 MHz, $CDCl_3$) δ -105.4 ppm; HRMS (ESI) (*m/z*) $[M+Na]^+$: exact mass calc. for $C_{22}H_{23}FO_4$: 393.1478, found: 393.1476.

2-Butoxyethyl 4-fluorobenzoate (8a)

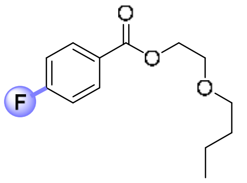

According to **General Procedure 1**. Yield: 2.11 g, 88%; colorless oil; IR (neat) ν (cm^{-1}): 2959, 2937, 2870, 1722, 1603, 1510, 1457, 1413, 1383, 1267, 1226, 1155, 1088, 1014, 980, 905, 854, 768, 690; ^1H NMR (400 MHz, $CDCl_3$) δ 8.12 – 8.01 (m, 2H), 7.15 – 7.03 (m, 2H), 4.44 (dd, J = 5.5, 4.2 Hz, 2H), 3.77 – 3.70 (m, 2H), 3.50 (t, J = 6.6 Hz, 2H), 1.62 – 1.51 (m, 2H), 1.43 – 1.29 (m, 2H), 0.90 (t, J = 7.4 Hz, 3H) ppm; ^{13}C NMR (101 MHz, $CDCl_3$) δ 167.0 (s), 165.6 (s), 164.5 (s), 132.2 (d, J = 9.3 Hz), 126.4 (d, J = 3.0 Hz), 115.4 (d, J

= 21.9 Hz), 71.1 (s), 68.5 (s), 64.3 (s), 31.6 (s), 19.2 (s), 13.8 (s) ppm; ^{19}F NMR (377 MHz, $CDCl_3$) δ -106.3 ppm; HRMS (ESI) (*m/z*) $[M]^+$: exact mass calc. for $C_{13}H_{17}FO_3$: 240.1162, found: 240.1167.

4-Phenylbutyl 4-fluorobenzoate (8b)

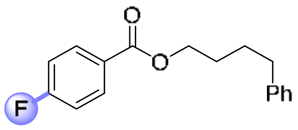

According to **General Procedure 1**. Yield: 2.48 g, 91%; colorless oil; IR (neat) ν (cm^{-1}): 3064, 3027, 2941, 2863, 1715, 1603, 1506, 1454, 1409, 1267, 1237, 1152, 1114, 1014, 950, 854, 768, 750, 701; ^{1}H NMR (400 MHz, $CDCl_3$) δ 8.12 – 8.02 (m, 2H), 7.36 – 7.27 (m, 2H), 7.25 – 7.17 (m, 3H), 7.16 – 7.07 (m, 2H), 4.35 (dd, *J* = 8.4, 4.0 Hz, 2H), 2.71 (t, *J* = 7.1 Hz, 2H), 1.88 – 1.74 (m, 4H) ppm; ^{13}C NMR (101 MHz, $CDCl_3$) δ 167.0 (s), 165.6 (s), 164.4 (s), 142.0 (s), 132.1 (d, *J* = 9.3 Hz), 128.4 (d, *J* = 2.6 Hz), 126.7 (d, *J* = 3.0 Hz), 125.9 (s), 115.5 (d, *J* = 21.9 Hz), 65.0 (s), 35.5 (s), 28.0 (d, *J* = 51.5 Hz) ppm; ^{19}F NMR (377 MHz, $CDCl_3$) δ -106.4 ppm; HRMS (EI) (*m/z*) $[M]^+$: exact mass calc. for $C_{17}H_{17}FO_2$: 272.1213, found: 272.1212.

1-Phenyldodecyl 4-fluorobenzoate (8c)

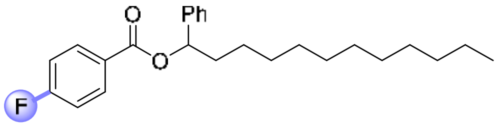

Prepared from 1-phenyldodecan-1-one in two steps. Step 1: According to **General Procedure 2**. Step 2: According to **General Procedure 1**.

Yield: 3.27 g, 85%; colorless oil; IR (neat) ν (cm^{-1}): 2922, 2855, 1722, 1602, 1505, 1457, 1412, 1267, 1151, 1110, 954, 853, 767, 700; ^{1}H NMR (400 MHz, $CDCl_3$) δ 8.17 – 8.07 (m, 2H), 7.46 – 7.27 (m, 5H), 7.16 – 7.07 (m, 2H), 5.99 (dd, *J* = 7.5, 6.3 Hz, 1H), 2.16 – 1.85 (m, 2H), 1.46 – 1.23 (m, 18H), 0.90 (t, *J* = 6.9 Hz, 3H) ppm; ^{13}C NMR (101 MHz, $CDCl_3$) δ 167.0 (s), 164.9 (s), 164.5 (s), 140.8 (s), 132.1 (d, *J* = 9.3 Hz), 128.5 (s), 127.9 (s), 126.8 (d, *J* = 3.0 Hz), 126.4 (s), 115.4 (d, *J* = 22.0 Hz), 36.5 (s), 31.9 (s), 29.6 (s), 29.5 (d, *J* = 9.2 Hz), 29.3 (d, *J* = 1.9 Hz), 25.5 (s), 22.7 (s), 14.1 (s) ppm; ^{19}F NMR (377 MHz, $CDCl_3$) δ -106.3 ppm; HRMS (EI) (*m/z*) $[M]^+$: exact mass calc. for $C_{25}H_{33}FO_2$: 384.2465, found: 384.2466.

1-Phenyldodecan-1-ol (5c)

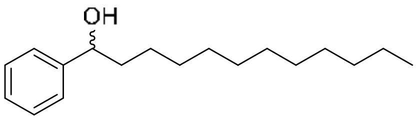

According to **General Procedure 2**. Yield: 2.60 g, 99%; colorless oil; ^{1}H NMR (400 MHz, $CDCl_3$) δ 7.33 – 7.15 (m, 5H), 4.57 (dd, *J* = 7.4, 5.9 Hz, 1H), 1.79 – 1.54 (m, 2H), 1.39 – 1.10 (m, 19H), 0.81 (t, *J* = 6.8 Hz, 3H) ppm; ^{13}C NMR (101 MHz, $CDCl_3$) δ 144.9, 128.4, 127.4, 125.9, 74.7, 39.1, 31.9, 29.6, 29.6, 29.6, 29.5,

29.5, 29.3, 25.8, 22.7, 14.1 ppm; HRMS (EI) (*m/z*) $[M]^+$: exact mass calc. for $C_{18}H_{30}O$: 262.2297, found: 262.2293.

Data are consistent with the literature.[12]

(1*R*,2*S*,5*R*)-2-Isopropyl-5-methylcyclohexyl 4-fluorobenzoate (8d)

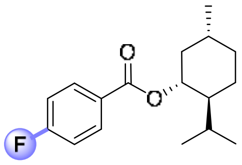

According to **General Procedure 1**. Yield: 2.48 g, 89%; colorless oil; ^{1}H NMR (400 MHz, $CDCl_3$) δ 8.14 – 7.98 (m, 2H), 7.17 – 7.04 (m, 2H), 4.92 (td, *J* = 10.9, 4.4 Hz, 1H), 2.20 – 2.06 (m, 1H), 1.94 (dtd, *J* = 14.0, 7.0, 2.7 Hz, 1H), 1.79 – 1.66 (m, 2H), 1.64 – 1.45 (m, 2H), 1.19 – 1.03 (m, 2H), 0.92 (dd, *J* = 6.8, 4.1 Hz, 6H), 0.79 (d, *J* = 7.0 Hz, 3H) ppm; ^{13}C NMR (101 MHz, $CDCl_3$) δ 166.9 (s), 165.1 (s), 164.3 (s), 132.0 (d, *J* = 9.2 Hz), 127.0 (d, *J* = 3.0 Hz), 115.3 (d, *J* = 21.9 Hz), 75.0 (s), 47.2 (s), 40.9 (s), 34.3 (s), 31.4 (s), 26.5 (s), 23.6 (s), 22.0 (s), 20.7 (s), 16.5 (s) ppm; ^{19}F NMR (377 MHz, $CDCl_3$) δ -106.7 ppm; HRMS (ESI) (*m/z*) $[M+Na]^+$: exact mass calc. for $C_{17}H_{23}FO_2$: 301.1580, found: 301.1569.
Data are consistent with the literature.[13]

3-Methyl-1-phenylbutyl 4-fluorobenzoate (8e)

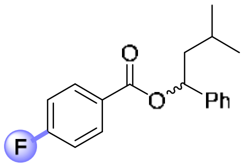

Prepared from 3-methyl-1-phenylbutan-1-one in two steps. Step 1: According to **General Procedure 2**. Step 2: According to **General Procedure 1**.

Yield: 2.50 g, 87%; colorless oil; ^{1}H NMR (400 MHz, $CDCl_3$) δ 8.24 – 8.07 (m, 2H), 7.57 – 7.29 (m, 5H), 7.20 – 7.00 (m, 2H), 6.15 (dd, *J* = 8.8, 5.3 Hz, 1H), 2.18 – 1.99 (m, 1H), 1.87 – 1.67 (m, 2H), 1.05 (dd, *J* = 8.5, 6.4 Hz, 6H) ppm; ^{13}C NMR (101 MHz, $CDCl_3$) δ 167.0 (s), 164.9 (s), 164.5 (s), 141.1 (s), 132.2 (d, *J* = 9.3 Hz), 128.6 (s), 128.0 (s), 126.8 (d, *J* = 3.0 Hz), 126.5 (s), 115.5 (d, *J* = 22.0 Hz), 75.4 (s), 45.7 (s), 24.9 (s), 22.9 (s), 22.4 (s) ppm; ^{19}F NMR (377 MHz, $CDCl_3$) δ -106.1 ppm; HRMS (EI) (*m/z*) $[M]^+$: exact mass calc. for $C_{18}H_{19}FO_2$: 286.1369, found: 286.1366.

Data are consistent with the literature.[14]

3-Methyl-1-phenylbutan-1-ol (5e)

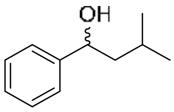

According to **General Procedure 2**. Yield: 1.62 g, 99%; colorless oil; ^{1}H NMR (400 MHz, $CDCl_3$) δ 7.36 – 7.14 (m, 5H), 4.63 (dd, *J* = 11.6, 6.2 Hz, 1H), 2.66 – 2.17 (m, 1H), 1.86 – 1.32 (m, 3H), 0.89 (dd, *J* = 6.4, 3.4 Hz, 6H) ppm; ^{13}C NMR (101 MHz, $CDCl_3$) δ 145.3, 128.4, 127.4, 125.9, 72.6, 48.3, 24.7, 23.1, 22.3 ppm; HRMS (EI) (*m/z*) $[M]^+$: exact mass calc. for $C_{11}H_{16}O$: 164.1201, found: 164.1197.

Data are consistent with the literature.[15]

((1*R*,2*R*,4*S*)-Bicyclo[2.2.1]heptan-2-yl)methyl 4-fluorobenzoate (8f)

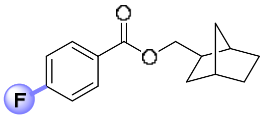

According to **General Procedure 1**. Yield: 2.30 g, 93%; colorless oil; IR (neat) ν (cm^{-1}): 2955, 2929, 2870, 1714, 1602, 1505, 1453, 1412, 1371, 1267, 1181, 1151, 1110, 1039, 961, 916, 853, 805, 767, 685; ^{1}H NMR (400 MHz, $CDCl_3$) δ 8.14 – 7.96 (m, 2H), 7.15 – 7.03 (m, 2H), 4.38 – 4.00 (m, 2H), 2.34 – 2.28 (m, 1H), 2.23 (dd, *J* = 9.9, 5.4 Hz, 1H), 1.99 – 1.70 (m, 1H), 1.63 – 1.48 (m, 2H), 1.48 – 1.28 (m, 3H), 1.27 – 1.07 (m, 2H), 0.77 (ddd, *J* = 12.3, 5.0, 2.3 Hz, 1H) ppm; ^{13}C NMR (101 MHz, $CDCl_3$) δ 166.9 (s), 165.6 (d, *J* = 10.7 Hz), 164.4 (s), 132.0 (d, *J* = 9.3 Hz), 126.8 (d, *J* = 2.9 Hz), 115.4 (d, *J* = 21.9 Hz), 68.3 (s), 67.0 (s), 41.0 (s), 39.7 (s), 38.8 (s), 38.5 (s), 38.4 (s), 36.7 (s), 36.2 (s), 35.2 (s), 34.0 (s), 33.5 (s), 29.8 (s), 29.7 (s), 28.8 (s), 22.6 (s) ppm; ^{19}F NMR (377 MHz, $CDCl_3$) δ -106.5 ppm; HRMS (ESI) (*m/z*) $[M+NH_4]^+$: exact mass calc. for $C_{15}H_{17}FO_2$: 266.1556, found: 266.1552.

6,7,8,9-Tetrahydro-5*H*-benzo[7]annulen-5-yl 4-fluorobenzoate (8g)

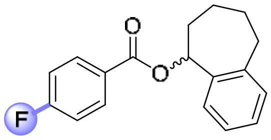

Prepared from 6,7,8,9-tetrahydro-5*H*-benzo[7]annulen-5-one in two steps. Step 1: According to **General Procedure 2**. Step 2: According to **General Procedure 1**.

Yield: 2.53 g, 89%; colorless oil; IR (neat) ν (cm^{-1}): 2929, 2855, 1714, 1602, 1505, 1446, 1408, 1360, 1267, 1151, 1110, 1013, 972, 924, 853, 805, 760, 685; ^{1}H NMR (400 MHz, $CDCl_3$) δ 8.27 – 8.11 (m, 2H), 7.48 – 7.38 (m, 1H), 7.25 – 7.10 (m, 5H), 6.33 – 6.19 (m, 1H), 3.18 – 3.06 (m, 1H), 2.87 (ddd, *J* = 14.2, 7.9, 3.1 Hz, 1H), 2.26 – 2.08 (m, 2H), 2.08 – 1.90 (m, 2H), 1.80 (ddd, *J* = 9.7, 9.0, 3.2 Hz, 2H) ppm; ^{13}C NMR (101 MHz, $CDCl_3$) δ 167.0 (s), 164.5 (s), 141.6 (s), 140.0 (s), 132.2 (d, *J* = 9.3 Hz), 129.9 (s), 127.8 (s), 127.0 – 125.8 (m), 115.7 (s), 115.5 (s), 77.0 (s), 36.0 (s), 33.4 (s), 27.8 (s), 27.2 (s) ppm; ^{19}F NMR (377 MHz, $CDCl_3$) δ -106.0 ppm; HRMS (EI) (*m/z*) $[M]^+$: exact mass calc. for $C_{18}H_{17}FO_2$: 284.1213, found: 284.1207.

Cyclododecyl 4-fluorobenzoate (8h)

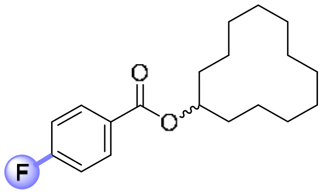

Prepared from cyclododecanone in two steps. Step 1: According to **General Procedure 2**. Step 2: According to **General Procedure 1**.

Yield: 2.76 g, 90%; colorless oil; IR (neat) ν (cm^{-1}): 2929, 2862, 1714, 1602, 1505, 1468, 1412, 1271, 1151, 1110, 1043, 1013, 984, 931, 902, 853, 767, 719, 685; ^{1}H NMR (400 MHz, $CDCl_3$) δ 8.11 – 7.94 (m, 2H), 7.19 – 6.96 (m, 2H), 5.24 (tt, *J* = 7.2, 4.7 Hz, 1H), 1.89 – 1.75 (m, 2H), 1.73 – 1.57 (m, 2H), 1.44 (dd, *J* = 12.5, 6.5 Hz, 8H), 1.36 (t, *J* = 8.4 Hz, 10H) ppm; ^{13}C NMR (101 MHz, $CDCl_3$) δ 167.1 – 166.6 (m), 165.3 (s), 164.6 – 164.0 (m), 132.0 (d, *J* = 9.3 Hz), 127.2 (d, *J* = 3.0 Hz), 115.3 (d, *J* = 21.9 Hz), 73.1 (s), 29.1 (s), 24.2 (s), 23.9 (s), 23.2 (d, *J* = 19.1 Hz), 20.8 (s) ppm; ^{19}F NMR (377 MHz, $CDCl_3$) δ -106.8 ppm; HRMS (EI) (*m/z*) $[M]^+$: exact mass calc. for $C_{19}H_{27}FO_2$: 306.1995, found: 306.1988.

Amyl 4-fluorobenzoate (8j)

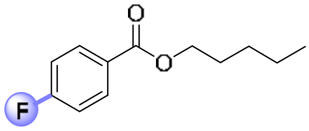

According to **General Procedure 1**. Yield: 2.06 g, 98%; colorless oil; IR (neat) ν (cm^{-1}): 2959, 2933, 2863, 1722, 1603, 1510, 1469, 1413, 1271, 1238, 1156, 1111, 1014, 969, 854, 768, 686; ^{1}H NMR (400 MHz, $CDCl_3$) δ 8.30 – 8.15 (m, 2H), 7.37 – 7.17 (m, 2H), 4.47 (t, *J* = 6.7 Hz, 2H), 2.02 – 1.84 (m, 2H), 1.67 – 1.46 (m, 4H), 1.10 (t, *J* = 7.0 Hz, 3H) ppm; ^{13}C NMR (101 MHz, $CDCl_3$) δ 165.6 (t, *J* = 126.8 Hz), 132.0 (d, *J* = 9.2 Hz), 126.8 (d, *J* = 3.0 Hz), 115.4 (d, *J* = 22.0 Hz), 65.2 (s), 28.3 (d, *J* = 23.7 Hz), 22.3 (s), 13.9 (s) ppm; ^{19}F NMR (377 MHz, $CDCl_3$) δ -106.6 (tt, *J* = 8.4, 5.5 Hz) ppm; HRMS (EI) (*m/z*) $[M]^+$: exact mass calc. for $C_{12}H_{15}FO_2$: 210.1056, found: 210.1055.

(3*R*,5*R*,8*R*,9*S*,10*S*,13*R*,14*S*,17*R*)-17-((*R*)-5-(Benzyloxy)-5-oxopentan-2-yl)-10,13-dimethylhexadecahydro-1*H*-cyclopenta[a]phenanthren-3-yl 4-fluorobenzoate (8k)

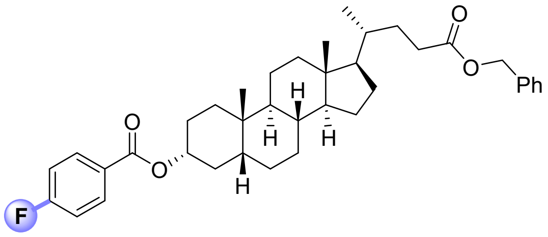

Prepared according to literature procedures.[16]

Step 1: To a solution of lithocholic acid (2.00 g, 5.31 mmol, 1.0 eq.) in 14 mL DMF, potassium carbonate (810.0 mg, 5.86 mmol, 1.1 eq.) was added. After 30 min of stirring, benzyl bromide (950.0 µL, 7.99 mmol, 1.5 eq.) was added to the mixture. The resulting solution was stirred for 3 hours at rt. Ethyl acetate and water were added to the reaction mixture. The organic layer was washed with water, dried over $MgSO_4$, and concentrated *in vacuo* to yield the crude product that was used for the next step without further purification.

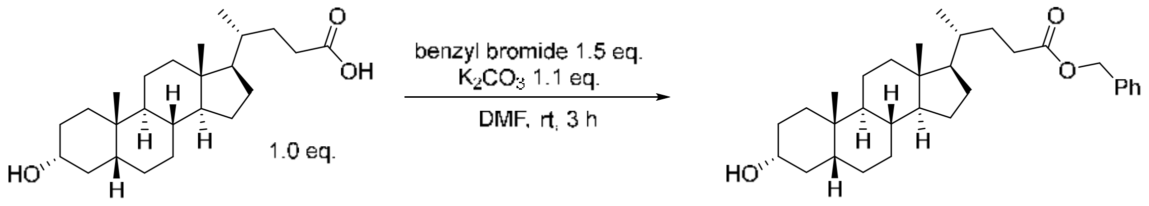

Step 2: To a solution of crude product of the first step reaction in 25 mL pyridine, 4-dimethylaminopyridine (630.0 mg, 5.31 mmol, 1.0 eq.) and 4-fluorobenzoyl chloride (1.25 mL, 10.62 mmol, 2.0 eq.) were added at 0 °C. The resulting mixture was stirred at rt for 24 hours. Aqueous hydrochloric acid was added to the mixture and was extracted with ethyl acetate. The organic layer was washed with aqueous sodium bicarbonate and water, dried over $MgSO_4$, and concentrated *in vacuo*. Purification of the residue by column chromatography on silica gel with 5% EtOAc in *n*-pentane provided the desired product.

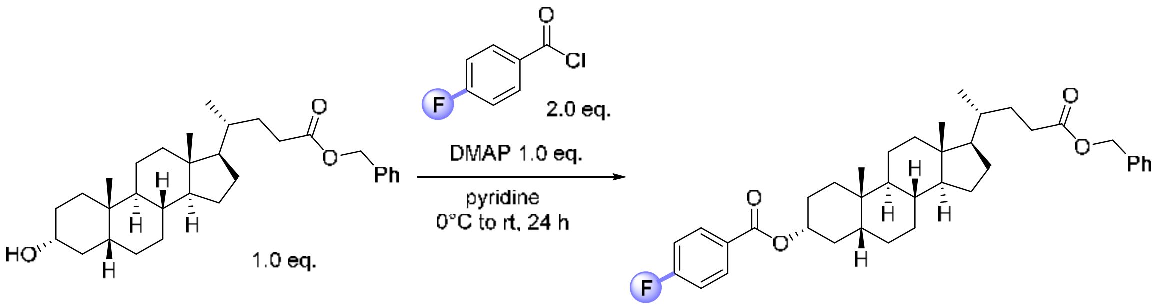

Yield: 2.75 g, 88%; white solid; IR (neat) ν (cm^{-1}): 2937, 2866, 1718, 1602, 1505, 1453, 1412, 1379, 1274, 1155, 1114, 1017, 984, 857, 767, 697; ^1H NMR (400 MHz, $CDCl_3$) δ 8.11 – 8.01 (m, 2H), 7.39 – 7.29 (m, 5H), 7.13 – 7.03 (m, 2H), 5.19 – 5.05 (m, 2H), 5.05 – 4.90 (m, 1H), 2.34 (dddd, *J* = 22.0, 15.5, 9.4, 5.9 Hz, 2H), 2.00 (dd, *J* = 21.9, 10.0 Hz, 2H), 1.91 – 1.76 (m, 5H), 1.67 (d, *J* = 12.4 Hz, 1H), 1.63 – 1.47 (m, 3H), 1.47 – 1.32 (m, 6H), 1.32 – 1.15 (m, 4H), 1.08 (tt, *J* = 13.2, 7.4 Hz, 5H), 0.96 (s, 3H), 0.92 (d, *J* = 6.3 Hz, 3H), 0.64 (s, 3H) ppm; ^{13}C NMR (101 MHz, $CDCl_3$) δ 174.0 (s), 166.8 (s), 165.1 (s), 164.3 (s), 136.1 (s), 133.3 (d, *J* = 9.7 Hz), 132.0 (d, *J* = 9.2 Hz), 128.4 (d, *J* = 30.7 Hz), 128.1 (s), 127.2 (d, *J* = 2.9 Hz), 116.2 (d, *J* = 22.3 Hz), 115.3 (d, *J* = 21.9 Hz), 75.1 (s), 66.0 (s), 56.5 (s), 56.0 (s), 42.7 (s), 41.9 (s), 40.5 (s), 40.1 (s), 35.8 (s), 35.3 (s), 35.1 (s), 34.6 (s), 32.3 (s), 31.2 (s), 31.0 (s), 28.2 (s), 27.0 (s), 26.8 (s), 26.3 (s), 24.2 (s), 23.3 (s), 20.9 (s), 18.3 (s), 12.0 (s) ppm; ^{19}F NMR (377 MHz, $CDCl_3$) δ -106.6 ppm; HRMS (ESI) (*m/z*) $[M+Na]^+$: exact mass calc. for $C_{38}H_{49}FO_4$: 611.3513, found: 611.3505.

N-((3s,5s,7s)-Adamantan-1-yl)-4-fluorobenzamide (10d)

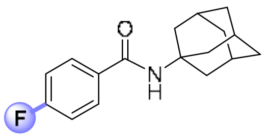

According to **General Procedure 3**. Yield: 2.60 g, 96%; white solid; ^{1}H NMR (400 MHz, $CDCl_3$) δ 7.76 – 7.59 (m, 2H), 7.12 – 6.94 (m, 2H), 5.81 (s, 1H), 2.09 (s, 9H), 1.69 (s, 6H) ppm; ^{13}C NMR (101 MHz, $CDCl_3$) δ 165.6 (s), 165.5 (s), 163.1 (s), 132.2 (d, *J* = 3.1 Hz), 129.0 (d, *J* = 8.8 Hz), 115.3 (d, *J* = 21.8 Hz), 52.3 (s), 41.6 (s), 36.3 (s), 29.4 (s) ppm; ^{19}F NMR (377 MHz, $CDCl_3$) δ -109.7 ppm; HRMS (EI) (*m/z*) $[M]^+$: exact mass calc. for $C_{17}H_{20}FNO$: 273.1529, found: 273.1516.
Data are consistent with the literature.[17]

4-Fluoro-N-pentylbenzamide (10a)

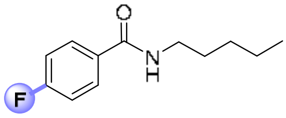

According to **General Procedure 3**. Yield: 1.90 g, 92%; colorless oil; ^{1}H NMR (400 MHz, $CDCl_3$) δ 7.83 – 7.70 (m, 2H), 7.07 – 6.95 (m, 2H), 6.81 (s, 1H), 3.43 – 3.23 (m, 2H), 1.63 – 1.47 (m, 2H), 1.37 – 1.19 (m, 4H), 0.93 – 0.75 (m, 3H) ppm; ^{13}C NMR (101 MHz, $CDCl_3$) δ 166.6 (s), 165.7 (s), 163.2 (s), 131.0 (d, *J* = 3.1 Hz), 129.2 (d, *J* = 8.8 Hz), 115.3 (d, *J* = 21.8 Hz), 40.2 (s), 29.3 (s), 29.1 (s), 22.3 (s), 13.9 (s) ppm; ^{19}F NMR (377 MHz, $CDCl_3$) δ -109.3 ppm; HRMS (EI) (*m/z*) $[M]^+$: exact mass calc. for $C_{12}H_{16}FNO$: 209.1216, found: 209.1208.
Data are consistent with the literature.[18]

Azepan-1-yl(4-fluorophenyl)methanone (10b)

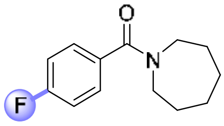

According to **General Procedure 3**. Yield: 1.97 g, 89%; white solid; ^{1}H NMR (400 MHz, $CDCl_3$) δ 7.31 (dt, *J* = 4.7, 4.1 Hz, 2H), 7.01 (t, *J* = 8.7 Hz, 2H), 3.73 – 3.40 (m, 2H), 3.30 (t, *J* = 5.4 Hz, 2H), 1.85 – 1.66 (m, 2H), 1.54 (d, *J* = 8.7 Hz, 6H) ppm; ^{13}C NMR (101 MHz, $CDCl_3$) δ 170.5 (s), 164.1 (s), 161.7 (s), 133.3 (d, *J* = 3.5 Hz), 128.6 (d, *J* = 8.3 Hz), 115.3 (d, *J* = 21.7 Hz), 49.7 (s), 46.4 (s), 29.4 (s), 27.7 (s), 27.2 (s), 26.3 (s) ppm; ^{19}F NMR (377 MHz, $CDCl_3$) δ -112.0 ppm; HRMS (EI) (*m/z*) $[M]^+$: exact mass calc. for $C_{13}H_{16}FNO$: 221.1216, found: 221.1199.
Data are consistent with the literature.[19]

_N_-Cycloheptyl-4-fluorobenzamide (10c)

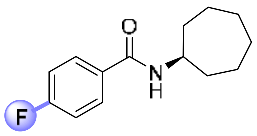

According to **General Procedure 3**. Yield: 2.30 g, 99%; white solid; IR (neat) ν (cm^{-1}): 3298, 2926, 2855, 1628, 1543, 1502, 1446, 1326, 1289, 1226, 1155, 1051, 1013, 887, 846, 801, 767, 711, 670; ^{1}H NMR (400 MHz, $CDCl_3$) δ 8.01 – 7.83 (m, 2H), 7.33 – 7.17 (m, 2H), 6.25 (s, 1H), 4.39 – 4.25 (m, 1H), 2.30 – 2.11 (m, 2H), 1.85 (td, *J* = 8.0, 2.2 Hz, 4H), 1.78 – 1.66 (m, 6H) ppm; ^{13}C NMR (101 MHz, $CDCl_3$) δ 165.8 (s), 165.3 (s), 163.3 (s), 131.3 (d, *J* = 3.2 Hz), 129.1 (d, *J* = 8.8 Hz), 115.4 (d, *J* = 21.9 Hz), 51.0 (s), 35.1 (s), 28.0 (s), 24.1 (s) ppm; ^{19}F NMR (377 MHz, $CDCl_3$) δ -109.3 ppm; HRMS (EI) (*m/z*) [M]$^{+}$: exact mass calc. for $C_{14}H_{18}FNO$: 235.1372, found: 235.1363.

_N_-(Cyclohexylmethyl)-4-fluorobenzamide (10e)

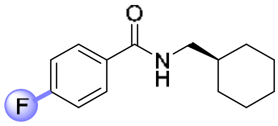

According to **General Procedure 3**. Yield: 2.16 g, 92%; white solid; ^{1}H NMR (400 MHz, $CDCl_3$) δ 8.12 – 7.84 (m, 2H), 7.40 – 7.19 (m, 2H), 6.42 (s, 1H), 3.45 (t, *J* = 6.4 Hz, 2H), 1.92 (dd, *J* = 16.9, 8.6 Hz, 4H), 1.87 – 1.81 (m, 1H), 1.81 – 1.68 (m, 1H), 1.54 – 1.25 (m, 3H), 1.25 – 1.08 (m, 2H) ppm; ^{13}C NMR (101 MHz, $CDCl_3$) δ 166.5 (s), 165.8 (s), 163.3 (s), 131.0 (d, *J* = 3.1 Hz), 129.1 (d, *J* = 8.9 Hz), 115.5 (d, *J* = 21.8 Hz), 46.3 (s), 38.0 (s), 30.9 (s), 26.3 (s), 25.8 (s) ppm; ^{19}F NMR (377 MHz, $CDCl_3$) δ -109.1 ppm; HRMS (EI) (*m/z*) [M]$^{+}$: exact mass calc. for $C_{14}H_{18}FNO$: 235.1372, found: 235.1368.
Data are consistent with the literature.[20]

4-Fluoro-_N_-(1-hydroxy-2-(hydroxymethyl)-4-(4-octylphenyl)butan-2-yl)benzamide (10f)

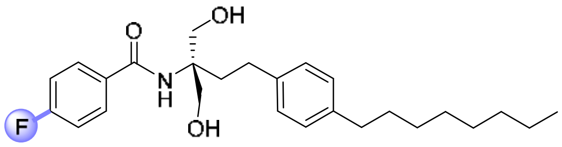

Prepared from Fingolimod Hydrochloride according to **General Procedure 3**. Yield: 1.26 g, 90%; white solid; IR (neat) v (cm^{-1}): 2926, 2855, 1714, 1643, 1602, 1535, 1498, 1461, 1364, 1233, 1159, 1095, 1051, 849, 812, 767; ^{1}H NMR (400 MHz, $CDCl_3$) δ 7.63 – 7.54 (m, 2H), 7.14 – 7.00 (m, 6H), 6.62 (s, 1H), 3.97 (d, *J* = 11.5 Hz, 2H), 3.84 (dd, *J* = 104.8, 11.5 Hz, 6H), 3.71 (d, *J* = 11.5 Hz, 2H), 2.74 – 2.60 (m, 2H), 2.60 – 2.47 (m, 2H), 2.15 – 2.00 (m, 2H), 1.55 (dd, *J* = 14.8, 7.3 Hz, 2H), 1.29 (s, 5H), 1.26 (s, 5H), 0.88 (t, *J* = 6.9 Hz, 3H) ppm; ^{13}C NMR (101 MHz, $CDCl_3$) δ 167.4 (s), 166.1 (s), 163.6 (s), 139.7 (d, *J* = 244.3 Hz), 130.4 (d, *J* = 3.1 Hz), 129.3 (d, *J* = 8.9 Hz), 128.4 (d, *J* = 53.7 Hz), 115.6 (d, *J* = 21.9 Hz), 65.8 (s), 61.6 (s), 35.5

(s), 34.3 (s), 31.8 (s), 31.5 (s), 29.4 (s), 29.3 (s), 29.2 (s), 22.6 (s), 14.1 (s) ppm; ^{19}F NMR (377 MHz, $CDCl_3$) δ -108.1 ppm; HRMS (ESI) (*m/z*) $[M+H]^+$: exact mass calc. for $C_{26}H_{36}FNO_3$: 430.2752, found: 430.2752.

(4-Fluorophenyl)((4b*S*,9*S*)-3-methoxy-6,7,8,8a,9,10-hexahydro-5*H*-9,4b-(epiminoethano)phenanthren-11-yl)methanone (10h)

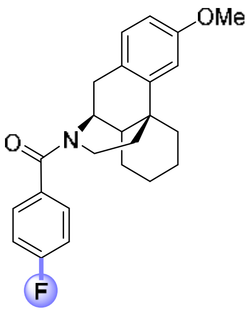

The substrate was synthesized in two steps. Step 1: Following the procedure of Olfoson et al.[21] A mixture of dextromethorphan (3.13 g, 11.50 mmol, 1.0 eq.), alpha-chloroethyl chloroformate (11 mL, 100.00 mmol, 9.0 eq.), 1,2-dichloroethane (48 mL), and $NaHCO_3$ (1.44 g, 17.00 mmol, 1.5 eq.) was allowed to reflux for 48 h. After filtration, the filtrate was concentrated under vacuum and 300 mL MeOH was added. The resulting mixture was heated to reflux for 3 h. The solvent was evaporated under vacuum and the residue was dissolved 35 mL DCM. The mixture was washed with NaOH (1.80 N, 6 mL) and water (to pH 7), dried over $MgSO_4$,and concentrated to afford an oil **25**. The product was used for the next step reaction.

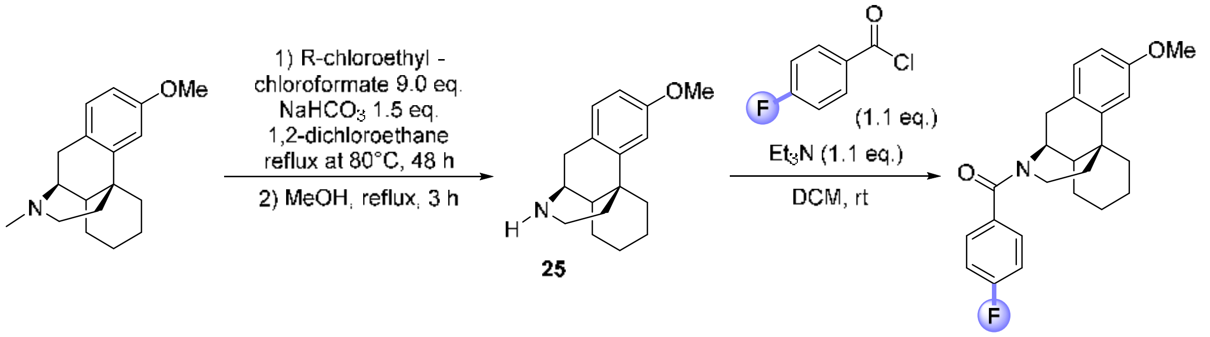

Step 2: According to **General Procedure 3**. Yield: 3.75 g, 86%; white solid; IR (neat) ν (cm^{-1}): 2929, 2855, 1714, 1625, 1498, 1423, 1371, 1326, 1297, 1271, 1241, 1155, 1121, 1039, 913, 849, 808, 760, 700; ^{1}H NMR (400 MHz, $CDCl_3$) δ 7.45 – 7.30 (m, 4H), 7.03 (tt, *J* = 14.5, 7.3 Hz, 6H), 6.87 – 6.77 (m, 2H), 6.71 (dd, *J* = 8.4, 2.4 Hz, 2H), 4.94 (s, 1H), 4.43 (dd, *J* = 13.7, 3.6 Hz, 1H), 3.84 (s, 1H), 3.75 (d, *J* = 3.4 Hz, 6H), 3.38 (dd, *J* = 13.6, 3.6 Hz, 1H), 3.21 (dd, *J* = 18.3, 6.2 Hz, 1H), 3.13 – 2.84 (m, 2H), 2.80 – 2.54 (m, 3H), 2.45 – 2.24 (m, 2H), 1.76 (d, *J* = 12.5 Hz, 1H), 1.72 – 1.41 (m, 9H), 1.39 – 1.17 (m, 8H), 1.04 (ddd, *J* = 47.0, 24.5, 12.3 Hz, 2H) ppm; ^{13}C NMR (101 MHz, $CDCl_3$) δ 169.3 (s), 169.1 (s), 164.3 (s), 161.9 (s), 158.6 (s), 158.5 (s), 140.2 (s), 132.9 (d, *J* = 2.9 Hz), 132.7 (d, *J* = 3.2 Hz), 129.1 (s), 129.0 (s), 128.8 (d, *J* = 9.6 Hz), 128.7 (s), 128.2 (s), 127.5 (s), 115.7 (s), 115.5 (s), 115.3 (s), 111.4 (s), 111.3 (s), 111.2 (s), 55.1 (s), 54.1 (s), 47.8 (s), 44.8 (s), 43.9 (s), 42.5 (s), 42.2 (s), 41.2 (s), 38.0 (s), 37.9 (s), 36.4 (s), 36.3 (s), 31.8 (s), 31.1 (s), 26.4

(s), 26.3 (s), 26.2 (s), 22.0 (s), 21.9 (s) ppm; ^{19}F NMR (377 MHz, $CDCl_3$) δ -111.3 ppm; HRMS (EI) (*m/z*) $[M]^+$: exact mass calc. for $C_{24}H_{26}FNO_2$: 379.1948, found: 379.1934.

(4b*S*,9*S*)-11-Methyl-6,7,8,8a,9,10-hexahydro-5*H*-9,4b-(epiminoethano)phenanthren-3-yl 4-fluorobenzoate (8l)

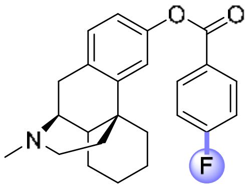

The substrate was synthesized in two steps.

Step 1: Following the procedure of Senderoff et al.[22a] dextromethorphan hydrobromide hydrate (2.70 g, 7.29 mmol, 1.0 eq.) was added to a round bottom flask equipped with a magnetic stir bar. Hydrobromic acid (16 mL, 48 wt.% in H_2O) was added, and the reaction was refluxed for 24 hours. After cooling the reaction mixture to rt, it was poured onto ice and the resultant solution was basified with saturated K_2CO_3 to pH = 10. The aqueous layer was extracted with DCM three times. The combined organic layer was washed with brine, dried over $MgSO_4$, filtered, and concentrated *in vacuo* to afford the crude product. The product was used for the next step reaction without further purification.

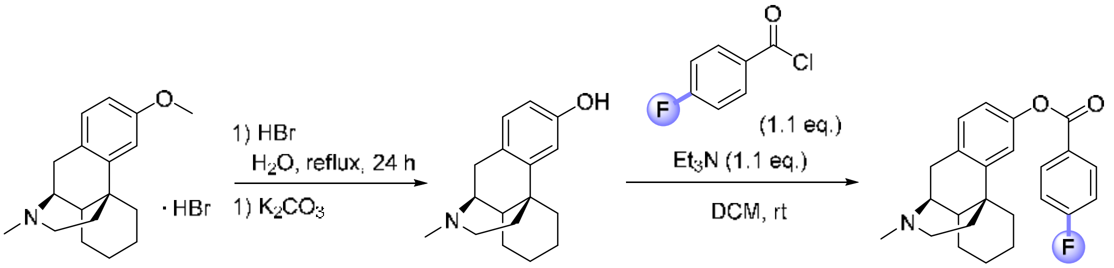

Step 2: According to **General procedure 1**.

Yield: 2.41 g, 87%; white solid; IR (neat) ν (cm^{-1}): 3406, 2929, 2858, 2378, 1736, 1602, 1494, 1435, 1360, 1256, 1211, 1151, 1058, 894, 853, 782, 755, 730, 685; ^{1}H NMR (400 MHz, $CDCl_3$) δ 8.22 – 8.11 (m, 2H), 7.17 – 7.07 (m, 3H), 7.04 (s, 1H), 6.94 (d, *J* = 8.3 Hz, 1H), 3.01 (d, *J* = 18.4 Hz, 1H), 2.82 (d, *J* = 23.3 Hz, 1H), 2.72 – 2.53 (m, 1H), 2.48 – 2.21 (m, 5H), 2.06 (t, *J* = 12.2 Hz, 1H), 1.82 (d, *J* = 12.6 Hz, 1H), 1.72 (td, *J* = 12.4, 3.9 Hz, 1H), 1.60 (t, *J* = 14.3 Hz, 1H), 1.49 (d, *J* = 6.4 Hz, 1H), 1.43 – 1.26 (m, 5H), 1.10 (dd, *J* = 23.7, 11.7 Hz, 1H) ppm; ^{13}C NMR (101 MHz, $CDCl_3$) δ 167.3 (s), 164.7 (s), 164.2 (s), 149.4 (s), 142.0 (s), 135.3 (s), 132.7 (d, *J* = 9.4 Hz), 128.6 (s), 126.0 (d, *J* = 3.0 Hz), 118.6 (s), 118.2 (s), 115.8 (s), 115.5 (s), 57.8 (s), 47.1 (s), 45.2 (s), 42.8 (s), 41.9 (s), 37.3 (s), 36.5 (s), 26.6 (s), 26.5 (s), 23.7 (s), 22.1 (s) ppm; ^{19}F NMR (377 MHz, $CDCl_3$) δ -105.1 ppm; HRMS (ESI) (*m/z*) $[M+H]^+$: exact mass calc. for $C_{24}H_{26}FNO_2$: 380.2020, found: 380.2064.

Methyl 2-(4-isobutylphenyl)propanoate (1y)

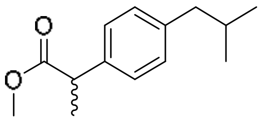

The methyl 2-(4-isobutylphenyl)propanoate was prepared according to the literature procedures.[22b] The mixture of racemic Ibuprofen (10.00 mmol, 1.0 eq.), methanol (30.00 mmol, 3.0 eq.), and DMAP (1.00 mmol, 0.1 eq.) in 10 mL DCM at 0 °C was stirred for 5 min. DCC (11.00 mmol, 1.1 eq.) was added to the mixture and stirred at 0 °C for 5min. The reaction was stirred at rt for 3 h. The resulting precipitates were filtered and the filtrate was treated with diluted HCl. The product was extracted with DCM. The organic layers were combined, dried over $MgSO_4$, and concentrated *in vacuo*. The residue was purified by column chromatography using 5% EtOAc in pentane to give the desired product.

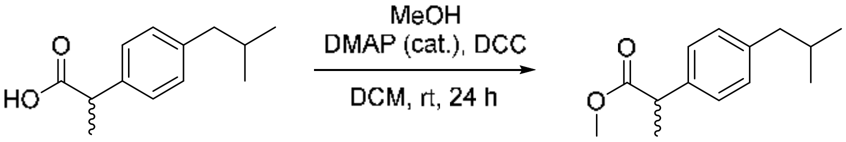

Yield: 2.14 g, 97%; colorless oil; ^{1}H NMR (400 MHz, $CDCl_3$) δ 7.27 (d, *J* = 8.1 Hz, 2H), 7.16 (d, *J* = 8.1 Hz, 2H), 3.77 (q, *J* = 7.2 Hz, 1H), 3.72 (s, 3H), 2.52 (d, *J* = 7.2 Hz, 2H), 1.91 (td, *J* = 13.6, 6.8 Hz, 1H), 1.56 (d, *J* = 7.2 Hz, 3H), 0.97 (d, *J* = 6.6 Hz, 6H) ppm; ^{13}C NMR (101 MHz, $CDCl_3$) δ 175.2, 140.5, 137.8, 129.3, 127.1, 55.7, 51.9, 45.1, 45.0, 34.9, 30.2, 25.5, 24.7, 22.4, 18.6 ppm; HRMS (ESI) (*m/z*) $[M]^+$: exact mass calc. for $C_{14}H_{20}O_2$: 220.1463, found: 220.1460.

Data are consistent with the literature.[22b]

1-(2-Ethylhexyl)-1-methylpyrrolidin-1-ium hexafluorophosphate (1aa)

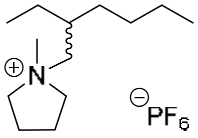

Prepared according to the **General Procedure 4**. Yield: 3.40 g, 99%.

IR (neat) ν (cm^{-1}): 2963, 2940, 2874, 1711, 1465, 1364, 1223, 1006, 932, 828; ^{1}H NMR (400 MHz, DMSO) δ 3.57 – 3.46 (m, 2H), 3.43 – 3.31 (m, 2H), 3.27 – 3.15 (m, 2H), 2.96 (s, 3H), 2.15 – 1.99 (m, 4H), 1.92 – 1.77 (m, 1H), 1.47 – 1.16 (m, 8H), 0.88 (td, *J* = 7.1, 4.9 Hz, 6H) ppm; ^{13}C NMR (101 MHz, DMSO) δ 67.5 (s), 63.9 (s), 47.6 (s), 34.2 (s), 32.5 (s), 28.0 (s), 25.8 (s), 22.8 (s), 21.1 (d, *J* = 4.1 Hz), 14.3 (s), 10.4 (s) ppm; ^{19}F NMR (377 MHz, DMSO) δ -69.8 (d, *J* = 711.4 Hz) ppm; ^{31}P NMR (162 MHz, DMSO) δ -143.0 (hept, *J* = 711.4 Hz) ppm; HRMS (EI) (*m/z*) $[M]^+$: exact mass calc. for $C_{13}H_{28}N$: 198.2216, found: 198.2218.

1-Heptyl-3-methyl-1*H*-imidazol-3-ium tetrafluoroborate (1ac)

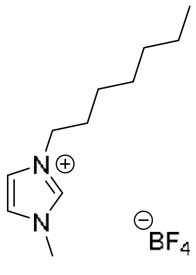

Prepared according to the **General Procedure 4**. Yield: 2.65 g, 98%; ^{1}H NMR (400 MHz, $CDCl_3$) δ 8.70 (s, 1H), 7.34 (dt, *J* = 17.2, 1.8 Hz, 2H), 4.10 (e, 2H), 3.86 (s, 3H), 1.80 (ь, *J* = 14.5, 7.3 Hz, 2H), 1.32 – 1.01 (m, 8H), 0.77 (t, *J* = 6.9 Hz, 3H) ppm; ^{13}C NMR (101 MHz, $CDCl_3$) δ 136.0 (s), 123.8 (s), 122.3 (s), 49.9 (s), 36.1 (s), 31.4 (s), 30.0 (s), 28.5 (s), 26.0 (s), 22.4 (s), 13.9 (s) ppm; ^{19}F NMR (377 MHz, $CDCl_3$) δ -151.4 – -151.6 (m, *J* = 10.9, 10.0 Hz); HRMS (EI) (*m/z*) $[M]^+$: exact mass calc. for $C_{11}H_{21}N_2$: 181.1699, found: 181.1703.

Data are consistent with the literature.[23a]

3-Methyl-1-(3-phenylpropyl)-1*H*-imidazol-3-ium hexafluorophosphate (1ad)

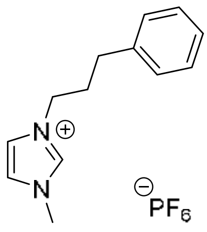

Prepared according to the **General Procedure 4**. Yield: 3.43 g, 99%; IR (neat) ν (cm^{-1}): 3168, 3124, 3030, 2937, 2866, 1707, 1603, 1573, 1498, 1454, 1364, 1226, 1170, 1111, 1029, 820, 746, 701; ^{1}H NMR (400 MHz, DMSO) δ 8.94 (s, 1H), 7.62 (t, *J* = 1.8 Hz, 1H), 7.53 (t, *J* = 1.7 Hz, 1H), 7.22 – 7.13 (m, 2H), 7.08 (ddd, *J* = 6.4, 5.3, 1.7 Hz, 3H), 4.06 (t, *J* = 7.2 Hz, 2H), 3.71 (s, 3H), 2.53 – 2.44 (m, 2H), 2.08 – 1.94 (m, 2H) ppm; ^{13}C NMR (101 MHz, DMSO) δ 140.9 (s), 137.0 (s), 128.8 (d, *J* = 15.7 Hz), 126.5 (s), 124.0 (s), 122.6 (s), 49.0 (s), 36.1 (s), 32.1 (s), 31.3 (s) ppm; ^{19}F NMR (377 MHz, DMSO) δ -69.7 (d, *J* = 711.4 Hz) ppm; ^{31}P NMR (162 MHz, DMSO) δ -142.9 (hept, *J* = 711.5 Hz) ppm; HRMS (EI) (*m/z*) $[M]^+$: exact mass calc. for $C_{13}H_{17}N_2$: 201.1386, found: 201.1391.

1-Butyl-2,3-dimethyl-1*H*-imidazol-3-ium bis((trifluoromethyl)sulfonyl)amide (1ae)

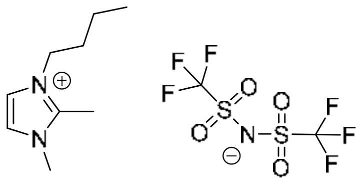

Prepared according to the **General Procedure 4**. Yield: 4.30 g, 99%; ^{1}H NMR (400 MHz, $CDCl_3$) δ 7.26 (s, 2H), 4.09 (t, J = 7.5 Hz, 2H), 3.82 (s, 3H), 2.63 (s, 3H), 1.91 – 1.65 (m, 2H), 1.41 (dq, J = 14.8, 7.3 Hz, 2H), 1.00 (t, J = 7.3 Hz, 3H) ppm; ^{13}C NMR (101 MHz, $CDCl_3$) δ 143.6 (s), 124.5 (s), 122.4 (s), 121.3 (s), 120.8 (s), 118.1 (s), 114.9 (s), 48.4 (s), 35.0 (s), 31.3 (s), 19.3 (s), 13.1 (s), 9.3 (s) ppm; ^{19}F NMR (377 MHz, $CDCl_3$) δ -79.8 (s) ppm; HRMS (EI) (*m/z*) [M]$^+$: exact mass calc. for $C_9H_{17}N_2$: 153.1386, found: 153.1390. Data are consistent with the literature.[23a]

***N*-(2-((S)-2-cyanopyrrolidin-1-yl)-2-oxoethyl)-4-fluoro-*N*-((1r,3R,5R,7S)-3-hydroxyadamantan-1-yl)benzamide (10g)**

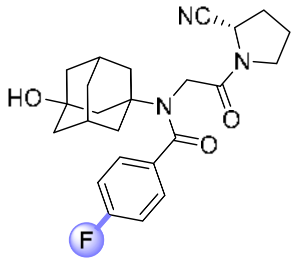

Prepared from Vildagliptin according to **General Procedure 3**. Purification was conducted via column chromatography (pure EtOAc) that afforded the desired product as white solid. Yield: 1.20 g, 94%.

IR (neat) ν (cm^{-1}): 3399, 2915, 2855, 2244, 1662, 1633, 1510, 1443, 1394, 1323, 1260, 1223, 1159, 1096, 1044, 999, 910, 846, 727; ^{1}H NMR (400 MHz, $CDCl_3$) δ 7.35 – 7.27 (m, 9H), 6.98 (t, J = 8.7 Hz, 9H), 4.73 – 4.54 (m, 4H), 3.90 (q, J = 18.3 Hz, 8H), 3.02 (dd, J = 17.7, 9.8 Hz, 8H), 2.67 (s, 5H), 2.34 – 1.93 (m, 56H), 1.76 – 1.40 (m, 28H) ppm; ^{13}C NMR (101 MHz, $CDCl_3$) δ 172.2 (s), 168.8 (s), 164.1 (s), 161.6 (s), 135.3 (d, J = 3.5 Hz), 128.0 (d, J = 8.2 Hz), 117.8 (s), 115.6 (d, J = 21.6 Hz), 77.4 (s), 69.5 (s), 61.3 (s), 49.0 (s), 47.3 (s), 46.8 (s), 45.4 (s), 44.0 (d, J = 17.2 Hz), 37.9 (s), 34.8 (s), 31.0 (d, J = 2.9 Hz), 29.5 (s), 25.1 (s) ppm; ^{19}F NMR (377 MHz, $CDCl_3$) δ -111.5 – -111.6 (m) ppm; HRMS (EI) (*m/z*) [M+Na]$^+$: exact mass calc. for $C_{24}H_{28}FN_3O_3$: 448.2012, found: 448.2010.

4-Phenylbutyl acetate (19)

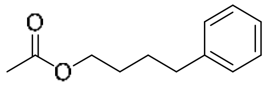

Prepared according to the **General Procedure 1**. Purification was conducted via column chromatography (5% EtOAc in PE) that afforded the desired product as colorless oil. Yield: 1.93 g, 97%.
^{1}H NMR (400 MHz, $CDCl_3$) δ 7.40 – 7.21 (m, 5H), 4.16 (dd, *J* = 8.6, 4.0 Hz, 2H), 2.72 (t, *J* = 7.2 Hz, 2H), 2.11 (s, 3H), 1.83 – 1.63 (m, 4H) ppm; ^{13}C NMR (101 MHz, $CDCl_3$) δ 171.2 (s), 142.1 (s), 128.4 (d, *J* = 4.3 Hz), 125.9 (s), 64.4 (s), 35.5 (s), 28.0 (d, *J* = 47.6 Hz), 21.0 (s) ppm; HRMS (EI) (*m/z*) [M]$^+$: exact mass calc. for $C_{12}H_{16}O_2$: 192.1150, found: 192.1145.

Data are consistent with the literature.[23b]

2.2 Synthesis of Fluorinated products via PS Fluorination

General Procedure A

Selectfluor® (**SF**) (0.564 mmol, 1.0 eq.), starting material (0.846 mmol, 1.5 eq.) and methyl 4-fluorobenzoate (1 mol% based on **SF**) are dissolved in 2.7 mL dry MeCN inside a 5 mL crimp vial equipped with stir bar. The vial was sealed and degassed via three cycles of freeze-pump-thaw and then filled with N_2. The reaction mixture was stirred under 400 nm (LED) irradiation for 24 hours at room temperature.

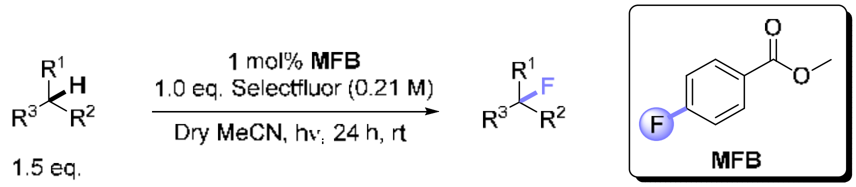

General Procedure B

SF (0.282 mmol, 1.0 eq.), starting material (0.423 mmol, 1.5 eq.) and methyl 4-fluorobenzoate (10 mol% based on **SF**) are dissolved in 2.7 mL dry MeCN inside a 5 mL crimp vial equipped with stir bar. The vial was sealed and degassed via three cycles of freeze-pump-thaw and then filled with N_2. The reaction mixture was stirred under 400 nm (LED) irradiation for 24 hours at room temperature.

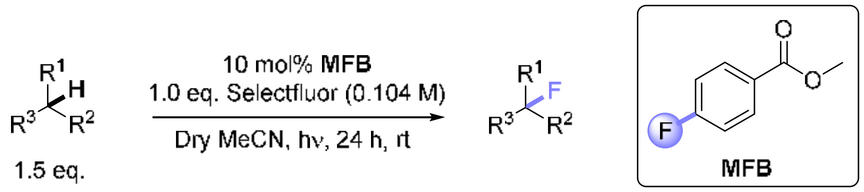

General Procedure C

SF (0.564 mmol, 1.0 eq.) and starting material (0.846 mmol, 1.5 eq.) are dissolved in 2.7 mL dry MeCN inside a 5 mL crimp vial equipped with stir bar. The vial was sealed and degassed via three cycles of freeze-pump-thaw and then filled with N_2. The reaction mixture was stirred under 400 nm (LED) irradiation for 24 hours at room temperature.

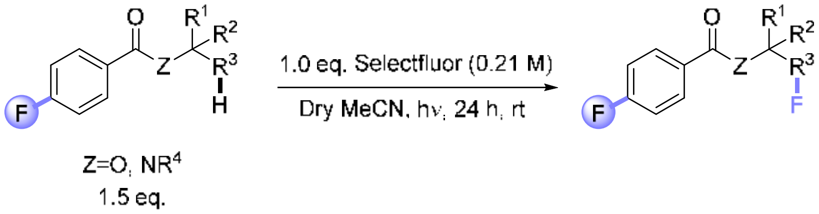

Isolation for General Procedure A, B and C

After the reaction, diethyl ether was added to the reaction mixture, and instant precipitation of unreacted **SF** and salt derived from **SF** was observed. The mixture was filtered into a flask and the residue was washed with more diethyl ether. Solvent was removed under reduced pressure. Purification of the residue was conducted by column chromatography using silica gel and the specified solvent to afford the corresponding product.

General Procedure D

To a solution of the ester in methanol (0.5 M), NaOH (3.0 eq.) was added, and the reaction mixture was refluxed for 30 min. The solvent was removed under vacuum and the residue was extracted with water and DCM. Organic layers were combined, dried over $MgSO_4$, filtered, and concentrated. Purification of the crude product was performed by column chromatography.

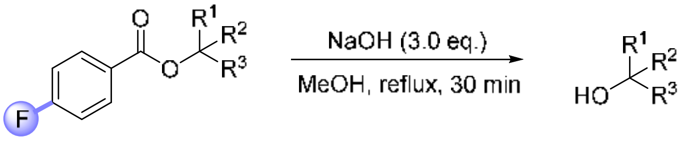

General Procedure E

Following procedure from literature[23c] an amide substrate (neat) was added to an oven-dried vial equipped with a stir bar, a positive pressure of N_2 was applied, and three evacuation/backfilling cycles under high vacuum were performed. Samarium(II) iodide (THF solution, 8.0 eq.) was added to the vial followed by the addition of Et_3N (72.0 eq.) and water (72.0 eq.) with vigorous stirring. Formation of a characteristic dark brown color of the SmI_2–Et_3N–H_2O complex was observed, and the reaction mixture was stirred for 18 h. Air was bubbled through the reaction mixture to oxidize the excess of Sm(II) and the reaction mixture was diluted with 30 mL DCM and NaOH (1.0 N, 10 mL). The aqueous layer was extracted with DCM (3 x 30 mL). The organic layers were combined, dried over $MgSO_4$, filtered, and concentrated under vacuum. The crude product was purified by column chromatography on silica gel.

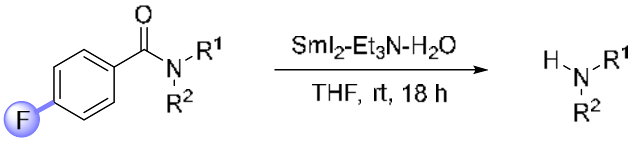

4-(3-fluorobutyl)benzamide (2e)

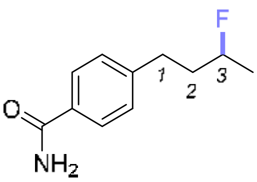

Prepared from 4-*n*-butylbenzamide according to the **General Procedure A** to give **2e** (66% NMR yield, (trifluoromethyl)benzene as IS) in 8 : 2.5 : 1 (3F : 2F : 1F) ratio (according to ^{19}F NMR). ^{9}F NMR (377 MHz, $CDCl_3$) δ -174.9 (2F), -178.4 (3F), -180.6 (1F) ppm; HRMS (EI) (*m/z*) $[M]^+$: exact mass calc. for $C_{11}H_{14}FNO$: 195.1059, found: 195.1058.

3-Fluoro-2,3-dihydro-1*H*-inden-1-one (2i)

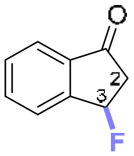

Prepared from 1-indanone according to the **General Procedure A** to give **2i** (41% NMR yield, (trifluoromethyl)benzene as IS) in 9 : 1 (3F : 2F) ratio (according to ^{19}F NMR). ^{19}F NMR (377 MHz, $CDCl_3$) δ -169.1 (3F), -194.4 (2F) ppm.
Data are consistent with the literature.[24]

13-Fluoro-*N,N*-dimethyltetradecan-1-amine (2s)

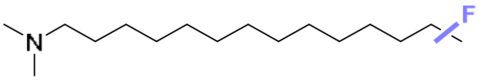

Prepared from *N,N*-dimethyltetradecylamine according to the general procedure **B** to give **2s** (33% NMR yield, (trifluoromethyl)benzene as IS). ^{19}F NMR (282 MHz, $CDCl_3$) δ -132.0, -156.4 ppm.

1-Fluorocyclohexane (2u)

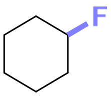

Prepared from cyclohexane according to the general procedure **A** to give **2t** (95% NMR yield, (trifluoromethyl)benzene as IS). ^{19}F NMR (282 MHz, CD_3CN) δ -171.0 ppm.
Data are consistent with the literature.[25]

1-Fluoro-1-methylcyclohexane (2v)

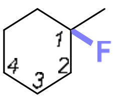

Prepared from cyclohexane according to the general procedure **A** to give **2u** (mixture of fluorinated isomers, difficult to assign fluorine positions) (96% NMR yield, (trifluoromethyl)benzene as IS). ^{19}F NMR (282 MHz, CD_3CN) δ -165.7, -172.6, -181.2, -182.7 ppm.

1-Fluorocyclooctane (2t)

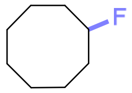

Prepared from cyclooctane according to the general procedure **A** to give **2m** (80 % NMR yield, (trifluoromethyl)benzene as IS). ^{19}F NMR (377 MHz, $CDCl_3$) δ –159.9 ppm.

Data are consistent with the literature.[25]

1-Fluorocyclododecane (2x)

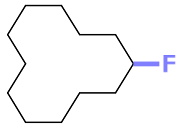

Prepared from cyclododecane according to the general procedure **A** to give **2w** (89% NMR yield, (trifluoromethyl)benzene as IS). ^{19}F NMR (282 MHz, $CDCl_3$) δ -177.0 ppm.

Data are consistent with the literature.[25]

4-(3-Fluorobutyl)benzonitrile (2d)

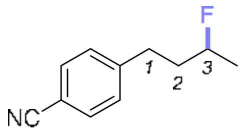

Prepared from 4-*n*-butylbenzonitrile according to **General Procedure B**. Purification was conducted via column chromatography (10% EtOAc in PE) that afforded the mixture of 3 isomers in 7 : 2 : 1 (3F : 2F : 1F) ratio (based on ^{19}F NMR) as yellow viscous liquid. Yield: 27.0 mg, 55%.

IR (neat) ν (cm^{-1}): 2978, 2937, 2228, 1740, 1610, 1505, 1453, 1386, 1349, 1282, 1200, 1133, 1110, 1062, 1021, 950, 930, 887, 841, 827, 775; ^{1}H NMR (400 MHz, $CDCl_3$) δ 7.55 – 7.49 (m, 2H), 7.25 (d, *J* = 8.2 Hz, 2H), 4.76 – 4.39 (m, 1H), 2.76 (m, 2H), 1.99 – 1.84 (m, 1H), 1.84 – 1.66 (m, 1H), 1.30 (dd, *J* = 23.8, 6.2 Hz, 3H) ppm; ^{13}C NMR (101 MHz, $CDCl_3$) δ 147.1 (s), 132.5 – 132.0 (m), 130.2 (s), 129.2 (s), 125.9 (d, *J* = 7.7 Hz), 119.0 (s), 109.9 (s), 95.7 (s), 93.9 (s), 90.4 (s), 88.8 (s), 41.3 (s), 41.1 (s), 39.3 (s), 39.1 (s), 38.1 (s), 37.9 (s), 31.5 (d, *J* = 4.6 Hz), 29.7 (s), 27.9 (s), 27.7 (s), 21.0 (s), 20.8 (s), 18.1 (d, *J* = 4.3 Hz), 13.7 (s), 9.3 (d, *J* = 5.7 Hz) ppm; ^{19}F NMR (377 MHz, $CDCl_3$) δ -175.3 ppm.

HRMS (EI) (*m/z*) $[M]^+$: exact mass calc. for $C_{11}H_{12}FN$: 177.0954, found: 177.0951.

(3-Bromo-1-fluoropropyl)benzene (2h)

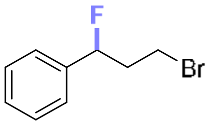

Prepared from (3-bromopropyl)benzene according to **General Procedure A**. Purification was conducted via column chromatography (1% DCM in PE) that afforded the product as yellow viscous liquid. Yield: 66.0 mg, 54%.

^{1}H NMR (400 MHz, $CDCl_3$) δ 7.56 – 7.32 (m, 5H), 5.69 (ddd, *J* = 47.8, 8.8, 3.9 Hz, 1H), 3.72 – 3.37 (m, 2H), 2.56 (m, 1H), 2.31 (m, 1H) ppm; ^{13}C NMR (101 MHz, $CDCl_3$) δ 139.0 (d, *J* = 19.6 Hz), 128.7 (s), 125.5 (d, *J* = 6.7 Hz), 93.0 (s), 91.3 (s), 40.2 (d, *J* = 24.5 Hz), 28.5 (d, *J* = 4.8 Hz) ppm; ^{19}F NMR (377 MHz, $CDCl_3$) δ -179.8 ppm. HRMS (EI) (*m/z*) $[M]^+$: exact mass calc. for $C_9H_{10}BrF$: 215.9950, found: 215.9939.

Data are consistent with the literature.[26]

2-Fluoro-2-phenylethyl acetate (2j)

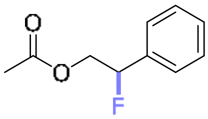

Prepared from phenethyl acetate according to **General Procedure A**. Purification was conducted via column chromatography (5% EtOAc in PE) that afforded the product as slightly yellow viscous liquid. Yield: 28.0 mg, 27%.

^{1}H NMR (400 MHz, $CDCl_3$) δ 7.49 – 7.30 (m, 5H), 5.80 – 5.50 (m, 1H), 4.49 – 4.29 (m, 2H), 2.12 (s, 3H) ppm; ^{13}C NMR (101 MHz, $CDCl_3$) δ 170.7 (s), 135.8 (d, *J* = 19.7 Hz), 129.0 (d, *J* = 1.6 Hz), 128.6 (s), 125.7 (d, *J* = 6.8 Hz), 91.7 (d, *J* = 175.8 Hz), 66.8 (d, *J* = 24.5 Hz), 20.8 (s) ppm; ^{19}F NMR (376 MHz, $CDCl_3$) δ -184.8 ppm.

HRMS (+APCI) (*m/z*) $[M+NH_4]^+$: exact mass calc. for $C_{10}H_{11}FO_2$: 200.1081, found: 200.1084.

Data are consistent with the literature.[27]

5-Fluorohexyl propionate (2k)

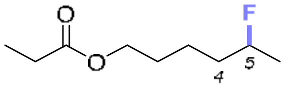

Prepared from hexyl propionate according to **General Procedure A.** Purification was conducted via column chromatography (5% EtOAc in PE) that afforded the mixture of two isomers in 3.5 : 1 (5F : 4F) ratio (based on ^{19}F NMR) as colorless viscous liquid. Yield: 67.0 mg, 68%.

IR (neat) ν (cm^{-1}): 2963, 2926, 2855, 1259, 1084, 1021, 864, 797, 700; ^{1}H NMR (300 MHz, $CDCl_3$) δ 4.81 – 4.27 (m, 3H), 4.08 (q, *J* = 6.3 Hz, 5H), 2.31 (q, *J* = 7.6 Hz, 5H), 1.64 (d, *J* = 8.0 Hz, 11H), 1.55 – 1.38 (m, 5H), 1.31 (dd, *J* = 23.9, 6.2 Hz, 7H), 1.12 (t, *J* = 7.6 Hz, 8H), 0.96 (t, *J* = 7.5 Hz, 2H) ppm; ^{13}C NMR (75

MHz, $CDCl_3$) δ 174.5 (s), 174.5 (s), 118.1 (s), 96.1 (s), 93.9 (s), 91.8 (s), 89.6 (s), 64.1 (s), 64.0 (s), 36.4 (d, J = 20.8 Hz), 31.1 (d, J = 21.3 Hz), 28.4 (s), 28.0 (d, J = 21.4 Hz), 27.5 (s), 24.5 (d, J = 4.3 Hz), 21.6 (d, J = 4.9 Hz), 20.9 (d, J = 22.8 Hz), 9.1 (s) ppm; ^{19}F NMR (282 MHz, $CDCl_3$) δ -173.47, -182.6 ppm.
HRMS (+APCI) (*m/z*) $[M+NH_4]^+$: exact mass calc. for $C_9H_{17}FO_2$: 194.1551, found: 194.1554.

(3a*R*,9a*S*,9b*R*)-8-Fluoro-3a,6,6,9a-tetramethyldecahydronaphtho[2,1-b]furan-2(1*H*)-one (2l)

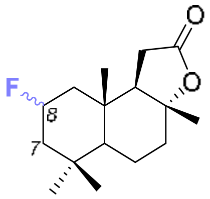

Prepared from (3a*R*)-(+)-Sclareolide according to **General Procedure A**. Purification was conducted via column chromatography (10% EtOAc in PE) that afforded the mixture of two isomers in 10:3 (8F : 7F) ratio (based on ^{19}F NMR) as white solid. Yield: 118.0 mg, 78%.
^1H NMR (400 MHz, $CDCl_3$) δ 5.02 – 4.83 (m, 1H), 4.83 – 4.63 (m, 1H), 2.49 – 2.29 (m, 3H), 2.22 (dddd, J = 10.2, 8.0, 5.7, 1.9 Hz, 3H), 2.14 – 1.56 (m, 19H), 1.58 – 1.35 (m, 4H), 1.35 – 1.04 (m, 19H), 1.04 – 0.76 (m, 28H) ppm; ^{13}C NMR (101 MHz, $CDCl_3$) δ 176.4 (s), 176.0 (s), 98.0 (s), 96.3 (s), 88.4 (s), 86.7 (s), 86.0 (s), 85.8 (s), 59.1 (s), 58.7 (s), 58.5 (s), 55.9 (d, J = 7.4 Hz), 49.4 (s), 47.8 (d, J = 16.0 Hz), 45.3 (t, J = 18.6 Hz), 42.8 (d, J = 17.6 Hz), 38.4 (d, J = 19.5 Hz), 37.3 (dd, J = 27.0, 14.7 Hz), 35.5 (s), 35.3 (s), 34.9 (d, J = 12.2 Hz), 33.5 (s), 33.2 (s), 32.9 (s), 32.6 (s), 32.2 (s), 28.6 (d, J = 10.4 Hz), 27.7 (d, J = 8.2 Hz), 23.2 (s), 23.0 (s), 22.3 (d, J = 2.0 Hz), 21.6 (t, J = 12.5 Hz), 20.3 – 19.9 (m), 16.1 (s), 15.8 (d, J = 6.6 Hz), 14.8 (s) ppm; ^{19}F NMR (377 MHz, $CDCl_3$) δ -180.2 (dd, J = 47.9, 4.2 Hz) 1F, -187.7 (td, J = 46.3, 13.5 Hz) 2F ppm; HRMS (ESI) (*m/z*) $[M+H]^+$: exact mass calc. for $C_{16}H_{25}FO_2$: 269.1911, found: 269.1921.
Data are consistent with the literature.[28a]

1-((1*r*,3*s*,5*R*,7*S*)-3-Fluoroadamantan-1-yl)ethan-1-one (2m)

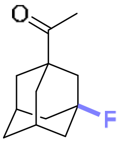

Prepared from 1-adamantyl methyl ketone according to **General Procedure A**. Purification was conducted via column chromatography (5% EtOAc in PE) that afforded the product as slightly yellow solid. Yield: 86.0 mg, 78%.
^1H NMR (300 MHz, $CDCl_3$) δ 2.41-2.3 (m, 2H) 2.10 (s, 3H), 1.91 (d, J = 5.8 Hz, 2H), 1.88-1.81 (m, 4H), 1.73-1.65 (m, 4H), 1.62-1.55 (m, 2H) ppm; ^{13}C NMR (75 MHz, $CDCl_3$) δ 211.4 (d, J = 1.7 Hz), 206.9 (s), 206.5 (s), 92.4 (d, J = 184.5 Hz), 50.8 (d, J = 9.4 Hz), 43.1 (d, J = 19.5 Hz), 41.8 (d, J = 17.5 Hz), 36.9 (d, J = 1.9 Hz), 34.8 (d, J = 2.0 Hz), 30.8 (d, J = 10.0 Hz), 24.6 (s) ppm; ^{19}F NMR (376 MHz, $CDCl_3$) δ -132.6 ppm. HRMS (EI) (*m/z*) $[M]^+$: exact mass calc. for $C_{12}H_{17}FO$: 196.1263, found: 196.1252.
Data are consistent with the literature.[28a]

(1*R*,3*S*,5*s*,7*s*)-5-Fluoroadamantan-2-one (2n)

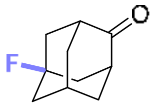

Prepared from 2-adamantanone according to **General Procedure B**. Purification was conducted via column chromatography (5% EtOAc in PE) that afforded the product as yellow solid. Yield: 29.0 mg, 62%.

^{1}H NMR (400 MHz, $CDCl_3$) δ 2.68 (s, 2H), 2.43 (s, 1H), 2.22 (s, 2H), 2.17 – 2.03 (m, 4H), 2.02 – 1.89 (m, 4H) ppm; ^{13}C NMR (101 MHz, $CDCl_3$) δ 214.9 (d, *J* = 1.5 Hz), 90.2 (d, *J* = 185.9 Hz), 47.1 (d, *J* = 10.3 Hz), 42.1 (d, *J* = 20.2 Hz), 41.6 (d, *J* = 17.7 Hz), 38.0 (d, *J* = 2.1 Hz), 30.5 (d, *J* = 9.9 Hz) ppm; ^{19}F NMR (377 MHz, $CDCl_3$) δ -140.8 ppm. HRMS (EI) (*m/z*) $[M]^+$: exact mass calc. for $C_{10}H_{13}FO$: 168.0950, found: 168.0945.

Data are consistent with the literature.[29]

1,10-dibromo-5-fluorodecane (2q)

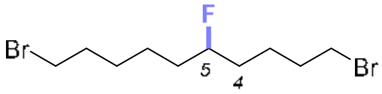

Prepared from 1,10-dibromodecane according to **General Procedure B**. Purification was conducted via column chromatography (1% DCM in PE) that afforded the mixture of two isomers in 2 : 1 (5F : 4F) ratio (based on ^{19}F NMR) as colorless viscous liquid. Yield: 49.0 mg, 55%.

IR (neat) ν (cm^{-1}): 2937, 2862, 1461, 1433, 1390, 1353, 1244, 1151, 1054, 969, 846, 805, 767, 730; ^{1}H NMR (400 MHz, $CDCl_3$) δ 4.61 – 4.35 (m, 1H), 3.51 – 3.36 (m, 4H), 1.97 – 1.30 (m, 14H) ppm; ^{13}C NMR (101 MHz, $CDCl_3$) δ 94.7 (s), 94.3 (s), 93.0 (s), 92.6 (s), 35.1 (s), 35.0 (s), 34.9 (s), 34.8 (s), 34.2 (d, *J* = 21.1 Hz), 33.8 (s), 33.7 (s), 33.7 (s), 33.5 (s), 33.5 (s), 32.6 (s), 32.5 (d, *J* = 11.8 Hz), 29.7 (s), 28.5 (s), 28.4 (d, *J* = 3.8 Hz), 28.0 (s), 27.9 (s), 24.9 (d, *J* = 4.6 Hz), 24.3 (d, *J* = 4.3 Hz), 23.8 (d, *J* = 4.4 Hz) ppm; ^{19}F NMR (377 MHz, $CDCl_3$) δ -181.4 (5F), -181.7 (4F) ppm.

HRMS (ESI) (*m/z*) $[M-HF]^+$: exact mass calc. for $C_{10}H_{19}Br_2F$: 295.9770 found: 295.9777.

5-Fluorodecanedinitrile (2r)

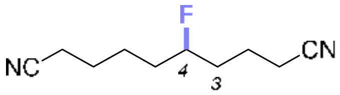

Prepared from decanedinitrile according to **General Procedure A**. Purification was conducted via column chromatography (30% EtOAc in PE) that afforded the mixture of two isomers in 5 : 1 (4F : 3F) ratio (based on ^{19}F NMR) as white solid. Yield: 42.0 mg, 41%.

^{1}H NMR (400 MHz, $CDCl_3$) δ 4.69 – 4.39 (m, 1H), 2.36 (ddd, *J* = 11.9, 9.6, 5.9 Hz, 4H), 1.90 – 1.44 (m, 10H) ppm; ^{13}C NMR (101 MHz, $CDCl_3$) δ 119.7 (d, *J* = 16.0 Hz), 119.4 (d, *J* = 14.6 Hz), 119.0 (s), 93.8 (s), 92.6 (s), 92.1 (s), 90.9 (s), 34.5 (s), 34.3 (s), 34.3 (s), 34.1 (s), 33.8 (d, *J* = 21.2 Hz), 30.9 (d, *J* = 21.5 Hz), 28.4

(d, J = 2.7 Hz), 28.3 (s), 25.2 (s), 25.1 (s), 25.1 (s), 24.3 (d, J = 4.2 Hz), 24.2 (d, J = 4.2 Hz), 21.3 (d, J = 3.8 Hz), 17.1 (s), 17.0 (s), 17.0 (s), 13.3 (d, J = 5.0 Hz) ppm; ^{19}F NMR (377 MHz, $CDCl_3$) δ -182.9 (4F), -185.4 (3F) ppm. HRMS (ESI) (*m/z*) $[M-H]^+$: exact mass calc. for $C_{10}H_{15}FN_2$: 181.1141, found: 181.1144.
Data are consistent with the literature.[28a]

(3*s*,5*s*,7*s*)-1-Fluoroadamantane (2w)

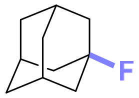

Prepared from adamantane according to **General Procedure A**. Purification was conducted via column chromatography (100% PE) that afforded the product as white solid. Yield: 50.0 mg, 58%.
^{1}H NMR (400 MHz, $CDCl_3$) δ 2.23 (s, 3H), 1.89 (dd, J = 5.6, 3.0 Hz, 6H), 1.69 – 1.58 (m, 6H) ppm; ^{13}C NMR (101 MHz, $CDCl_3$) δ 92.5 (d, J = 183.3 Hz), 42.7 (d, J = 17.0 Hz), 35.8 (d, J = 2.1 Hz), 31.4 (d, J = 9.7 Hz) ppm; ^{19}F NMR (377 MHz, $CDCl_3$) δ -128.9 ppm. HRMS (EI) (*m/z*) $[M]^+$: exact mass calc. for $C_{10}H_{15}F$: 154.1158, found: 154.1155.
Data are consistent with the literature.[30]

(1*r*,3*s*,5*R*,7*S*)-3-Fluoroadamantan-1-ol (2b)

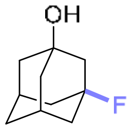

Prepared from 1-adamantanol according to **General Procedure A**. Purification was conducted via column chromatography (20% EtOAc in PE) that afforded the product as white solid. Yield: 87.0 mg, 91%.
^{1}H NMR (400 MHz, $CDCl_3$) δ 2.35 (s, 2H), 1.93 (s, 1H), 1.88 (d, J = 5.7 Hz, 2H), 1.80 (dd, J = 5.0, 3.3 Hz, 4H), 1.64 (s, 4H), 1.48 (d, J = 2.6 Hz, 2H) ppm; ^{13}C NMR (101 MHz, $CDCl_3$) δ 93.3 (d, J = 185.6 Hz), 71.0 (d, J = 11.9 Hz), 50.4 (d, J = 17.1 Hz), 43.7 (d, J = 1.5 Hz), 41.3 (d, J = 17.6 Hz), 34.4 (d, J = 2.1 Hz), 31.3 (d, J = 10.3 Hz) ppm; ^{19}F NMR (376 MHz, $CDCl_3$) δ -133.4 ppm.
HRMS (EI) (*m/z*) $[M]^+$: exact mass calc. for monofluorinated product $C_{10}H_{15}FO$: 170.1107, found: 170.1103.

Data are consistent with the literature.[28a]

4-Fluoropentyl benzoate (2a)

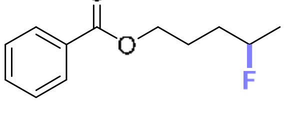

Prepared from pentyl benzoate according to **General Procedure A**. Purification was conducted via column chromatography (2% EtOAc in PE) that afforded the product as colorless oil. Yield: 63.0 g, 54%.

^{1}H NMR (400 MHz, $CDCl_3$) δ 8.04 (d, *J* = 7.7 Hz, 2H), 7.56 (t, *J* = 7.4 Hz, 1H), 7.44 (t, *J* = 7.7 Hz, 2H), 4.87 – 4.60 (m, 1H), 4.43 – 4.28 (m, 2H), 2.03 – 1.62 (m, 4H), 1.36 (dd, *J* = 23.8, 6.2 Hz, 3H) ppm; ^{13}C NMR (101 MHz, $CDCl_3$) δ 166.5 (s), 132.9 (s), 130.3 (s), 128.9 (d, *J* = 118.4 Hz), 90.4 (d, *J* = 165.2 Hz), 64.6 (s), 33.5 (d, *J* = 21.2 Hz), 24.5 (d, *J* = 4.7 Hz), 21.0 (d, *J* = 22.7 Hz) ppm; ^{19}F NMR (377 MHz, $CDCl_3$) δ -173.9 ppm. HRMS (+APCI) (*m/z*) $[M+H]^+$: exact mass calc. for $C_{12}H_{15}FO_2$: 211.1129, found: 211.1132.

Data are consistent with the literature.[28a]

4-Fluoropentyl 4-methylbenzenesulfonate (2f)

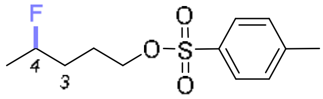

Prepared from 4-*n*-pentyl 4-methylbenzenesulfonate according to **General Procedure A**. Purification was conducted via column chromatography (10% EtOAc in PE) that afforded the mixture of 2 isomers of the product in 5 : 1 (4F : 3F) ratio (based on ^{19}F NMR) as yellow viscous liquid. Yield: 60.0 mg, 41%.

NMR data of the major product is provided.
IR (neat) ν (cm^{-1}): 2981, 2933, 1599, 1494, 1446, 1356, 1174, 1095, 1021, 969, 916, 812, 738, 663; ^{1}H NMR (400 MHz, $CDCl_3$) δ 7.78 (d, *J* = 8.3 Hz, 4H), 7.34 (d, *J* = 8.0 Hz, 4H), 4.59 (ddq, *J* = 49.0, 12.3, 6.1 Hz, 2H), 4.05 (qt, *J* = 9.8, 6.3 Hz, 4H), 2.44 (s, 6H), 1.93 – 1.50 (m, 9H), 1.28 (dd, *J* = 23.8, 6.2 Hz, 6H) ppm; ^{13}C NMR (101 MHz, $CDCl_3$) δ 144.8 (s), 133.0 (s), 128.8 (d, *J* = 201.0 Hz), 90.0 (d, *J* = 165.5 Hz), 70.1 (s), 32.7 (d, *J* = 21.1 Hz), 24.7 (d, *J* = 4.3 Hz), 21.6 (s), 20.9 (d, *J* = 22.6 Hz) ppm; ^{19}F NMR (376 MHz, $CDCl_3$) δ -174.7 ppm.
HRMS (ESI) (*m/z*) $[M+Na]^+$: exact mass calc. for $C_{12}H_{17}FO_3S$: 283.0775, found: 283.0777.

3-Fluoro-3-methylbutyl benzoate (2c)

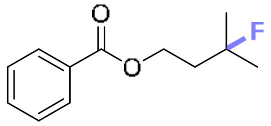

Prepared from isopentyl benzoate according to **General Procedure B**. Purification was conducted via column chromatography (2% EtOAc in PE) that afforded the product as yellow viscous liquid. Yield: 21.0 mg, 35%.
^{1}H NMR (400 MHz, $CDCl_3$) δ 8.06 – 8.00 (m, 2H), 7.56 (t, *J* = 7.4 Hz, 1H), 7.44 (t, *J* = 7.7 Hz, 2H), 4.48 (t, *J* = 6.8 Hz, 2H), 2.13 (dt, *J* = 19.4, 6.8 Hz, 2H), 1.45 (d, *J* = 21.5 Hz, 6H) ppm; ^{13}C NMR (101 MHz, $CDCl_3$) δ 166.5 (s), 132.9 (s), 130.2 (s), 129.5 (s), 128.4 (s), 94.2 (d, *J* = 166.1 Hz), 60.9 (d, *J* = 6.2 Hz), 39.8 (d, *J* = 23.1 Hz), 27.1 (d, *J* = 24.6 Hz) ppm; ^{19}F NMR (376 MHz, $CDCl_3$) δ -138.6 ppm. HRMS (+APCI) (*m/z*) $[M+H]^+$: exact mass calc. for $C_{12}H_{15}FO_2$: 211.1129, found: 211.1131.
Data are consistent with the literature.[31a]

4-Phenylbutyl benzoate (17)

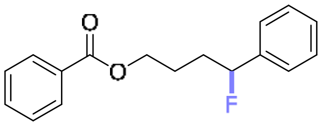

According to **General Procedure A** (with 150 mol% **MFB**). Yield: 69.0 mg, 45%; colorless oil; IR (neat) ν (cm^{-1}): 3064, 3034, 2956, 1715, 1603, 1495, 1454, 1387, 1271, 1178, 1115, 1070, 1029, 951, 850, 805, 760, 712, ; ^{1}H NMR (400 MHz, $CDCl_3$) δ 7.94 (dd, *J* = 5.2, 3.3 Hz, 2H), 7.58 – 7.42 (m, 1H), 7.41 – 7.19 (m, 7H), 5.43 (ddd, *J* = 47.5, 8.1, 4.0 Hz, 1H), 4.42 – 4.15 (m, 2H), 2.14 – 1.70 (m, 4H) ppm; ^{13}C NMR (101 MHz, $CDCl_3$) δ 166.6 (s), 140.0 (d, *J* = 19.8 Hz), 133.0 (s), 130.3 (s), 129.6 (s), 128.6 (s), 128.4 (s), 125.5 (d, *J* = 6.9 Hz), 94.1 (d, *J* = 171.3 Hz), 64.5 (s), 33.8 (d, *J* = 24.1 Hz), 24.6 (d, *J* = 4.1 Hz) ppm; ^{19}F NMR (377 MHz, $CDCl_3$) δ -175.8 – -176.3 (m) ppm; HRMS (ESI) (*m/z*) $[M]^+$: exact mass calc. for $C_{17}H_{17}FO_2$: 272.1213, found: 272.1205.

(1*R*,2*S*)-2-Chloro-11-fluoro-1-phenyldodecyl diphenylphosphinate (2g)

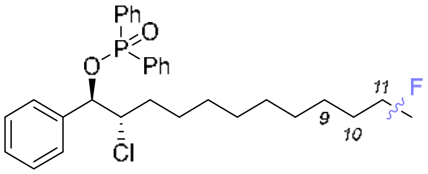

Prepared from (1*R*,2*S*)-2-Chloro-1-phenyldodecyl diphenylphosphinate according to **General Procedure A.** Purification was conducted via column chromatography (50% EtOAc in PE) that afforded the mixture of several isomers (difficult to assign fluorine positions) as colorless viscous liquid. Yield: 218.0 mg, 75%.

IR (neat) ν (cm^{-1}): 3060, 2929, 2855, 1591, 1494, 1438, 1382, 1259, 1230, 1129, 987, 920, 857, 805, 753, 730, 693; ^{1}H NMR (400 MHz, $CDCl_3$) δ 7.88 (dd, *J* = 12.5, 7.6 Hz, 2H), 7.67 – 7.52 (m, 3H), 7.48 (dt, *J* = 10.3, 5.3 Hz, 2H), 7.39 (t, *J* = 7.4 Hz, 1H), 7.34 – 7.18 (m, 7H), 5.48 (dd, *J* = 9.5, 5.7 Hz, 1H), 4.74 – 4.29 (m, 1H), 4.26 (dd, *J* = 9.2, 3.7 Hz, 1H), 1.79 – 1.14 (m, 17H), 0.95 (dt, *J* = 6.6, 5.5 Hz, 1H) ppm; ^{13}C NMR (101 MHz, $CDCl_3$) δ 137.2 – 135.5 (m), 132.4 (s), 132.2 (d, *J* = 2.6 Hz), 131.9 (d, *J* = 2.6 Hz), 131.8 (d, *J* = 10.5 Hz), 131.6 (d, *J* = 10.3 Hz), 131.4 (s), 131.0 (s), 130.4 (d, *J* = 4.3 Hz), 128.4 (d, *J* = 13.2 Hz), 128.1 (dd, *J* = 8.1, 5.1 Hz), 127.5 (s), 96.5 (s), 95.4 – 94.7 (m), 93.6 – 93.1 (m), 91.0 (d, *J* = 163.9 Hz), 79.8 – 79.1 (m), 65.7 – 65.0 (m), 37.2 (d, *J* = 20.9 Hz), 36.9 (d, *J* = 20.5 Hz), 35.4 – 34.3 (m), 33.6 – 32.9 (m), 31.7 (dd, *J* = 12.6, 9.1 Hz), 30.0 – 28.9 (m), 28.9 – 28.4 (m), 28.0 (d, *J* = 21.4 Hz), 27.2 (d, *J* = 4.3 Hz), 26.4 – 25.7 (m), 25.3 – 24.5 (m), 24.5 – 24.2 (m), 22.6 (dd, *J* = 9.4, 3.2 Hz), 21.0 (d, *J* = 22.8 Hz), 18.4 (d, *J* = 4.8 Hz), 14.2 – 13.8 (m), 9.4 (d, *J* = 5.8 Hz) ppm; ^{31}P NMR (162 MHz, $CDCl_3$) δ 33.32, 33.30, 33.19, 33.15, 33.11, 33.07, 33.03, 33.03, 33.00 ppm; ^{19}F NMR (377 MHz, $CDCl_3$) δ -172.5, -172.6, -180.6, -180.6, -180.7, -180.8, -180.9, -181.0, -181.7, -181.8 ppm.
HRMS (ESI) (*m/z*) $[M+H]^+$: exact mass calc. for $C_{30}H_{37}ClFO_2P$: 515.2276, found: 515.2281.

Methyl (4*R*)-4-((5*S*,8*S*,9*S*,10*S*,13*R*,14*S*,17*R*)-1-fluoro-10,13-dimethyl-3,7,12-trioxohexadecahydro-1*H*-cyclopenta[a]phenanthren-17-yl)pentanoate (2o)

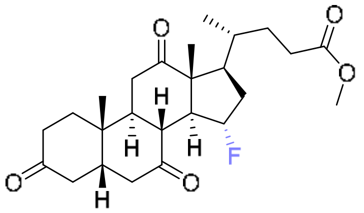

Prepared from Methyl (*R*)-4-((5*S*,8*R*,9*S*,10*S*,13*R*,14*S*,17*R*)-10,13-dimethyl-3,7,12-trioxohexadecahydro-1*H*-cyclopenta[a]phenanthren-17-yl)pentanoate according to **General Procedure A**. Purification was conducted via column chromatography (50% EtOAc in PE) that afforded the product as white solid. Yield: 132.0 mg, 54%.

IR (neat) ν (cm^{-1}): 2955, 2262, 1707, 1461, 1435, 1386, 1274, 1248, 1174, 1103, 834, 771, 726, 685; ^1H NMR (400 MHz, $CDCl_3$) δ 5.01 – 4.75 (m, 1H), 3.64 (s, 3H), 3.11 (t, *J* = 11.7 Hz, 1H), 2.92 (dd, *J* = 13.0, 5.8 Hz, 1H), 2.78 (t, *J* = 12.8 Hz, 1H), 2.38 – 2.32 (m, 2H), 2.32 – 2.25 (m, 3H), 2.25 – 2.18 (m, 5H), 2.15 (dd, *J* = 7.1, 5.2 Hz, 2H), 2.08 (dd, *J* = 13.1, 2.9 Hz, 1H), 1.92 (ddd, *J* = 18.7, 9.2, 5.1 Hz, 2H), 1.84 – 1.75 (m, 1H), 1.61 (td, *J* = 14.1, 5.2 Hz, 1H), 1.41 – 1.34 (m, 4H), 1.31 – 1.21 (m, 1H), 1.08 (s, 3H), 0.81 (d, *J* = 6.6 Hz, 3H) ppm; ^{13}C NMR (101 MHz, $CDCl_3$) δ 209.8 (s), 209.0 (s), 206.8 (s), 174.2 (s), 94.8 (s), 92.9 (s), 56.9 (d, *J* = 4.9 Hz), 56.1 (d, *J* = 19.5 Hz), 51.5 (s), 47.8 (s), 46.1 (s), 45.3 (s), 45.0 (s), 43.6 (d, *J* = 1.1 Hz), 42.6 (s), 38.2 (s), 36.7 (d, *J* = 24.5 Hz), 36.2 (s), 36.1 (s), 34.9 (d, *J* = 13.7 Hz), 31.1 (s), 30.1 (s), 21.8 (s), 17.9 (s), 13.0 (s) ppm; ^{19}F NMR (377 MHz, $CDCl_3$) δ -169.4 ppm; HRMS (ESI) (*m/z*) $[M+H]^+$: exact mass calc. for $C_{25}H_{35}FO_5$: 435.2541, found: 435.2547.

Fluorinated (+)-4-Cholesten-3-on (2p)

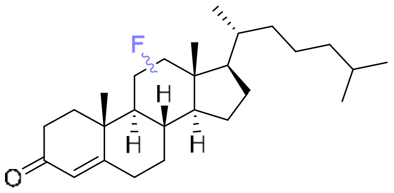

Prepared from (+)-4-cholesten-3-on according to **General Procedure A**. Purification was conducted via column chromatography (5% EtOAc in PE) that afforded the mixture of isomers (difficult to assign fluorine positions) as white solid. Yield: 52.0 mg, 23%.

IR (neat) ν (cm^{-1}): 2933, 2870, 1715, 1677, 1465, 1379, 1267, 1230, 1185, 1081, 1029, 962, 868, 779, 686; ^1H NMR (400 MHz, $CDCl_3$) δ 5.75 (d, *J* = 23.6 Hz, 1H), 5.13 – 4.63 (m, 1H), 2.49 – 2.22 (m, 4H), 2.00 (dt, *J* = 11.7, 4.9 Hz, 2H), 1.89 – 1.77 (m, 2H), 1.73 – 1.65 (m, 2H), 1.57 – 1.49 (m, 3H), 1.48 – 1.38 (m, 3H), 1.37 – 1.23 (m, 6H), 1.21 – 1.08 (m, 7H), 1.05 – 0.81 (m, 12H) ppm; ^{13}C NMR (101 MHz, $CDCl_3$) δ 199.4, 170.9, 124.8, 124.7, 123.9, 123.8, 100.9, 100.8, 99.2, 99.1, 63.9, 63.8, 63.7, 63.7, 53.5, 53.5, 52.7, 43.8, 43.8, 43.7, 43.7, 39.7, 39.4, 39.4, 39.3, 39.3, 38.5, 36.0, 35.9, 35.6, 35.5, 35.4, 34.8, 34.4, 34.0, 33.9, 33.7, 33.6,

33.2, 32.7, 31.8, 31.7, 31.4, 30.1, 29.7, 27.9, 25.7, 24.8, 24.1, 23.8, 23.7, 22.8, 22.5, 22.5, 20.8, 20.6, 18.6, 18.5, 18.5, 18.3, 17.6, 17.4, 17.3, 13.3, 13.2 ppm; ^{19}F NMR (377 MHz, $CDCl_3$) δ -163.0, -163.1, -171.8, -171.9 ppm; HRMS (ESI) (*m/z*) $[M]^+$: exact mass calc. for $C_{27}H_{44}FO_5$: 402.3298, found: 402.3277.

Methyl 2-(4-(1-fluoro-2-methylpropyl)phenyl)propanoate (2y)

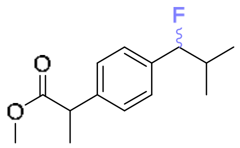

Prepared from methyl 2-(4-isobutylphenyl)propanoate according to **General Procedure A**. Purification was conducted via column chromatography (5% EtOAc in PE) that afforded the desired product as colorless oil. Yield: 74.0 mg, 55%.

^{1}H NMR (400 MHz, $CDCl_3$) δ 7.35 – 7.19 (m, 5H), 5.08 (dd, *J* = 47.0, 6.8 Hz, 1H), 3.78 – 3.69 (m, 1H), 3.66 (d, *J* = 1.2 Hz, 3H), 2.21 – 1.94 (m, 1H), 1.50 (d, *J* = 7.2 Hz, 3H), 1.05 – 0.98 (m, 3H), 0.85 (d, *J* = 6.9 Hz, 3H) ppm; ^{13}C NMR (151 MHz, $CDCl_3$) δ 175.1 (s), 174.9 (s), 140.3 (t, *J* = 2.1 Hz), 138.7 (s), 138.3 (d, *J* = 20.6 Hz), 130.6 (s), 127.3 (s), 127.2 (s), 126.4 (d, *J* = 7.0 Hz), 99.1 (d, *J* = 173.5 Hz), 52.0 (s), 52.0 (s), 47.2 (d, *J* = 22.9 Hz), 45.1 (s), 45.0 (s), 34.2 (d, *J* = 22.8 Hz), 31.6 (s), 26.6 (d, *J* = 24.5 Hz), 22.6 (s), 18.6 (d, *J* = 1.8 Hz), 18.3 (d, *J* = 5.7 Hz), 17.5 (d, *J* = 5.2 Hz), 14.1 (s) ppm; ^{19}F NMR (377 MHz, $CDCl_3$) δ -180.1 (ddd, *J* = 47.0, 17.1, 8.0 Hz) ppm; HRMS (EI) (*m/z*) $[M]^+$: exact mass calc. for $C_{14}H_{19}FO_2$: 238.1369, found: 238.1363.

Data are consistent with the literature.[31b]

4-Fluoro-4-phenylbutyl acetate (20)

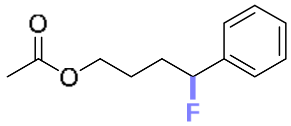

Prepared from 4-phenylbutyl acetate according to **General Procedure A** (1.5 eq. **MFB**). Purification was conducted via column chromatography (2% EtOAc in PE) that afforded the desired product as colorless oil. Yield: 35.0 mg, 29%.

IR (neat) ν (cm^{-1}): 3064, 3030, 2956, 1737, 1495, 1454, 1364, 1237, 1144, 1044, 969, 895, 764, 701; ^{1}H NMR (400 MHz, $CDCl_3$) δ 7.38 – 7.21 (m, 5H), 5.39 (ddd, *J* = 47.7, 8.2, 4.4 Hz, 1H), 4.11 – 3.96 (m, 2H), 1.98 – 1.95 (m, 3H), 1.95 – 1.58 (m, 4H) ppm; ^{13}C NMR (101 MHz, $CDCl_3$) δ 171.1 (s), 140.0 (d, *J* = 19.8 Hz), 128.5 (s), 128.4 (d, *J* = 1.9 Hz), 125.5 (d, *J* = 6.9 Hz), 94.0 (d, *J* = 171.1 Hz), 63.9 (s), 33.8 (s), 33.6 (s), 24.4 (d, *J* = 4.2 Hz), 20.9 (s) ppm; ^{19}F NMR (377 MHz, $CDCl_3$) δ -175.9 – -176.4 (m); HRMS (EI) (*m/z*) $[M]^+$: exact mass calc. for $C_{12}H_{15}FO_2$: 210.1056, found: 210.1060.

1-((5)-2-Ethyl-5-fluorohexyl)-1-methylpyrrolidin-1-ium hexafluorophosphate (2aa)

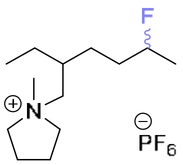

Prepared from 1-(2-ethylhexyl)-1-methylpyrrolidin-1-ium hexafluorophosphate according to **General Procedure A** (2.0 eq. **SF**). Purification was conducted via column chromatography (10% MeOH in DCM) that afforded the desired product as colorless oil. Yield: 163.0 mg, 80%.

IR (neat) ν (cm^{-1}): 2963, 2930, 2974, 1465, 1387, 1059, 913, 831, 727; ^{1}H NMR (400 MHz, $CDCl_3$) δ 4.80 – 4.53 (m, 1H), 3.51 (td, *J* = 11.5, 5.1 Hz, 4H), 3.33 – 3.11 (m, 2H), 3.03 (s, 3H), 2.24 (s, 4H), 1.63 (ddd, *J* = 15.1, 13.1, 7.2 Hz, 1H), 1.54 – 1.41 (m, 3H), 1.38 – 1.21 (m, 4H), 0.92 (tt, *J* = 13.9, 4.7 Hz, 5H) ppm; ^{13}C NMR (101 MHz, $CDCl_3$) δ 91.8 (d, *J* = 25.0 Hz), 90.2 (d, *J* = 24.9 Hz), 77.3 (s), 68.2 (s), 53.5 (s), 48.2 (s), 34.6 (d, *J* = 20.1 Hz), 33.1 (s), 32.9 (d, *J* = 3.8 Hz), 32.6 (s), 28.2 (s), 28.2 (s), 28.1 (s), 25.6 (d, *J* = 4.0 Hz), 21.0 (d, *J* = 7.8 Hz), 20.8 (d, *J* = 7.8 Hz), 9.9 (s) ppm; ^{19}F NMR (377 MHz, None) δ -71.7 (d, *J* = 707.5 Hz), -171.6 – -172.7 (m) ppm; HRMS (EI) (*m/z*) $[M]^+$: exact mass calc. for $C_{13}H_{27}FN$: 216.2122, found: 216.2128.

5-(6-fluoroheptyl)dihydrofuran-2(3H)-one (2ab)

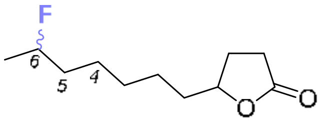

C_6:C_5:C_4 = 2:1.5:1

Prepared from 5-heptyldihydrofuran-2(3*H*)-one according to **General Procedure A**. Purification was conducted via column chromatography (10% EtOAc in PE) that afforded the mixture of isomers (6F:5F:4F = 2:1.5:1) as colorless oil. Yield: 65.0 mg, 57%.

IR (neat) ν (cm^{-1}): 2937, 2863, 1771, 1461, 1424, 1387, 1353, 1286, 1178, 1129, 1014, 977, 917, 839, 805, 731; ^{1}H NMR (400 MHz, $CDCl_3$) δ 4.78 – 4.26 (m, 2H), 2.59 – 2.43 (m, 2H), 2.41 – 2.24 (m, 1H), 1.90 – 1.79 (m, 1H), 1.77 – 1.55 (m, 4H), 1.52 – 1.19 (m, 8H), 0.94 (t, *J* = 7.5 Hz, 1H) ppm; ^{13}C NMR (101 MHz, $CDCl_3$) δ 177.2 (s), 177.2 (s), 177.1 (d, *J* = 0.5 Hz), 177.0 (s), 96.2 (d, *J* = 1.5 Hz), 95.0 (s), 94.8 (s), 94.7 (s), 94.6 (d, *J* = 1.6 Hz), 94.4 (s), 93.3 (s), 93.1 (d, *J* = 13.5 Hz), 92.7 (s), 90.9 (dd, *J* = 164.2, 1.0 Hz), 80.9 (d, *J* = 1.3 Hz), 80.9 (s), 80.8 (s), 80.7 (s), 80.2 (s), 77.3 (s), 60.4 (s), 37.2 (dd, *J* = 20.8, 1.2 Hz), 36.7 (d, *J* = 20.7 Hz), 35.5 (s), 35.5 (s), 35.3 (s), 35.0 (s), 35.0 (s), 34.8 (d, *J* = 2.9 Hz), 34.7 (s), 34.6 (s), 34.6 (s), 34.5 (s), 34.4 (d, *J* = 3.8 Hz), 31.9 (s), 31.8 (d, *J* = 3.6 Hz), 31.5 (s), 31.3 (s), 31.0 (d, *J* = 3.9 Hz), 30.6 (d, *J* = 21.3 Hz), 29.7 (d, *J* = 4.3 Hz), 29.3 (s), 29.2 (s), 29.1 (s), 29.0 (s), 28.8 (s), 28.8 (s), 28.7 (s), 28.6 (s), 28.2 (d, *J* = 1.2 Hz), 28.0 (s), 28.0 (s), 27.9 (s), 27.9 (s), 27.3 – 27.1 (m), 25.2 (d, *J* = 0.8 Hz), 24.9 (s), 24.9 (d, *J* = 0.6 Hz), 24.8 (s), 22.7 (s), 22.5 (s), 21.2 (d, *J* = 4.3 Hz), 21.1 (d, *J* = 0.7 Hz), 21.0 (s), 21.0 (s), 20.9 (d, *J* = 0.9 Hz), 18.3 (dd, *J* = 4.7, 0.9 Hz), 14.2 (s), 14.1 (s), 13.9 (s), 13.9 (s), 9.4 (d, *J* = 5.8 Hz) ppm; ^{19}F NMR (377

MHz, $CDCl_3$) δ -172.7 – -173.4 (m), -181.1 – -181.9 (m), -181.9 – -182.4 (m); HRMS (EI) (*m/z*) $[M+NH_4]^+$: exact mass calc. for $C_{11}H_{19}FO_2$: 220.1713, found: 220.1708.

1-(6-Fluoroheptyl)-3-methyl-1*H*-imidazol-3-ium tetrafluoroborate (2ac)

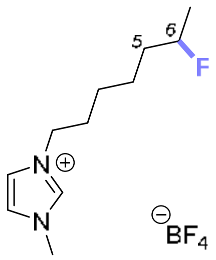

C_6:C_5 = 2:1

Prepared from 1-heptyl-3-methyl-1*H*-imidazol-3-ium tetrafluoroborate according to **General Procedure A** (2.0 eq. **SF**). Purification was conducted via column chromatography (10% MeOH in DCM) that afforded the mixture of two isomers (6F:5F = 2:1) as colorless oil. Yield: 158.0 mg, 98%.

IR (neat) ν (cm^{-1}): 3638, 3161, 3124, 2926, 2859, 1625, 1573, 1461, 1387, 1286, 1170, 1036, 917, 850, 731; ^1H NMR (400 MHz, $CDCl_3$) δ 8.60 (s, 1H), 7.33 – 7.17 (m, 2H), 4.62 – 4.13 (m, 1H), 4.09 – 3.97 (m, 2H), 3.79 (s, 3H), 2.01 – 1.65 (m, 3H), 1.53 – 1.22 (m, 5H), 1.16 (ddd, *J* = 16.7, 9.7, 4.0 Hz, 3H), 0.90 – 0.76 (m, 1H) ppm; ^{13}C NMR (101 MHz, $CDCl_3$) δ 136.2 (s), 123.7 (s), 123.0 (s), 122.3 (s), 122.3 (s), 122.2 (s), 120.8 (s), 95.3 (d, *J* = 167.1 Hz), 90.9 (d, *J* = 163.9 Hz), 90.1 (s), 50.0 (s), 49.8 (s), 49.7 (s), 46.6 (s), 36.4 (d, *J* = 20.7 Hz), 36.2 (s), 34.3 (s), 33.8 (d, *J* = 21.0 Hz), 33.3 (s), 31.5 (s), 30.2 (s), 30.0 (s), 29.9 (s), 29.8 (s), 28.5 (s), 28.0 (d, *J* = 21.2 Hz), 26.1 (s), 25.8 (s), 24.4 (d, *J* = 4.7 Hz), 22.5 (s), 22.1 (s), 21.9 (d, *J* = 4.1 Hz), 21.0 (d), 20.1 (s), 17.4 (s), 14.0 (s), 13.6 (s), 9.3 (d, *J* = 5.8 Hz) ppm; ^{19}F NMR (377 MHz, $CDCl_3$) δ -151.7 – -151.8 (m), -172.6 – -173.9 (m), -181.95 – -183.00 (m) ppm; ^{11}B NMR (128 MHz, $CDCl_3$) δ -1.23 (s) ppm; HRMS (EI) (*m/z*) $[M]^+$: exact mass calc. for $C_{11}H_{20}FN_2$: 199.1605, found: 199.1607.

1-(3-Fluoro-3-phenylpropyl)-3-methyl-1*H*-imidazol-3-ium hexafluorophosphate (2ad)

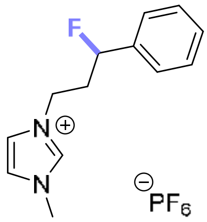

Prepared from 3-methyl-1-(3-phenylpropyl)-1*H*-imidazol-3-ium hexafluorophosphate according to **General Procedure A** (2.0 eq. **SF**). Purification was conducted via column chromatography (10% MeOH in DCM) that afforded the desired product as colorless oil. Yield: 144.0 mg, 70%.

IR (neat) ν (cm^{-1}): 3653, 3168, 3123, 2922, 2851, 2263, 1737, 1685, 1577, 1498, 1457, 1368, 1215, 1170, 1096, 1055, 988, 917, 828, 753, 701; ^1H NMR (400 MHz, CD_3CN) δ 8.15 (s, 1H), 7.22 – 7.09 (m, 6H), 5.31 (ddd, *J* = 48.0, 9.1, 3.7 Hz, 1H), 4.09 (t, *J* = 7.1 Hz, 2H), 3.56 (s, 3H), 2.35 – 2.04 (m, 2H) ppm; ^{13}C NMR

(101 MHz, CD_3CN) δ 139.4 (d, *J* = 19.3 Hz), 136.8 (s), 129.5 (d, *J* = 2.2 Hz), 129.4 (s), 129.3 (s), 128.6 (s), 126.2 (d, *J* = 6.7 Hz), 123.7 (d, *J* = 130.1 Hz), 117.9 (s), 92.1 (d, *J* = 169.2 Hz), 46.6 (d, *J* = 4.3 Hz), 37.0 (d, *J* = 23.8 Hz), 36.4 (s) ppm; ^{19}F NMR (377 MHz, CD_3CN) δ -71.6 (d, *J* = 706.7 Hz), -176.1 – -176.5 (m) ppm; ^{31}P NMR (162 MHz, CD_3CN) δ -143.1 (hept, *J* = 706.8 Hz) ppm; HRMS (EI) (*m/z*) $[M]^+$: exact mass calc. for $C_{13}H_{16}FN_2$: 219.1292, found: 219.1297.

1-(3-Fluorobutyl)-2,3-dimethyl-1*H*-imidazol-3-ium bis((trifluoromethyl)sulfonyl)amide (2ae)

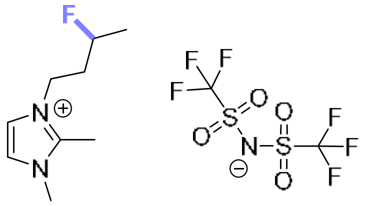

Prepared from 1-butyl-2,3-dimethyl-1*H*-imidazol-3-ium bis((trifluoromethyl)sulfonyl)amide according to **General Procedure A** (2.0 eq. **SF**). Purification was conducted via column chromatography (10% MeOH in DCM) that afforded the desired product as colorless oil. Yield: 173.0 mg, 68%.

IR (neat) ν (cm^{-1}): 3153, 2941, 2263, 1592, 1543, 1465, 1349, 1178, 1133, 1051, 965, 928, 883, 846, 790, 738; ^1H NMR (400 MHz, CD_3CN) δ 7.18 – 7.01 (m, 2H), 4.67 – 4.36 (m, 1H), 4.03 (t, 2H), 3.56 (s, 3H), 2.37 (s, 3H), 1.97 – 1.83 (m, 2H), 1.20 (dd, *J* = 24.2, 6.2 Hz, 3H) ppm; ^{13}C NMR (101 MHz, CD_3CN) δ 145.3 (s), 125.3 (s), 122.9 (d, *J* = 19.3 Hz), 122.1 (s), 121.4 (d, *J* = 8.9 Hz), 118.9 (s), 117.9 (s), 115.7 (s), 88.6 (d, *J* = 163.9 Hz), 48.6 (s), 45.2 (d, *J* = 4.5 Hz), 36.6 (s), 36.4 (s), 35.3 (d, *J* = 7.0 Hz), 31.8 (s), 20.7 (s), 20.4 (s), 19.7 (s), 13.3 (s), 9.6 (s) ppm; ^{19}F NMR (377 MHz, CD_3CN) δ -78.9 (s), -175.7 – -176.2 (m) ppm; HRMS (EI) (*m/z*) $[M]^+$: exact mass calc. for $C_9H_{16}FN_2$: 171.1292, found: 171.1295.

***N*-(2-((S)-2-cyanopyrrolidin-1-yl)-2-oxoethyl)-4-fluoro-*N*-((1R,3R,5S,7R)-3-fluoro-5-hydroxyadamantan-1-yl)benzamide (11g)**

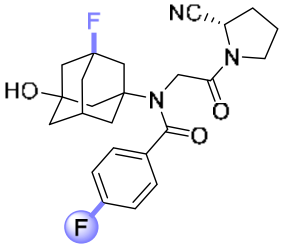

Prepared from *N*-(2-((S)-2-cyanopyrrolidin-1-yl)-2-oxoethyl)-4-fluoro-*N*-((1r,3R,5R,7S)-3-hydroxyadamantan-1-yl)benzamide according to **General Procedure C**. Purification was conducted via column chromatography (50% EtOAc in PE) that afforded desired product as white solid. Yield: 143.0 mg, 57%.

IR (neat) ν (cm^{-1}): 3407, 2922, 2863, 1707, 1636, 1510, 1446, 1394, 1357, 1323, 1260, 1223, 1159, 1036, 1003, 958, 850, 768, 712, 678; ^1H NMR (400 MHz, $CDCl_3$) δ 7.43 – 7.10 (m, 2H), 6.97 (td, *J* = 8.6, 5.4 Hz, 2H), 4.77 – 4.56 (m, 1H), 3.89 (q, *J* = 18.6 Hz, 2H), 2.98 (d, *J* = 6.0 Hz, 2H), 2.60 – 1.32 (m, 18H) ppm; ^{13}C

NMR (101 MHz, $CDCl_3$) δ 172.3 (s), 172.2 (s), 168.8 (s), 168.6 (d, J = 1.1 Hz), 164.1 (s), 164.0 (s), 161.6 (s), 161.6 (s), 161.5 (s), 135.3 (d, J = 3.5 Hz), 134.8 (d, J = 3.4 Hz), 128.1 – 127.8 (m), 117.8 (s), 117.8 (s), 115.5 (ddd, J = 18.8, 12.2, 6.7 Hz), 94.1 (d, J = 4.2 Hz), 92.3 (d, J = 4.3 Hz), 77.4 (s), 70.6 (d, J = 12.8 Hz), 61.8 (d, J = 12.4 Hz), 60.2 (s), 49.3 (dd, J = 11.5, 5.2 Hz), 49.1 (s), 49.0 (s), 47.2 (s), 46.0 (d, J = 4.2 Hz), 43.9 (d, J = 15.0 Hz), 43.2 (d, J = 19.7 Hz), 42.4 (d, J = 15.7 Hz), 40.3 (d, J = 17.9 Hz), 36.5 (d, J = 8.5 Hz), 29.9 (s), 29.7 (d, J = 11.4 Hz), 29.6 (s) ppm; ^{19}F NMR (377 MHz, None) δ -112.1 – -112.3 (m), -133.6 (s) ppm; HRMS (EI) (*m/z*) $[M+H]^+$: exact mass calc. for $C_{24}H_{27}F_2N_3O_3$: 444.2099, found: 444.2098.

Fluorinated 1-cyclohexyl-2-methoxy-2-oxo-1-phenylethyl 4-fluorobenzoate (9i)

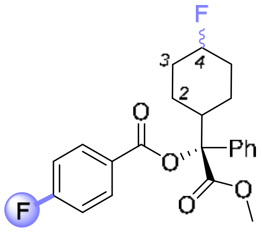

Prepared from 1-cyclohexyl-2-methoxy-2-oxo-1-phenylethyl 4-fluorobenzoate according to **General Procedure C**. Purification was conducted via column chromatography (5% EtOAc in PE) that afforded the mixture of isomers (4F:3F:2F = 4.8:4.5:1) as white solid. Yield: 116.0 mg, 53%.

IR (neat) ν (cm^{-1}): 2948, 2870, 1729, 1602, 1505, 1449, 1412, 1360, 1274, 1237, 1155, 1088, 1025, 954, 857, 767, 704; ^1H NMR (400 MHz, $CDCl_3$) δ 8.33 – 8.14 (m, 2H), 7.73 – 7.58 (m, 2H), 7.52 – 7.36 (m, 3H), 7.31 – 7.21 (m, 2H), 5.12 – 4.24 (m, 1H), 3.85 (dd, J = 7.0, 3.2 Hz, 3H), 3.02 – 2.52 (m, 1H), 2.13 (ddd, J = 20.4, 10.4, 2.8 Hz, 1H), 2.07 – 1.90 (m, 1H), 1.87 – 1.66 (m, 2H), 1.62 (dt, J = 9.1, 4.9 Hz, 1H), 1.51 – 1.28 (m, 2H), 1.29 – 0.99 (m, 1H); ^{13}C NMR (101 MHz, $CDCl_3$) δ 170.5 (s), 170.0 (d, J = 4.6 Hz), 167.3 (d, J = 1.9 Hz), 164.8 (d, J = 1.8 Hz), 164.0 (s), 164.0 (s), 163.9 (d, J = 1.2 Hz), 136.7 (t, J = 13.4 Hz), 132.4 (dd, J = 9.4, 2.8 Hz), 128.5 – 127.6 (m), 126.5 – 125.5 (m, J = 11.3, 10.5 Hz), 116.3 – 115.5 (m), 89.8 (d, J = 10.3 Hz), 88.7 (s), 88.1 (d, J = 10.6 Hz), 87.1 (s), 86.5 (d, J = 25.0 Hz), 52.4 (d, J = 1.4 Hz), 45.4 (s), 41.0 (d, J = 3.3 Hz), 32.2 (dd, J = 42.2, 20.9 Hz), 30.7 (dd, J = 21.3, 3.8 Hz), 30.3 (d, J = 21.4 Hz), 29.7 (s), 27.0 (s), 26.6 (s), 22.0 (s), 21.2 (s), 20.1 (s) ppm; ^{19}F NMR (377 MHz, $CDCl_3$) δ -104.9 (s), -105.0 (d, J = 10.9 Hz), -105.21 (s), -105.2 (d, J = 6.6 Hz), -167.7 (d, J = 61.0 Hz), -170.4 (s), -184.1 (d, J = 73.0 Hz), -185.9 (s) ppm; HRMS (ESI) (*m/z*) $[M+Na]^+$: exact mass calc. for $C_{22}H_{22}F_2O_4$: 411.1378, found: 411.1379.

2-(3-Fluorobutoxy)ethyl 4-fluorobenzoate (9a)

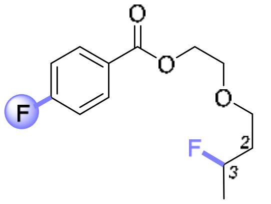

Prepared from 2-butoxyethyl 4-fluorobenzoate according to **General Procedure C**. Purification was conducted via column chromatography (5% EtOAc in PE) that afforded the mixture of two isomers in 2 : 1 (3F : 2F) ratio (based on ^{19}F NMR) as slightly yellow oil. Yield: 95.0 mg, 65%.

IR (neat) ν (cm^{-1}): 2959, 2877, 1722, 1602, 1509, 1457, 1412, 1386, 1271, 1237, 1155, 1092, 987, 894, 857, 767, 689; ^{1}H NMR (400 MHz, $CDCl_3$) δ 8.20 – 7.90 (m, 2H), 7.22 – 6.98 (m, 2H), 4.98 – 4.29 (m, 3H), 4.07 – 3.50 (m, 3H), 1.99 – 1.48 (m, 2H), 1.34 (dd, J = 24.0, 6.2 Hz, 2H), 1.04 – 0.76 (m, 2H) ppm; ^{13}C NMR (101 MHz, $CDCl_3$) δ 167.0 (s), 165.6 (s), 164.5 (s), 132.2 (d, J = 9.3 Hz), 126.3 (d, J = 3.0 Hz), 115.5 (d, J = 22.0 Hz), 94.2 (d, J = 171.1 Hz), 88.1 (d, J = 164.1 Hz), 72.8 (d, J = 22.1 Hz), 69.5 (s), 68.8 (s), 67.1 (d, J = 5.2 Hz), 64.1 (d, J = 8.7 Hz), 37.0 (d, J = 20.8 Hz), 24.6 (d, J = 21.1 Hz), 21.1 (d, J = 22.5 Hz), 9.2 (d, J = 5.8 Hz) ppm; ^{19}F NMR (377 MHz, $CDCl_3$) δ -106.1, -106.2, -175.8, -187.2 ppm; HRMS (ESI) (*m/z*) $[M+H]^+$: exact mass calc. for $C_{13}H_{16}F_2O_3$: 259.1140, found: 259.1142.

4-Fluoro-4-phenylbutyl 4-fluorobenzoate (9b)

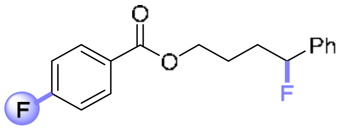

Prepared from 4-phenylbutyl 4-fluorobenzoate according to **General Procedure C**. Purification was conducted via column chromatography (1% EtOAc in PE) that afforded the product as colorless oil. Yield: 115.0 mg, 70%.

IR (neat) ν (cm^{-1}): 3063, 3030, 2955, 1714, 1602, 1505, 1453, 1408, 1267, 1237, 1151, 1110, 961, 916, 853, 764, 700; ^{1}H NMR (400 MHz, $CDCl_3$) δ 8.10 – 8.00 (m, 2H), 7.44 – 7.30 (m, 5H), 7.17 – 7.05 (m, 2H), 5.53 (ddd, J = 47.5, 8.0, 4.0 Hz, 1H), 4.46 – 4.29 (m, 2H), 2.19 – 1.86 (m, 4H) ppm; ^{13}C NMR (101 MHz, $CDCl_3$) δ 167.0 (s), 165.5 (s), 164.5 (s), 140.0 (d, J = 19.8 Hz), 132.1 (d, J = 9.3 Hz), 128.5 (s), 128.4 (d, J = 1.8 Hz), 126.5 (d, J = 3.0 Hz), 125.5 (d, J = 6.9 Hz), 115.5 (d, J = 22.0 Hz), 94.0 (d, J = 171.4 Hz), 64.6 (s), 33.8 (d, J = 24.1 Hz), 24.5 (d, J = 4.2 Hz) ppm; ^{19}F NMR (377 MHz, $CDCl_3$) δ -106.2, -176.1 ppm; HRMS (ESI) (*m/z*) $[M+H]^+$: exact mass calc. for $C_{17}H_{16}F_2O_2$: 291.1191, found: 291.1193.

Fluorinated 1-phenyldodecyl 4-fluorobenzoate (9c)

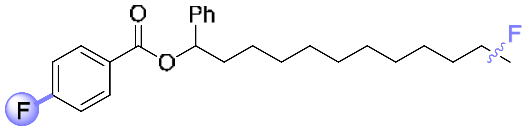

Prepared from 1-phenyldodecyl 4-fluorobenzoate according to **General Procedure C**. Purification was conducted via column chromatography (1% EtOAc in PE) that afforded the mixture of several isomers (difficult to assign fluorine positions) as white solid. Yield: 197.0 g, 87%.

IR (neat) ν (cm^{-1}): 2929, 2858, 1722, 1602, 1505, 1457, 1412, 1367, 1267, 1155, 1110, 1013, 954, 909, 853, 767, 700; ^{1}H NMR (400 MHz, $CDCl_3$) δ 8.18 – 8.03 (m, 2H), 7.34 (ddt, J = 14.2, 7.2, 4.3 Hz, 5H), 7.22 – 7.02 (m, 2H), 5.98 (t, J = 6.9 Hz, 1H), 4.79 – 4.29 (m, 1H), 2.22 – 1.85 (m, 2H), 1.59 (dddd, J = 13.2, 10.8,

10.2, 4.7 Hz, 3H), 1.50 – 1.39 (m, 3H), 1.39 – 1.25 (m, 11H), 1.00 – 0.87 (m, 2H) ppm; ^{13}C NMR (101 MHz, $CDCl_3$) δ 167.0 (s), 164.9 (s), 164.5 (s), 140.8 (s), 132.1 (d, *J* = 9.3 Hz), 129.1 – 128.4 (m), 127.9 (d, *J* = 6.6 Hz), 126.8 (s), 126.4 (s), 115.6 (s), 115.3 (s), 95.2 (dd, *J* = 18.2, 4.2 Hz), 94.8 (s), 93.7 – 93.2 (m), 91.8 (s), 90.2 (s), 37.1 (d, *J* = 15.1 Hz), 36.8 (s), 36.6 – 36.1 (m), 35.4 – 34.4 (m), 31.7 (dd, *J* = 9.6, 6.0 Hz), 30.0 – 29.0 (m), 27.3 (d, *J* = 4.5 Hz), 25.5 (d, *J* = 9.0 Hz), 25.0 (d, *J* = 4.8 Hz), 22.6 (dd, *J* = 7.9, 1.6 Hz), 21.1 (s), 20.9 (s), 18.4 (d, *J* = 4.8 Hz), 14.3 – 13.8 (m), 9.4 (d, *J* = 5.8 Hz) ppm; ^{19}F NMR (377 MHz, $CDCl_3$) δ -106.0, -106.1, -106.2, -106.3, -106.3, -106.3, -172.6, -180.5, -180.6, -180.7, -180.8, -180.9, -181.0, -181.0, -181.3, -181.4, -181.7 ppm; HRMS (ESI) (*m/z*) $[M+NH_4]^+$: exact mass calc. for $C_{25}H_{32}F_2O_2$: 420.2709, found: 420.2713.

(1*R*,2*R*,5*R*)-2-(2-Fluoropropan-2-yl)-5-methylcyclohexyl 4-fluorobenzoate (9d)

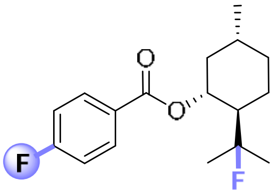

Prepared from (1*R*,2*S*,5*R*)-2-isopropyl-5-methylcyclohexyl 4-fluorobenzoate according to **General Procedure C**. Purification was conducted via column chromatography (5% EtOAc in PE) that afforded the product as colorless viscous oil. Yield: 100.0 mg, 60%.

IR (neat) ν (cm^{-1}): 2959, 2933, 2877, 1714, 1602, 1505, 1461, 1412, 1371, 1326, 1267, 1151, 1110, 1013, 987, 894, 853, 767, 685; ^{1}H NMR (400 MHz, $CDCl_3$) δ 8.25 – 8.16 (m, 2H), 7.32 – 7.19 (m, 2H), 5.21 – 5.01 (m, 1H), 4.80 (d, *J* = 48.8 Hz, 1H), 2.30 – 2.20 (m, 1H), 2.17 – 2.06 (m, 2H), 1.96 – 1.81 (m, 1H), 1.74 (dd, *J* = 23.4, 11.7 Hz, 1H), 1.53 – 1.43 (m, 1H), 1.40 (t, *J* = 6.3 Hz, 1H), 1.20 (d, *J* = 6.7 Hz, 3H), 1.07 (d, *J* = 6.9 Hz, 3H), 0.95 (d, *J* = 6.9 Hz, 3H) ppm; ^{13}C NMR (101 MHz, $CDCl_3$) δ 167.0 (s), 165.1 (s), 164.5 (s), 132.1 (d, *J* = 9.3 Hz), 126.7 (d, *J* = 3.0 Hz), 115.4 (d, *J* = 22.0 Hz), 91.7 (d, *J* = 172.4 Hz), 73.7 (s), 40.2 (s), 34.4 (d, *J* = 20.2 Hz), 34.0 (d, *J* = 1.1 Hz), 29.1 (d, *J* = 21.4 Hz), 25.9 (s), 20.4 (s), 17.2 (d, *J* = 3.5 Hz), 16.2 (s) ppm; ^{19}F NMR (377 MHz, $CDCl_3$) δ -106.4, -199.9 ppm. HRMS (ESI) (*m/z*) $[M+H]^+$: exact mass calc. for $C_{17}H_{22}F_2O_2$: 297.1661, found: 297.1658.

3-Fluoro-3-methyl-1-phenylbutyl 4-fluorobenzoate (9e)

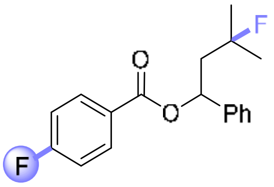

Prepared from 3-methyl-1-phenylbutyl 4-fluorobenzoate according to **General Procedure C**. Purification was conducted via column chromatography (1% EtOAc in PE) that afforded the product as colorless viscous oil. Yield: 93.0 mg, 54%.

IR (neat) ν (cm^{-1}): 2981, 2937, 1722, 1602, 1505, 1457, 1412, 1375, 1271, 1155, 1110, 1013, 857, 767, 700; ^{1}H NMR (400 MHz, $CDCl_3$) δ 8.14 – 8.02 (m, 2H), 7.47 – 7.39 (m, 2H), 7.39 – 7.27 (m, 3H), 7.16 – 7.06 (m, 2H), 6.23 (dd, J = 9.3, 3.3 Hz, 1H), 2.51 (ddd, J = 20.3, 15.1, 9.4 Hz, 1H), 2.18 (ddd, J = 18.3, 15.2, 3.3 Hz, 1H), 1.44 (dd, J = 21.5, 1.6 Hz, 6H) ppm; ^{13}C NMR (101 MHz, $CDCl_3$) δ 167.1 (s), 164.6 (s), 164.5 (s), 141.0 (s), 132.2 (d, J = 9.3 Hz), 128.6 (s), 128.1 (s), 126.5 (d, J = 2.9 Hz), 126.3 (s), 115.5 (d, J = 22.0 Hz), 95.0 (s), 93.3 (s), 73.2 (d, J = 5.2 Hz), 47.6 (d, J = 23.0 Hz), 27.5 (d, J = 24.5 Hz), 27.0 (d, J = 24.7 Hz) ppm; ^{19}F NMR (377 MHz, $CDCl_3$) δ -106.0, -136.4 ppm; HRMS (ESI) (*m/z*) $[M+NH_4]^+$: exact mass calc. for $C_{18}H_{18}F_2O_2$: 322.1613, found: 322.1616.

Fluorinated ((1*R*,2*R*,4*S*)-bicyclo[2.2.1]heptan-2-yl)methyl 4-fluorobenzoate (9f)

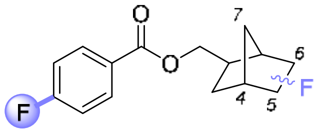

Prepared from ((1*R*,2*R*,4*S*)-bicyclo[2.2.1]heptan-2-yl)methyl 4-fluorobenzoate according to **General Procedure C**. Purification was conducted via column chromatography (5% EtOAc in PE) that afforded the mixture of several isomers (difficult to assign fluorine positions) as colorless viscous oil. Yield: 78.0 mg, 52%.

IR (neat) ν (cm^{-1}): 2963, 2877, 1714, 1602, 1505, 1453, 1412, 1349, 1267, 1155, 1110, 976, 853, 767, 685; ^{1}H NMR (400 MHz, $CDCl_3$) δ 8.10 – 7.94 (m, 2H), 7.17 – 7.01 (m, 2H), 5.05 – 4.41 (m, 1H), 4.34 – 4.12 (m, 1H), 4.11 – 3.98 (m, 1H), 2.65 – 1.94 (m, 3H), 1.79 – 1.64 (m, 2H), 1.63 – 1.42 (m, 1H), 1.41 – 1.17 (m, 2H), 1.12 – 0.47 (m, 1H) ppm; ^{13}C NMR (101 MHz, $CDCl_3$) δ 167.1 – 166.8 (m), 165.6 – 165.2 (m), 164.6 – 164.3 (m), 132.6 – 131.9 (m), 126.4 (dt, J = 6.7, 3.1 Hz), 115.7 – 115.2 (m), 96.4 – 96.0 (m), 94.5 – 94.2 (m), 93.2 (s), 91.4 (s), 67.7 (d, J = 1.3 Hz), 67.1 (d, J = 3.3 Hz), 65.9 (s), 65.6 (s), 64.3 (d, J = 4.5 Hz), 49.0 (d, J = 19.9 Hz), 44.4 (dd, J = 20.1, 6.8 Hz), 42.5 (d, J = 19.2 Hz), 42.1 (d, J = 12.4 Hz), 41.9 (s), 40.6 – 40.0 (m), 39.8 (d, J = 17.8 Hz), 39.4 (s), 39.2 (s), 37.4 (d, J = 6.8 Hz), 37.1 (d, J = 9.4 Hz), 36.0 (t, J = 10.1 Hz), 35.7 – 35.3 (m), 34.8 – 34.5 (m), 33.2 (s), 33.0 (s), 32.5 (d, J = 1.8 Hz), 31.8 (d, J = 14.7 Hz), 31.4 (s), 29.6 (s), 26.8 (d, J = 11.2 Hz), 26.4 (d, J = 10.3 Hz), 22.8 (d, J = 10.3 Hz), 21.7 (s), 14.1 (s) ppm; ^{19}F NMR (377 MHz, $CDCl_3$) δ -106.0, -106.1, -106.1, -106.2, -106.2, -158.9, -161.2, -162.5, -162.7, -168.8 ppm; HRMS (ESI) (*m/z*) $[M+H]^+$: exact mass calc. for $C_{15}H_{16}F_2O_2$: 267.1191, found: 267.1195.

7-fluoro-6,7,8,9-tetrahydro-5*H*-benzo[7]annulen-5-yl 4-fluorobenzoate (9g)

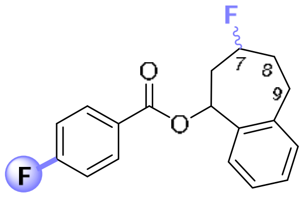

Prepared from 6,7,8,9-tetrahydro-5*H*-benzo[7]annulen-5-yl 4-fluorobenzoate according to **General Procedure C**. Purification was conducted via column chromatography (1% EtOAc in PE) that afforded the mixture of three isomers (7F : 8F : 9F) as white solid. Yield: 112.0 mg, 66%.

IR (neat) ν (cm^{-1}): 3071, 3026, 2940, 2866, 1718, 1602, 1505, 1453, 1412, 1364, 1267, 1155, 1107, 1002, 887, 853, 764, 689; ^{1}H NMR (400 MHz, $CDCl_3$) δ 8.28 – 8.05 (m, 2H), 7.57 – 7.34 (m, 1H), 7.35 – 7.09 (m, 5H), 6.59 – 6.00 (m, 1H), 5.31 – 4.55 (m, 1H), 3.70 – 2.68 (m, 2H), 2.64 – 1.85 (m, 4H) ppm; ^{13}C NMR (101 MHz, $CDCl_3$) δ 167.1 (d, *J* = 2.0 Hz), 167.1 (s), 164.6 (s), 164.6 (s), 164.6 (s), 164.5 (s), 164.5 (s), 164.4 (s), 164.2 (s), 140.2 (s), 140.1 (s), 139.2 (s), 138.9 (s), 134.0 (d, *J* = 14.6 Hz), 133.6 (d, *J* = 10.9 Hz), 132.3 (s), 132.2 (dd, *J* = 9.5, 1.4 Hz), 131.6 (s), 131.4 (s), 130.1 (s), 129.9 (s), 129.0 (s), 128.9 (s), 128.3 (s), 128.0 (s), 127.2 (s), 127.2 (s), 126.6 (s), 126.6 (s), 126.5 (d, *J* = 3.1 Hz), 126.4 (d, *J* = 3.1 Hz), 125.6 (s), 115.7 (dd, *J* = 22.0, 2.9 Hz), 91.4 (s), 90.6 (s), 89.7 (d, *J* = 3.4 Hz), 88.9 (s), 76.1 (s), 75.5 (s), 42.1 (d, *J* = 23.8 Hz), 41.4 (d, *J* = 23.3 Hz), 38.7 (d, *J* = 21.8 Hz), 34.8 (s), 33.6 (d, *J* = 20.5 Hz), 32.7 (d, *J* = 22.4 Hz), 31.8 (d, *J* = 22.3 Hz), 29.2 (d, *J* = 9.4 Hz), 28.7 (d, *J* = 9.1 Hz), 28.2 (d, *J* = 12.2 Hz) ppm; ^{19}F NMR (377 MHz, $CDCl_3$) δ -105.6, -105.7, -105.7, -163.0, -171.0, -173.5 ppm; HRMS (EI) (*m/z*) [M]$^+$: exact mass calc. for $C_{18}H_{16}F_2O_2$: 302.1118, found: 302.1115.

5-fluorocyclododecyl 4-fluorobenzoate (9h)

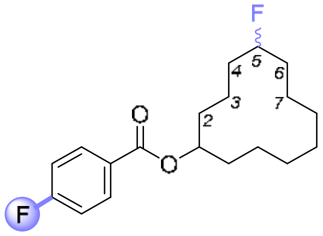

Prepared from cyclododecyl 4-fluorobenzoate according to **General Procedure C**. Purification was conducted via column chromatography (1% EtOAc in PE) that afforded the mixture of six isomers (2F:3F:4F:5F:6F:7F) as white solid. Yield: 112.0 mg, 61%.

IR (neat) ν (cm^{-1}): 2944, 2862, 1714, 1602, 1505, 1468, 1412, 1274, 1155, 1114, 991, 957, 913, 857, 767, 689; ^{1}H NMR (400 MHz, $CDCl_3$) δ 8.12 – 7.95 (m, 2H), 7.17 – 7.00 (m, 2H), 5.33 – 5.08 (m, 1H), 4.89 – 4.60 (m, 1H), 1.90 – 1.73 (m, 4H), 1.72 – 1.58 (m, 4H), 1.55 – 1.31 (m, 12H) ppm; ^{13}C NMR (101 MHz, $CDCl_3$) δ 167.0 – 166.8 (m), 165.4 – 165.0 (m), 164.5 – 164.3 (m), 132.2 – 131.8 (m), 126.9 (ddd, *J* = 8.3, 4.7, 2.0 Hz), 115.5 (d, *J* = 1.8 Hz), 115.3 (d, *J* = 1.7 Hz), 93.2 (s), 93.0 (s), 92.9 (d, *J* = 7.4 Hz), 92.5 (s), 92.2 (s), 91.3 (dd, *J* = 25.2, 13.6 Hz), 90.8 (s), 90.6 (s), 72.8 (s), 72.6 (s), 72.5 (s), 72.3 (s), 72.3 (s), 71.9 (s), 30.7 (s), 30.5 (s), 30.4 (s), 30.3 (d, *J* = 5.8 Hz), 30.2 (s), 30.1 (dd, *J* = 8.9, 3.5 Hz), 29.9 (s), 29.7 (s), 29.7 (s), 29.6 (s), 29.4 (s), 29.3 (d, *J* = 12.7 Hz), 29.1 (s), 29.0 (s), 28.8 (s), 28.5 (dd, *J* = 21.7, 5.9 Hz), 28.0 (s), 27.9 (s), 27.1 (d, *J* = 22.2 Hz), 26.2 (d, *J* = 7.2 Hz), 25.7 (t, *J* = 4.6 Hz), 24.5 (s), 24.3 (d, *J* = 15.1 Hz), 24.1 (s), 24.1 (s), 24.0 (s), 23.9 (s), 23.9 (s), 23.7 (s), 23.5 (s), 23.4 (s), 23.3 (s), 23.0 (d, *J* = 6.8 Hz), 22.0 (s), 21.7 (d, *J* = 2.4 Hz), 21.6 (s), 21.6 (s), 21.5 (s), 21.5 (s), 21.2 (d, *J* = 7.3 Hz), 20.7 (s), 20.7 (s), 20.6 (s), 20.6 (s), 20.5 (d, *J* = 6.5 Hz), 20.3 (dd, *J* = 6.9, 1.5 Hz), 19.8 (s), 19.7 (s), 19.7 (s), 19.1 (s), 19.0 (d, *J* = 7.5 Hz), 16.5

(d, J = 7.2 Hz) ppm; ^{19}F NMR (377 MHz, $CDCl_3$) δ -106.5, -106.5, -106.5, -106.6, -106.6, -106.7, -176.6, -176.7, -176.8, -177.0, -177.0, -177.4 ppm; HRMS (ESI) (m/z) $[M+H]^+$: exact mass calc. for $C_{19}H_{26}F_2O_2$: 325.1974, found: 325.1979.

Fluorinated amyl 4-fluorobenzoate (9j)

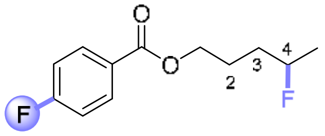

According to **General Procedure 1**. Yield: 95.0 mg, 74% (C_4:C_3:C_2=8:2:1); colorless oil; IR (neat) ν (cm^{-1}): 2978, 1718, 1603, 1510, 1454, 1413, 1271, 1238, 1156, 1111, 1014, 984, 857, 768, 690; ^{1}H NMR (400 MHz, $CDCl_3$) δ 8.27 – 8.13 (m, 2H), 7.34 – 7.18 (m, 2H), 5.01 – 4.65 (m, 1H), 4.66 – 4.36 (m, 2H), 2.28 – 1.77 (m, 4H), 1.51 (dd, J = 23.8, 6.2 Hz, 2.6H), 1.20 – 1.03 (m, 0.4H) ppm; ^{13}C NMR (101 MHz, $CDCl_3$) δ 167.0 (s), 165.6 (s), 164.5 (s), 132.1 (d, J = 9.3 Hz), 126.6 (d, J = 3.0 Hz), 115.5 (d, J = 22.0 Hz), 92.3 (d, J = 168.5 Hz), 90.4 (d, J = 165.3 Hz), 64.9 (s), 64.8 (s), 61.4 (d, J = 4.6 Hz), 34.0 (d, J = 21.3 Hz), 33.5 (d, J = 21.2 Hz), 30.0 (d, J = 19.8 Hz), 28.4 (d, J = 4.2 Hz), 28.1 (s), 24.6 (d, J = 4.6 Hz), 22.0 (d, J = 5.2 Hz), 21.0 (d, J = 22.8 Hz), 9.3 (d, J = 5.7 Hz) ppm; ^{19}F NMR (377 MHz, $CDCl_3$) δ -106.1 – -106.2 (m), -106.3 (tt, J = 8.5, 5.5 Hz), -173.6 – -174.5 (m), -184.2 – -185.1 (m) ppm; HRMS (EI) (m/z) $[M]^+$: exact mass calc. for $C_{12}H_{14}F_2O_2$: 228.0962, found: 228.0957.

4-fluoropentyl 4-fluorobenzoate (9j-1)

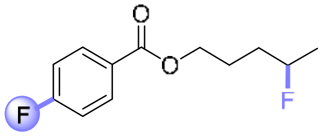

Data for major isomer depicted: IR (neat) ν (cm^{-1}): 2978, 1718, 1603, 1510, 1450, 1409, 1271, 1156, 1111, 1014, 980, 857, 768, 690; ^{1}H NMR (300 MHz, $CDCl_3$) δ 8.10 – 7.99 (m, 2H), 7.19 – 7.04 (m, 2H), 4.90 – 4.59 (m, 1H), 4.41 – 4.28 (m, 2H), 2.00 – 1.60 (m, 4H), 1.36 (dd, J = 23.9, 6.2 Hz, 3H) ppm; ^{13}C NMR (101 MHz, $CDCl_3$) δ 167.0 (s), 165.6 (s), 164.5 (s), 132.1 (d, J = 9.3 Hz), 126.6 (d, J = 3.0 Hz), 115.5 (d, J = 22.0 Hz), 90.4 (d, J = 165.3 Hz), 64.8 (s), 33.5 (d, J = 21.2 Hz), 24.6 (d, J = 4.6 Hz), 21.0 (d, J = 22.7 Hz) ppm; ^{19}F NMR (377 MHz, $CDCl_3$) δ -106.3 (tt, J = 8.4, 5.5 Hz), -173.7 – -174.3 (m) ppm; HRMS (EI) (m/z) $[M]^+$: exact mass calc. for $C_{12}H_{14}F_2O_2$: 228.0962, found: 228.0956.

Fluorinated (3*R*,5*R*,8*R*,9*S*,10*S*,13*R*,14*S*,17*R*)-17-((*R*)-5-(benzyloxy)-5-oxopentan-2-yl)-10,13-dimethylhexadecahydro-1*H*-cyclopenta[a]phenanthren-3-yl 4-fluorobenzoate (9k)

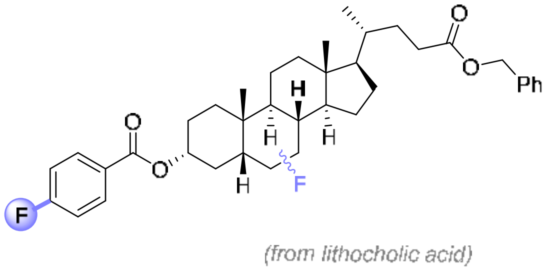

Prepared from (3*R*,5*R*,8*R*,9*S*,10*S*,13*R*,14*S*,17*R*)-17-((*R*)-5-(benzyloxy)-5-oxopentan-2-yl)-10,13-dimethylhexadecahydro-1*H*-cyclopenta[a]phenanthren-3-yl 4-fluorobenzoate according to **General Procedure C**. Purification was conducted via column chromatography (2% EtOAc in PE) that afforded the mixture of isomers (difficult to assign fluorine positions) as white solid. Yield: 68.0 mg, 20%.

IR (neat) ν (cm^{-1}): 2937, 2870, 1718, 1602, 1505, 1453, 1412, 1379, 1323, 1274, 1155, 1114, 987, 857, 767, 697; ^{1}H NMR (400 MHz, $CDCl_3$) δ 8.14 – 7.93 (m, 2H), 7.45 – 7.27 (m, 5H), 7.08 (t, *J* = 8.1 Hz, 2H), 5.20 – 5.05 (m, 2H), 5.04 – 4.83 (m, 1H), 2.49 – 2.23 (m, 2H), 2.11 – 1.90 (m, 3H), 1.91 – 1.74 (m, 5H), 1.73 – 1.48 (m, 5H), 1.48 – 0.99 (m, 14H), 0.99 – 0.88 (m, 5H), 0.65 (dd, *J* = 13.4, 4.4 Hz, 2H) ppm; ^{13}C NMR (101 MHz, $CDCl_3$) δ 173.9 (q, *J* = 3.8 Hz), 173.8 (s), 173.6 (s), 173.6 (s), 167.0 (s), 166.9 (s), 166.9 (d, *J* = 1.1 Hz), 165.0 (s), 165.0 (s), 164.9 (d, *J* = 3.8 Hz), 164.7 (s), 164.5 (s), 164.4 (s), 164.4 (d, *J* = 1.0 Hz), 164.3 (s), 138.4 (s), 136.2 (s), 136.2 (s), 136.1 (s), 132.2 (d, *J* = 9.4 Hz), 132.1 (d, *J* = 9.2 Hz), 131.9 (s), 128.5 (s), 128.3 (s), 128.2 (s), 128.1 (s), 127.4 (d, *J* = 3.0 Hz), 127.1 (d, *J* = 2.9 Hz), 127.0 (d, *J* = 2.9 Hz), 127.0 (s), 127.0 – 126.8 (m), 126.6 (d, *J* = 2.8 Hz), 122.3 (s), 115.4 (dd, *J* = 21.9, 4.9 Hz), 100.8 (s), 100.4 (s), 99.1 (s), 98.6 (s), 96.7 (s), 95.9 (s), 95.0 (s), 94.9 (s), 94.3 (s), 94.1 (s), 93.2 (s), 92.6 (s), 90.9 (s), 90.4 (s), 89.2 (s), 88.7 (s), 75.0 (s), 74.3 (d, *J* = 2.7 Hz), 74.2 (d, *J* = 2.7 Hz), 71.4 (d, *J* = 28.5 Hz), 66.1 (t, *J* = 3.8 Hz), 63.8 (d, *J* = 17.0 Hz), 61.4 (dd, *J* = 17.8, 7.7 Hz), 56.7 (s), 56.4 – 55.9 (m), 55.8 (s), 55.2 (s), 53.5 (d, *J* = 1.3 Hz), 53.4 (s), 50.3 (s), 47.5 (s), 46.5 (d, *J* = 15.0 Hz), 45.9 (d, *J* = 18.4 Hz), 44.1 (d, *J* = 5.8 Hz), 43.9 (d, *J* = 5.2 Hz), 43.5 (d, *J* = 6.6 Hz), 43.2 (s), 42.8 (d, *J* = 5.5 Hz), 42.7 (s), 42.6 (s), 42.4 (s), 42.3 (s), 42.2 (s), 42.0 (s), 41.8 (s), 41.7 (s), 41.6 (s), 41.3 (d, *J* = 1.2 Hz), 40.9 (s), 40.8 (s), 40.5 (s), 40.4 (d, *J* = 2.0 Hz), 40.3 (s), 40.2 (s), 40.1 – 39.4 (m), 38.7 (d, *J* = 9.4 Hz), 37.3 (d, *J* = 10.6 Hz), 37.1 (s), 36.9 (s), 36.6 (s), 36.2 (d, *J* = 7.5 Hz), 35.9 (s), 35.8 (s), 35.7 (s), 35.2 (dd, *J* = 5.9, 3.6 Hz), 35.1 (s), 35.1 (s), 35.0 (s), 34.9 (s), 34.8 (s), 34.7 (s), 34.6 (s), 34.6 (d, *J* = 3.8 Hz), 34.6 (s), 34.4 (s), 34.3 (s), 34.3 (s), 34.2 (s), 34.1 (d, *J* = 0.7 Hz), 34.0 (s), 33.9 (s), 33.7 (s), 33.5 (s), 33.4 (s), 33.2 (s), 33.1 (s), 32.9 (s), 32.5 (s), 32.3 (s), 32.2 (s), 31.9 (s), 31.9 (s), 31.8 (s), 31.5 (dd, *J* = 12.5, 6.7 Hz), 31.2 (d, *J* = 1.7 Hz), 31.1 (s), 31.0 (s), 30.9 (s), 30.7 (s), 30.4 (s), 30.2 (s), 30.2 (s), 29.7 (s), 29.7 (s), 29.4 (d, *J* = 10.1 Hz), 28.4 (d, *J* = 2.0 Hz), 28.1 (s), 28.1 (s), 28.0 (s), 26.9 (s), 26.8 (s), 26.7 (s), 26.6 (s), 26.6 (s), 26.4 (s), 26.3 (s), 26.3 (s), 26.0 (t, *J* = 4.5 Hz), 25.9 (s), 25.7 (d, *J* = 5.1 Hz), 24.7 (d, *J* = 6.3 Hz), 24.2 (s), 24.1 (s), 24.1 (s), 23.4 (s), 23.3 (s), 23.3 (s), 23.3 (s), 23.2 (s), 23.0 (s), 22.7 (s), 21.2 (s), 21.1 (s), 20.8 (s), 20.7 (s), 20.7 (s), 20.6 (s), 20.5 (s), 18.9 (s), 18.6 (d, *J* = 3.8 Hz), 18.4 (s), 18.3 (s), 18.3 (d, *J* = 2.0 Hz), 18.2 (s), 18.1 (s), 17.9 (s), 17.8 (s), 16.7 (s), 16.6 (s), 16.5 (s), 14.2 (s), 13.4 (s), 13.2 (s), 13.0 – 12.8 (m), 12.0 (s), 12.0 (s), 11.8 (s) ppm; ^{19}F NMR (376 MHz, $CDCl_3$) δ -106.0, -106.2, -106.3, -106.3, -106.4, -106.4, -106.5, -106.5, -106.6, -150.4, -163.4, -166.7, -171.3, -173.1, -177.0, -182.9, -185.8, -194.0 ppm; HRMS (ESI) (*m/z*) $[M+Na]^+$: exact mass calc. for $C_{25}H_{35}FO_5$: 629.3413, found: 629.3411.

4-Fluoro-*N*-((1*r*,3*s*,5*R*,7*S*)-3-fluoroadamantan-1-yl)benzamide (11d)

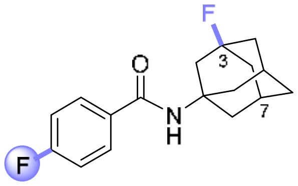

Prepared from *N*-((3*s*,5*s*,7*s*)-adamantan-1-yl)-4-fluorobenzamide according to **General Procedure C**. Purification was conducted via column chromatography (10% EtOAc in PE) that afforded the mixture of *mono*- and *difluorinated* products in 5:1 (3F:3F+7F) ratio as white solid. Yield: 115.0 mg, 70%.

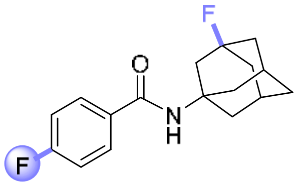

IR (neat) ν (cm^{-1}): 3309, 2918, 2862, 1714, 1643, 1602, 1539, 1498, 1457, 1356, 1312, 1230, 1159, 1110, 1017, 950, 902, 849, 767, 685; ^{1}H NMR (400 MHz, $CDCl_3$) δ 7.78 – 7.64 (m, 2H), 7.16 – 6.99 (m, 2H), 5.87 (s, 1H), 2.39 (s, 2H), 2.28 (d, *J* = 5.8 Hz, 2H), 2.05 (s, 4H), 1.97 – 1.81 (m, 4H), 1.60 (ddd, *J* = 22.7, 16.3, 7.1 Hz, 2H) ppm; ^{13}C NMR (101 MHz, $CDCl_3$) δ 165.8 (s), 165.7 (s), 163.3 (s), 131.7 (d, *J* = 3.1 Hz), 129.0 (d, *J* = 8.9 Hz), 115.5 (d, *J* = 21.8 Hz), 93.2 (s), 91.4 (s), 55.3 (d, *J* = 12.0 Hz), 46.5 (d, *J* = 18.9 Hz), 41.6 (d, *J* = 17.5 Hz), 40.1 (d, *J* = 1.6 Hz), 34.6 (d, *J* = 1.9 Hz), 31.0 (d, *J* = 10.2 Hz) ppm; ^{19}F NMR (377 MHz, $CDCl_3$) δ -109.1, -133.1 ppm; HRMS (ESI) (*m/z*) $[M+H]^+$: exact mass calc. for $C_{17}H_{19}F_2NO$: 292.1507, found: 292.1513.

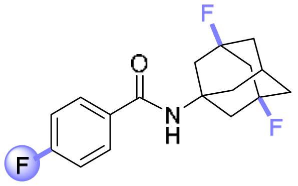

^{1}H NMR (400 MHz, $CDCl_3$) δ 7.72 (dd, *J* = 8.7, 5.3 Hz, 2H), 7.10 (t, *J* = 8.5 Hz, 2H), 5.92 (s, 1H), 2.59 – 2.47 (m, 1H), 2.42 – 2.23 (m, 4H), 2.23 – 2.17 (m, 1H), 2.10 (ddd, *J* = 10.3, 6.1, 3.9 Hz, 1H), 2.00 (s, 2H), 1.92 – 1.80 (m, 4H) ppm; ^{13}C NMR (101 MHz, $CDCl_3$) δ 165.96 (d, *J* = 12.0 Hz), 163.51 (s), 131.26 (s), 129.13 (d, *J* = 8.9 Hz), 115.79 (s), 115.57 (s), 93.23 (d, *J* = 14.5 Hz), 91.35 (d, *J* = 14.5 Hz), 55.60 (t, *J* = 13.1 Hz), 47.35 (t, *J* = 19.3 Hz), 45.45 (dt, *J* = 10.5, 5.6 Hz), 40.33 (dt, *J* = 9.9, 6.2 Hz), 38.84 (s), 29.72 (s), 29.27 (t, *J* = 11.3 Hz) ppm; ^{19}F NMR (376 MHz, $CDCl_3$) δ -108.48, -138.94 ppm; HRMS (ESI) (*m/z*) $[M+H]^+$: exact mass calc. for $C_{17}H_{18}F_3NO$: 309.1340, found: 309.1345.

4-Fluoro-*N*-(4-fluoropentyl)benzamide (11a)

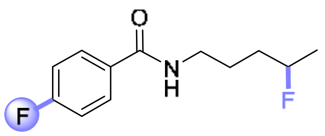

Prepared from 4-fluoro-*N*-pentylbenzamide according to **General Procedure C**. Purification was conducted via column chromatography (15% EtOAc in PE) that afforded the product as slightly yellow oil. Yield: 41.0 mg, 32%.

IR (neat) ν (cm^{-1}): 2937, 2862, 1636, 1595, 1546, 1502, 1449, 1315, 1230, 1159, 1095, 1017, 969, 849, 767, 678; ^{1}H NMR (400 MHz, $CDCl_3$) δ 7.77 (ddd, *J* = 8.8, 5.0, 2.4 Hz, 2H), 7.15 – 7.05 (m, 2H), 6.17 (s, 1H), 4.70 (dddd, *J* = 13.7, 9.3, 6.2, 3.1 Hz, 1H), 3.49 (q, *J* = 6.6 Hz, 2H), 1.85 – 1.62 (m, 4H), 1.34 (dd, *J* = 24.0, 6.2 Hz, 3H) ppm; ^{13}C NMR (101 MHz, $CDCl_3$) δ 166.5 (s), 165.9 (s), 163.4 (s), 130.8 (d, *J* = 3.2 Hz), 129.1 (d, *J* = 8.9 Hz), 115.6 (d, *J* = 21.9 Hz), 91.5 (s), 89.9 (s), 39.8 (s), 34.2 (d, *J* = 20.9 Hz), 25.4 (d, *J* = 3.9 Hz), 21.1 (s), 20.9 (s) ppm; ^{19}F NMR (377 MHz, $CDCl_3$) δ -108.9, -173.3 ppm; HRMS (EI) (*m/z*) $[M]^+$: exact mass calc. for $C_{13}H_{15}F_2NO$: 227.1122, found: 227.1116.

(4-fluoroazepan-1-yl)(4-fluorophenyl)methanone (11b)

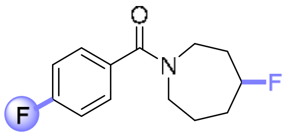

Prepared from azepan-1-yl(4-fluorophenyl)methanone according to **General Procedure C**. Purification was conducted via column chromatography (20% EtOAc in PE) that afforded the product as white solid. Yield: 36.0 mg, 27%.

IR (neat) ν (cm^{-1}): 2933, 2866, 2489, 2068, 1632, 1513, 1453, 1375, 1297, 1233, 1162, 1110, 980, 849, 764, 693; ^{1}H NMR (400 MHz, MeOD) δ 7.90 – 7.80 (m, 2H), 7.22 – 7.11 (m, 2H), 4.53 – 4.43 (m, 1H), 4.26 (dddd, *J* = 16.8, 12.3, 6.5, 4.0 Hz, 1H), 3.37 (t, *J* = 7.0 Hz, 2H), 1.83 – 1.39 (m, 6H) ppm; ^{13}C NMR (101 MHz, MeOD) δ 169.0 (s), 167.3 (s), 164.8 (s), 132.2 (d, *J* = 3.2 Hz), 130.7 (d, *J* = 8.9 Hz), 116.3 (d, *J* = 22.1 Hz), 98.8 (dd, *J* = 25.4, 6.4 Hz), 95.8 (d, *J* = 23.0 Hz), 94.1 (d, *J* = 23.8 Hz), 49.7 – 48.6 (m), 48.5 (s), 48.3 (s), 40.8 (s), 30.7 (dd, *J* = 24.5, 20.6 Hz), 30.3 (d, *J* = 1.3 Hz), 23.4 (dd, *J* = 3.3, 1.4 Hz) ppm; ^{19}F NMR (377 MHz, MeOD) δ -109.4, -194.5, -195.6 ppm; HRMS (EI) (*m/z*) $[M]^+$: exact mass calc. for $C_{13}H_{15}F_2NO$: 239.1122, found: 239.1078.

Fluorinated *N*-cycloheptyl-4-fluorobenzamide (11c)

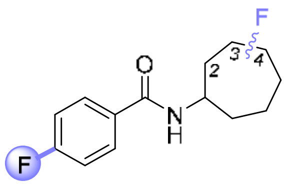

Prepared from *N*-cycloheptyl-4-fluorobenzamide according to **General Procedure C**. Purification was conducted via column chromatography (15% EtOAc in PE) that afforded the mixture of isomers (difficult to assign fluorine positions) as white solid. Yield: 43.0 mg, 30%.

IR (neat) ν (cm^{-1}): 2933, 2862, 1632, 1598, 1543, 1498, 1330, 1285, 1230, 1159, 1095, 995, 905, 849, 805, 767, 730; ^{1}H NMR (400 MHz, $CDCl_3$) δ 7.80 – 7.69 (m, 2H), 7.11 – 7.03 (m, 2H), 6.45 – 5.97 (m, 1H), 5.07 – 4.58 (m, 1H), 4.58 – 4.04 (m, 1H), 2.38 – 1.82 (m, 6H), 1.81 – 1.65 (m, 2H), 1.62 – 1.52 (m, 1H), 1.47 – 1.37 (m, 1H) ppm; ^{13}C NMR (101 MHz, $CDCl_3$) δ 165.9 (s), 165.4 (s), 165.4 (s), 163.3 (s), 130.9 (d, *J* = 3.1 Hz), 129.1 (dd, *J* = 8.9, 1.2 Hz), 115.5 (d, *J* = 21.9 Hz), 94.0 (s), 93.4 (s), 93.0 (s), 92.4 (s), 91.8 (s), 91.4 (s), 91.4 (s), 89.7 (s), 50.8 (s), 50.1 (s), 46.8 (d, *J* = 10.4 Hz), 45.6 (d, *J* = 7.4 Hz), 40.9 (d, *J* = 22.7 Hz), 40.4 (d, *J* = 21.2 Hz), 35.4 (d, *J* = 14.0 Hz), 35.1 (s), 34.9 (s), 34.5 (dd, *J* = 21.9, 7.5 Hz), 34.1 (dd, *J* = 21.6, 2.8 Hz), 30.1 (dd, *J* = 22.4, 4.2 Hz), 28.5 (d, *J* = 10.9 Hz), 28.0 (s), 27.7 (d, *J* = 5.0 Hz), 25.2 (s), 24.5 (s), 24.4 (s), 24.1 (s), 22.7 (d, *J* = 8.9 Hz), 21.9 (d, *J* = 7.9 Hz), 18.8 (d, *J* = 8.3 Hz), 18.4 (d, *J* = 6.7 Hz) ppm; ^{19}F NMR (377 MHz, $CDCl_3$) δ -108.8, -108.9, -108.9, -109.0, -109.3, -164.9, -168.5, -168.8, -169.3, -169.7 ppm; HRMS (EI) (*m/z*) $[M]^+$: exact mass calc. for $C_{14}H_{17}F_2NO$: 253.1278, found: 253.1267.

Fluorinated *N*-(cyclohexylmethyl)-4-fluorobenzamide (11e)

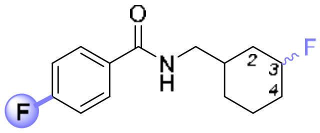

Prepared from *N*-(cyclohexylmethyl)-4-fluorobenzamide according to **General Procedure C**. Purification was conducted via column chromatography (15% EtOAc in PE) that afforded the mixture of 3 isomers (3F:4F:2F = 8:4:3) as white solid. Yield: 47.0 mg, 33%.

IR (neat) ν (cm^{-1}): 2937, 2862, 1636, 1601, 1546, 1502, 1449, 1315, 1230, 1159, 1095, 1017, 969, 849, 812, 767, 715, 678; ^{1}H NMR (400 MHz, $CDCl_3$) δ 7.77 (ddd, *J* = 8.1, 6.5, 4.9 Hz, 2H), 7.21 – 6.98 (m, 2H), 6.45 – 6.07 (m, 1H), 5.02 – 4.34 (m, 1H), 3.58 – 3.17 (m, 2H), 2.12 – 1.93 (m, 2H), 1.92 – 1.66 (m, 2H), 1.66 – 1.45 (m, 2H), 1.45 – 1.34 (m, 1H), 1.34 – 1.17 (m, 1H), 1.13 – 0.87 (m, 1H) ppm; ^{13}C NMR (101 MHz, $CDCl_3$) δ 166.6 (d, *J* = 4.2 Hz), 165.9 (s), 165.9 (s), 163.4 (s), 163.4 (s), 130.8 (dt, *J* = 5.4, 3.9 Hz), 129.1 (d, *J* = 8.8 Hz), 115.5 (dd, *J* = 21.9, 2.0 Hz), 92.9 (s), 92.6 (s), 91.2 (s), 90.9 (s), 89.7 (s), 89.4 (s), 88.0 (s), 87.8 (s), 45.8 (s), 45.6 (s), 45.5 (d, *J* = 1.4 Hz), 45.1 (d, *J* = 3.1 Hz), 37.0 (s), 36.9 – 36.8 (m), 36.7 (s), 36.2 (d, *J* = 10.1 Hz), 35.1 (d, *J* = 21.2 Hz), 32.6 (s), 32.5 (s), 31.7 (d, *J* = 18.7 Hz), 30.9 (s), 30.6 (d, *J* = 21.2 Hz), 30.1 (d, *J* = 21.2 Hz), 29.7 (s), 29.3 (d, *J* = 1.9 Hz), 27.9 (d, *J* = 11.5 Hz), 25.8 (s), 24.5 (d, *J* = 1.4 Hz), 22.5 (d, *J* = 11.4 Hz), 19.7 (d, *J* = 1.4 Hz) ppm; ^{19}F NMR (377 MHz, $CDCl_3$) δ -108.7, -108.8, -108.9, -108.9, -168.6, -170.9, -183.5, -185.4 ppm; HRMS (EI) (*m/z*) $[M]^+$: exact mass calc. for $C_{14}H_{17}F_2NO$: 253.1278, found: 253.1275.

Fluorinated 4-fluoro-*N*-(1-hydroxy-2-(hydroxymethyl)-4-(4-octylphenyl)butan-2-yl)benzamide (11f)

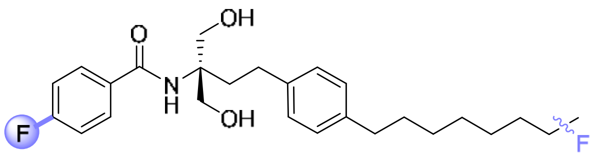

Prepared from 4-fluoro-*N*-(1-hydroxy-2-(hydroxymethyl)-4-(4-octylphenyl)butan-2-yl)benzamide according to the **General Procedure C** to give **11f** (38% NMR yield, (trifluoromethyl)benzene as IS). ^{19}F NMR (376 MHz, CD_3CN) δ -165.3, -165.8, -166.8, -170.1 ppm; HRMS (ESI) (*m/z*) $[M+H]^+$: exact mass calc. for $C_{26}H_{36}FNO_3$: 448.2658, found: 448.2660.

Fluorinated Haloperidol (13)

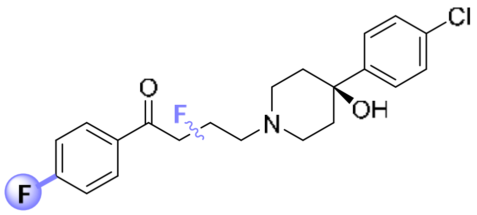

Prepared from Haloperidol according to the **General Procedure C** to give **13** (43% NMR yield, (trifluoromethyl)benzene as IS). ^{19}F NMR (377 MHz, $CDCl_3$) δ -104.9, -105.1, -125.8, -125.9, -127.1, -127.3 ppm. As a proof of concept experiment, the compound was not isolated.

((4b*R*,9*S*)-8a-fluoro-3-methoxy-6,7,8,8a,9,10-hexahydro-5*H*-9,4b-(epiminoethano)phenanthren-11-yl)(4-fluorophenyl)methanone (11h)

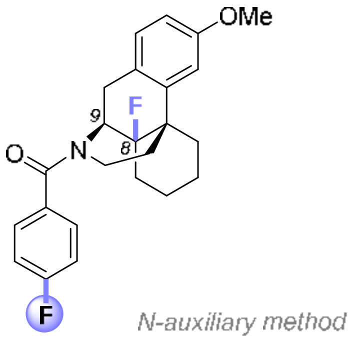

Prepared from (4-fluorophenyl)((4b*S*,9*S*)-3-methoxy-6,7,8,8a,9,10-hexahydro-5*H*-9,4b-(epiminoethano)-phenanthren-11-yl)methanone according to **General Procedure C**. Purification was conducted via column chromatography (20% EtOAc in PE) that afforded a mixture of two isomers of the product in 3 : 1 (8F : 9F) ratio as white solid. Yield: 85.0 mg, 38%.

IR (neat) ν (cm^{-1}): 2929, 2855, 1714, 1628, 1490, 1423, 1360, 1330, 1282, 1222, 1159, 1107, 1013, 931, 849, 797, 760, 708; ^{1}H NMR (400 MHz, $CDCl_3$) δ 7.50 – 7.29 (m, 7H), 7.07 (dt, *J* = 24.1, 8.3 Hz, 7H), 6.86 (dd, *J* = 19.4, 8.5 Hz, 7H), 4.93 (s, 2H), 4.48 (d, *J* = 10.5 Hz, 1H), 3.85 (s, 11H), 3.45 (d, *J* = 10.3 Hz, 2H),

3.27 (dd, J = 18.4, 6.3 Hz, 2H), 3.03 (dd, J = 18.0, 5.8 Hz, 1H), 2.99 – 2.82 (m, 5H), 2.81 – 2.55 (m, 5H), 1.79 – 1.51 (m, 18H), 1.48 – 1.12 (m, 18H) ppm; ^{13}C NMR (101 MHz, $CDCl_3$) δ 169.4 (d, J = 1.7 Hz), 169.4 (s), 164.4 (s), 161.9 (s), 152.8 (s), 152.1 (s), 150.3 (s), 149.6 (s), 146.6 (s), 146.5 (s), 146.4 (s), 134.6 (d, J = 3.6 Hz), 132.8 (s), 132.5 (d, J = 2.4 Hz), 130.0 (d, J = 4.5 Hz), 129.2 (s), 129.0 (d, J = 8.4 Hz), 128.7 (d, J = 8.3 Hz), 126.8 (d, J = 9.8 Hz), 123.2 (d, J = 3.4 Hz), 123.1 (d, J = 2.8 Hz), 115.8 (s), 115.6 (s), 115.3 (s), 111.3 (s), 110.9 (s), 56.6 (d, J = 4.7 Hz), 56.2 (s), 55.2 (s), 53.9 (s), 47.6 (s), 45.9 (s), 45.0 (s), 43.8 (s), 42.6 (s), 42.4 (d, J = 1.3 Hz), 42.1 (s), 39.5 (s), 38.7 (s), 38.6 (s), 38.5 (s), 37.7 (d, J = 11.3 Hz), 37.2 (d, J = 13.7 Hz), 36.5 (s), 31.8 (s), 31.4 (s), 31.4 (s), 31.1 (s), 30.1 (s), 29.7 (s), 26.7 (s), 26.3 (s), 26.2 (s), 22.8 (s), 22.7 (s), 22.0 – 21.7 (m) ppm; ^{19}F NMR (376 MHz, $CDCl_3$) δ -111.2, -111.3, -111.3, -134.8, -134.9, -138.9, -139.0 ppm; HRMS (EI) (*m/z*) $[M]^+$: exact mass calc. for $C_{24}H_{25}F_2NO_2$: 397.1853, found: 397.1845.

Fluorinated (4b*S*,9*S*)-11-methyl-6,7,8,8a,9,10-hexahydro-5*H*-9,4b-(epiminoethano)-phenanthren-3-yl 4-fluorobenzoate (9l)

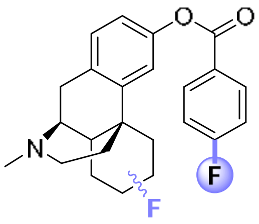

O-auxiliary method

Prepared from (4b*S*,9*S*)-11-methyl-6,7,8,8a,9,10-hexahydro-5*H*-9,4b-(epiminoethano)phenanthren-3-yl 4-fluorobenzoate according to the **General Procedure A** to give **9k** (42% NMR yield, (trifluoromethyl)benzene as IS). ^{19}F NMR (376 MHz, $CDCl_3$) δ -104.8, -129.6 ppm. As a proof of concept experiment, the compound was not isolated.

5-Fluorocyclododecan-1-ol (14h)

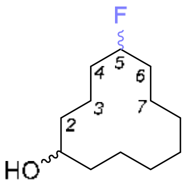

Prepared from 5-fluorocyclododecyl 4-fluorobenzoate according to **General Procedure D**. Purification was conducted via column chromatography (10% EtOAc in PE) that afforded the product as white solid. Yield: 167.0 mg, 96%.

IR (neat) ν (cm^{-1}): 2937, 2858, 1468, 1364, 1125, 1080, 1043, 1006, 946, 909, 730; ^{1}H NMR (400 MHz, $CDCl_3$) δ 4.79 – 4.55 (m, 1H), 3.85 – 3.73 (m, 1H), 1.83 – 1.70 (m, 2H), 1.69 – 1.50 (m, 6H), 1.49 – 1.27 (m, 13H) ppm; ^{13}C NMR (101 MHz, $CDCl_3$) δ 94.2 (d, J = 8.5 Hz), 93.3 (s), 93.0 (s), 92.9 (s), 92.7 (s), 92.6 (s), 92.5 (s), 92.5 (s), 91.7 (s), 91.1 (dd, J = 31.5, 14.0 Hz), 70.6 (d, J = 11.6 Hz), 69.2 (s), 68.9 (s), 68.8 (s), 68.7 (s), 68.4 (s), 68.2 (s), 33.0 (s), 32.9 (s), 32.8 (s), 32.7 (s), 32.7 (s), 32.3 (s), 32.3 (s), 32.2 (s), 31.2 (s),

31.0 (s), 30.8 (s), 30.8 (s), 30.8 (s), 30.6 (s), 30.5 (s), 30.4 (d, J = 3.4 Hz), 30.3 (s), 30.2 (s), 30.2 (s), 30.0 (s), 29.9 (s), 29.8 (s), 29.8 (s), 29.7 (s), 29.7 (s), 29.5 (d, J = 6.6 Hz), 28.8 (d, J = 10.1 Hz), 28.6 (s), 28.5 (d, J = 10.3 Hz), 28.4 (s), 28.4 (s), 27.6 (d, J = 21.8 Hz), 26.6 (d, J = 6.9 Hz), 25.8 (s), 25.7 (s), 25.1 (d, J = 21.5 Hz), 24.4 (d, J = 1.2 Hz), 24.2 (s), 24.1 (s), 23.9 (d, J = 11.0 Hz), 23.6 (s), 23.5 (s), 23.5 (d, J = 1.5 Hz), 23.1 (d, J = 4.8 Hz), 22.2 (s), 22.2 (s), 22.0 (s), 21.8 (s), 21.7 (s), 21.7 (s), 21.5 (d, J = 7.7 Hz), 21.2 (d, J = 6.4 Hz), 20.9 (s), 20.8 (s), 20.7 (s), 20.6 – 20.4 (m), 20.2 (d, J = 6.3 Hz), 19.9 (d, J = 7.3 Hz), 19.3 (d, J = 2.1 Hz), 19.2 – 18.8 (m), 18.7 (d, J = 7.5 Hz), 16.2 (d, J = 7.9 Hz) ppm; ^{19}F NMR (377 MHz, $CDCl_3$) δ -176.1, -176.4, -176.6, -176.7, -176.7, -176.9, -177.0, -177.1 ppm; HRMS (ESI) (*m/z*) $[M+NH_4]^+$: exact mass calc. for $C_{12}H_{23}FO$: 220.2071, found: 220.2071.

(1*S*,2*S*,5*S*)-2-(2-Fluoropropan-2-yl)-5-methylcyclohexan-1-ol (14d)

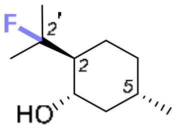

Prepared from (1*R*,2*R*,5*R*)-2-(2-fluoropropan-2-yl)-5-methylcyclohexyl 4-fluorobenzoate according to **General Procedure D**. Purification was conducted via column chromatography (10% EtOAc in PE) that afforded the product as white solid. Yield: 166.0 mg, 95%.

IR (neat) ν (cm^{-1}): 2959, 2937, 2873, 1464, 1367, 1271, 1215, 1181, 1133, 1073, 1032, 969, 887, 827, 790, 738, 667; ^{1}H NMR (400 MHz, $CDCl_3$) δ 4.40 (ddd, J = 53.3, 48.5, 3.5 Hz, 2H), 4.04 – 3.35 (m, 3H), 2.28 – 1.81 (m, 7H), 1.80 – 1.73 (m, 6H), 1.66 – 1.39 (m, 7H), 1.39 – 1.08 (m, 10H), 1.04 – 0.74 (m, 28H) ppm; ^{13}C NMR (101 MHz, $CDCl_3$) δ 98.0 (s), 96.5 (s), 96.3 (s), 94.9 (s), 92.9 (s), 91.2 (s), 71.0 (d, J = 0.9 Hz), 70.6 (s), 70.1 (d, J = 1.8 Hz), 69.1 (d, J = 10.3 Hz), 68.1 (s), 60.4 (s), 50.2 (s), 49.3 (s), 49.2 (s), 47.8 (d, J = 10.2 Hz), 46.0 (d, J = 21.8 Hz), 43.1 (s), 41.5 (d, J = 9.8 Hz), 38.1 (d, J = 1.1 Hz), 36.5 (d, J = 18.6 Hz), 36.2 (d, J = 22.5 Hz), 35.6 (d, J = 21.1 Hz), 34.6 (d, J = 20.3 Hz), 34.1 (s), 29.3 (d, J = 19.2 Hz), 28.6 (d, J = 21.3 Hz), 27.5 (d, J = 24.5 Hz), 25.8 (s), 25.7 (s), 25.4 (s), 25.2 (s), 22.5 (s), 22.3 (s), 20.9 (t, J = 7.5 Hz), 20.6 (s), 19.3 (d, J = 9.8 Hz), 18.7 (d, J = 1.4 Hz), 17.6 (d, J = 1.6 Hz), 17.3 (d, J = 3.6 Hz), 17.2 (s), 16.0 (s), 15.8 (s), 14.1 (s), 14.0 (s) ppm; ^{19}F NMR (377 MHz, $CDCl_3$) δ -149.5, -178.4, -199.6 ppm; HRMS (ESI) (*m/z*) $[M+NH_4]^+$: exact mass calc. for $C_{10}H_{19}FO$: 292.1758, found: 292.1757.

(1*r*,3*s*,5*R*,7*S*)-3-Fluoroadamantan-1-amine (15c)

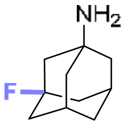

Prepared from 4-Fluoro-*N*-((1*r*,3*s*,5*R*,7*S*)-3-fluoroadamantan-1-yl)benzamide according to **General Procedure E.** Purification was conducted via column chromatography (5% MeOH in DCM) that afforded the product as slightly yellow solid. Yield: 17.0 mg, 98%.

IR (neat) ν (cm^{-1}): 2929, 2862, 2668, 2571, 1591, 1502, 1457, 1356, 1118, 1010, 939, 898, 834, 730; ^{1}H NMR (400 MHz, MeOD) δ 2.48 – 2.39 (m, *J* = 2.5 Hz, 2H), 1.99 (d, *J* = 5.4 Hz, 2H), 1.95 – 1.84 (m, 4H), 1.81 (s, 4H), 1.68 – 1.56 (m, 2H) ppm; ^{13}C NMR (101 MHz, MeOD) δ 93.1 (s), 91.2 (s), 55.2 (d, *J* = 11.8 Hz), 49.7 – 48.9 (m), 48.7 (s), 48.5 (s), 48.3 (s), 46.6 (d, *J* = 21.1 Hz), 41.9 (d, *J* = 17.9 Hz), 40.1 (d, *J* = 1.3 Hz), 34.7 (d, *J* = 1.9 Hz), 32.0 (d, *J* = 10.0 Hz) ppm; ^{19}F NMR (377 MHz, MeOD) δ -133.9 ppm; HRMS (EI) (*m/z*) $[M]^+$: exact mass calc. for $C_{10}H_{16}FN$: 169.1267, found: 169.1264.

(4b*R*,8a*R*,9*S*)-8a-fluoro-3-methoxy-6,7,8,8a,9,10-hexahydro-5*H*-9,4b-(epiminoethano)phenanthrene (15f)

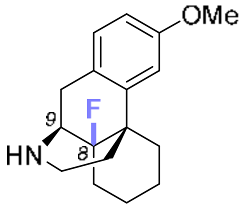

Prepared from ((4b*R*,9*S*)-8a-fluoro-3-methoxy-6,7,8,8a,9,10-hexahydro-5*H*-9,4b-(epiminoethano) phenanthrene-11-yl)(4-fluorophenyl)methanone according to the **General Procedure E**. Purification was conducted via column chromatography (5% MeOH in DCM) that afforded the product as slightly yellow solid. Yield: 26.0 mg, 93%.

IR (neat) ν (cm^{-1}): 3414, 2926, 2855, 2769, 2676, 2467, 1741, 1655, 1618, 1573, 1491, 1442, 1274, 1267, 1245, 1204, 1159, 1089, 1051, 958, 854, 816, 757, 701; ^{1}H NMR (400 MHz, $CDCl_3$) δ 9.11 (brs, 1H), 6.99 – 6.71 (m, 3H), 3.86 (d, *J* = 1.9 Hz, 3H), 3.79 (dd, *J* = 9.8, 4.6 Hz, 2H), 3.33 – 3.23 (m, 2H), 2.91 (d, *J* = 13.2 Hz, 1H), 2.73 (dd, *J* = 13.2, 9.9 Hz, 1H), 2.39 – 2.19 (m, 1H), 2.14 (d, *J* = 12.4 Hz, 1H), 2.10 – 1.95 (m, 2H), 1.82 (d, *J* = 12.3 Hz, 1H), 1.70 – 1.55 (m, 4H) ppm; ^{13}C NMR (101 MHz, $CDCl_3$) δ 160.4, 159.2, 152.5, 152.3, 150.0, 149.8, 147.1, 147.0, 147.0, 146.9, 138.9, 138.9, 138.7, 133.4, 133.4, 131.3, 129.6, 129.4, 128.5, 128.1, 127.2, 127.2, 126.7, 126.6, 125.6, 125.5, 123.7, 123.6, 115.6, 115.5, 112.8, 112.4, 111.9, 110.8, 110.7, 110.6, 58.8, 56.6, 56.2, 55.3, 55.2, 52.2, 51.1, 41.9, 40.6, 40.3, 40.1, 37.9, 37.9, 37.7, 37.5, 37.5, 37.2, 37.0, 36.8, 36.3, 36.2, 35.6, 35.5, 29.6, 27.9, 27.7, 25.7, 25.6, 25.5, 25.3, 22.4, 21.4, 14.0, 0.9 ppm; ^{19}F NMR (376 MHz, $CDCl_3$) δ -134.5, -137.5 ppm; HRMS (EI) (*m/z*) $[M]^+$: exact mass calc. for $C_{17}H_{22}FNO$: 275.1685, found: 275.1675.

Fluorinated cyclohexylmethanamine (15b)

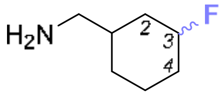

Prepared from fluorinated *N*-(cyclohexylmethyl)-4-fluorobenzamide according to the **General Procedure E**. Purification was conducted via column chromatography (5% MeOH in DCM) that afforded the mixture of 3 isomers (3F:4F:2F = 8:4:3) as slightly yellow solid. Yield: 25.0 mg, 95%.

IR (neat) ν (cm^{-1}): 3422, 2933, 2676, 2490, 2207, 2054, 1610, 1510, 1461, 1394, 1219, 1163, 1103, 1033, 958, 835, 809, 731, 686; ^{1}H NMR (400 MHz, $CDCl_3$) δ 7.62 (brs, 2H), 5.08 – 4.31 (m, 2H), 3.00 – 2.85 (m, 1H), 2.20 (dd, *J* = 16.7, 7.5 Hz, 1H), 1.93 (tdd, *J* = 26.2, 18.6, 10.9 Hz, 2H), 1.77 – 1.19 (m, 4H), 1.09 (ddd, *J* = 25.0, 14.7, 9.8 Hz, 1H), 0.92 – 0.77 (m, 1H) ppm; ^{13}C NMR (101 MHz, $CDCl_3$) δ 91.7, 90.0, 89.0, 88.7, 87.3, 87.0, 45.7, 45.3, 45.2, 44.8, 44.7, 36.4, 36.2, 35.6, 34.7, 34.6, 34.5, 34.3, 34.0, 33.9, 32.2, 32.0, 31.8, 31.3, 31.1, 30.3, 30.4, 30.2, 29.9, 29.7, 29.6, 29.5, 29.2, 28.8, 28.8, 27.6, 27.5, 25.8, 25.2, 24.2, 22.6, 21.9, 21.8, 20.9, 19.3, 14.1, 14.1, 14.0, 13.9, 10.9, 10.9, 10.9, 0.9 ppm; ^{19}F NMR (376 MHz, $CDCl_3$) δ -169.3, -171.5, -183.7, -185.8 ppm; HRMS (EI) (*m/z*) $[M]^+$: exact mass calc. for $C_7H_{14}FN$: 131.1110, found: 131.1108.

***N*-cinnamyl-4-fluorobenzamide (22)**

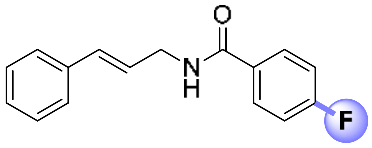

Prepared from cinnamyl amine and 4-fluorobenzoyl chloride by **General Procedure 3**. Yellow solid (250.0 mg, 91%). Data: IR (neat) ν (cm^{-1}): 3295, 3064, 3027, 2914, 1633, 1620, 1543, 1498, 1361, 1312, 1290, 1230, 1159, 1096, 973, 962, 850, 841, 747, 742, 690; ^{1}H NMR (400 MHz, $CDCl_3$) δ 7.82 – 7.73 (m, 2H), 7.34 – 7.14 (m, 5H), 7.08 – 6.95 (m, 2H), 6.65 (s, 1H), 6.49 (d, *J* = 15.9 Hz, 1H), 6.24 – 6.12 (m, 1H), 4.13 (dd, *J* = 8.3, 3.4 Hz, 2H) ppm; ^{13}C NMR (101 MHz, $CDCl_3$) δ 166.4 (s), 166.0 (s), 163.5 (s), 136.5 (s), 132.5 (s), 130.6 (d, *J* = 3.1 Hz), 129.4 (d, *J* = 8.9 Hz), 128.6 (s), 127.8 (s), 126.4 (s), 125.3 (s), 115.6 (d, *J* = 21.9 Hz), 42.2 (s) ppm; ^{19}F NMR (377 MHz, $CDCl_3$) δ -108.6 – -108.7 (m) ppm; HRMS (EI) (*m/z*) $[M]^+$: exact mass calc. for $C_{16}H_{14}FNO$: 255.1059, found: 255.1062.

***N,N'*-((3,4-diphenylcyclobutane-1,2-diyl)bis(methylene))bis(4-fluorobenzamide) (23)**

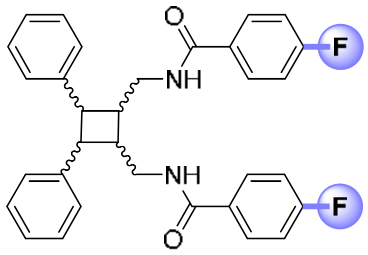

Prepared from *N*-cinnamyl-4-fluorobenzamide (**22**). After charging **22** (0.05 mmol) to a vial and dissolving in MeCN (1 mL for 0.05 M) under air, the reaction was *not* degassed and was irradiated with 400 nm LEDs for 72 h. 6× reaction vials were combined. Following evaporation of solvent *in vacuo*, purification was conducted via column chromatography (50% EtOAc in PE) that afforded **23** a colorless oil (24.0 mg, 31%), the single head-to-head regioisomer as a mixture of two diastereomers (*all-trans* **23-1**: *all-cis* **23-2** = 5 : 1):

***N,N'*-(((1*R*,2*R*,3*S*,4*S*)-3,4-diphenylcyclobutane-1,2-diyl)bis(methylene))bis(4-fluorobenzamide) (23-1)**

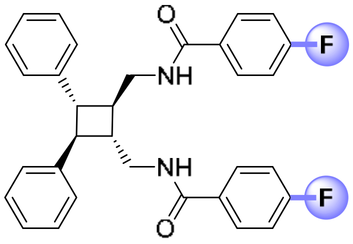

IR (neat) ν (cm^{-1}): 3310, 3071, 2926, 2859, 1726, 1655, 1603, 1543, 1510, 1454, 1413, 1342, 1260, 1234, 1156, 1129, 1088, 980, 913, 850, 820, 760, 701, 678; ^{1}H NMR (400 MHz, $CDCl_3$) δ 8.04 – 7.96 (m, 2H), 7.40 (ddd, *J* = 9.0, 5.9, 1.9 Hz, 5H), 7.09 – 7.02 (m, 2H), 5.12 (d, *J* = 6.6 Hz, 1H), 4.01 (td, *J* = 6.7, 4.6 Hz, 1H), 3.74 (dd, *J* = 16.7, 4.6 Hz, 1H), 3.57 (dd, *J* = 16.8, 6.7 Hz, 1H), 2.54 (s, 1H) ppm; ^{13}C NMR (101 MHz, $CDCl_3$) δ 137.5 (s), 129.6 (d, *J* = 8.8 Hz), 128.9 (s), 128.8 (s), 126.3 (s), 115.2 (d, *J* = 21.8 Hz), 81.0 (s), 66.6 (s), 48.1 (s) ppm; ^{19}F NMR (377 MHz, $CDCl_3$) δ -109.8 – -109.9 (m) ppm; HRMS (EI) (*m/z*) $[M]^+$: exact mass calc. for $C_{32}H_{28}F_2N_2O_2$: 510.2119, found: 510.2121 (peak #1).

***N,N'*-(((1*R*,2*S*,3*R*,4*S*)-3,4-diphenylcyclobutane-1,2-diyl)bis(methylene))bis(4-fluorobenzamide) (23-2)**

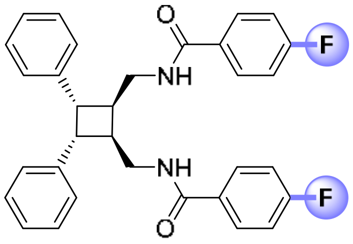

IR (neat) ν (cm^{-1}): 3310, 3071, 2926, 2859, 1726, 1655, 1603, 1543, 1510, 1454, 1413, 1342, 1260, 1234, 1156, 1129, 1088, 980, 913, 850, 820, 760, 701, 678; ^{1}H NMR (300 MHz, $CDCl_3$) δ 7.94 – 7.87 (m, 4H), 7.85 – 7.79 (m, 4H), 7.46 – 7.31 (overlaps with the other diastereomer, m, 5H), 7.18 – 6.98 (overlaps with the other diastereomer, m, 5H), 5.04 (d, *J* = 4.4 Hz, 2H), 4.95 – 4.81 (m, 3H), 4.14 – 4.06 (m, 1H), 3.87 (dd, *J* = 15.0, 9.9 Hz, 2H), 2.62 (brs, 2H) ppm; ^{13}C NMR (101 MHz, $CDCl_3$) δ 165.9 (s), 163.4 (s), 154.8 (s), 139.2 (s), 130.4 (d, *J* = 8.9 Hz), 129.8 (d, *J* = 9.1 Hz), 128.7 (d, *J* = 2.8 Hz), 128.7 (s), 128.2 (s), 126.3 (s), 115.8 (s), 115.6 (d, *J* = 1.5 Hz), 115.4 (s), 82.9 (s), 73.7 (s), 55.0 (s) ppm; ^{19}F NMR (377 MHz, $CDCl_3$) δ -107.6 – -107.9 (m), -108.4 – -108.7 (m) ppm; HRMS (EI) (*m/z*) $[M]^+$: exact mass calc. for $C_{32}H_{28}F_2N_2O_2$: 510.2119, found: 510.2122 (peak #2).

Cinnamyl 4-fluorobenzoate (24)

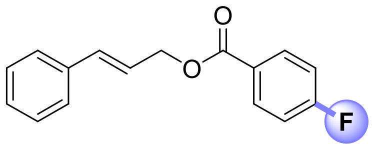

Prepared from cinnamyl alcohol and 4-fluorobenzoyl chloride by **General Procedure 1.** Colorless viscous oil (1.32 g, 94%). Data: IR (neat) ν (cm^{-1}): 3070, 3030, 2890, 2820, 1701, 1650, 1513, 1403, 1380, 1261, 1221, 1114, 1011, 932, 825, 768, 701; ^{1}H NMR (400 MHz, $CDCl_3$) δ 8.35 – 8.16 (m, 2H), 7.62 – 7.54 (m, 2H), 7.53 – 7.32 (m, 3H), 7.32 – 7.17 (m, 2H), 6.89 (d, *J* = 15.9 Hz, 1H), 6.56 (dt, *J* = 15.9, 6.4 Hz, 1H), 5.13 (dd, *J* = 6.4, 1.3 Hz, 2H) ppm; ^{13}C NMR (101 MHz, $CDCl_3$) δ 167.1 (s), 165.4 (s), 164.6 (s), 136.2 (s), 134.5 (s), 132.3 (d, *J* = 9.3 Hz), 128.7 (s), 128.5 (d, *J* = 7.5 Hz), 128.2 (s), 126.7 (s), 126.5 (d, *J* = 3.0 Hz), 123.1 (s), 115.6 (d, *J* = 21.9 Hz), 65.7 (s) ppm; ^{19}F NMR (377 MHz, $CDCl_3$) δ -106.1 (dq, *J* = 8.4, 5.5 Hz) ppm; HRMS (EI) (*m/z*) $[M]^+$: exact mass calc. for $C_{16}H_{13}FO_2$: 256.0900, found: 256.0903.

(3,4-Diphenylcyclobutane-1,2-diyl)bis(methylene) bis(4-fluorobenzoate) (25)

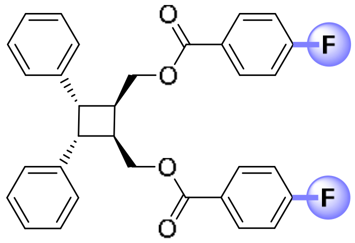

Prepared from cinnamyl-4-fluorobenzoate (**24**). After charging **24** (0.05 mmol) to a vial and dissolving in MeCN (1 mL for 0.05 M) under air, the reaction was *not* degassed and was irradiated with 400 nm LEDs for 72 h. 6× reaction vials were combined. Following evaporation of solvent *in vacuo*, purification was conducted via column chromatography (5% EtOAc in PE) that afforded **25** as a colorless oil, (67.0 mg, 87%) as a single head-to-head regioisomer and single diastereomer:

IR (neat) ν (cm^{-1}): 3320, 3070, 2920, 2810, 1711, 1641, 1598, 1542, 1503, 1441, 1398, 1319, 1220, 1054, 974, 853, 827, 778, 702; ^{1}H NMR (400 MHz, $CDCl_3$) δ 8.07 – 7.94 (m, 2H), 7.31 – 7.18 (m, 5H), 7.10 – 6.98 (m, 2H), 4.65 (dd, *J* = 12.3, 3.3 Hz, 1H), 4.25 (dd, *J* = 12.3, 5.9 Hz, 1H), 3.80 (d, *J* = 1.9 Hz, 1H), 3.31 (ddd, *J* = 5.5, 3.2, 2.1 Hz, 1H) ppm; ^{13}C NMR (101 MHz, $CDCl_3$) δ 167.2 (s), 165.3 (s), 164.7 (s), 136.2 (s), 132.4 (d, *J* = 9.3 Hz), 128.6 (s), 128.5 (s), 125.9 (d, *J* = 3.0 Hz), 125.7 (s), 115.7 (d, *J* = 22.0 Hz), 64.9 (s), 59.4 (s), 56.5 (s) ppm; ^{19}F NMR (377 MHz, $CDCl_3$) δ -105.6 (tt, *J* = 8.4, 5.5 Hz) ppm; HRMS (EI) (*m/z*) $[M]^+$: exact mass calc. for $C_{32}H_{26}F_2O_4$: 512.1799, found: 512.1801.

3 Mechanistic Studies

Considering that triplet state energies of methyl benzoate (77.9 kcal mol^{-1} [32]), benzonitrile (77.0 kcal mol^{-1} [32]), and benzamide (79.4 kcal mol^{-1} [33]) are all higher than the energy gap between singlet and triplet states of **SF** (61.4 kcal mol^{-1} [28a]), TTET from the **PSCats** to **SF** by formation of **PSCat-SF** exciplex should be feasible in each case. However, methyl benzoate, 4-*n*-butyl benzonitrile, and 4-*n*-butyl benzamide could not be used as an exogenous **PSCat** (entries 1,9,10, Table S5). Presumably, either i) they form a "weak" exciplex, that is too short-lived to approach the substance to undergo downstream HAT and FAT processes, or ii) they form an exciplex a concentration too small to give productive downstream chemistry. Unlike amyl benzoate, 4-*n*-butyl benzonitrile and 4-*n*-butyl benzamide did not undergo self-fluorination at their *n*-butyl chains. Yet, in the presence of catalytic **MFB**, both underwent fluorination at their alkyl chains (Scheme S1), which confirms the necessity of the benzoate moiety in corresponding fluorination reactions.

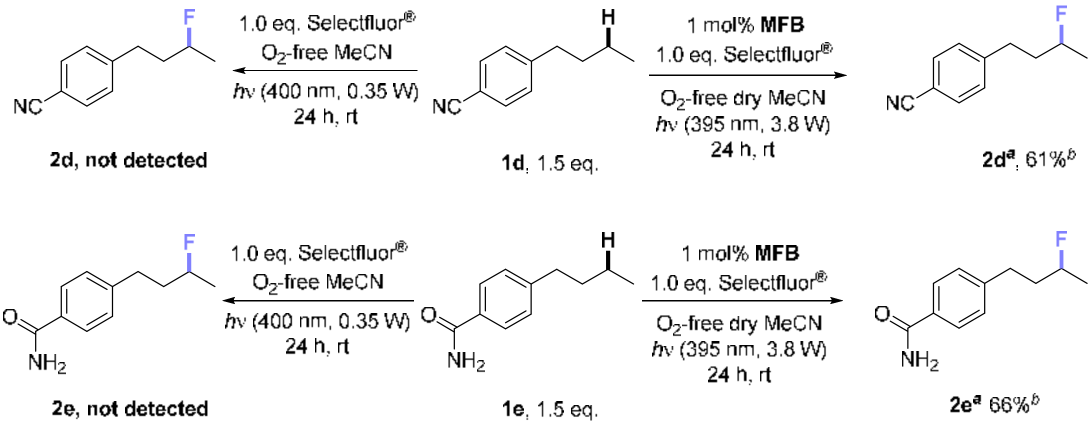

Scheme S1. Control experiments on fluorination of benzonitrile and benzamide. ^{a} only the major isomer of the product is depicted, ^{b} overall NMR yield.

The reason that amyl benzoate did undergo self-fluorination but that methyl benzoate cannot be used as an exogenous **PSCat**, is likely that they both form a "weak" exciplex, however, in the case of amyl benzoate, the exciplex does not need to diffuse to a molecule of substrate, but rather can undergo rapid, intramolecular self-fluorination at its alkyl chain. Although the triplet energy of **MFB** is not known, we assume that the *p*-fluorine substituent of **MFB** assisted in the formation of longer-lived exciplex, or has a triplet energy that is much closer matched to that of **SF**, which resulted in its utilization as an efficient exogenous **PSCat**.

3.1 Radical Trapping

To investigate the intermediacy of radicals in the reaction, a radical trapping experiment with 1.5 eq. of TEMPO was performed. As clear evidence of the alkyl chain radical intermediate, LC-MS data (Figure S5) detected a product which matched the TEMPO-bound **16** and no fluorination products was detected (Scheme S2). As has been reported in the literature,[34] the radical dication of **SF** can act as a hydrogen atom transfer (HAT) reagent. Presumably, the alkyl radical intermediate forms via HAT between the radical dication of **SF** and the substrate (hexyl propionate, **1k**). Thus, the selectivity of fluorination reactions depends on the bond dissociation enthalpy (BDE) and the hydricity (electron richness) of C(sp^3)–H positions

within the substrate as per previous reports.[28a] Selectivity favors the C(sp^3)–H with the lowest BDE and highest hydricity (electron richness).

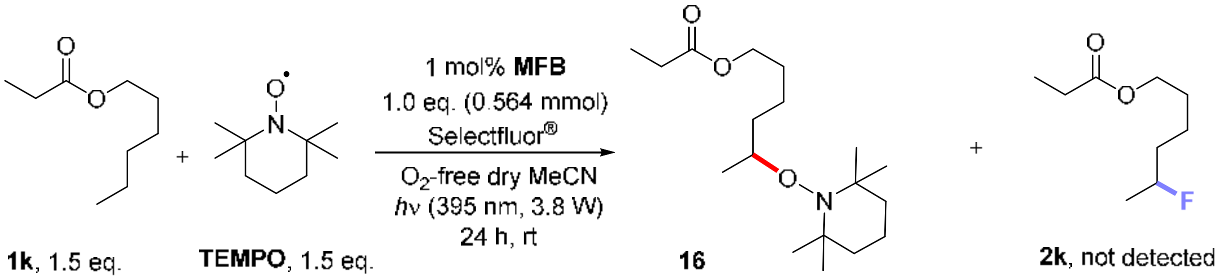

Scheme S2. Radical trapping reaction with TEMPO.

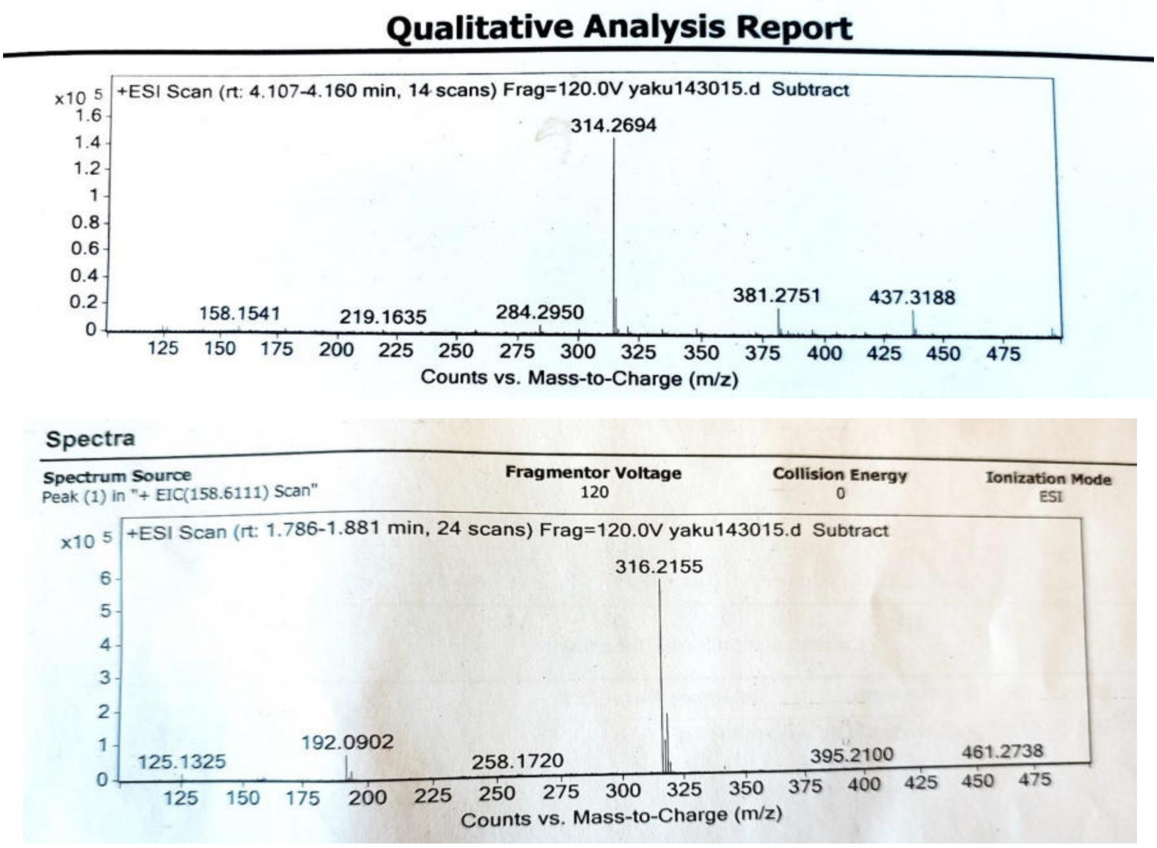

Figure S5. Mass spectra for TEMPO-trapped radicals from LC-MS analysis.

3.2 UV-visible Spectroscopy Studies

Formation of an exciplex in the case of our **PSCat** (**MFB**) is hypothesized based on the previous proposals of Tan and co-workers[28b] as well as Egami, Hamashima and co-workers.[35] Their first clue to a **PSCat**-**SF** exciplex was the reported changes in the UV-vis absorption and fluorescence of **PSCat** in the presence of **SF**. Therefore, we elected to measure UV-vis absorption spectra of individual substrates separately and compared these to the UV-vis absorptions of reaction mixtures corresponding to the 'catalytic method'

(Figures S6-S7). As can be seen below, under these conditions the absorption of the reaction mixtures were identical to that of **SF**. Moreover, no different could be observed between **MFB** and **MB** (methyl benzoate), confirming that the enabling role of the F atom in the **PSCat** is not related to absorptive properties.

Comparison 1 (catalytic method) preparation:

Substrate (Sub):

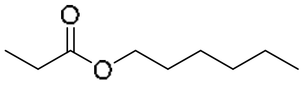

1k

Substrate: 0.15 M, **SF**: 0.1 M, **MFB** (methyl 4-fluorobenzoate): 10 mol% with regards to **SF**, Reaction mixture (RM): 0.15 M Sub + 0.1 M **SF**.

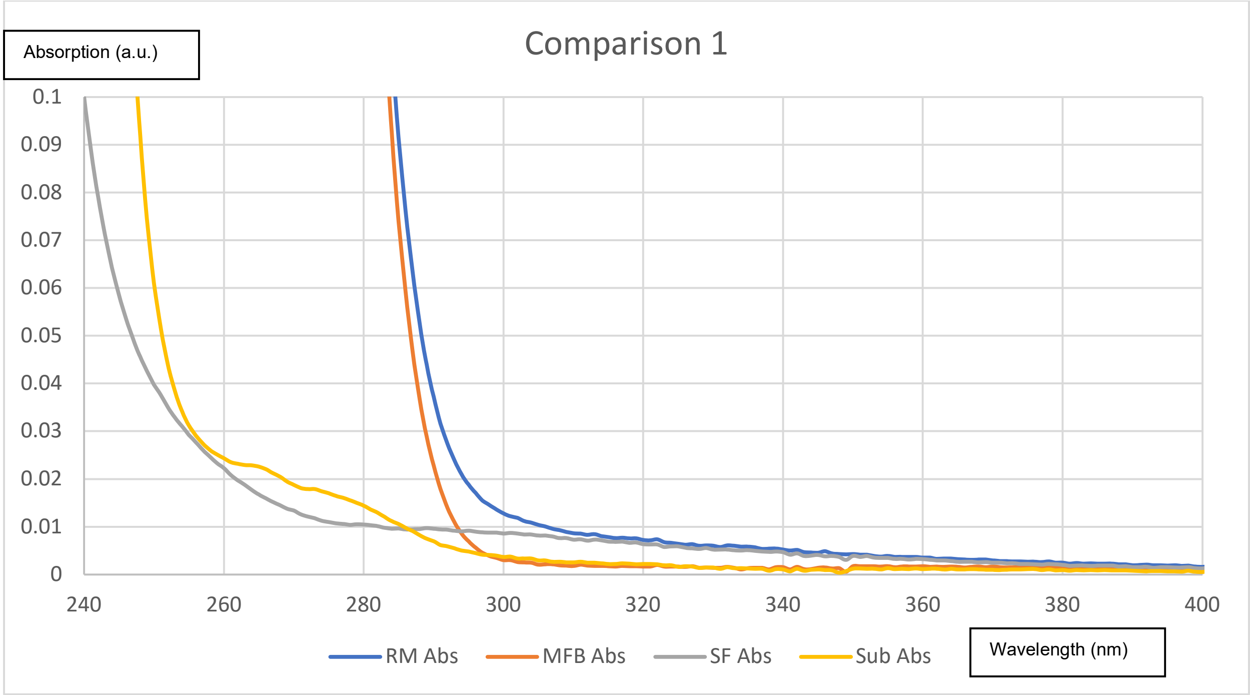

Figure S6. UV-vis data of **Comparison 1**.

Comparison 2 (catalytic method) preparation:

Substrate (Sub):

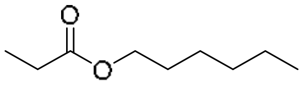

1k

Substrate: 0.15 M, **SF**: 0.1 M, **MB** (methyl benzoate): 10 mol% with regards to **SF**, Reaction mixture (RM): 0.15 M Sub + 0.1 M **SF**

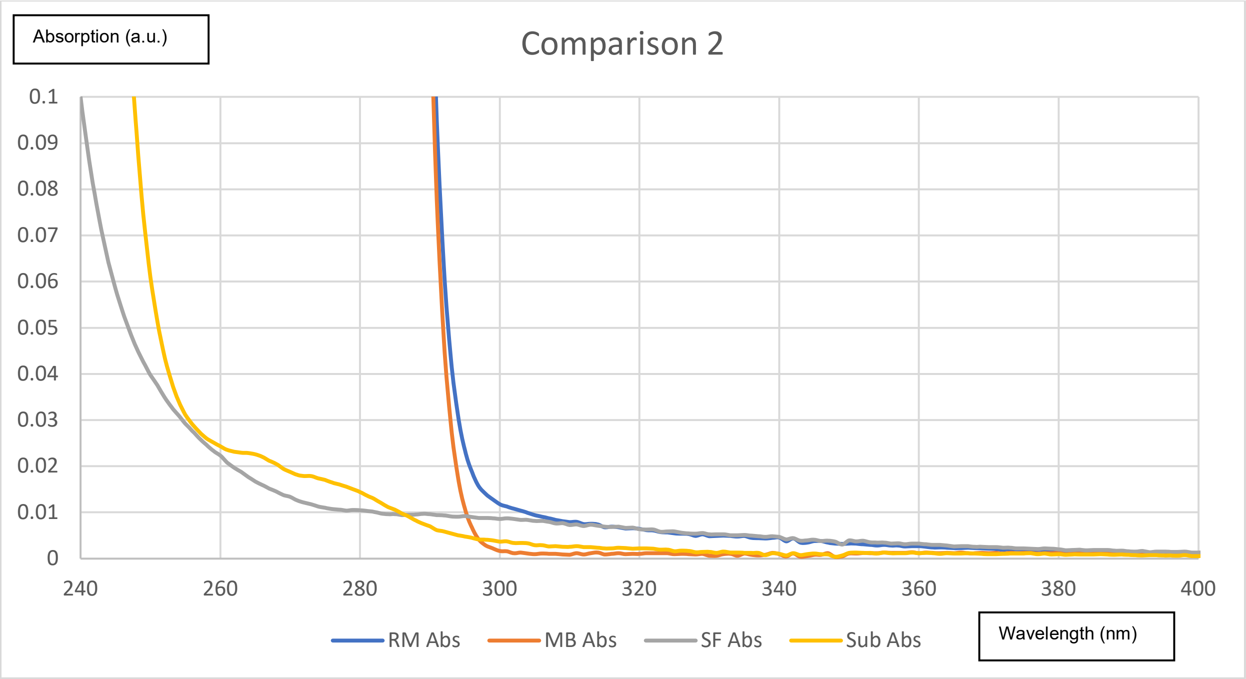

Figure S7. UV-vis data of **Comparison 2**.

We then compared the UV-vis absorptions of reaction components of the reactions of **5b** and **8b** (Figures S8-S9). In the synthetic **PSAux**-type reaction, **5b** gives only traces of fluorinated product while **8b** gives a high yield of fluorinated product (see main manuscript, Table 5). As can be seen below, under these conditions the reaction mixtures gave clear absorptions at the LED wavelength of the synthetic reaction (λ = 400 nm) while individual components absorbed only traces. However, again no difference could be detected between the reaction mixture of **5b** or **8b**, confirming that the F atom in the **PSAux** is not related to absorptive properties

Comparison 3 (auxiliary method) preparation:

Substrate:

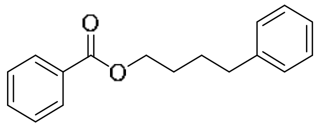

5b

Substrate: 0.15 M, **SF**: 0.1 M, Reaction mixture (RM): 0.15 M **5b** + 0.1 M **SF**

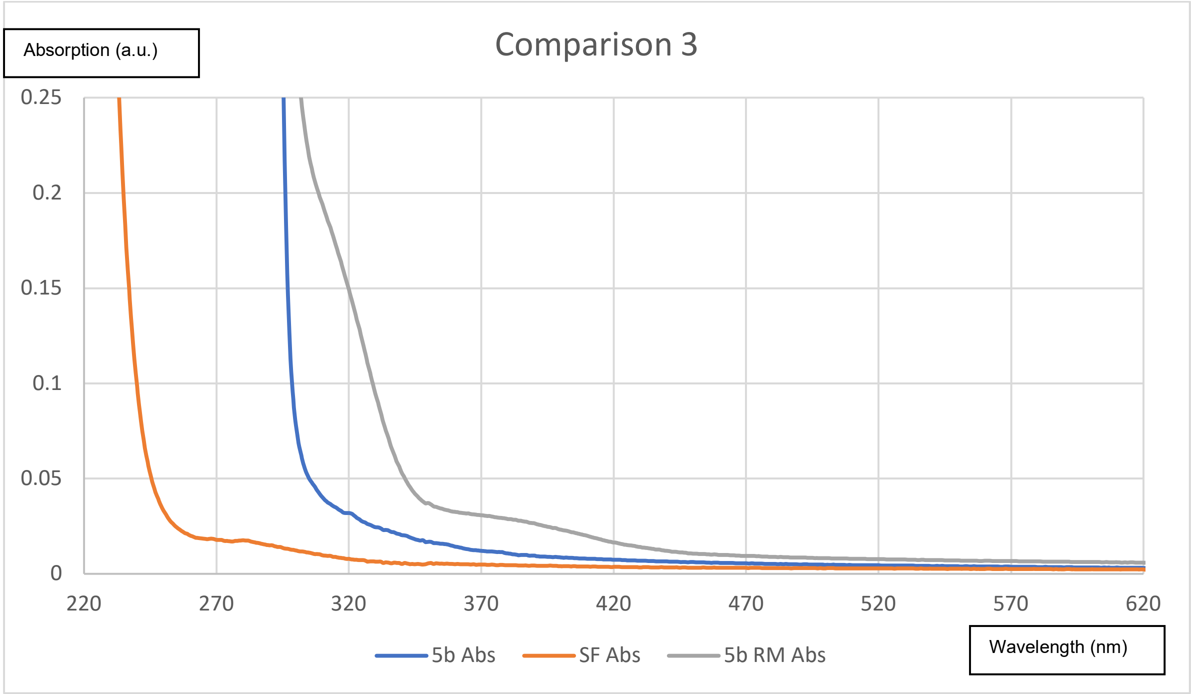

Figure S8. UV-vis data of **Comparison 3**.

Comparison 4 (auxiliary method) preparation:

Substrate:

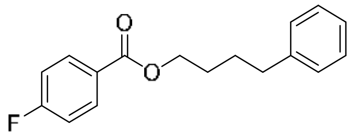

8b

0.15 M, **SF**: 0.1 M, Reaction mixture (RM): 0.15 M **5b** + 0.1 M **SF**

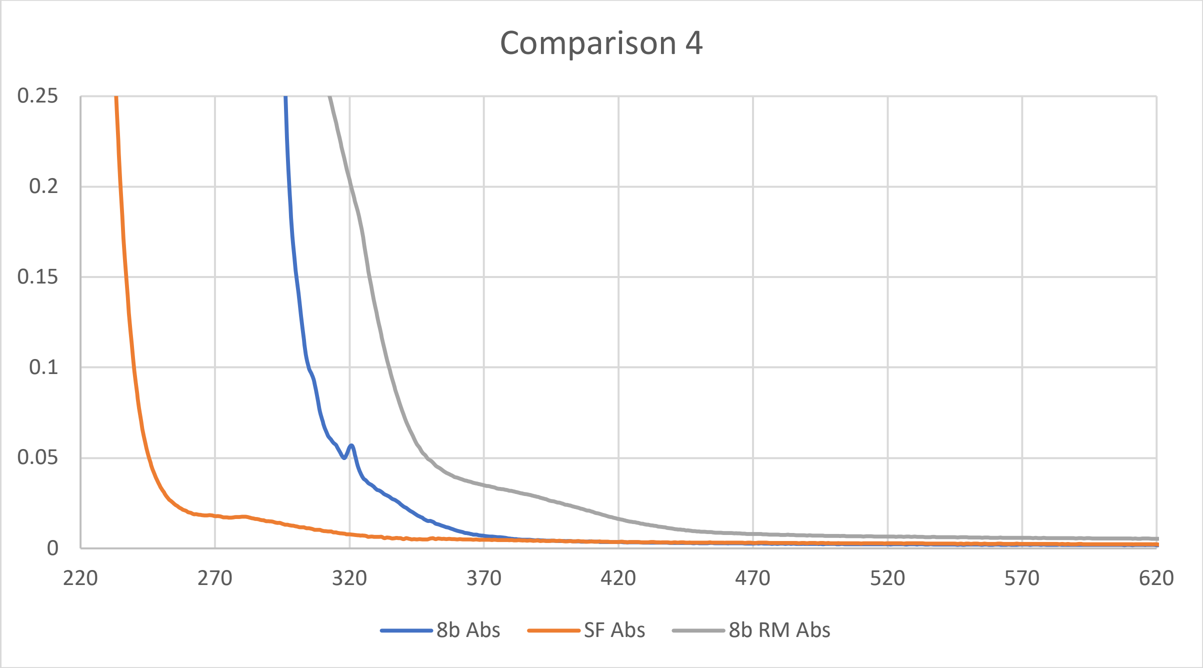

Figure S9. UV-vis data of **Comparison 4**.

3.3 Luminescence Measurements

Information is gained about which reactants (quenchers) are most strongly involved in excited state deactivation by measuring the luminescence intensity and lifetime of the **PSCat** in the presence and absence of an increasing concentration of quenchers. The relationship between luminescence intensity and quencher concentration is described by the Stern-Volmer relationship:[36]

$$\frac{I_0}{I} = 1 + k_{SV} * [Q]$$

Where: I_0 - is the intensity of luminescence without the quencher, I - is the intensity of luminescence with the quencher, [Q] - is the concentration of the quencher and k_{SV} - is the Stern-Volmer constant.

For steady-state luminescence measurements, a 0.2 mM concentration of **MFB** (ca. 10x less than that reaction condition **B** of the fluorination reactions) and the concentrations of **SF** employed ranged from 1.6 mM (ca. 100x less than that of reaction condition **B**) to 15.6 mM (ca. 10x less than that of reaction condition **B**). Spectroscopic concentrations had to be kept lower than preparative concentrations to: i) ensure full solubility of **SF**, ii) ensure that luminescence-derived photon counts did not saturate the detector.

On its own, **MFB** (at the representative 0.2 mM concentration) was stable and absolutely no photodecomposition was observed after repeated measurements for 5 min (Figure S10). In comparison, **SF** (at the representative 15.6 mM concentration) displayed a slight decrease in emission intensity after 6 min,

and this continued to decrease with repeated measurements until 18 min (Figure S11), at a slow rate (−5.6 x 10^{-3} $Ln_{[Emission\ Counts]}$ / min).

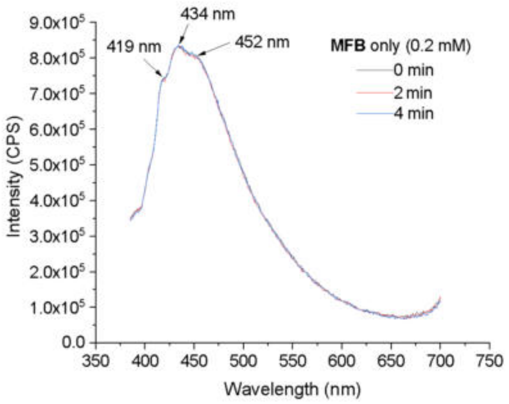

Figure S10. **MFB** photostability, measured repeatedly over time.

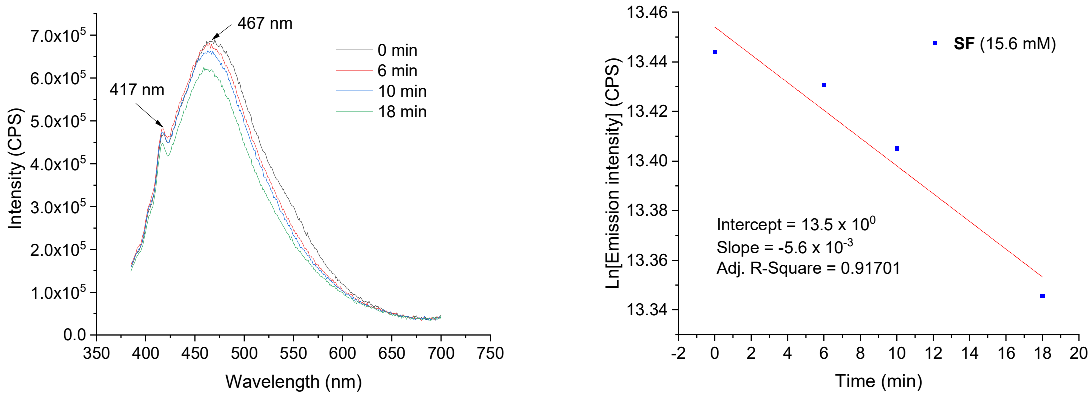

Figure S11. **SF** photostability (left), λ_{max} = 467 nm peak height (right) measured repeatedly over time.

Upon mixing **MFB** (0.2 mM, 12.5 mol% vs. **SF**) with 1.6 mM **SF** (8 eq.), a considerable decrease in the ca. 434 nm peak intensity was observed (by 42%) and the peak shape was altered (Figure S12, left vs. right). The peak was different from both **SF** (no peak at λ_{max} = 467 nm) *and* **MFB** (different shape) alone. This new peak profile barely decreased, even after 5 min of repeated measurements (Figure S12, right). Mixing **MFB** (0.2 mM) with larger excesses of **SF**: 3.8 mM (10 eq.) and 7.9 mM (20 eq.) led to peaks that more closely resembled **SF** *and* led to faster photodecomposition rates of the **SF** peak: −24.9 x 10^{-3} $Ln_{[Emission\ Counts]}$ / min and −62.8 x 10^{-3} $Ln_{[Emission\ Counts]}$ / min (Figures S13,S14). *In conclusion,* ***SF*** *photodecomposes faster in the presence of MFB than in its absence.* This corroborates an energy transfer (E_nT) between **MFB** and **SF**.

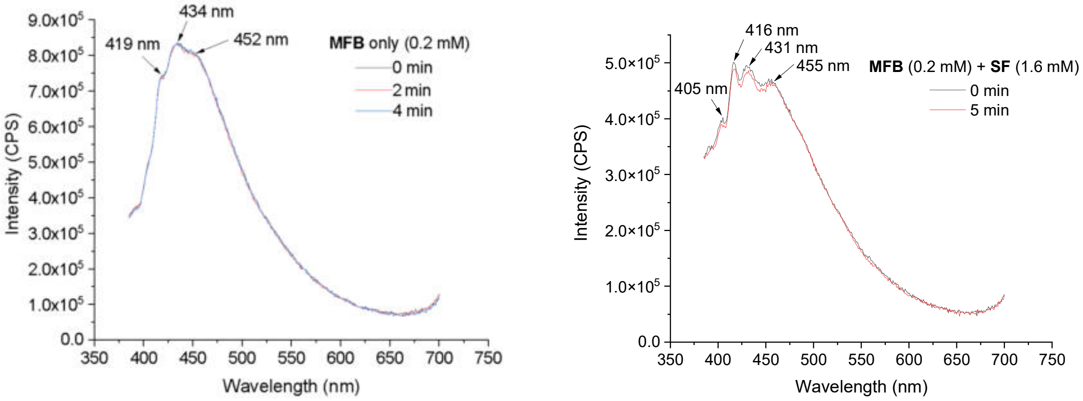

Figure S12. Emission of **MFB** alone (left) and in the presence of 1.6 mM (4 eq.) of **SF** (right).

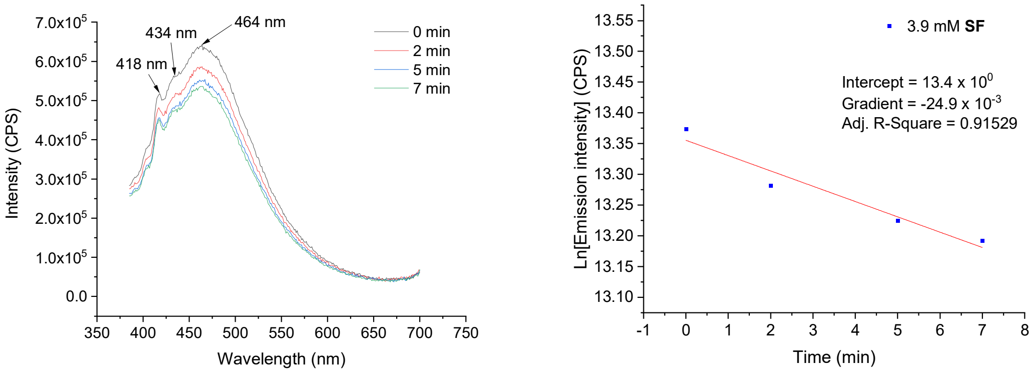

Figure S13. Emission of **MFB** with 3.9 mM (10 eq.) **SF** (left) and λ_{max} = 467 nm peak height (right) measured repeatedly over time.

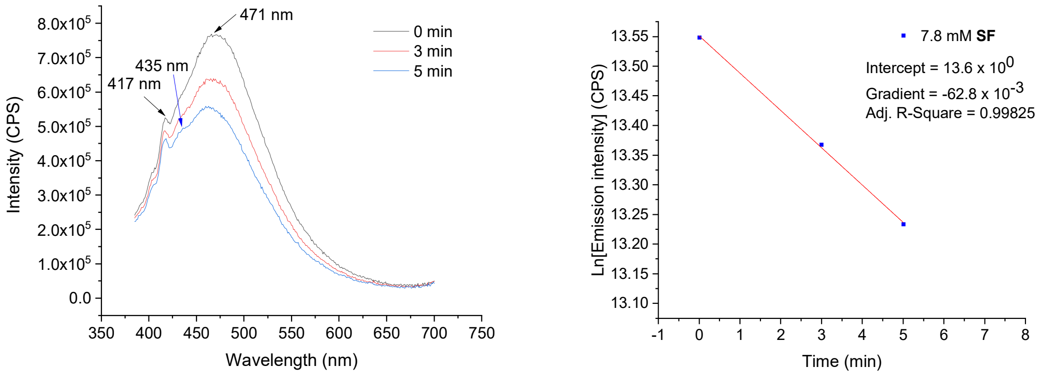

Figure S14. Emission spectra of MFB with 20 eq. **SF** (left) and λ_{max} = 467 nm peak height (right) measured repeatedly over time.

Experiments were repeated (Figures S15, left and right) and examined to higher [**SF**] (1.6→15.6 mM). Due to the increasing photodecomposition rate at higher [**SF**], the exact intensities were difficult to reproduce

between experiments, but the trend was the same – as [**SF**] increases, the intensity of the **SF** peak (at λ_{max} = 467 nm) increases to a point, then decreases as the photodecomposition takes over. As seen in Figure S15 (left, 15.6 mM **SF**), eventually the spectrum resembles that of **MFB** + 1.6 mM **SF**; Figure S12 (right).

Table S7. Steady state luminescence measurement of **MFB** (0.2 mM in MeCN), see Figure S11, right.

Entry	Selectfluor concentration (mM)	Intensity at 433 nm	Intensity at 464 nm
1	0 (**Figure S10**)	833687	-
2	1.6 (8 eq.)	484878	468398
3	3.9 (20 eq.)	562448	643149
4	7.8 (40 eq.)	582848	766066
5	11.7 (60 eq.)	640507	895729
6	15.6 (78 eq.)	507306	573777

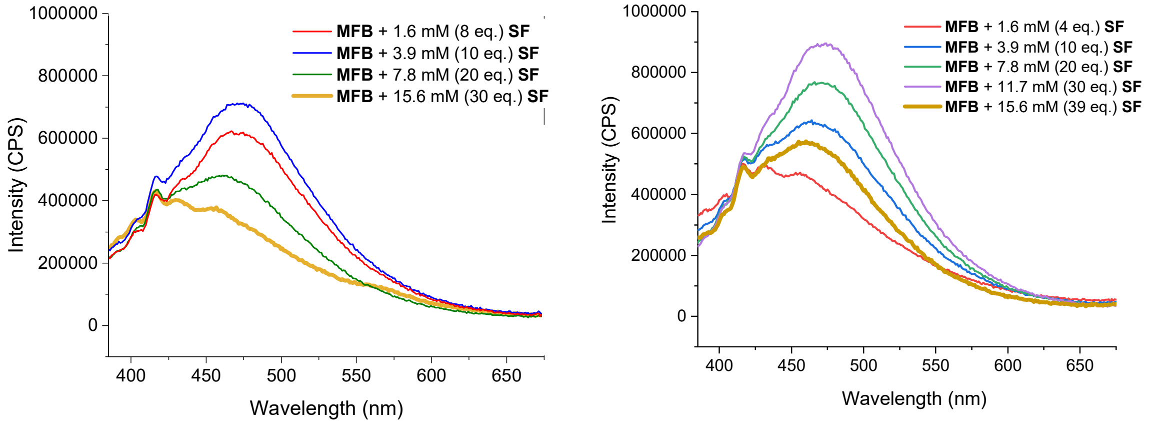

Figure S15. Steady state luminescence measurement of **MFB** (0.2 mM in MeCN) with increasing [**SF**]. Left: Run 1 (peak increases 1.6→7.8 mM, then decreases 7.8→15.6 mM). Right: Run 2 (peak increases 1.6 mM→11.7 mM, then decreases 11.7 mM→15.6 mM).

Consistent with these observations is the formation of an **MFB**-**SF** assembly, either before (preassembly) or after (exciplex) light irradiation (Figure S16, which is stable and accelerates the photodecomposition of **SF** to its radical cation to initiate the reaction. While further, advanced spectroscopic investigations (transient absorption spectroscopy) lie outside of the scope of the current study, *we note that spectroscopic and DFT evidence was provided for an anthraquinone-**SF** exciplex in a related study by Lu, Soo, Tan and co-workers.*[28b]

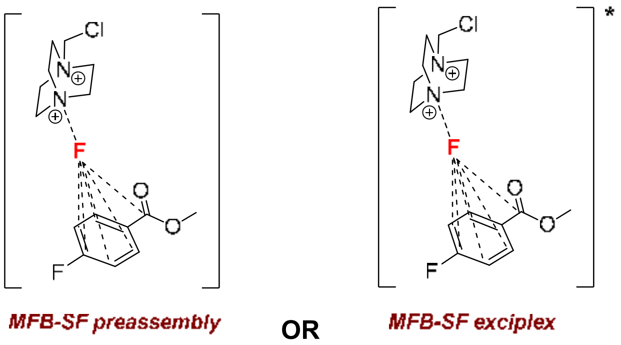

Figure S16. Proposed structure for the **MFB**-**SF** preassembly or exciplex based on literature.[28b]

Lifetime measurements were obtained by Time-Correlated Single Photon Counting (TCSPC) spectroscopy. TCSPC-derived lifetime data are shown in Table S8. Using the same solution (0.2 mM) of **MFB**, TCSPC monitoring emission at 433 nm revealed a biexponential decay where the major component had a lifetime of 18 ns (a sample measured at 0.02 mM gave a similar result). The lifetime of **SF** was then measured (at λ_{max} = 467 nm), affording a biexponential decay where the major component had a lifetime of 28 ns (Figure S17). A mixture of **MFB** (0.2 mM) + **SF** (15.6 mM) was then measured (at λ_{max} = 467 nm), affording a biexponential decay where the major component had a slightly shorter lifetime of 25 ns (Figure S17). It is safe to assume the resulting data corresponds predominantly to **SF** due to i) the monitored wavelength being optimal for **SF** and sub-optimal for **MFB** and ii) the large concentration difference between **SF** and **MFB**. These data consist with the steady-state emission data, revealing that **SF** decays more rapidly in the presence of **MFB**. However, no further insightful information can be extracted from the lifetime data.

Table S8. Lifetime measurements of **MFB**, **SF** and their combination.

Entry	[**MFB**] (mM)	[**SF**] (mM)	Lifetimes τ_1, τ_2 (ns)	CHISQ
1	0.2	0	18.0 (63%), 3.6 (37%)[a]	1.3
2	0	15.6	28.0 (74%), 3.5 (26%)[b]	2.0
3	0.2	15.6	25.4 (79%), 3.6 (21%)[b]	1.4

All decays fitted to two exponentials. Single exponentials gave poor fitting (χ^2 >2.0). [a]Emission wavelength = 433 nm. [b]Emission wavelength = 464 nm.

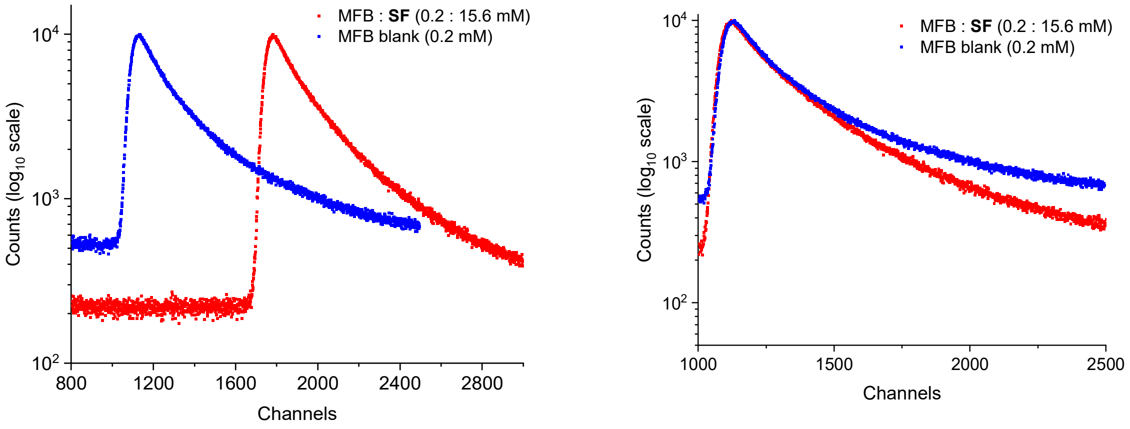

Figure S17. Exponential decays of emissions at 433 nm (blue) and 464 nm (red). Raw decay data (left). Calibrated and overlayed decay profiles (right).

3.4 Advanced NMR-spectroscopic Investigations

3.4.1 General

NMR experiments were performed on a Bruker Avance III HD 600 (600.03 MHz) spectrometer with a 5 mm fluorine selective TBIF probe and TopSpin 3.2 or TopSpin 3.6. For *in situ* illumination-NMR spectroscopy the reported setup with 5 mm amberized thin wall NMR tubes was applied.[37] NMR data were processed, evaluated and plotted with TopSpin 3.2 and 4.0 software. ^{1}H- and ^{13}C-NMR spectra without TMS as internal reference were calibrated on the solvent residual peak of CD_3CN (δ(^{1}H) = 1.94 ppm) or $CDCl_3$ (δ(^{1}H) = 7.26 ppm, δ(^{13}C) = 77.16 ppm); otherwise on TMS (δ(^{1}H) = 0.00 ppm). Further plotting of the obtained data was performed with Origin 2019 and Corel Draw 2020 software.

3.4.2 Chemicals

Commercially available chemicals were, unless otherwise stated, used without further purification. CD_3CN and CH_3CN were distilled over CaH_2, degassed using the freeze-pump-thaw method and stored in a flask with 3Å molecular sieve inside the glove box. TMS was degassed using the freeze-pump-thaw method and stored in a Schlenk-flask with 3Å molecular sieve.

3.4.3 Method for quantitative reaction monitoring by *in situ* illumination-NMR spectroscopy

For illumination of the NMR sample inside the NMR spectrometer an illumination setup as described in literature with a Seoul UV CA3535 series (CUN0GF1A) LED, emitting at a peak wavelength of 405 nm, was applied.[37] The LED was operated with maximum forward current (1.4 A). The kinetic studies were performed at r.t. (25 °C) as the standard reaction. The starting point of each kinetic (t = 0 s) was measured without illumination and during illumination, ^{1}H- and ^{19}F-spetra were recorded alternately. The NMR experiments were measured as following to obtain spectra for quantitative evaluation: Due to the high concentration of the NMR samples (see NMR sample preparation), a S/N of > 250:1 was already achieved

in single scan experiments. Additional S/N enhancement was achieved by removing heteronuclear ^{1}H-^{19}F-coupling using a zgig pulse program (inverse gated decoupling). A relaxation delay d1 = 300 s was applied to ensure full relaxation of all signals before every scan and pulse lengths for ^{1}H-experiments were calibrated.

3.4.4 Reaction monitoring of photosensitization auxiliary method

3.4.4.1 NMR sample preparation

The entire NMR sample preparation was executed under inert gas conditions. The actual preparation was done inside glove box, the glass fiber insert for *in situ* illumination was inserted following Schlenk line technique. Selectfluor® (22.0 mg, 62.1 µmol, 1.0 eq.) was weighed in an oven dried amberized 5 mm thin wall NMR tube. A 300 µL aliquot of substrate **8b** in anhydrous and degassed CD_3CN (500 µL) (amount of substrate **8b**: see Table S9) and pentafluorobenzene (2.9 µL, 18.6 µmol, 0.3 eq.) as internal standard were added to the NMR tube. In order to maximize the amount of dissolved Selectfluor® and to ensure a homogeneous sample, the sample was shaken intensively. Lastly a *in situ* illumination insert as described in literature (glass fiber: Thorlabs; fiber type: MM, FP1500URT, 0.50 NA, 300 - 1200 nm, 1500 µm core) was inserted into the NMR tube and fastened with enough parafilm to prevent oxygen and moisture from penetrating the sample.[37]

Table S9. Different amounts of substrate for reaction monitoring of photosensitization auxiliary method.

entry	equivalents	concentration [mM]	amount of substrate [µmol][a]	mass [mg][a]
1	1.5	310.5	155.3	42.3
2	2.0	414.0	207.0	56.4
3	2.5	517.5	258.8	70.5
4	3.0	621.0	310.5	84.6

[a] The amounts are based on the 500 µL stock solution.

3.4.4.2 Reaction monitoring of photosensitization auxiliary method applying different substrate equivalents

For a first insight in the reaction kinetic of the photosensitization auxiliary method, a reaction from the synthetic part of this work with the same concentrations was investigated (Scheme S3).

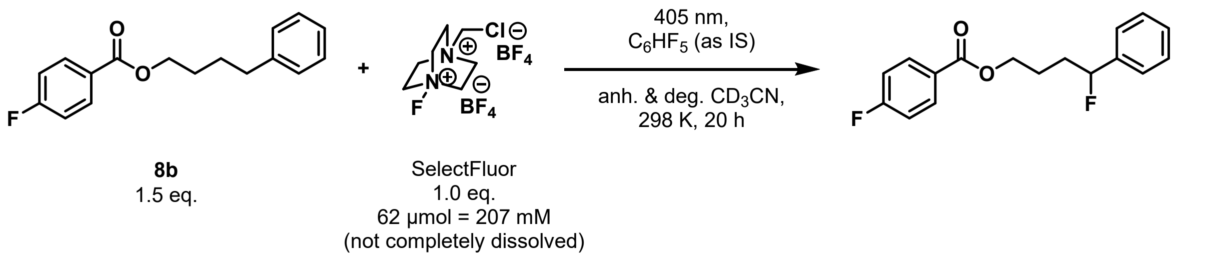

Scheme S3: Model reaction for reaction monitoring by *in situ* illumination-NMR spectroscopy of photosensitization auxiliary method.

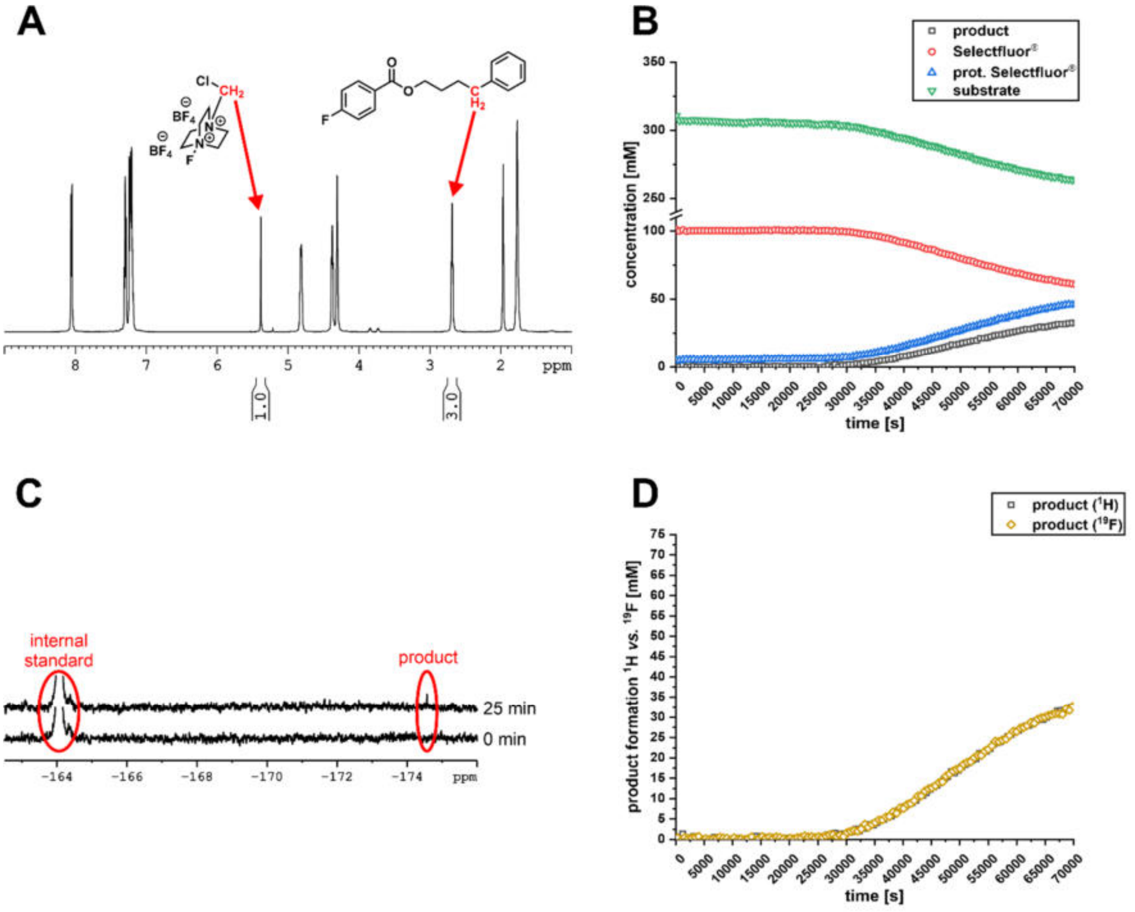

E

	slope (yEr±) [mM/s]	R^2 (cor.)
product	9.64E-4 (0.89E-5)	0.998
Selectfluor®	11.7E-4 (0.86E-5)	0.998
Prot. Selectfluor®	11.9E-4 (0.76E-5)	0.999
substrate	11.7E-4 (1.13E-5)	0.997

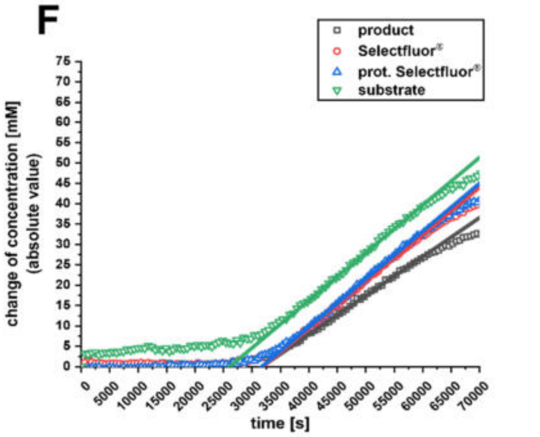

Figure S18. Data from the model reaction for *in situ* illumination NMR-spectroscopy shown in Scheme S3. **A:** Due to the high concentration and moderate solubility of Selectfluor®, it is not completely dissolved. Therefore, substrate **8b** and Selectfluor® are present in a ratio of 1.0:3.0 instead of 1.0:1.5. **B:** The reaction does not occur over a typical reaction profile. Instead, an induction phase of 9.2 h is required before the reaction begins with a first-order rate. **C:** The first traces of product, however, can be detected after just 25 minutes. **D:** Product build-up curves were measured over ^{1}H{^{19}F} and ^{19}F{^{1}H} spectra without discernible difference. **E:** Rates for the linear build-up of the different reagents and products. The substrate and Selectfluor® are consumed at the same rate as the protonated Selectfluor® is formed. The product forms at a slightly slower rate. **F:** For a better comparison of the different processes, the absolute values of concentration changes are shown.

Due to the high concentration and moderate solubility of Selectfluor® in CD_3CN, it has not completely dissolved. Therefore, substrate **8b** and Selectfluor® were not in a ratio of 1.5:1.0 as weighed, but in a ratio of 1.0:3.0 (Figure S18, **A**). The reaction shows a very long induction phase of approximately 9.2 h. Afterwards product forms with a build-up curve typical for a first-order reaction (Figure S18, **B**). Interestingly however, the first traces of product can be detected after 25 minutes in the $^{19}F\{^1H\}$ -spectra (Figure S18 **C**), which provide the same kinetic profile as the ^1H measurements (Figure S18, **D**). Looking at the rates for the different reagents and products during the linear build shows a similar consumption of substrate (11.7E-4 mM/s) and Selectfluor® (11.7E-4 mM/s) as the fluorinating agent. Protonated Selectfluor® (11.9E-4 mM/s), a stoichiometric by-product, also forms at the same rate. The monofluorinated product is obtained at a somewhat slower rate (9.64E-4 mM/s). This indicates that, in addition to the monofluorination, other reactions such as multiple fluorination or elimination reactions must take place to a small component (Figure S18, **E** and **F**).

For further studies the effect of substrate loading on the kinetic profile has been examined. 1.5, 2.0, 2.5 and 3.0 equivalents of substrate **8b** were examined for this.

A

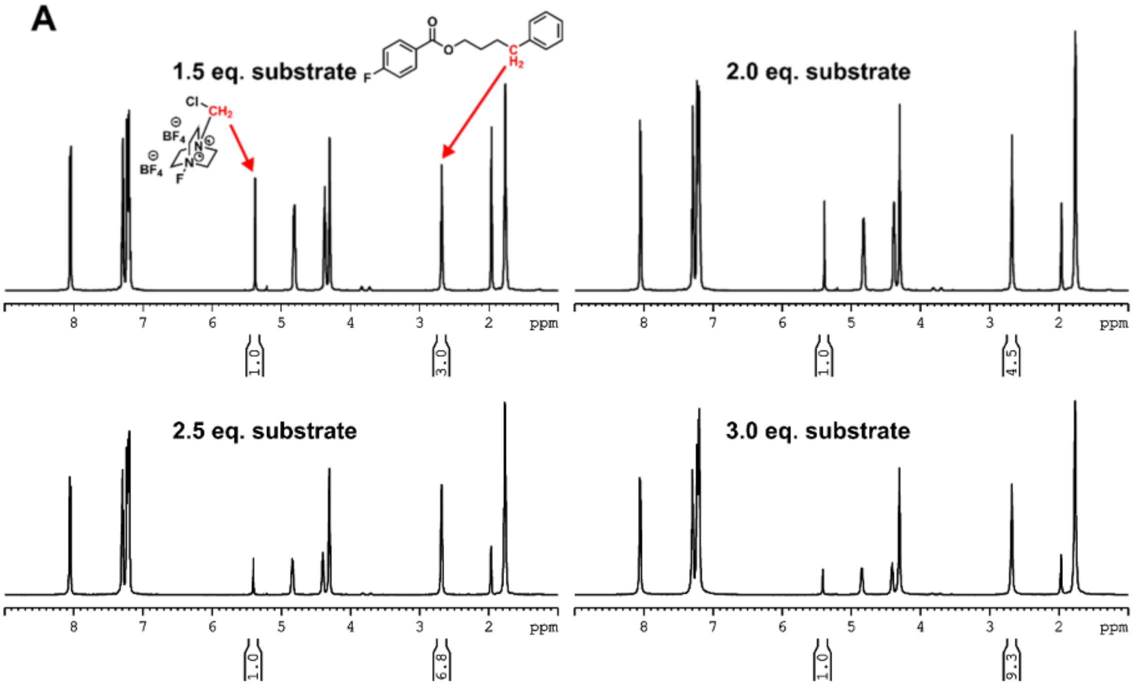

B

Ratio Substrate/Selectfluor® (weighed in)	Ratio Substrate/Selectfluor® (dissolved)
1.5 : 1.0	3.0 : 1.0
2.0 : 1.0	4.5 : 1.0
2.5 : 1.0	6.8 : 1.0
3.0 : 1.0	9.3 : 1.0

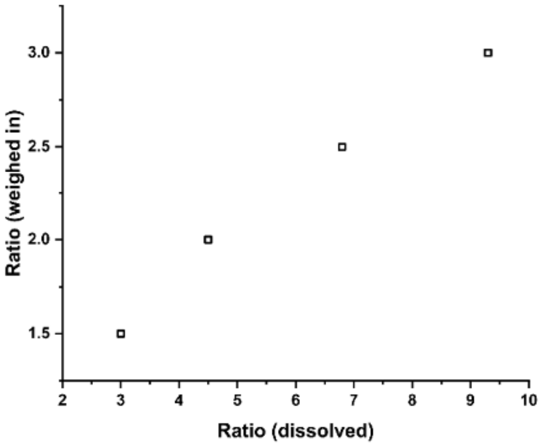

Figure S19. Comparison of the ratios of substrate **8b** and Selectfluor® weighed in *vs.* in solution for different amounts of substrate **8b**. **A:** Based on the integrals of Selectfluor®- and substrate-signals, the ratio of the two reagents in solution can be determined. **B:** Evaluation of the different ratios shows that the solubility of Selectfluor® decreases as the amount of substrate increases.

Based on the NMR spectra (Figure S19, **A**), it can be seen that due to the moderate solubility of Selectfluor®, the ratios of Selectfluor® to substrate **8b** in solution do not correspond to those weighed in. *There is a clear trend that the solubility of Selectfluor® continues to decrease as the substrate equivalents increases*. While 50% of Selectfluor® are dissolved at the beginning of the reaction with 1.5 eq. of substrate **8b**, it is only 32% when 3.0 eq. substrate are applied.

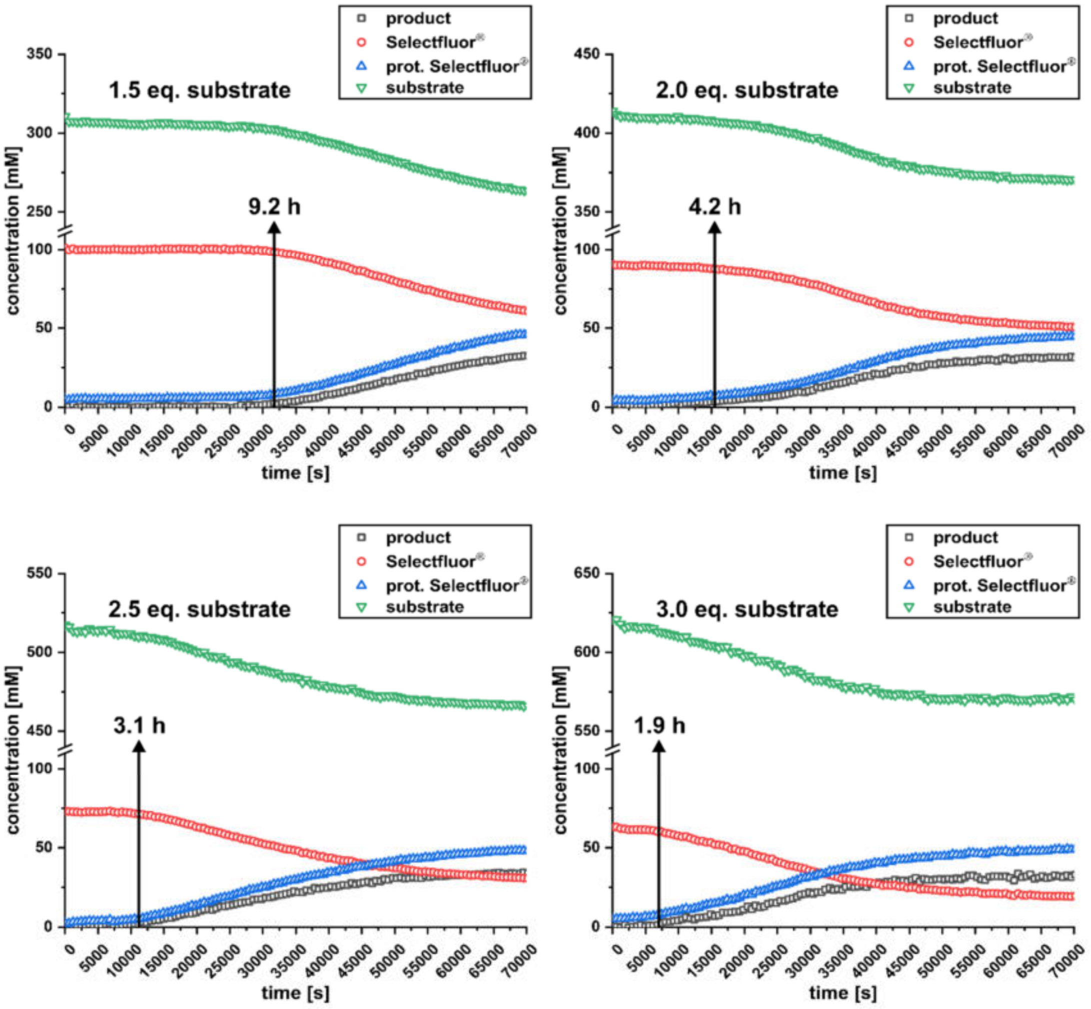

Figure S20. Kinetic profiles of the photosensitization auxiliary reaction with different substrate loadings. As the amount of substrate **8b** increases, the induction phase becomes shorter. Apart from that, the reaction profiles are very similar.

When repeating the reaction with different amounts of substrate (Figure S20), a clear trend emerges: The induction phase with scarcely any product formation becomes shorter as the substrate loading increases. While the standard reaction with 1.5 eq. of substrate **8b** has an induction phase of 9.2 h, it takes ca. 4.2 h with 2 eq. of substrate **8b**. With 2.5 eq. it takes ca. 3.1 h, while when using 3 eq. substrate **8b** it only takes ca. 1.9 h until product formation with a profile typical of a first-order reaction begins. In summary, by doubling the amount of substrate **8b**, *the induction phase can be shortened by 79%.*

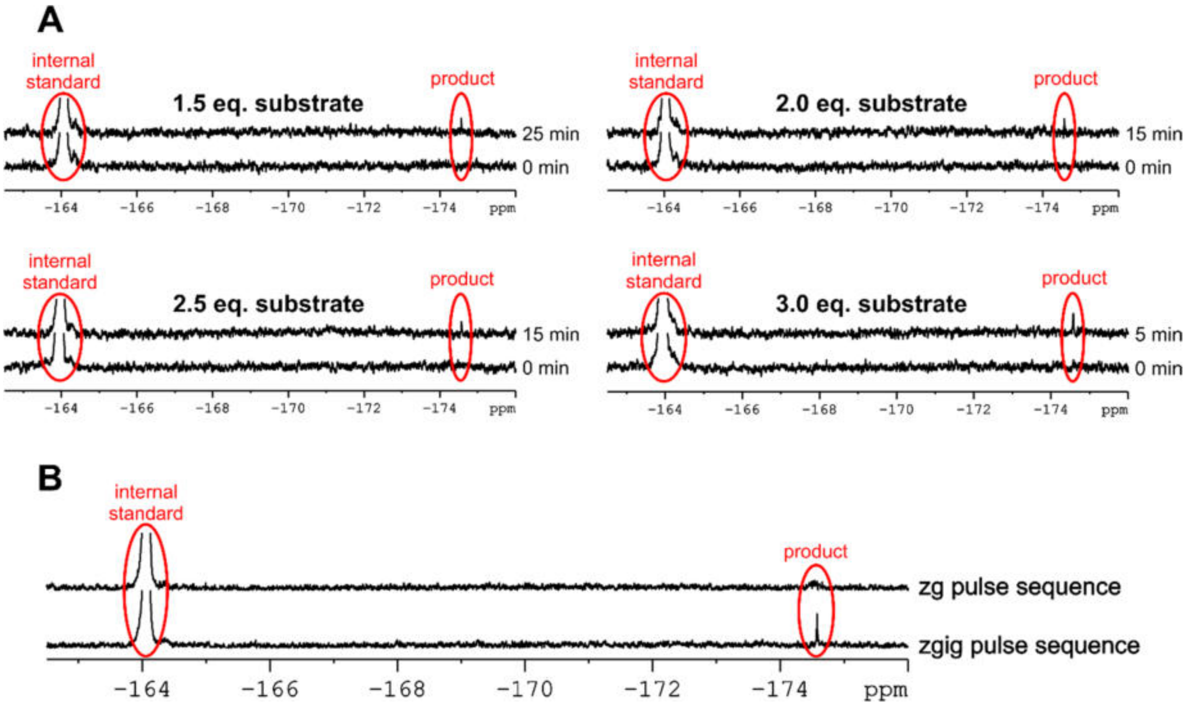

Figure S21. A: Despite the long induction phases, first traces of product can be detected independent of the amount of substrate **8b** after a significantly shorter time. However, the first-order type product formation begins much later. **B:** By using a zgig pulse sequence instead of the standard zg pulse sequence, traces of product can be identified which would otherwise be almost indistinguishable from baseline noise.

Even if it takes more than 9 h for significant product formation to begin, the first traces of product can be detected much earlier. In the standard reaction with 1.5 eq. of substrate **8b**, a small product signal can be detected after just 25 minutes. Increasing the amount of substrate also reduces the time until an initial, small amount of product starts to form. With 2.0 and 2.5 eq. substrate, products can be detected after just 15 minutes. With 3.0 eq. substrate, a clear product signal can already be identified in the first spectrum after the illumination has been started (Figure S21 **A**). This also shows the clear advantage of the zgig pulse sequence. When using a zg pulse sequence, the standard pulse sequence for routine NMR measurements, the product signal is obtained as a multiplet due to the heteronuclear ^{1}H-^{19}F coupling. Due to the low concentration of the product at the beginning of the reaction and hence a low signal intensity, this is hardly distinguishable from the baseline noise of the ^{19}F-spectrum (Figure S21 **B** top). In contrast, inverse gated decoupling, which is implemented in the zgig pulse sequence, suppresses the heteronuclear ^{1}H-^{19}F coupling. As a result, the product signal appears as a more intense singlet, which clearly stands out from the baseline noise (Figure S21 **B** bottom). However, since there is no signal enhancement through NOE as it is with power gated decoupling (zgpg pulse sequence), the standard method for decoupling, the spectra obtained are still quantitative.

To gain insight in the role of product, the reaction with 1.5 eq. substrate was repeated with additional 10 mM product.

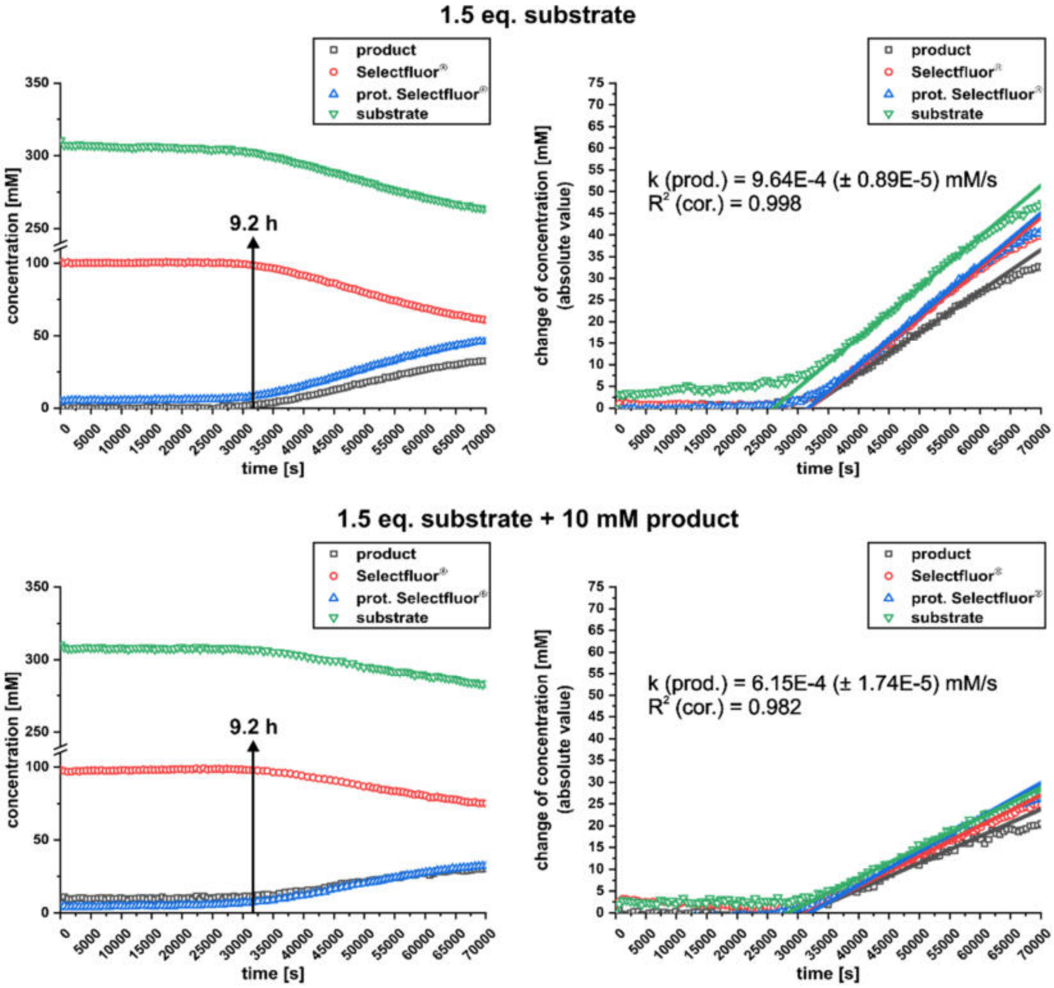

Figure S22. Kinetic profiles of the photosensitization auxiliary reaction with 1.5 eq. substrate **8b** (top) and additional 10 mM product **8b** (bottom). Since the additional product whether shortens the induction phase nor increases the rate for product formation an autocatalytic effect of the product can be excluded.

Comparison of the reaction profiles of the standard reaction (Figure S22 top) and the one with additional 10 mM product **8b** (Figure S22 bottom) shows that the length of the induction phase remains unchanged. In both reactions, it takes ca. 9.2 h before a distinct product formation occurs. A positive contribution of the product to the reaction mechanism can also be ruled out when looking at the rate of product formation. The additional product even reduces the reaction rate for product formation in the linear build-up by 36% from 9.64E-4 mM/s to 6.15E-4 mM/s.

1.5 eq. substrate

	slope (yEr±) [mM/s]	R^2 (cor.)
product	9.64E-4 (0.89E-5)	0.998
Selectfluor®	11.7E-4 (0.86E-5)	0.998
Prot. Selectfluor®	11.9E-4 (0.76E-5)	0.999
substrate	11.7E-4 (1.13E-5)	0.997

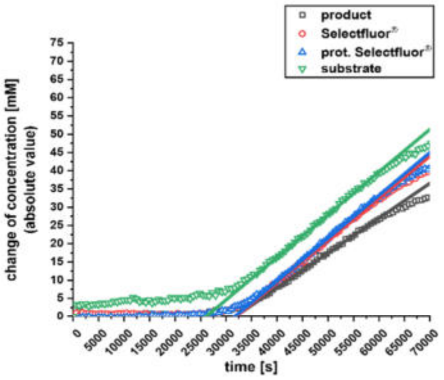

2.0 eq. substrate

	slope (yEr±) [mM/s]	R^2 (cor.)
product	9.92E-4 (2.55E-5)	0.986
Selectfluor®	12.4E-4 (1.56E-5)	0.997
Prot. Selectfluor®	12.3E-4 (1.30E-5)	0.998
substrate	13.5E-4 (2.47E-5)	0.993

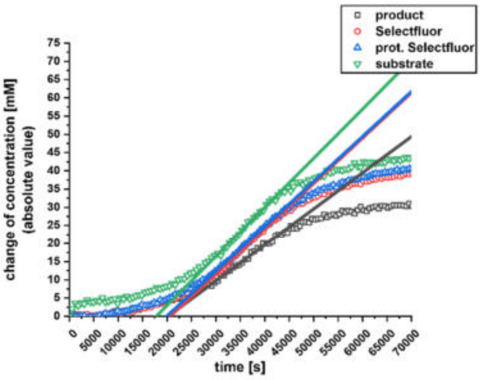

2.5 eq. substrate

	slope (yEr±) [mM/s]	R^2 (cor.)
product	8.46E-4 (1.27E-5)	0.994
Selectfluor®	10.2E-4 (1.02E-5)	0.997
Prot. Selectfluor®	10.8E-4 (1.29E-5)	0.996
substrate	11.4E-4 (2.43E-5)	0.988

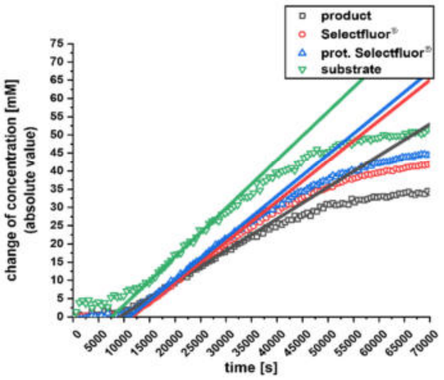

3.0 eq. substrate

	slope (yEr±) [mM/s]	R^2 (cor.)
product	9.70E-4 (2.37E-5)	0.983
Selectfluor®	11.6E-4 (1.06E-5)	0.998
Prot. Selectfluor®	11.7E-4 (1.25E-5)	0.997
substrate	13.7E-4 (3.18E-5)	0.985

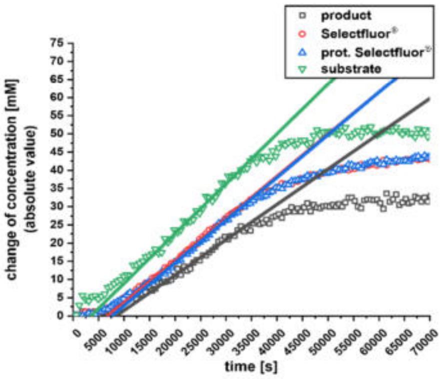

Figure S23. Rates and reaction kinetics (plotted as absolute changes) for photosensitization auxiliary reactions with different substrate loadings. With an increasing amount of substrate **8b**, the induction phase shortens. The rate at which the concentrations of the different species change is unaffected.

Plotting the absolute values of the concentration changes of all species involved, i.e. reagents (substrate **8b** and Selecfluor®) and products (product and protonated Selectfluor®), enables to read the transformation into each other out directly from the course of the curve. Overall, an increased substrate loading primarily affects the induction phase of the reaction. As in Figure S20, a shortening of the induction phase with increasing amount of substrate **8b** is observable. However, the amount of product that is generated and the rate at which this happens remains unchanged. The situation is similar with the other three species shown. The fact that everything of consumed Selectfluor® (**SF**) is transformed into its protonated form is reflected by the almost identical curves. The slightly smaller amount of generated product in all reactions indicates side reactions taking place in parallel, such as multiple fluorination and elimination reactions. A possible explanation for the shortening of the induction phase as the substrate loading increases can be found in the initial curve of the substrate kinetics. From almost the first spectrum, substrate **8b** is consumed up to a concentration of 5 mM. This process begins earlier and more pronounced as the amount of substrate increases. This fact in combination with the traces of product, which can also be detected from the beginning (see Figure S21), possibly indicates a so far unknown pre-aggregate, which is required for effective product formation. Further studies are ongoing to uncover the nature of aggregation changes.

3.4.5 Reaction monitoring of photocatalytic C(sp^3)-H fluorination

3.4.5.1 NMR sample preparation

The NMR samples were prepared as for reaction monitoring of photosensitization auxiliary method under inert gas conditions (see Reaction monitoring of photosensitization auxiliary method, NMR sample preparation).

For monitoring the influence of catalyst loading a stock solution of **MFB** (16.6 mM in anhydrous and degassed CD_3CN) was prepared, which was stored inside a glove box refrigerator. Depending on the catalyst loading to be examined (see Table S10), the corresponding amount was transferred from the stock solution to an NMR tube loaded with Selectfluor® (22.0 mg, 62.1 µmol, 1.0 eq.) and then diluted with enough anhydrous and degassed CD_3CN to reach a volume of 300 µL. Afterwards, substrate **1k** (14.7 mg, 16.8 µL, 93.2 µmol, 1.5 eq.) and pentafluorobenzene as internal standard (2.9 µL, 18.6 µmol, 0.3 eq.) were added. The sample was shaken intensively to ensure a homogeneous and saturated Selectfluor® solution. Lastly a *in situ* illumination insert as described in literature (glass fiber: Thorlabs; fiber type: MM, FP1500URT, 0.50 NA, 300 - 1200 nm, 1500 µm core) was inserted into the NMR tube, which was fastened with enough parafilm to prevent oxygen and moisture from penetrating the sample.[37]

Table S10. Different catalyst loadings for reaction monitoring of photocatalytic C(sp^3)-H fluorination.

Entry	Catalyst loading [mol%]	Amount of stock solution [µL]
1	1	37.5
2	5	188
3	5	188
4	8	300

To investigate the influence of substrate loading a stock solution of catalyst **MFB** and pentafluorobenzene as internal standard (1.0 mM respectively 7.5 mM in anhydrous and degassed CD_3CN/ CH_3CN 1:1) was prepared, which was stored inside glove box refrigerator. Depending on the substrate loading to be examined (see Table S11), the corresponding amount of substrate **1k** and a 300 µL aliquot of the catalyst/ internal standard stock solution were added to the with Selectfluor® (10.6 mg, 30.0 µmol, 1.0 eq.) loaded NMR tube. To ensure a homogenous sample, the NMR tube was shaken intensively and finally a *in situ* illumination insert as described in literature (glass fiber: Thorlabs; fiber type: MM, FP1500URT, 0.50 NA, 300 - 1200 nm, 1500 µm core) was inserted into the NMR tube and fastened with enough parafilm to prevent oxygen and moisture from penetrating the sample.[37]

Table S11. Different substrate loadings for reaction monitoring of photocatalytic C(sp^3)-H fluorination.

Entry	Equivalents	Volume [µL][a]	Amount of substrate [µmol][a]
1	1.5	8.1	45
2	2.0	10.8	60
3	2.0	10.8	60
4	3.0	16.3	90

3.4.5.2 Reaction monitoring of photocatalytic C(sp^3)-H fluorination with different catalyst and substrate loadings

To gain information about the reaction kinetic of photocatalytic C(sp^3)-H fluorination, the following reaction from the synthetic part of this work was chosen.

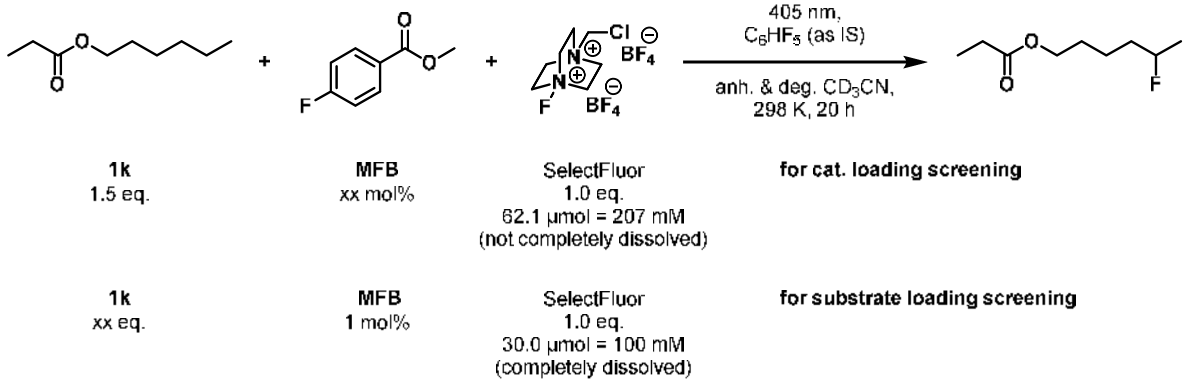

Scheme S4: Model reaction for reaction monitoring of photocatalytic C(sp^3)-H fluorination by *in situ* illumination-NMR spectroscopy.

As with the photosensitization auxiliary fluorination reaction, it was investigated how the kinetics of the reaction change by varying the catalyst loading or the amount of substrate **8b**. The same concentrations as in the synthetic part of this work were used to screen for the influence of catalyst loading. To investigate the influence of the substrate, however, the concentrations of all involved reagents were decreased until all Selectfluor® weighed in was also completely dissolved, which was the case at c = 100 mM. So this time the weighed-in conditions correspond precisely to the dissolved ones.

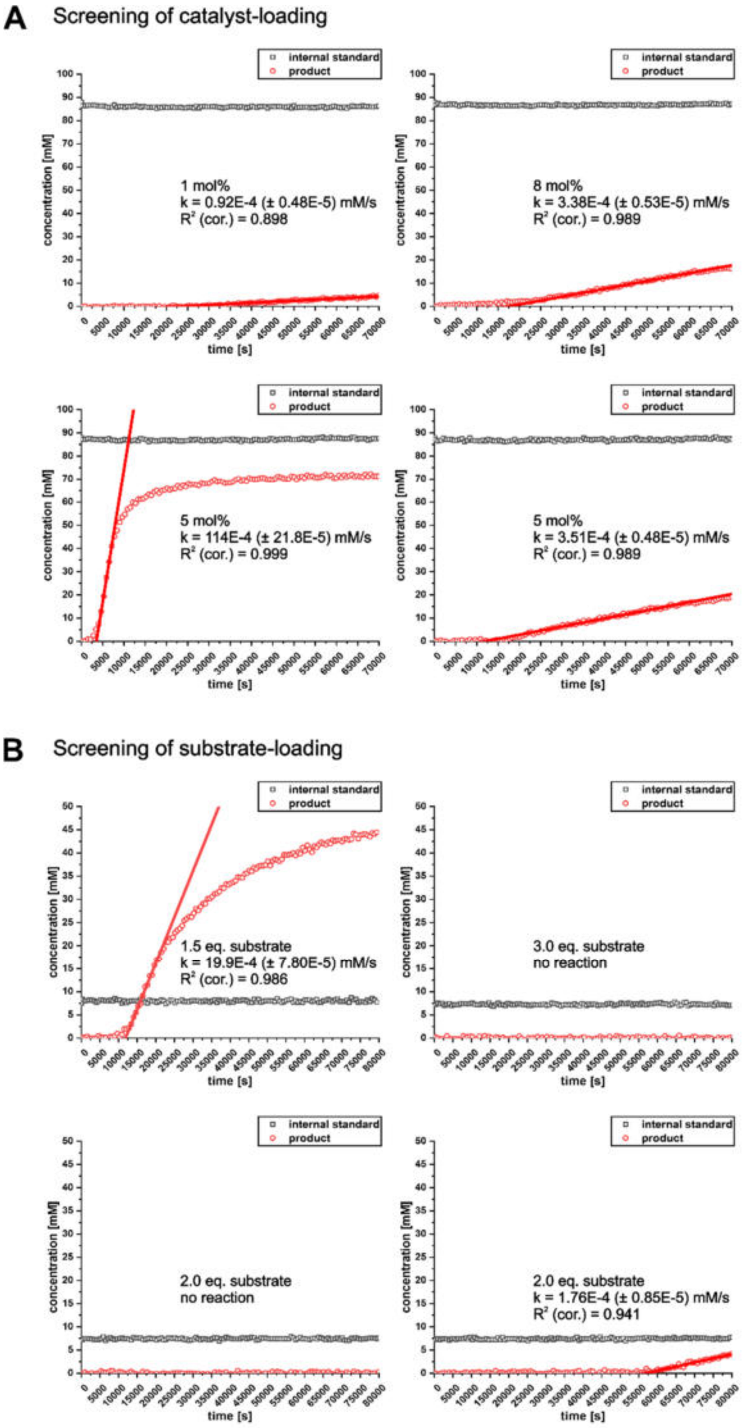

Figure S24. Reaction monitoring of photocatalytic C(sp^3)-H fluorination reaction with different catalyst- and substrate loadings. Similar to the photosensitization auxiliary fluorination reactions an induction phase is required before the reaction proceeds *via* a first order reaction profile. **A:** Product curves with different catalyst loadings: The reaction with 5 mol% was performed twice, one yielding product with a very high rate and one with a rate comparable to the other catalyst loadings. **B:** Reaction profiles showing the product formation with different substrate loadings: While the reaction with 1.5 equivalents of substrate **1k** gave the product in a very good rate and conversion, no product was obtained with increasing the amount of substrate. The reaction with 2.0 eq. was done twice, once product was generated, but after a very long induction phase and with a rate, similar to those from catalyst loading screening.

Performing photocatalytic C(sp^3)-H fluorination reaction with 1 mol% catalyst loading, product was generated after an induction phase of 8.3 h at a rate of 0.92E-4 mM/s. Increasing the catalyst loading to 5 or 8 mol% improved the product formation in a similar way. In both cases the induction phase is shortened by 41% to about 4.9 h and the product is formed at a rate almost 4 times higher than with 1 mol% catalyst loading (3.51E-4 mM/s for 5 mol% and 3.38E-4 mM/s for 8 mol%). In one example with 5 mol% catalyst loading, product was generated after approximately 40 min with a rate of 119E-4 mM/s. As with the screening of catalyst loading (Figure S24 **A**), different substrate loadings gave results that were difficult to classify (Figure S24 **B**): In contrast to the 1.5 eq. reaction of catalyst loading screening, here a by far more pronounced product formation could be observed. Product was yielded after an induction phase of 3.1 h with a rate of 19.9E-4 mM/s. When increasing the substrate loading, however, no product was obtained. Only in one example product was generated after a very long induction phase of 15.6 h and a rate comparable to that from the catalyst loading studies (1.76E-4 mM/s). Despite the generally less pronounced and slower product formation, “catalytic” PS fluorination nevertheless shows a reaction behavior like photosensitization auxiliary fluorination, consisting of an induction phase followed by first-order reaction type product generation.

In summary, the photocatalytic C(sp^3)-H fluorination reaction kinetics were *poorly reproducible*. This emphasizes the importance of agitation of the slurry reaction mixture (in the NMR tube experiments, **SF** sediments at the bottom of the tube). Moreover, it highlights a *key advantage of the “auxiliary” PS fluorination in terms of robustness and reliability*. The reason for the high robustness and generally better performance of “auxiliary” PS fluorination compared to the “catalytic” method, however, is the subject of further investigations.

3.4.6 Method for aggregate-investigations by diffusion ordered spectroscopy (DOSY) in photosensitization auxiliary fluorination

For DOSY measurements the dstebpgp3s pulse program, a convection suppressing DSTE (double stimulated echo) pulse sequence developed by Jerschow and Müller was applied in a pseudo 2D mode.[38] The diffusion time delay was set to 100 ms and a gradient pulse of 1350 μs was applied. Sine.100 as gradient program and a linear gradient ramp with 20 increments between 10% and 95% of the maximum gradient strength were used. For z-only gradients 100 %, -13.17%, -17.13% and -15.17% were used. NMR data were processed and evaluated with TopSpin 3.2 (Topspin T1/T2 Module). Diffusion coefficients and average volumes were obtained according to Jerschow and Müller.[38]

3.4.6.1 NMR sample preparation

The entire NMR sample preparation was executed similar as for reaction monitoring of photosensitization auxiliary fluorination under inert gas conditions (see Reaction monitoring of photosensitization auxiliary fluorination, NMR sample preparation). The actual preparation was done inside glove box, anhydrous and degassed TMS and in case of the DOSY reaction monitoring also the glass fiber insert for *in situ* illumination were added following Schlenk line technique. To examine the pure components, depending on the concentration, they were weighed directly into the NMR tube or a dilution series was used to achieve the respective concentration (see Table S12). The sample preparation for investigating the reaction mixtures (1.5 eq. respectively 3.0 eq. substrate **8b**) was carried out as for the reaction monitoring of “auxiliary ”PS fluorination (see Table S9) but without additional pentafluorobenzene as internal standard.

Table S12. Different amounts of substrate and Selectfluor® for DOSY studies of the pure compounds in anhydrous and degassed CD_3CN at 25 °C.

Entry	Compound	Concentration [mM]
1	substrate **8b**	621.0
2		310.5
3		1.0
4	Selectfluor®	207[a]
5		1.0

[a] Weighed in concentration. Due to moderate solubility, the concentration in solution is lower.

3.4.6.2 DOSY studies of photosensitization auxiliary fluorination using pure compounds at different concentrations and reaction mixtures with different substrate loadings and substrates

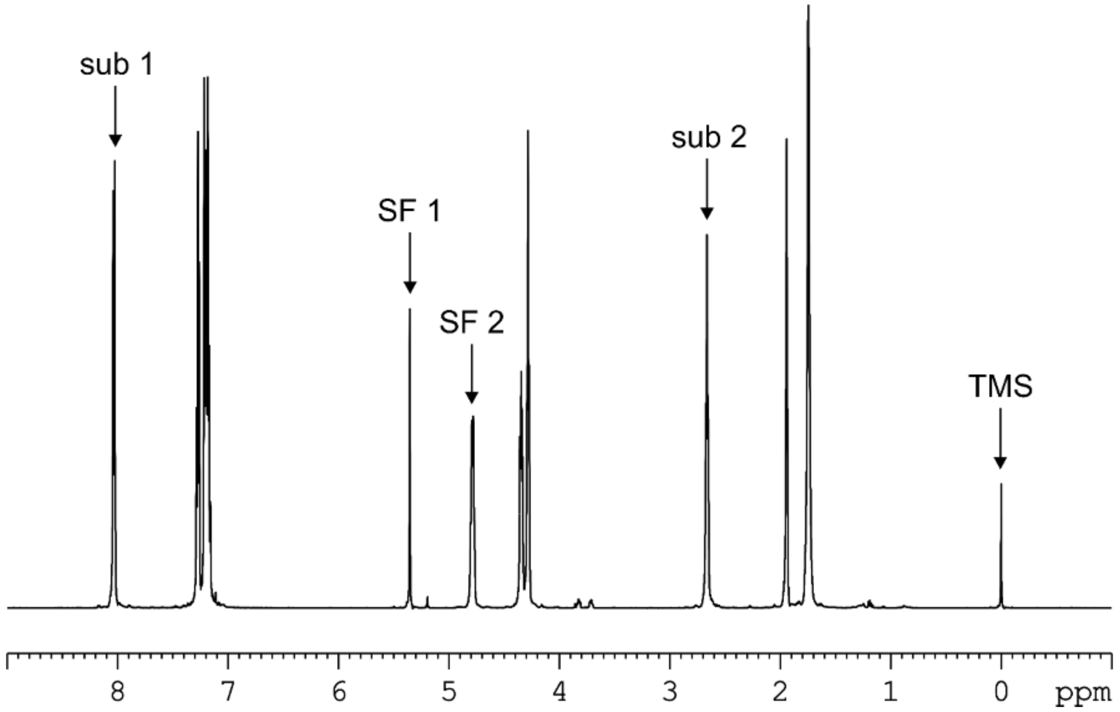

Figure S25. Representative ^{1}H-NMR spectrum of the photosensitization auxiliary fluorination of substrate **8b** measured in anhydrous and degassed CD_3CN at 25 °C. The volumes given in Tables S13 to S15 correspond to mean values with SD. In the investigations of both, the pure compounds (Table S13) and the reaction mixtures (Table S14 and S15), the intense and non-overlapping signals sub 1 and sub 2 were used for substrate **8b** and the signals **SF 1** and **SF 2** for Selectfluor®.

To obtain the volumes of the pure reagents at the concentrations present in the reaction, they were first examined as pure samples (Table S13).

Table S13: Mean diffusion coefficients and average volumes of substrate **8b** and Selectfluor® as pure compounds at different concentrations in anhydrous and degassed CD_3CN at 25 °C.

Entry	Compound	Concentration [mM]	Mean diffusion coefficient [E-9 m²/s] with SD	Average volume [Å³] with SD
1	substrate **8b**	621.0	6.48 ± 0.0291	431.4 ± 5.371
2		310.5	7.33 ± 0.0493	410.1 ± 7.44
3		1.0	8.52 ± 0.0375	390.0 ± 5.019
4	Selectfluor®	207[a]	5.03 ± 0.0128	952.2 ± 6.439
5		1.0	6.61 ± 0.104	714.4 ± 29.06

[a] Weighed in concentration. Due to moderate solubility, the concentration in solution is lower.

At the concentrations found in the standard reaction with 1.5 eq. substrate **8b**, the substrate **8b** has a volume of 410.1 ± 7.44 Å^3 (Table S13 entry 2) and Selectfluor® of 952.2 ± 6.439 Å^3 (Table S13 entry 4). At a concentration of 621 mM (Table S13 entry 1), which is present in the reaction with 3.0 eq, the volume of substrate **8b** increases slightly by 5.2%. If the concentration is significantly reduced to 1 mM (Table S13 entry 3), the volume diminishes by a similar amount to 390.0 ± 5.019 Å^3. When Selectfluor® is diluted to a concentration of 1 mM (Table S13 entry 5), the volume is also reduced, but by 25%. To check whether substrate **8b** and Selectfluor® are already present as monomers in the 1 mM samples, the monomer-volumes were calculated based on the known intermolecular van der Waals radii of the corresponding functional groups and atoms. [39,40] A volume of 247.23 Å^3 for substrate **8b** and 332.27 Å^3 for Selectfluor® were received. Since the calculated volumes, which are in general good approximations, differ significantly from the ones received by DOSY, this suggests that *the two reagents are still aggregated even at such low concentrations.*

Next, mixtures of substrate **8b** and Selectfluor® with 1.5 eq. substrate (Table S14) and 3.0 eq. (Table S15) were examined before and during the reaction.

Table S14: Mean diffusion coefficients and average volumes of substrate **8b** and Selectfluor® in a reaction mixture with 1.5 equivalents substrate **8b** at different times (reaction states) in anhydrous and degassed CD_3CN at 25 °C.

**Entry**	**Compound**	**Reaction status**	**Mean diffusion coefficient [E-9 m^2/s] with SD**	**Average volume [Å^3] with SD**
1	substrate **8b**	No illumination	6.74 ± 0.0131	423.8 ± 2.671
2		Illumination started	6.80 ± 0.0694	408.6 ± 10.63
3		Distinct product formation starts	6.81 ± 0.029	409.0 ± 4.151
4		Reaction finished	6.75 ± 0.047	408.5 ± 7.612
5	Selectfluor®	No illumination	5.13 ± 0.0713	845.4 ± 30.77
6		Illumination started	5.16 ± 0.0183	816.3 ± 7.574
7		Distinct product formation starts	5.15 ± 0.041	822.3 ± 17.09
8		Reaction finished	5.13 ± 0.0243	817.1 ± 10.15

Table S15: Mean diffusion coefficients and average volumes of substrate **8b** and Selectfluor® in a reaction mixture with 3.0 equivalents substrate **8b** at different times (reaction states) in anhydrous and degassed CD_3CN at 25 °C.

Entry	Compound	Reaction status	Mean diffusion coefficient [E-9 m²/s] with SD	Average volume [Å³] with SD
1	substrate **8b**	No illumination	6.07 ± 0.0244	428.6 ± 4.765
2		Illumination started	6.16 ± 0.0786	417.2 ± 13.71
3		Distinct product formation starts	6.17 ± 0.0284	429.2 ± 5.378
4		Reaction finished	6.11 ± 0.0633	427.5 ± 11.48
5	Selectfluor®	No illumination	4.64 ± 0.0498	846.0 ± 23.74
6		Illumination started	4.73 ± 0.0239	810.0 ± 10.70
7		Distinct product formation starts	4.70 ± 0.0439	852.3 ± 20.81
8		Reaction finished	4.67 ± 0.0352	839.2 ± 16.53

In order to determine whether there is a change in the volumes of the reagents involved during photosensitization auxiliary fluorination, their volume was identified for certain reaction states. It turns out, that in both reactions (1.5 eq substrate **8b**, Table S14 and 3.0 eq. substrate **8b**, Table S15), the volume of the two reagents , substrate **8b** and Selectfluor®, remains almost unchanged. The average volume of the substrate in the 1.5 equivalent reaction (412.5 ± 6.266 Å³) and 3.0 equivalent reaction (425.6 ± 8.833 Å³) do not differ from those, when substrate **8b** is present as a pure compound (see Table S13). The average volume of Selectfluor®, however, is reduced to 825.3 ± 16.40 Å³ for the 1.5 eq. reaction and 836.9 ± 17.95 Å³ for the 3.0 eq. reaction. This corresponds to a reduction of 13% (1.5 eq. reaction) respectively 12% (3.0 eq. reaction) compared to Selectfluor® as a pure compound of the same concentration (see Table S13 entry 4).

To check the origin of the Selectfluor® de-aggregation inside the reaction mixture with substrate **8b**, the unreactive substrate **5b** without the fluorine in *para*-position was selected.

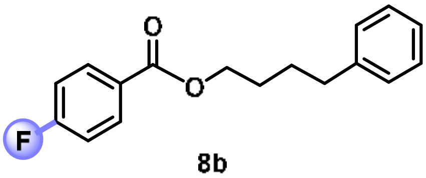

8b
= **'sub'**

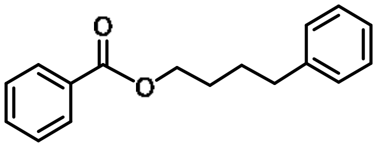

5b
= **'sub-H'**

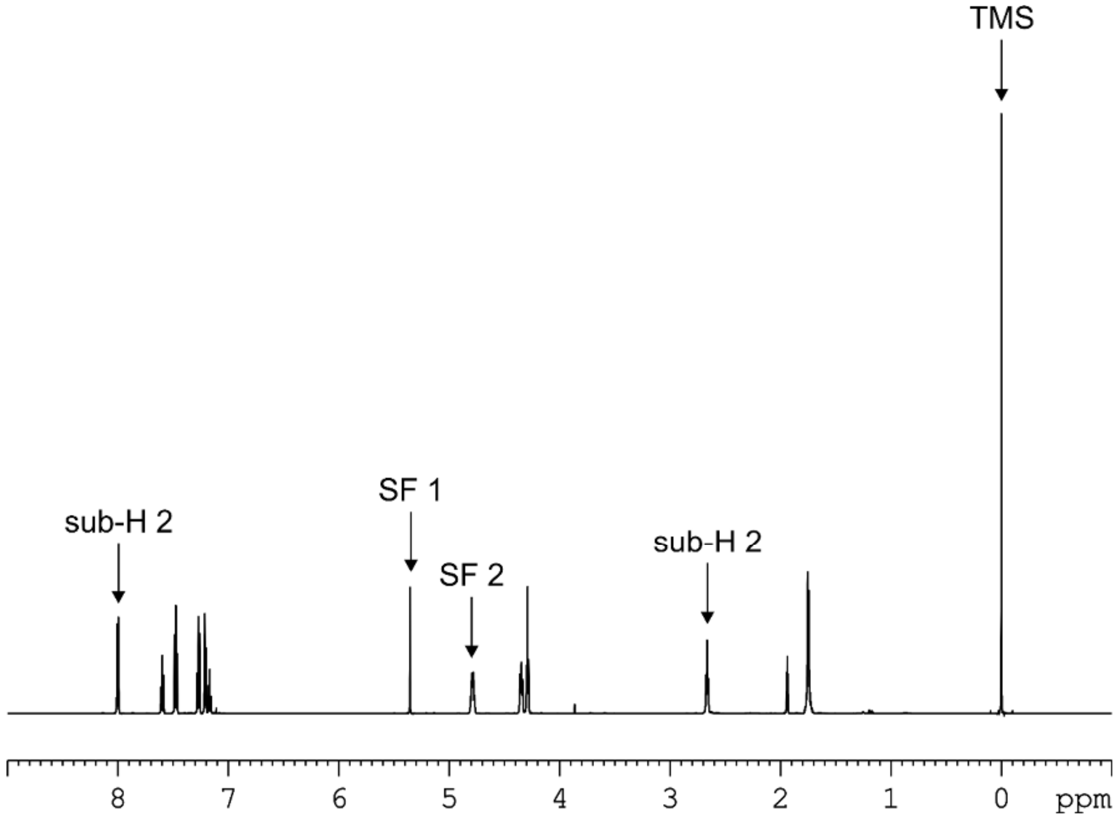

Figure S26. ^{1}H-NMR spectrum of substrate **5b** (1.5 eq., 310.5 mM) and Selectfluor® (1.0 eq., not completely dissolved) measured in anhydrous and degassed CD_3CN at 25 °C. The volumes given in Table S16 correspond to mean values with SD. For this, the intense and non-overlapping signals sub-H 1 and sub-H 2 were used for substrate **5b** and the signals **SF** 1 and **SF** 2 for Selectfluor®.

Table S16: Mean diffusion coefficients and average volumes of substrate **5b** and Selectfluor® in a mixture with 1.5 eq. substrate **5b** and 1.0 eq. Selectfluor® in anhydrous and degassed CD_3CN at 25 °C.

Entry	Compound	Concentration [mM]	Mean diffusion coefficient [E-9 m^2/s] with SD	Average volume [$Å^3$] with SD
1	substrate **5b**	310.5	6.78 ± 0.0839	413.9 ± 13.24
2	Selectfluor®	207[a]	4.95 ± 0.0181	917.5 ± 8.864

[a] Weighed in concentration. Due to moderate solubility, the concentration in solution is lower.

Similar to the previous investigations, a sample containing 1.5 eq. substrate **5b** and 1.0 eq. Selectfluor® was prepared. With 413.9 ± 13.24 $Å^3$, the volume of substrate **5b** is very similar to that of substrate **8b** (412.5 ± 6.266 $Å^3$, see Table S14). This is not surprising given that only the fluorine in the *para*-position has been replaced by a hydrogen atom. On the other hand, looking at the volume of Selectfluor® shows a clear effect of substrate **8b** compared to **5b**. While Selectfluor® mixed with 1.5 eq. **8b** was de-aggregated by 13% compared to its volume as pure compound with the same concentration (see Table S13 entry 4), its volume only decreases by 3.6% upon mixing with **5b**. *Considering the good reactivity of **8b** vs **5b**, it is suggested that the ability of the **PSAux** F atom to de-aggregate Selectfluor® may play a key role in initiating reactivity.*

3.4.6.3 DOSY plots

Pure compounds (Table S13)

entry 1 entry 2

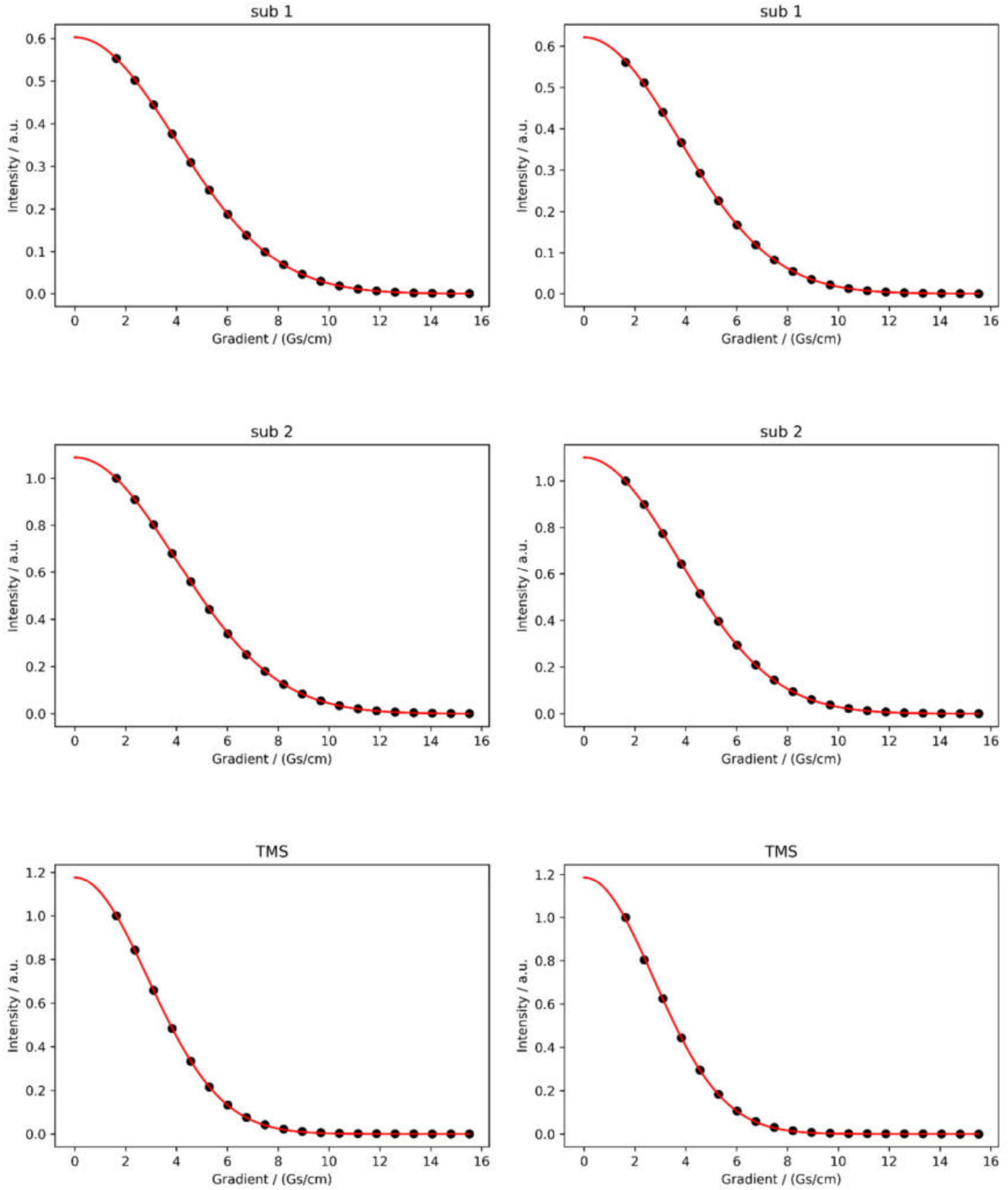

entry 3

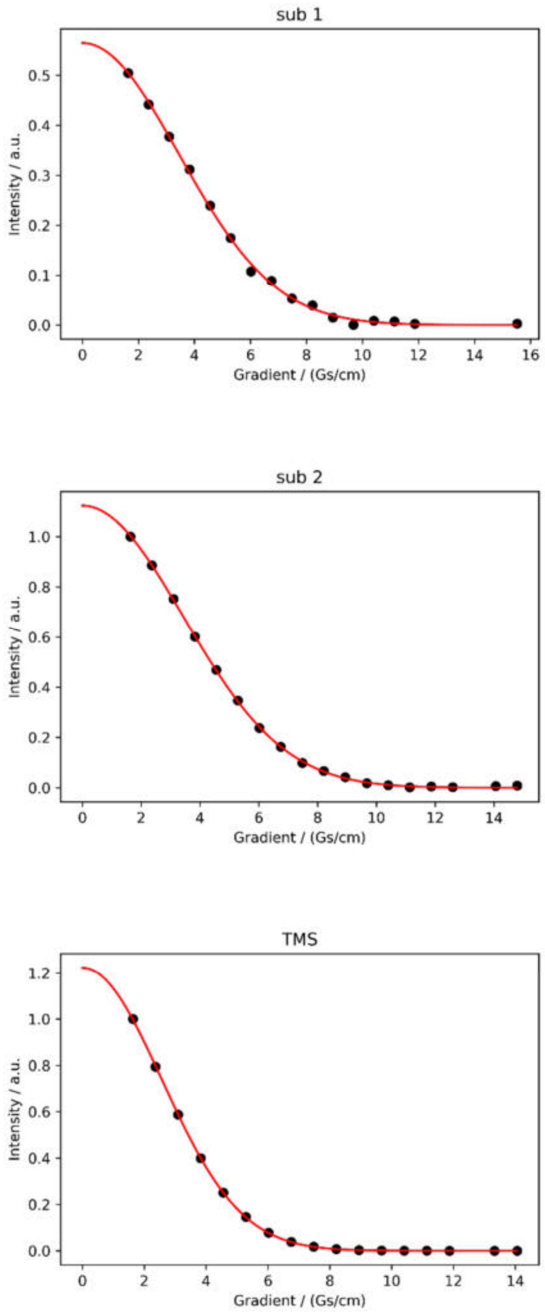

entry 4

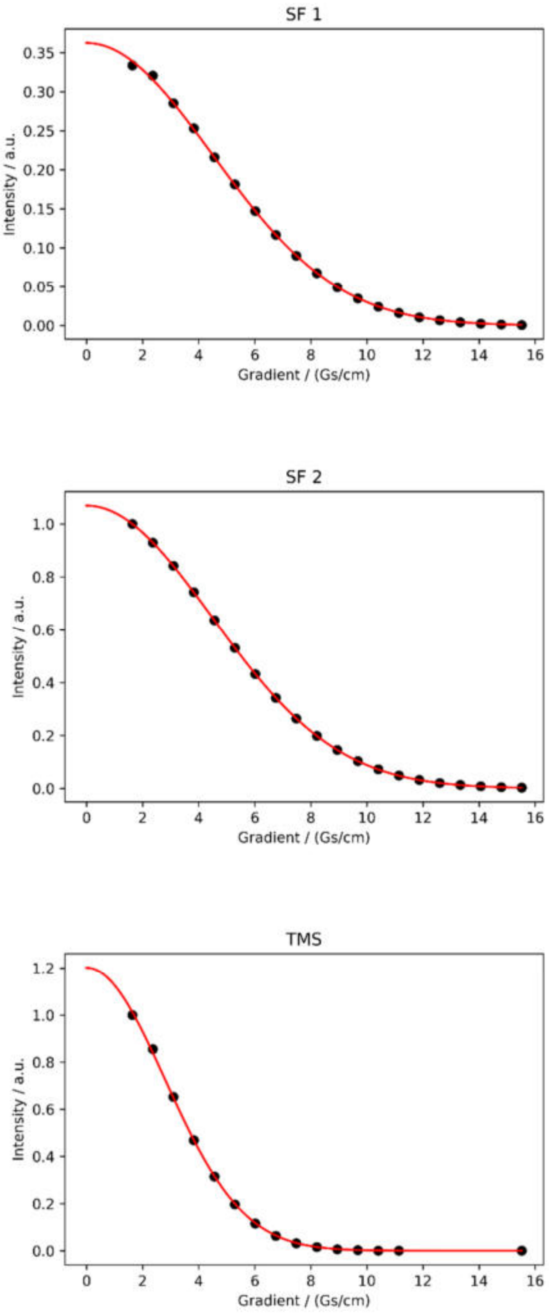

entry 5

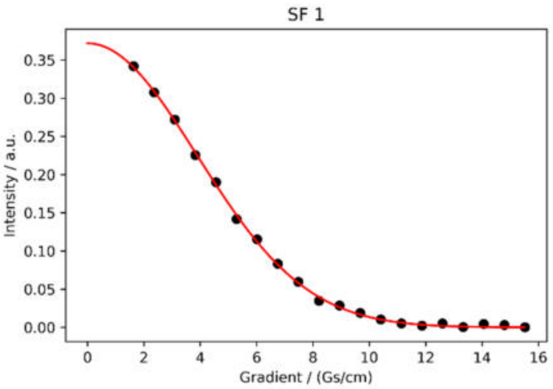

SF 1
Intensity / a.u.
0.35
0.30
0.25
0.20
0.15
0.10
0.05
0.00
0
2
4
6
8
10
12
14
16
Gradient / (Gs/cm)

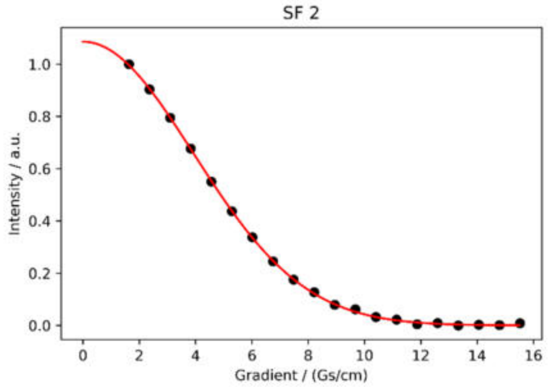

SF 2
Intensity / a.u.
1.0
0.8
0.6
0.4
0.2
0.0
0
2
4
6
8
10
12
14
16
Gradient / (Gs/cm)

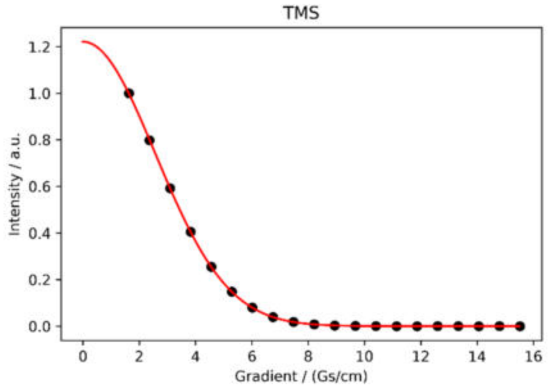

TMS
Intensity / a.u.
1.2
1.0
0.8
0.6
0.4
0.2
0.0
0
2
4
6
8
10
12
14
16
Gradient / (Gs/cm)

1.5 eq. reaction (Table S14)

entry 1

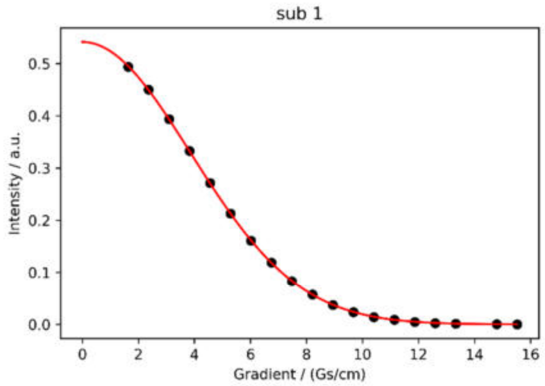

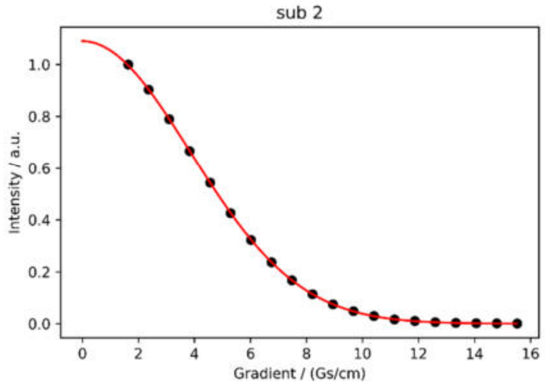

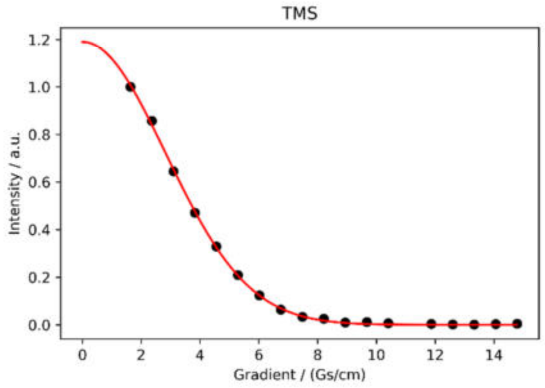

entry 2

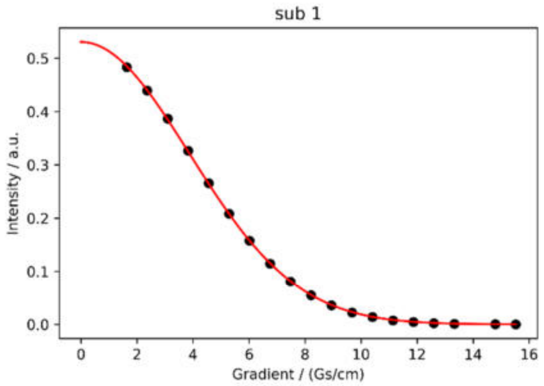

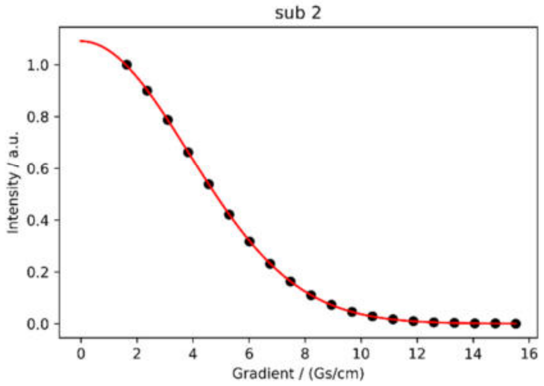

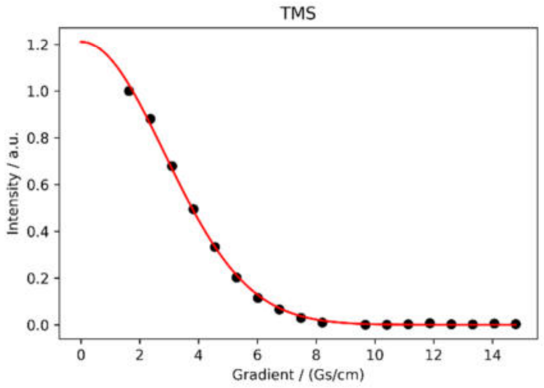

entry 3

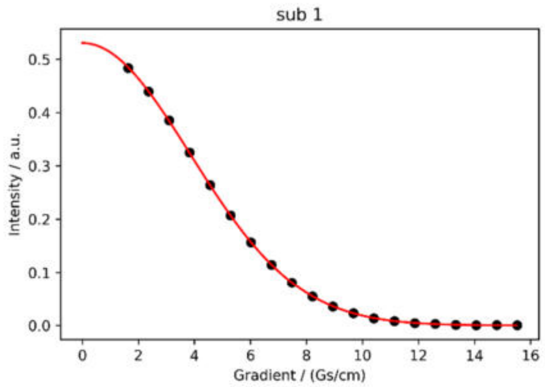

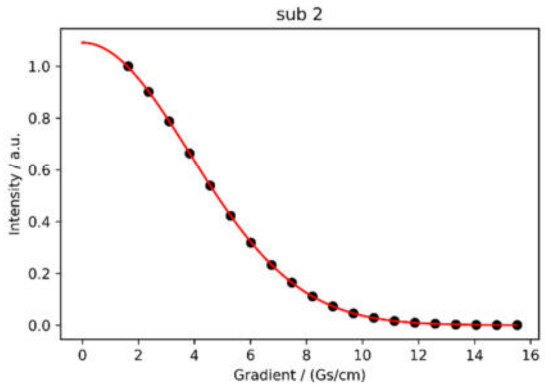

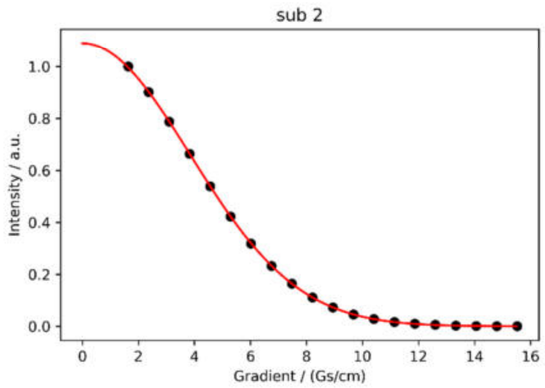

entry 4

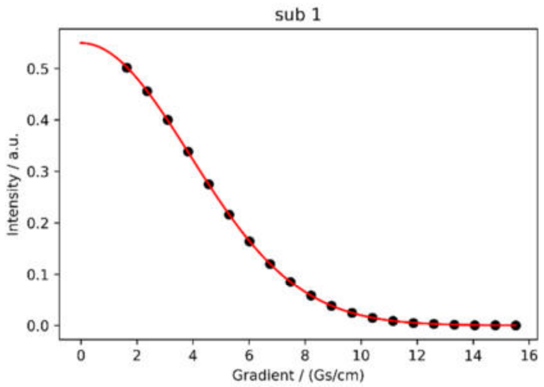

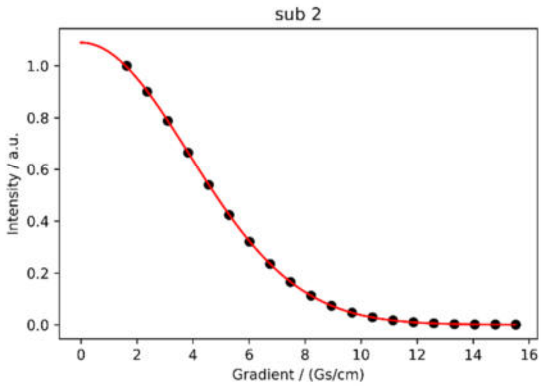

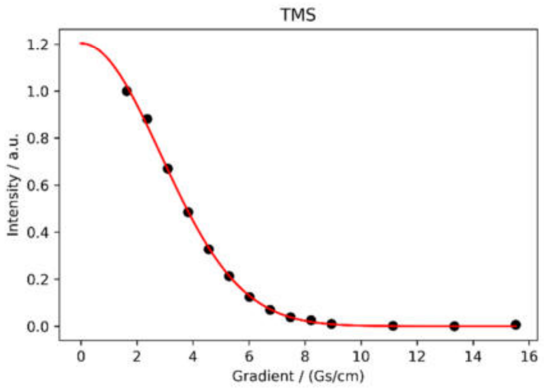

entry 5

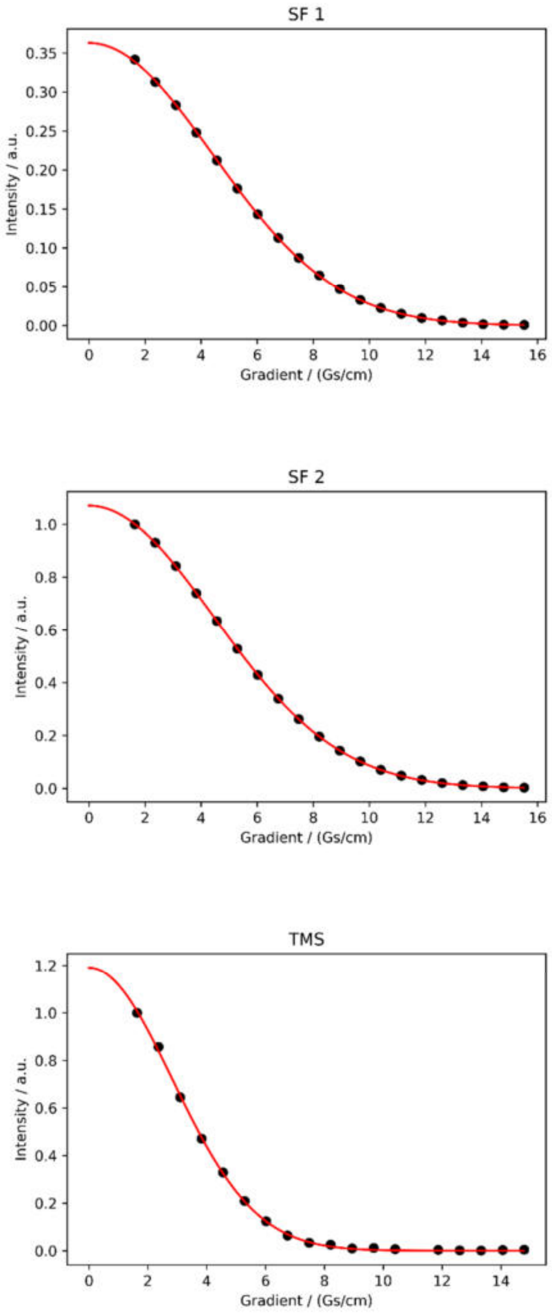

entry 6

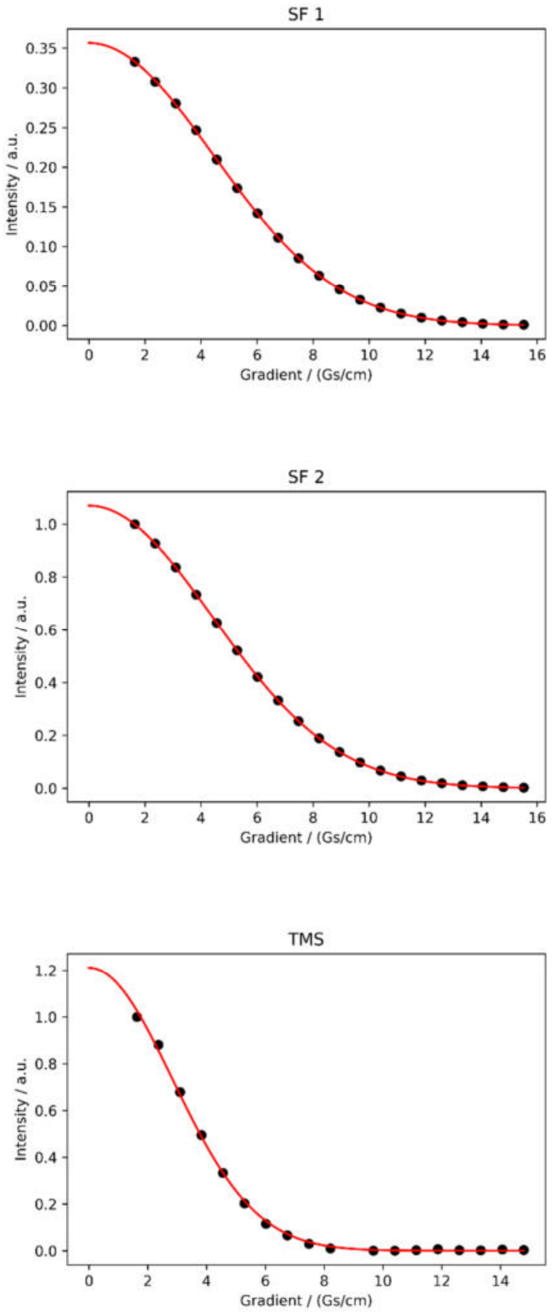

entry 7

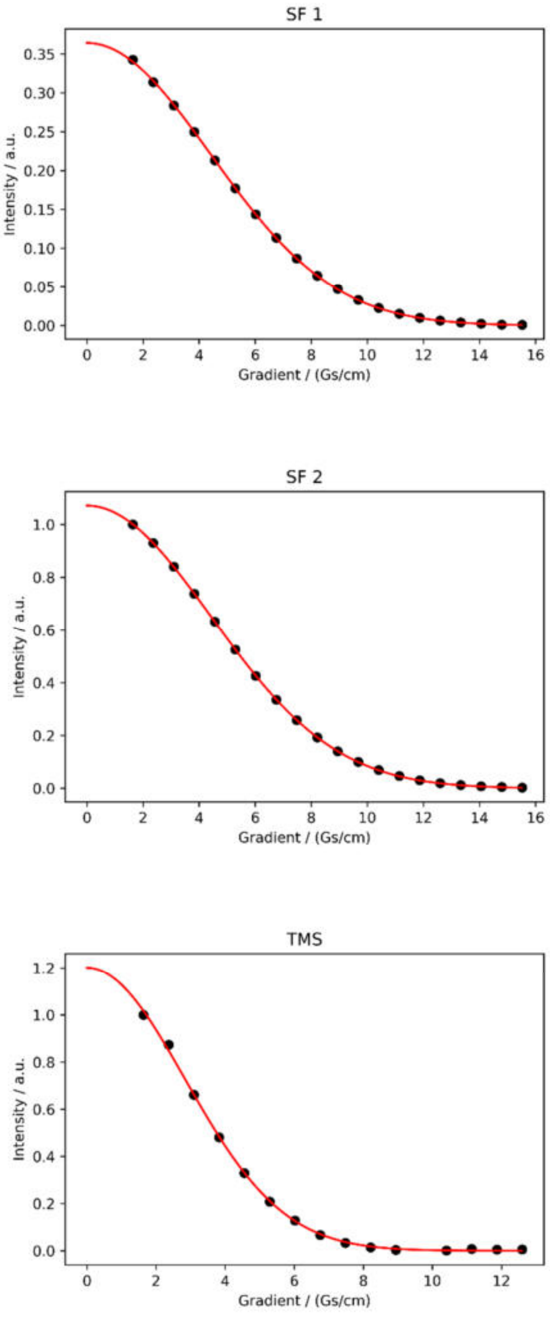

entry 8

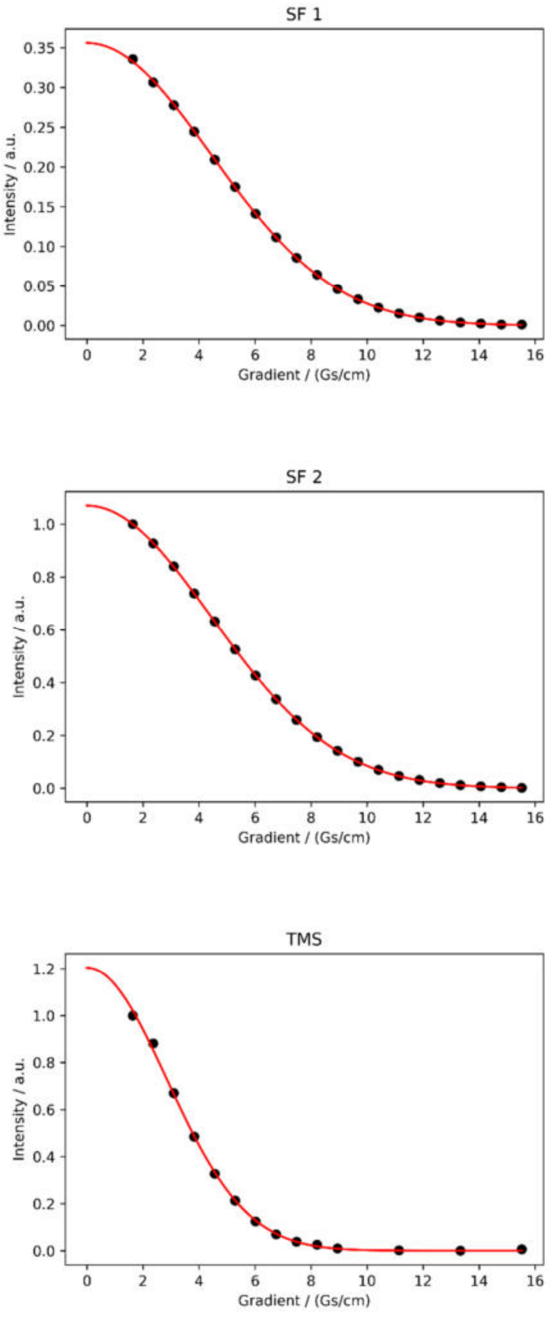

3.0 eq. reaction (Table S15)

entry 1

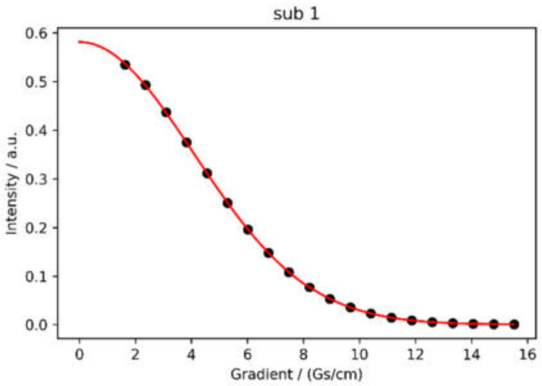

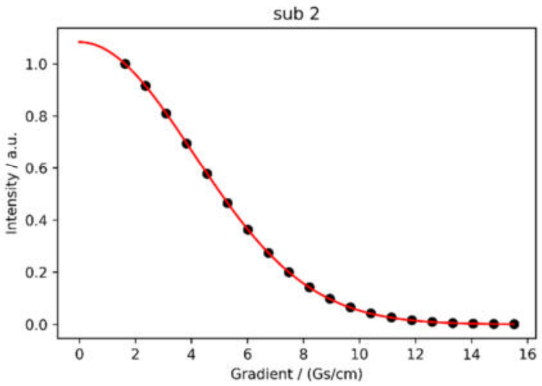

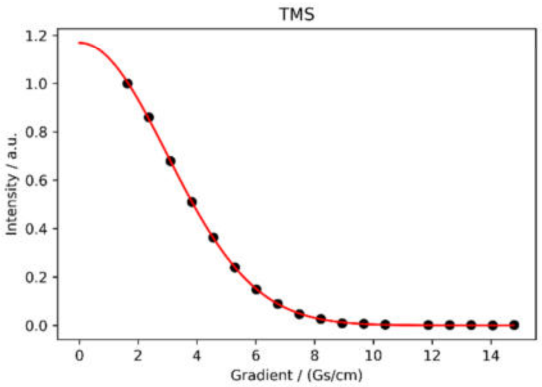

entry 2

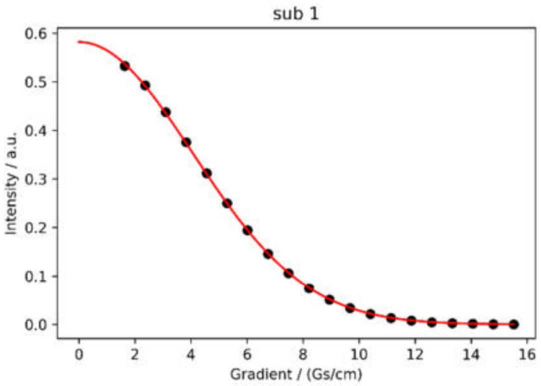

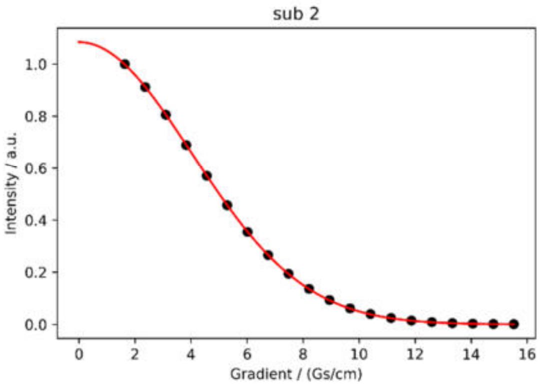

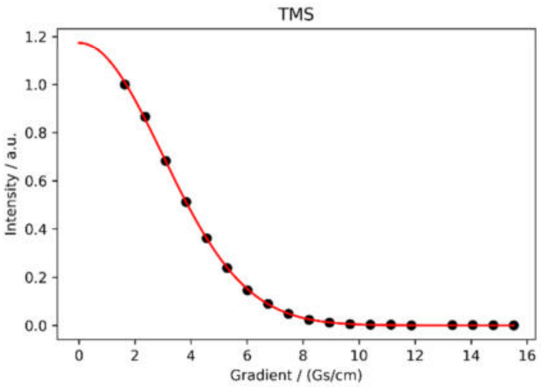

entry 3

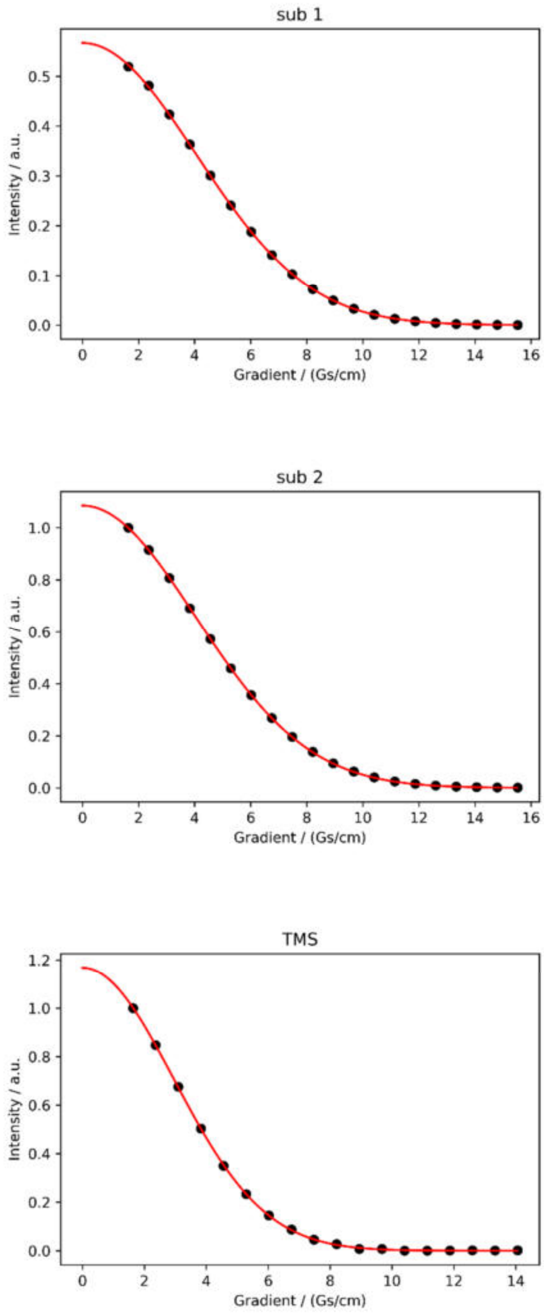

entry 4

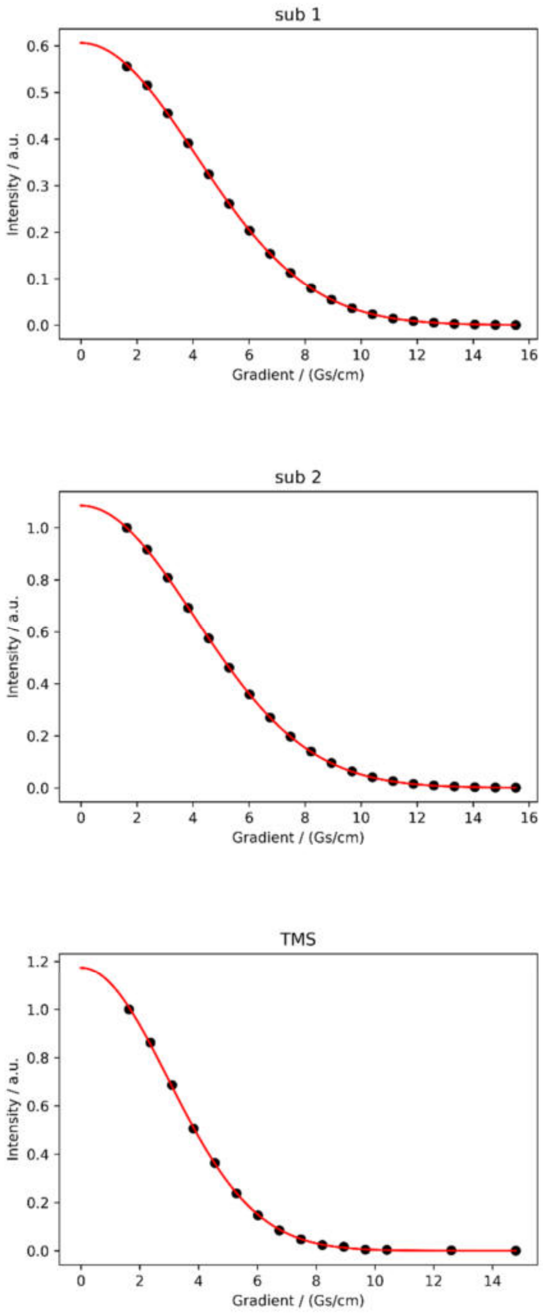

entry 5

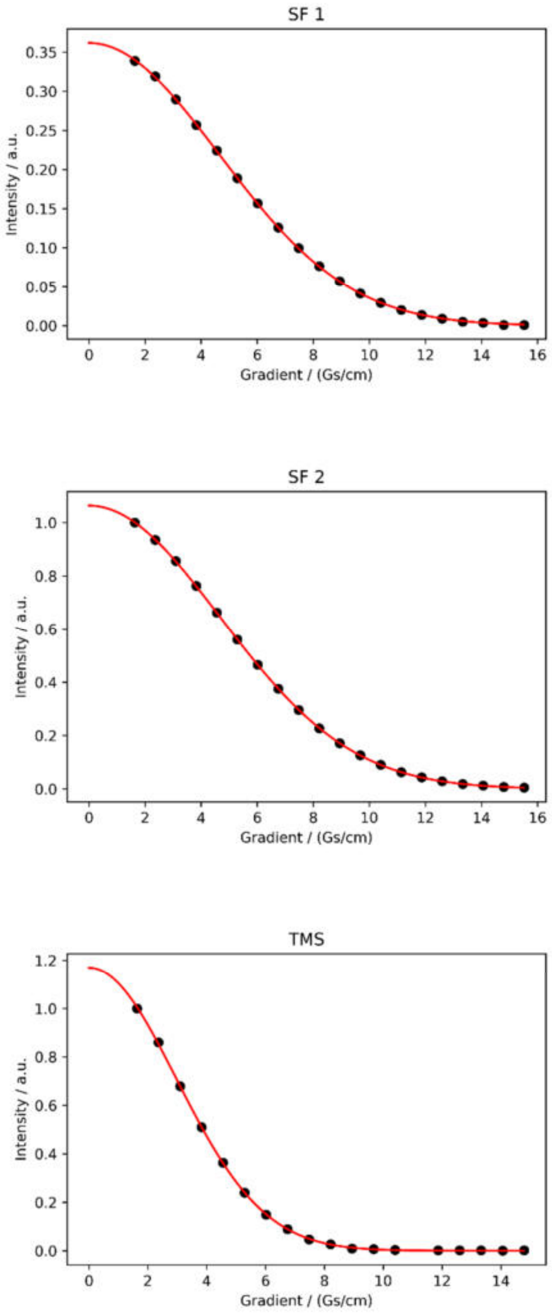

entry 6

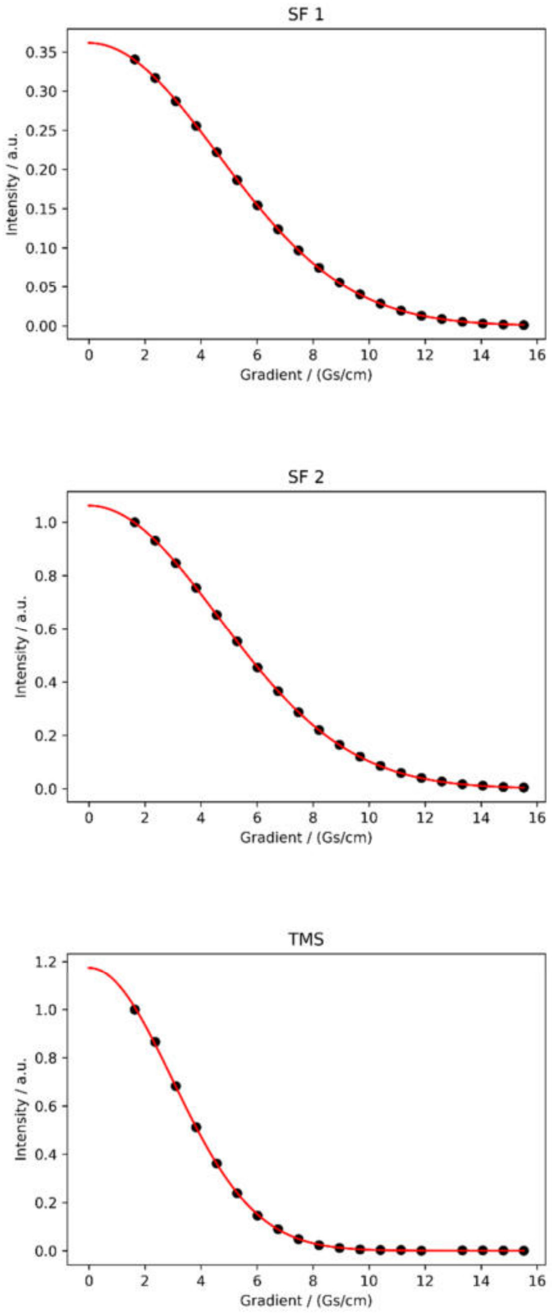

entry 7

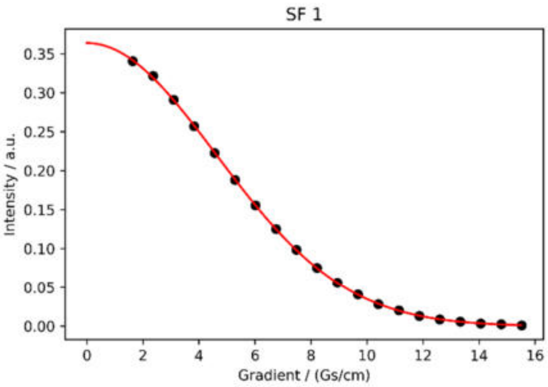

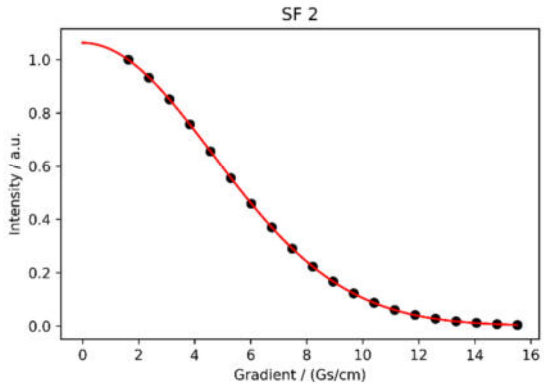

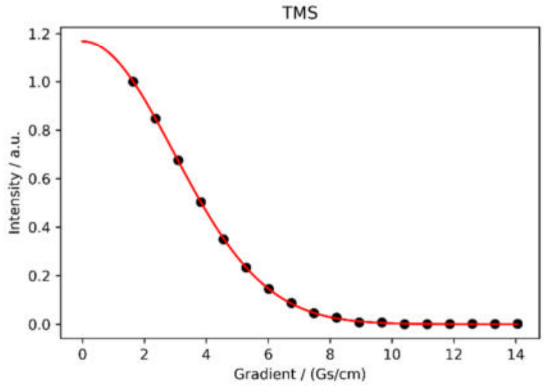

entry 8

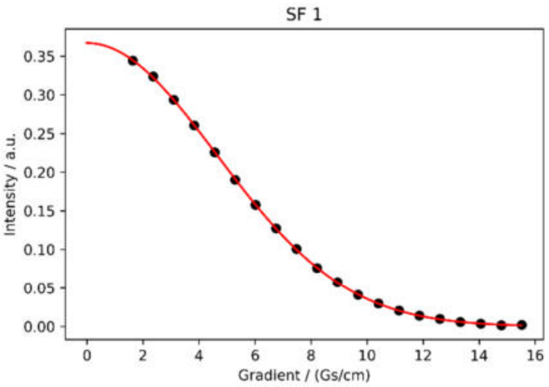

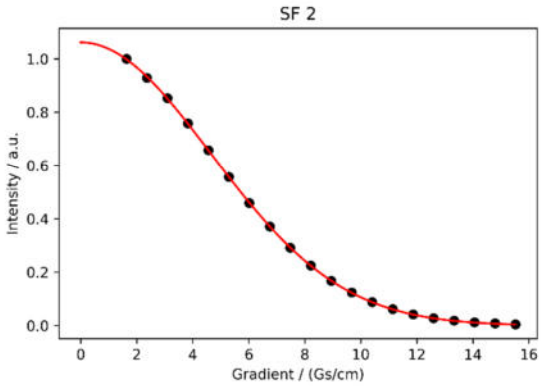

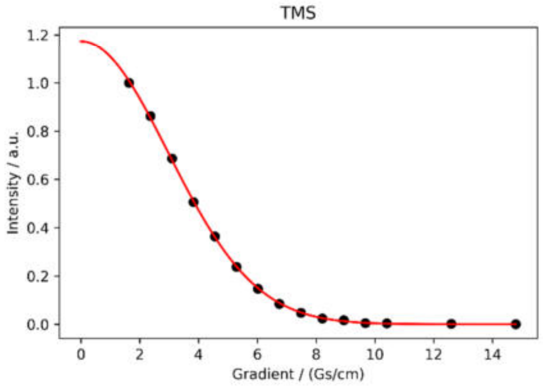

1.5 eq. substrate 5b + 1.0 eq. Selectfluor® (Table S16)

entry 1

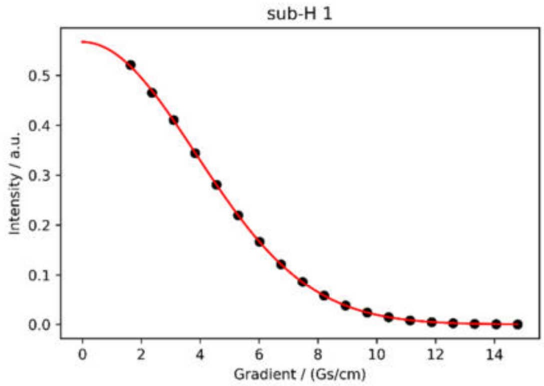

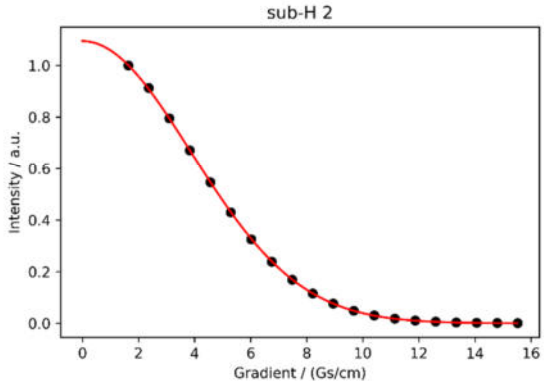

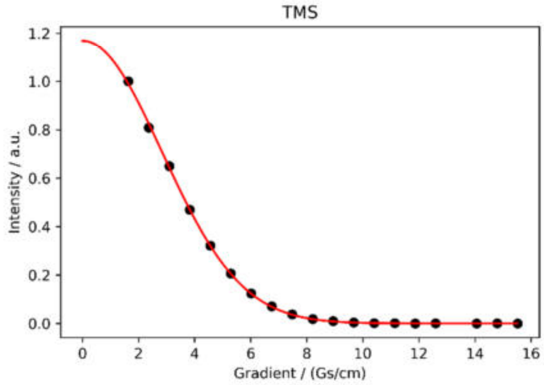

entry 2

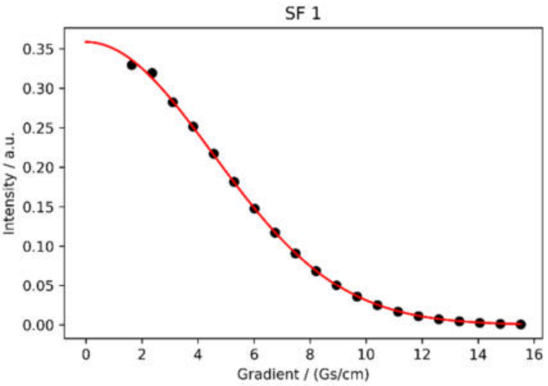

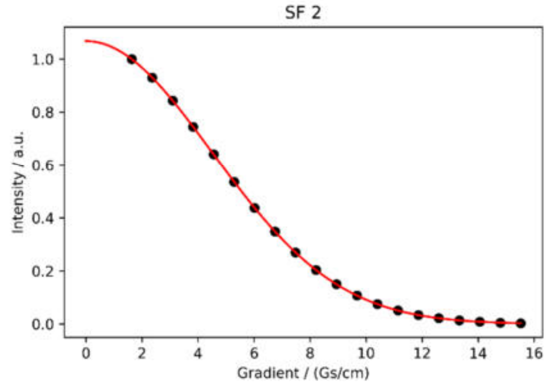

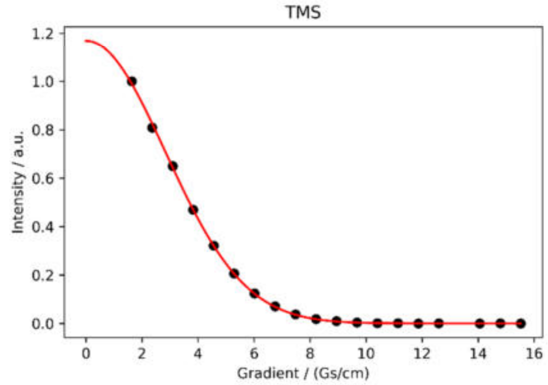

3.4.7 Structural elucidation of **2o** in $CDCl_3$ at 25 °C

To assign the signals for **2o** in $CDCl_3$ (saturated solution) at 25 °C (Figure S27) a series of 1D and 2D NMR experiments (1D-^{1}H; 1D-^{19}F{^{1}H}; 1D-^{13}C{^{1}H}; 2D-^{1}H, ^{1}H correlated spectroscopy (COSY); 2D-^{1}H, ^{1}H nuclear Overhauser enhancement spectroscopy (NOESY), 2D-^{1}H, ^{13}C heteronuclear multiple bond correlation (HMBC); 2D-^{1}H, ^{13}C heteronuclear single quantum coherence (HSQC)) was measured (Figure S28-S34). Next to the routine 1D-^{13}C{^{1}H} a 1D-^{13}C{^{1}H,^{19}F} with additional ^{19}F-decoupling was measured. By comparing the two NMR spectra the carbons next to the fluorine substituent can explicitly be identified and assigned *via* the associated ^{13}C-^{19}F coupling constants($^1J_{CF}$ – $^3J_{CF}$). (Figure S35).

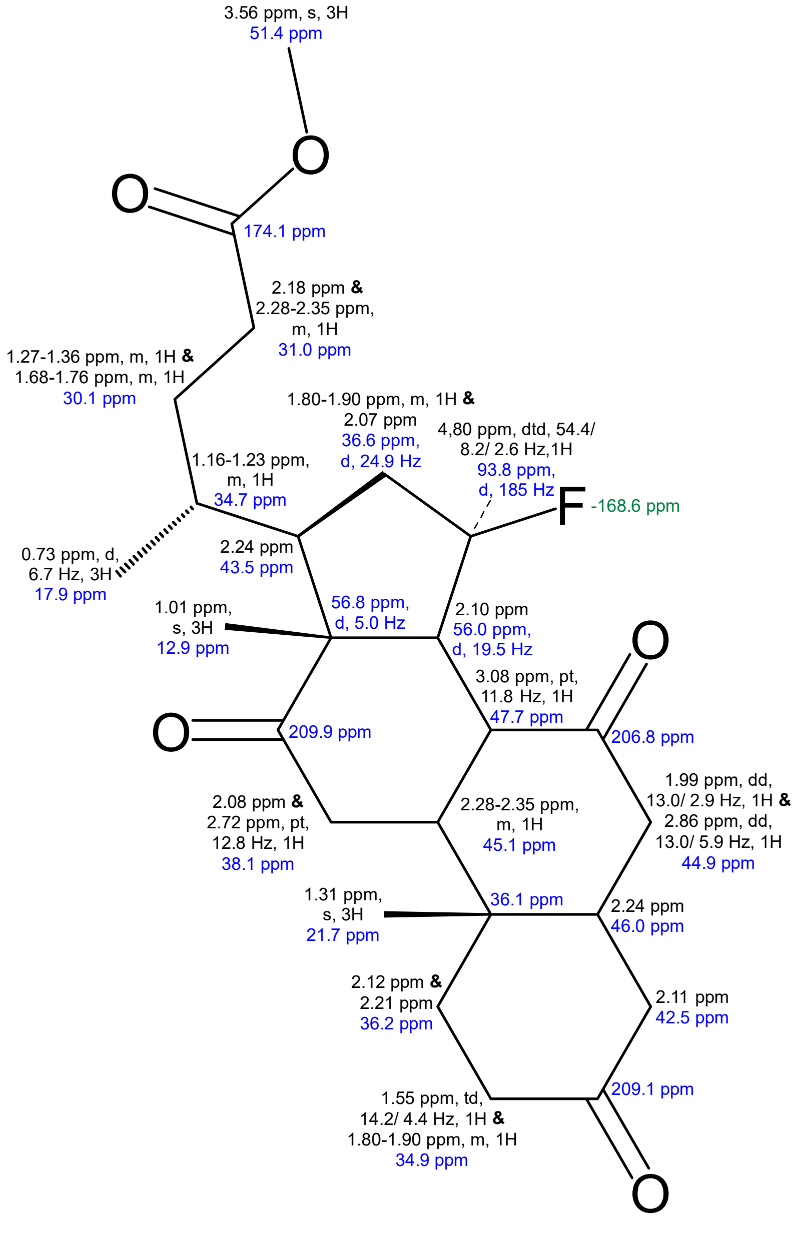

Figure S27. Assignment of **2o** in $CDCl_3$ (saturated solution) at 25 °C. ^{1}H chemical shift, multiplicity, coupling constants and integral are highlighted black, ^{13}C blue and ^{19}F green.

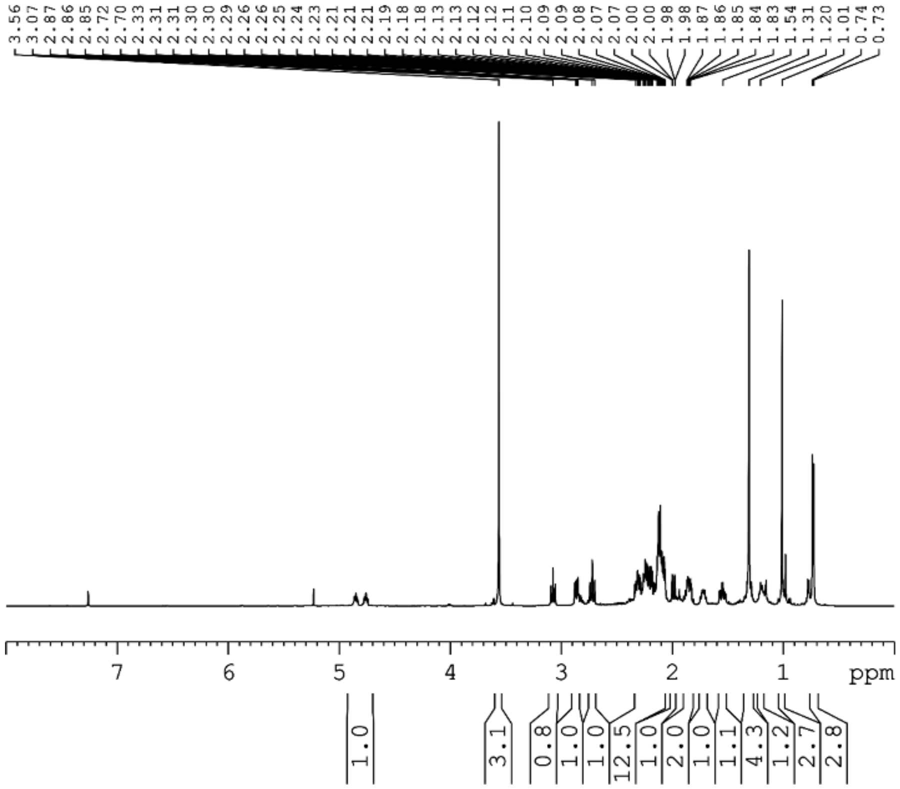

Figure S28: 1D-^{1}H spectrum (ns = 16) of **2o** in $CDCl_3$ (saturated solution) measured at 25 °C.

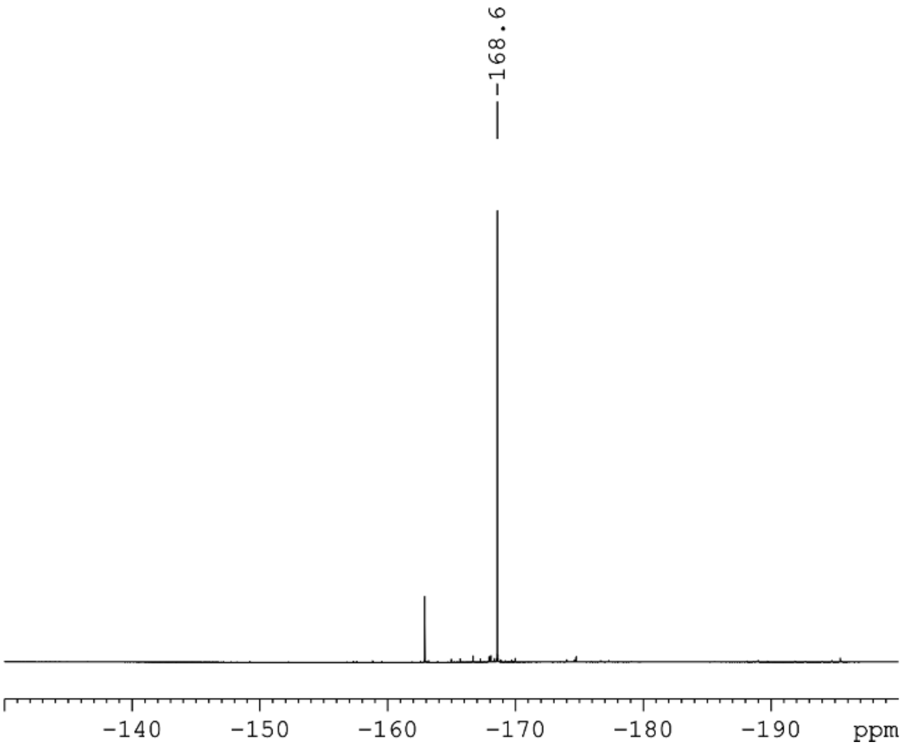

Figure S29. 1D-$^{19}F\{^{1}H\}$ spectrum (ns = 8) of **2o** in $CDCl_3$ (saturated solution) measured at 25 °C.

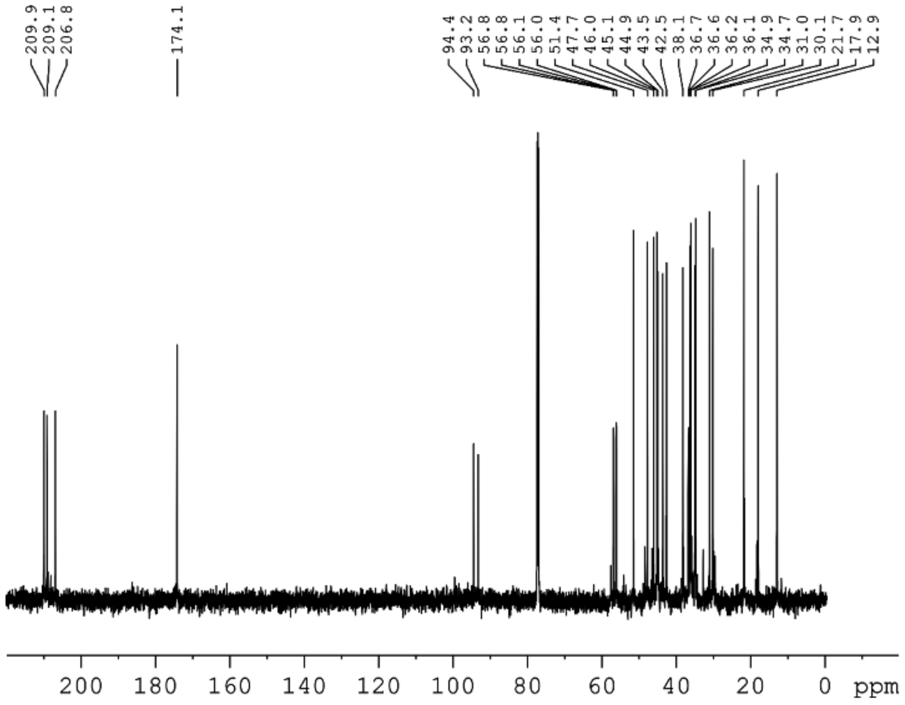

Figure S30. 1D-$^{13}C\{^1H\}$ spectrum (ns = 1024) of **2o** in $CDCl_3$ (saturated solution) measured at 25 °C.

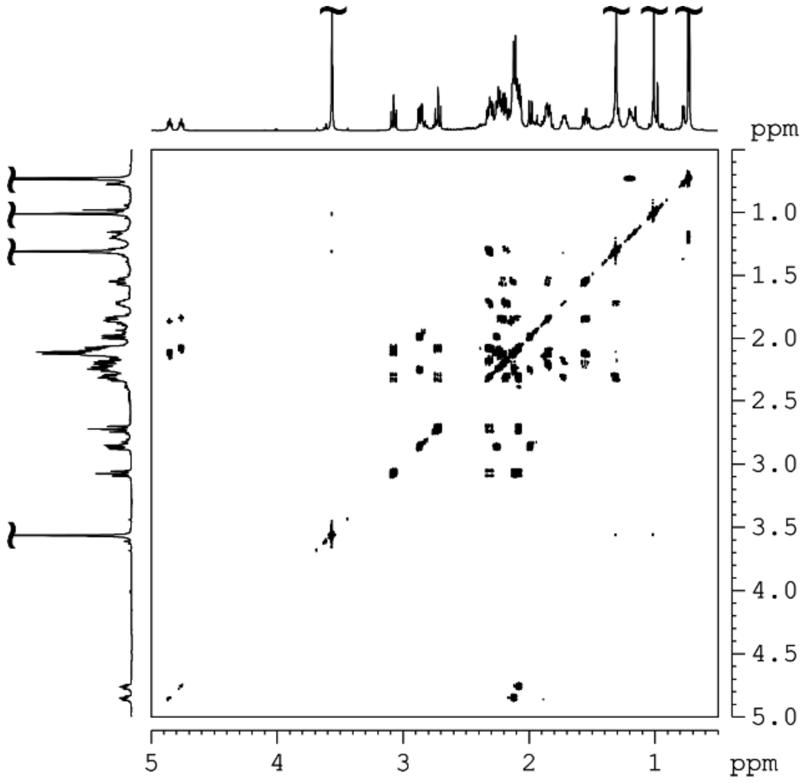

Figure S31. 2D-^1H, ^1H COSY spectrum of **2o** in $CDCl_3$ (saturated solution) measured at 25 °C.

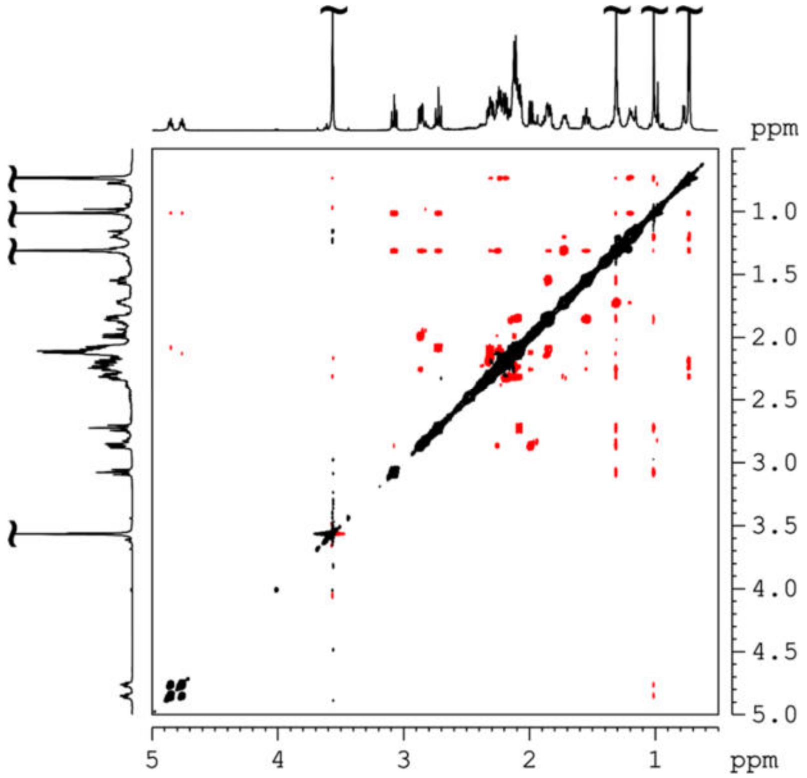

Figure S32. 2D-^{1}H, ^{1}H NOESY spectrum (mixing time = 0.7 s) of **2o** in $CDCl_3$ (saturated solution) measured at 25 °C.

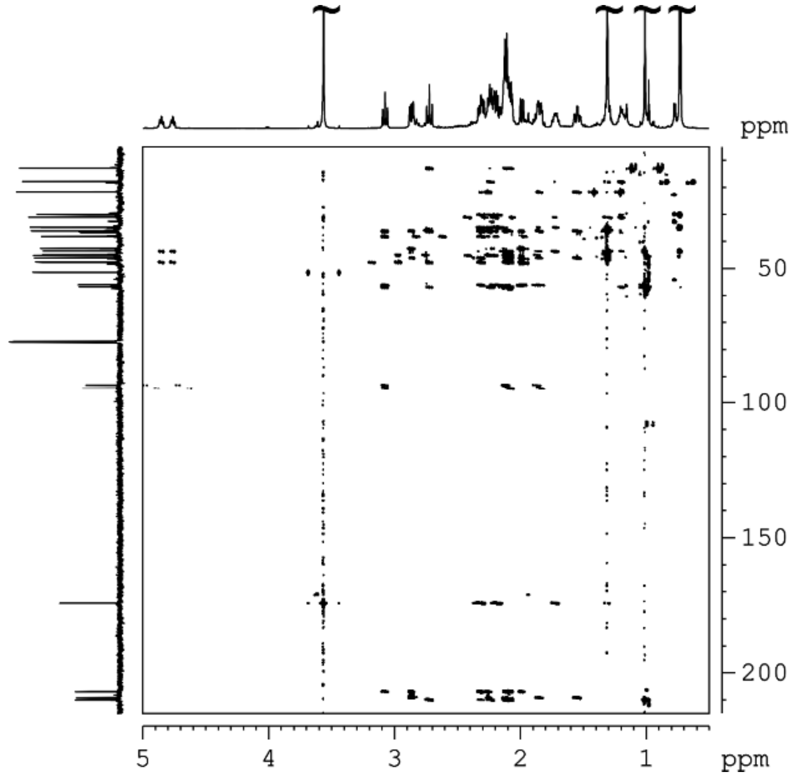

Figure S33. 2D-^{1}H, ^{13}C HMBC spectrum of **2o** in $CDCl_3$ (saturated solution) measured at 25 °C.

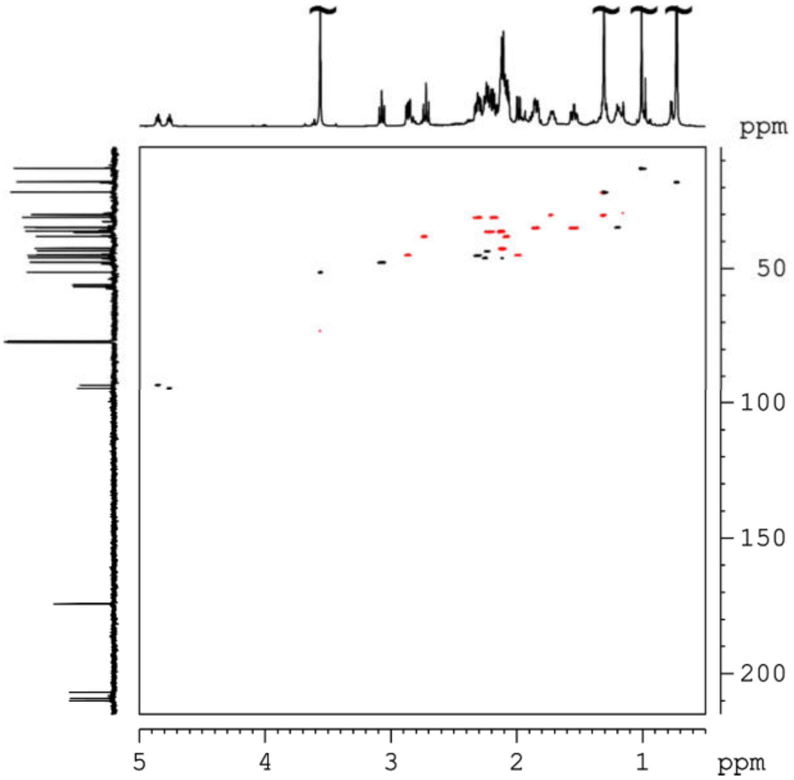

Figure S34. 2D-^{1}H, ^{13}C HSQC spectrum of **2o** in $CDCl_3$ (saturated solution) measured at 25 °C.

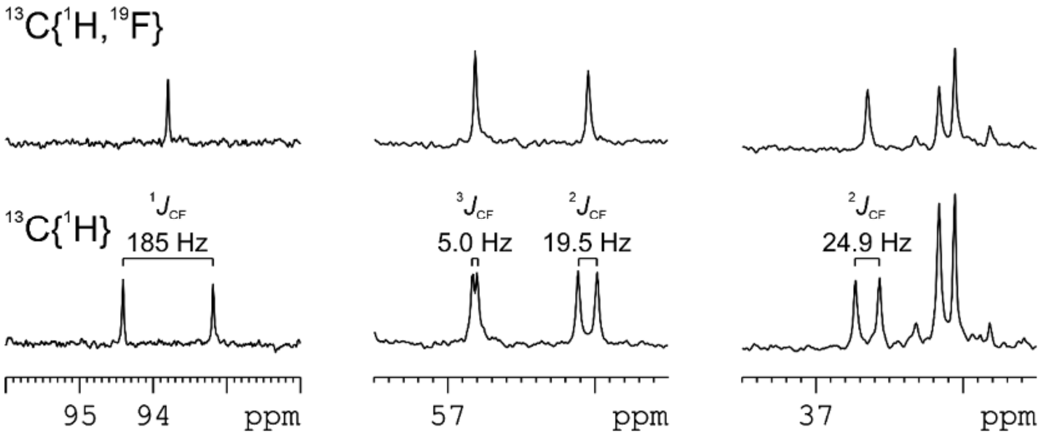

Figure S35. Sections of a routine 1D-^{13}C{^{1}H} spectrum (ns = 1024) (bottom) and a 1D-^{13}C{^{1}H,^{19}F} spectrum (ns = 1024) (top) with additional ^{19}F-decoupling. Because the adjacent ^{13}C signals merge to one signal with additional ^{19}F-decoupling, these signals must correspond to one carbon close to the fluorine substituent. The magnitude of *J*-coupling provides information on the number of bonds between in this case carbon and fluorine. In general, the smaller the coupling constant, the more bonds are between the coupled nuclei.

3.5 Density Functional Theory Computational Details and Cartesian Coordinates

Computations were performed using Density Functional Theory (DFT)[41] using the Gaussian09 software package.[42] Geometry optimizations were carried out using CAM-B3LYP[43], ωB97X-D[44a] or M06-2X [44b] functional with a 6-31+g(d,p) [45] basis set. Solvation was modeled implicitly using the Conductor-like Polarizable Continuum Model (CPCM) [46] in acetonitrile. For the triplet excited states, vertical excitation energy (from singlet ground state: S_0 to triplet excited state: T_1) was calculated from the optimized geometries using Time Dependent-Density Functional Theory (TD-DFT) [48] (code: "td=(triplet)") with their respective unrestricted functionals.

The summary for the calculated triplet energies of various sensitizers at various functionals, together with the T_1 values obtained from the literature (see Table 4, main manuscript) is shown below. As seen on chart, the functional that gave the closest values with the literature reports is CAM-B3LYP, hence the calculated T_1 value for the catalyst **MFB** is reported as 78.3 kcal mol^{-1}.

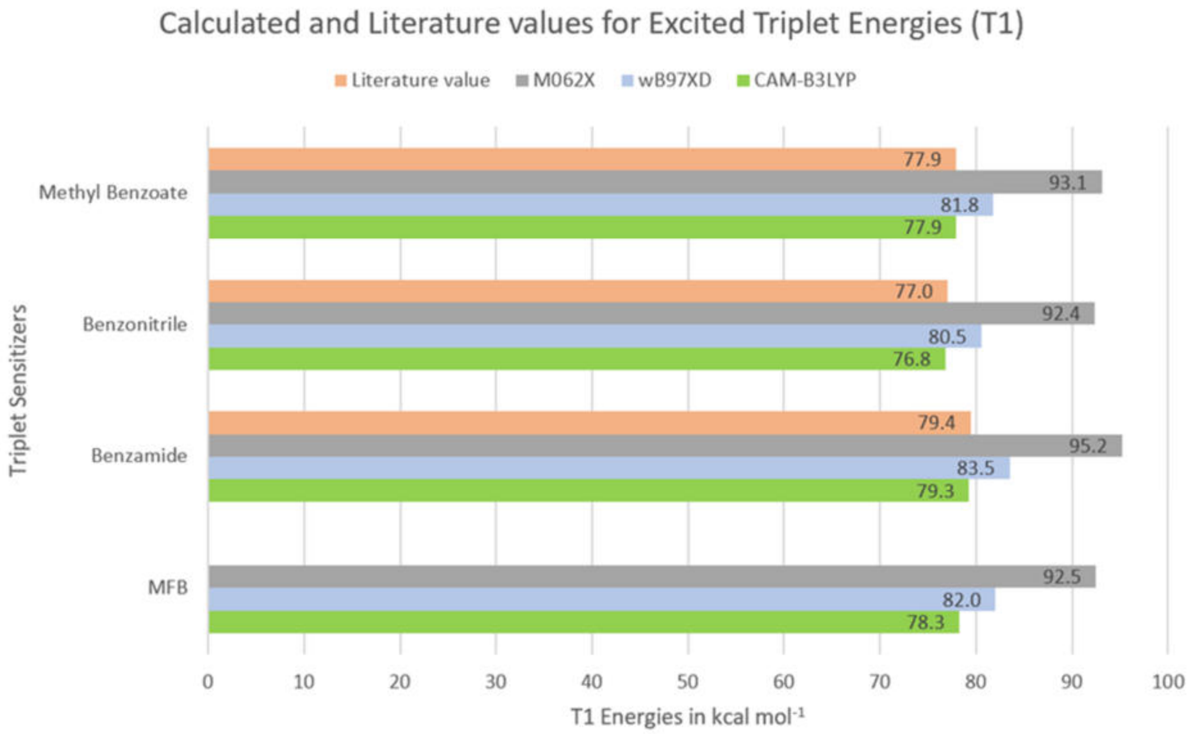

Figure S36. Summary of T_1 energies using various DFT functionals.

Summary of ground state cartesian coordinates and T_1 energies:

Methyl Benzoate:

CAM-B3LYP

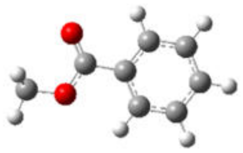

C	2.98737300	-0.28377200	-0.00003200
C	2.12179000	-1.37569200	-0.00001000
C	0.74591800	-1.17519700	0.00001800
C	0.23259100	0.12448000	0.00002700
C	1.10226100	1.21737200	0.00000400
C	2.47659700	1.01303200	-0.00002500
H	2.51938900	-2.38513100	-0.00001500
H	0.06922500	-2.02107100	0.00003500
H	0.68994600	2.22000000	0.00001000
H	3.14978900	1.86385000	-0.00004200
C	-1.23133900	0.39016100	0.00006100
C	-3.39216300	-0.55550000	-0.00004000
H	-3.70702200	-0.01189100	-0.89186700
H	-3.80533200	-1.56173400	-0.00012500
H	-3.70709300	-0.01201300	0.89183800
O	-1.72459700	1.50371600	0.00001800
O	-1.96638400	-0.72595200	0.00000700
H	4.06077400	-0.44342600	-0.00005400

TD-DFT T_1 = 3.3801 eV

ωB97XD

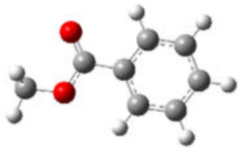

C	2.98962900	-0.28254000	-0.00006500
C	2.12420500	-1.37651900	-0.00000900
C	0.74666600	-1.17745600	0.00004700
C	0.23138300	0.12324700	0.00005200
C	1.10108800	1.21820400	-0.00000500
C	2.47699700	1.01495500	-0.00006300
H	2.52369600	-2.38540100	-0.00001000
H	0.07195900	-2.02572900	0.00009000
H	0.68908300	2.22155900	-0.00000100
H	3.15003900	1.86610300	-0.00010600
C	-1.23497200	0.39058200	0.00012000
C	-3.39094500	-0.55682300	-0.00007600
H	-3.70766000	-0.01426500	-0.89266800
H	-3.80372200	-1.56348000	-0.00023200
H	-3.70779000	-0.01448900	0.89260500
O	-1.72550200	1.50525900	0.00004400
O	-1.96739900	-0.72585100	0.00000900
H	4.06329800	-0.44144800	-0.00010900

TD-DFT T_1 = 3.5468 eV

M06-2X

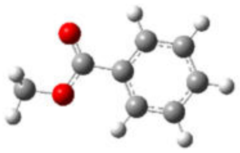

C	2.98888600	-0.27898700	-0.00005600
C	2.12570100	-1.37553300	-0.00001100
C	0.74667000	-1.17929400	0.00003800
C	0.23280400	0.12122600	0.00004500
C	1.09726900	1.21956100	-0.00000100
C	2.47435200	1.01869300	-0.00005200
H	2.52706100	-2.38372500	-0.00001300
H	0.06967400	-2.02628900	0.00007400
H	0.67925800	2.22097200	0.00000400
H	3.14554900	1.87124000	-0.00008700
C	-1.23477200	0.38633900	0.00010500
C	-3.38821200	-0.54925700	-0.00006700
H	-3.69551400	-0.00060400	-0.89199600
H	-3.81193800	-1.55098300	-0.00019700
H	-3.69563100	-0.00078800	0.89193500
O	-1.72585700	1.49784000	0.00003700
O	-1.96634600	-0.73163200	0.00000900
H	4.06297100	-0.43597100	-0.00009500

TD-DFT T_1 = 4.0364 eV

Benzonitrile

CAM-B3LYP

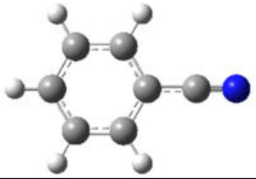

C	-1.47879200	-1.20884700	-0.00000100
C	-0.08974900	-1.21612900	0.00000600
C	0.60292000	-0.00004300	0.00000200
C	-0.08971400	1.21611500	0.00000300
C	-1.47871900	1.20889000	0.00000100
C	-2.17173000	0.00001900	-0.00000500
H	-2.02073300	-2.14816000	-0.00000400
H	0.45762300	-2.15189000	0.00000100
H	0.45775100	2.15182200	0.00000000
H	-2.02067000	2.14819800	0.00000100
H	-3.25664600	0.00007000	-0.00000500
C	2.03926700	-0.00002400	-0.00000200
N	3.19739600	0.00001000	-0.00000300

TD-DFT T_1 = 3.3299 eV

ωB97XD

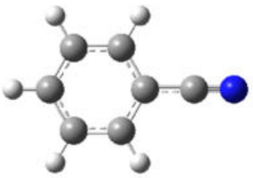

C	-1.48011600	-1.21024600	-0.00000100
C	-0.08945600	-1.21810000	0.00000700
C	0.60258700	-0.00004200	0.00000100
C	-0.08942200	1.21808600	0.00000300
C	-1.48004500	1.21028800	0.00000200
C	-2.17339300	0.00001900	-0.00000500
H	-2.02177500	-2.14981900	-0.00000500
H	0.45747400	-2.15442500	0.00000200
H	0.45759700	2.15436000	0.00000000
H	-2.02171200	2.14985600	0.00000100
H	-3.25847700	0.00007100	-0.00000400
C	2.04022300	-0.00002300	-0.00000200
N	3.20066000	0.00000900	-0.00000300

TD-DFT T_1 = 3.492 eV

M06-2X

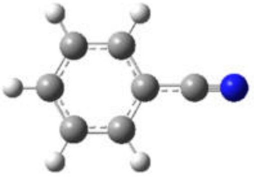

C	-1.48030100	-1.21105200	-0.00000100
C	-0.08902600	-1.21880000	0.00000200
C	0.60096200	-0.00004600	0.00000200
C	-0.08898200	1.21878000	0.00000100
C	-1.48022200	1.21110000	0.00000000
C	-2.17352800	0.00002400	-0.00000100
H	-2.02294600	-2.15023500	-0.00000100
H	0.46179200	-2.15317100	-0.00000200
H	0.46193400	2.15309300	-0.00000200
H	-2.02287200	2.15028000	0.00000100
H	-3.25870100	0.00007900	-0.00000200
C	2.04140000	-0.00002500	0.00000100
N	3.19985400	0.00001000	-0.00000200

TD-DFT T_1 = 4.0058 eV

Benzamide

CAM-B3LYP

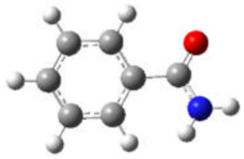

C	-2.57205100	-0.04583300	0.01843600
C	-1.84752000	-1.22285900	-0.15122200
C	-0.45640600	-1.19063800	-0.16502000
C	0.21903500	0.02346700	-0.01542200
C	-0.51328500	1.20226600	0.14075000
C	-1.90254200	1.16758400	0.16460500
H	-3.65682100	-0.07345700	0.03211600
H	-2.36468000	-2.16803100	-0.27867300
H	0.09038400	-2.11466100	-0.32138500
H	0.01956600	2.14050000	0.24641200
H	-2.46371900	2.08690700	0.29584600
C	1.71429000	0.12424300	-0.03597900
N	2.41130800	-0.98452300	0.29478800
H	3.41926900	-0.92834400	0.31337700
H	1.97628400	-1.79924000	0.69717300
O	2.28143100	1.17482500	-0.34815900

TD-DFT T_1 = 3.4376 eV

ωB97XD

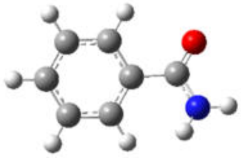

C	-2.57344900	-0.04651900	0.02057900
C	-1.84799100	-1.22117400	-0.17145800
C	-0.45526600	-1.18766200	-0.18704800
C	0.21948500	0.02571800	-0.01723000
C	-0.51327700	1.20313800	0.15885500
C	-1.90412300	1.16619800	0.18570900
H	-3.65835700	-0.07540800	0.03684300
H	-2.36424900	-2.16471300	-0.31466500
H	0.09278500	-2.10856200	-0.36101600
H	0.01615200	2.14236200	0.27916200
H	-2.46471400	2.08348900	0.33361800
C	1.71719200	0.12393600	-0.04099400
N	2.40647700	-0.97600300	0.33856500
H	3.41380900	-0.92540700	0.36236500
H	1.96541500	-1.76049600	0.79061700
O	2.28730000	1.15736800	-0.39841900

TD-DFT T_1 = 3.6201 eV

M06-2X

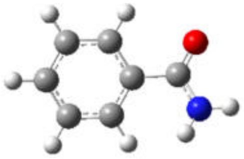

C	-2.57243800	-0.04637500	0.01998700
C	-1.84702600	-1.22469500	-0.15397700
C	-0.45374100	-1.19194000	-0.16944500
C	0.21894500	0.02452000	-0.01659600
C	-0.51166500	1.20540000	0.14202600
C	-1.90314400	1.16947900	0.16761800
H	-3.65740100	-0.07463900	0.03532400
H	-2.36404800	-2.16983600	-0.28397500
H	0.09559300	-2.11473400	-0.33100100
H	0.02353800	2.14347900	0.24857700
H	-2.46499400	2.08835900	0.30126300
C	1.71677800	0.12637000	-0.03695300
N	2.40637000	-0.98749400	0.29843600
H	3.41431300	-0.93269000	0.33047400
H	1.96453100	-1.78665800	0.72489400
O	2.28220300	1.17282800	-0.35382100

TD-DFT T_1 = 4.1273 eV

MFB

CAM-B3LYP

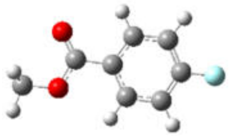

C	2.52642400	-0.13412500	-0.00002700
C	1.73817100	-1.27233500	-0.00000400
C	0.35824800	-1.11439500	0.00002700
C	-0.20477000	0.16493200	0.00003700
C	0.62334100	1.28997100	0.00001200
C	2.00414900	1.14843600	-0.00002100
H	2.19918200	-2.25303600	-0.00001100
H	-0.28347700	-1.98668400	0.00004600
H	0.17550200	2.27685300	0.00001900
H	2.66630800	2.00626300	-0.00004000
C	-1.67594400	0.37491700	0.00007600
C	-3.79782800	-0.65479700	-0.00003900
H	-4.13342800	-0.12418400	-0.89204800
H	-4.17049900	-1.67665400	-0.00012300
H	-4.13350300	-0.12430400	0.89201300
F	3.87255900	-0.28229300	-0.00006000
O	-2.20934000	1.46964700	0.00002300
O	-2.36614200	-0.76880100	0.00001600

TD-DFT T_1 = 3.3936 eV

ωB97XD

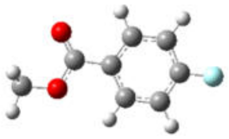

C	2.53010800	-0.13464300	0.00000800
C	1.73908400	-1.27398600	0.00000200
C	0.35799900	-1.11533800	-0.00000900
C	-0.20603500	0.16515800	-0.00001300
C	0.62323000	1.29126200	-0.00000400
C	2.00517700	1.14935600	0.00000600
H	2.19830300	-2.25546700	0.00000600
H	-0.28226700	-1.98938800	-0.00001500
H	0.17715300	2.27936900	-0.00000600
H	2.66618200	2.00815400	0.00001200
C	-1.67975900	0.37681700	-0.00002600
C	-3.79593700	-0.65788200	0.00000000
H	-4.13492400	-0.12952800	-0.89285100
H	-4.16527300	-1.68127300	-0.00006500
H	-4.13490800	-0.12964800	0.89292800
F	3.87264700	-0.28220900	0.00002000
O	-2.21135700	1.47223500	0.00002300
O	-2.36630500	-0.76808500	-0.00001800

TD-DFT T_1 = 3.5565 eV

M06-2X

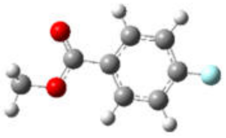

C	2.52871600	-0.13219400	-0.00000900
C	1.74099000	-1.27445900	0.00000600
C	0.35855200	-1.11938200	0.00002100
C	-0.20412600	0.16111300	0.00000900
C	0.61971300	1.29073200	-0.00000400
C	2.00273800	1.15201500	-0.00000700
H	2.20639800	-2.25352400	0.00000400
H	-0.28349600	-1.99262400	0.00003400
H	0.16726400	2.27667100	-0.00001200
H	2.66502500	2.01004500	-0.00001100
C	-1.67861600	0.37154000	0.00000400
C	-3.79420300	-0.64691900	-0.00001200
H	-4.12244800	-0.11087500	-0.89212300
H	-4.17804000	-1.66447100	-0.00013100
H	-4.12247100	-0.11108100	0.89221700
F	3.86980300	-0.27737000	-0.00000500
O	-2.20936700	1.46453300	0.00001700
O	-2.36601200	-0.77359500	-0.00001500

TD-DFT T_1 = 4.0100 eV

The nature interaction of the interaction between SelectFluor® (**SF**) and benzoates in the ground state was explored computationally using ωB97X-D[44]/6-31++g(2d,p)[45] level of theory with CPCM = acetonitrile solvation model was used). To probe the nature of non-covalent interactions and interacting orbitals between **MFB**-**SF** complex, NBO calculations and Second Order Perturbation theory analysis was carried out. This provides the interaction energies (E2) between donor and acceptor orbitals of the given system.

Cartesian coordinates of the optimized structures:

SelectFluor:

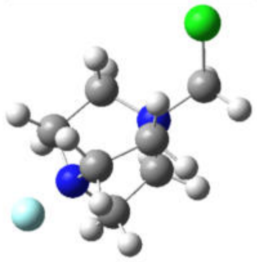

functional: ωB97X-D/6-31++g(2d,p)
Charge = 2, Multiplicity = 1

F	3.18349800	-0.54938600	0.00407800
C	1.26041800	-0.64456000	-1.29798100
C	1.18936200	-0.91111400	1.14031500
C	1.74703300	1.27006300	0.15449500
C	-0.26728400	-0.51034800	-1.15887900
H	1.55356000	-1.67854600	-1.46761500
H	1.67853300	-0.00658300	-2.07371200
C	-0.21173800	-0.29431900	1.28525300
H	1.79214000	-0.74119100	2.03014200
H	1.16722700	-1.97292000	0.90170100
H	2.06110100	1.51887000	1.16680600
H	2.42143900	1.72379600	-0.56907800
C	0.28209800	1.64198900	-0.13307300
H	-0.74018800	-1.46878300	-0.95761800
H	-0.69219400	-0.08266100	-2.06543500
H	-0.25392100	0.44535100	2.08363400
H	-0.94838000	-1.07222400	1.47575000
H	-0.06280000	2.38601200	0.58314800
H	0.14851000	2.02656900	-1.14296700
N	1.85216700	-0.21067200	0.00134700
N	-0.57464200	0.40501600	0.00010300
C	-2.01594500	0.84690200	0.00972800
H	-2.17430200	1.42340700	0.91844000
H	-2.18473100	1.45330800	-0.87756900
Cl	-3.13316600	-0.51541300	-0.00303700

G = −943.964696 hartrees

SelectFluor / methyl 4-fluorobenzoate complex :

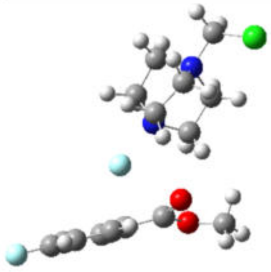

functional: ωB97X-D/6-31++g(2d,p)
Charge = 2, Multiplicity = 1

C	2.93547800	0.71411400	0.18006000
C	3.46956300	0.46859500	1.40975700
H	3.05714000	0.94384000	2.29136200
C	4.55909900	-0.42058700	1.51582800
C	5.10645500	-1.06751100	0.37839600
C	4.57869400	-0.82642200	-0.85513500
H	4.98036600	-0.62842300	2.49326800
H	4.96605900	-1.29052300	-1.75361900
O	0.99790600	1.81802900	0.92581900
F	1.04305300	-0.83138500	-0.97329000
C	-1.04203800	-0.82052800	0.89516400
C	-1.44369200	0.51993300	-1.06021800
C	-1.42265100	-1.88125700	-1.23123000
C	-2.54548900	-0.63224100	1.20704900
H	-0.44434200	-0.04676900	1.37248400
H	-0.67595900	-1.79302600	1.21636800
C	-2.98278800	0.39500100	-0.98824700
H	-1.10665900	0.66905200	-2.08378100
H	-1.07647800	1.34318300	-0.45132900
H	-1.34539900	-1.72095700	-2.30401700
H	-0.82694600	-2.75418000	-0.97703600
C	-2.88899300	-2.04671700	-0.77032900
H	-2.76233800	0.35289700	1.61081200
H	-2.92155800	-1.38008300	1.90295900
H	-3.43150300	0.18222100	-1.95766800
H	-3.44673500	1.28804100	-0.57811400
H	-3.56209100	-2.22861400	-1.60617200
H	-3.00889500	-2.85202100	-0.04730800
N	-0.89095100	-0.71980900	-0.54467900
N	-3.32260200	-0.76801700	-0.08471600
C	-4.80430400	-0.85834400	0.16120800
H	-5.29815300	-0.90394500	-0.80610900
H	-4.99107500	-1.76961000	0.72387300
Cl	-5.43928000	0.50998900	1.06115400
C	1.76215800	1.62974400	0.00600700
O	1.28259500	2.42572000	-1.08088800
C	0.58630200	3.56447300	-0.56774300
H	0.41161800	4.26243300	-1.35972600
H	-0.34994500	3.25327600	-0.15361900
H	1.17623000	4.02978200	0.19407900
C	3.52801200	0.11514500	-0.97659800
H	3.21998300	0.33671100	-1.97706100
F	6.15477800	-1.90402600	0.53253900

G = −1503.096159 hartrees

ΔG$_{bind}$ = 4.6 kcal mol^{-1}

SelectFluor / methyl benzoate complex :

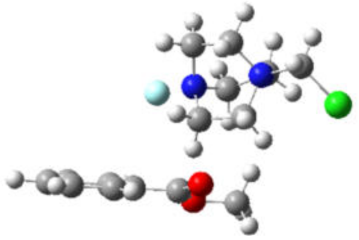

functional: ωB97X-D/6-31++g(2d,p)
Charge = 2, Multiplicity = 1

C	3.29080500	0.39932900	0.27093800
C	3.14462900	-0.62861500	1.20419900
H	2.30015800	-0.61942800	1.88377100
C	4.08798800	-1.64539900	1.27217700
C	5.17518600	-1.64341500	0.40073000
C	5.32226800	-0.62001100	-0.53280500
H	3.97707500	-2.43718300	2.00442300
H	6.16611900	-0.62056500	-1.21365300
O	1.14766600	1.34972000	0.72485900
F	0.52894100	-1.58563800	-0.78048800
C	-0.92365300	-1.00521600	0.93785900
C	-1.03636600	0.05662100	-1.26818000
C	-1.66858600	-2.30226600	-1.00884900
C	-2.28758900	-0.32415700	1.14936600
H	-0.10707500	-0.35266700	1.24916100
H	-0.85167700	-1.98226700	1.41242900
C	-2.55392100	0.29396800	-1.20956200
H	-0.69237700	-0.07741400	-2.29150800
H	-0.45730600	0.83327600	-0.76952400
H	-1.62292400	-2.31232400	-2.09626200
H	-1.29250800	-3.24130900	-0.60694900
C	-3.07285400	-1.98368300	-0.46605000
H	-2.18286900	0.74675500	1.30838800
H	-2.80426400	-0.76537100	1.99990400
H	-3.05547400	-0.01125000	-2.12727000
H	-2.76176700	1.34455600	-1.01582900
H	-3.82445000	-2.17924300	-1.22888500
H	-3.30447800	-2.56538000	0.42486000
N	-0.77060700	-1.21333100	-0.53193900
N	-3.13347500	-0.52336100	-0.08335000
C	-4.57808800	-0.17274300	0.15906200
H	-5.10147700	-0.26591600	-0.79028500
H	-4.96966300	-0.86857500	0.89846800
Cl	-4.77183300	1.47307700	0.75956400
C	2.24989600	1.45691300	0.21616600
O	2.63223900	2.53658200	-0.44782600
C	1.67525200	3.59836800	-0.55955200
H	2.18309700	4.38903800	-1.10784400
H	0.79530800	3.25856800	-1.10874700
H	1.38227400	3.94711700	0.43218200
C	4.38623000	0.40560600	-0.59578700
H	4.49674800	1.20201100	-1.32200700
H	5.91037400	-2.43935400	0.44947000

G = −1403.866871 hartrees

ΔG$_{bind}$ = 5.6 kcal mol^{-1}

Geometry optimization of the **MFB-SF** assembly revealed that the F atom of **SF** interacts with the benzoate. F interacts with the carbonyl carbon perpendicular to the N−F bond (distance between F−C = 2.7 Å, angle between N−F−C = 97°). Furthermore, F is oriented to the carbonyl at an angle of 104° (note that the Bürgi–

Dunitz angle is 107°). This suggests that electrons of F interact with the π* of C=O. On the other hand the arene ring tends to interact at the tip (i.e., closer to 180°) of N-F bond as follows: i) C_{ipso}–F interaction (distance = 2.7 Å , angle = 124°); ii) C_{para}–F interaction (distance = 2.7 Å , angle = 153°). These structural observations suggests that **SF** interacts with the catalyst interacts *via* 2 modes of halogen bonding. Halogen bonding is known in the literature[49] to interact with electron donors linearly *via* its sigma hole or with electron acceptors at the halogen's periphery *via* its lone pairs. The N−F moiety of **SF** was reported to undergo halogen bonding to pyridine N atoms.[50]

On the other hand, such a dual binding mode is not observed for methyl benzoate. The binding of **MFB** and **SF** is 1.0 kcal mol^{-1} more favorable than that of methyl benzoate and **SF**, which is in line with **MFB** being a superior de-aggregating agent (see Section 3.8). Such a binding interaction may direct **SF** in regioselective fluorinations by the **PSAux** method, but advanced spectroscopic probing is needed to confirm this proposal.

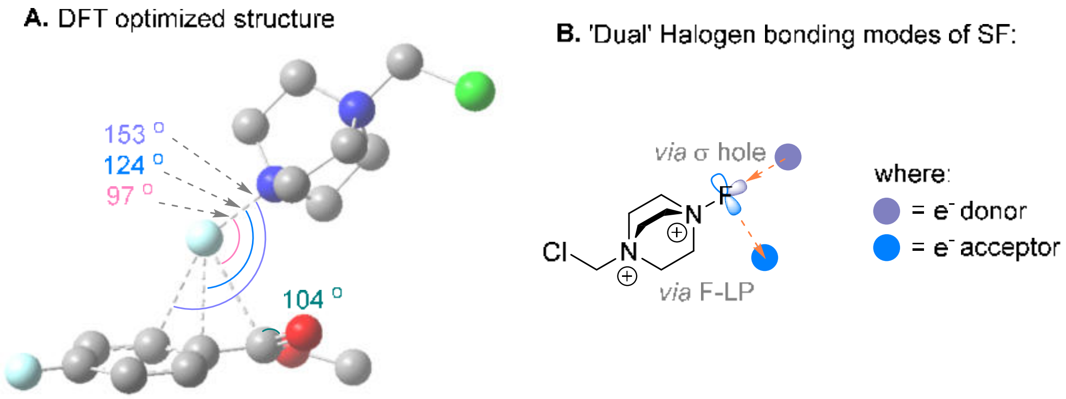

Figure S37. A: DFT optimized structure of the assembly of methyl 4-fluorobenzoate and SelectFluor and relevant angles. **B:** Possible 'dual' binding mores of SelectFluor.

Second Order Perturbation Theory Analysis of Fock Matrix in NBO Basis:

To probe the nature of non-covalent interactions and interacting orbitals between **MFB**-**SF** complex, NBO calculations and Second Order Perturbation theory analysis was carried out. This will provide the interaction energies (E2) between donor and acceptor orbitals of the given system. The following significant interactions support the notion of a dual halogen bonding mode of **SF** with **MFB**:

LP electrons of F to π*C=O (total) = 2.0 kcal mol^{-1}

π electrons of C_{ipso}-C_{para} to σ*F-N = 35.2 kcal mol^{-1}

SelectFluor / methyl 4-fluorobenzoate complex :

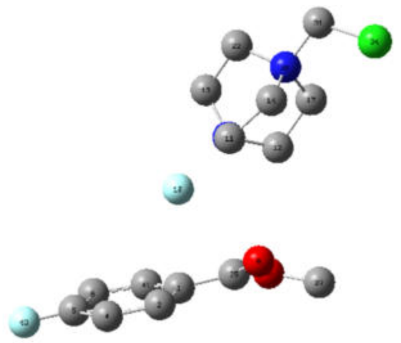

Threshold for printing: 0.50 kcal/mol
(Intermolecular threshold: 0.05 kcal/mol)

Donor NBO (i)	Acceptor NBO (j)	E(2) kcal/mol	E(j)-E(i) a.u.	F(i,j) a.u.
**from unit 1 to unit 2**				
2. BD (1) C 1 - C 35	/264. RY*(2) C 12	0.06	1.61	0.009
2. BD (1) C 1 - C 35	/358. RY*(1) H 19	0.07	1.88	0.010
3. BD (1) C 1 - C 41	/649. BD*(1) F 10 - N 29	0.19	0.57	0.010
4. BD (2) C 1 - C 41	/224. RY*(2) F 10	0.15	1.12	0.013
4. BD (2) C 1 - C 41	/226. RY*(4) F 10	0.08	1.17	0.010
4. BD (2) C 1 - C 41	/649. BD*(1) F 10 - N 29	35.00	0.08	0.048
7. BD (2) C 2 - C 4	/649. BD*(1) F 10 - N 29	1.88	0.09	0.012
11. BD (2) C 5 - C 6	/649. BD*(1) F 10 - N 29	0.08	0.11	0.003
15. BD (1) O 9 - C 35	/424. RY*(2) N 29	0.07	7.50	0.021
16. BD (2) O 9 - C 35	/227. RY*(5) F 10	0.05	2.37	0.010
16. BD (2) O 9 - C 35	/323. RY*(1) H 15	0.19	1.51	0.015
16. BD (2) O 9 - C 35	/358. RY*(1) H 19	0.07	1.57	0.010
16. BD (2) O 9 - C 35	/649. BD*(1) F 10 - N 29	0.10	0.26	0.005
16. BD (2) O 9 - C 35	/651. BD*(1) C 11 - H 15	0.94	1.00	0.027
16. BD (2) O 9 - C 35	/656. BD*(1) C 12 - H 19	0.48	0.99	0.020
43. BD (1) C 35 - O 36	/436. RY*(14) N 29	0.06	9.25	0.021
45. BD (1) C 37 - H 38	/656. BD*(1) C 12 - H 19	0.07	1.11	0.008
46. BD (1) C 37 - H 39	/358. RY*(1) H 19	0.15	1.69	0.014
46. BD (1) C 37 - H 39	/656. BD*(1) C 12 - H 19	0.95	1.11	0.029
47. BD (1) C 37 - H 40	/358. RY*(1) H 19	0.10	1.69	0.012
47. BD (1) C 37 - H 40	/656. BD*(1) C 12 - H 19	0.11	1.11	0.010
48. BD (1) C 41 - H 42	/649. BD*(1) F 10 - N 29	0.64	0.41	0.016
75. LP (1) O 9	/651. BD*(1) C 11 - H 15	0.83	1.32	0.030
75. LP (1) O 9	/656. BD*(1) C 12 - H 19	0.16	1.32	0.013
76. LP (2) O 9	/649. BD*(1) F 10 - N 29	0.27	0.13	0.006
76. LP (2) O 9	/651. BD*(1) C 11 - H 15	0.25	0.87	0.014
76. LP (2) O 9	/656. BD*(1) C 12 - H 19	0.39	0.87	0.017
83. LP (1) O 36	/430. RY*(8) N 29	0.07	2.64	0.012
84. LP (2) O 36	/264. RY*(2) C 12	0.08	1.23	0.009
84. LP (2) O 36	/265. RY*(3) C 12	0.06	1.41	0.009
84. LP (2) O 36	/649. BD*(1) F 10 - N 29	0.10	0.18	0.004
84. LP (2) O 36	/654. BD*(1) C 12 - C 17	0.10	0.78	0.008
84. LP (2) O 36	/656. BD*(1) C 12 - H 19	0.13	0.91	0.010
648. BD*(2) O 9 - C 35	/651. BD*(1) C 11 - H 15	0.14	0.46	0.021
675. BD*(1) C 35 - O 36	/657. BD*(1) C 12 - N 29	0.05	0.02	0.004
**from unit 2 to unit 1**				
17. BD (1) F 10 - N 29	/590. RY*(3) C 41	0.05	1.94	0.009
17. BD (1) F 10 - N 29	/591. RY*(4) C 41	0.05	1.98	0.009

17. BD (1) F 10 - N 29	/636. BD*(2) C 1 - C 41	0.22	0.65	0.012
19. BD (1) C 11 - H 15	/204. RY*(2) O 9	0.09	1.49	0.010
19. BD (1) C 11 - H 15	/648. BD*(2) O 9 - C 35	0.14	0.71	0.009
21. BD (1) C 11 - N 29	/100. RY*(13) C 1	0.06	2.62	0.011
21. BD (1) C 11 - N 29	/523. RY*(11) C 35	0.08	2.46	0.012
21. BD (1) C 11 - N 29	/525. RY*(13) C 35	0.10	3.05	0.015
21. BD (1) C 11 - N 29	/597. RY*(10) C 41	0.05	2.92	0.011
23. BD (1) C 12 - H 18	/678. BD*(1) C 37 - H 39	0.06	1.22	0.008
24. BD (1) C 12 - H 19	/578. RY*(1) H 39	0.05	1.62	0.008
24. BD (1) C 12 - H 19	/678. BD*(1) C 37 - H 39	0.19	1.23	0.014
25. BD (1) C 12 - N 29	/100. RY*(13) C 1	0.05	2.62	0.010
25. BD (1) C 12 - N 29	/523. RY*(11) C 35	0.07	2.46	0.012
25. BD (1) C 12 - N 29	/525. RY*(13) C 35	0.08	3.05	0.014
25. BD (1) C 12 - N 29	/597. RY*(10) C 41	0.06	2.92	0.012
29. BD (1) C 13 - N 29	/100. RY*(13) C 1	0.10	2.62	0.015
29. BD (1) C 13 - N 29	/523. RY*(11) C 35	0.10	2.46	0.014
29. BD (1) C 13 - N 29	/525. RY*(13) C 35	0.14	3.05	0.019
29. BD (1) C 13 - N 29	/597. RY*(10) C 41	0.05	2.92	0.011
32. BD (1) C 14 - N 30	/597. RY*(10) C 41	0.08	2.85	0.013
35. BD (1) C 17 - N 30	/597. RY*(10) C 41	0.08	2.85	0.014
38. BD (1) C 22 - N 30	/597. RY*(10) C 41	0.08	2.85	0.013
39. BD (1) N 30 - C 31	/523. RY*(11) C 35	0.09	2.41	0.013
39. BD (1) N 30 - C 31	/524. RY*(12) C 35	0.07	2.85	0.012
39. BD (1) N 30 - C 31	/525. RY*(13) C 35	0.13	3.00	0.018
39. BD (1) N 30 - C 31	/581. RY*(4) H 39	0.05	1.94	0.009
77. LP (1) F 10	/633. BD*(1) C 1 - C 2	0.06	1.79	0.010
77. LP (1) F 10	/648. BD*(2) O 9 - C 35	0.08	1.18	0.009
77. LP (1) F 10	/680. BD*(1) C 41 - H 42	0.07	1.72	0.010
78. LP (2) F 10	/636. BD*(2) C 1 - C 41	0.33	0.69	0.015
78. LP (2) F 10	/648. BD*(2) O 9 - C 35	0.11	0.70	0.008
79. LP (3) F 10	/633. BD*(1) C 1 - C 2	0.17	1.24	0.013
79. LP (3) F 10	/639. BD*(2) C 2 - C 4	0.06	0.64	0.006
79. LP (3) F 10	/646. BD*(1) C 6 - C 41	0.09	1.17	0.009
79. LP (3) F 10	/648. BD*(2) O 9 - C 35	1.84	0.63	0.032
649. BD*(1) F 10 - N 29	/100. RY*(13) C 1	0.06	1.90	0.021
649. BD*(1) F 10 - N 29	/523. RY*(11) C 35	0.07	1.75	0.021
649. BD*(1) F 10 - N 29	/525. RY*(13) C 35	0.10	2.33	0.029
649. BD*(1) F 10 - N 29	/597. RY*(10) C 41	0.06	2.21	0.021
649. BD*(1) F 10 - N 29	/636. BD*(2) C 1 - C 41	0.39	0.27	0.014
649. BD*(1) F 10 - N 29	/639. BD*(2) C 2 - C 4	0.06	0.29	0.006
649. BD*(1) F 10 - N 29	/643. BD*(2) C 5 - C 6	0.11	0.30	0.008
649. BD*(1) F 10 - N 29	/648. BD*(2) O 9 - C 35	0.05	0.28	0.006

3.6 Quantum Yield Measurement

Quantum yield is a measurement for probing the photon efficiency of photochemistry reactions to confirm whether radical chain processes are involved.[49] The previously reported apparatus[50] shown in Figure S38 combines optoelectronic measurement of the absorbed amount of light with the quantitative measurement of product formed by ^{19}F NMR. For this measurement, 400 nm LED (Manufacturer – Luxeon, Type – LHUV-0400-0450, I_{max} – 100 mA, U_{max} – 3.1 V) was used. For an accurate optoelectronic measurement, it is important that the reaction mixture is clear and transparent to avoid the interference of light scattering on the measurement. Therefore, a 0.1 M (1 eq.) concentration of **SF** and a 0.15 M (1.5 eq.) concentration of substrate **8b** were used, where everything was dissolved. Due to low intensity of LED and large distance between LED and the sample, the reaction was slow, and it was run for 96 h. The experiment was carried our two times and, in both cases, similar values of Φ was obtained (Table S17). The obtained values for Φ

are all markedly less than 1, suggesting it is very unlikely that a radical chain mechanism is not involved (although a radical chain mechanism with an efficient mechanism for chain-death/termination cannot be fully excluded).

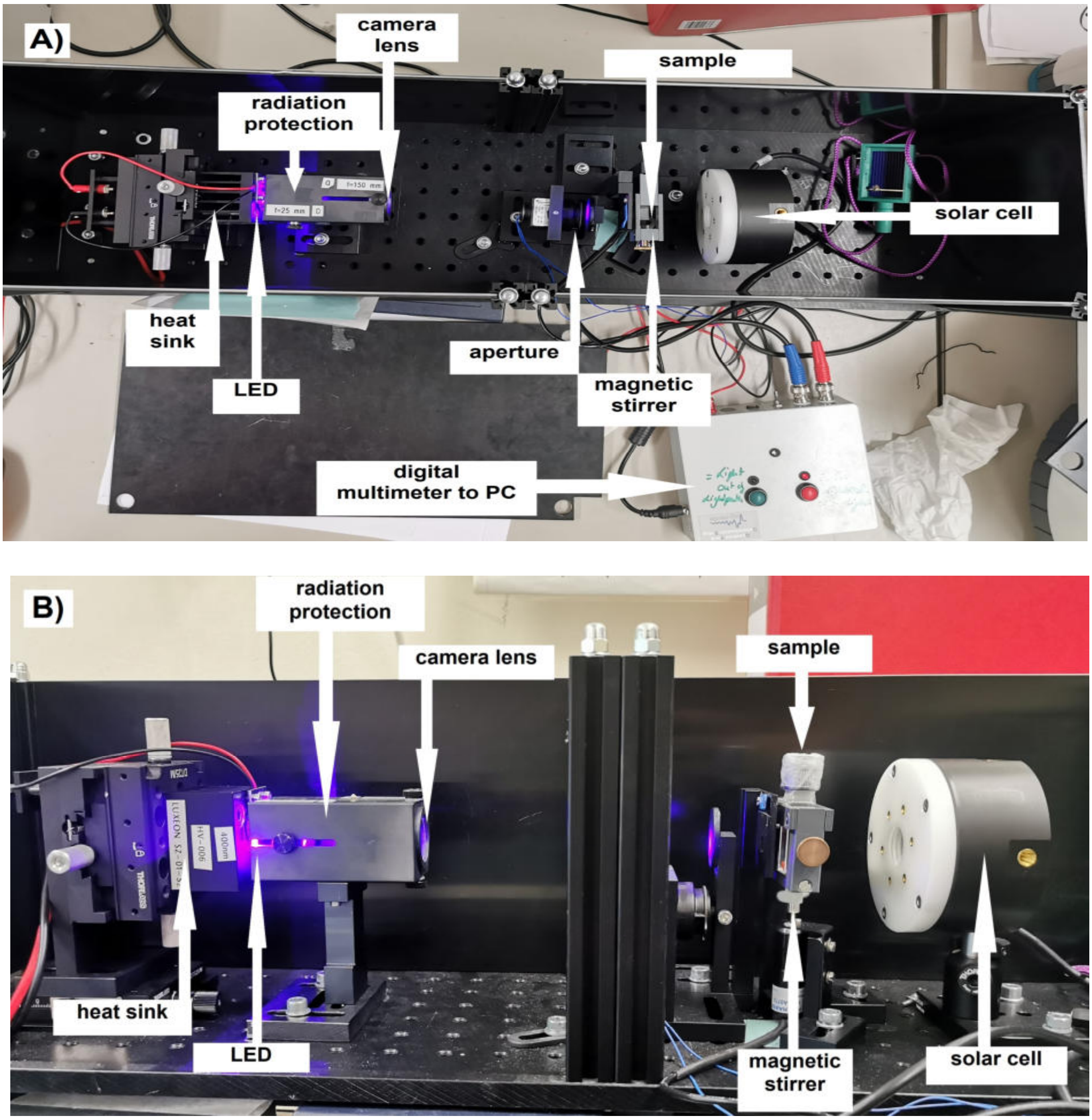

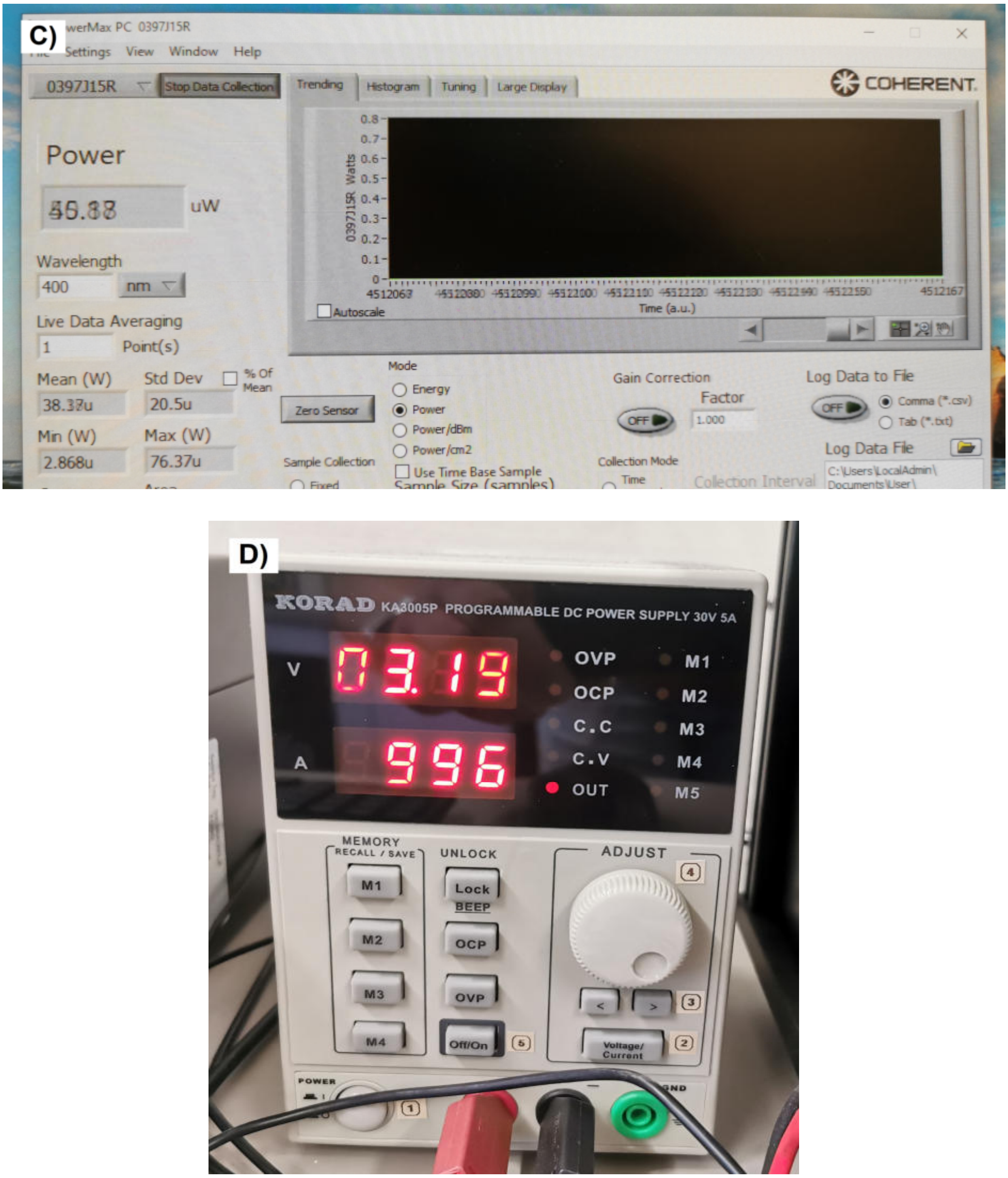

Figure S38. A) and **B)** Assembled setup for quantitative irradiation of reaction mixture using high 400 nm LED, as previously reported.[50] **C)** Application of PC for running the quantum yield measurement. **D)** Adjustable power supply "KORAD KA3005D – Precision Variable Adjustable 30 V, 5 A DC Power Supply Digital Regulated Lab Grade".

The quantum yield was calculated by the following equation:

$$\phi = \frac{N_{prod}}{N_{photons,abs}} = N_A hc \frac{c_{prod} V}{P_{abs} \Delta t \lambda_{LED}}$$

where, N_A is the Avogadro constant, h is Planck's constant, c is the speed of light, c_{prod} is the product concentration, V is the sample volume, Δt is the illumination time and λ_{LED} is the central wavelength of the LED. The absorbed radiant power P_{abs} can be calculated from P_{ref} and P_{sample}, where a small correction factor is applied to correct for back reflection from the terminal glass/air interface.[50]

Table S17. Calculation of quantum yield Φ after 96 h irradiation time from the radiant power of the reference (solvent, P_{ref}) and the radiant power of reaction mixture (P_{sample}) with stirring.

Entry	Irradiation time (min)	P_{ref} (μW)	P_{sample} (μW)	^{19}F NMR Yield[a] (%)	Φ
1	5801	69.00	65.70	12	0.00585 (i.e. 0.6%)
2	5760	69.00	65.85	9	0.00461 (i.e. 0.5%)

[a] Pentafluorobenzene was used as an internal standard for quantification of product yields by ^{19}F NMR.

4 ^{1}H NMR, ^{13}C NMR and ^{19}F NMR Spectra

^{1}H NMR of compound **MFB** in $CDCl_3$

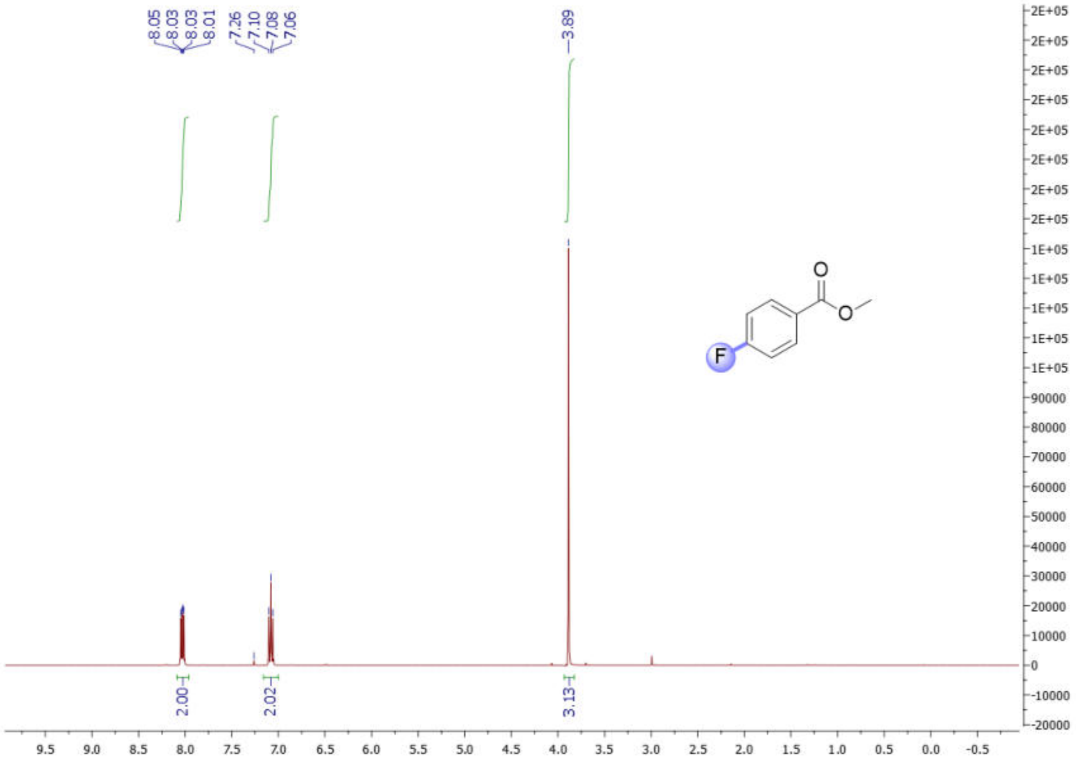

^{13}C NMR of compound **MFB** in $CDCl_3$

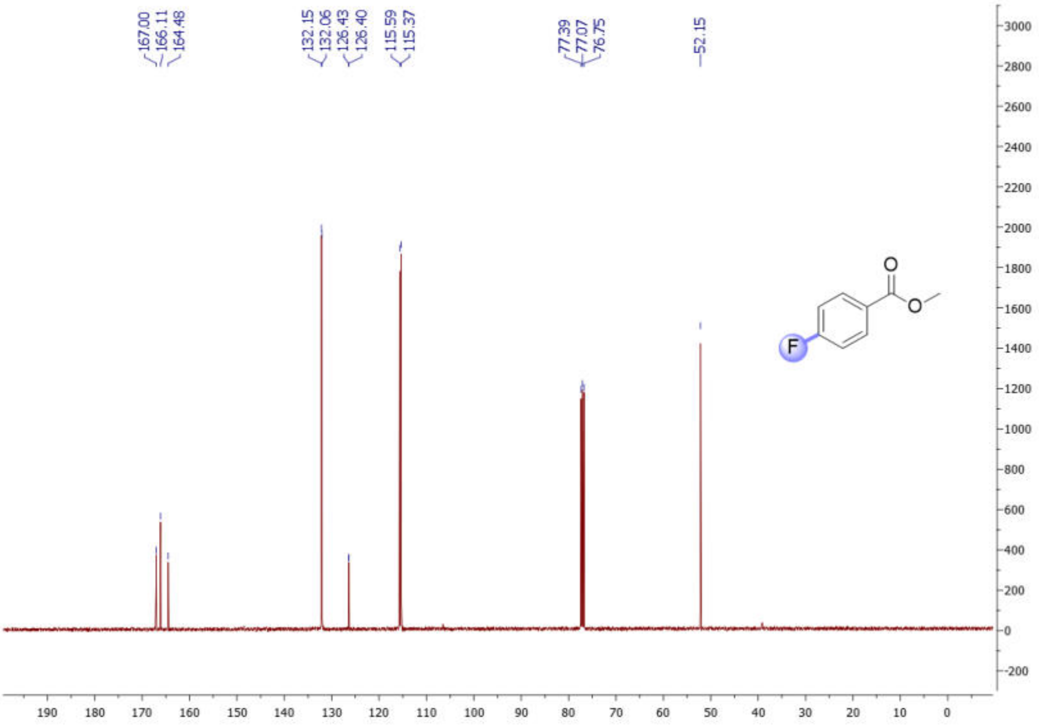

^{19}F NMR of compound **MFB** in $CDCl_3$

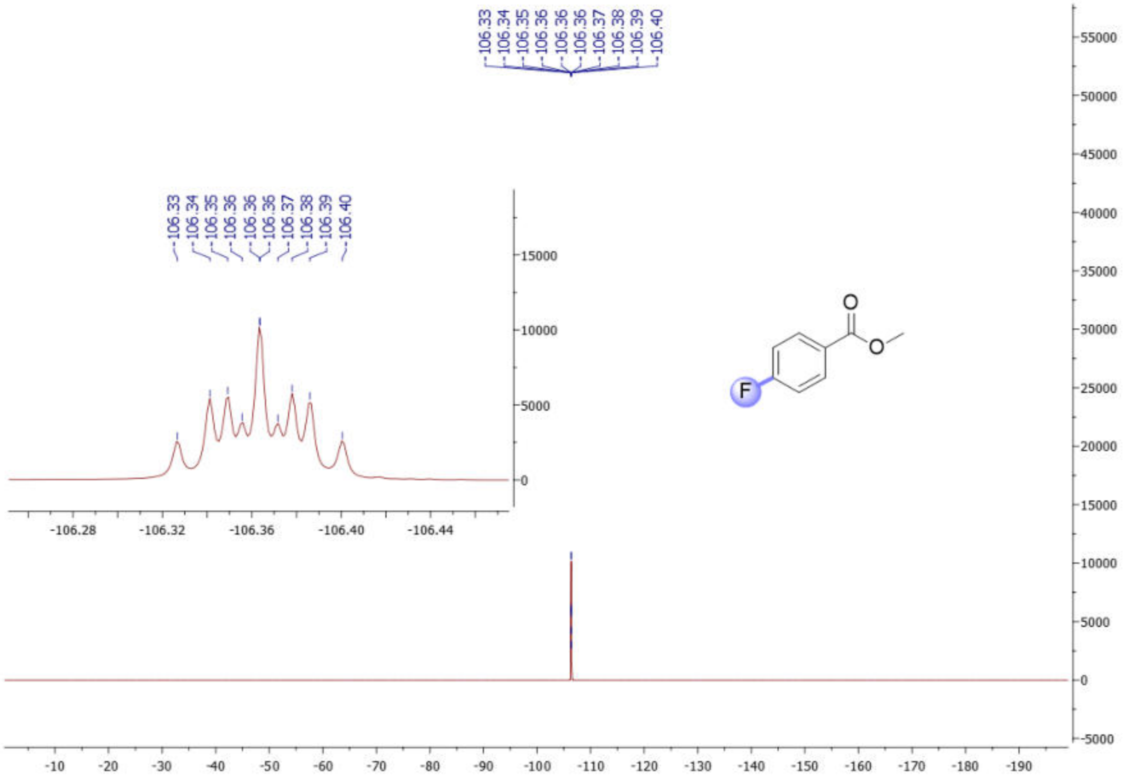

^{1}H NMR of compound **22** in $CDCl_3$

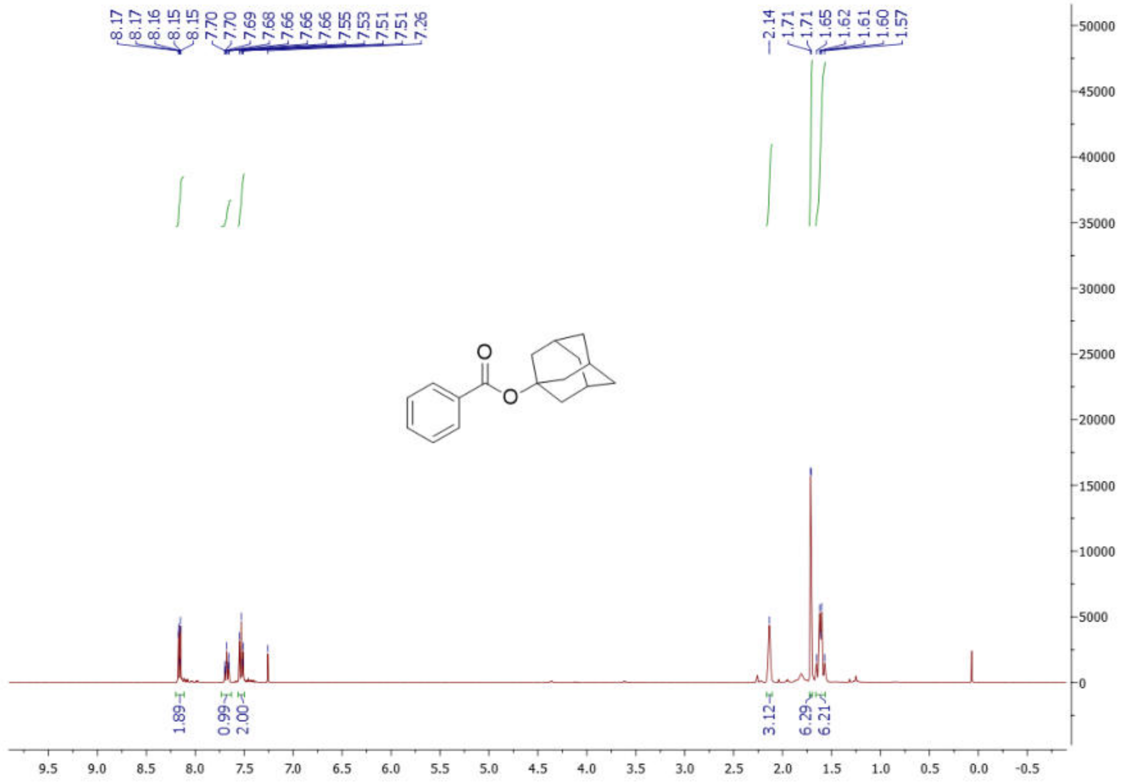

^{13}C NMR of compound **22** in $CDCl_3$

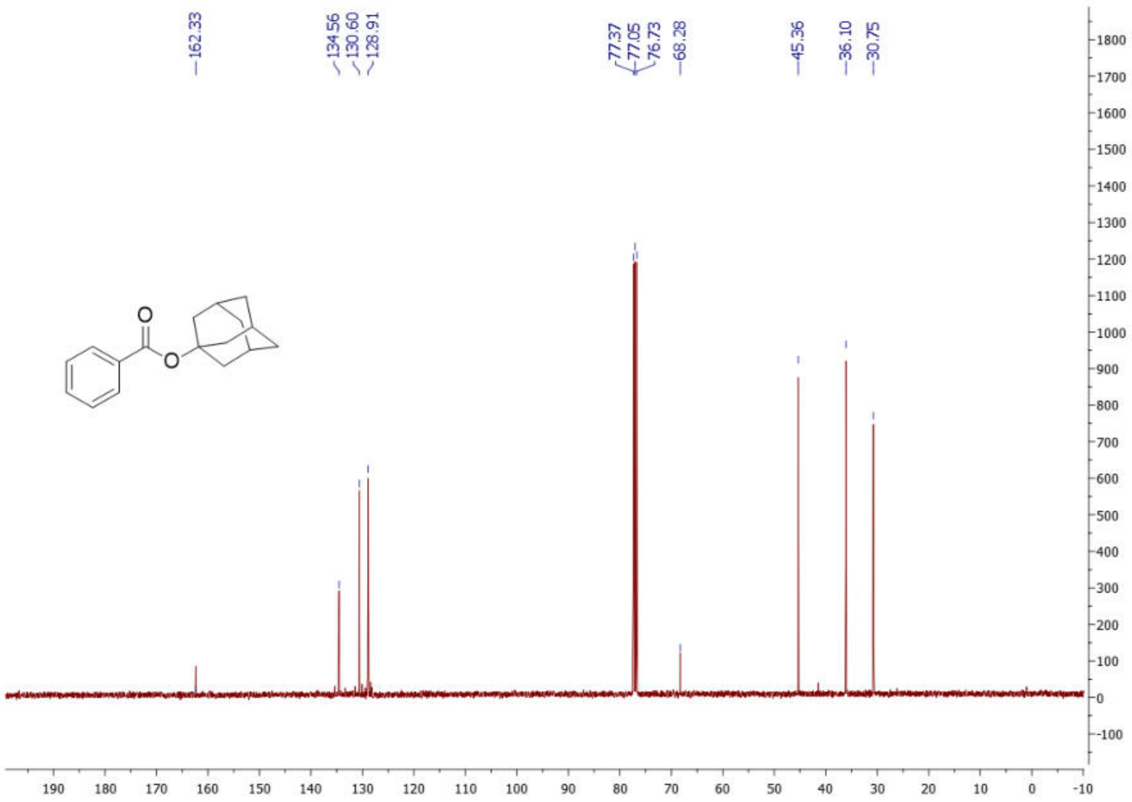

^{1}H NMR of compound **24** in $CDCl_3$

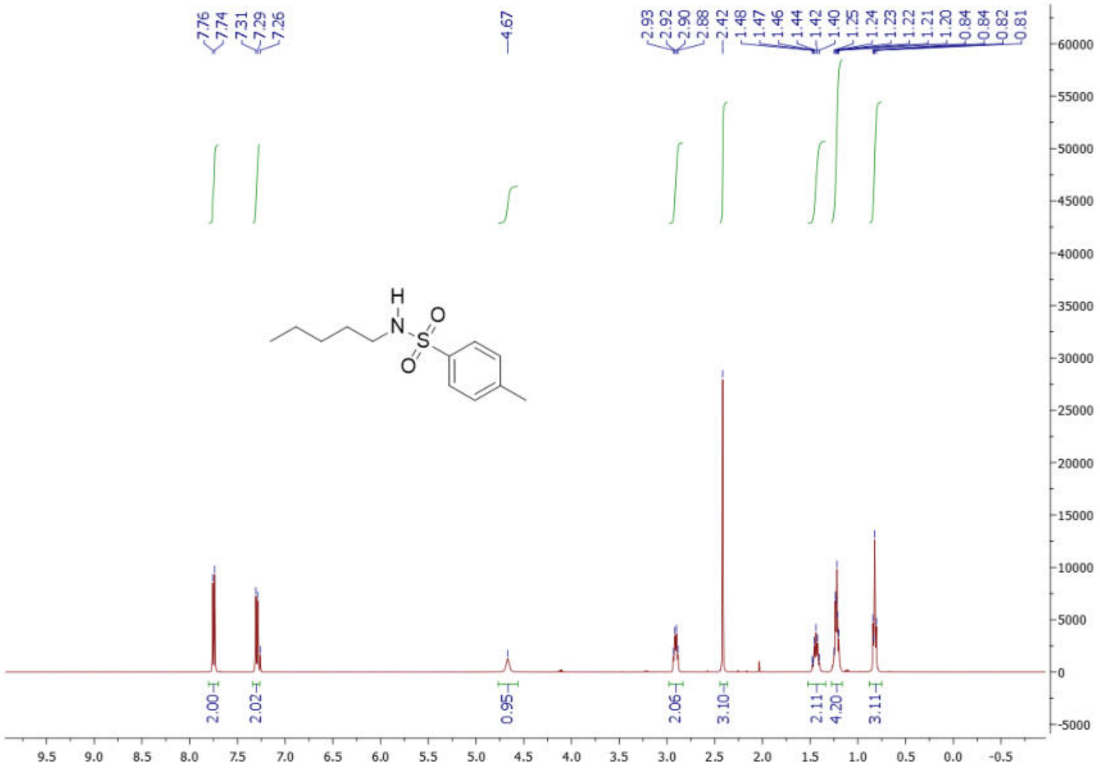

^{13}C NMR of compound **24** in $CDCl_3$

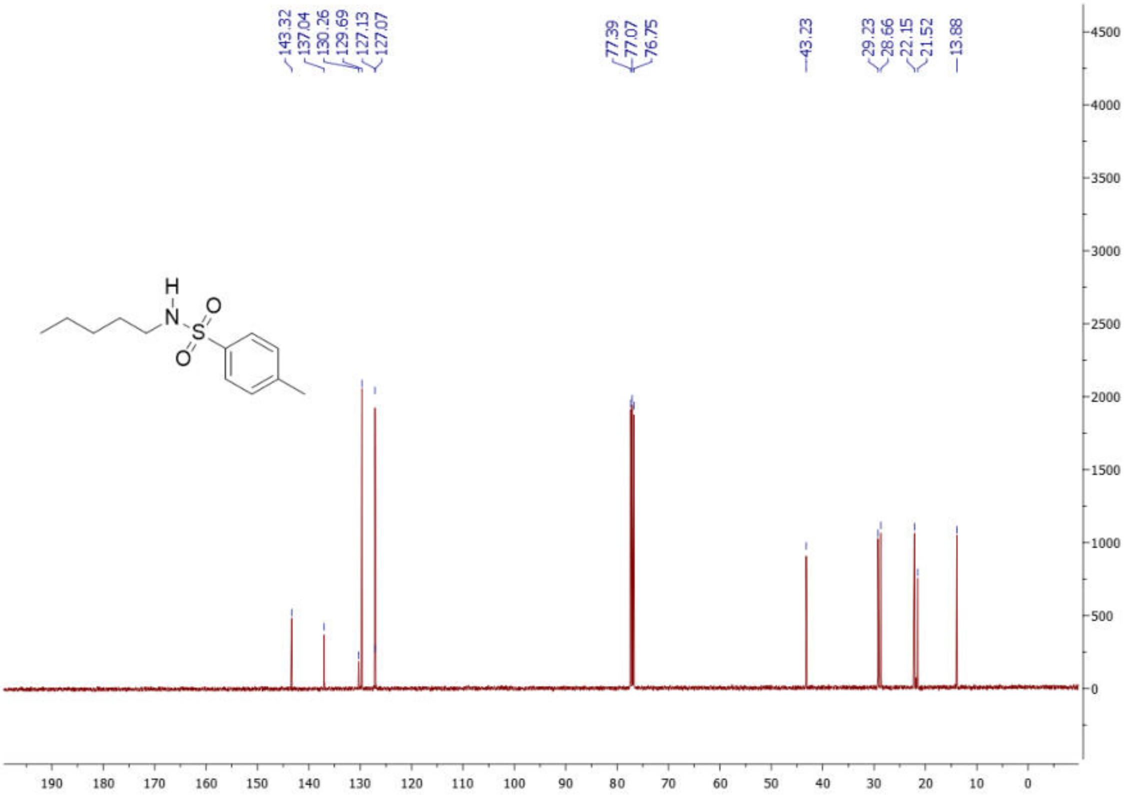

^{1}H NMR of compound **1f** in $CDCl_3$

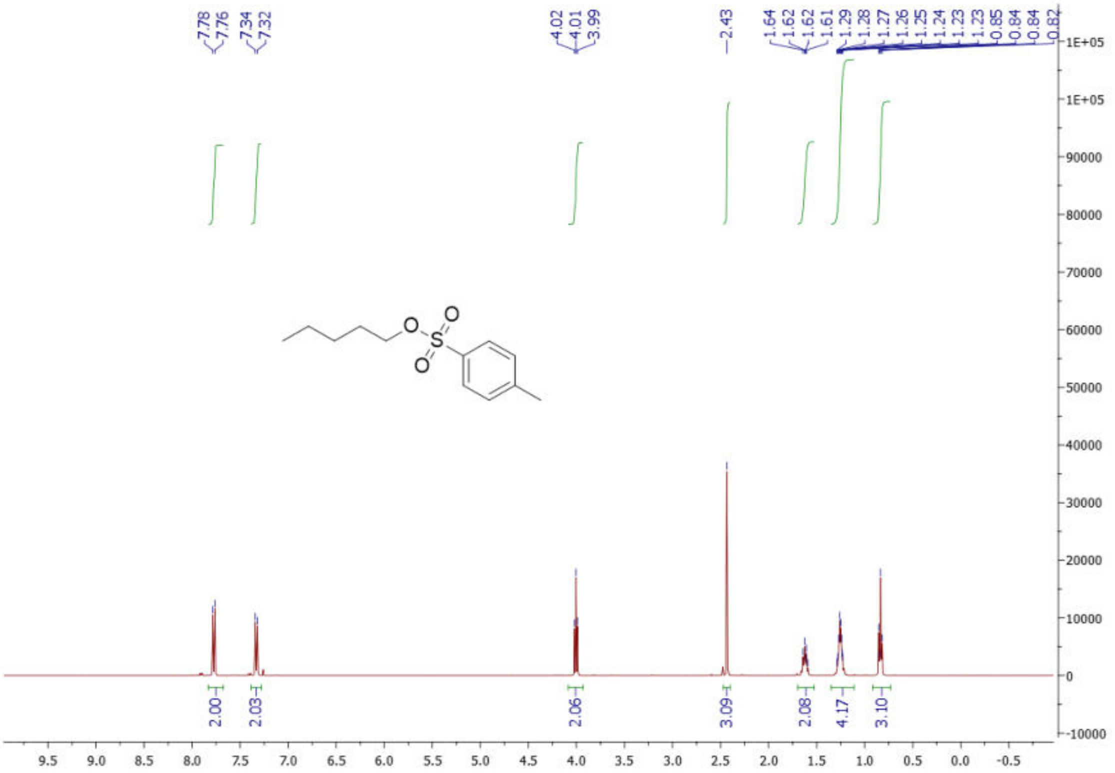

^{13}C NMR of compound **1f** in $CDCl_3$

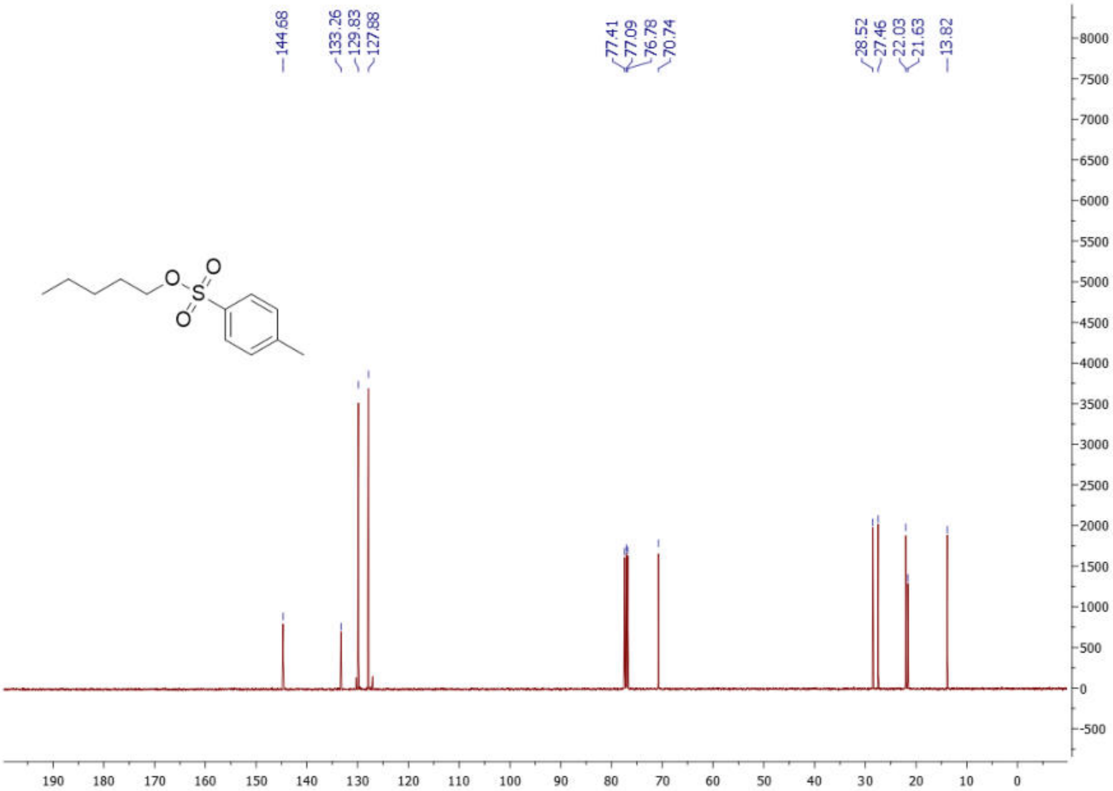

^{1}H NMR of compound **1c** in $CDCl_3$

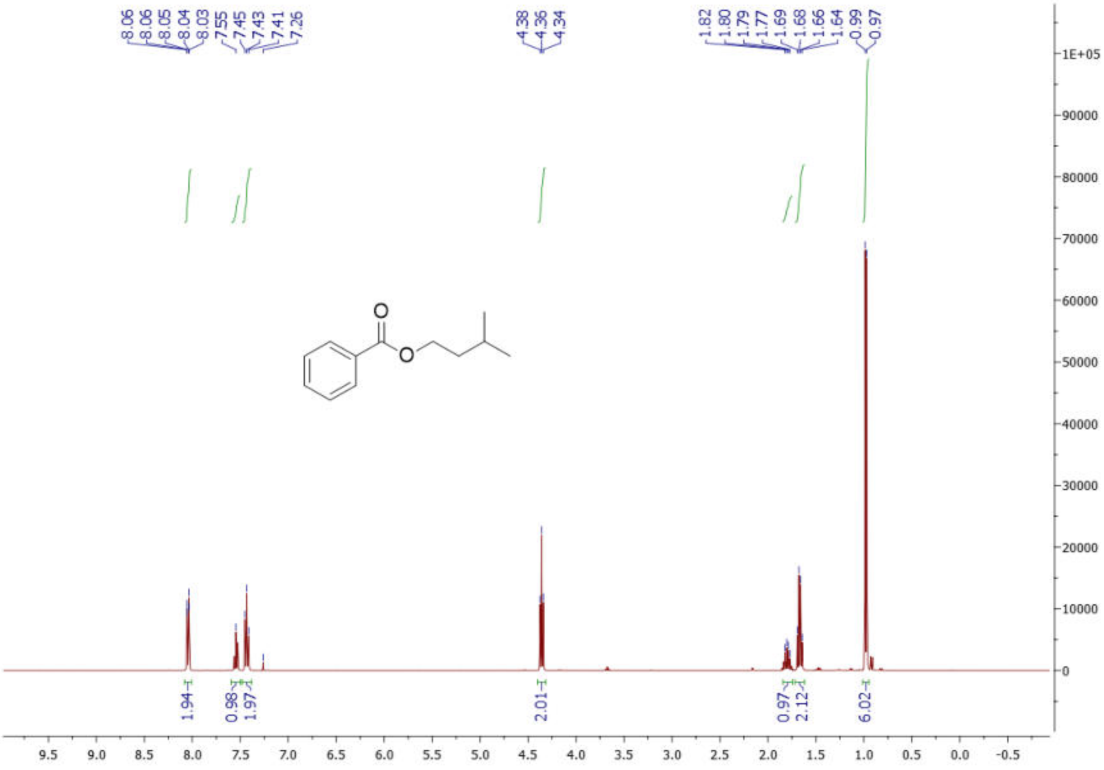

^{13}C NMR of compound **1c** in $CDCl_3$

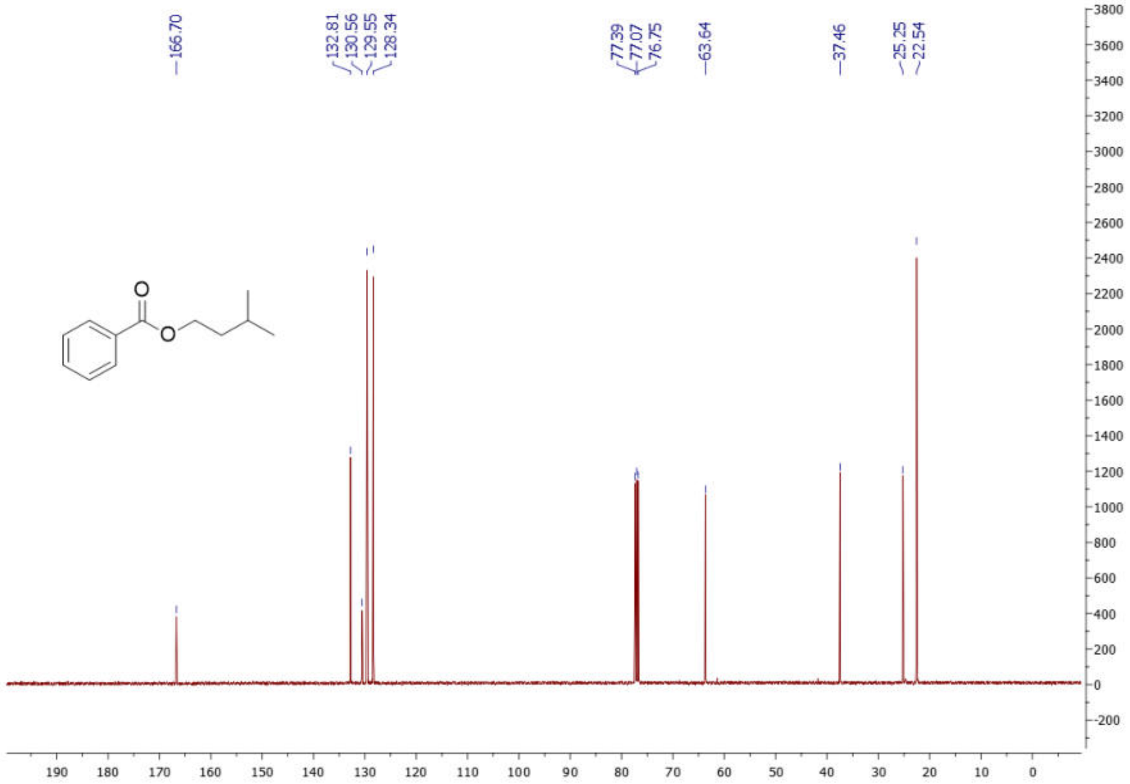

^{1}H NMR of compound **25** in $CDCl_3$

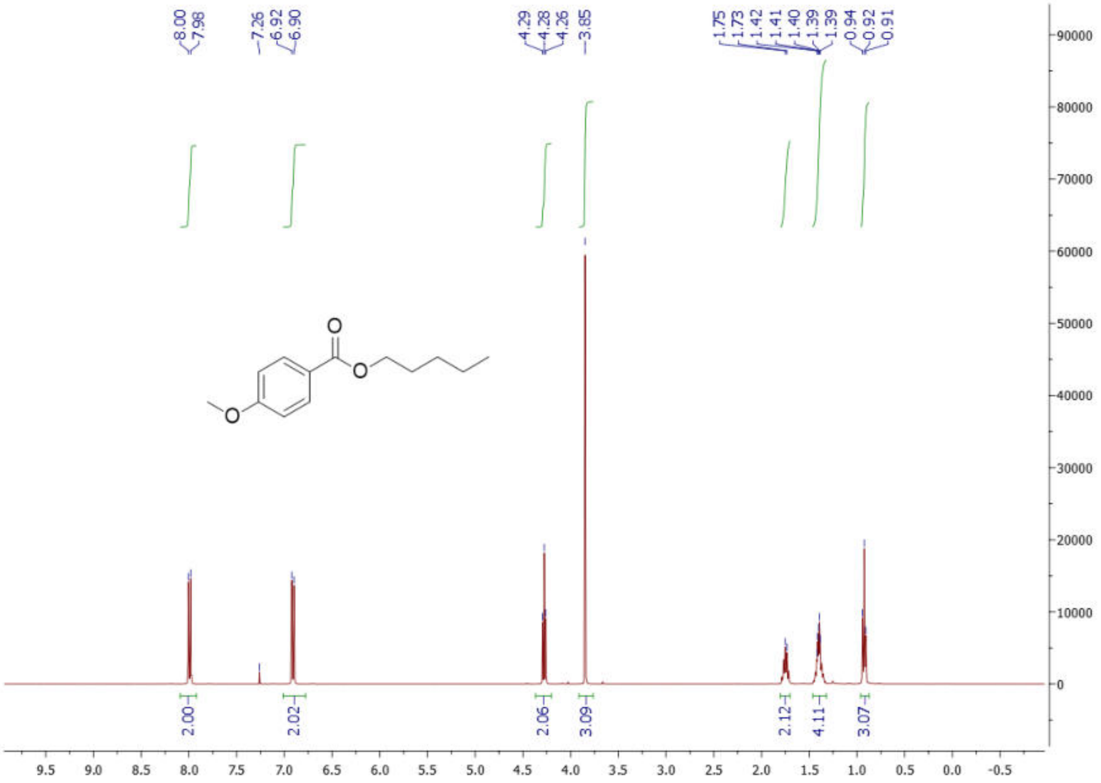

^{13}C NMR of compound **25** in $CDCl_3$

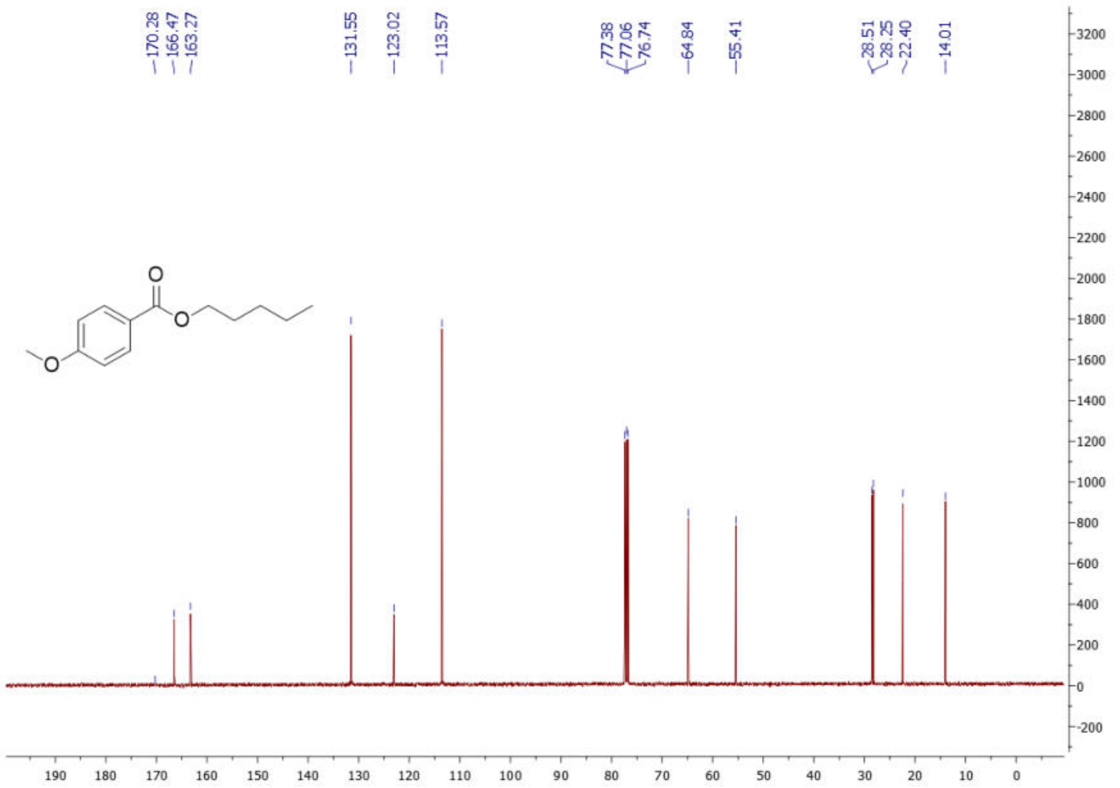

^{1}H NMR of compound **5b** in $CDCl_3$

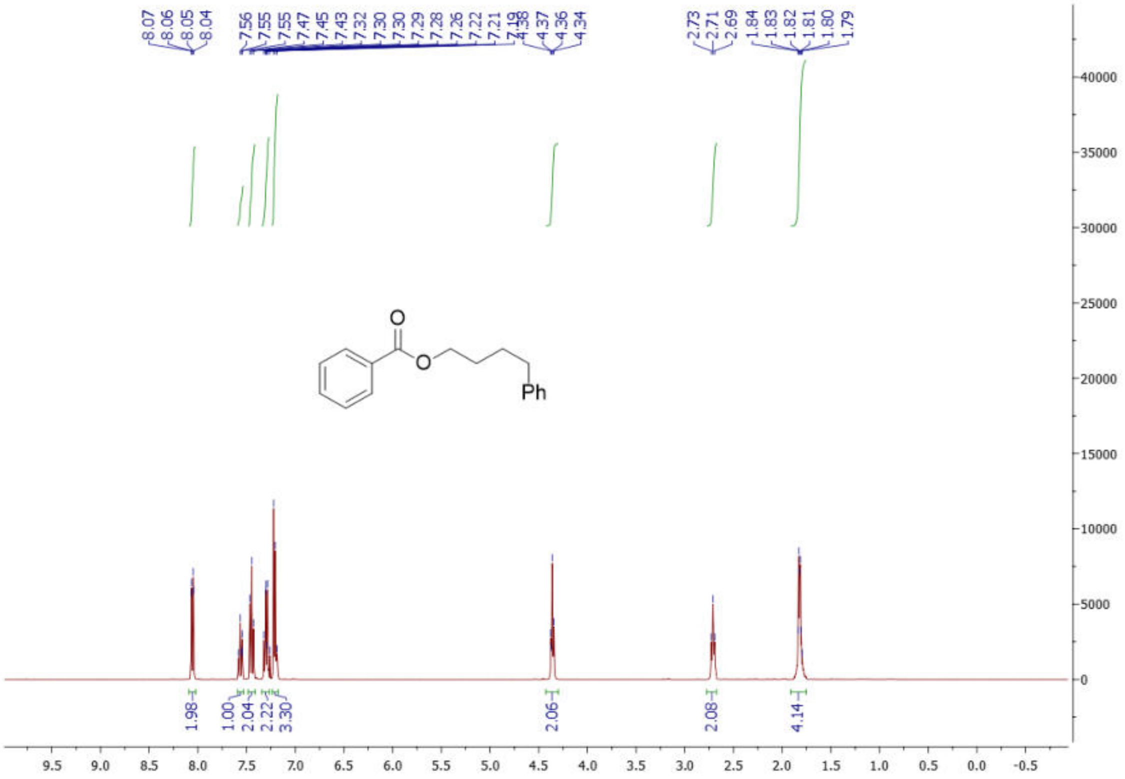

^{13}C NMR of compound **5b** in $CDCl_3$

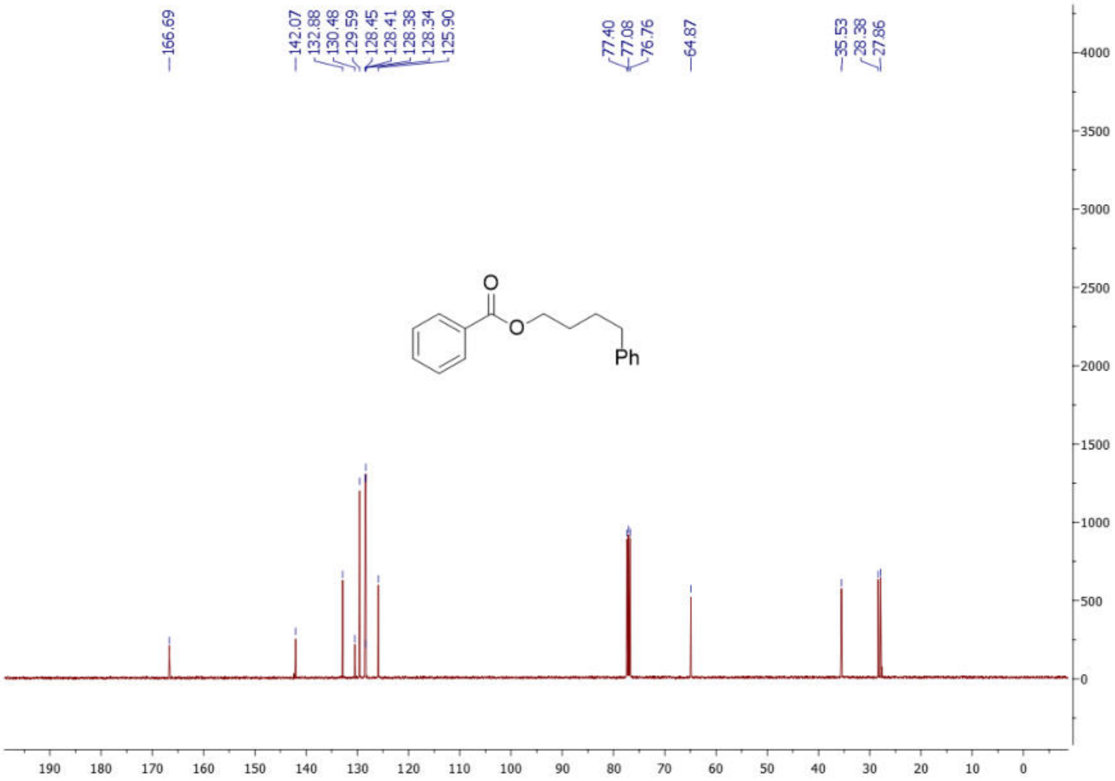

^{1}H NMR of compound **1g** in $CDCl_3$

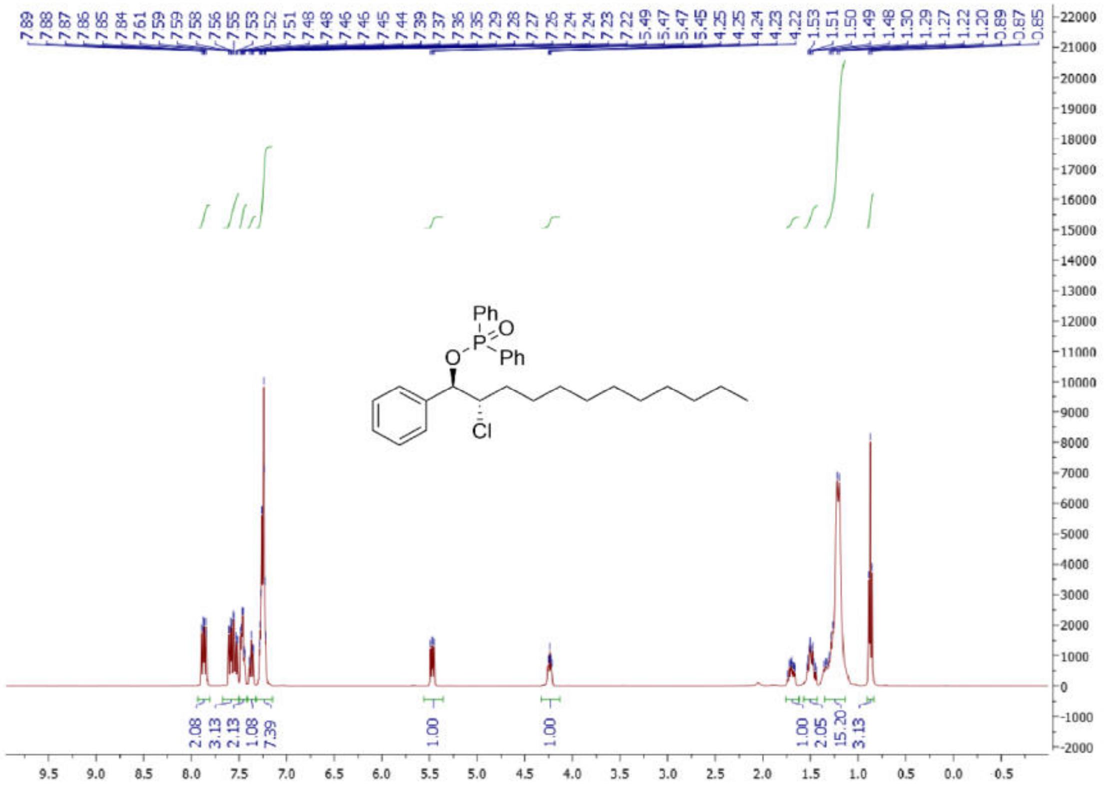

^{13}C NMR of compound **1g** in $CDCl_3$

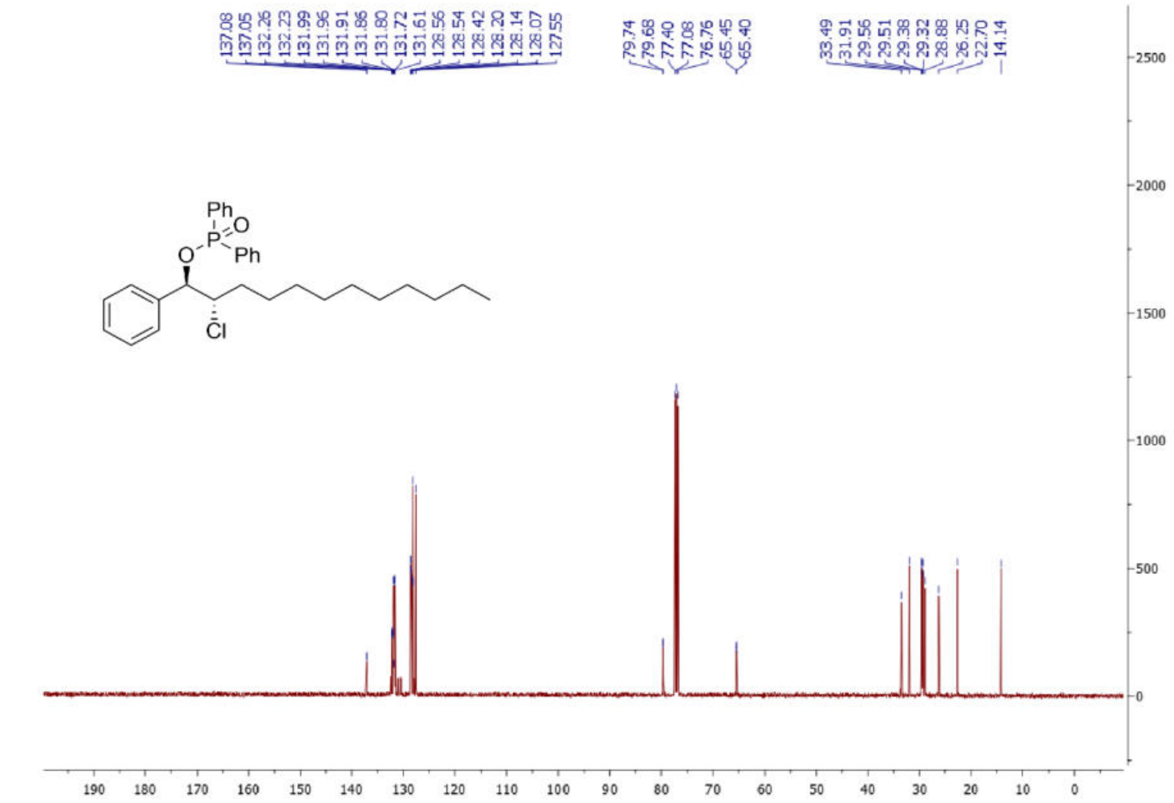

^{1}H NMR of compound **1o** in $CDCl_3$

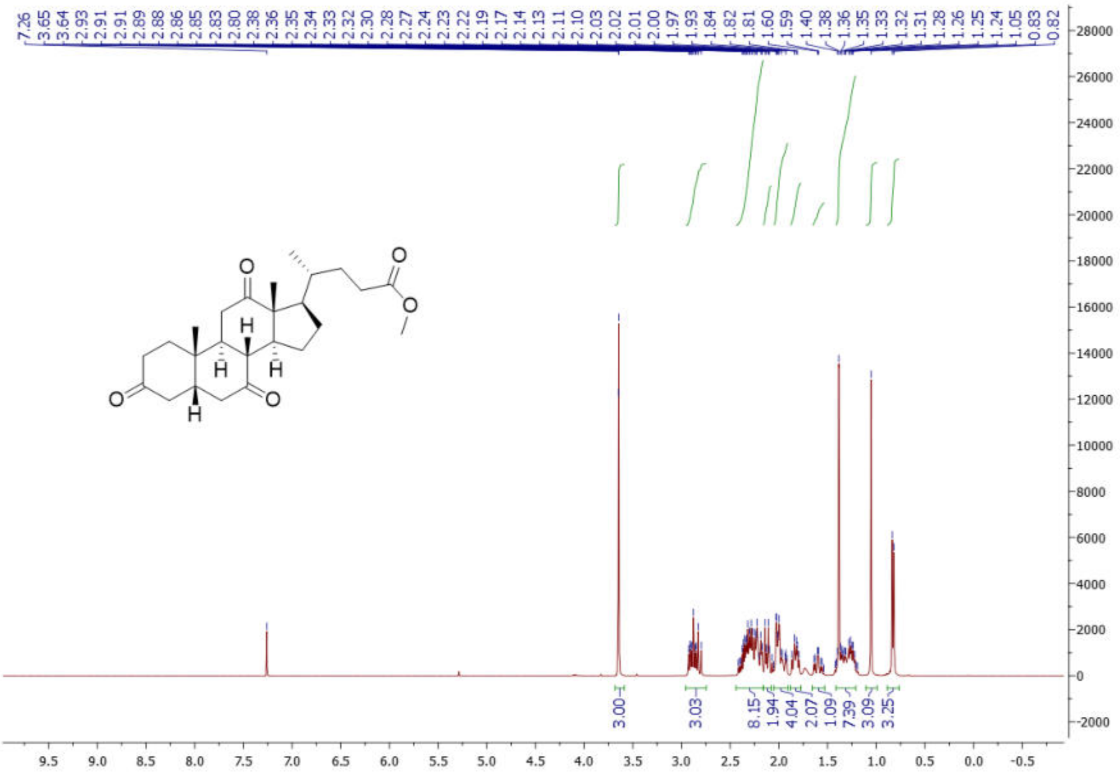

^{13}C NMR of compound **1o** in $CDCl_3$

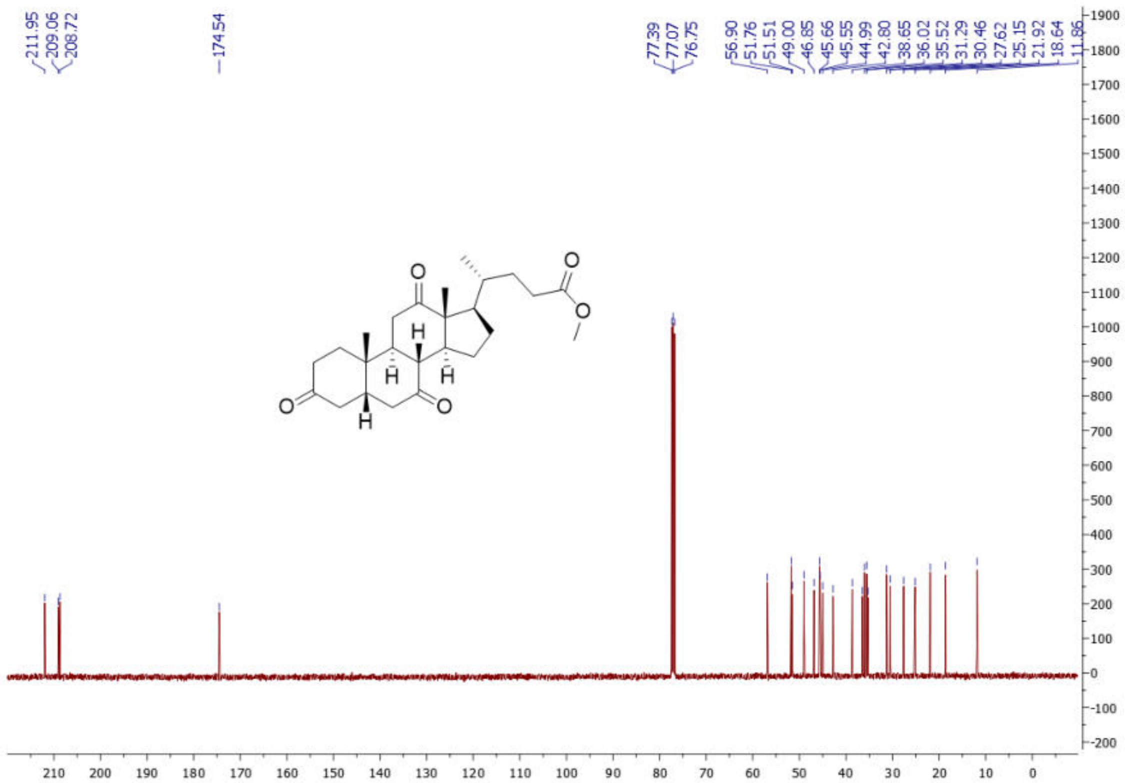

^{1}H NMR of compound **1y** in $CDCl_3$

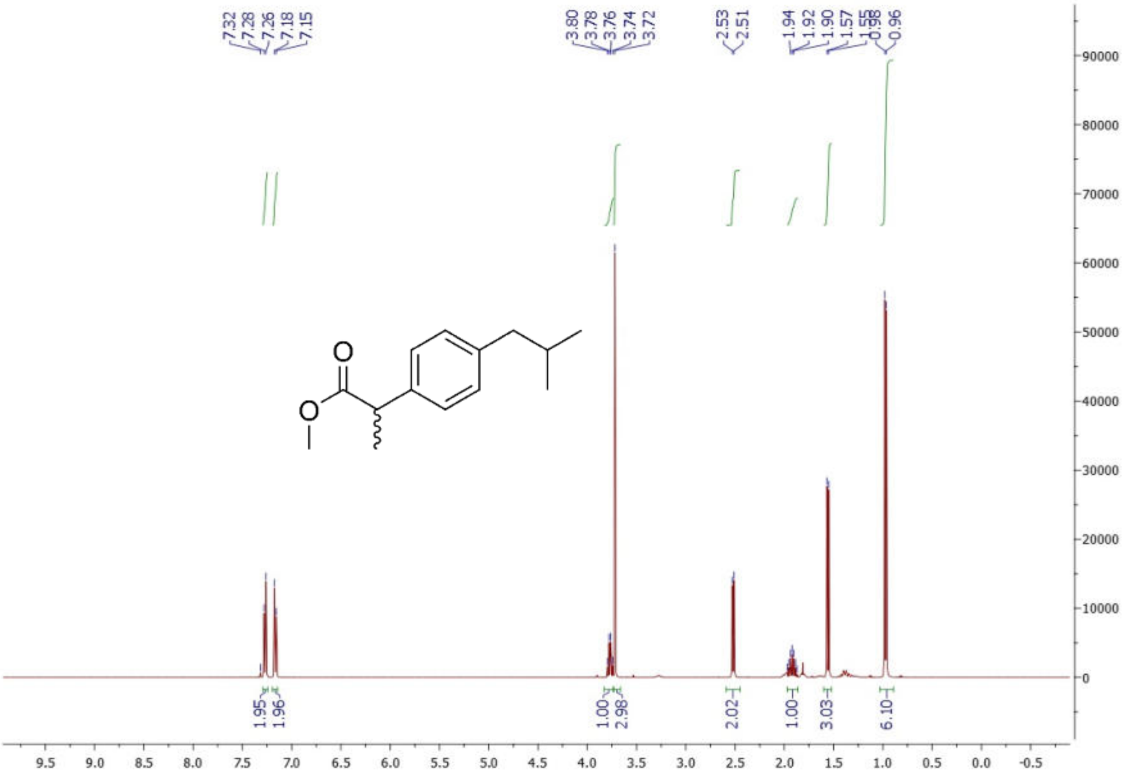

^{13}C NMR of compound **1y** in $CDCl_3$

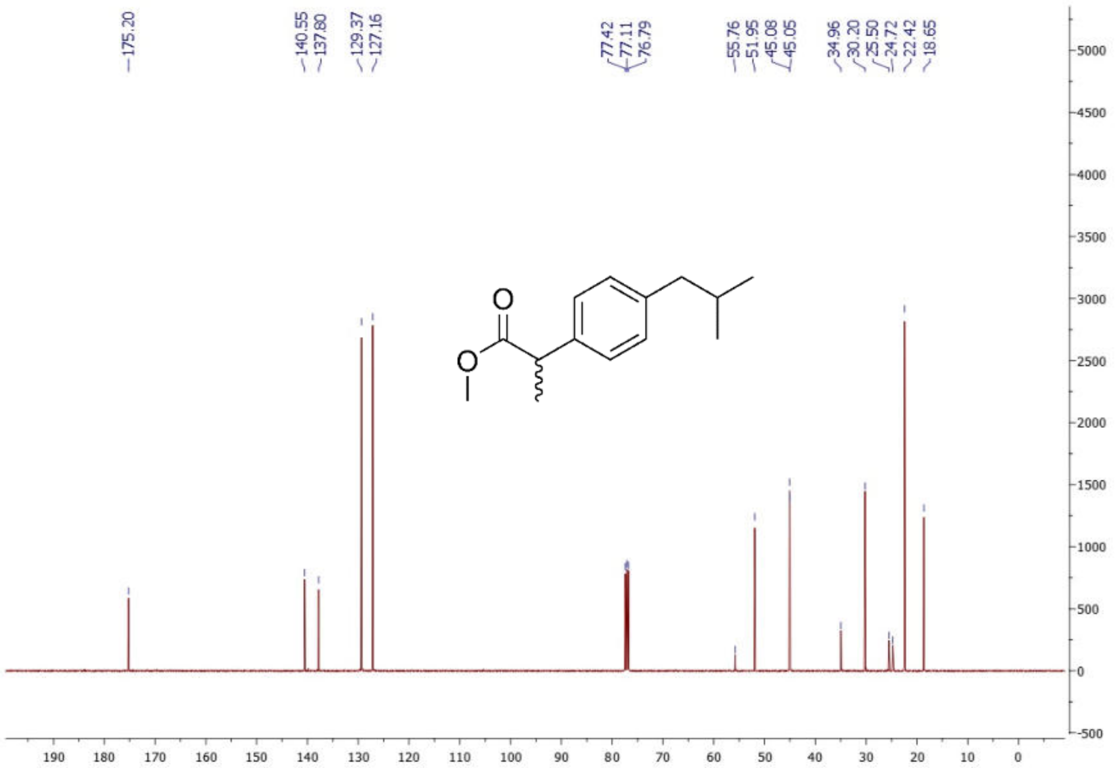

^{1}H NMR of compound **19** in $CDCl_3$

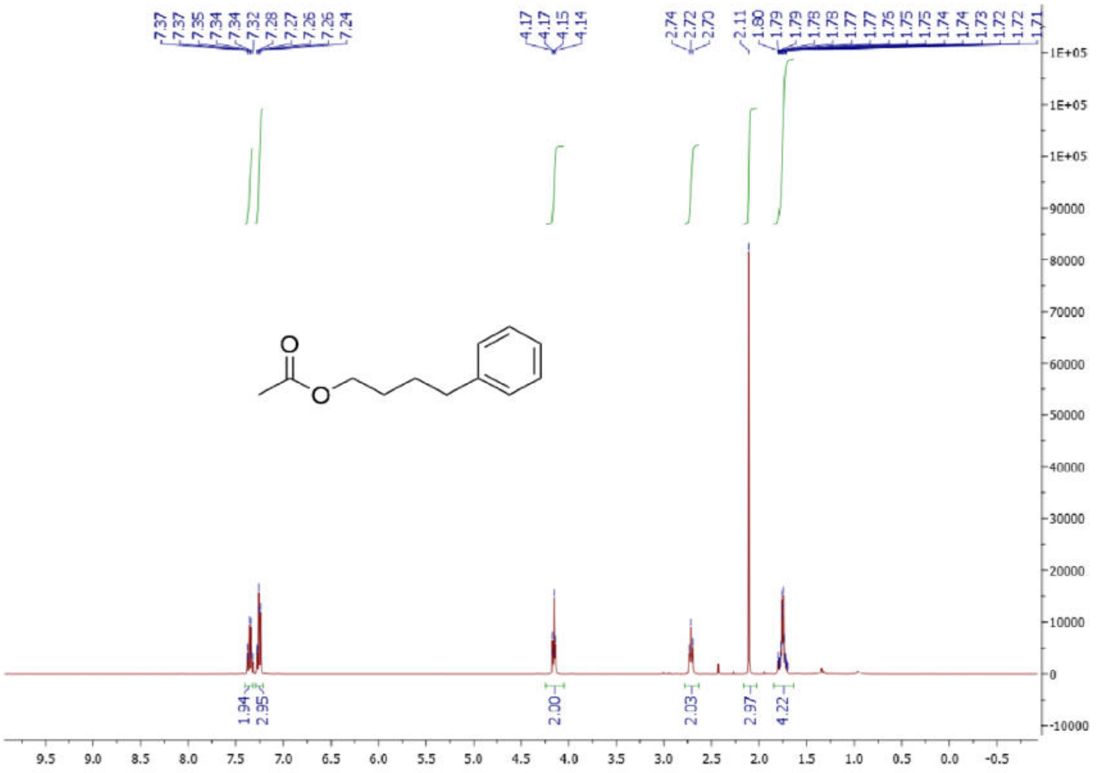

^{13}C NMR of compound **19** in $CDCl_3$

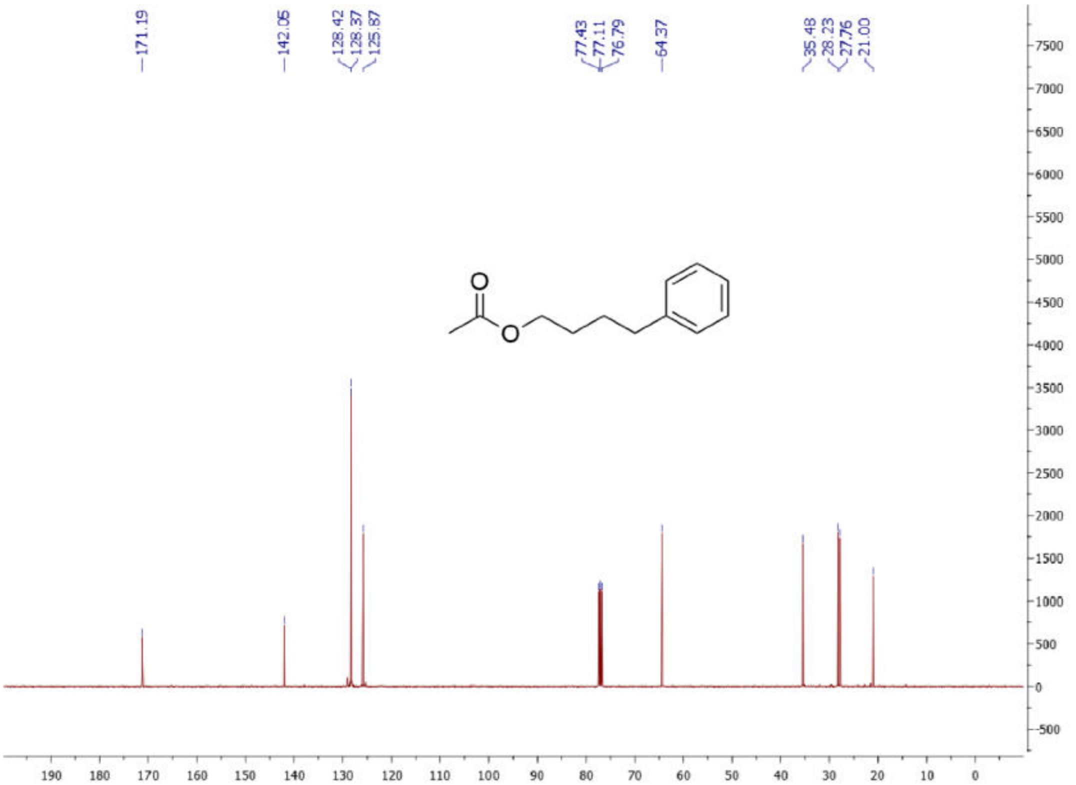

^{1}H NMR of compound **1aa** in $CDCl_3$

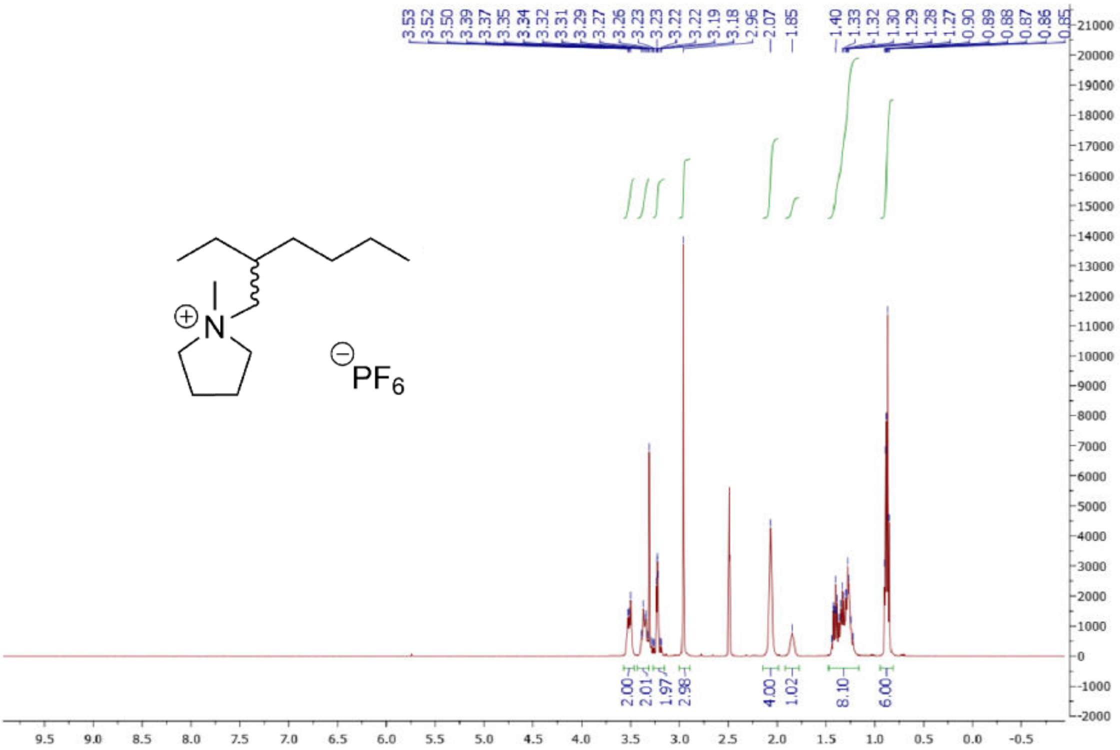

^{13}C NMR of compound **1aa** in $CDCl_3$

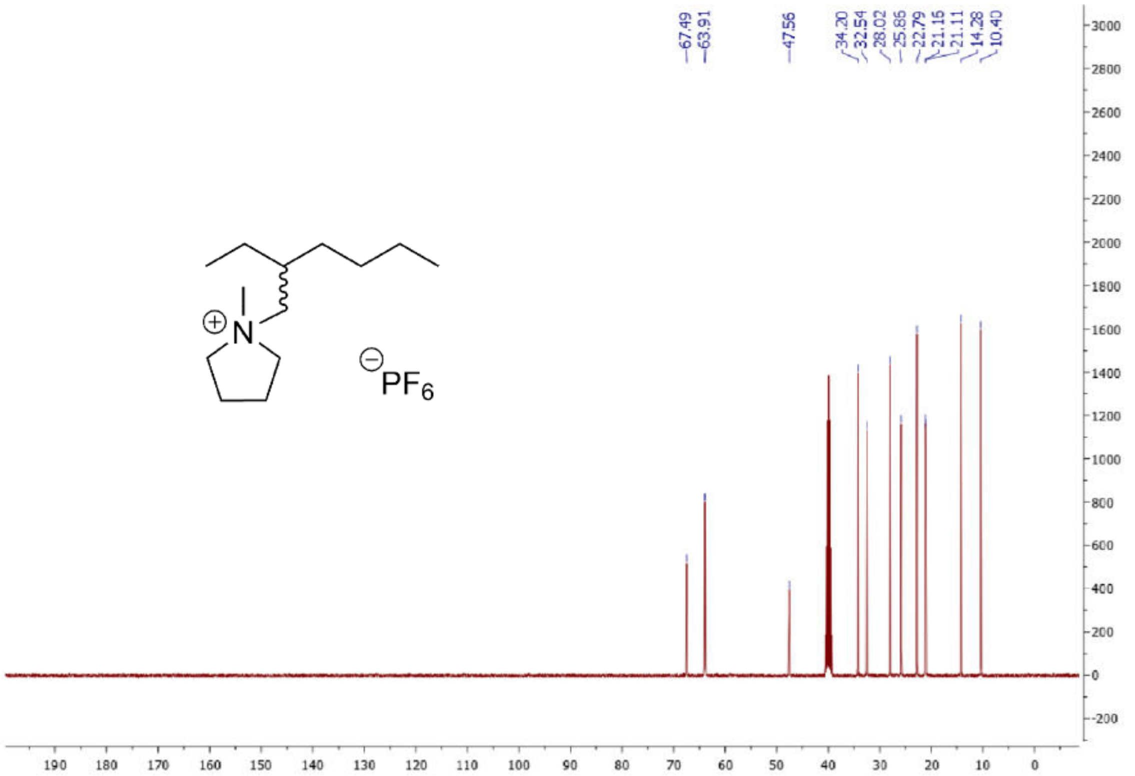

^{19}F NMR of compound **1aa** in $CDCl_3$

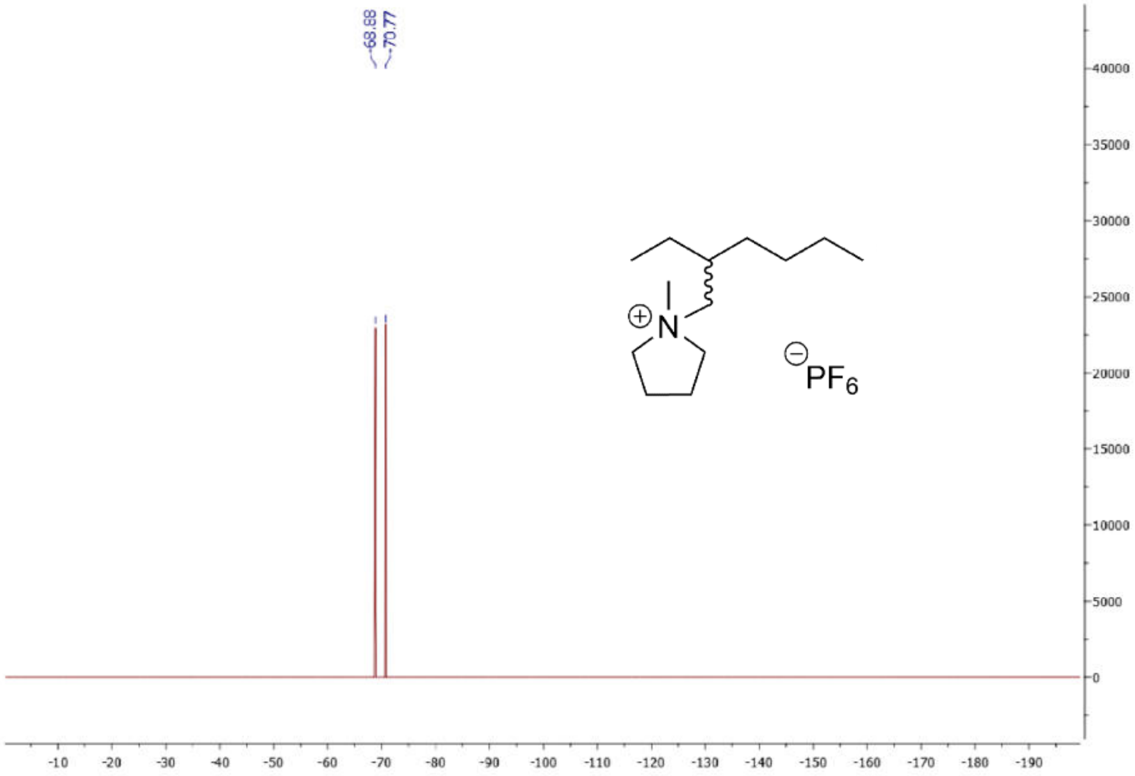

^{1}H NMR of compound **1ae** in $CDCl_3$

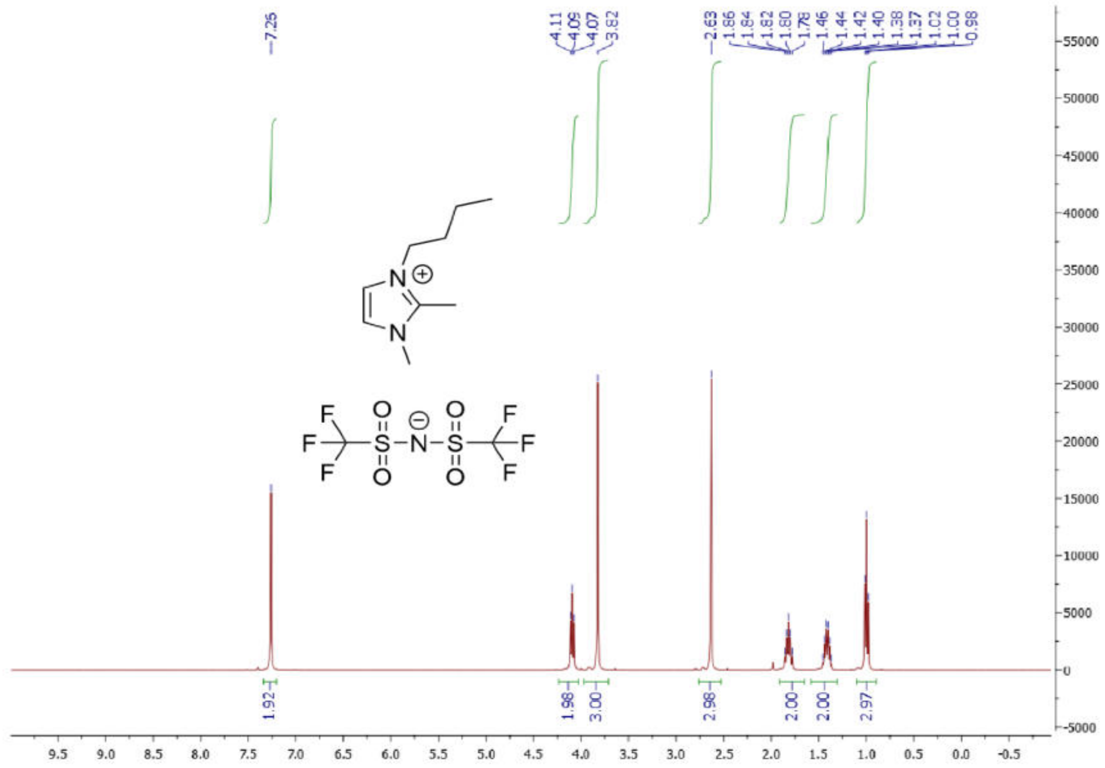

^{13}C NMR of compound **1ae** in $CDCl_3$

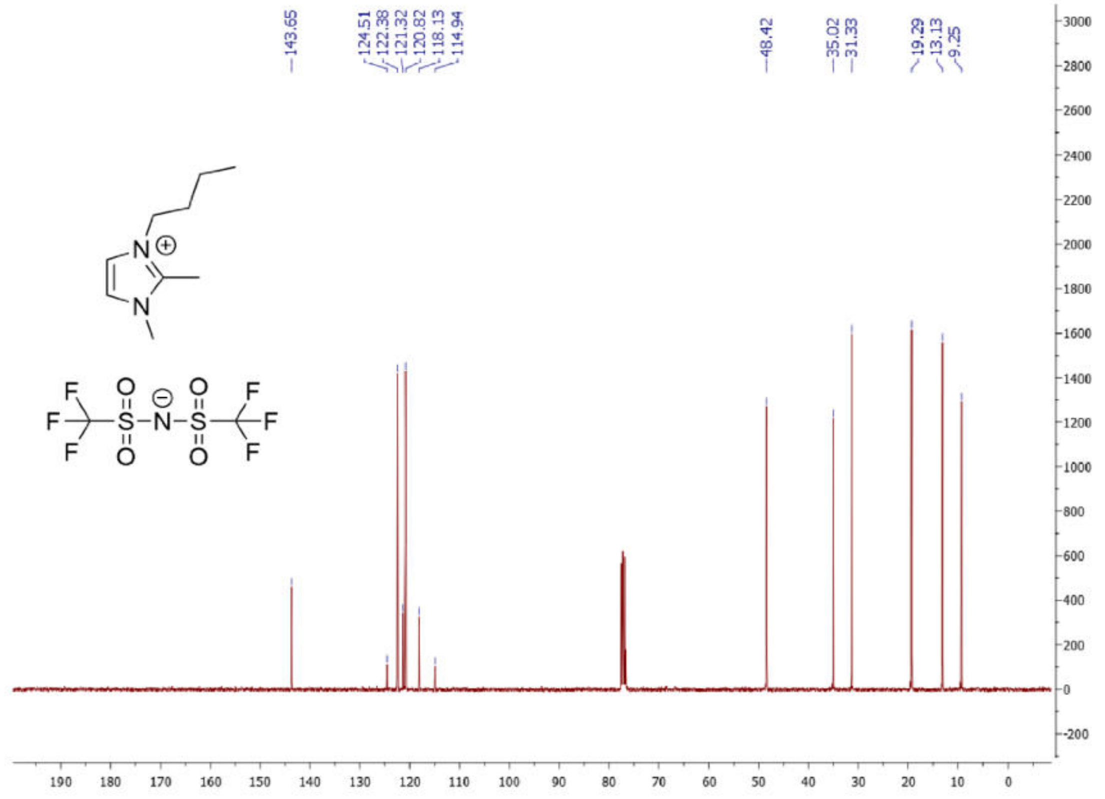

^{19}F NMR of compound **1ae** in $CDCl_3$

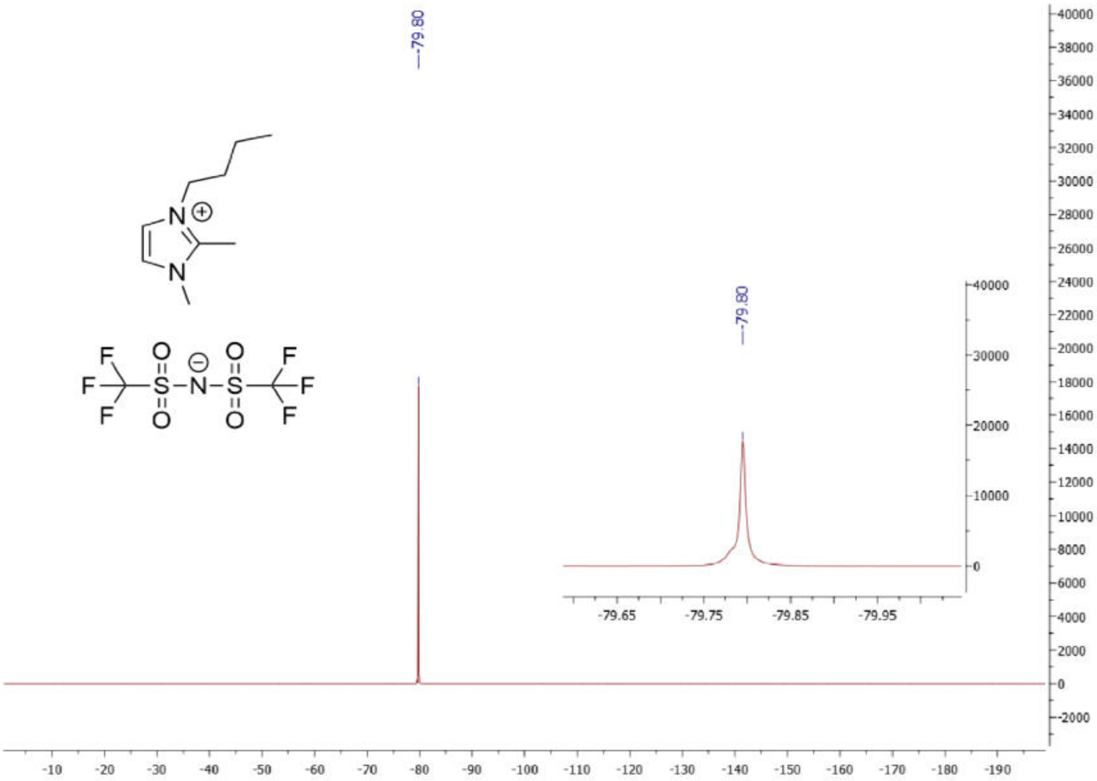

^{1}H NMR of compound **1ad** in $CDCl_3$

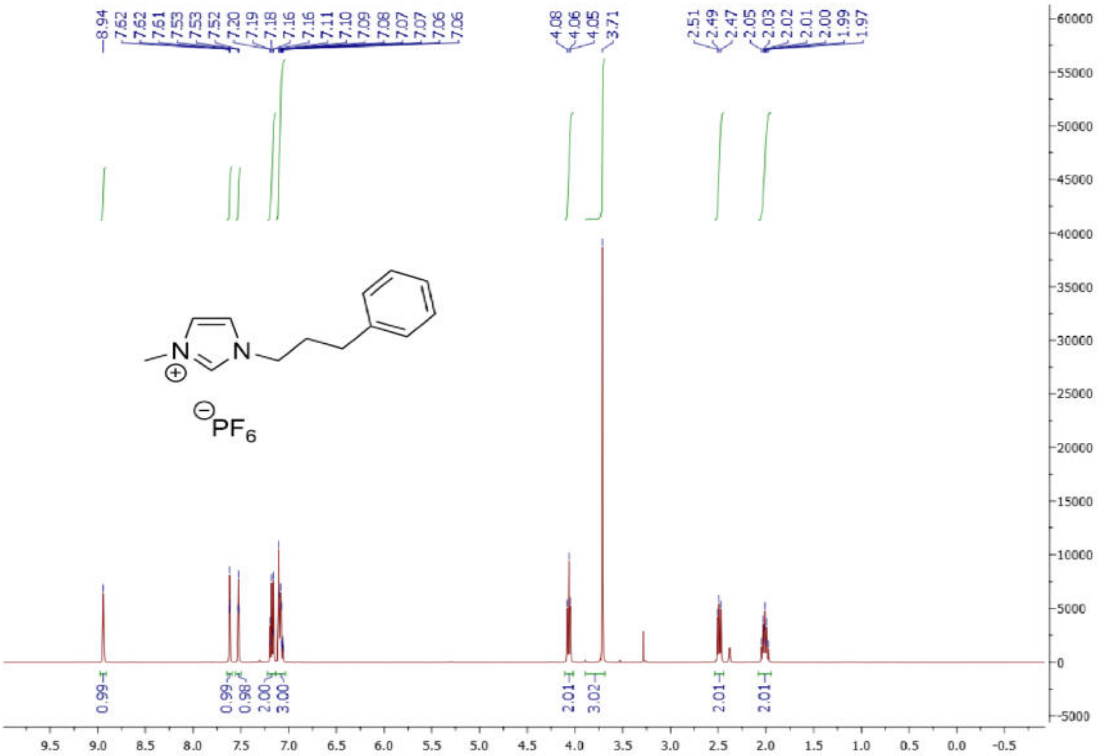

^{13}C NMR of compound **1ad** in $CDCl_3$

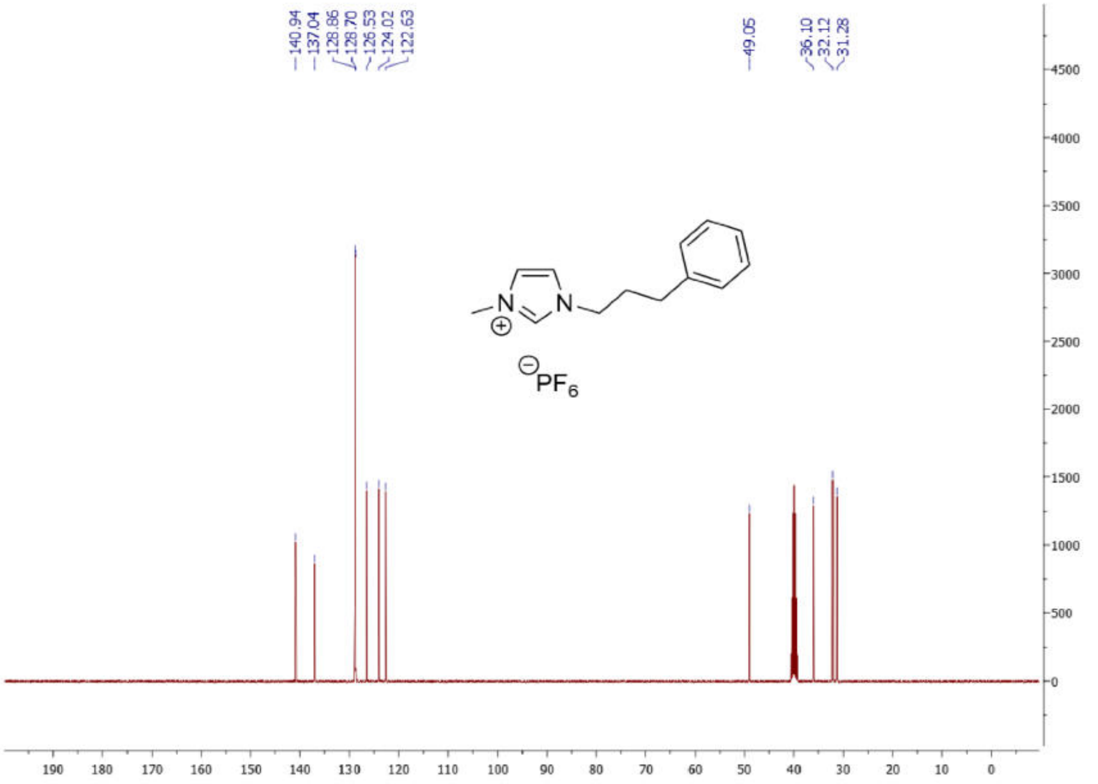

^{19}F NMR of compound **1ad** in $CDCl_3$

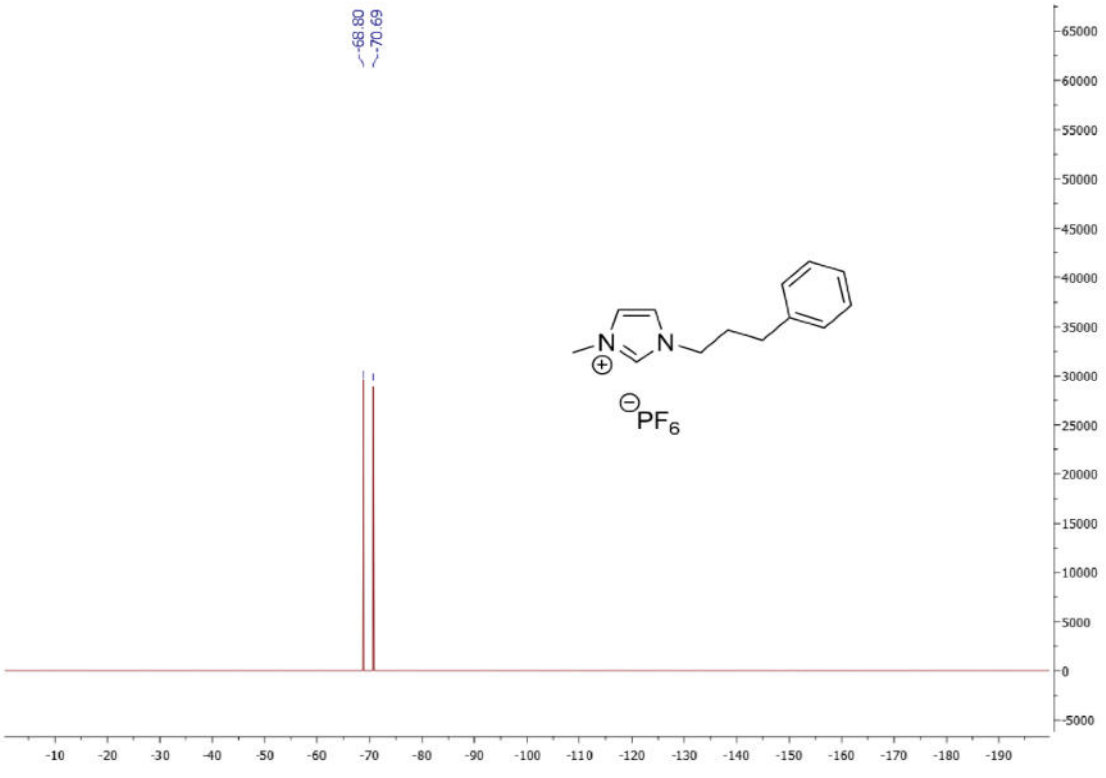

^{31}P NMR of compound **1ad** in $CDCl_3$

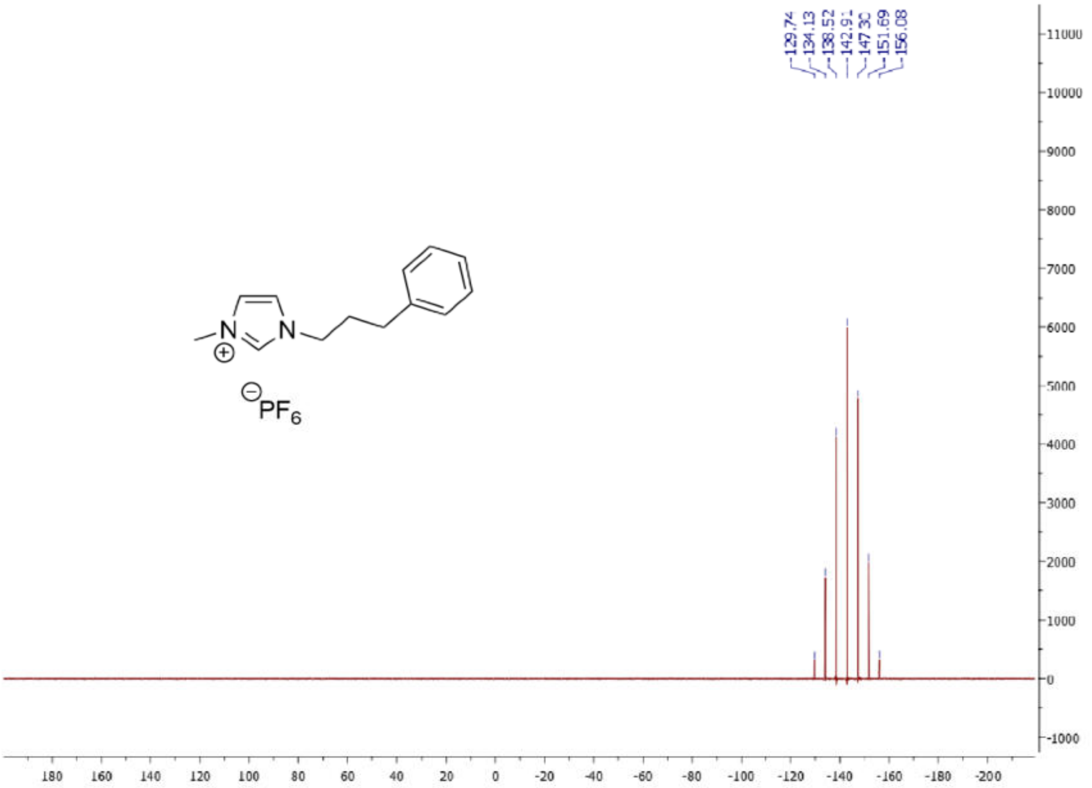

^{1}H NMR of compound **1ac** in $CDCl_3$

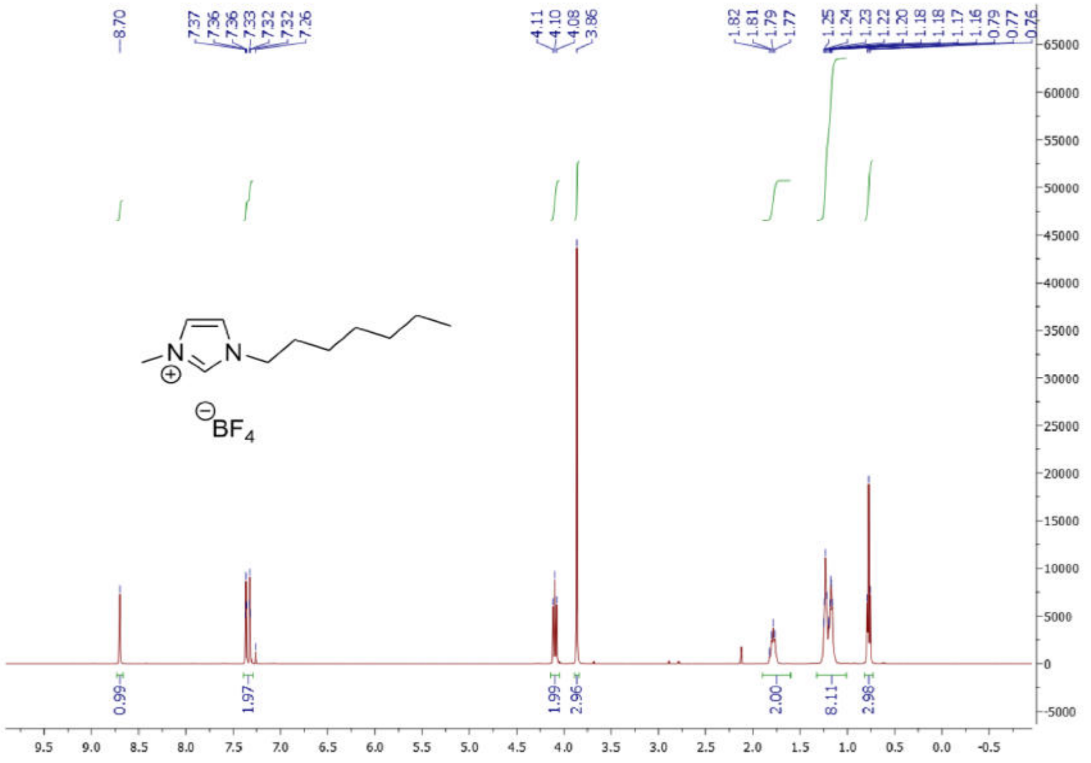

^{13}C NMR of compound **1ac** in $CDCl_3$

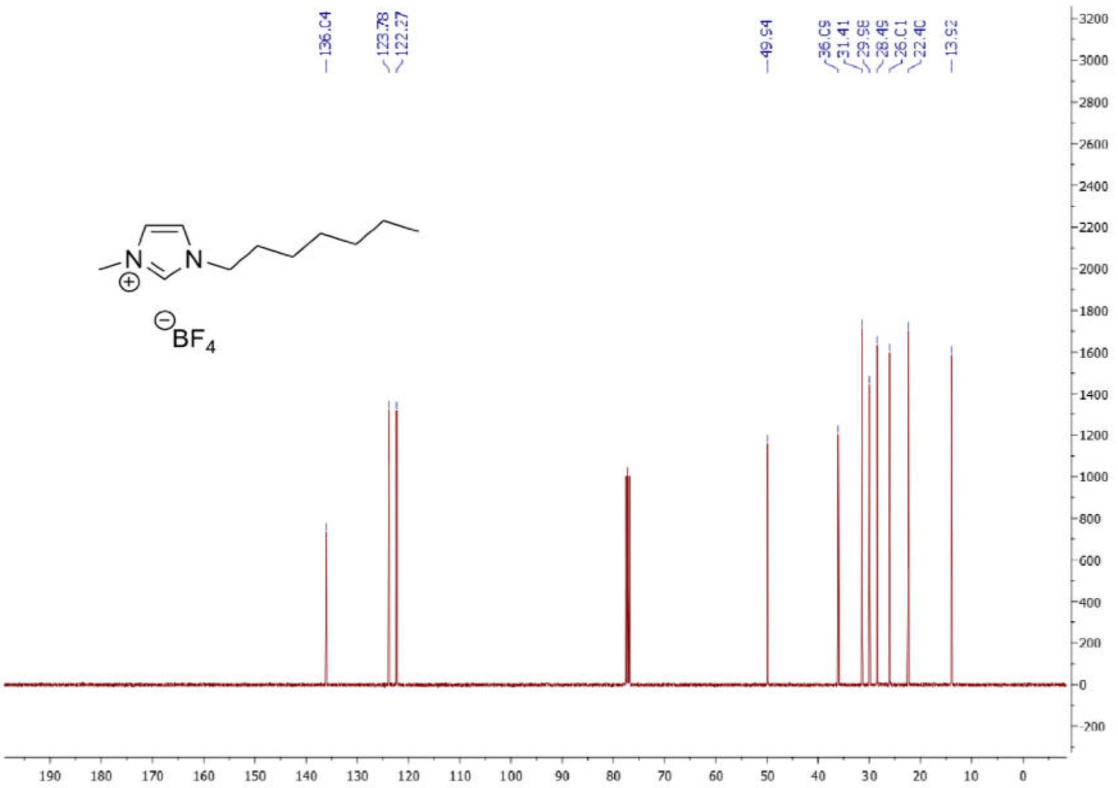

^{19}F NMR of compound **1ac** in $CDCl_3$

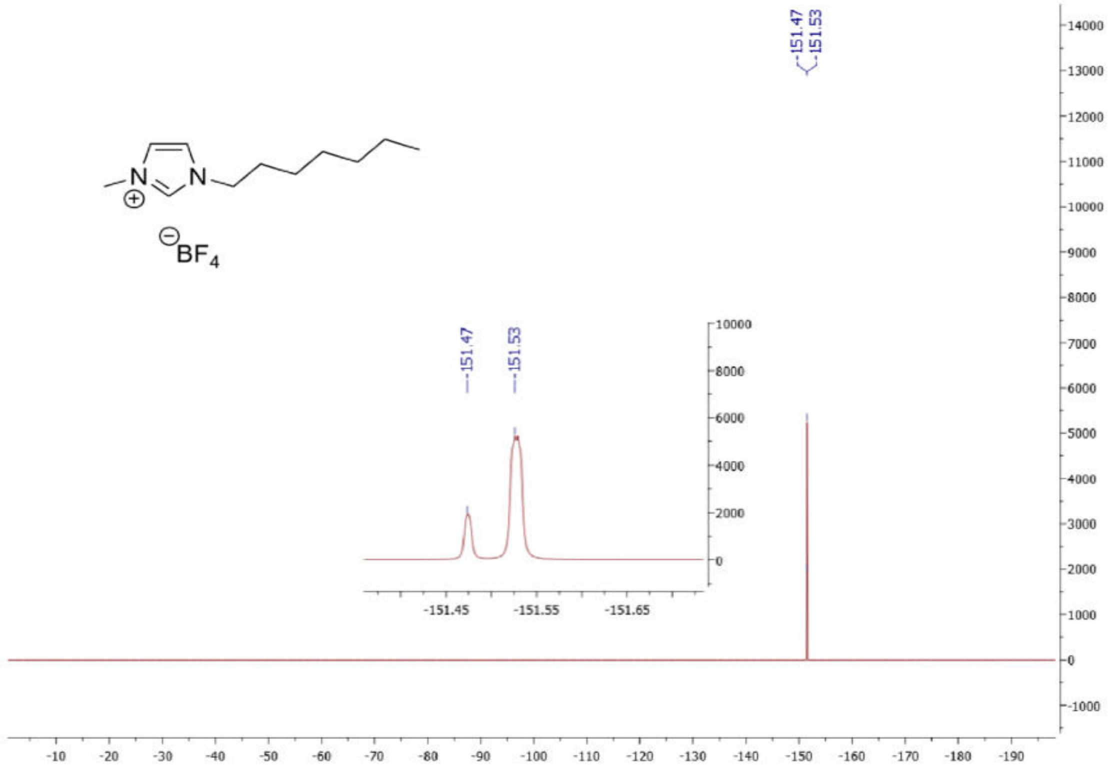

^{1}H NMR of compound **8i** in $CDCl_3$

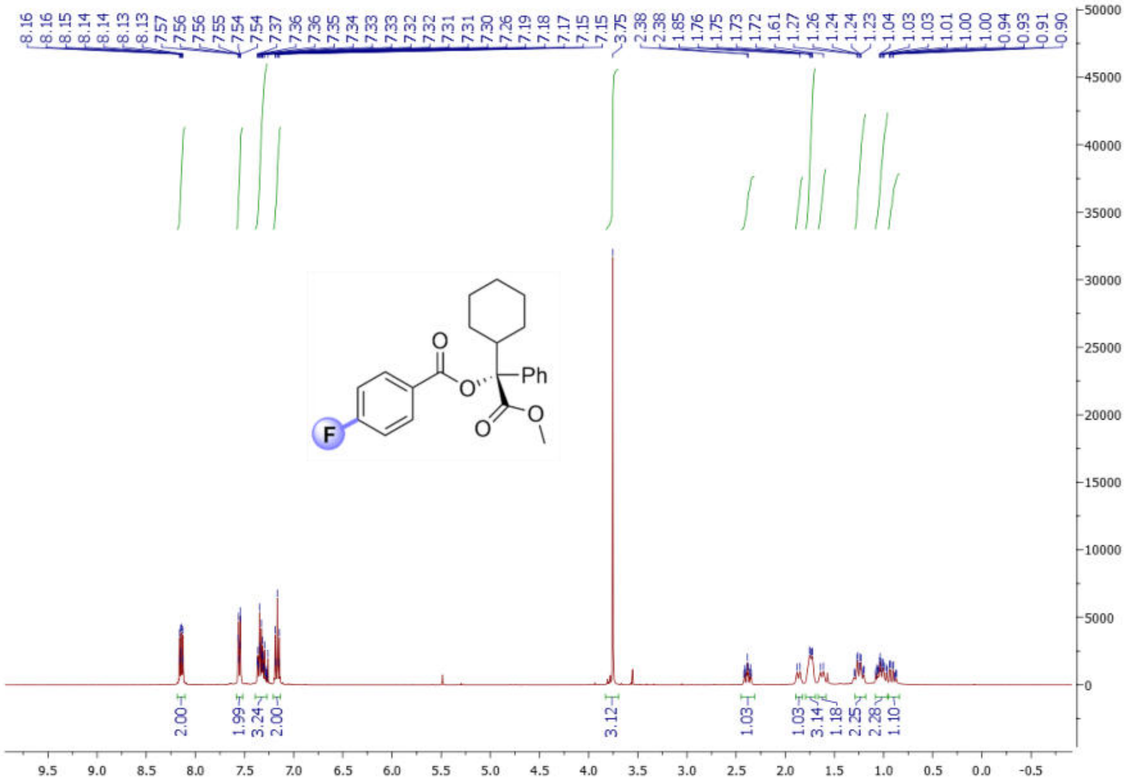

^{13}C NMR of compound **8i** in $CDCl_3$

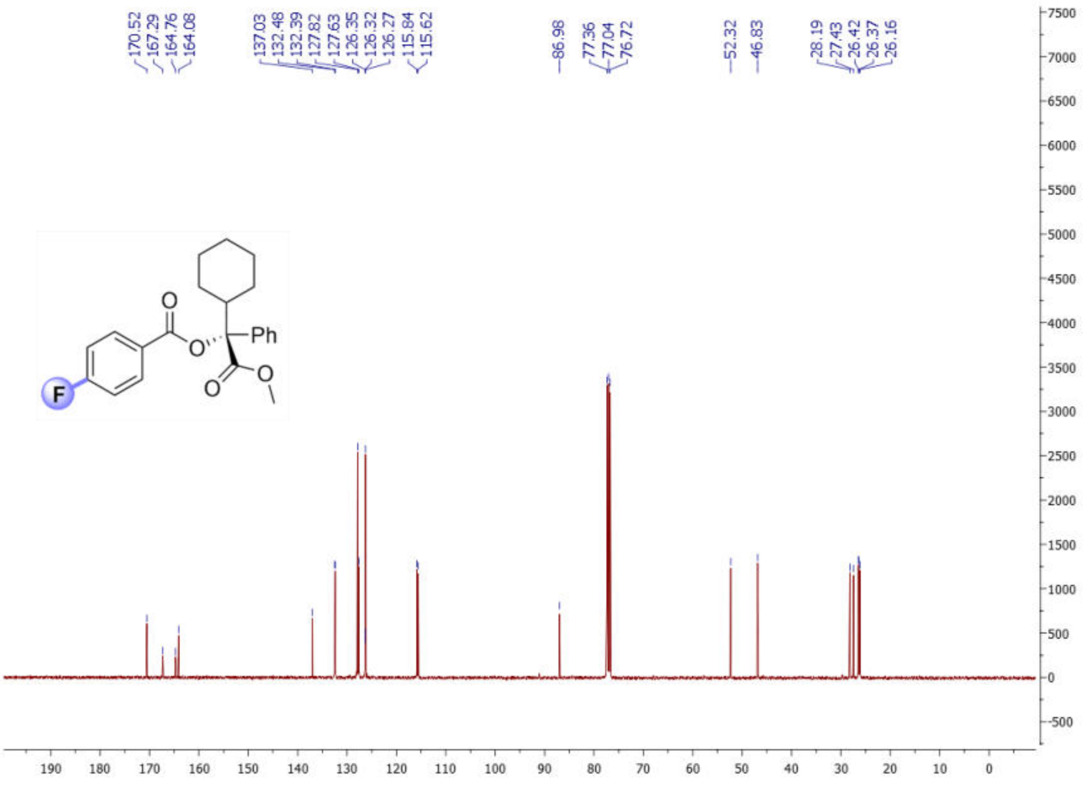

^{19}F NMR of compound **8i** in $CDCl_3$

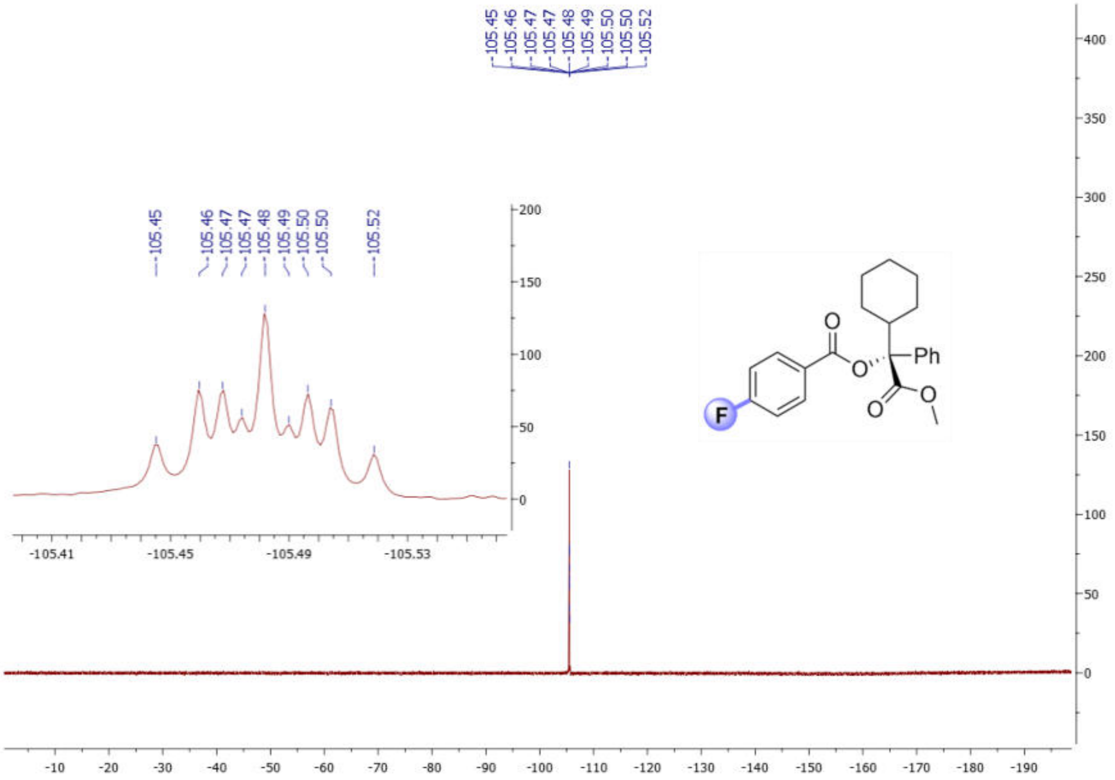

^{1}H NMR of compound **8a** in $CDCl_3$

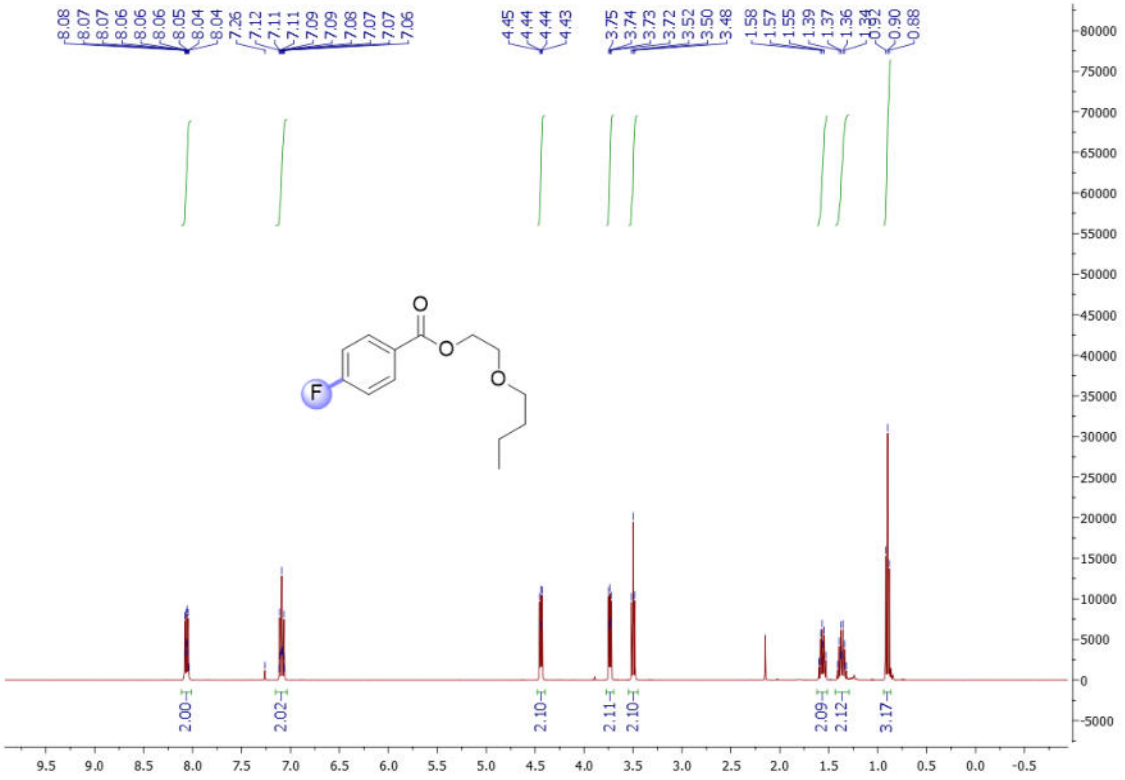

^{13}C NMR of compound **8a** in $CDCl_3$

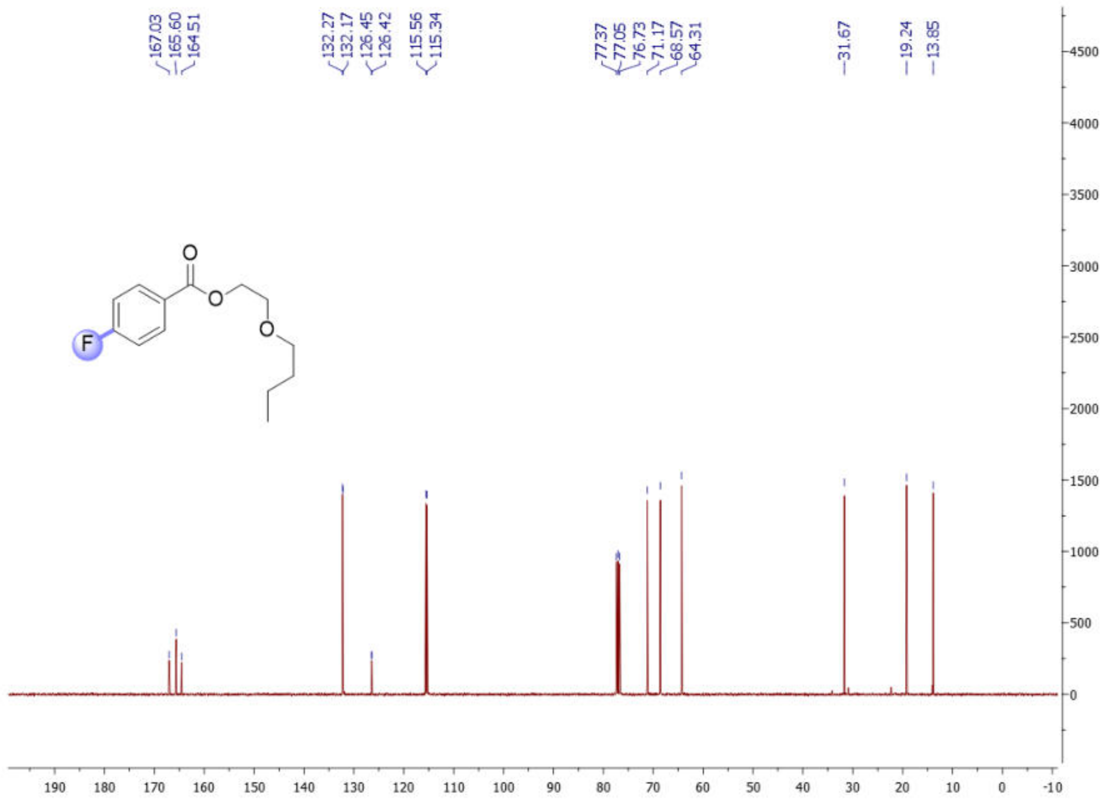

^{1}H NMR of compound **8b** in $CDCl_3$

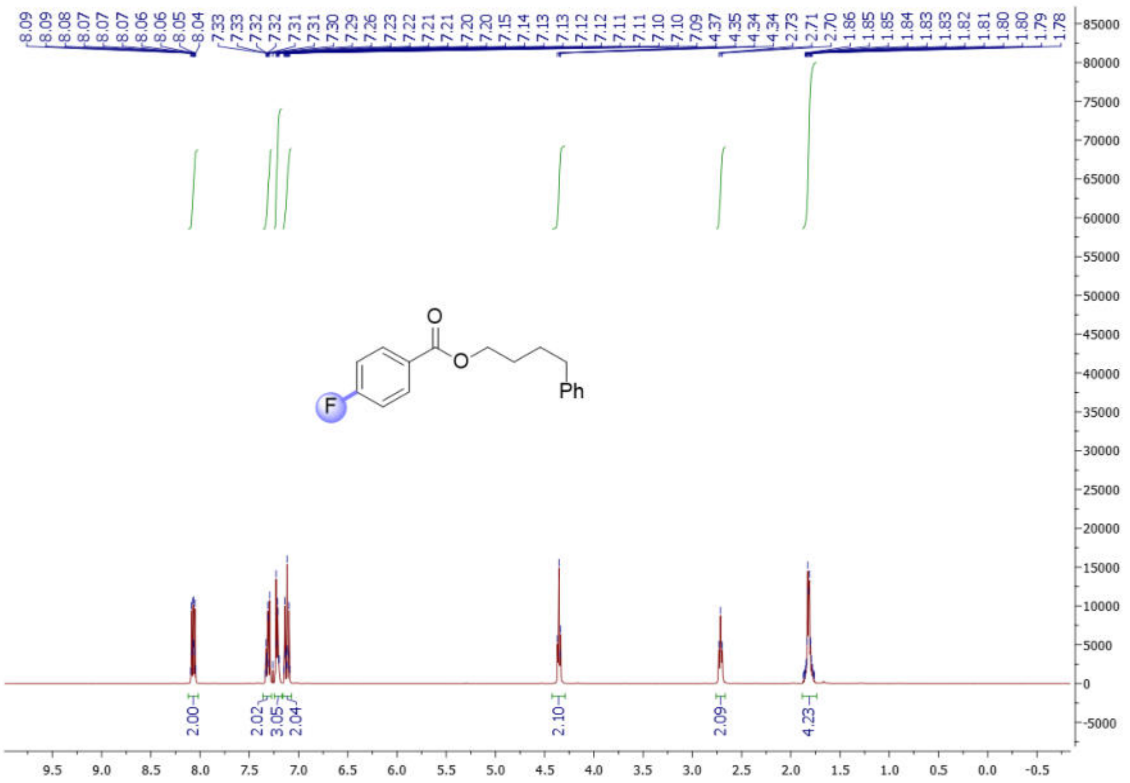

^{13}C NMR of compound **8b** in $CDCl_3$

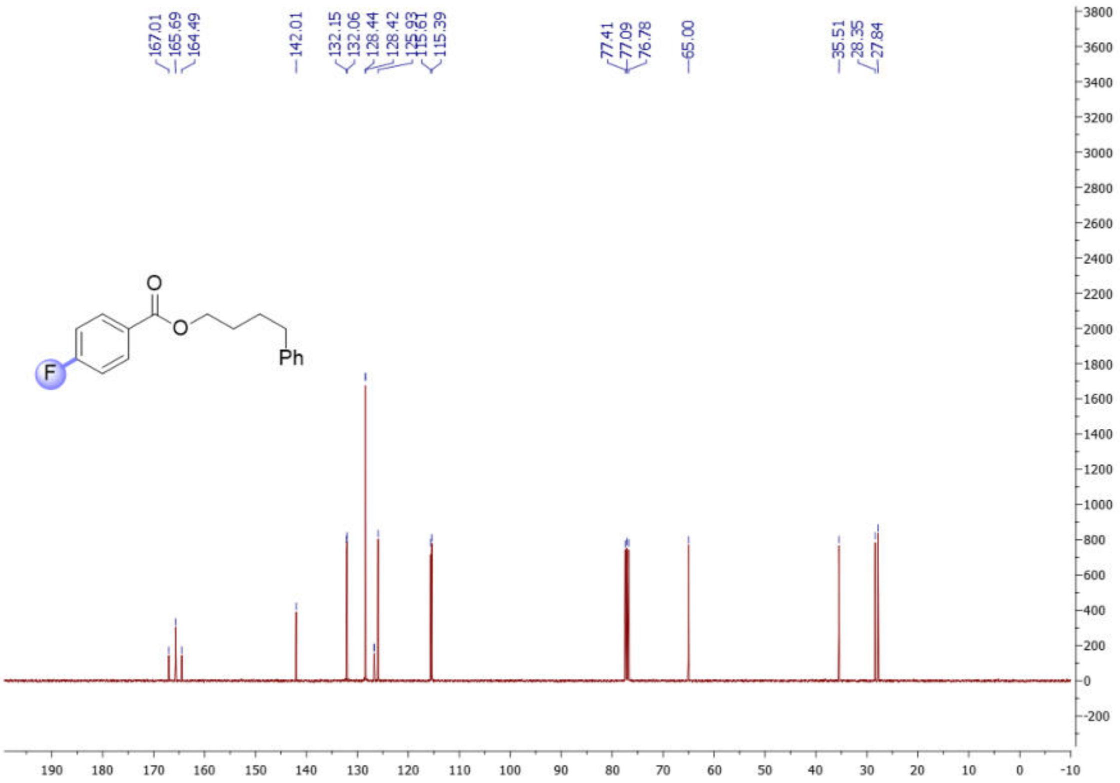

^{19}F NMR of compound **8b** in $CDCl_3$

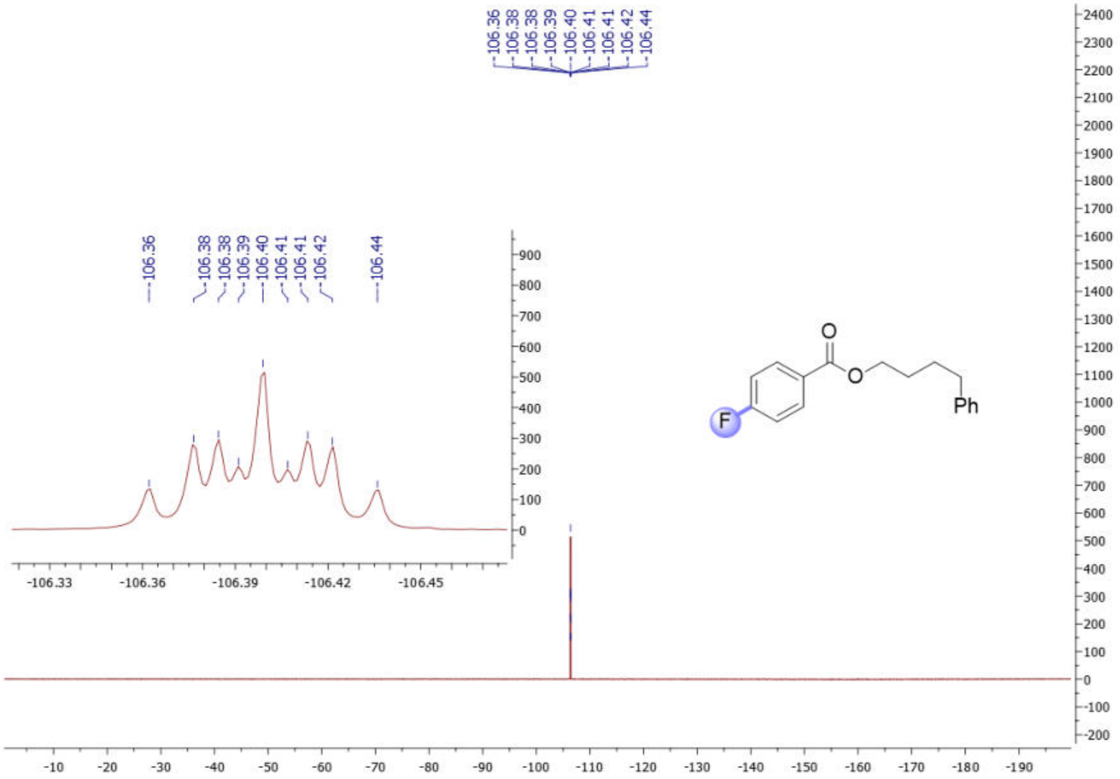

^{1}H NMR of compound **8c** in $CDCl_3$

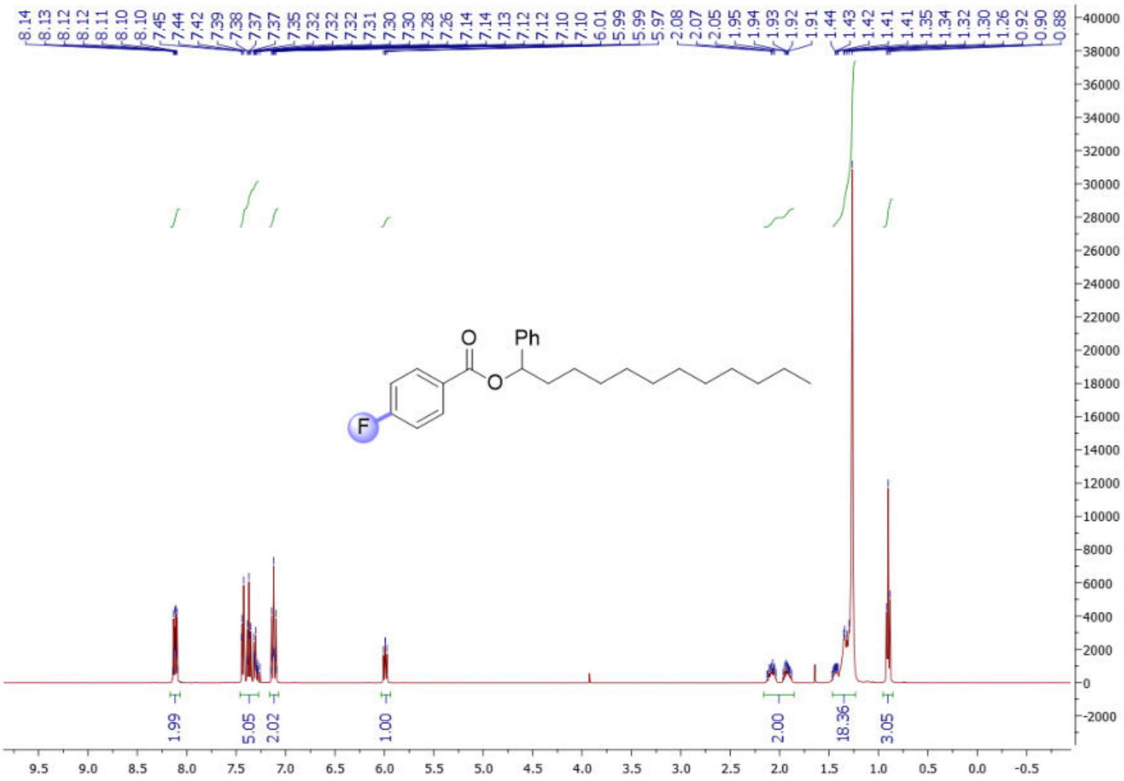

^{13}C NMR of compound **8c** in $CDCl_3$

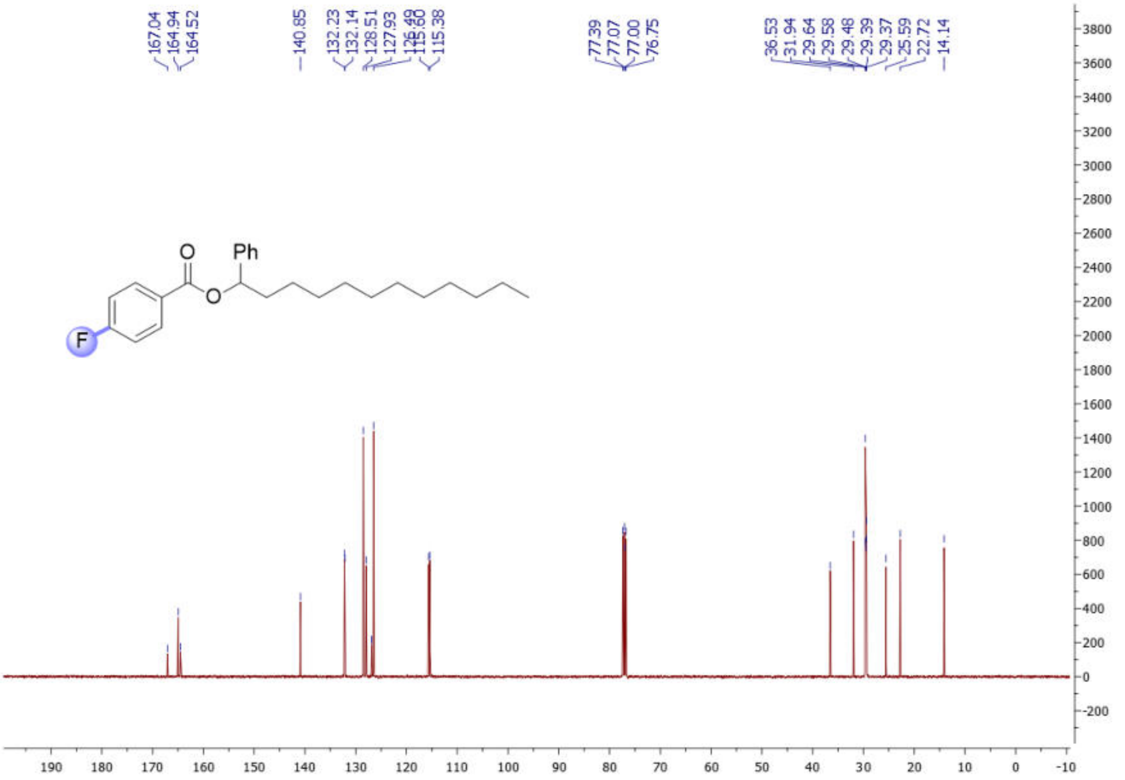

^{19}F NMR of compound **8c** in $CDCl_3$

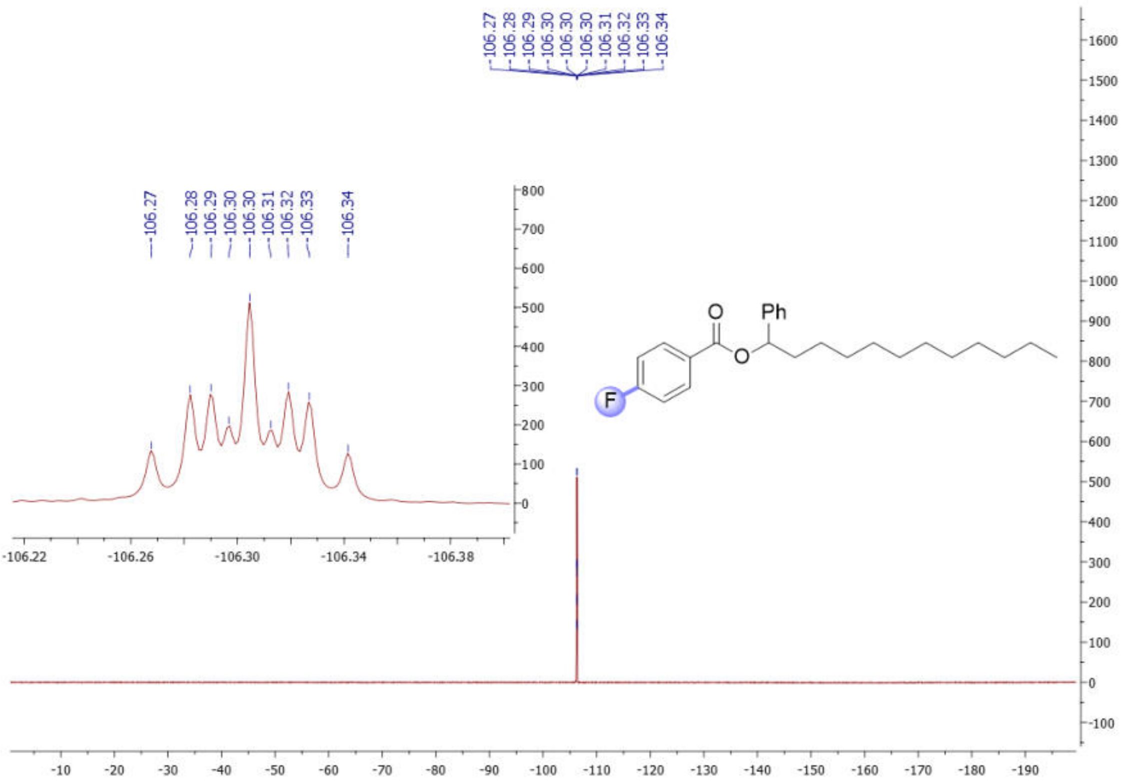

^{1}H NMR of compound **8cc** in $CDCl_3$

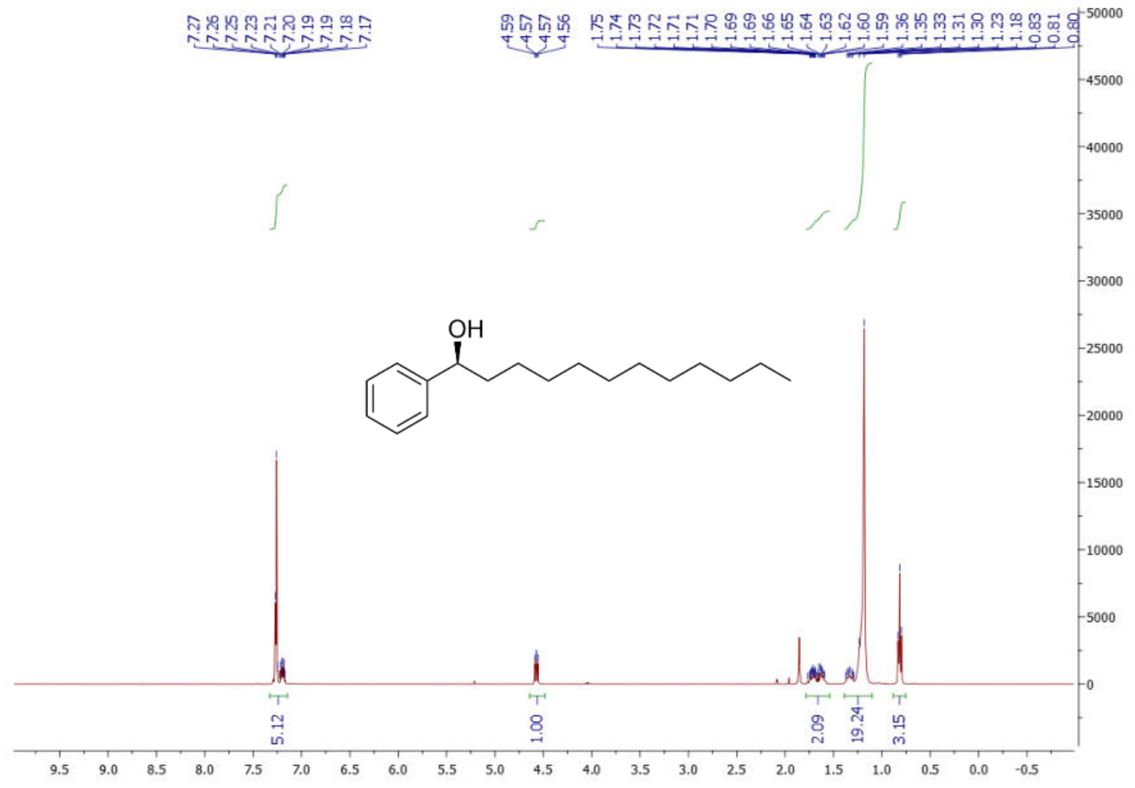

^{13}C NMR of compound **8cc** in $CDCl_3$

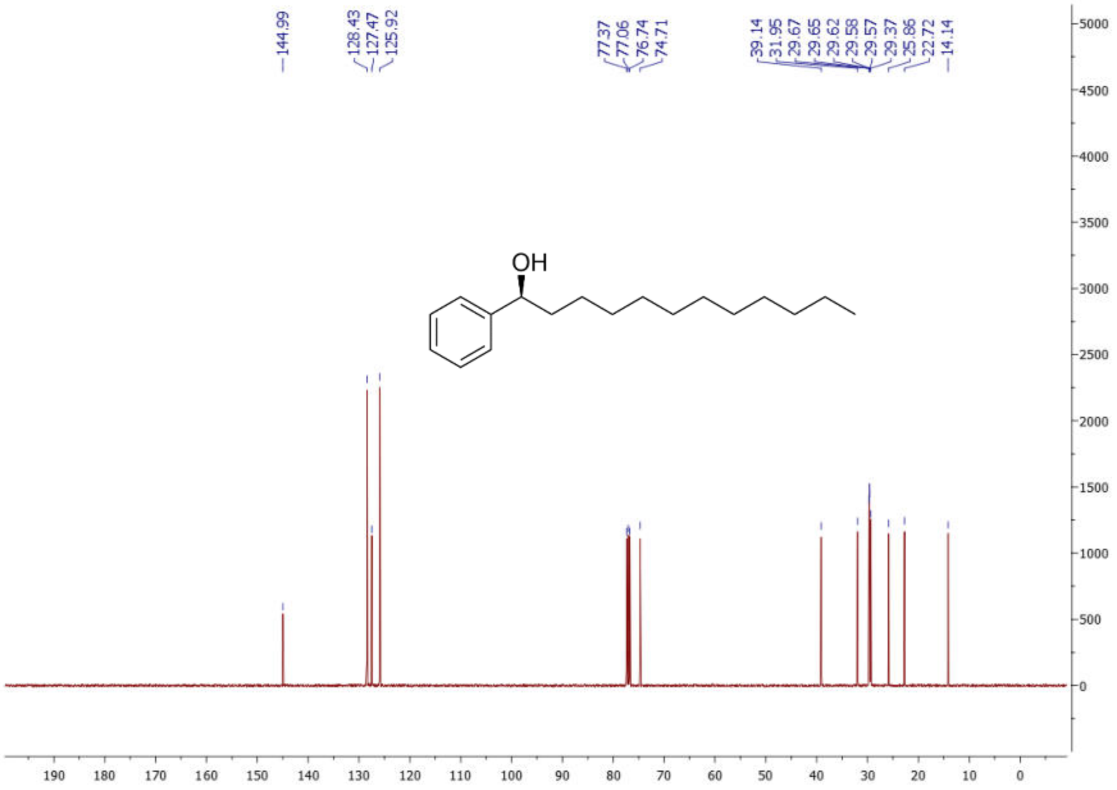

^{1}H NMR of compound **8d** in $CDCl_3$

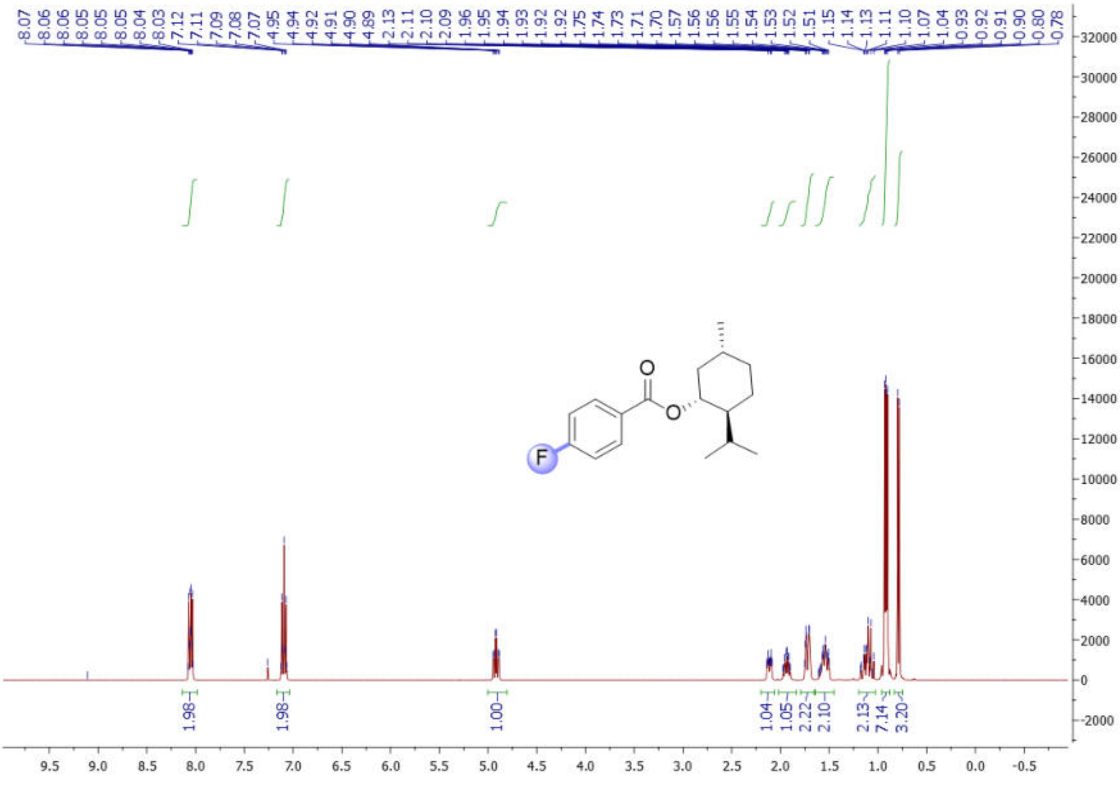

^{13}C NMR of compound **8d** in $CDCl_3$

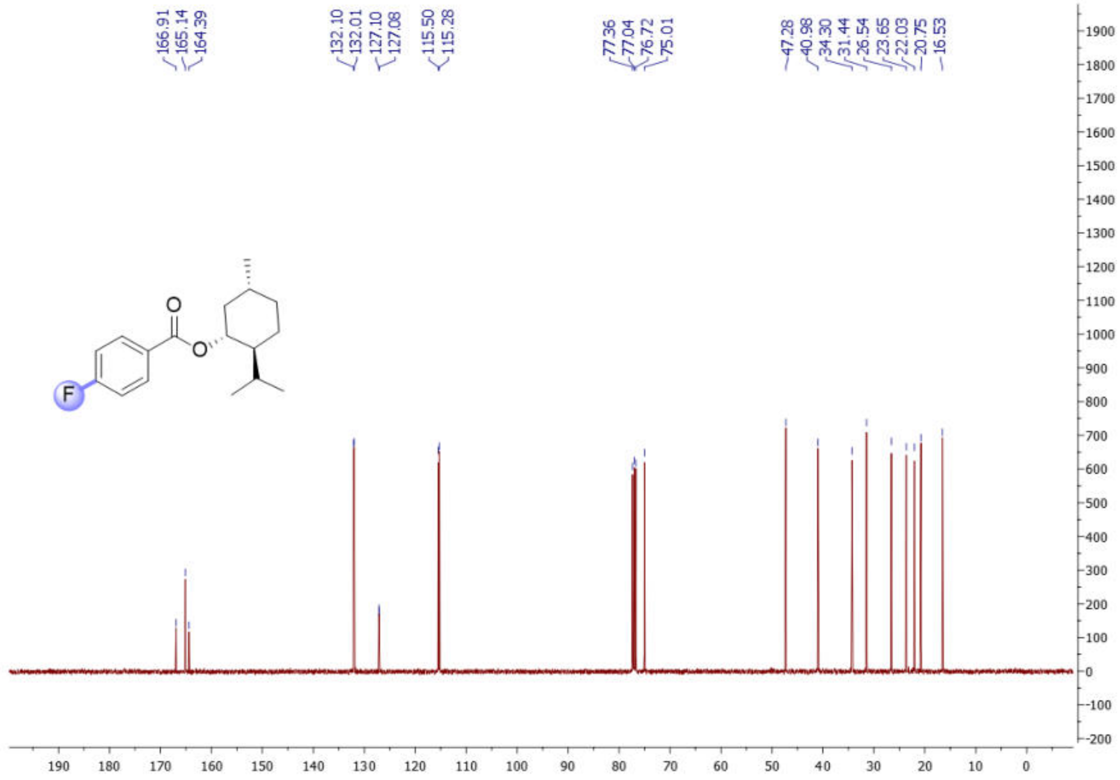

^{19}F NMR of compound **8d** in $CDCl_3$

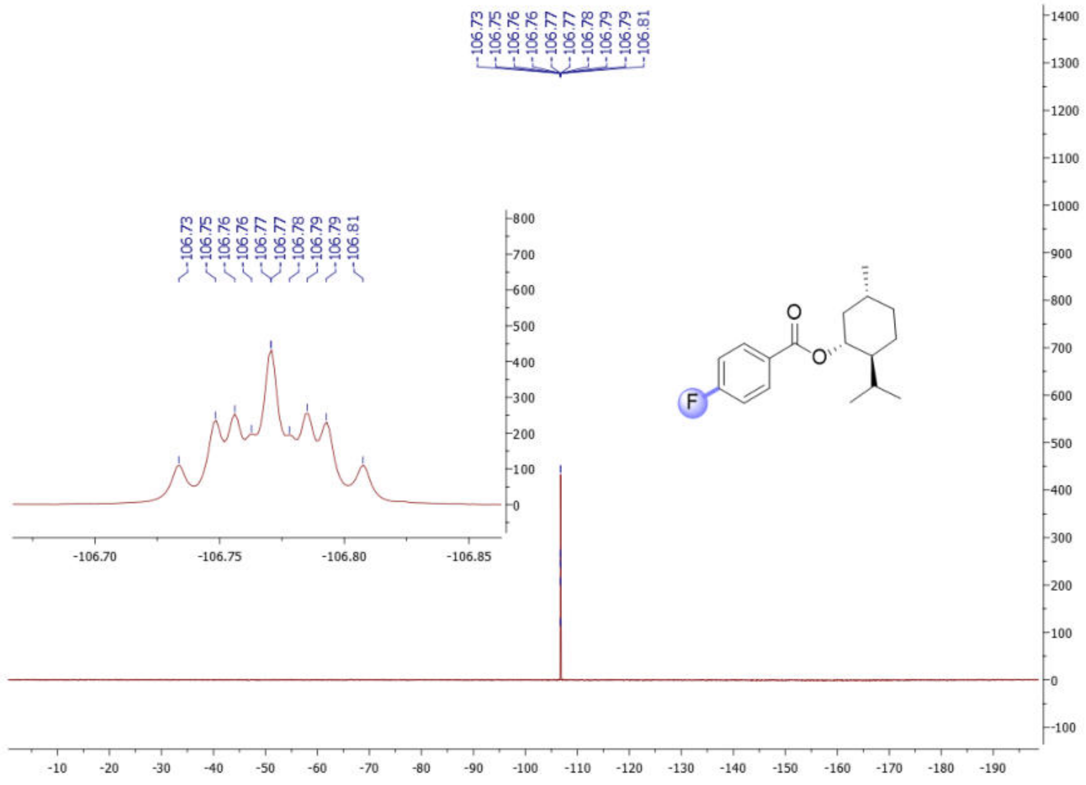

^1H NMR of compound **8e** in $CDCl_3$

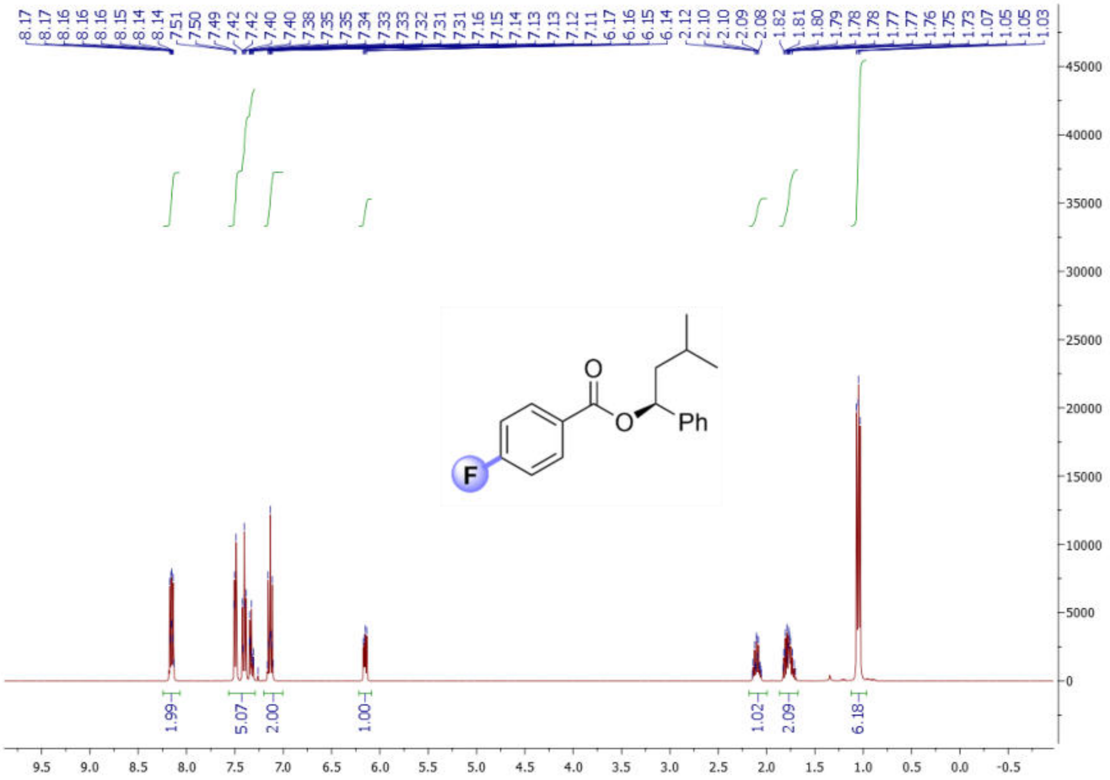

^{13}C NMR of compound **8e** in $CDCl_3$

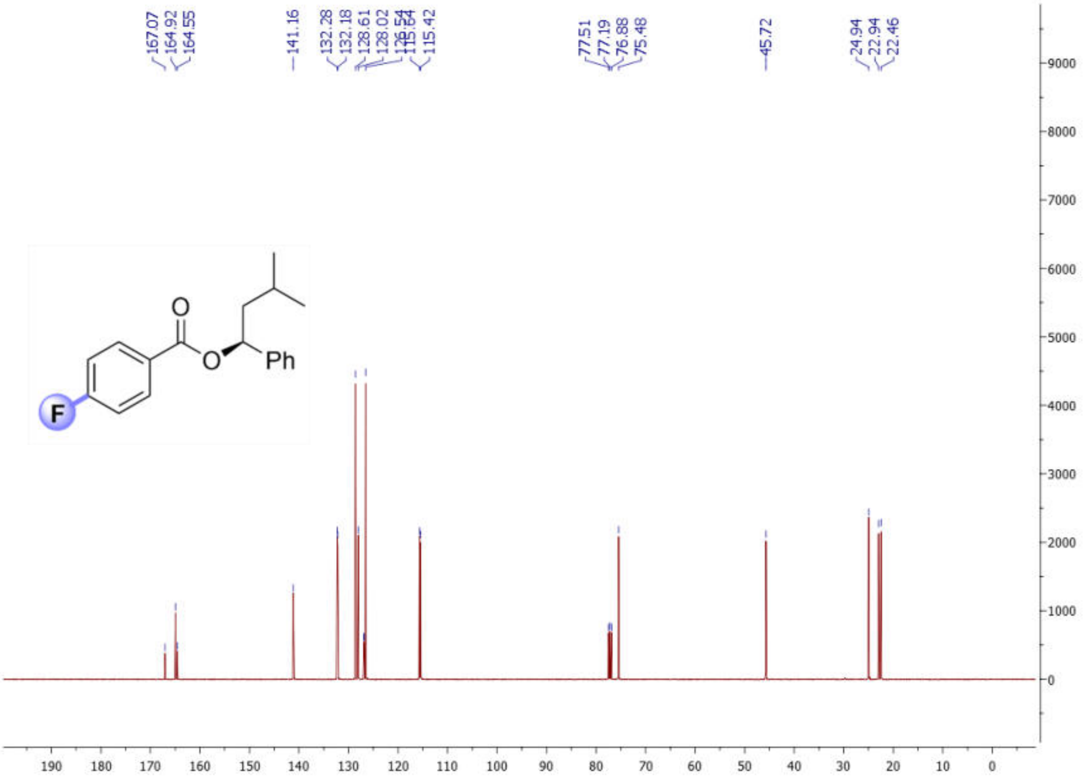

^{19}F NMR of compound **8e** in $CDCl_3$

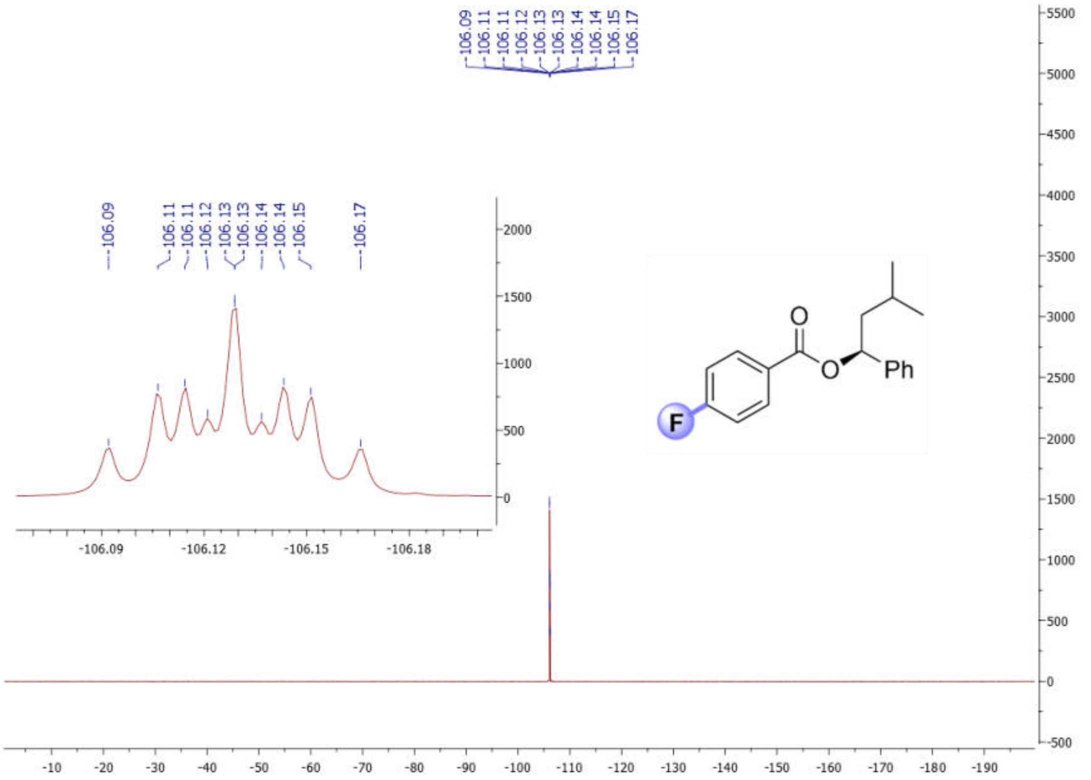

^{1}H NMR of compound **8ee** in $CDCl_3$

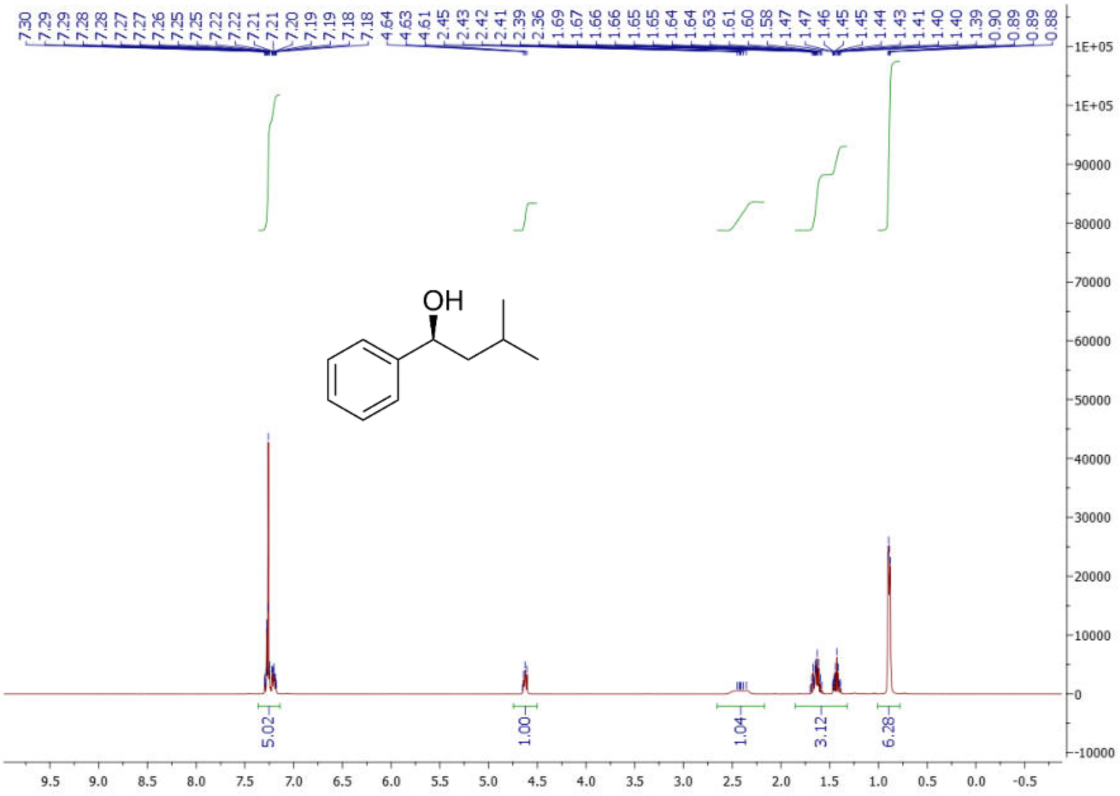

^{13}C NMR of compound **8ee** in $CDCl_3$

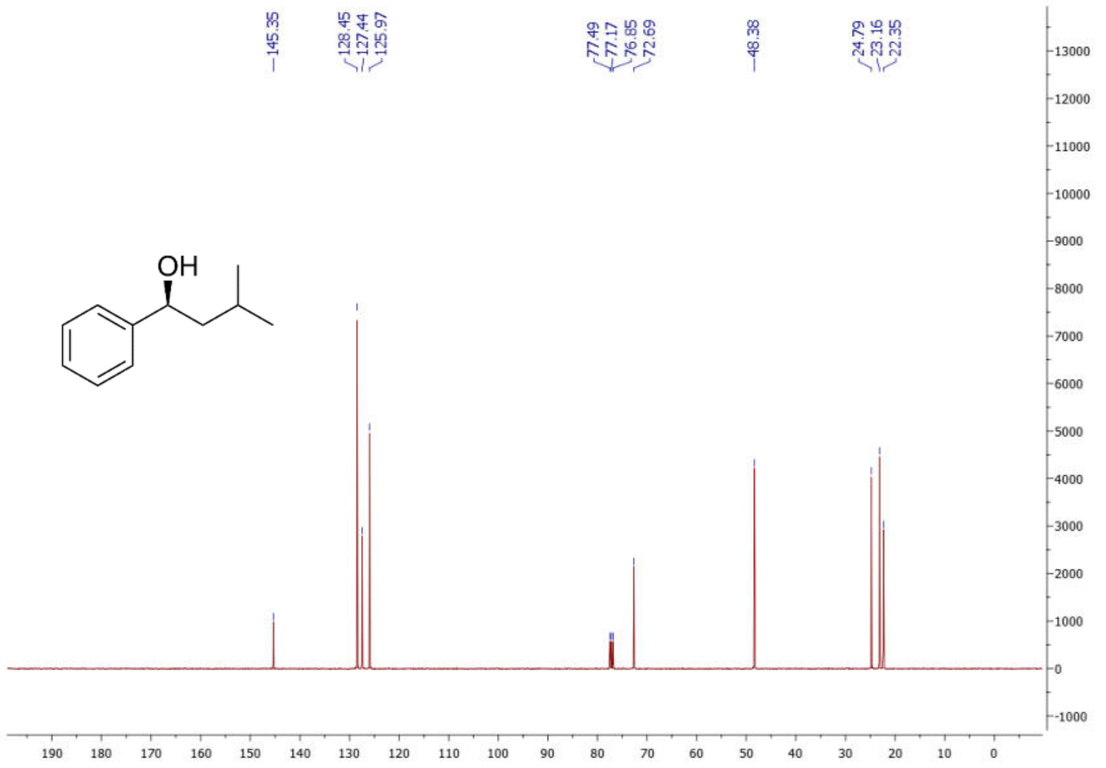

^{1}H NMR of compound **8f** in $CDCl_3$

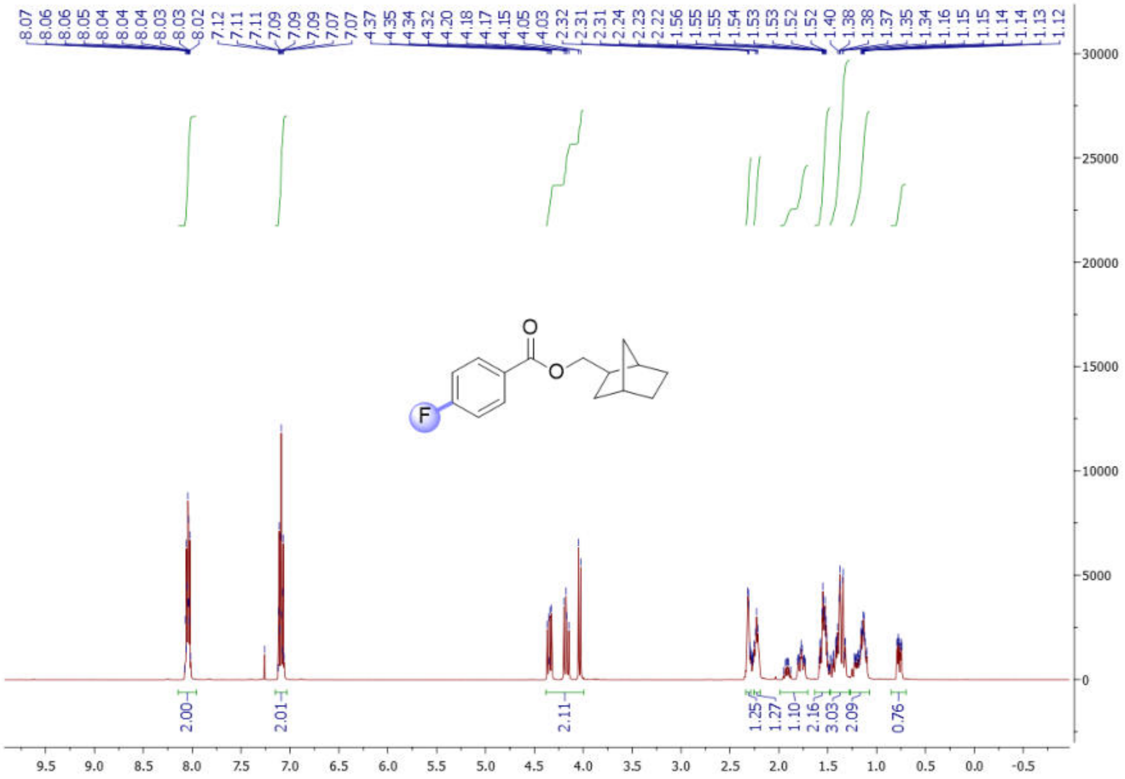

^{13}C NMR of compound **8f** in $CDCl_3$

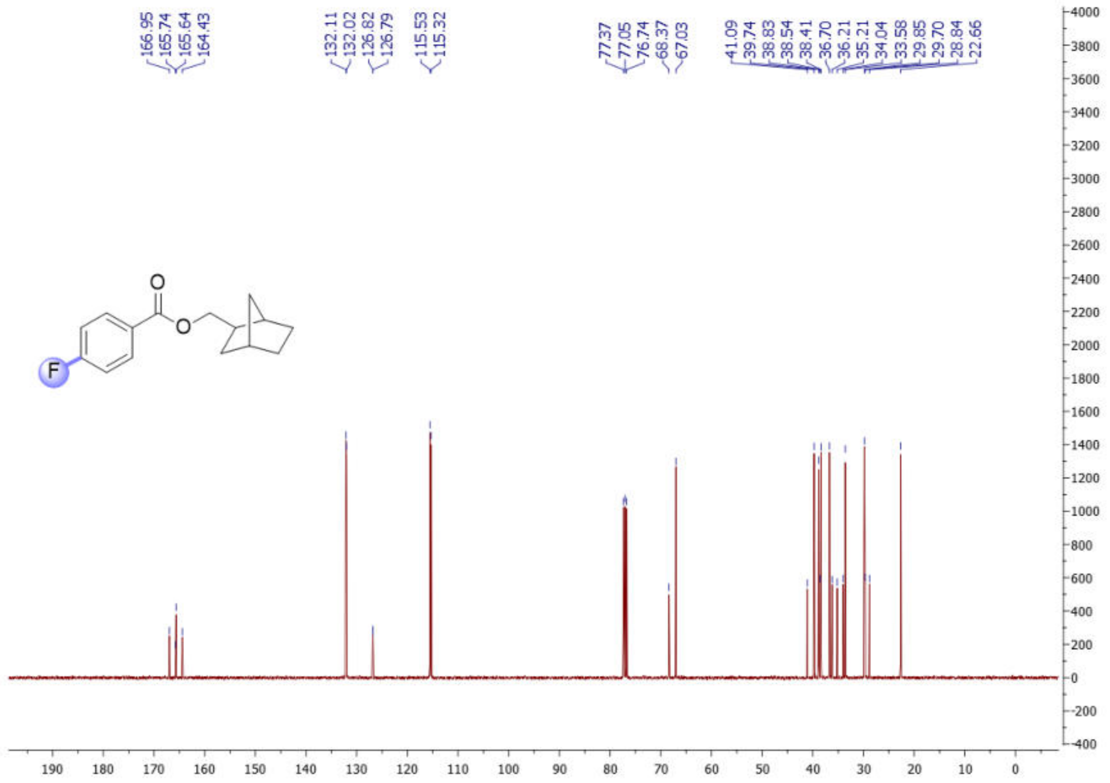

^{19}F NMR of compound **8f** in $CDCl_3$

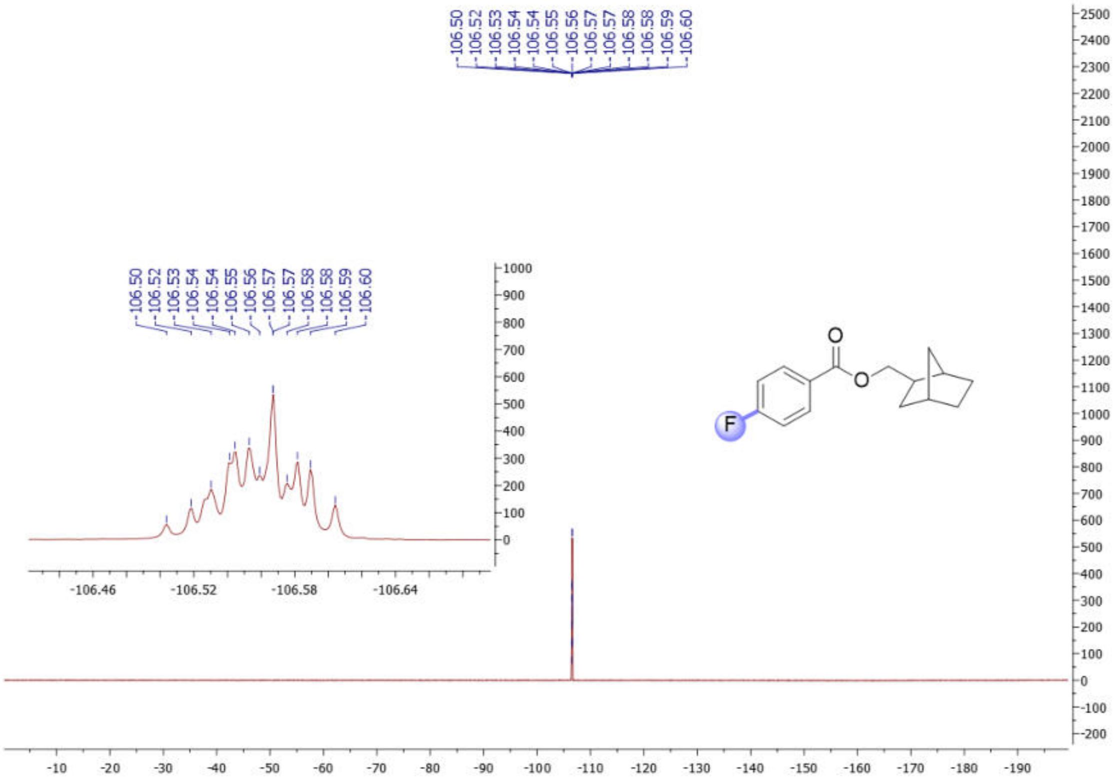

^{1}H NMR of compound **8g** in $CDCl_3$

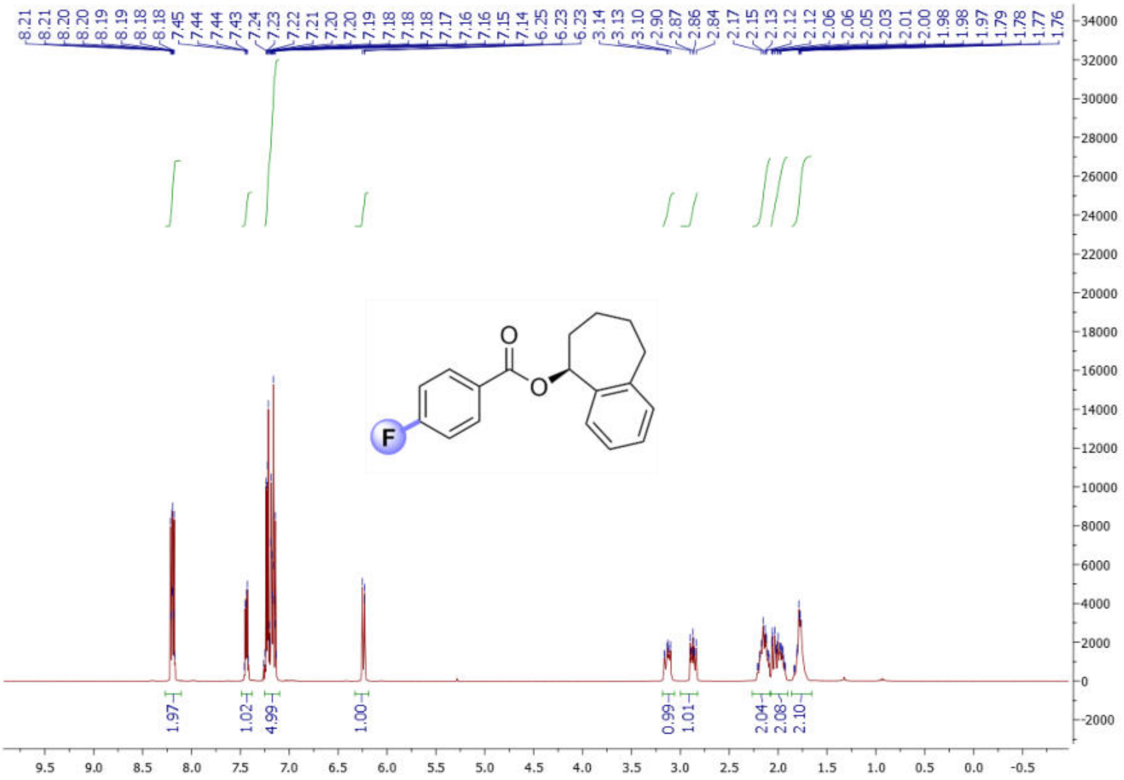

^{13}C NMR of compound **8g** in $CDCl_3$

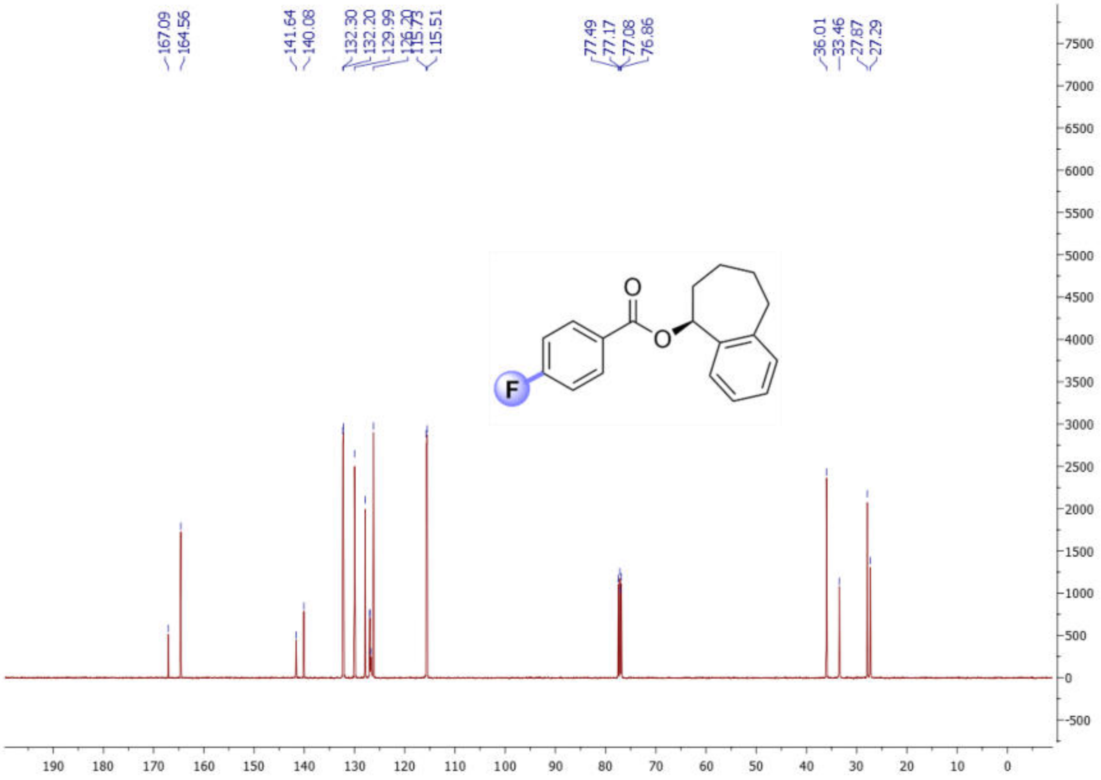

^{19}F NMR of compound **8g** in $CDCl_3$

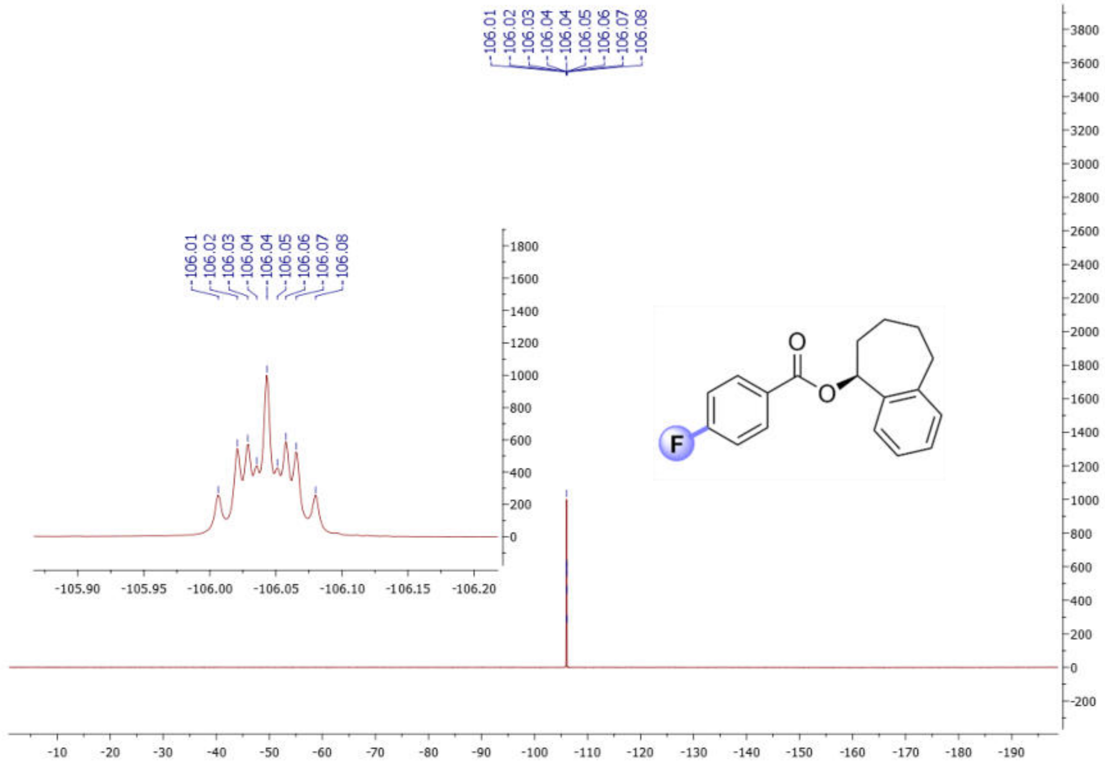

^{1}H NMR of compound **8h** in $CDCl_3$

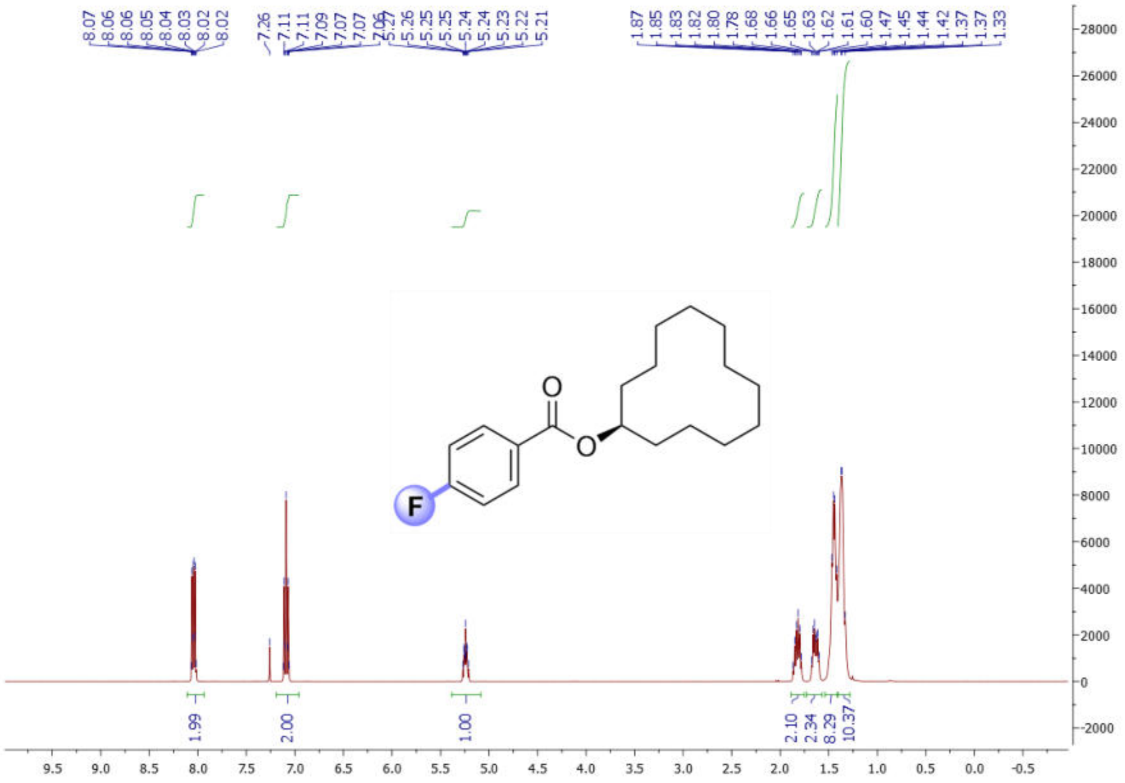

^{13}C NMR of compound **8h** in $CDCl_3$

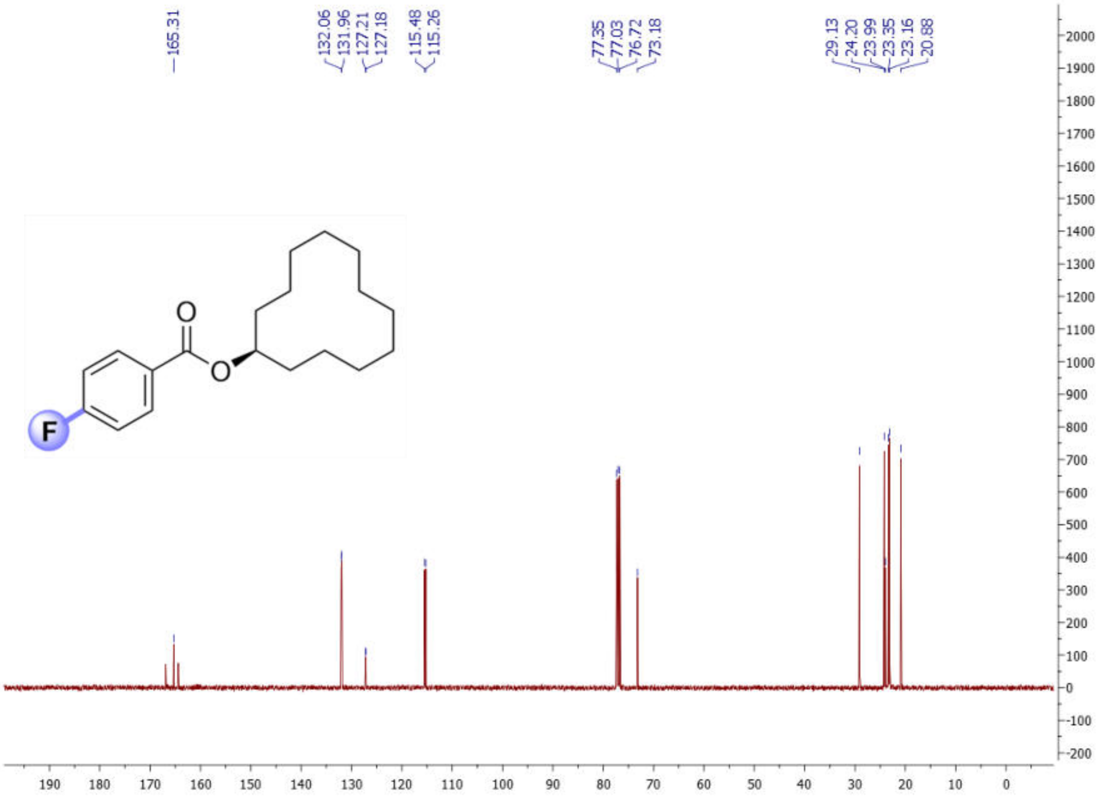

^{19}F NMR of compound **8h** in $CDCl_3$

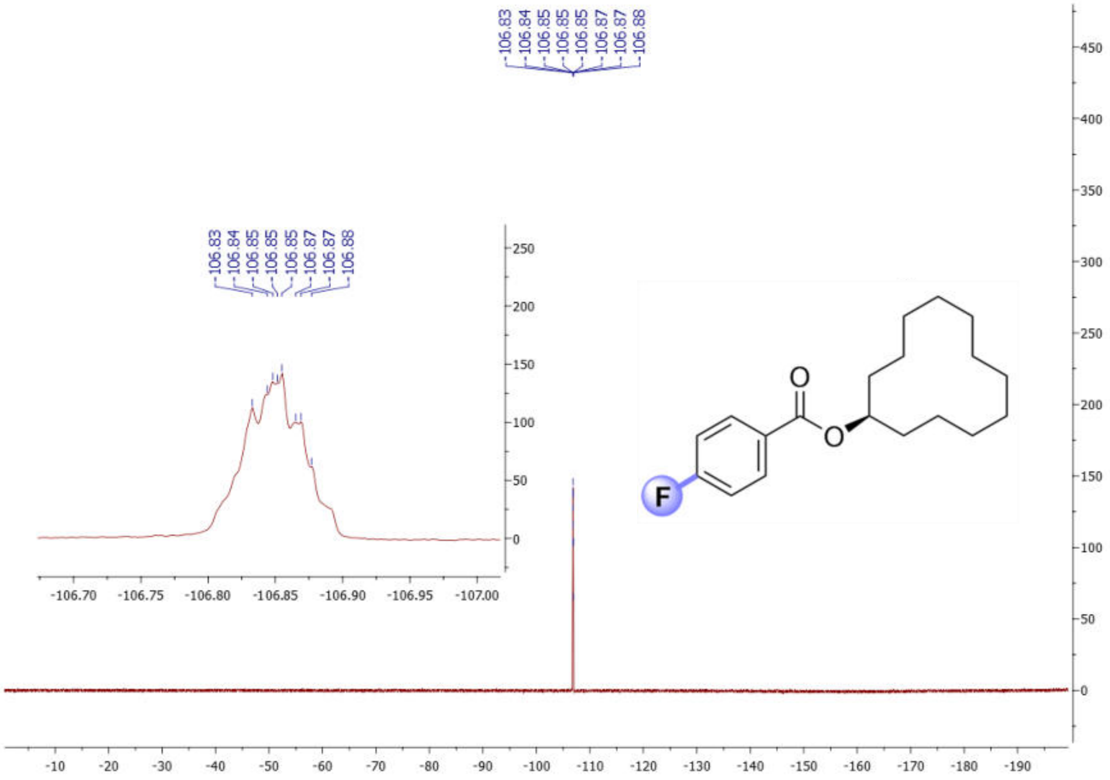

^{1}H NMR of compound **8j** in $CDCl_3$

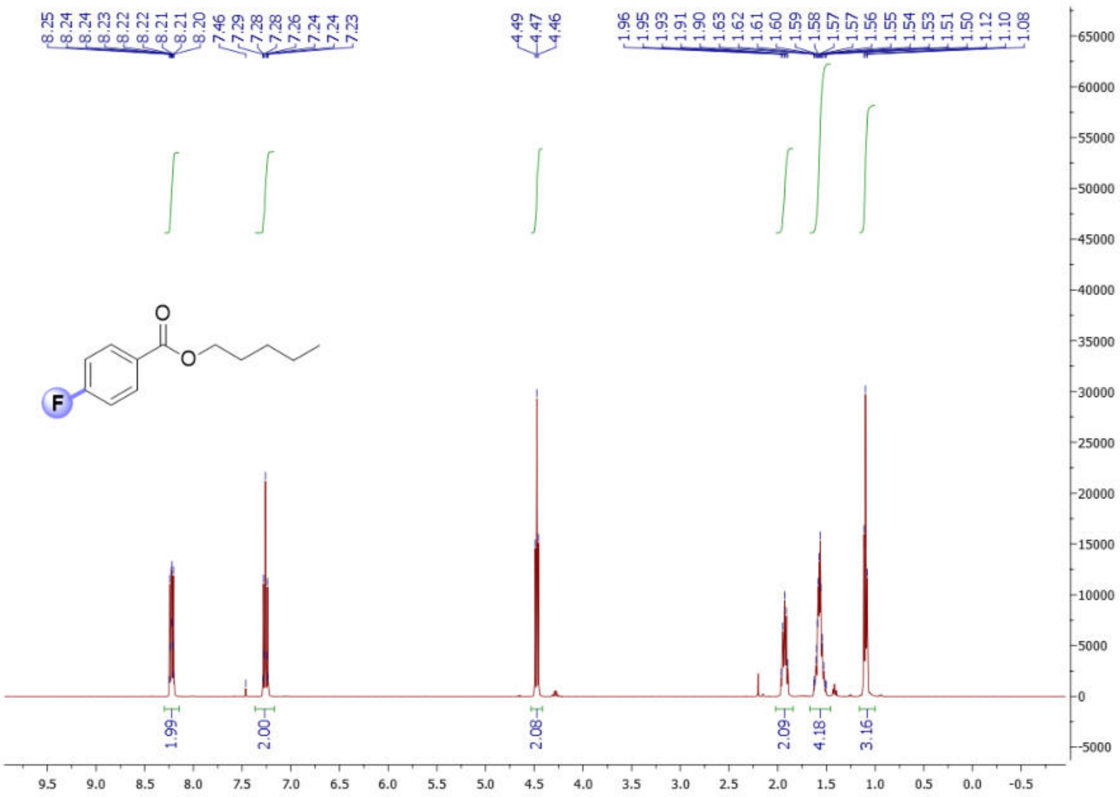

^{13}C NMR of compound **8j** in $CDCl_3$

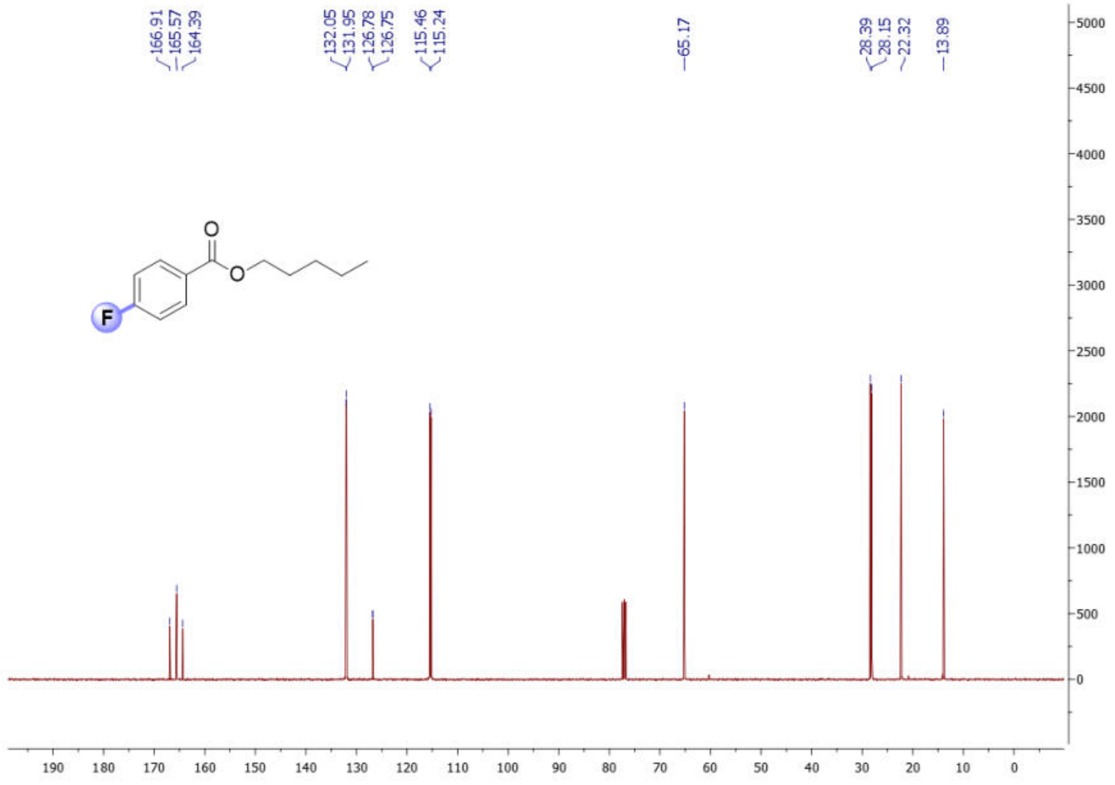

^{19}F NMR of compound **8j** in $CDCl_3$

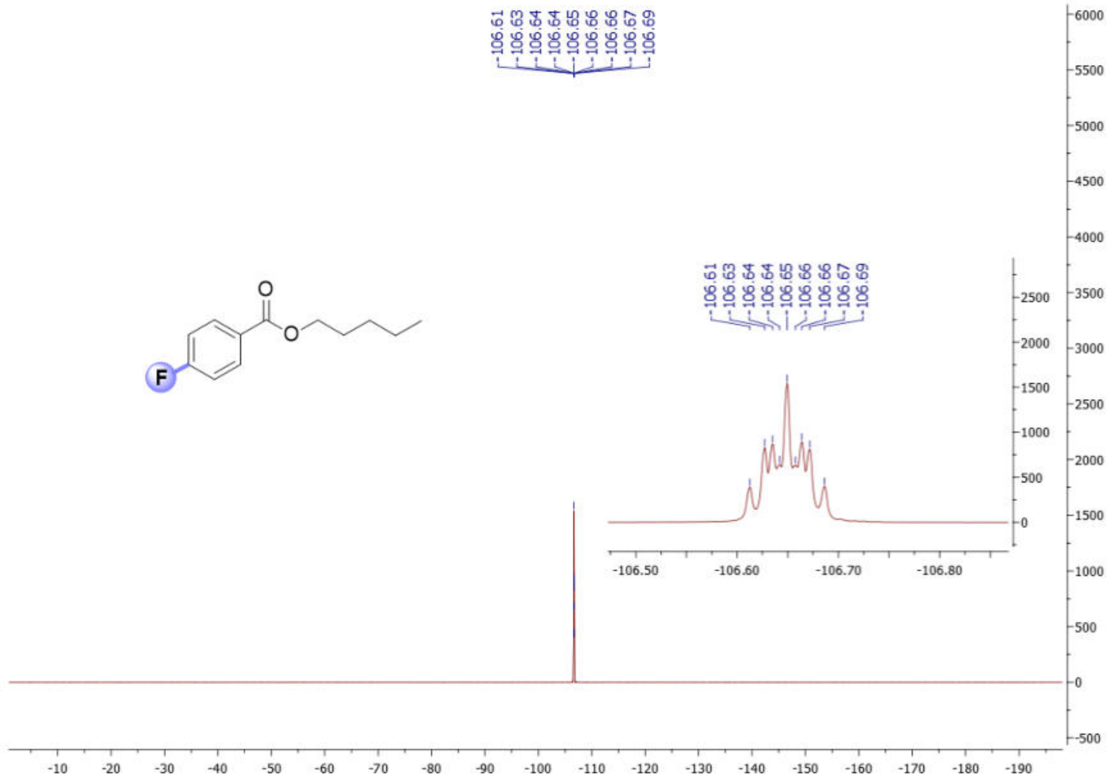

^{1}H NMR of compound **8k** in $CDCl_3$

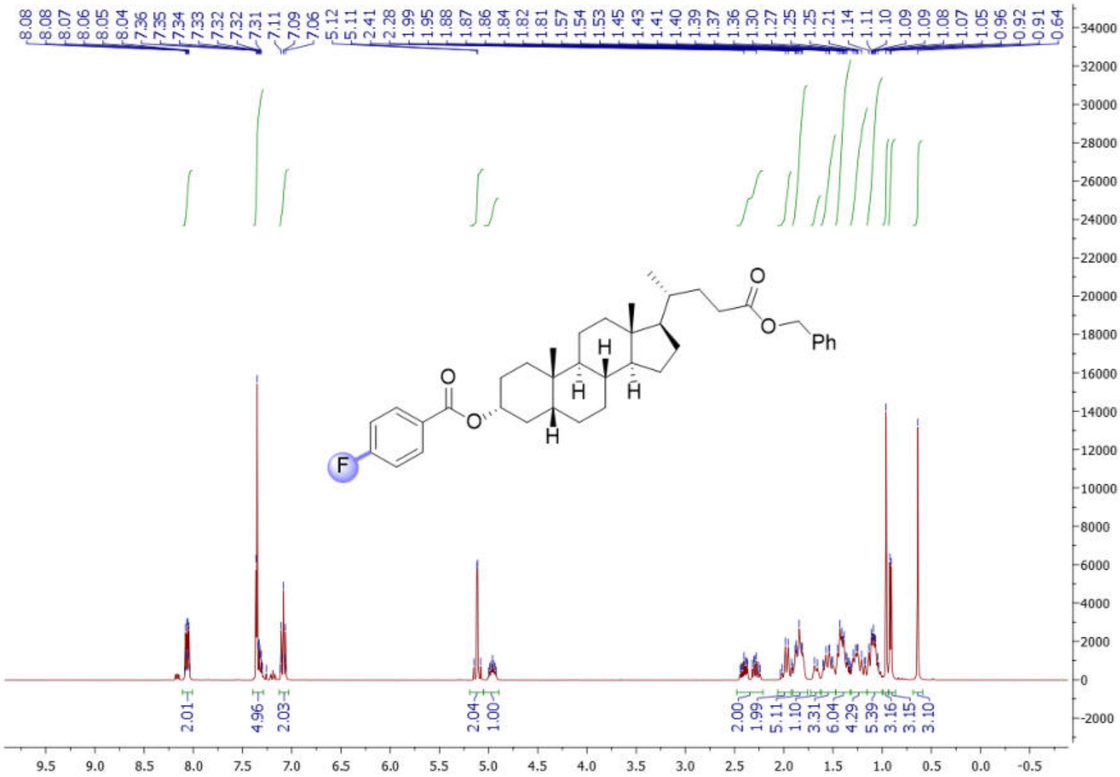

^{13}C NMR of compound **8k** in $CDCl_3$

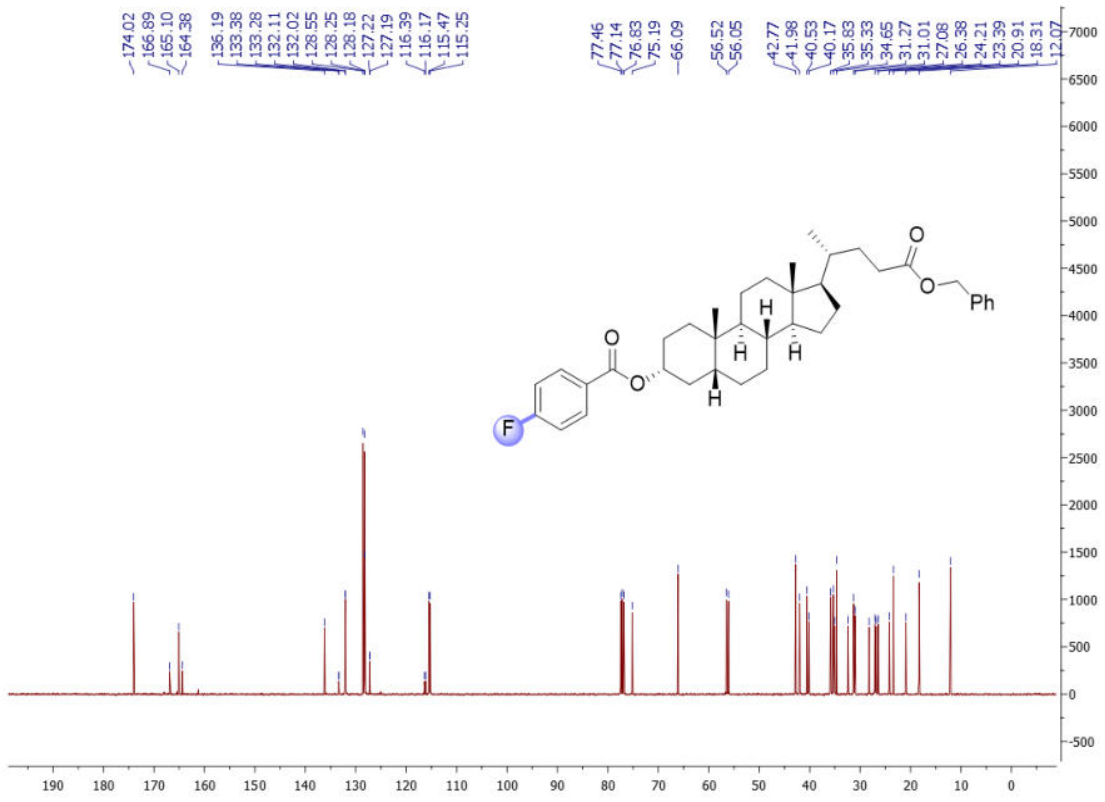

^{19}F NMR of compound **8k** in $CDCl_3$

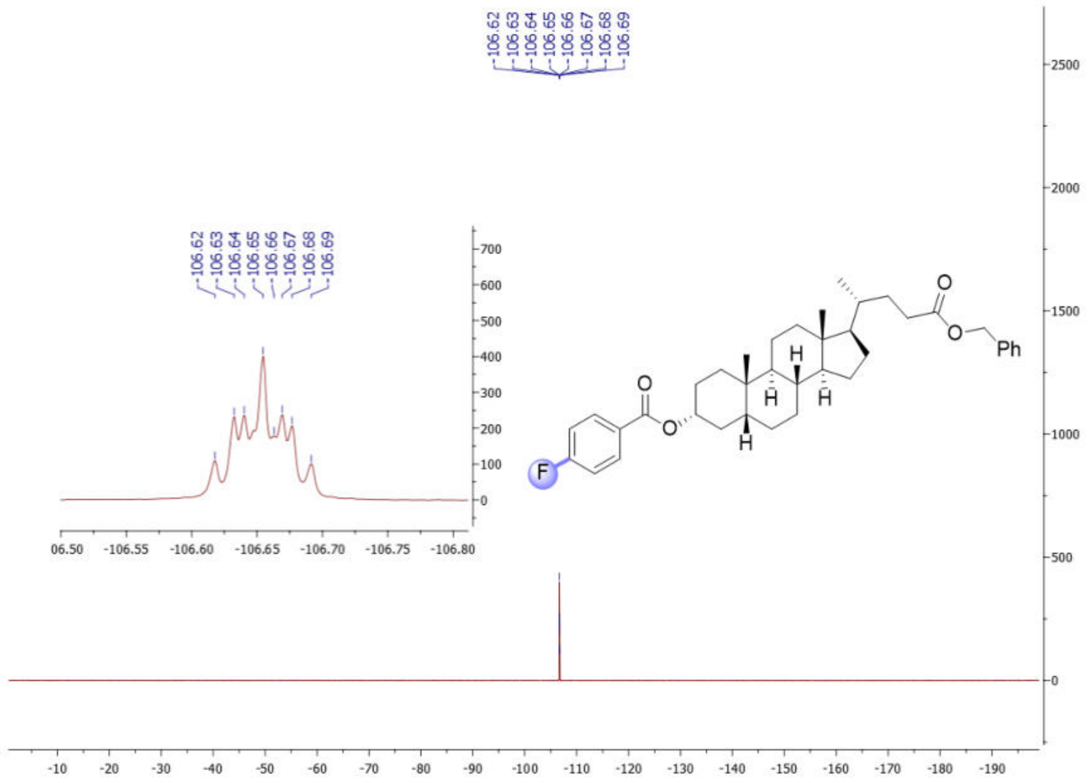

^{1}H NMR of compound **10d** in $CDCl_3$

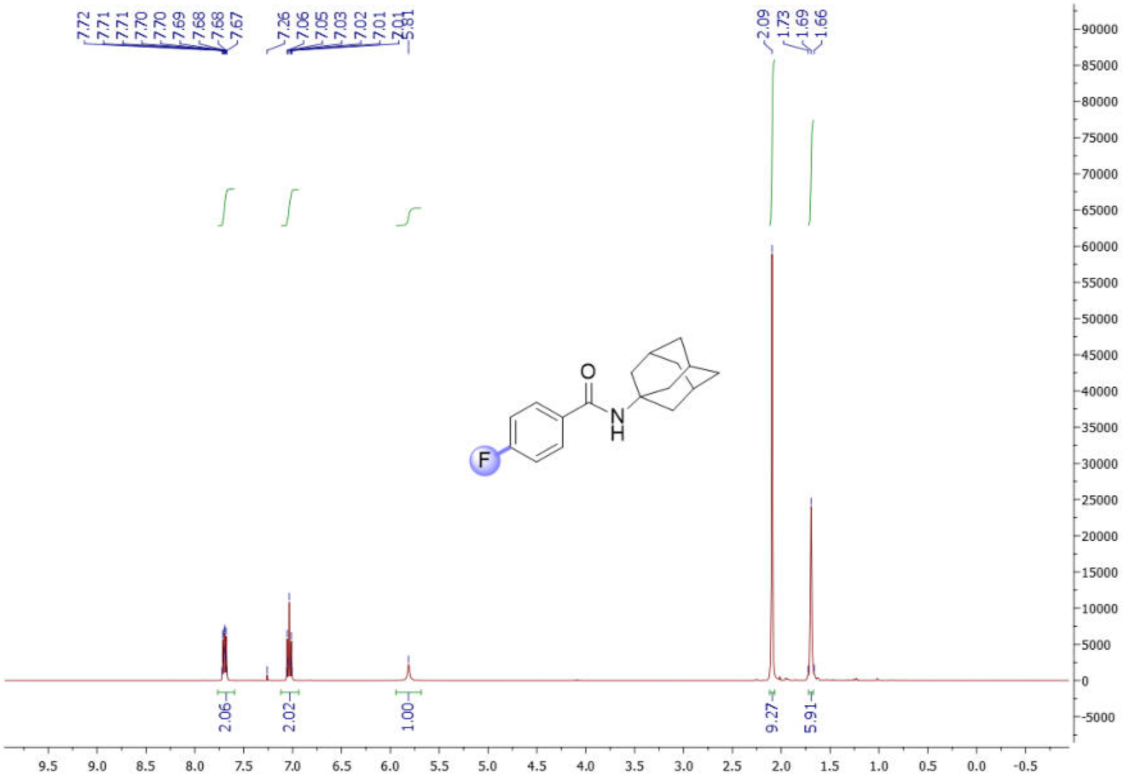

^{13}C NMR of compound **10d** in $CDCl_3$

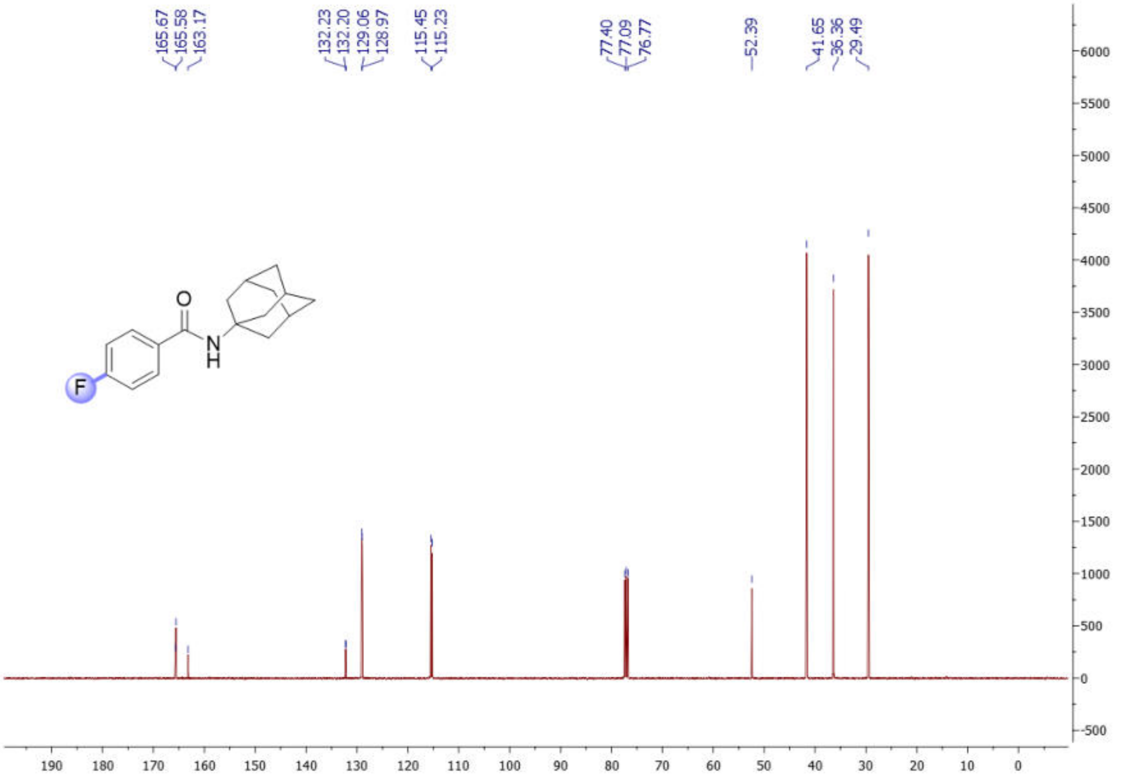

^{19}F NMR of compound **10d** in $CDCl_3$

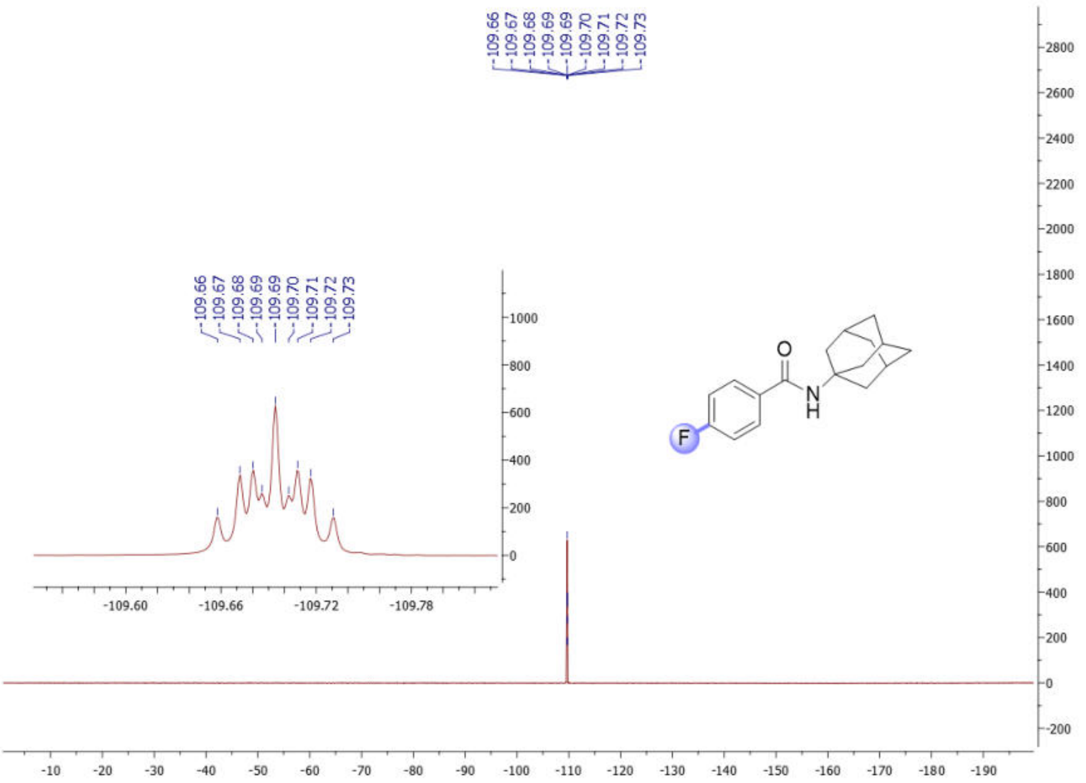

^{1}H NMR of compound **10a** in $CDCl_3$

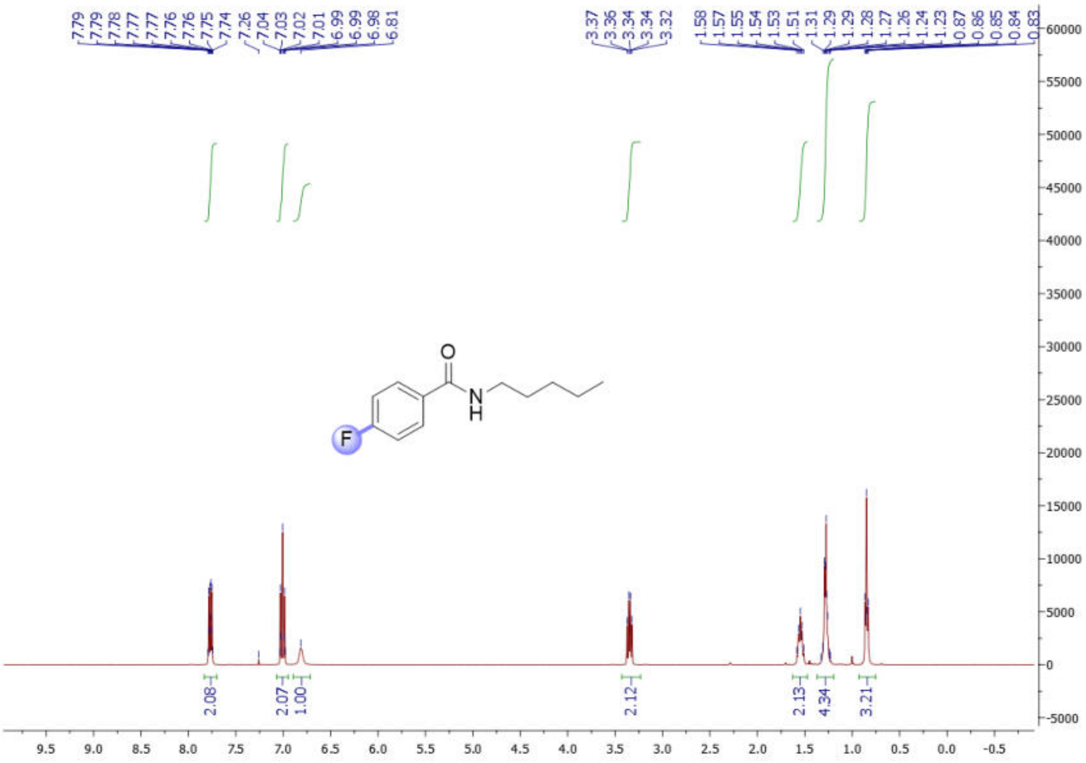

^{13}C NMR of compound **10a** in $CDCl_3$

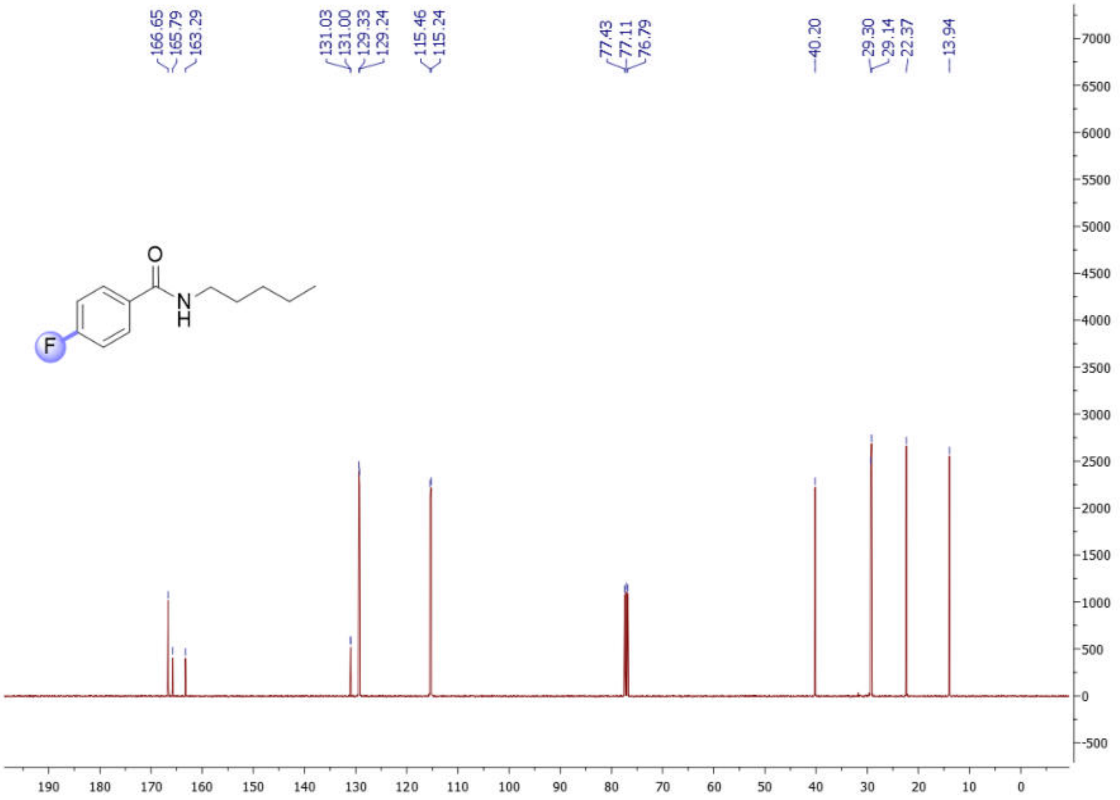

^{19}F NMR of compound **10a** in $CDCl_3$

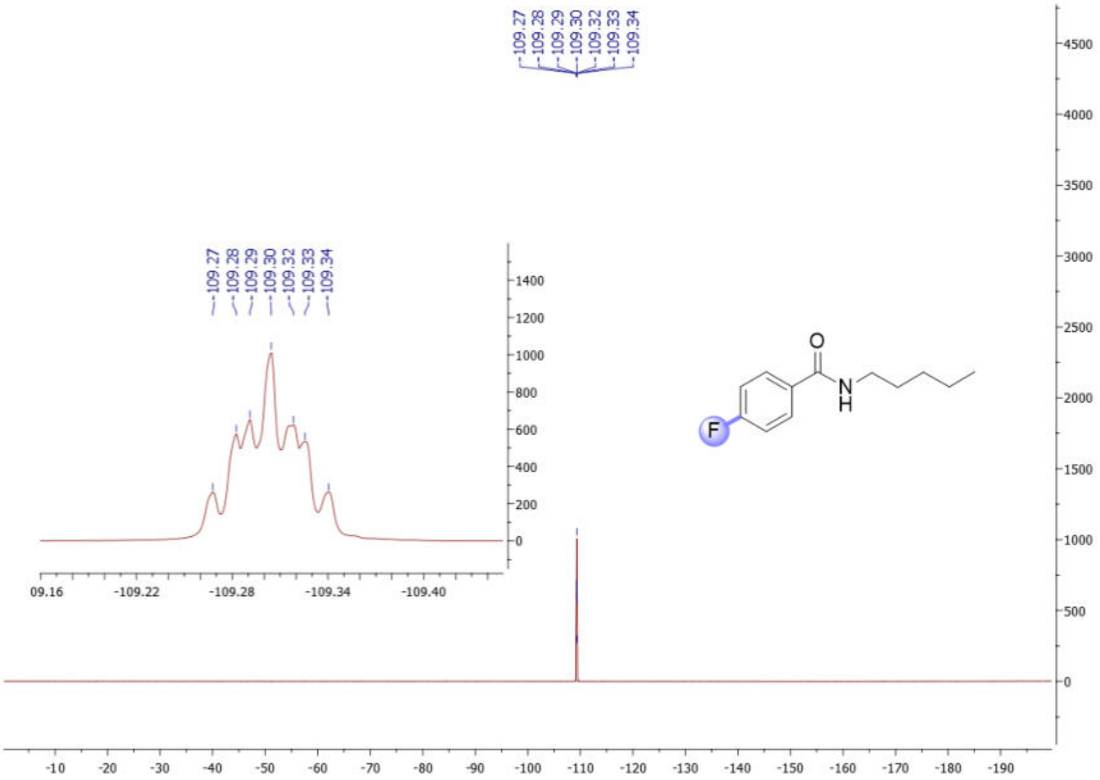

^{1}H NMR of compound **10b** in $CDCl_3$

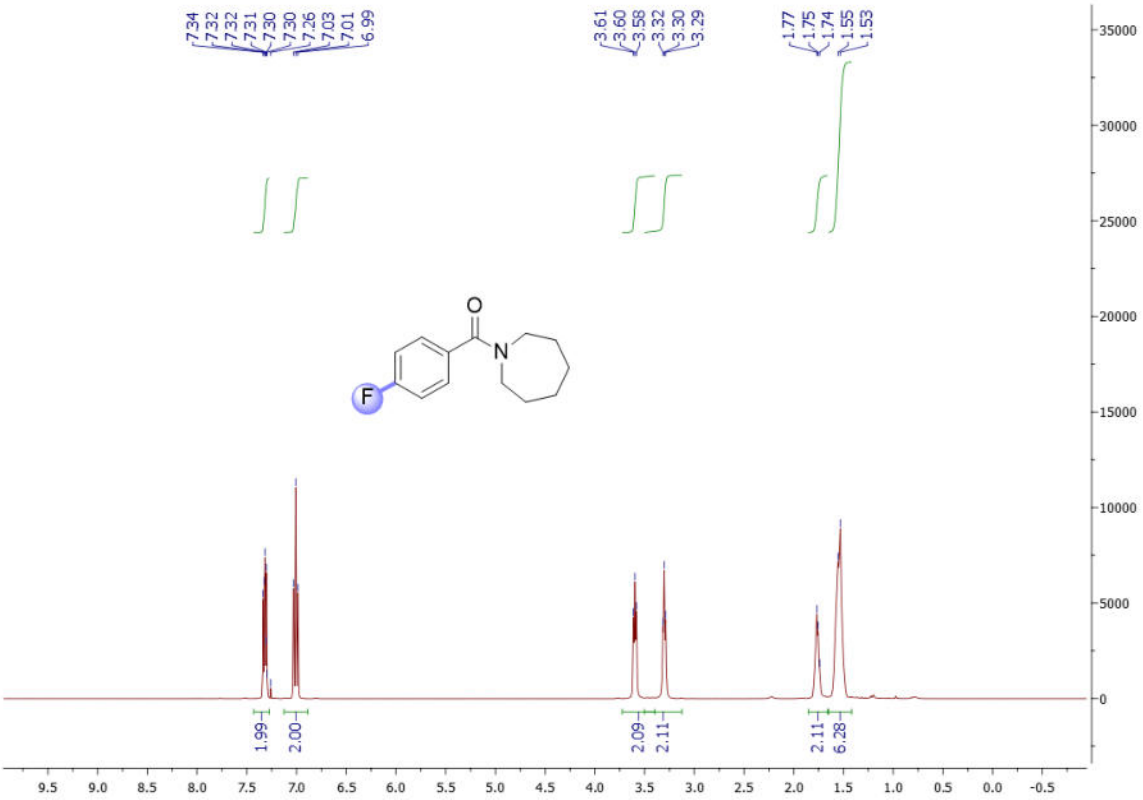

^{13}C NMR of compound **10b** in $CDCl_3$

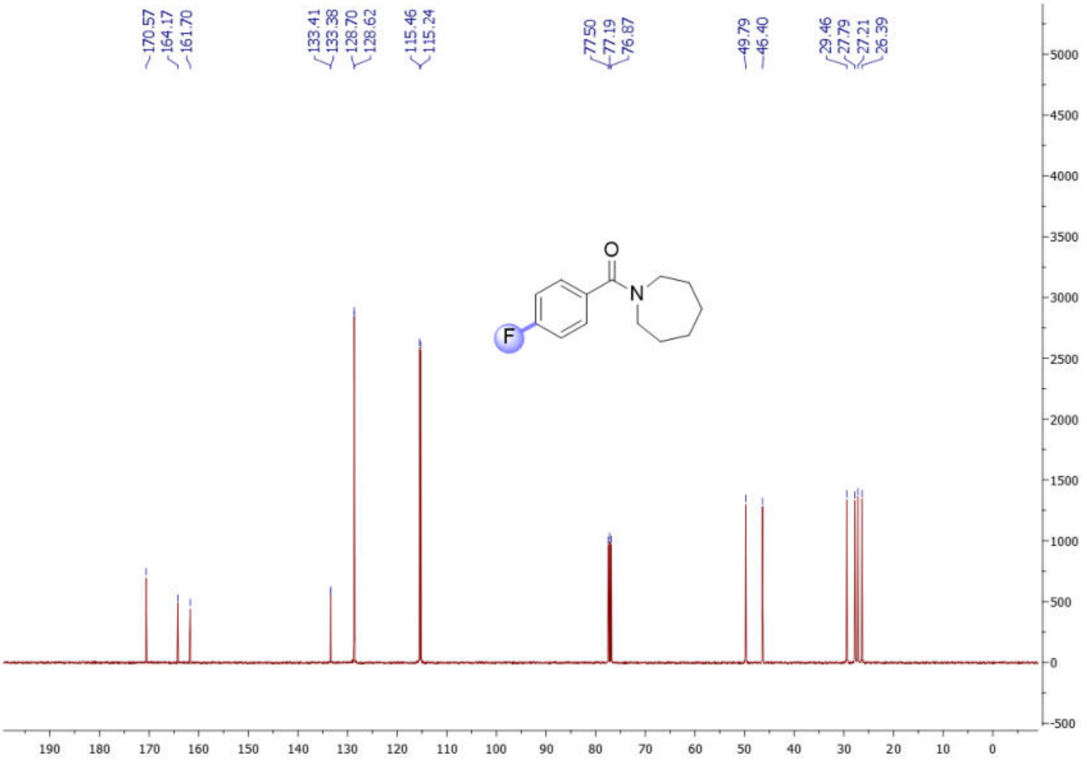

^{19}F NMR of compound **10b** in $CDCl_3$

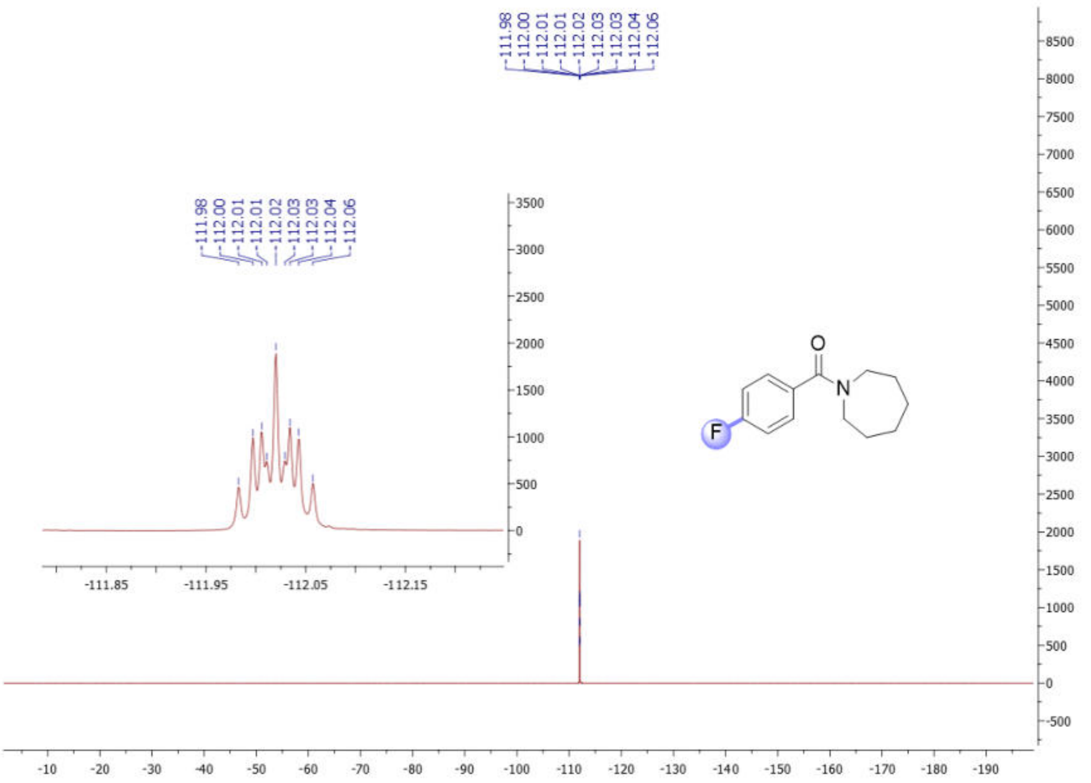

^{1}H NMR of compound **10c** in $CDCl_3$

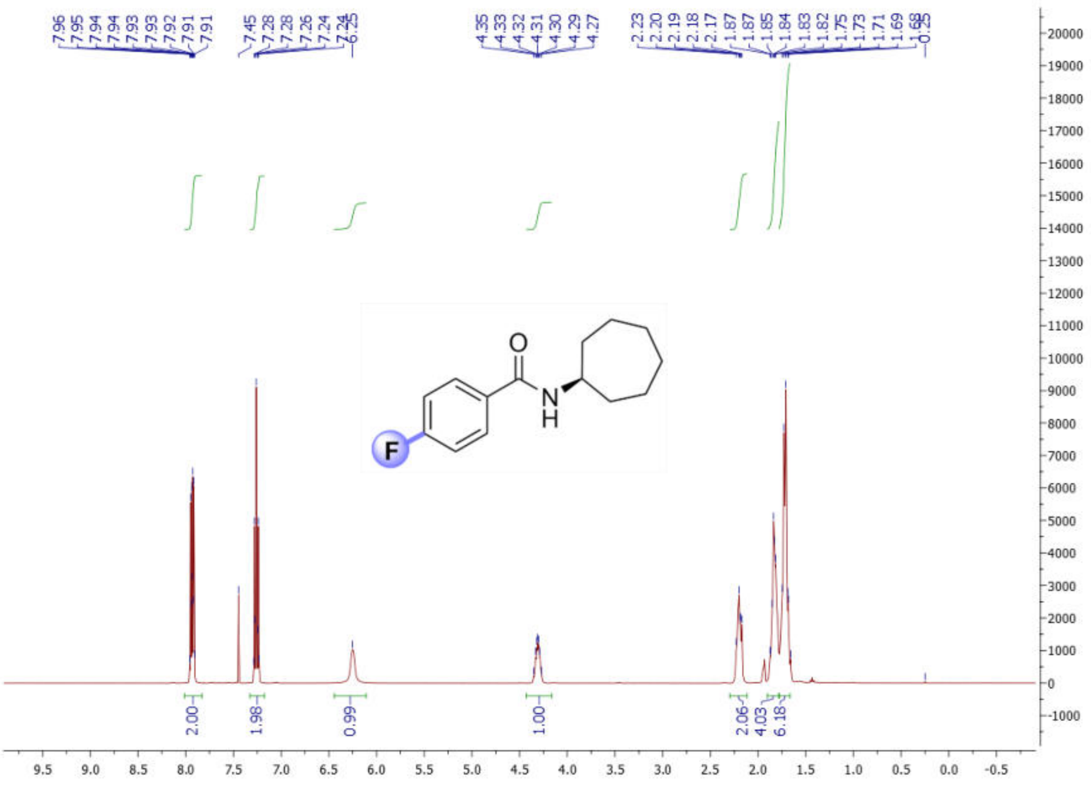

^{13}C NMR of compound **10c** in $CDCl_3$

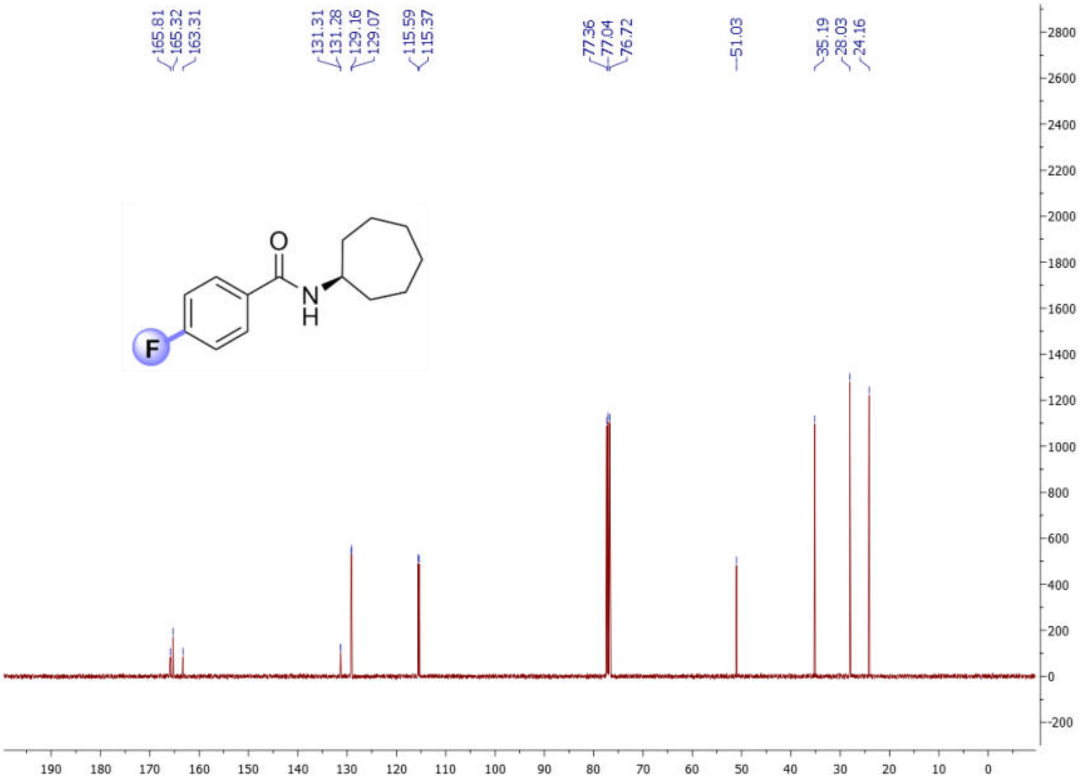

^{19}F NMR of compound **10c** in $CDCl_3$

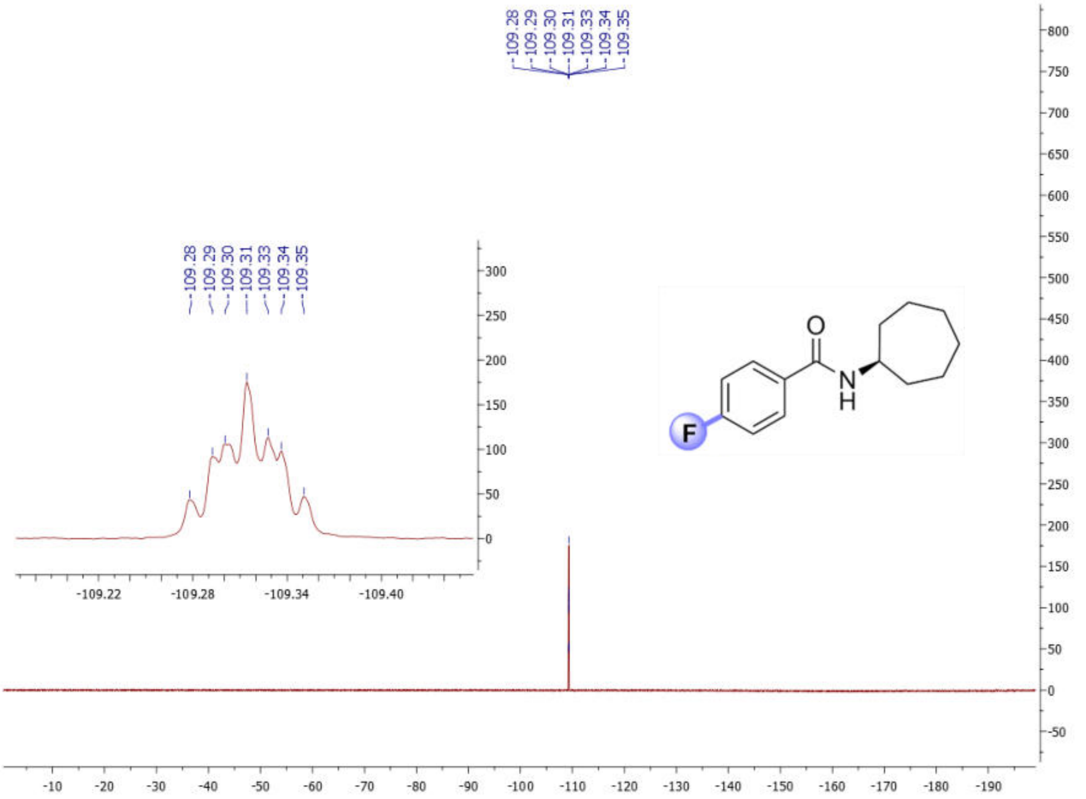

^{1}H NMR of compound **10e** in $CDCl_3$

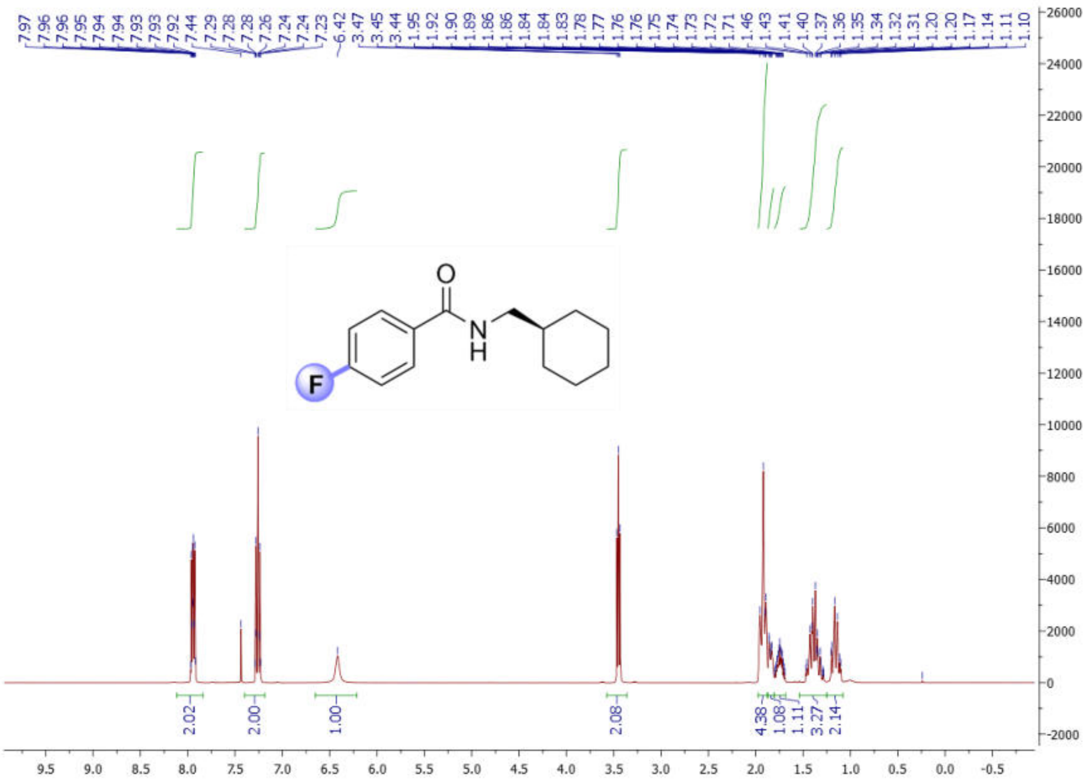

^{13}C NMR of compound **10e** in $CDCl_3$

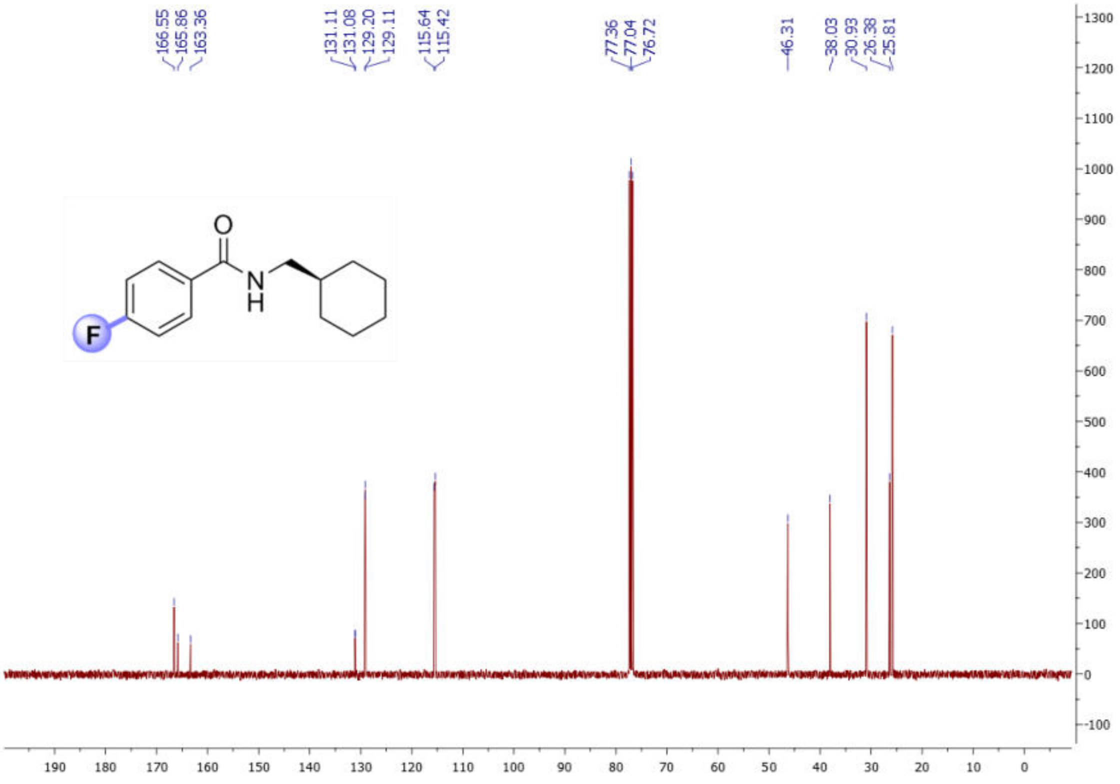

^{19}F NMR of compound **10e** in $CDCl_3$

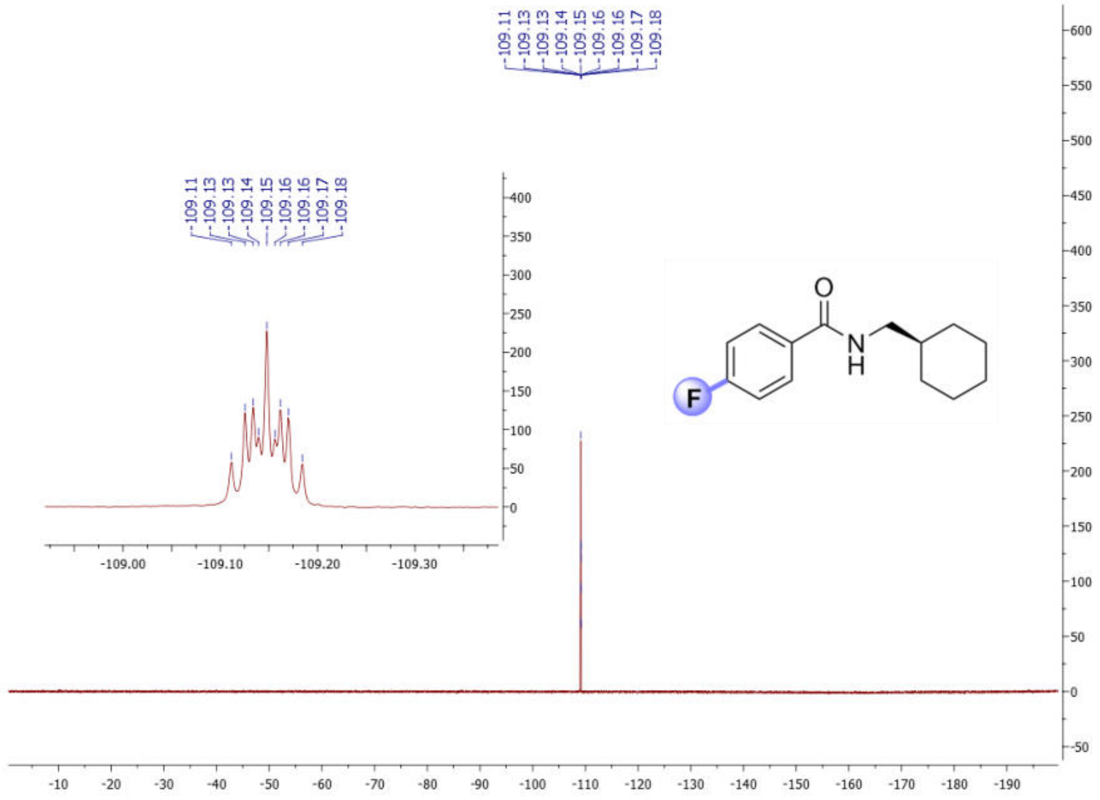

^{1}H NMR of compound **10f** in $CDCl_3$

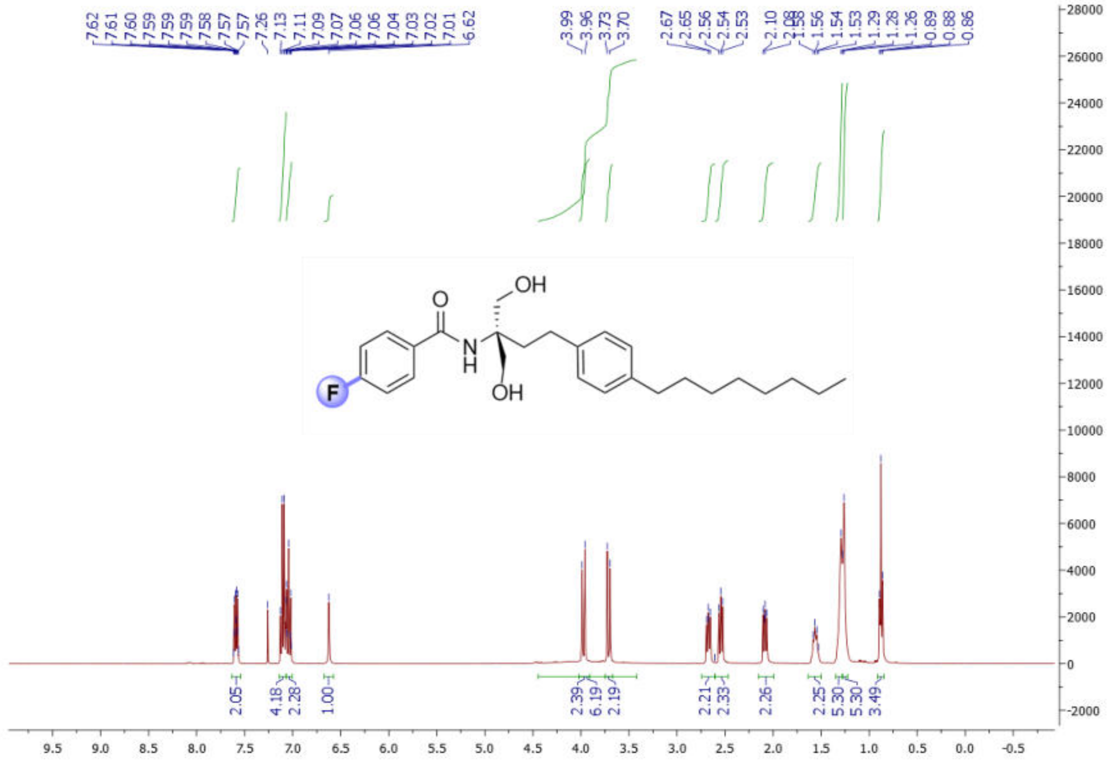

^{13}C NMR of compound **10f** in $CDCl_3$

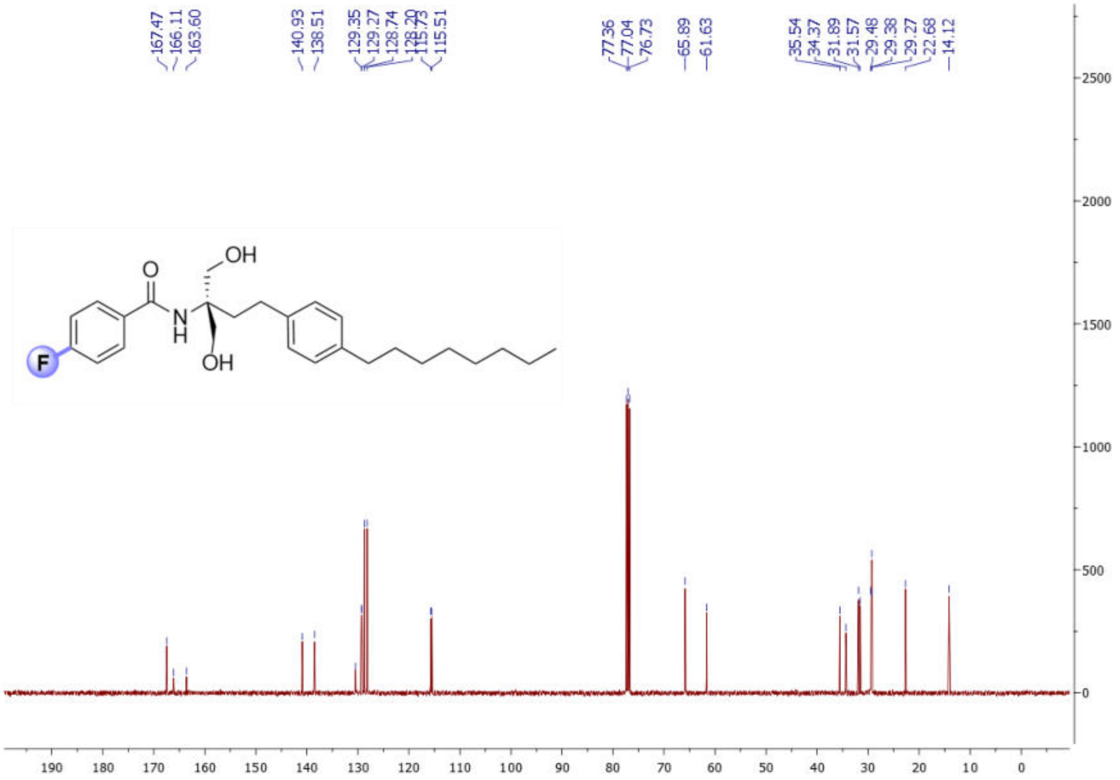

^{19}F NMR of compound **10f** in $CDCl_3$

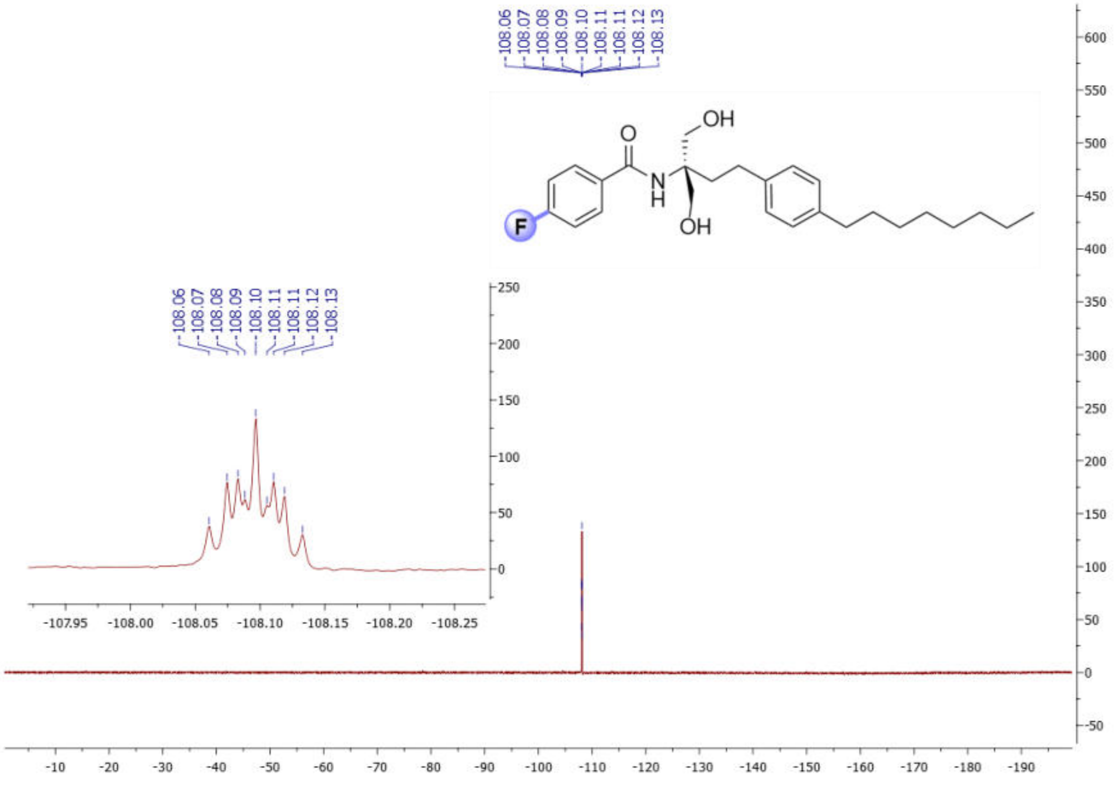

^{1}H NMR of compound **10g** in $CDCl_3$

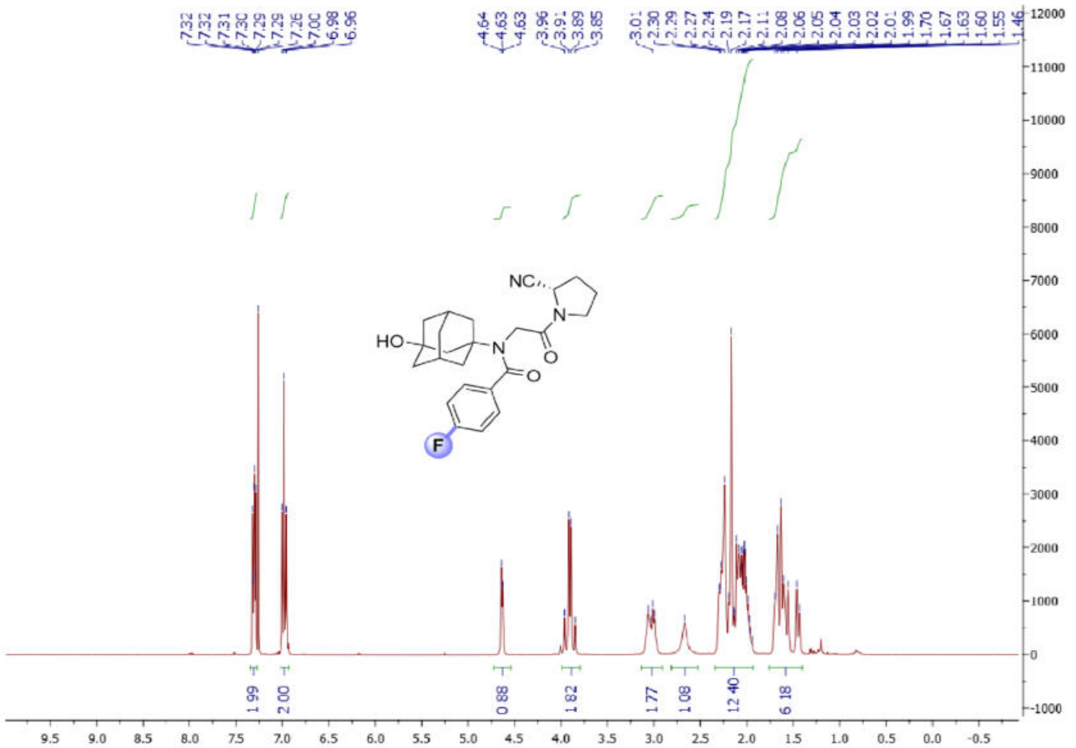

^{13}C NMR of compound **10g** in $CDCl_3$

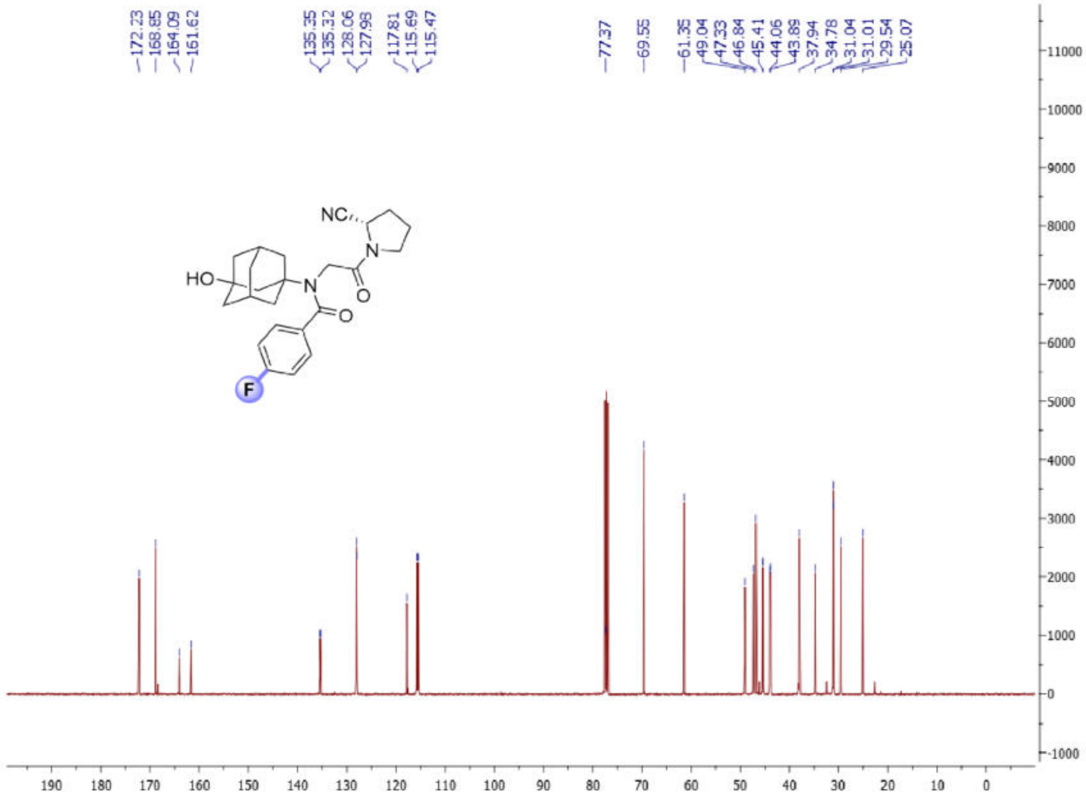

^{19}F NMR of compound **10g** in $CDCl_3$

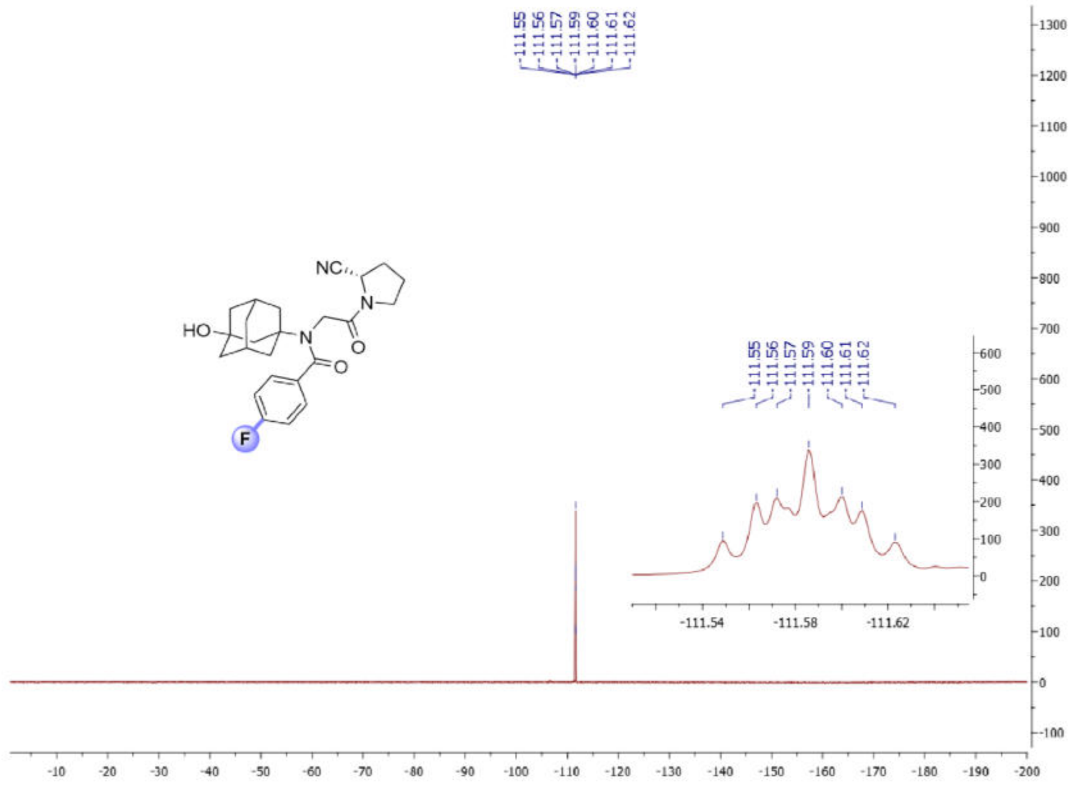

^{1}H NMR of compound **10h** in $CDCl_3$

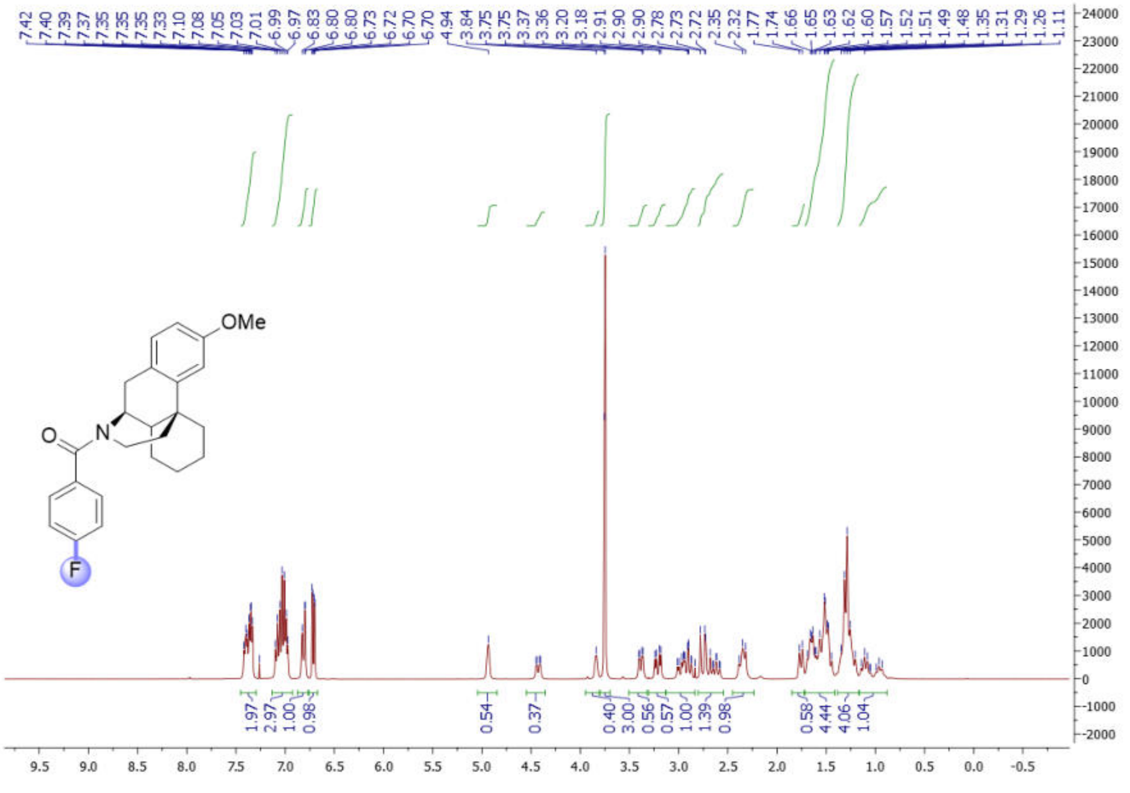

^{13}C NMR of compound **10h** in $CDCl_3$

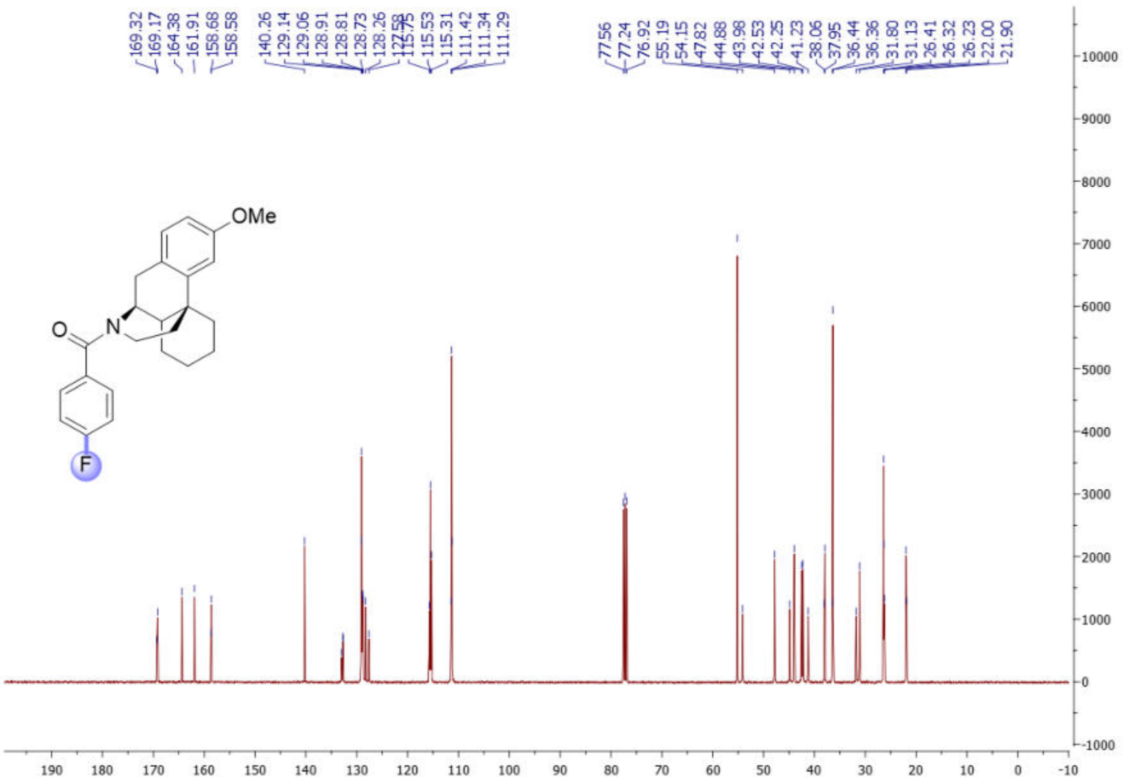

^{19}F NMR of compound **10h** in $CDCl_3$

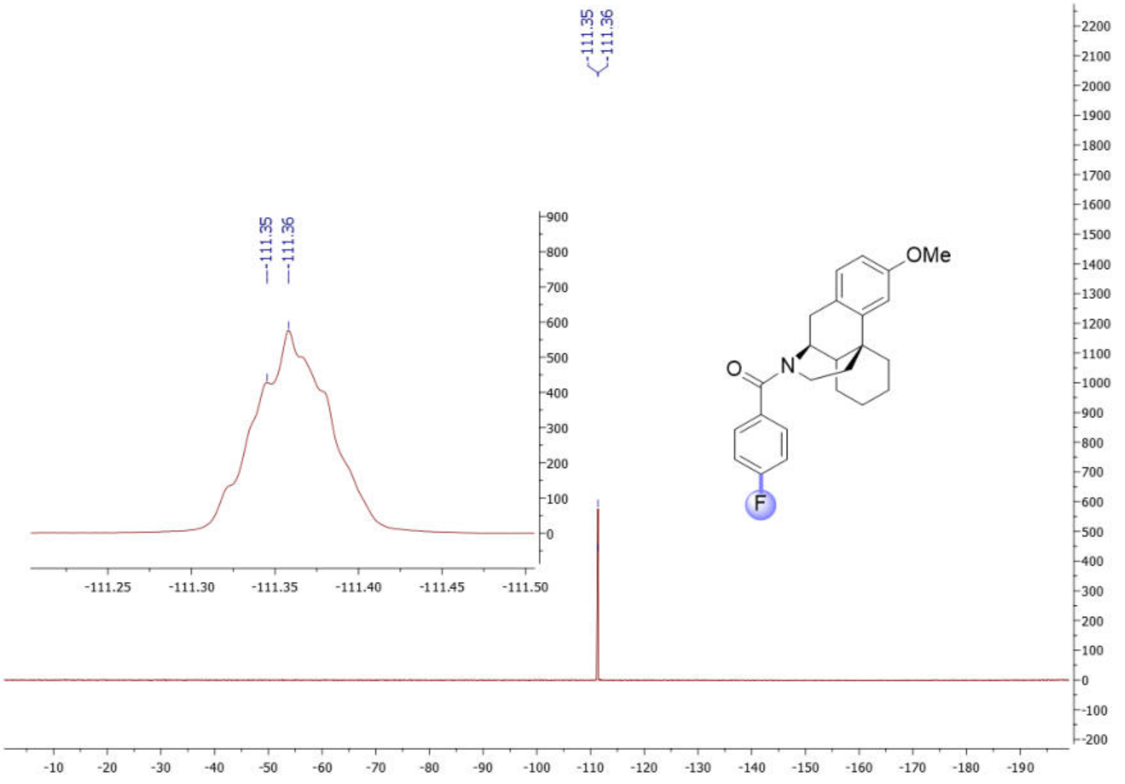

^{1}H NMR of compound **8l** in $CDCl_3$

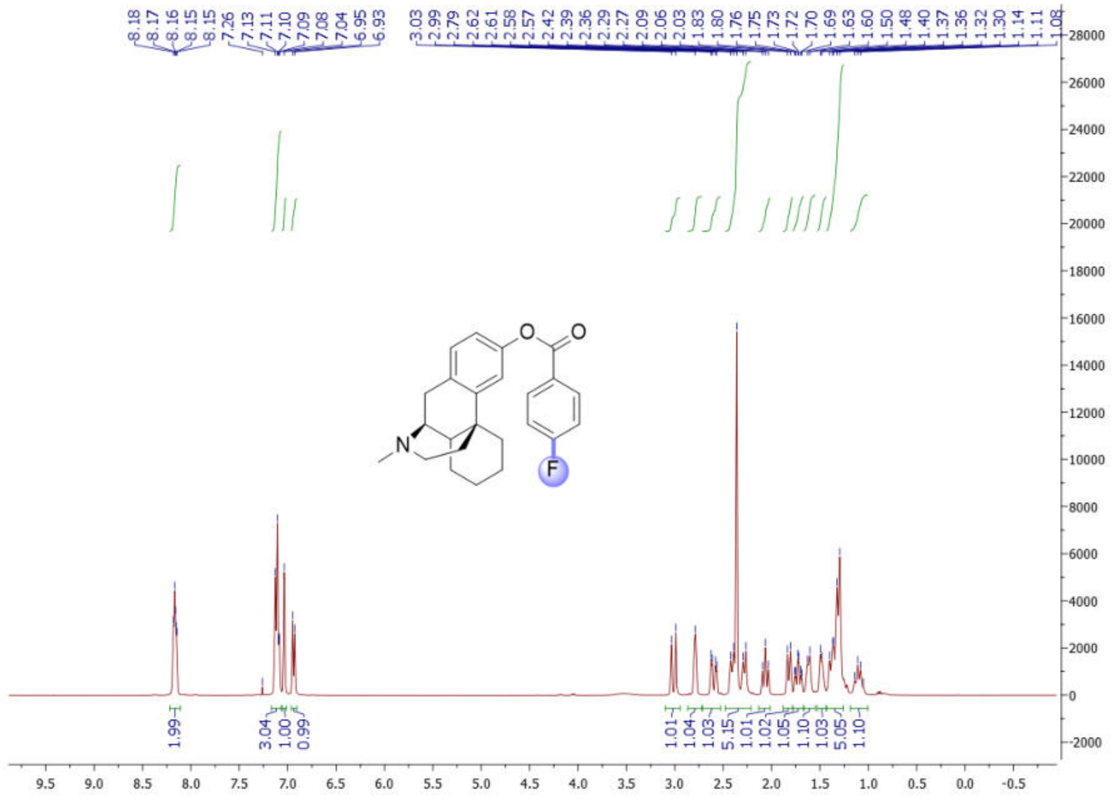

^{13}C NMR of compound **8l** in $CDCl_3$

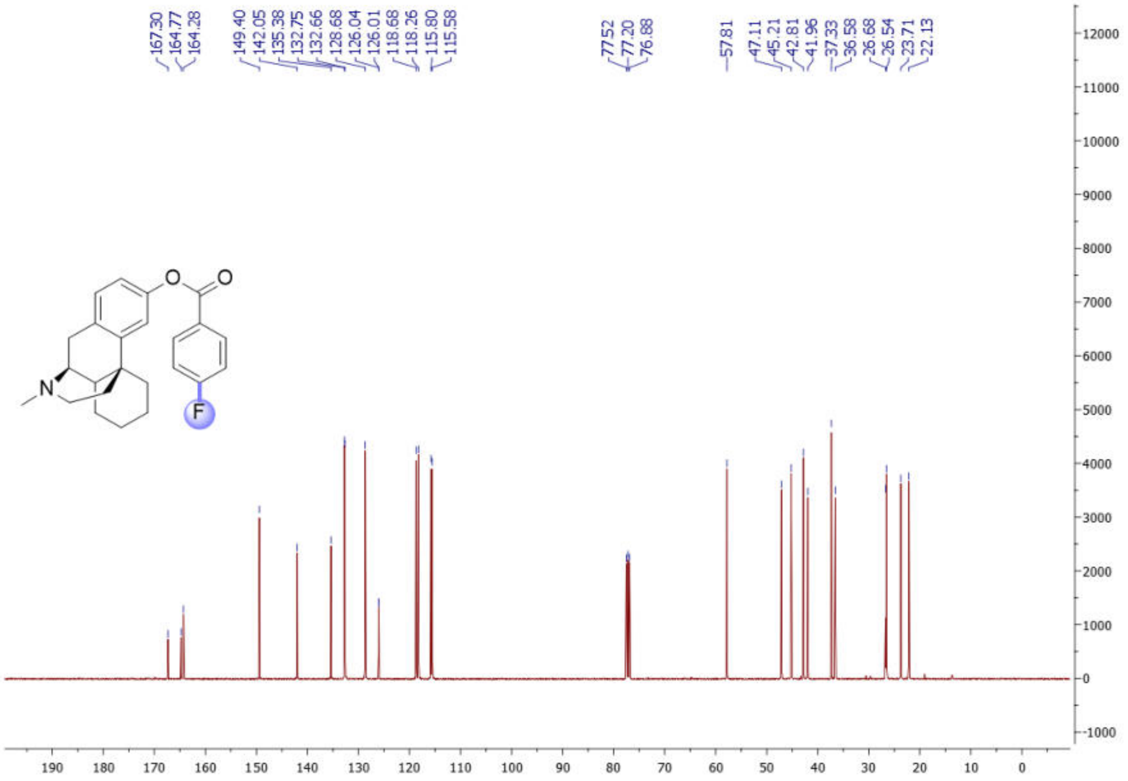

^{19}F NMR of compound **8l** in CDCl3

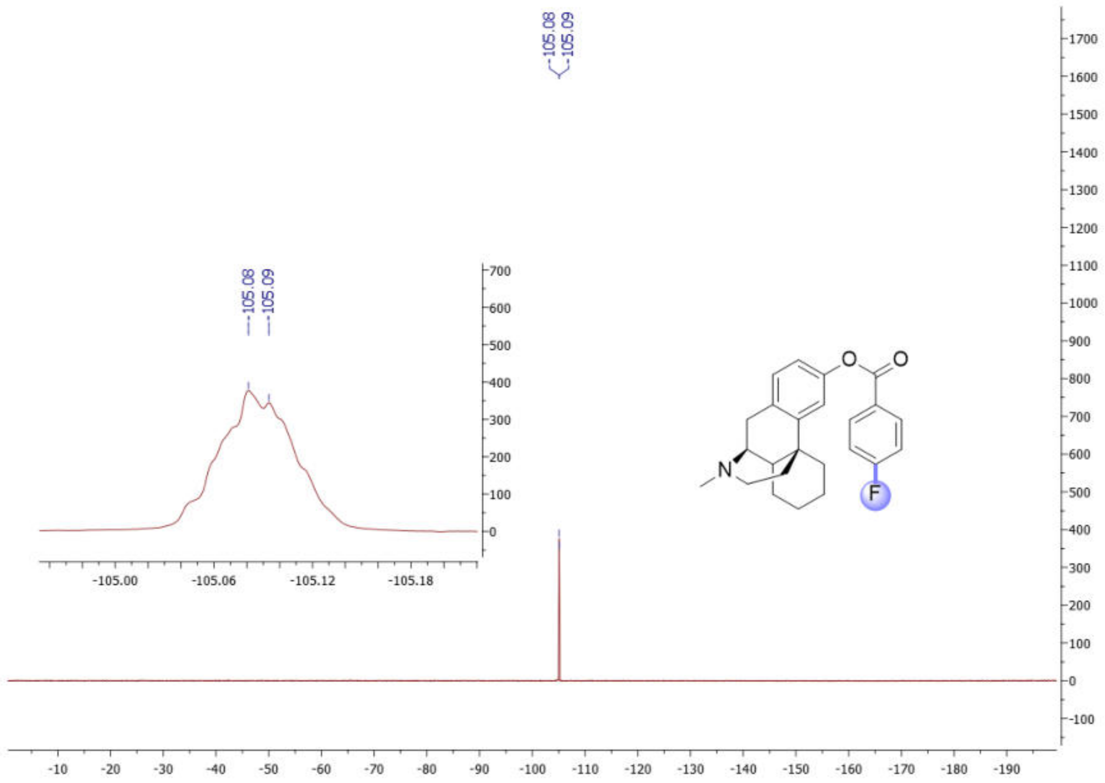

^{1}H NMR of compound **2d** in CDCl3

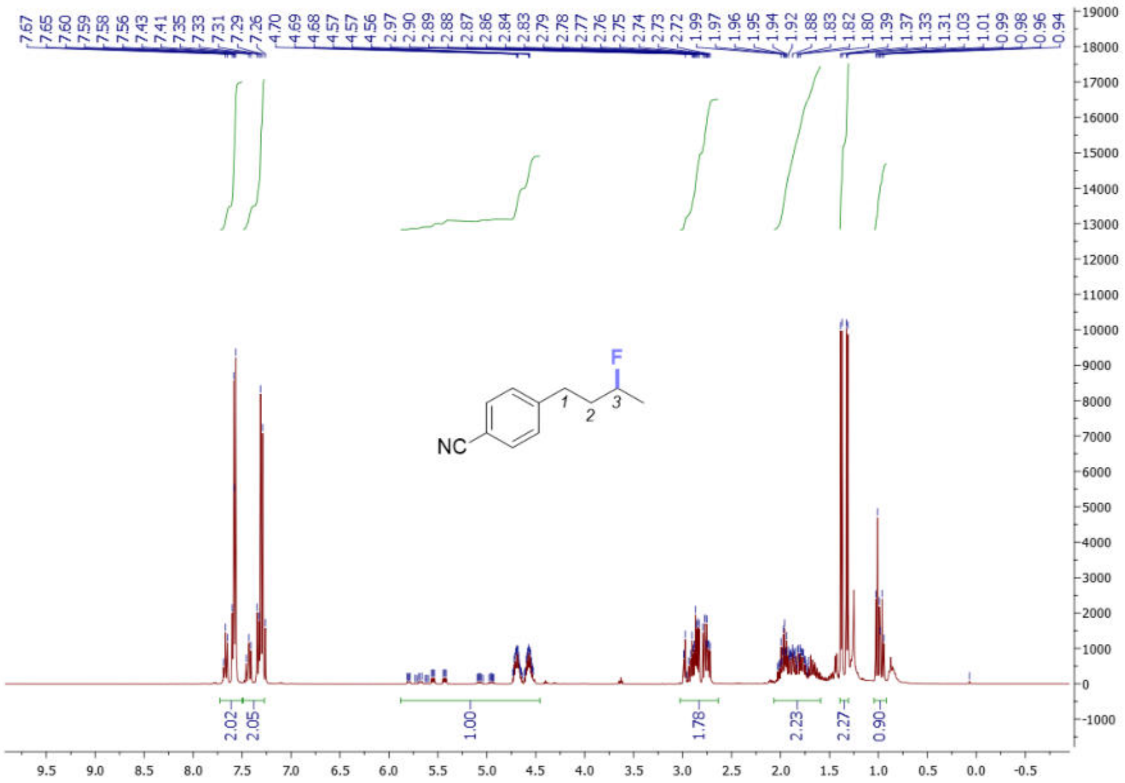

^{13}C NMR of compound **2d** in $CDCl_3$

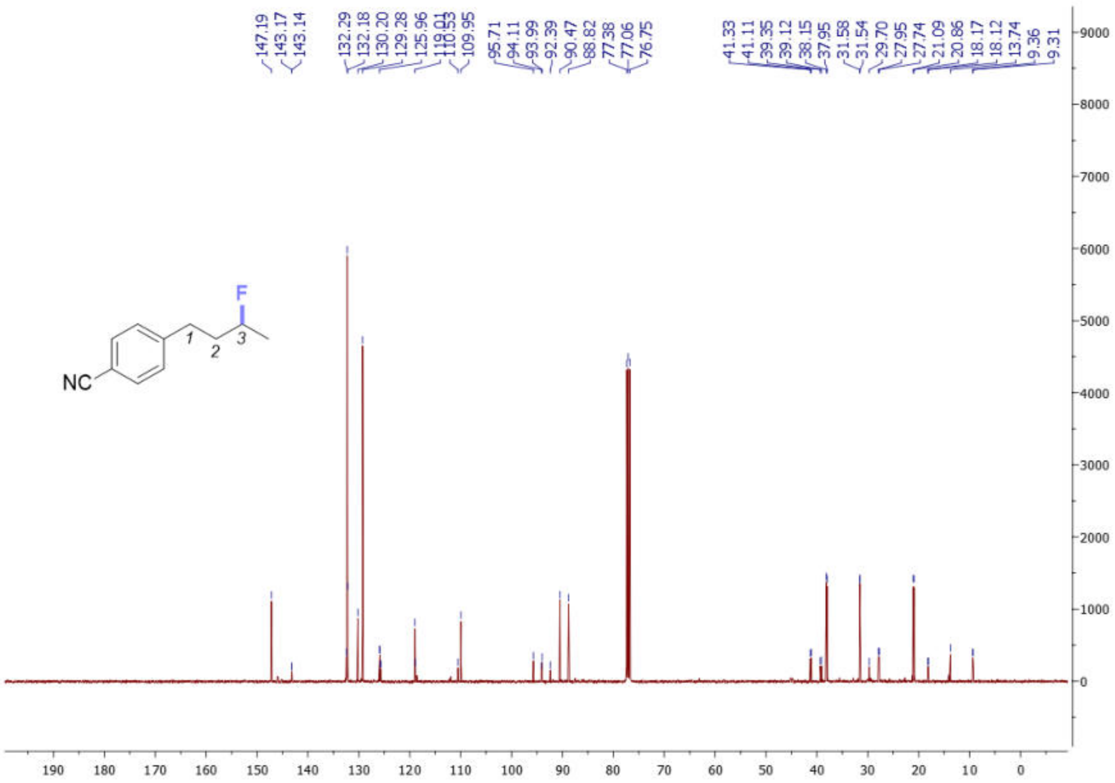

^{19}F NMR of compound **2d** in $CDCl_3$

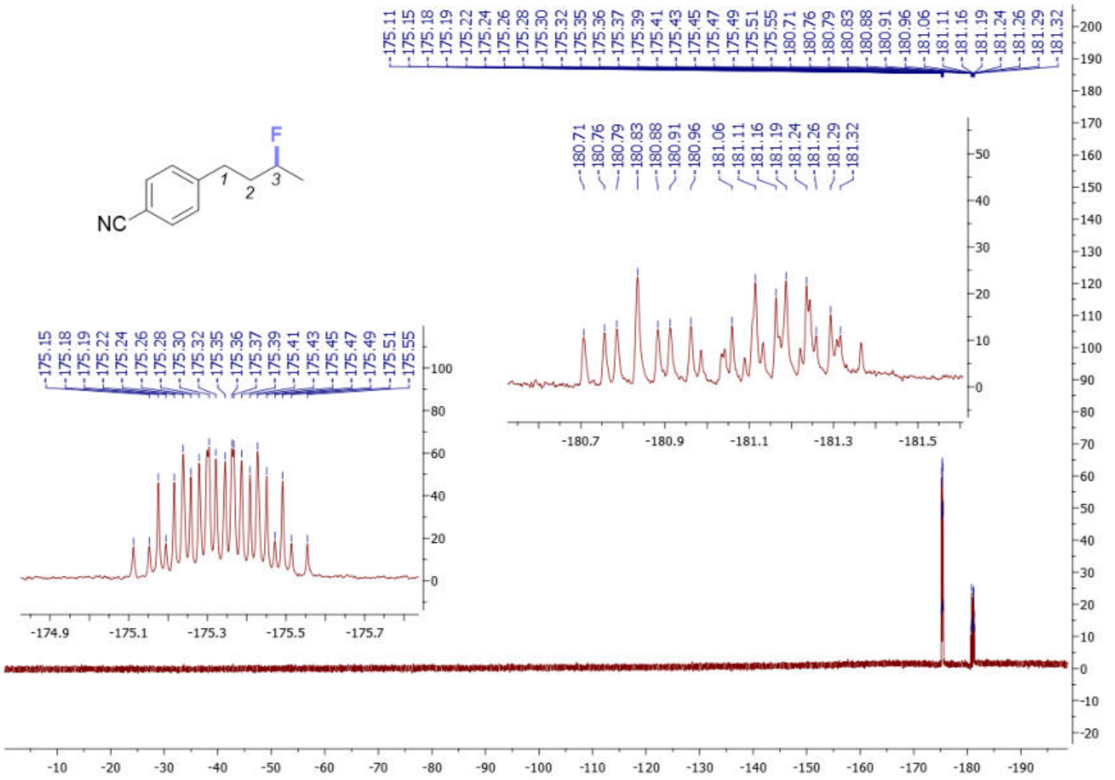

$^{19}F\{^1H\}$ NMR of compound **2d** in $CDCl_3$

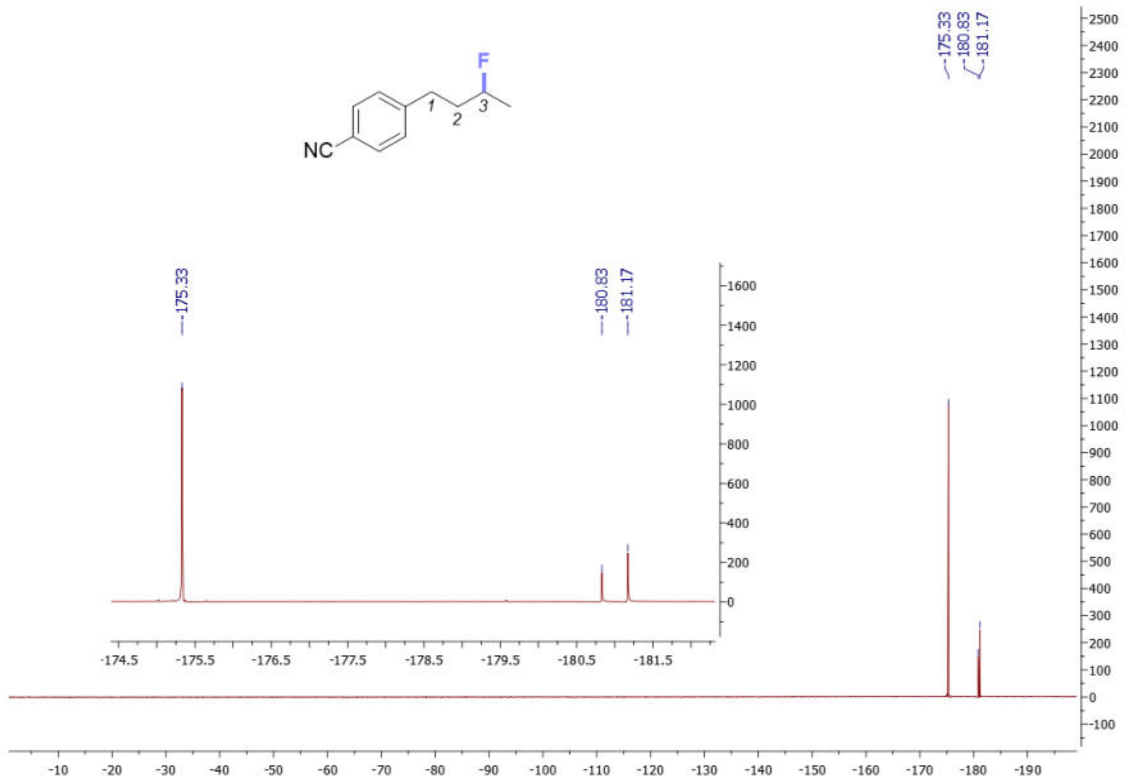

^1H NMR of compound **2h** in $CDCl_3$

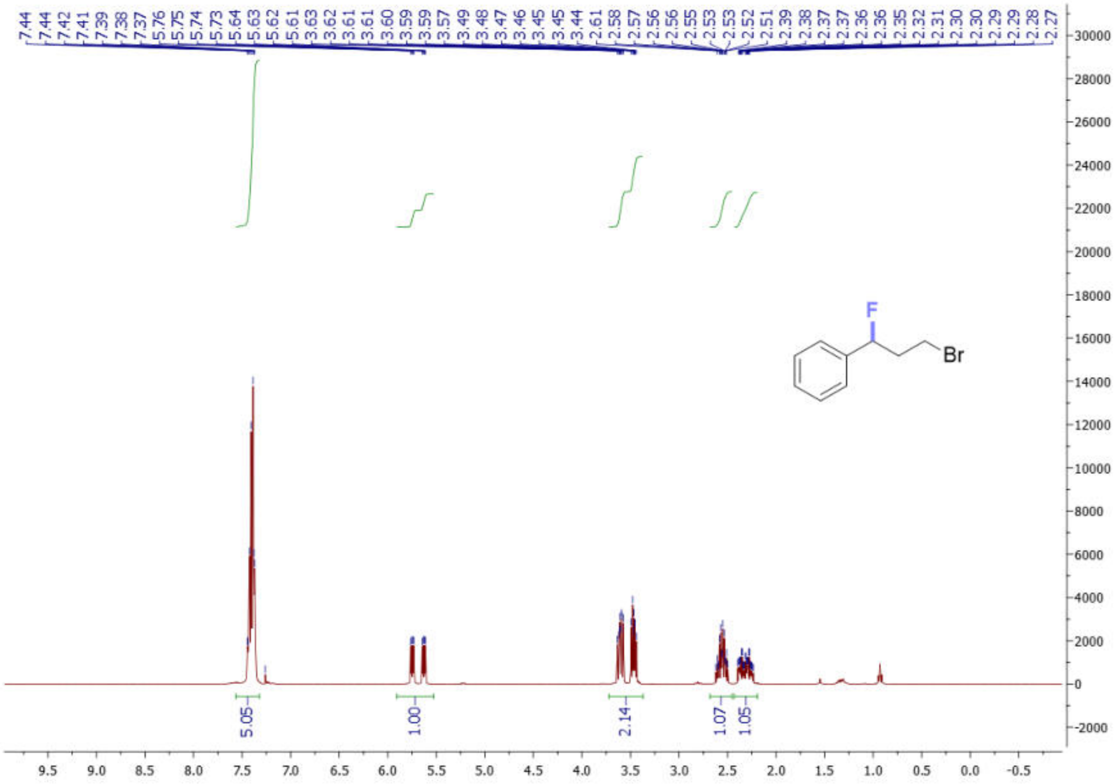

^{13}C NMR of compound **2h** in $CDCl_3$

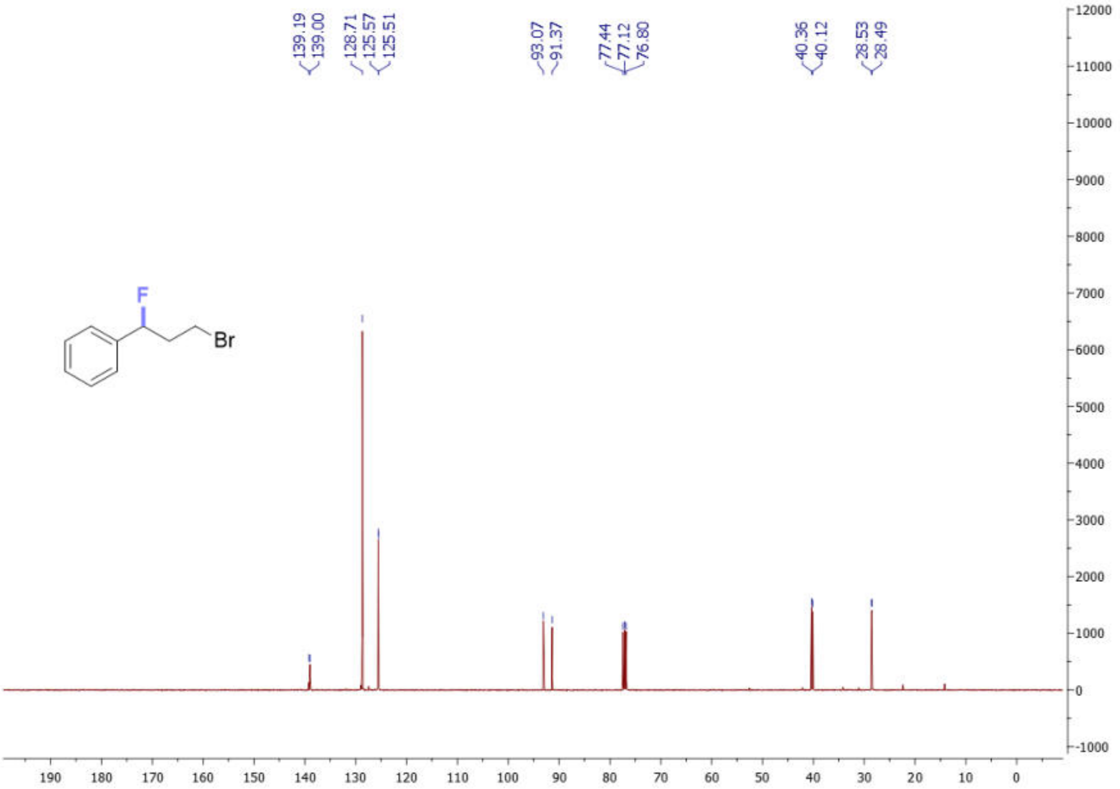

^{19}F NMR of compound **2h** in $CDCl_3$

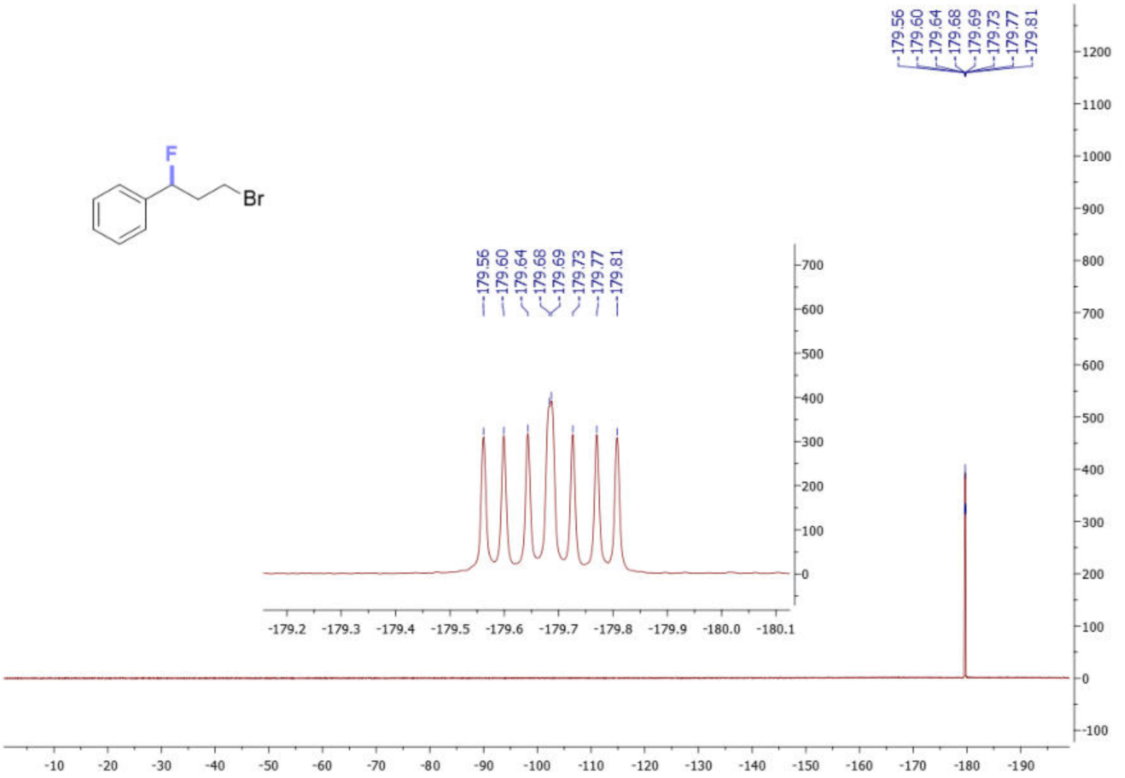

^{1}H NMR of compound **2j** in $CDCl_3$

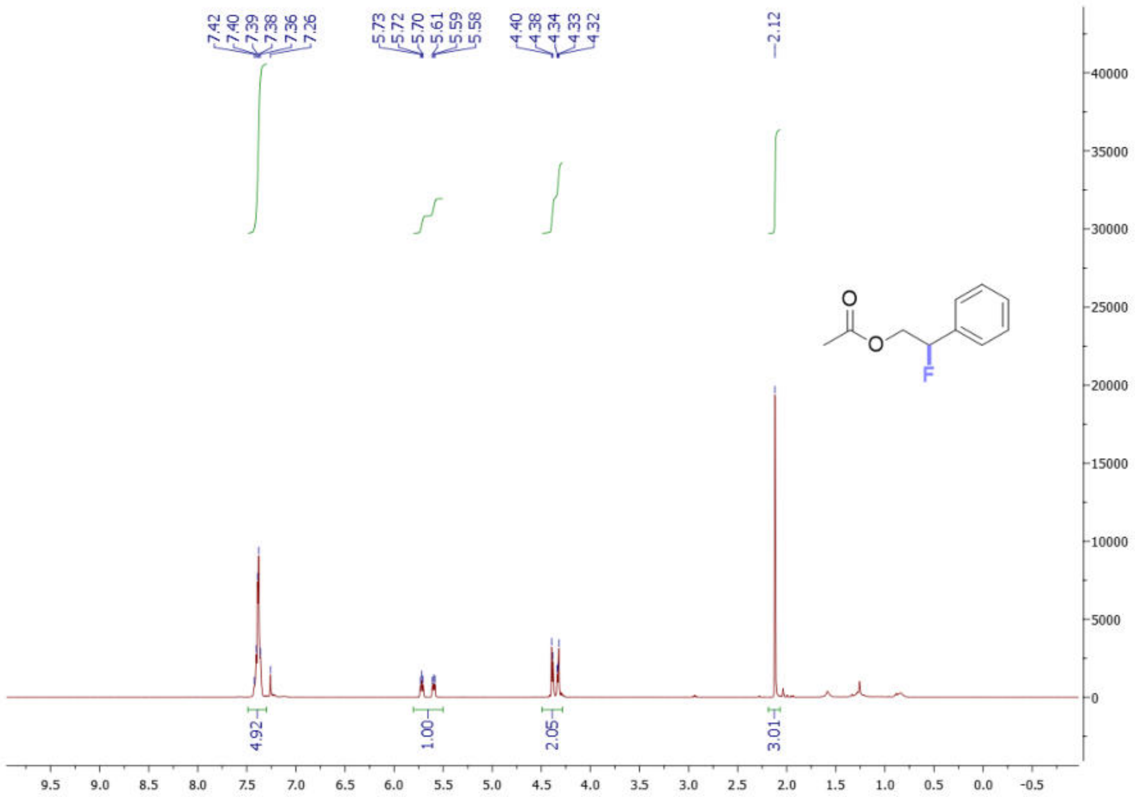

^{13}C NMR of compound **2j** in $CDCl_3$

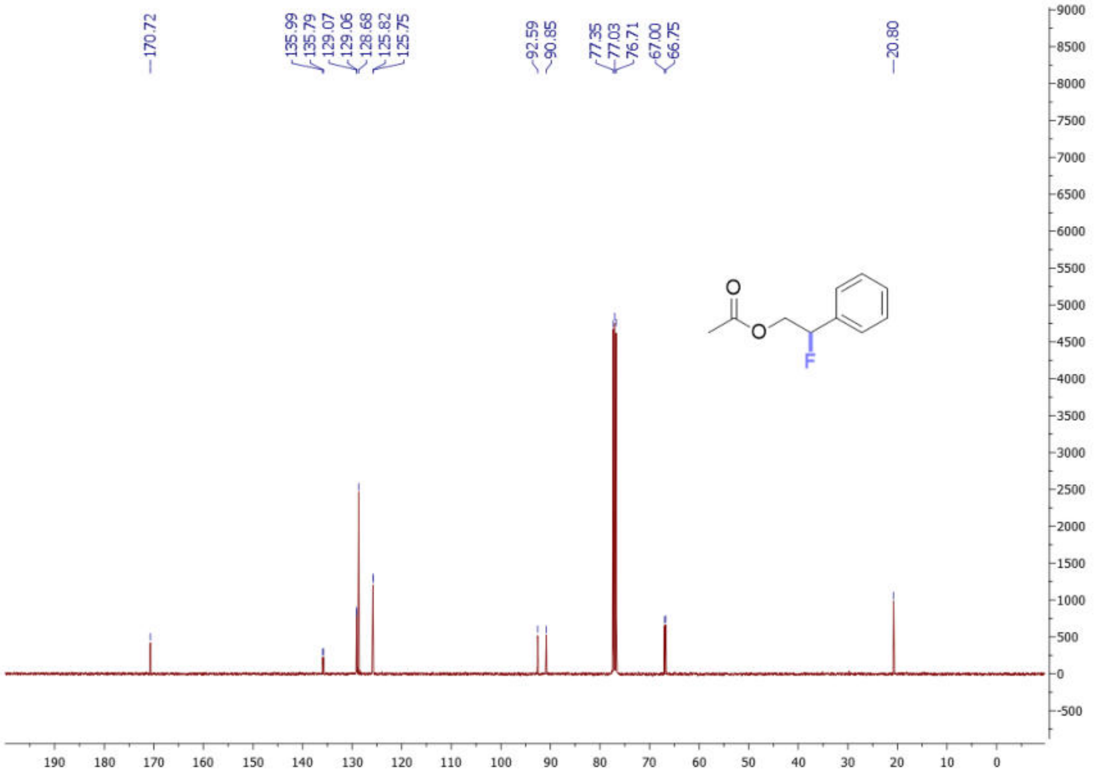

^{19}F NMR of compound **2j** in $CDCl_3$

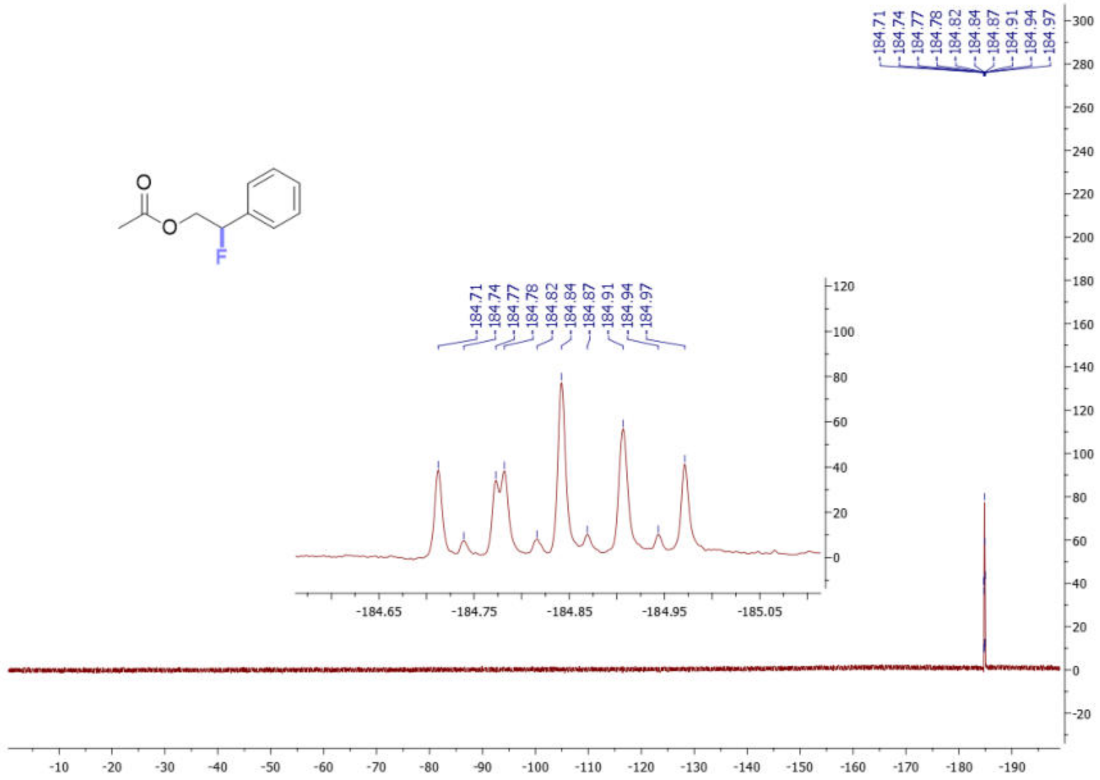

^{1}H NMR of compound **2k** in $CDCl_3$

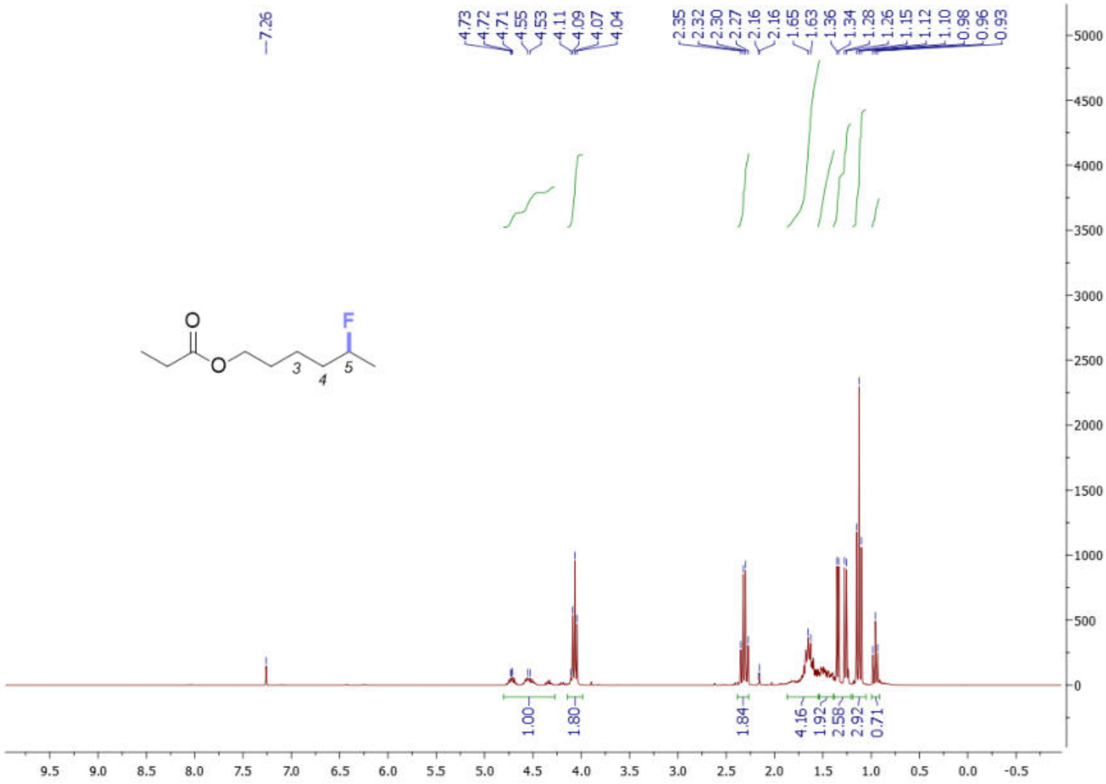

^{13}C NMR of compound **2k** in $CDCl_3$

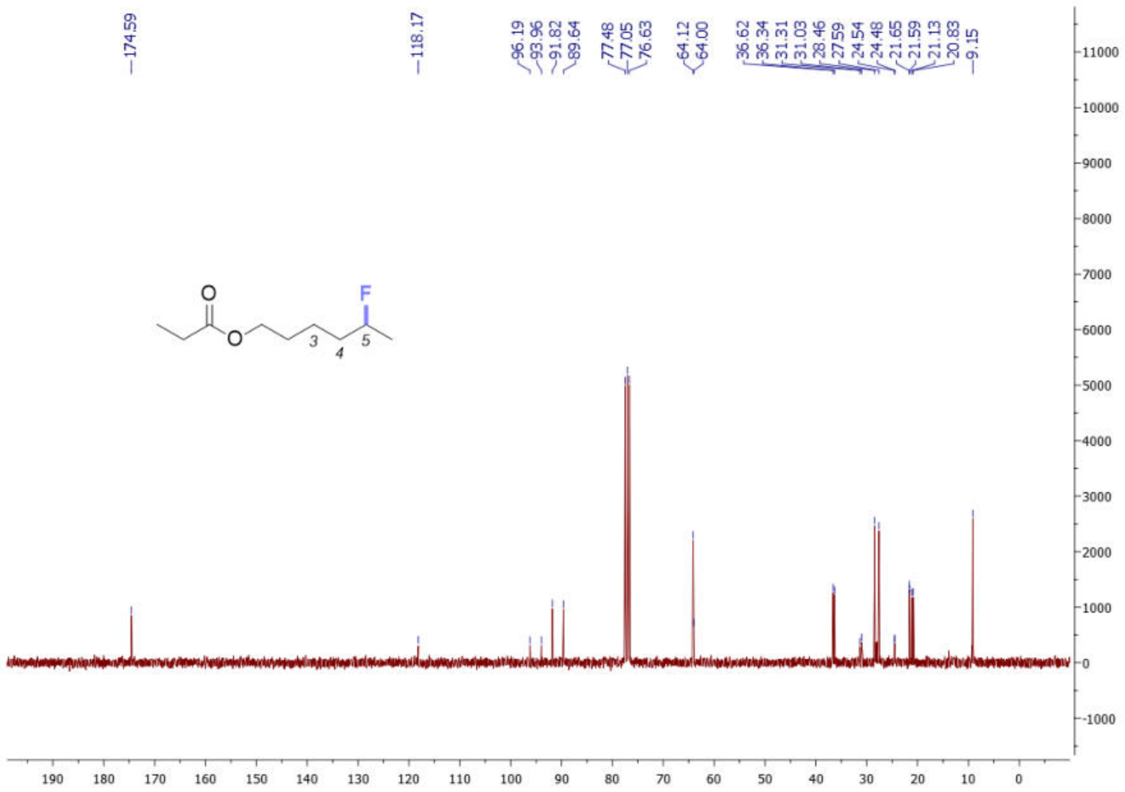

^{19}F NMR of compound **2k** in $CDCl_3$

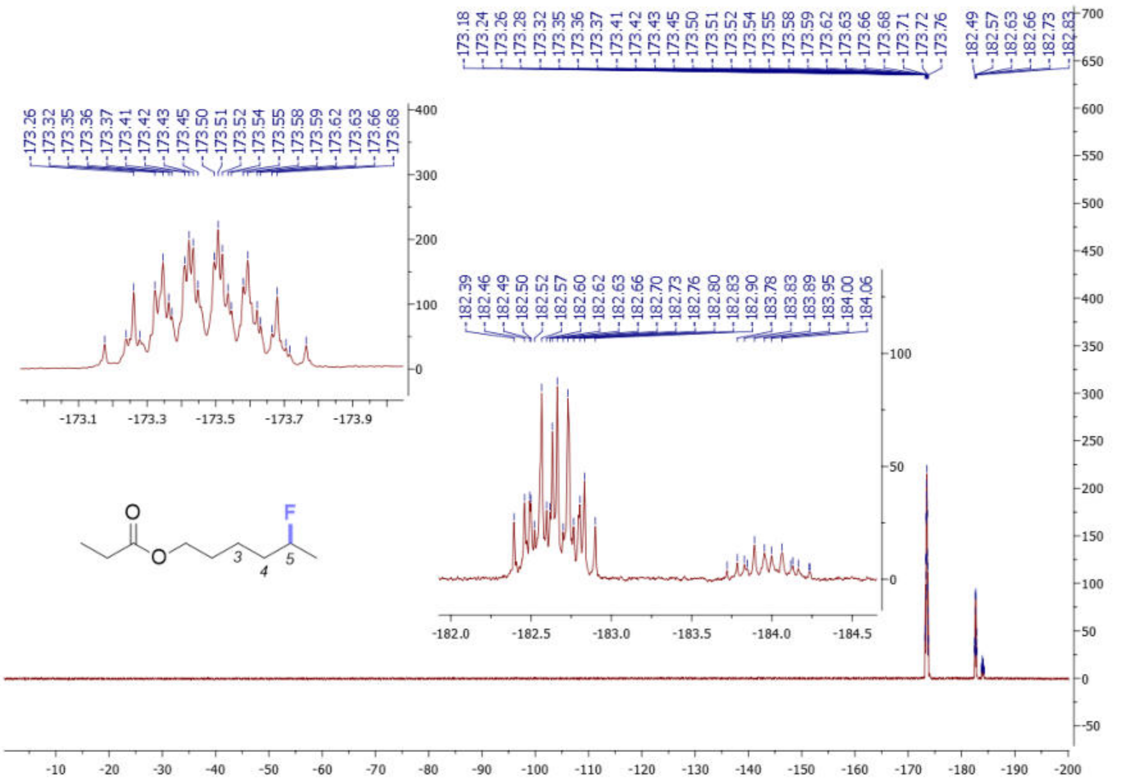

$^{19}F\{^1H\}$ NMR of compound **2k** in $CDCl_3$

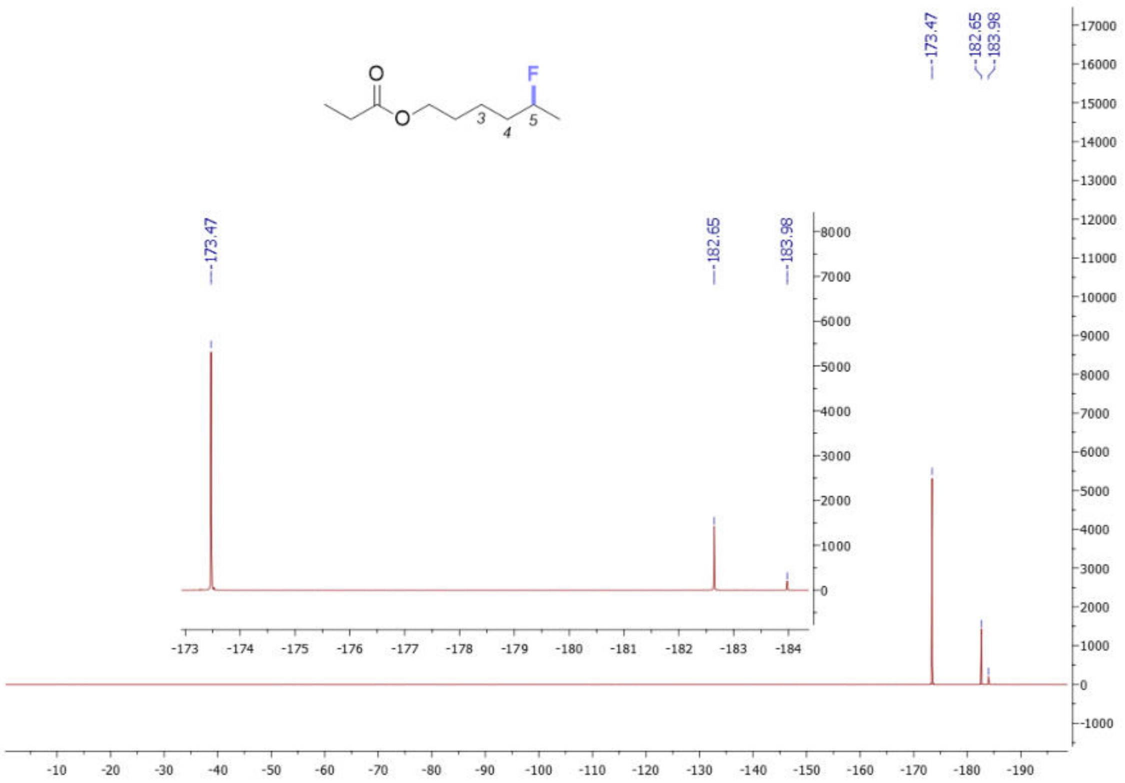

^1H NMR of compound **2l** in $CDCl_3$

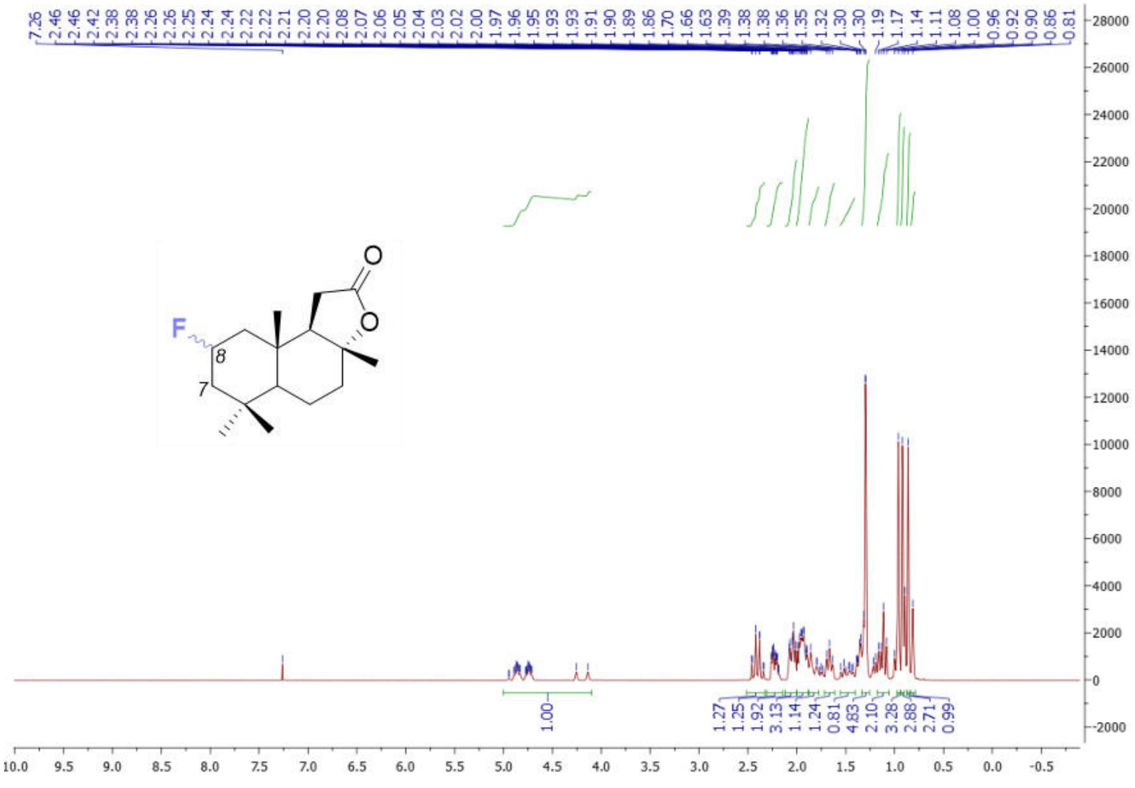

^{13}C NMR of compound **2l** in $CDCl_3$

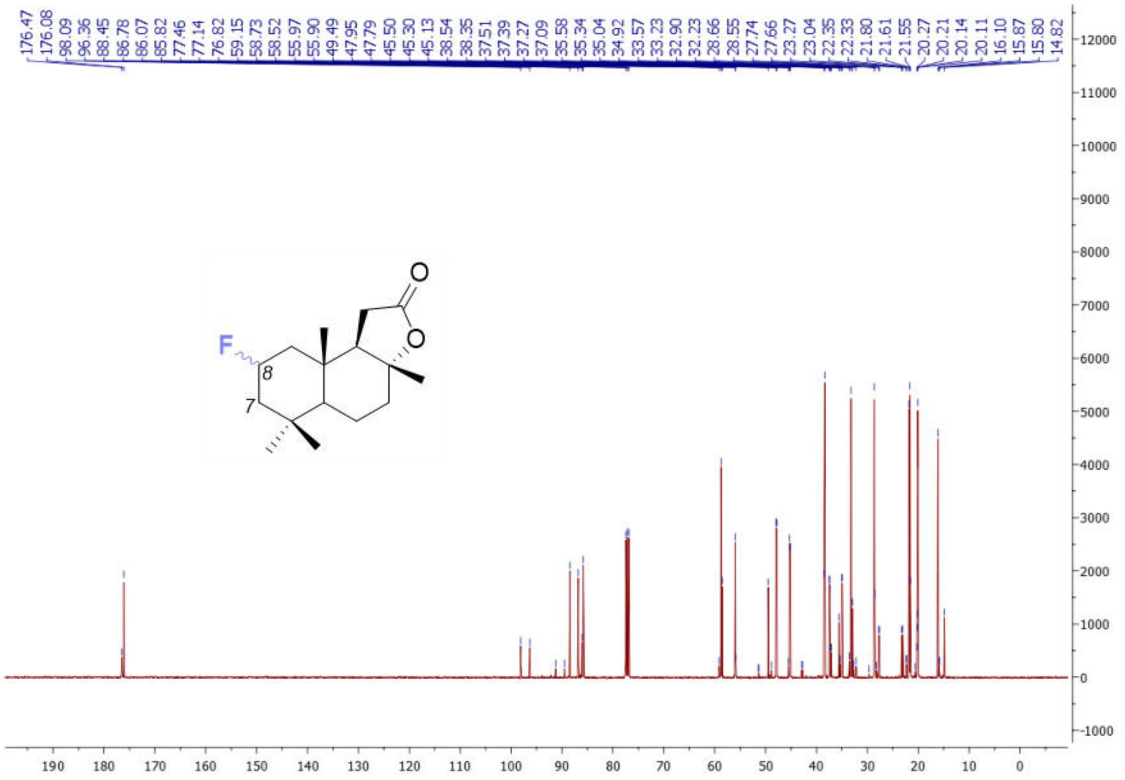

^{19}F NMR of compound **2l** in $CDCl_3$

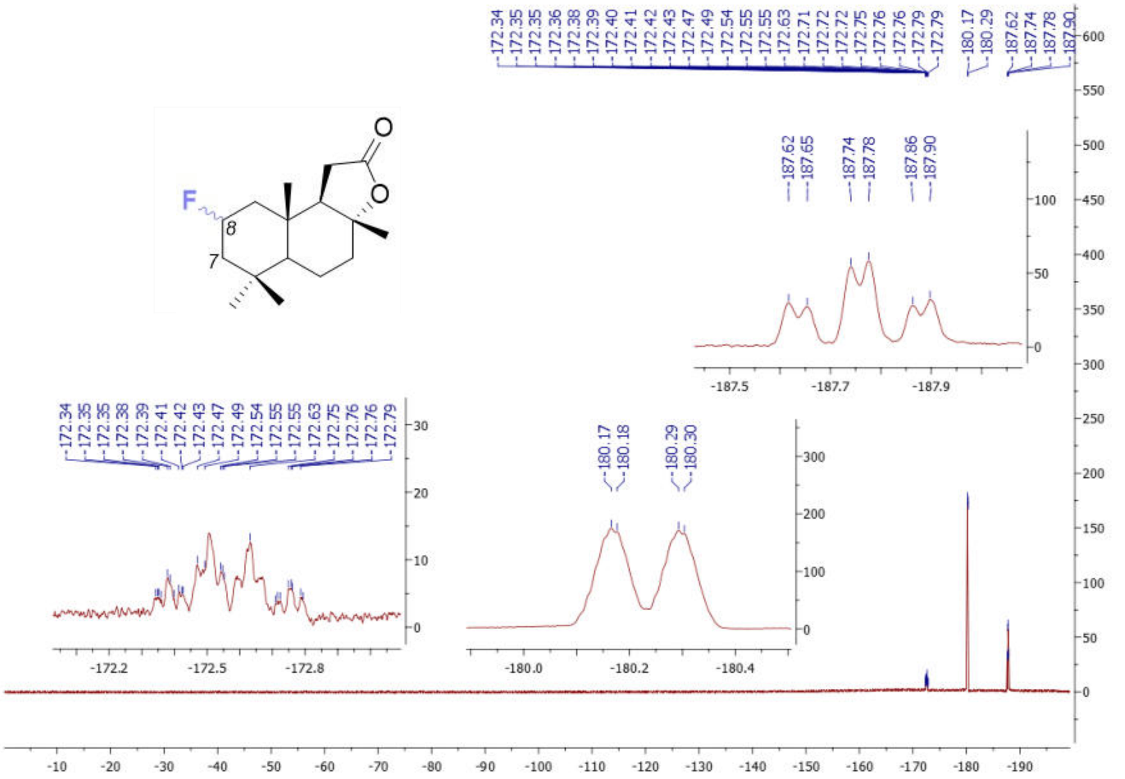

^{1}H NMR of compound **2m** in $CDCl_3$

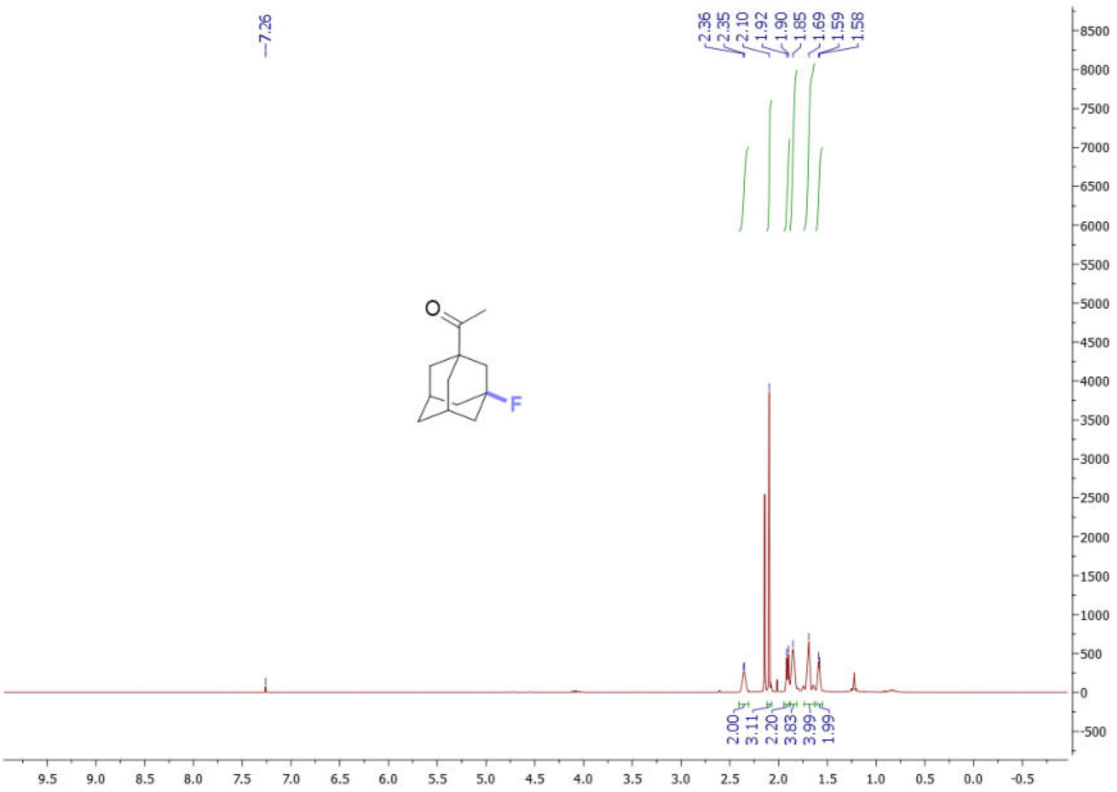

^{13}C NMR of compound **2m** in $CDCl_3$

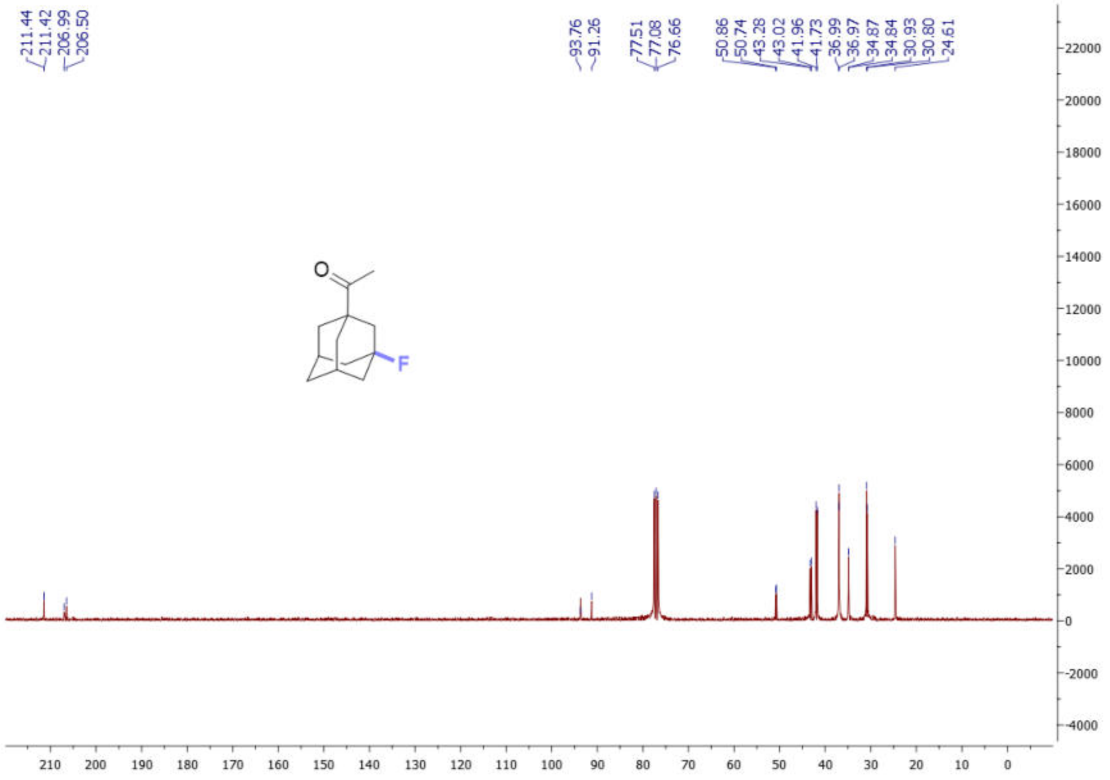

^{19}F NMR of compound **2m** in $CDCl_3$

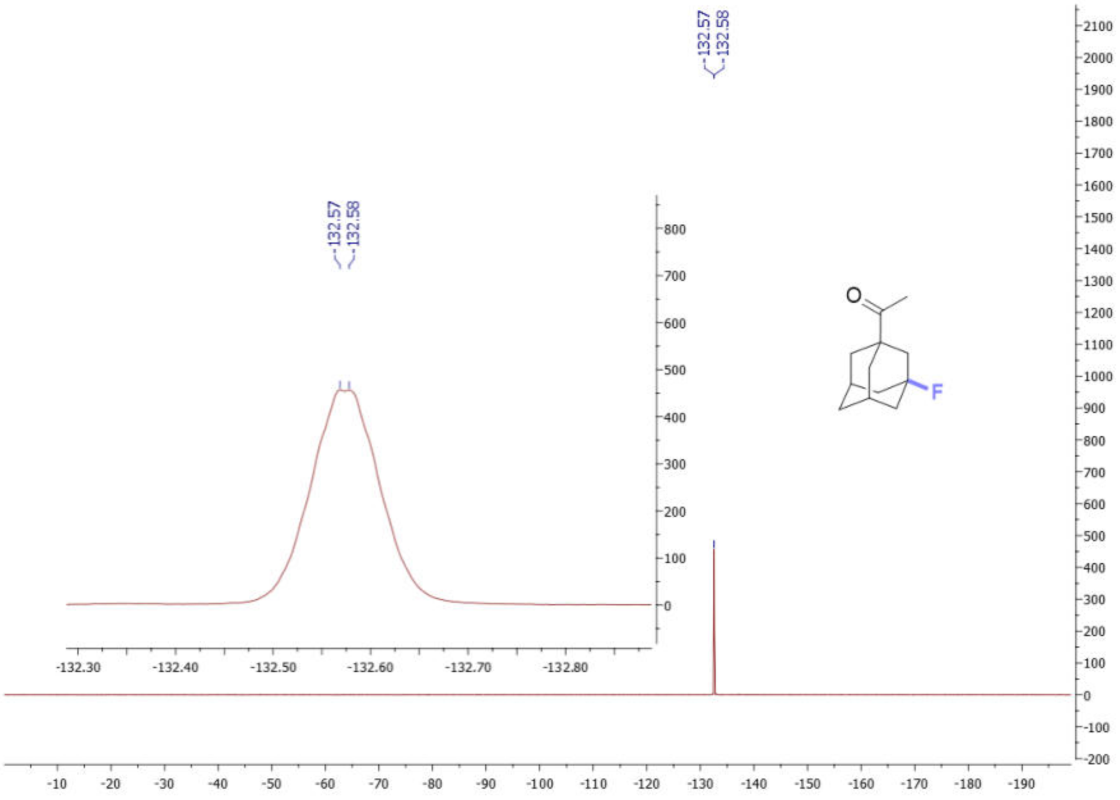

^{1}H NMR of compound **2n** in $CDCl_3$

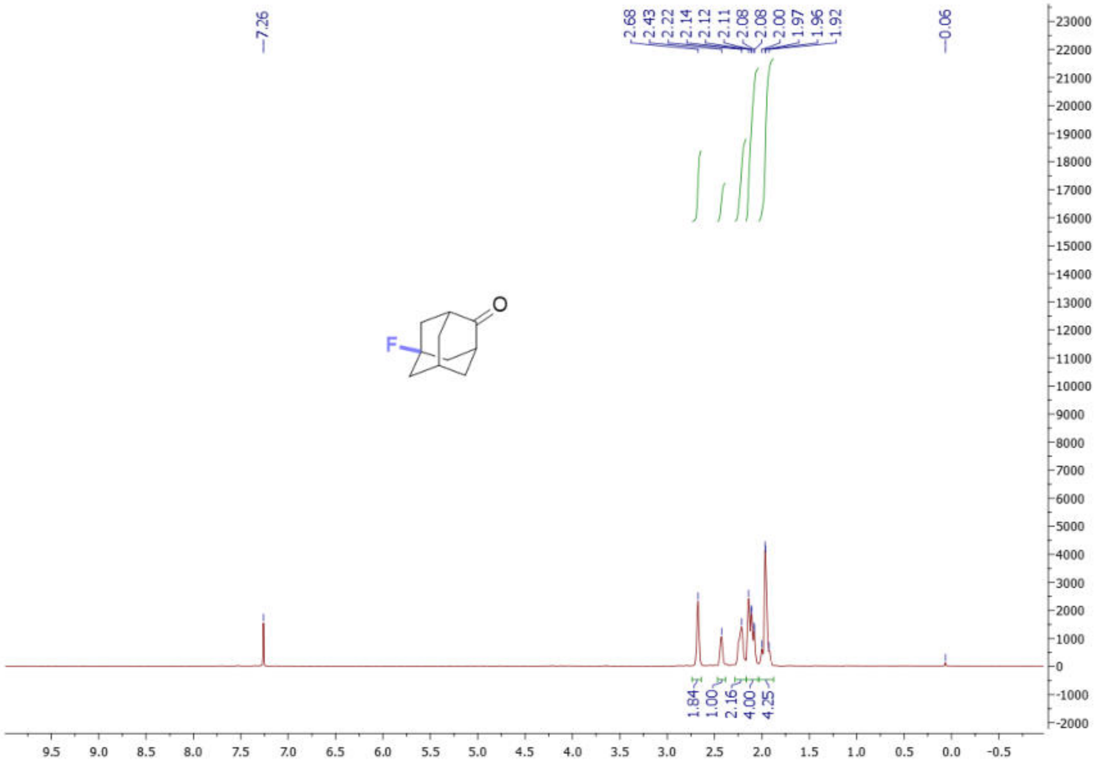

^{13}C NMR of compound **2n** in $CDCl_3$

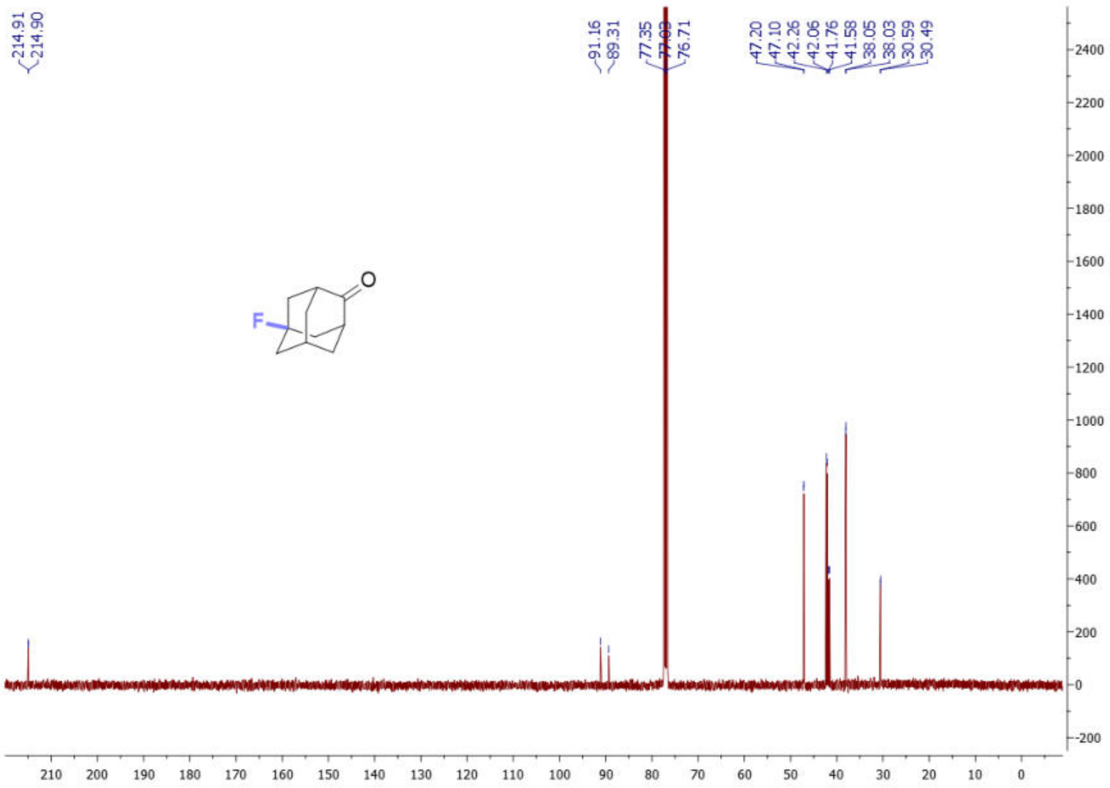

^{19}F NMR of compound **2n** in $CDCl_3$

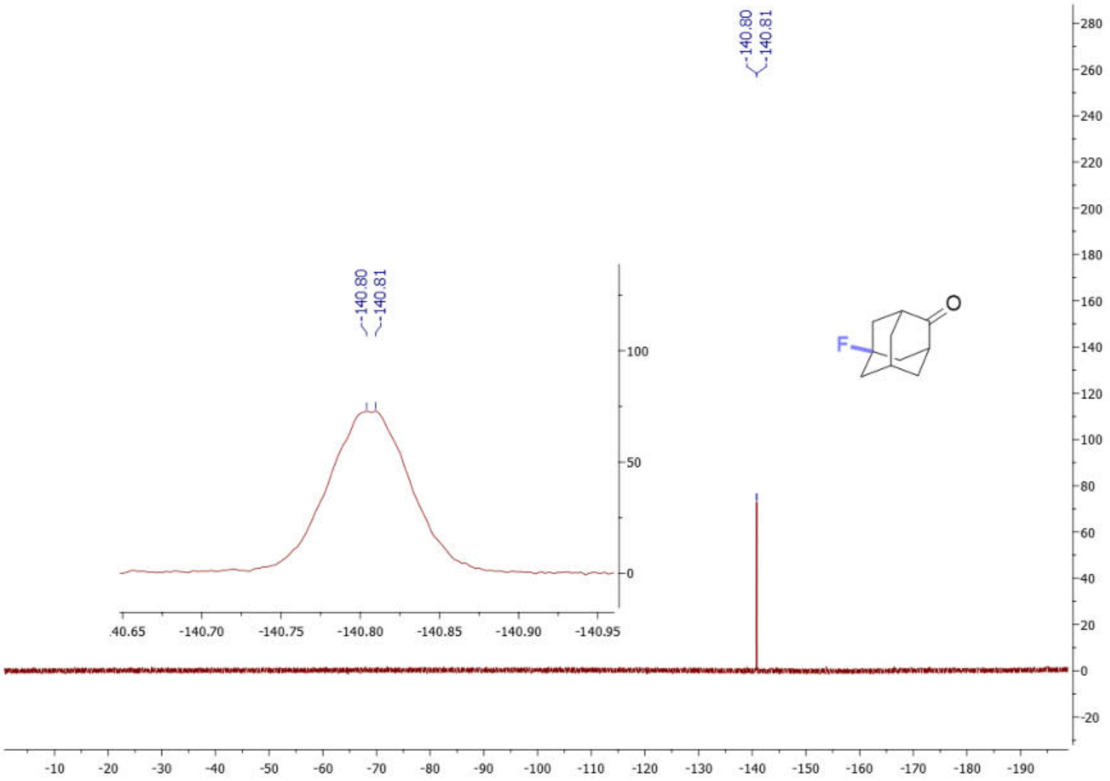

^{1}H NMR of compound **2q** in $CDCl_3$

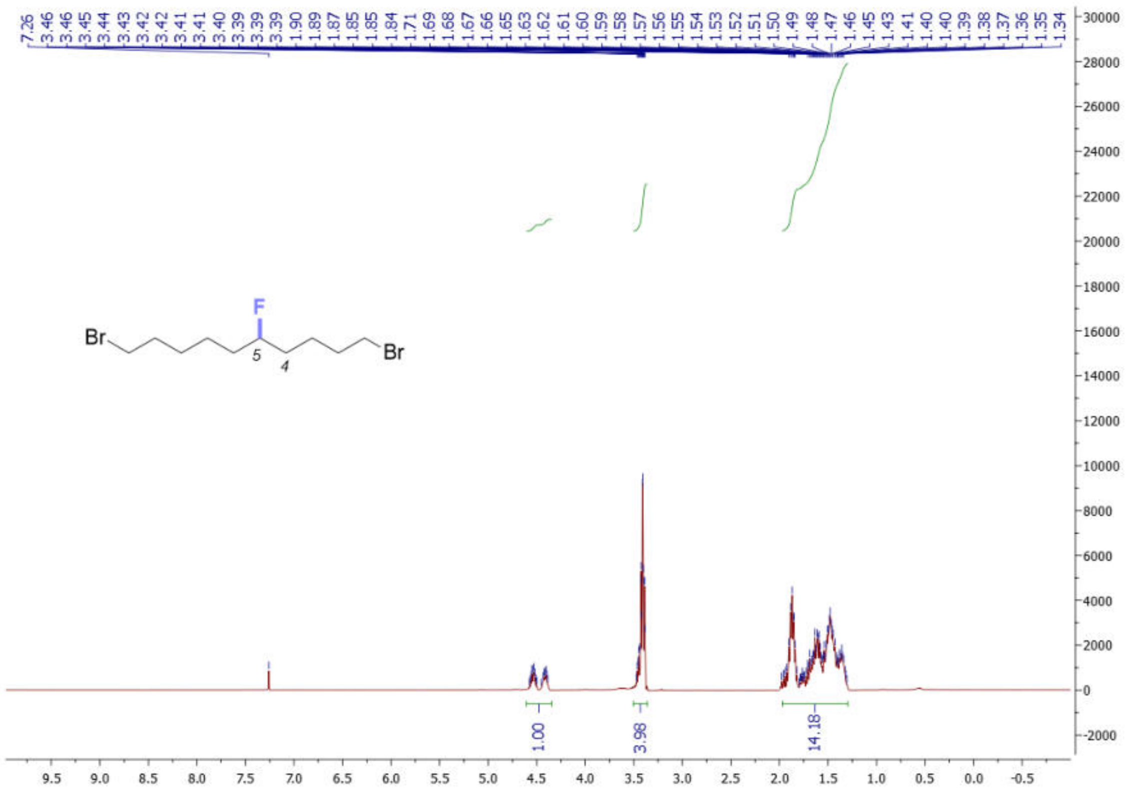

^{13}C NMR of compound **2q** in $CDCl_3$

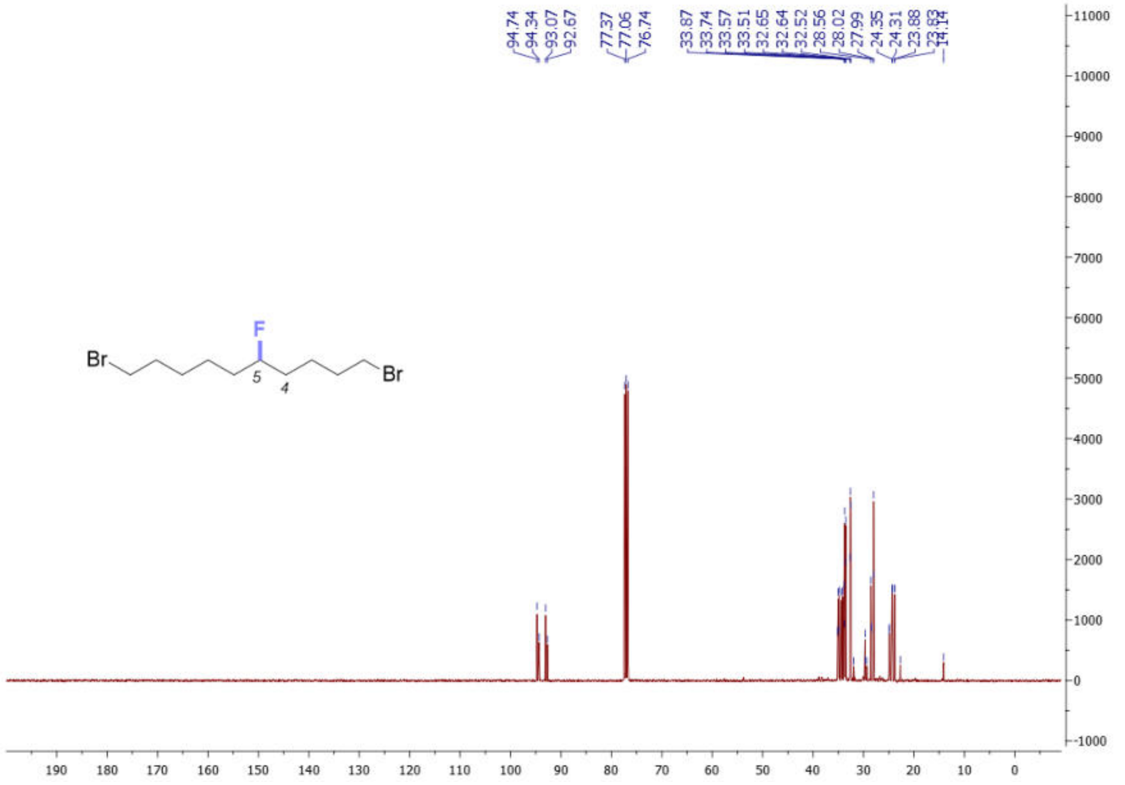

^{19}F NMR of compound **2q** in $CDCl_3$

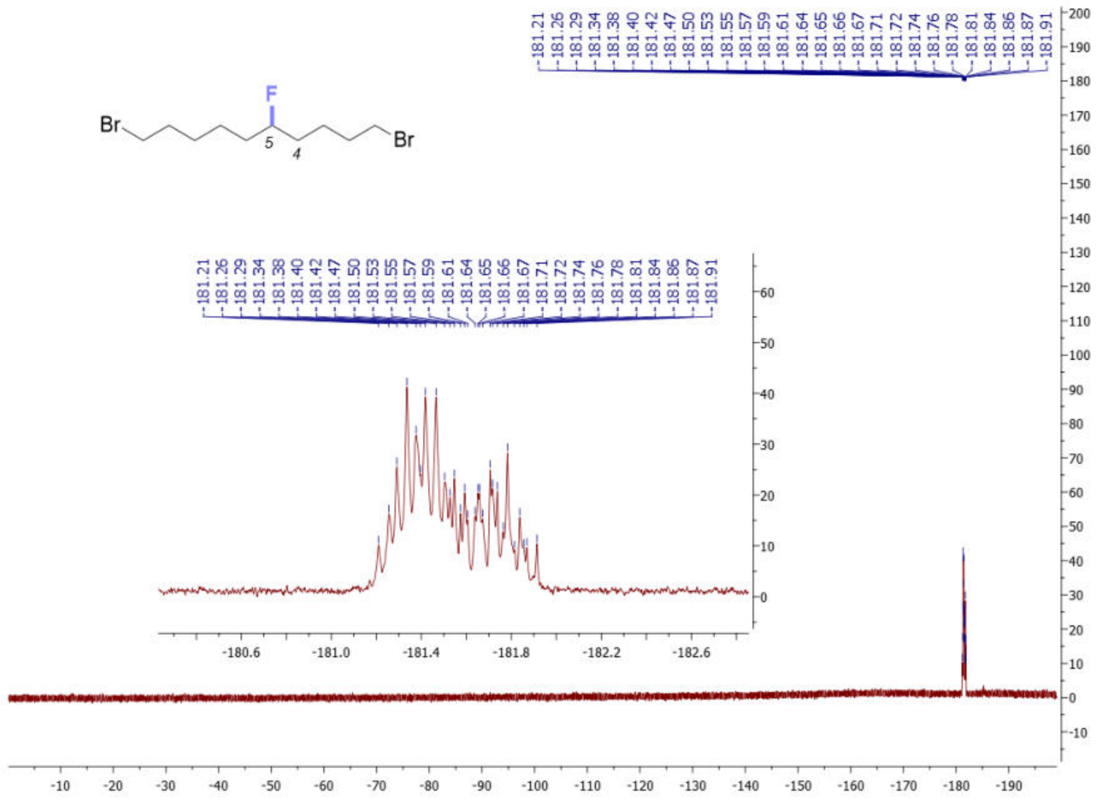

$^{19}F\{^1H\}$ NMR of compound **2q** in $CDCl_3$

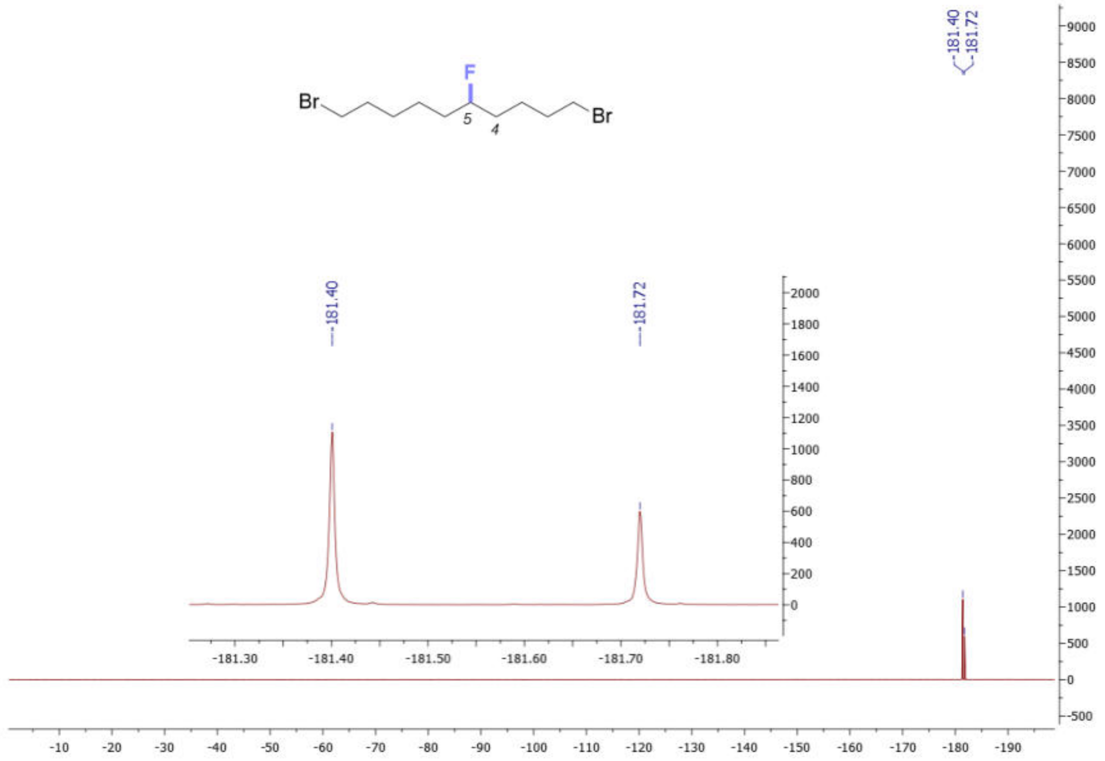

^{1}H NMR of compound **2r** in $CDCl_3$

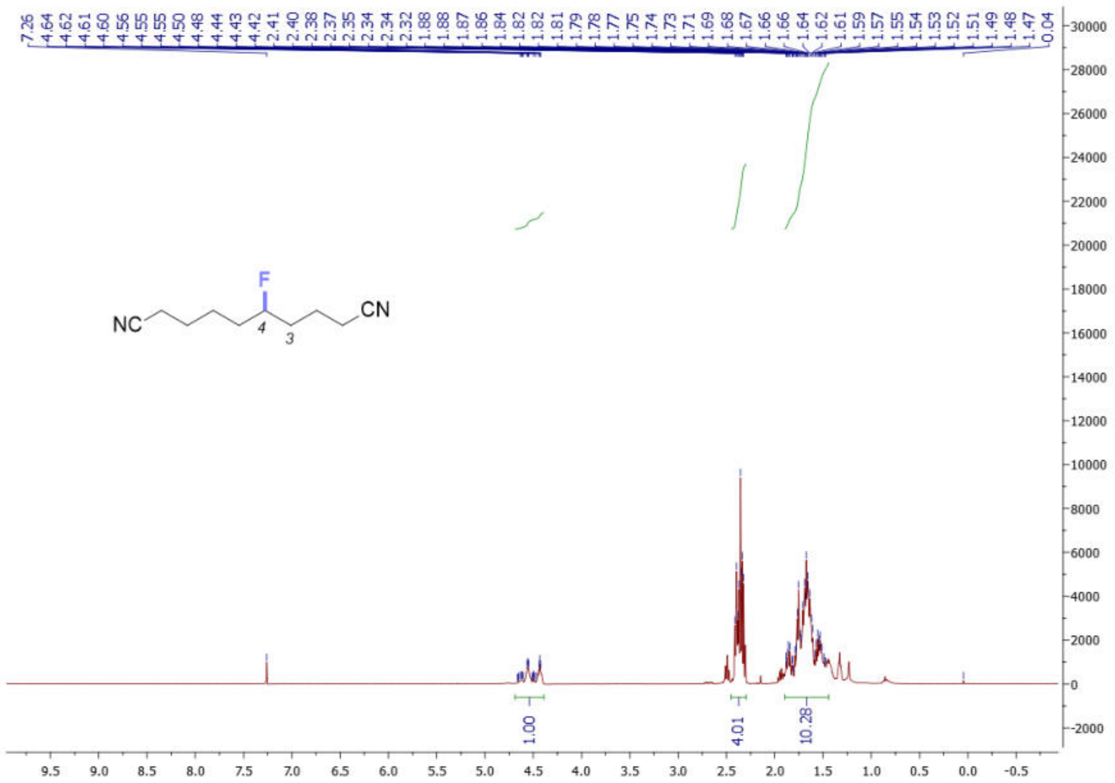

^{13}C NMR of compound **2r** in $CDCl_3$

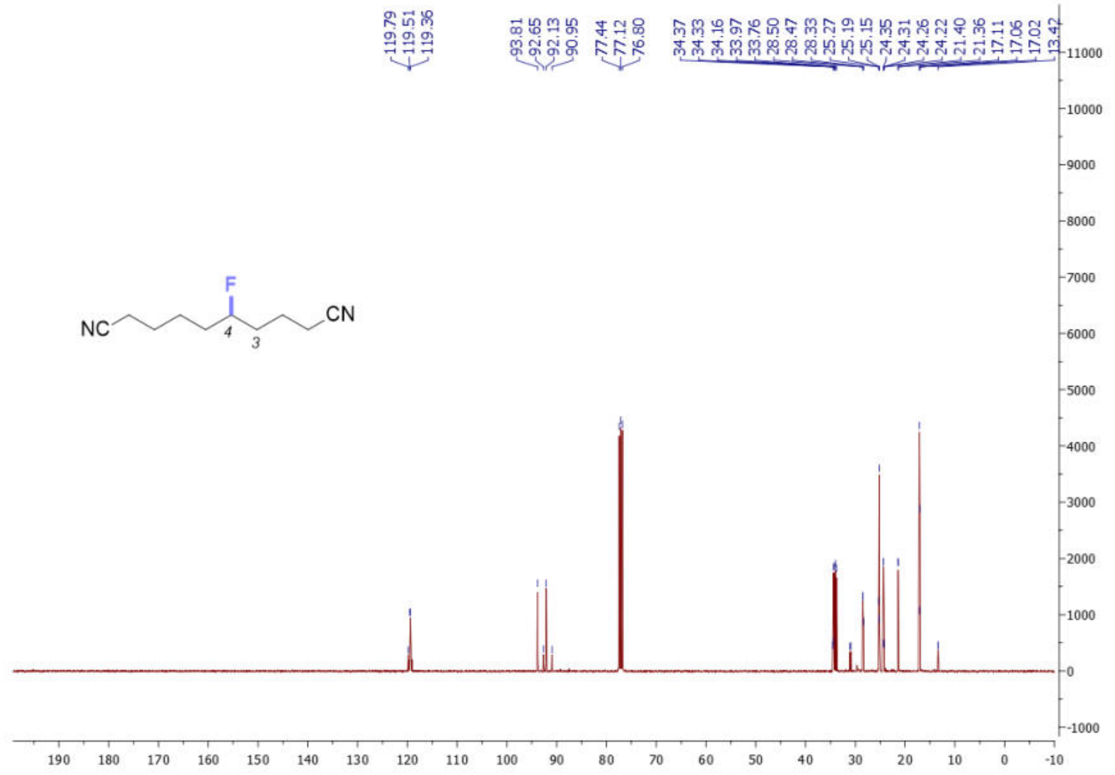

^{19}F NMR of compound **2r** in $CDCl_3$

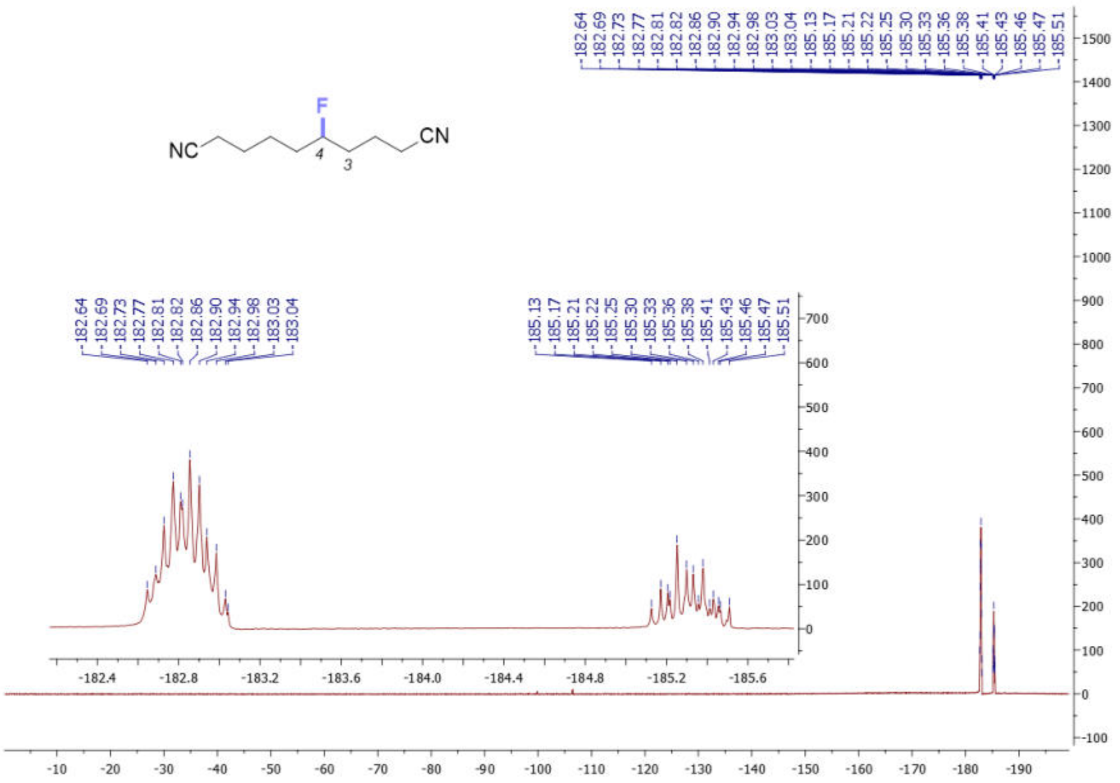

^{19}F{^{1}H} NMR of compound **2r** in $CDCl_3$

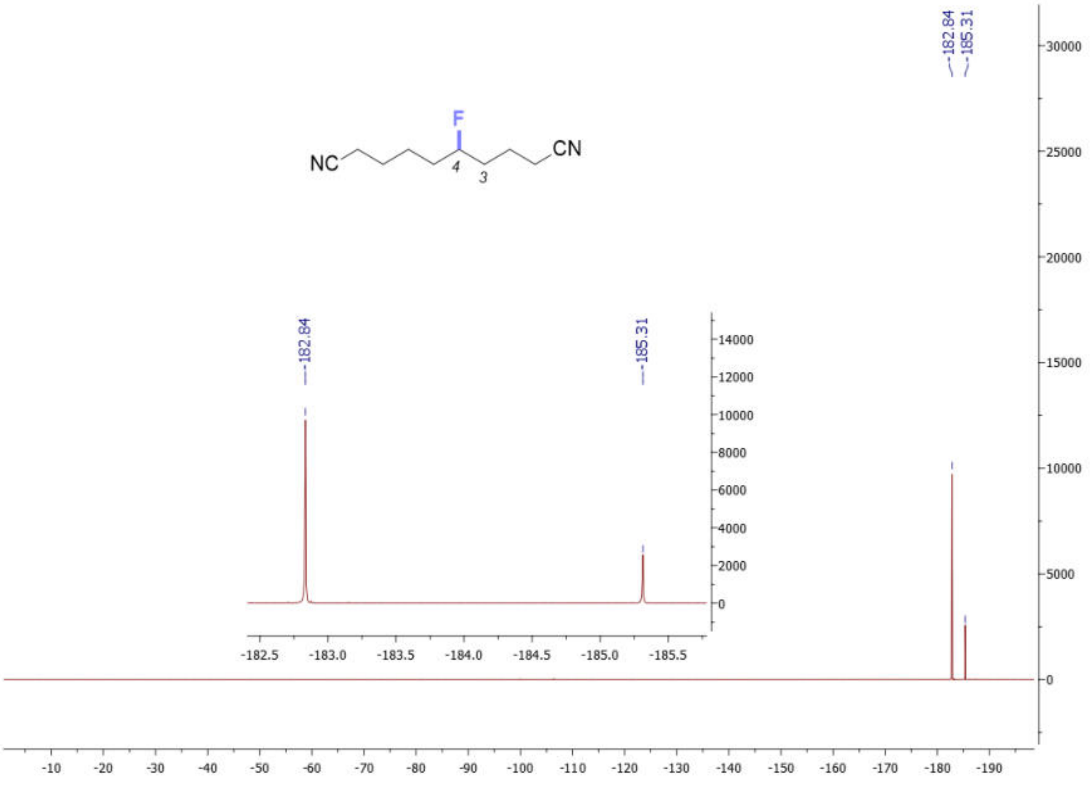

^{1}H NMR of compound **2w** in $CDCl_3$

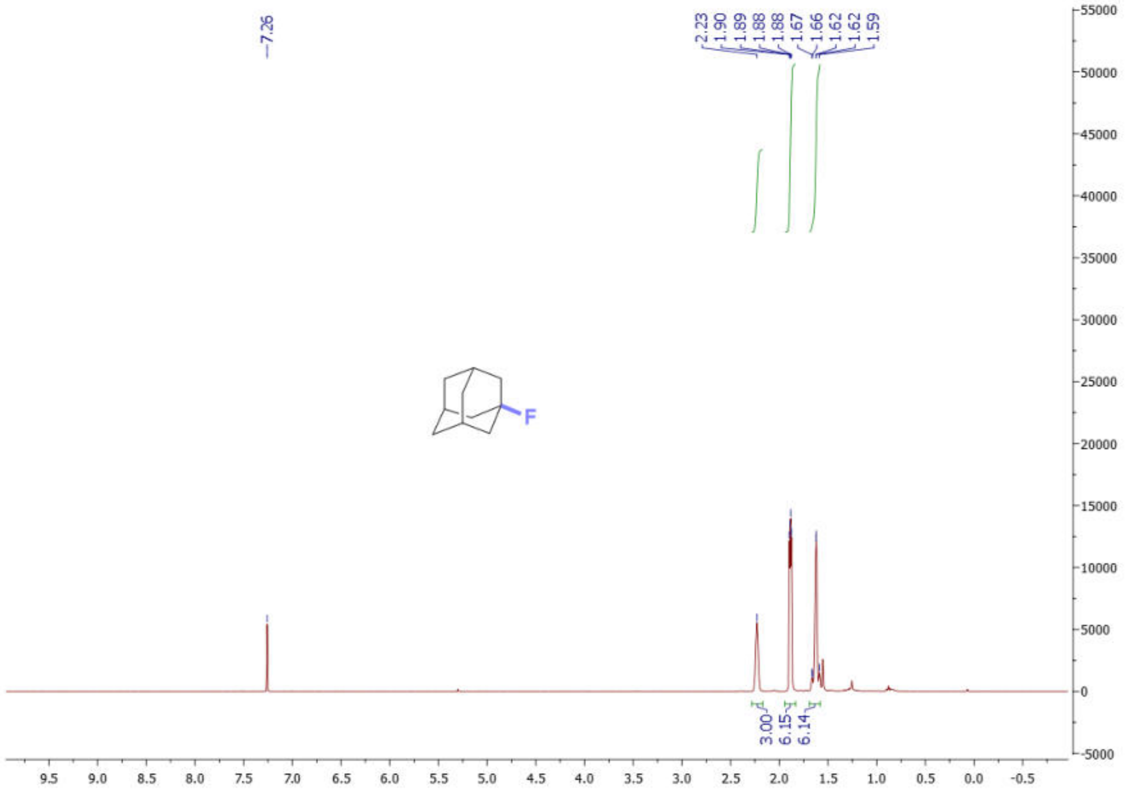

^{13}C NMR of compound **2w** in $CDCl_3$

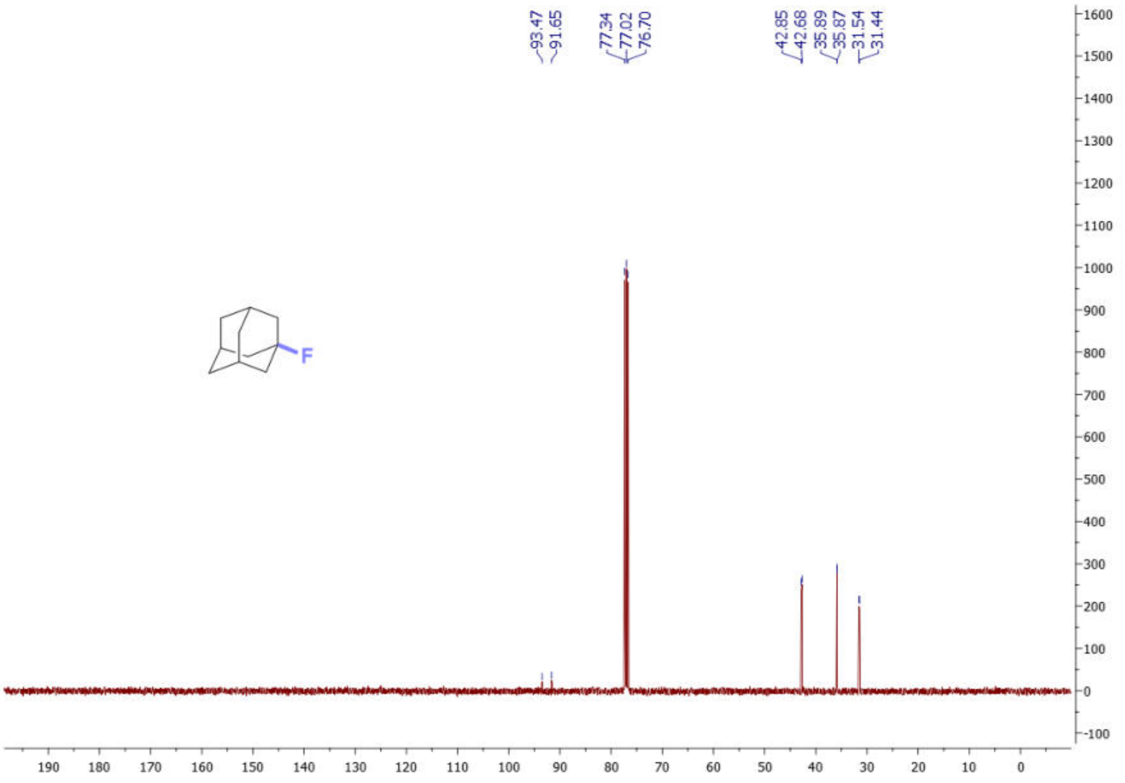

^{19}F NMR of compound **2w** in $CDCl_3$

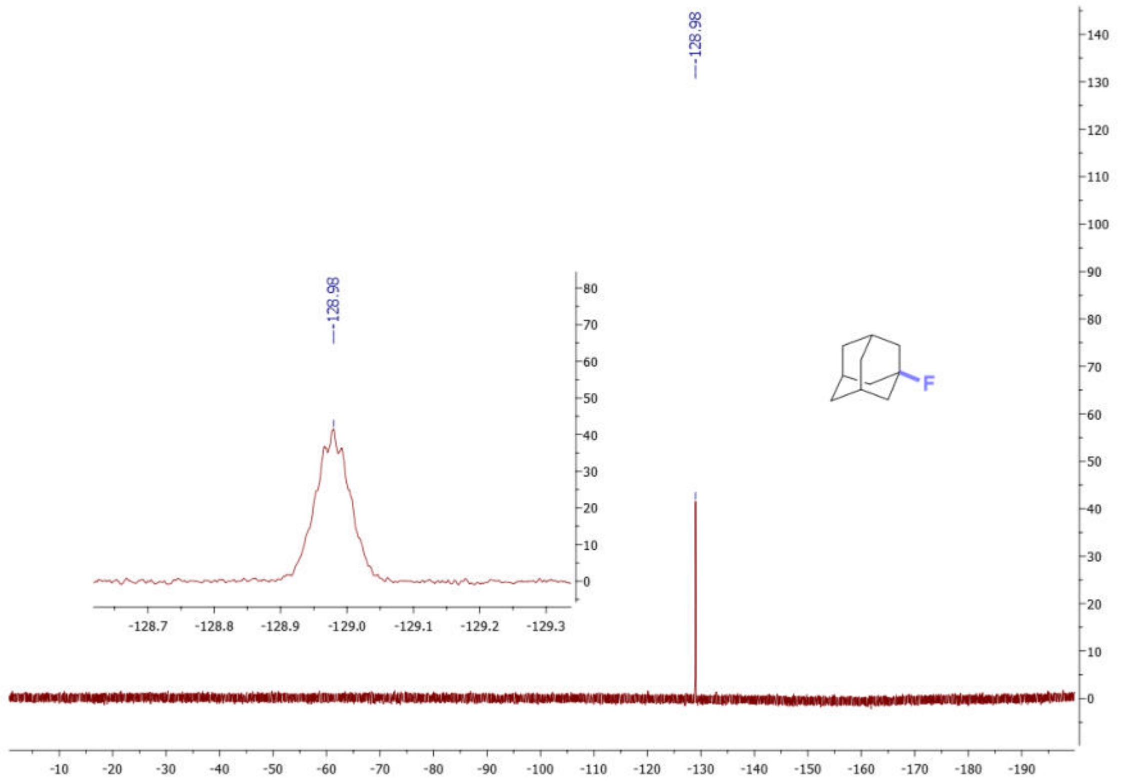

^{1}H NMR of compound **2b** in $CDCl_3$

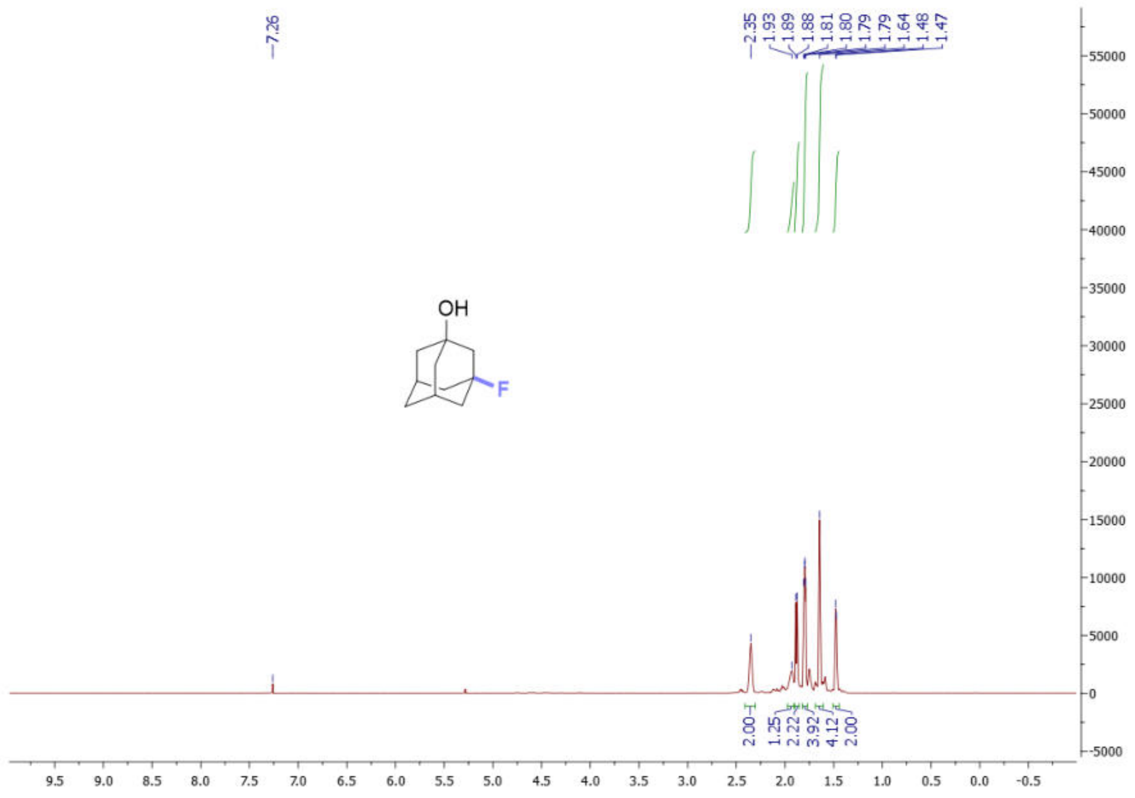

^{13}C NMR of compound **2b** in $CDCl_3$

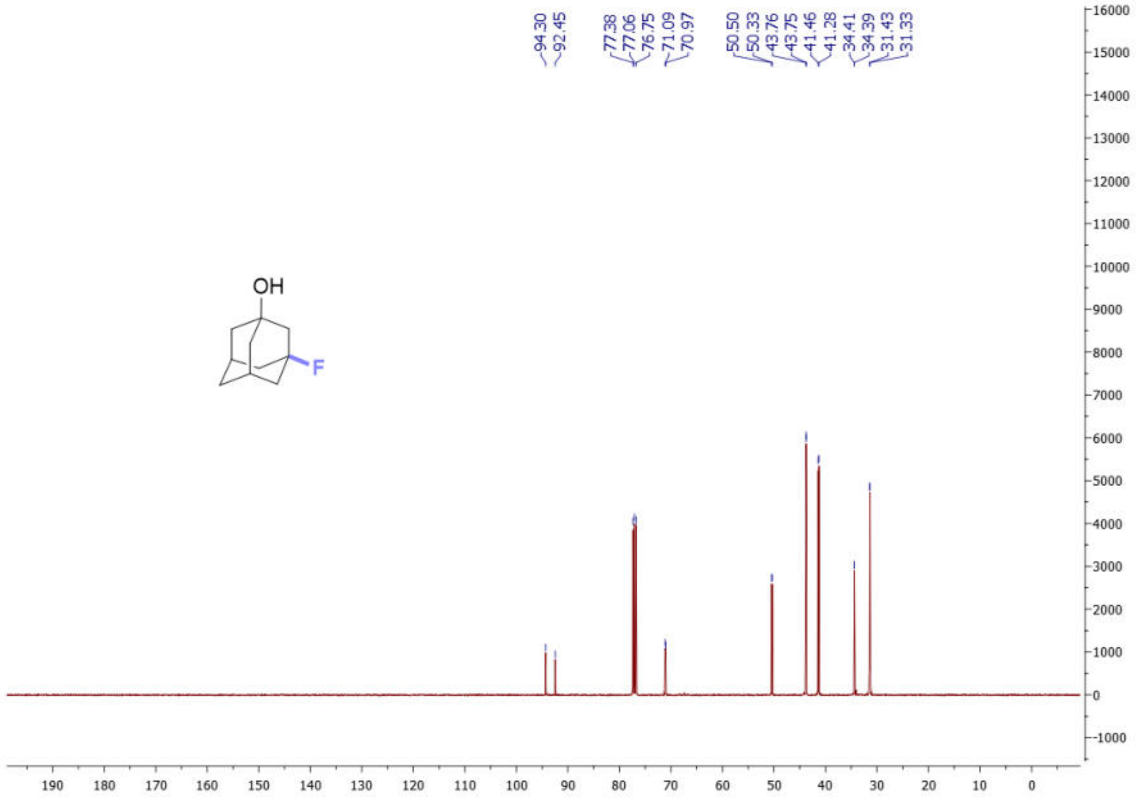

^{19}F NMR of compound **2b** in $CDCl_3$

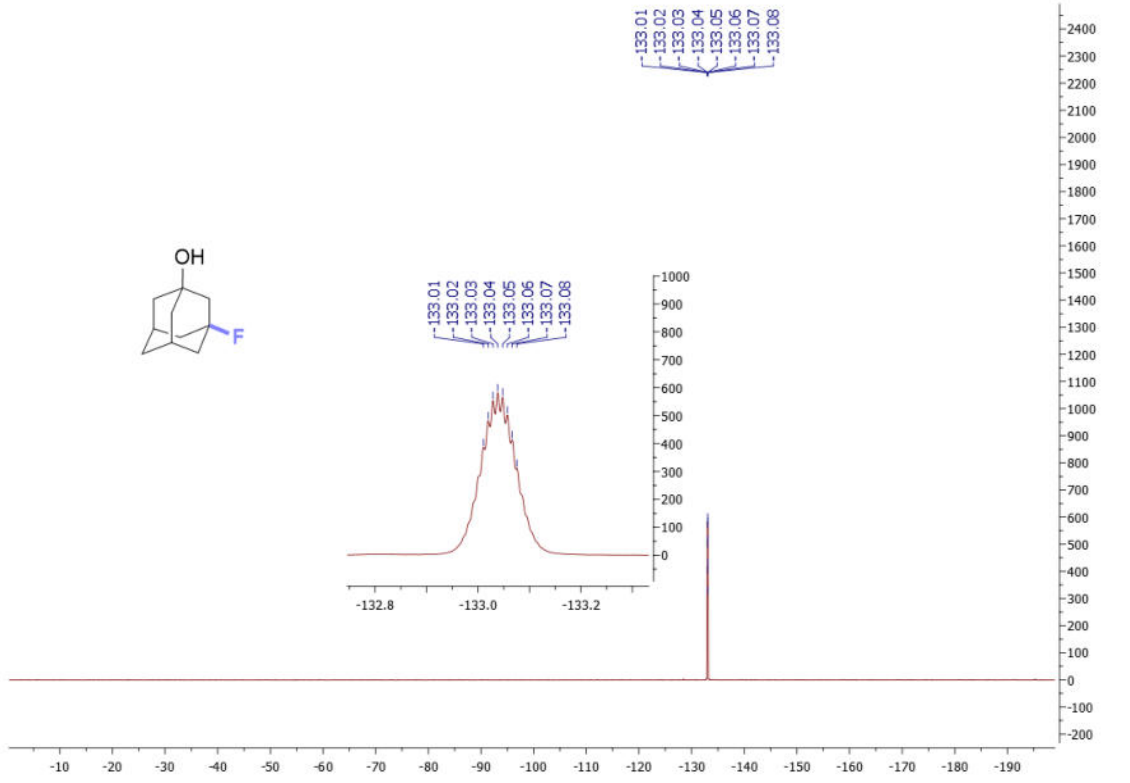

^{1}H NMR of compound **2a** in $CDCl_3$

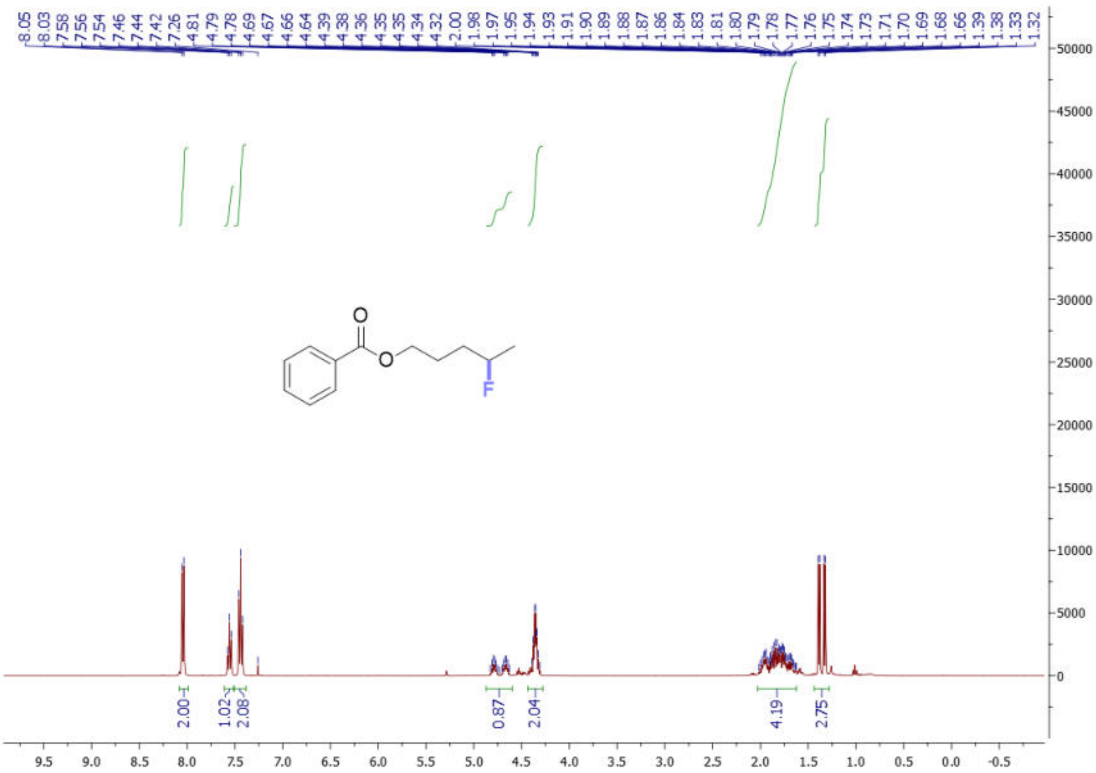

^{13}C NMR of compound **2a** in $CDCl_3$

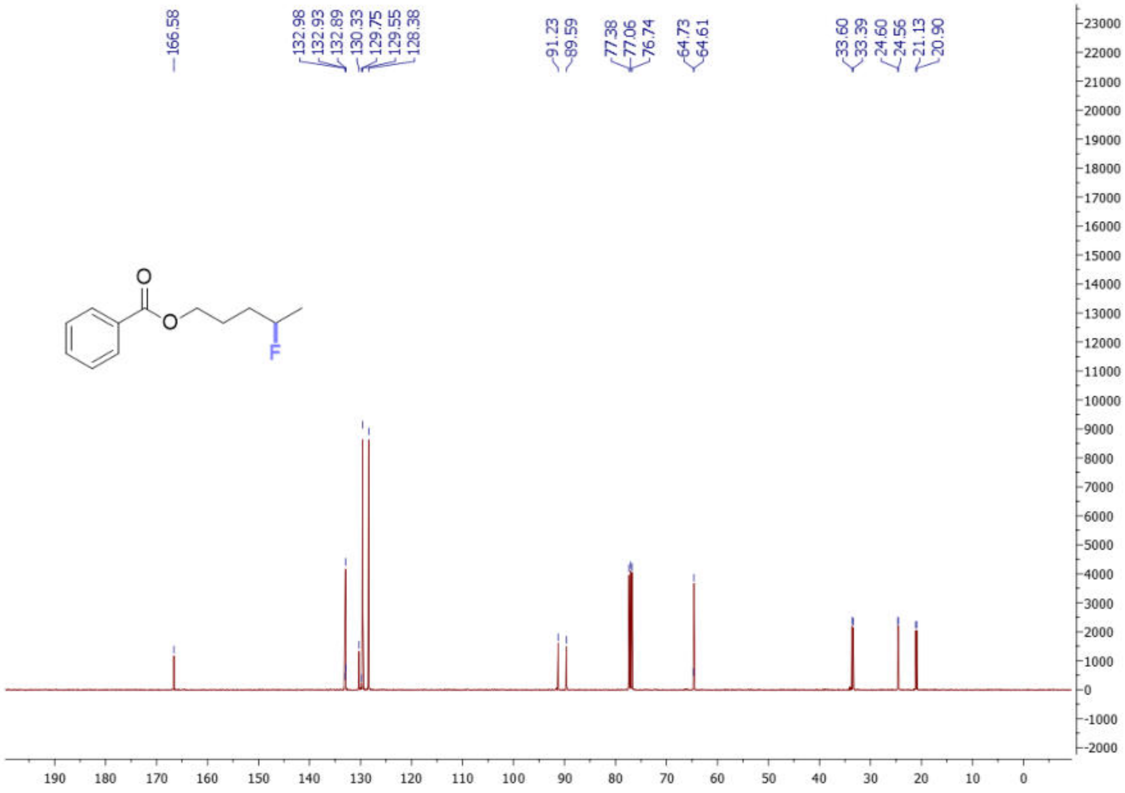

^{19}F NMR of compound **2a** in $CDCl_3$

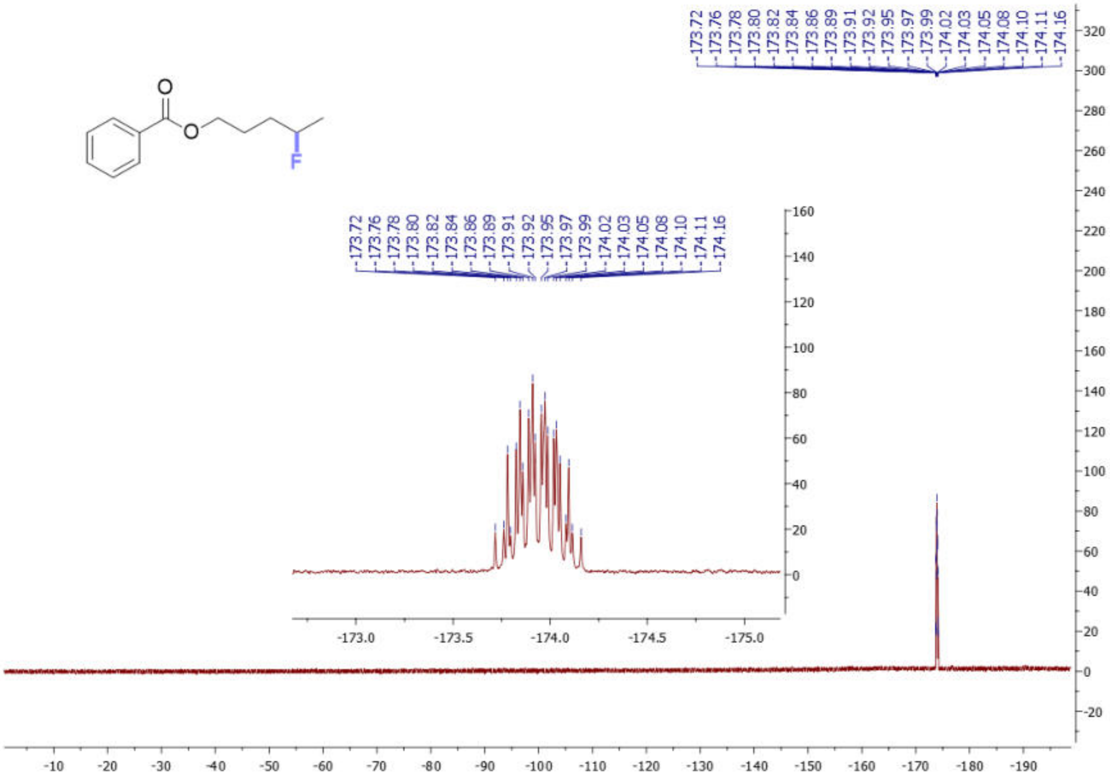

^{1}H NMR of compound **2f** in $CDCl_3$

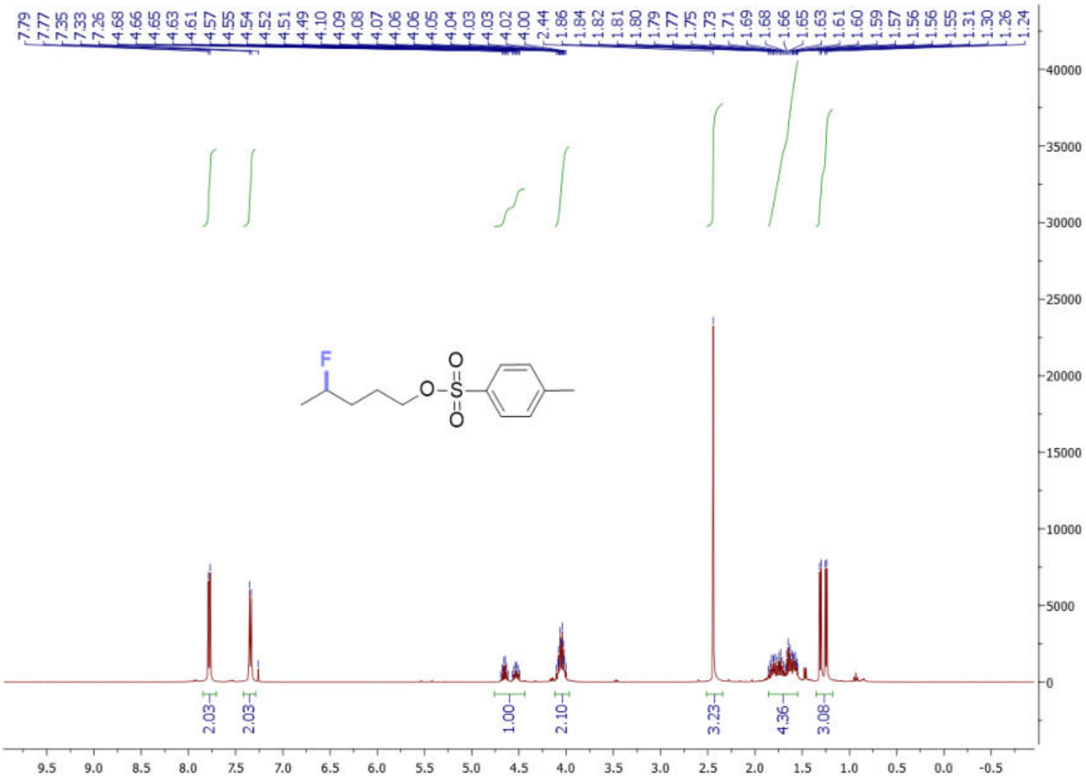

^{13}C NMR of compound **2f** in $CDCl_3$

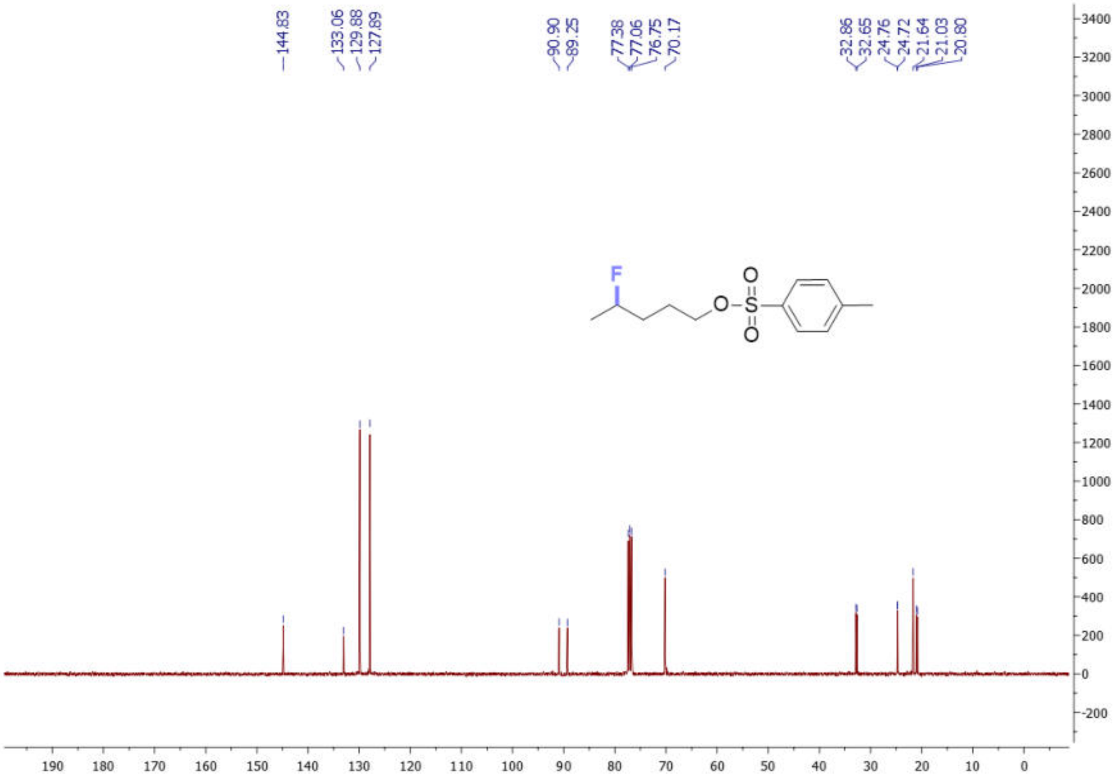

^{19}F NMR of compound **2f** in $CDCl_3$

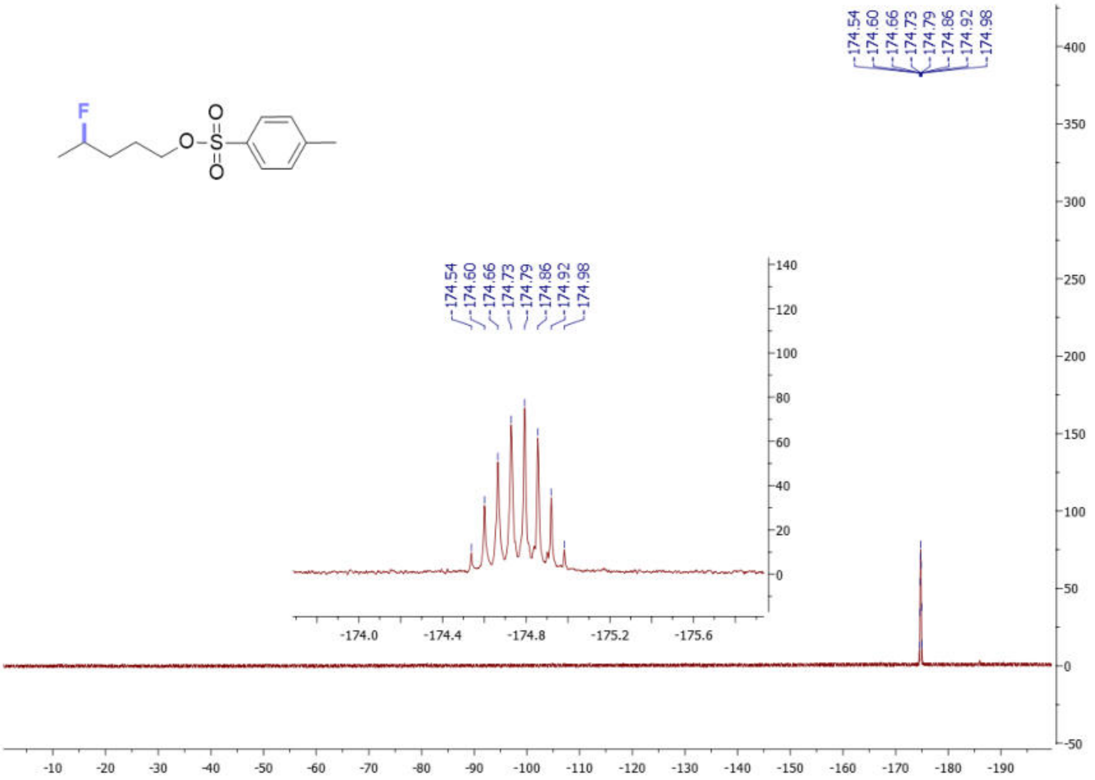

^{1}H NMR of compound **2c** in $CDCl_3$

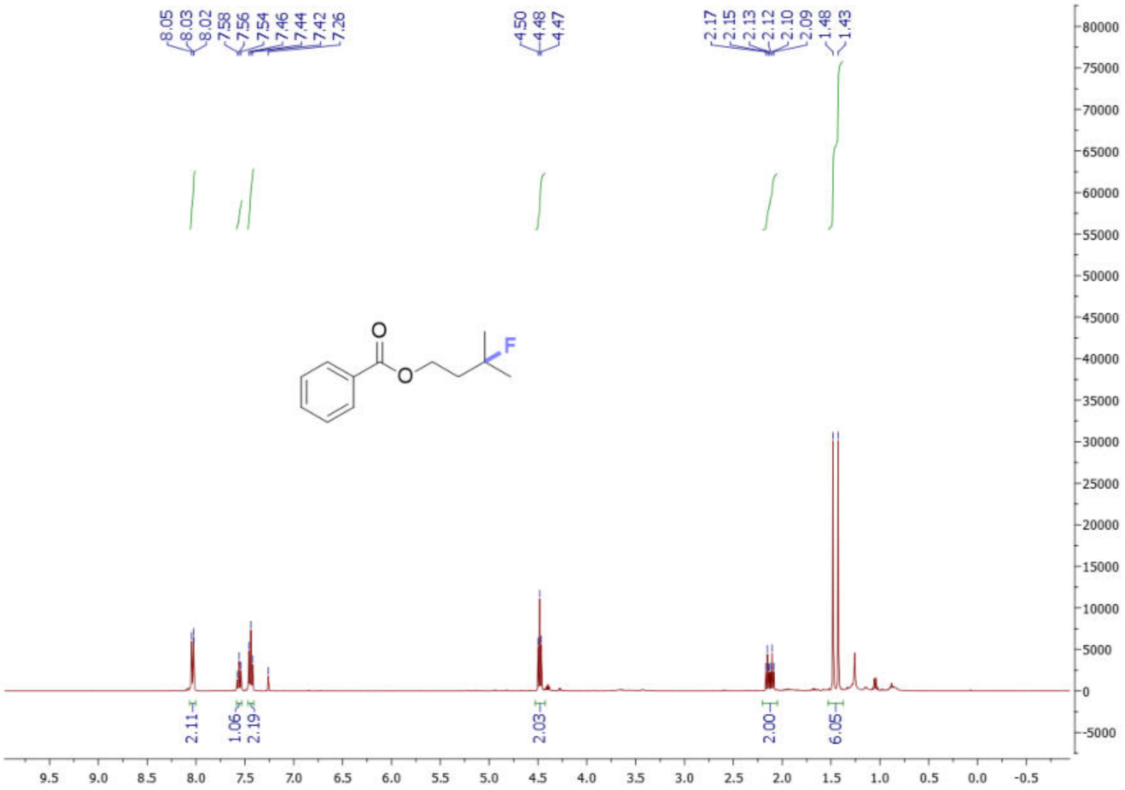

^{13}C NMR of compound **2c** in $CDCl_3$

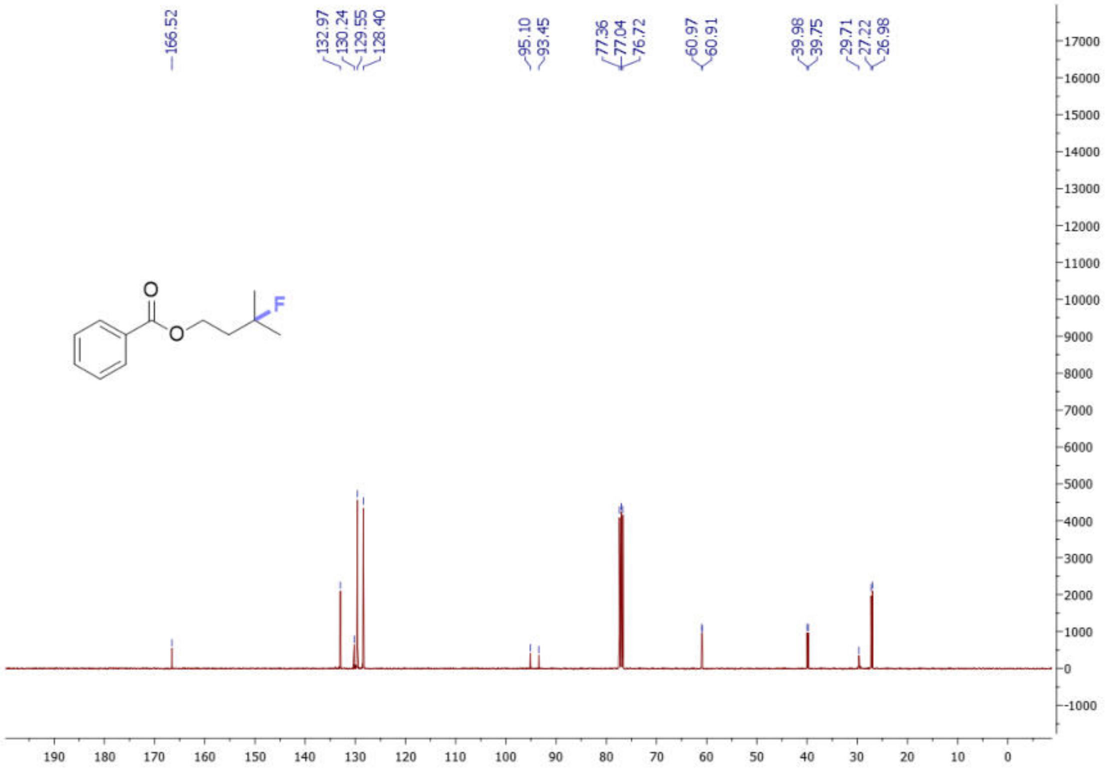

^{19}F NMR of compound **2c** in $CDCl_3$

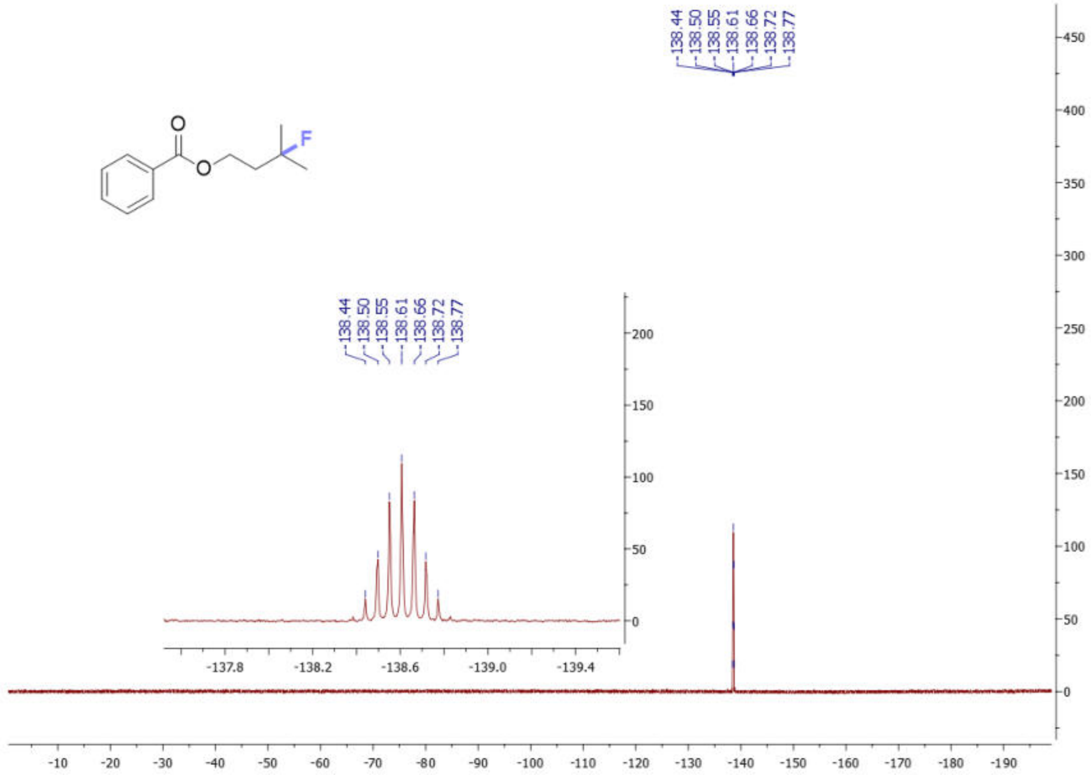

^{1}H NMR of compound **17** in $CDCl_3$

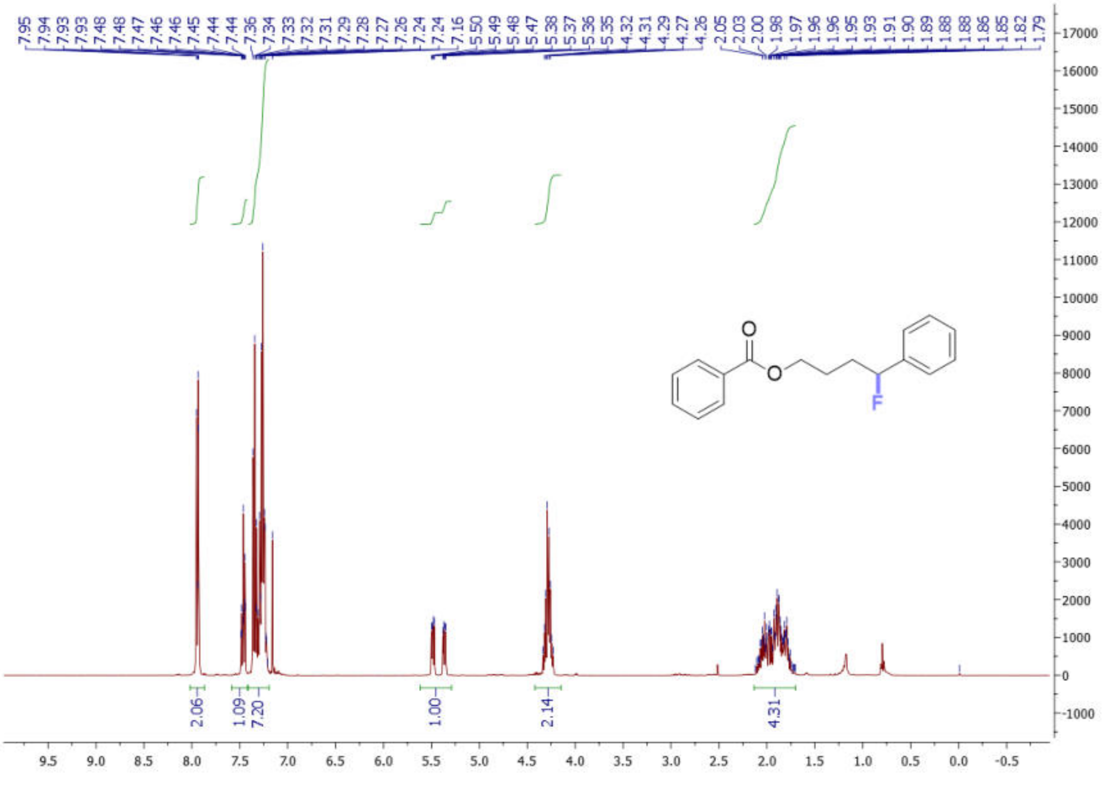

^{13}C NMR of compound **17** in $CDCl_3$

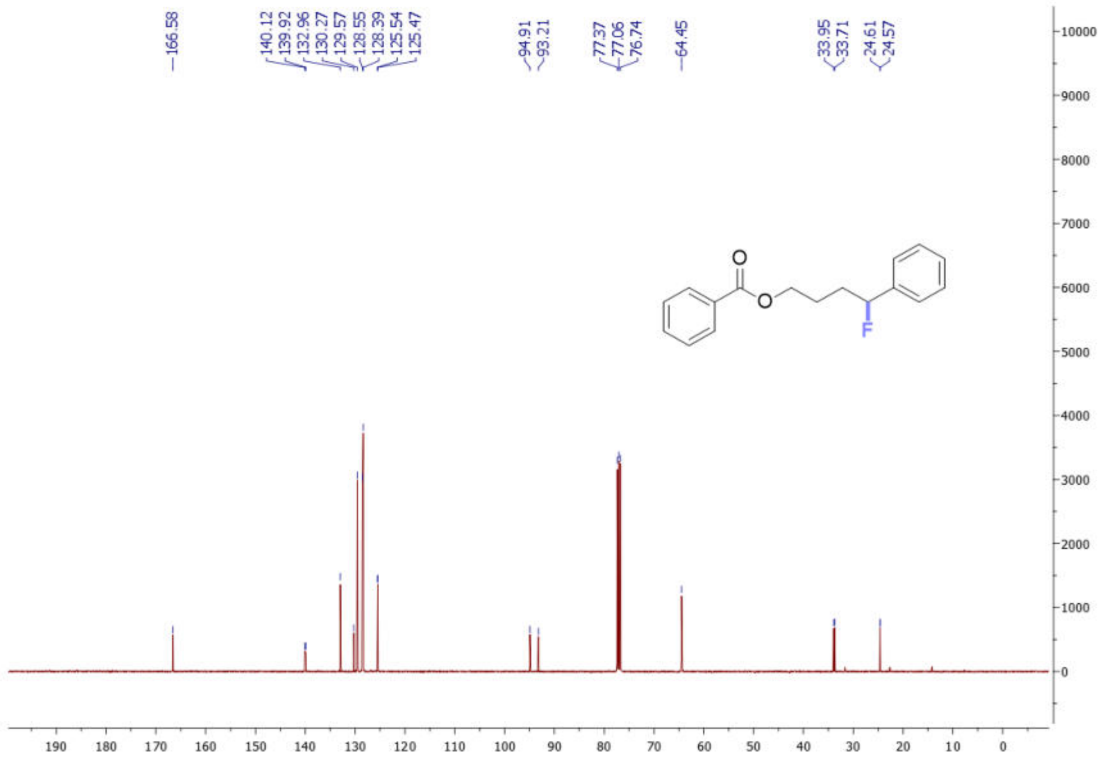

^{19}F NMR of compound **17** in $CDCl_3$

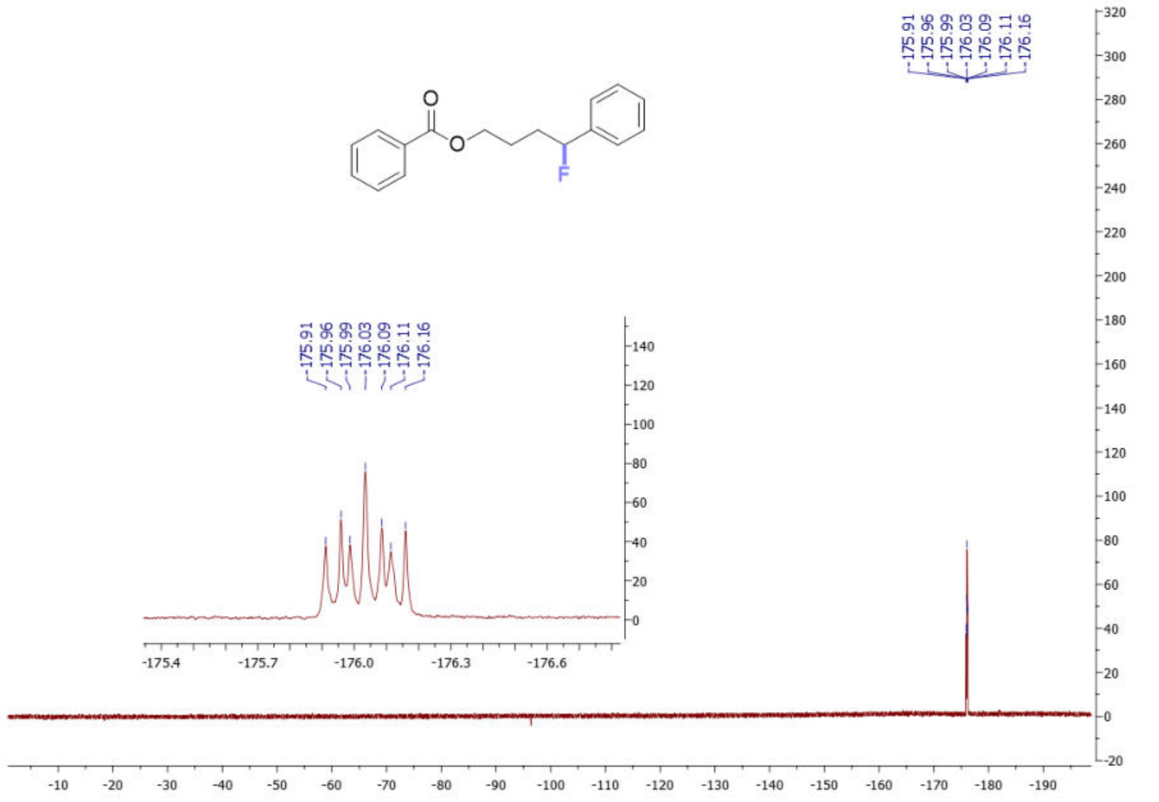

^{1}H NMR of compound **2g** in $CDCl_3$

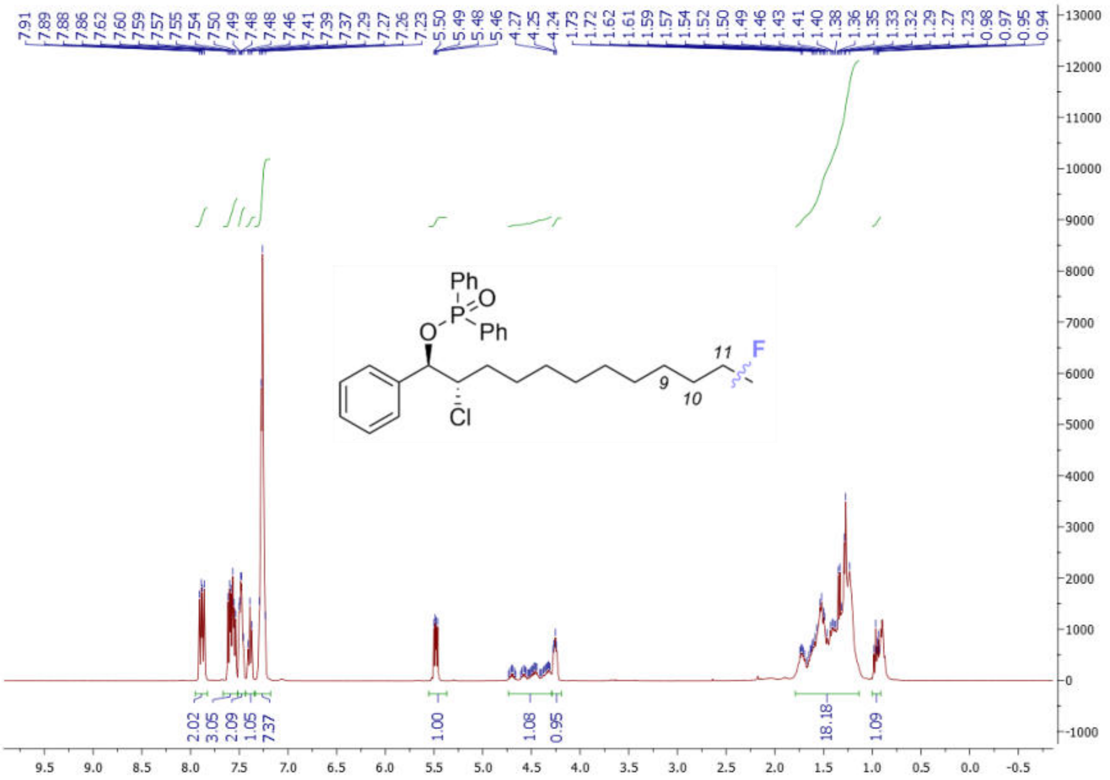

^{13}C NMR of compound **2g** in $CDCl_3$

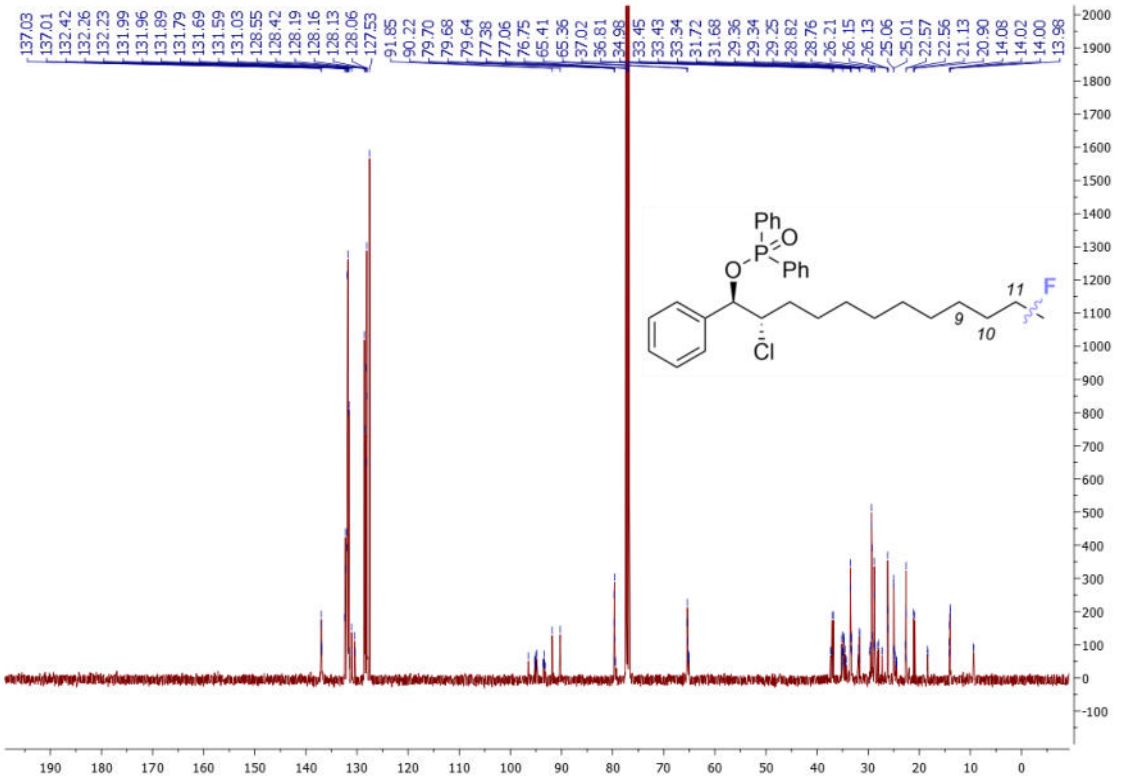

$^{19}F\{^{1}H\}$ NMR of compound **2g** in $CDCl_3$

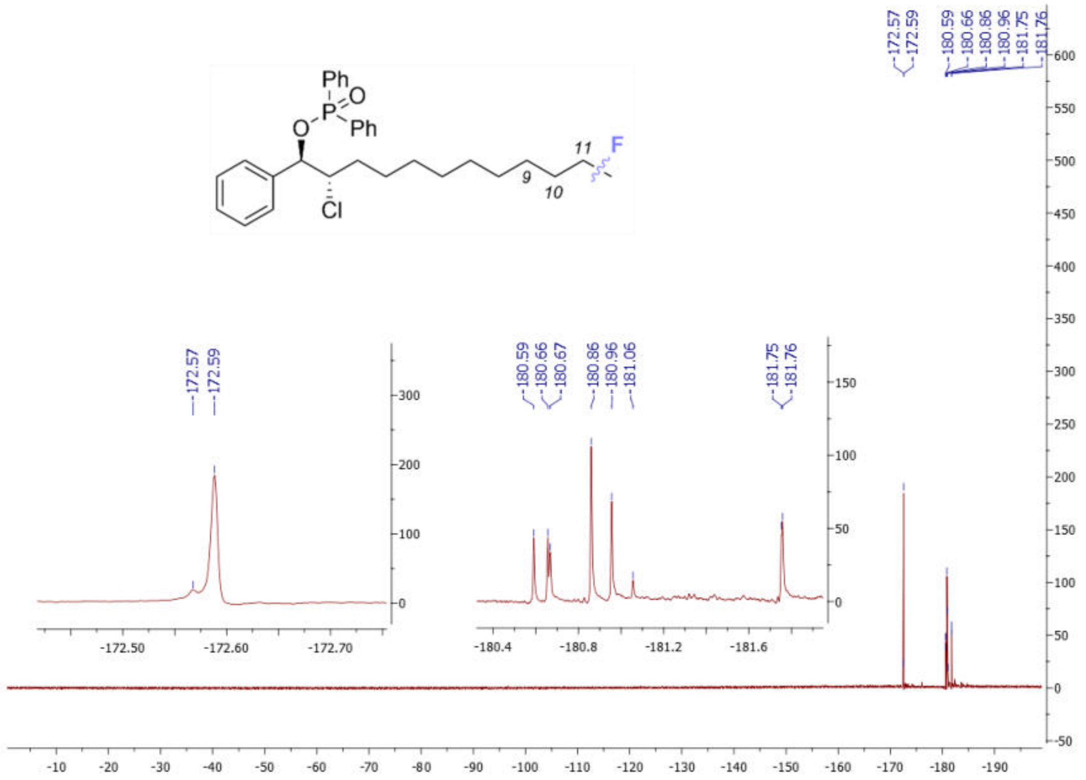

^{31}P NMR of compound **2g** in $CDCl_3$

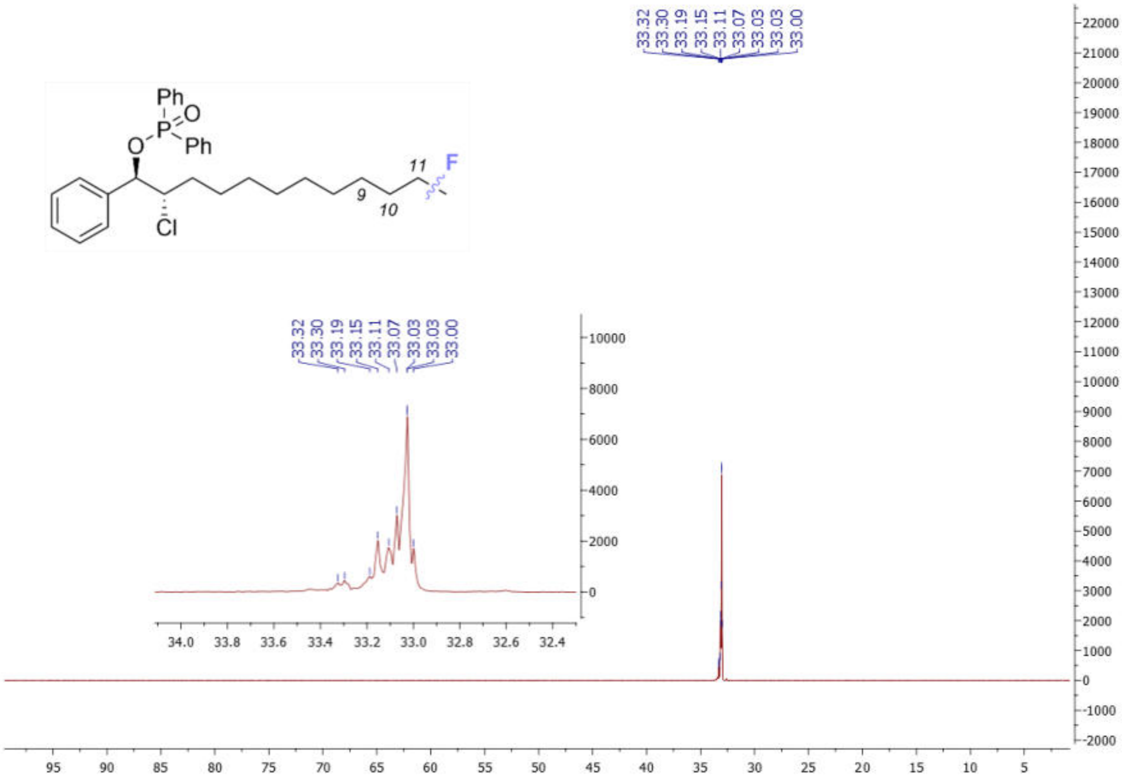

^{1}H NMR of compound **2o** in $CDCl_3$

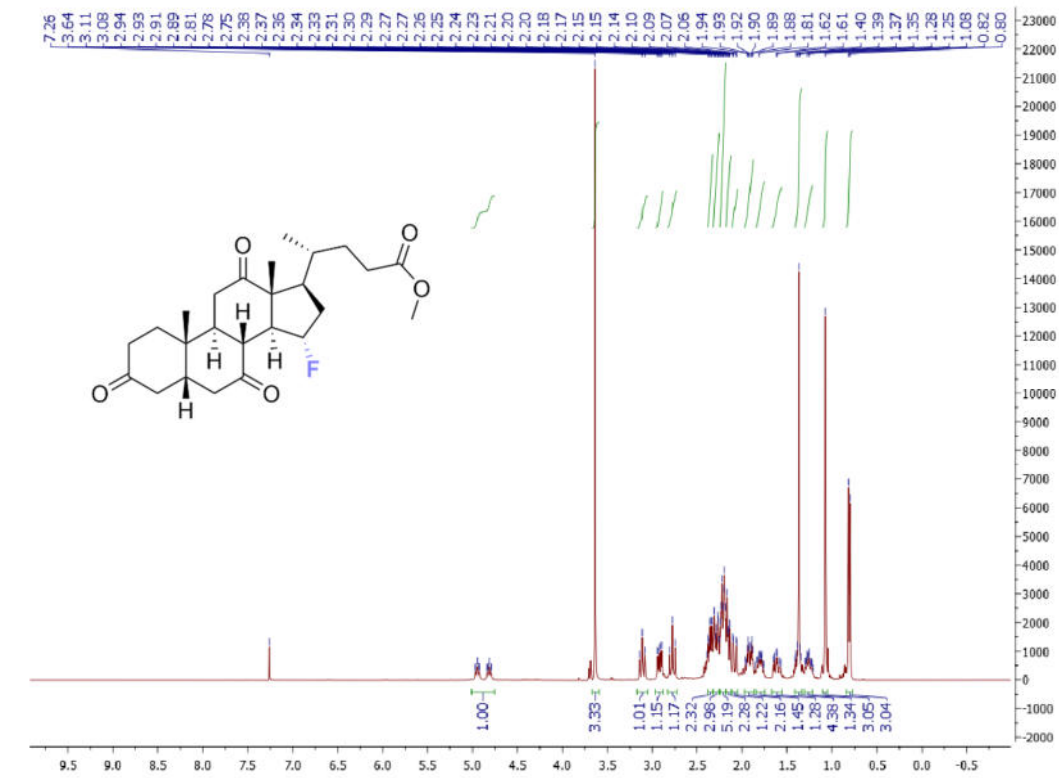

^{13}C NMR of compound **2o** in $CDCl_3$

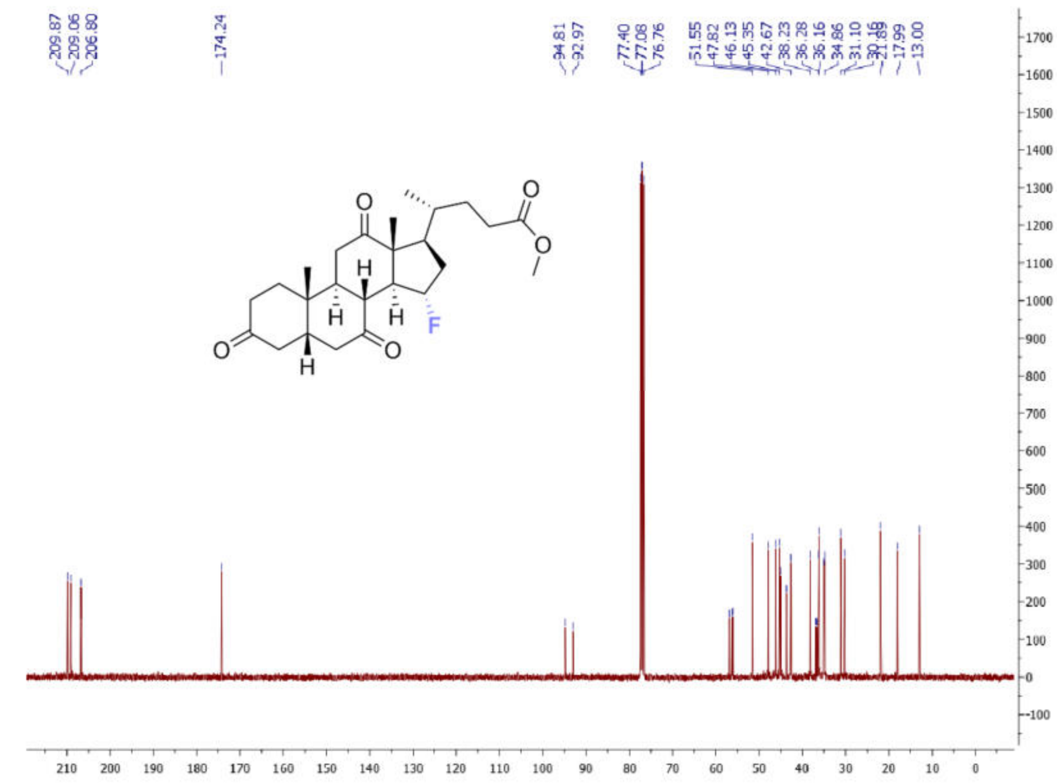

^{19}F NMR of compound **2o** in $CDCl_3$

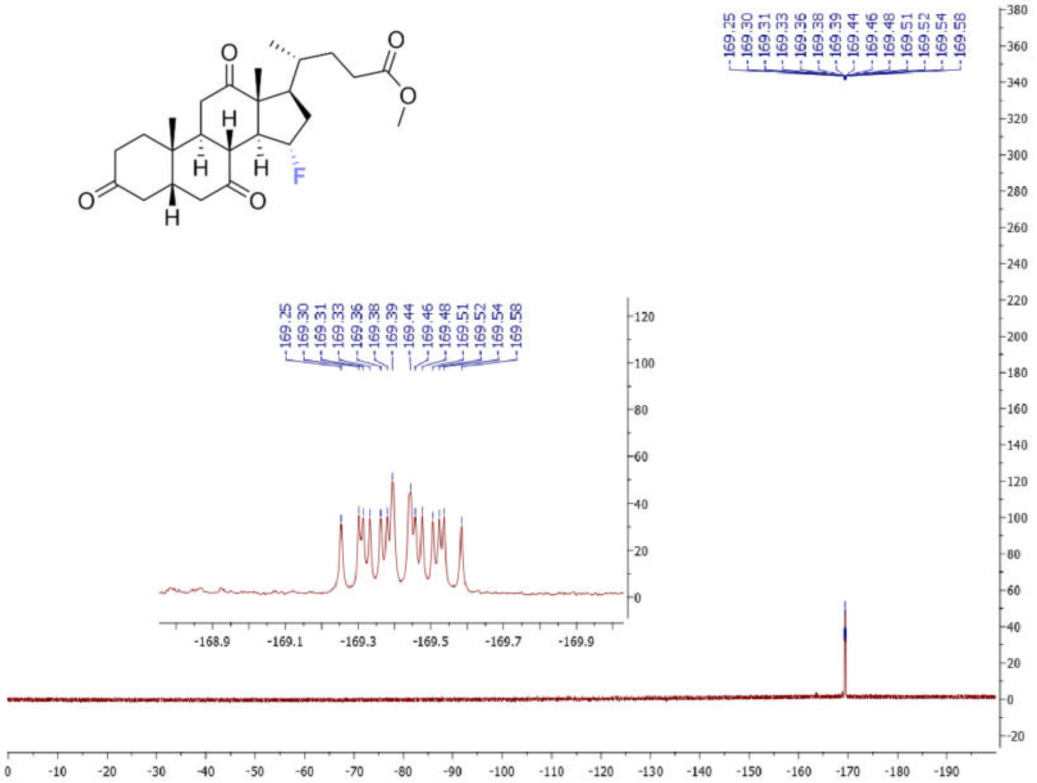

$^{19}F\{^1H\}$ NMR of compound **2o** in $CDCl_3$

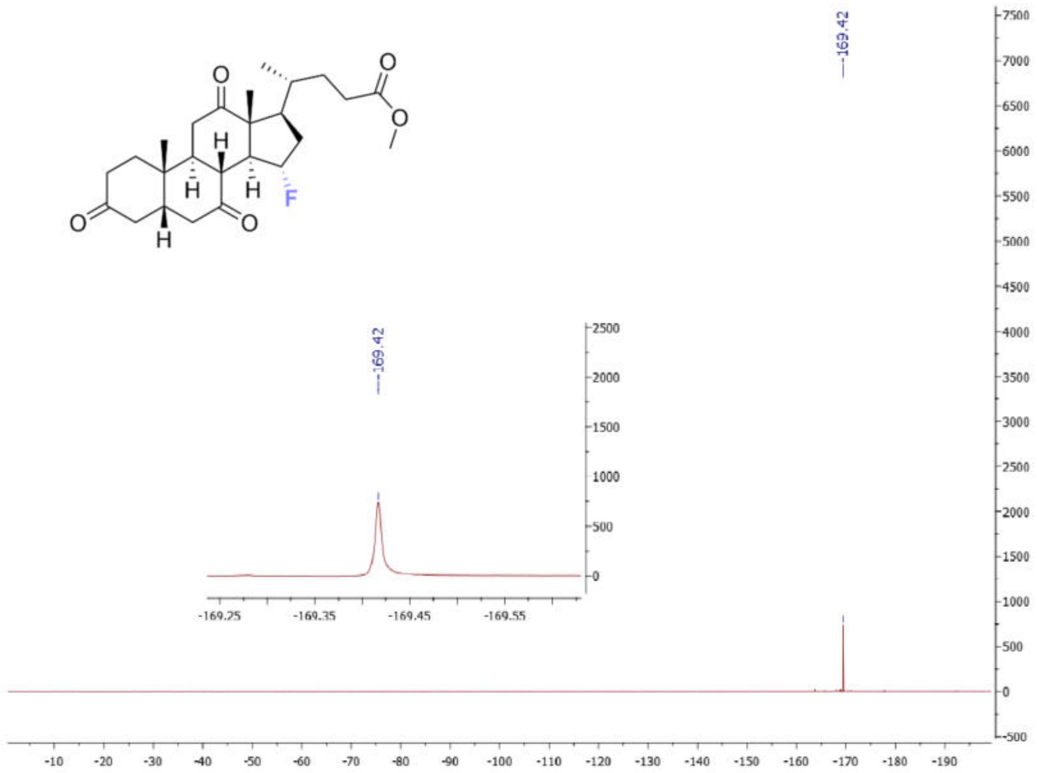

^{1}H NMR of compound **2p** in $CDCl_3$

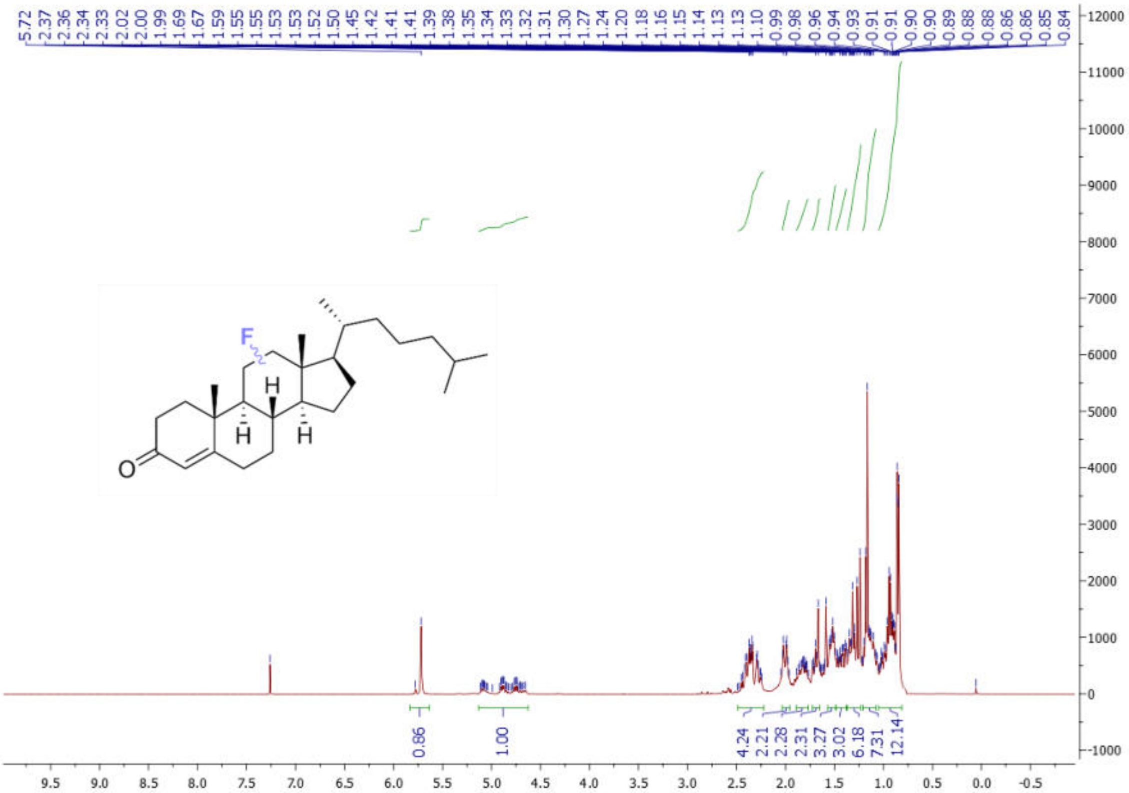

^{13}C NMR of compound **2p** in $CDCl_3$

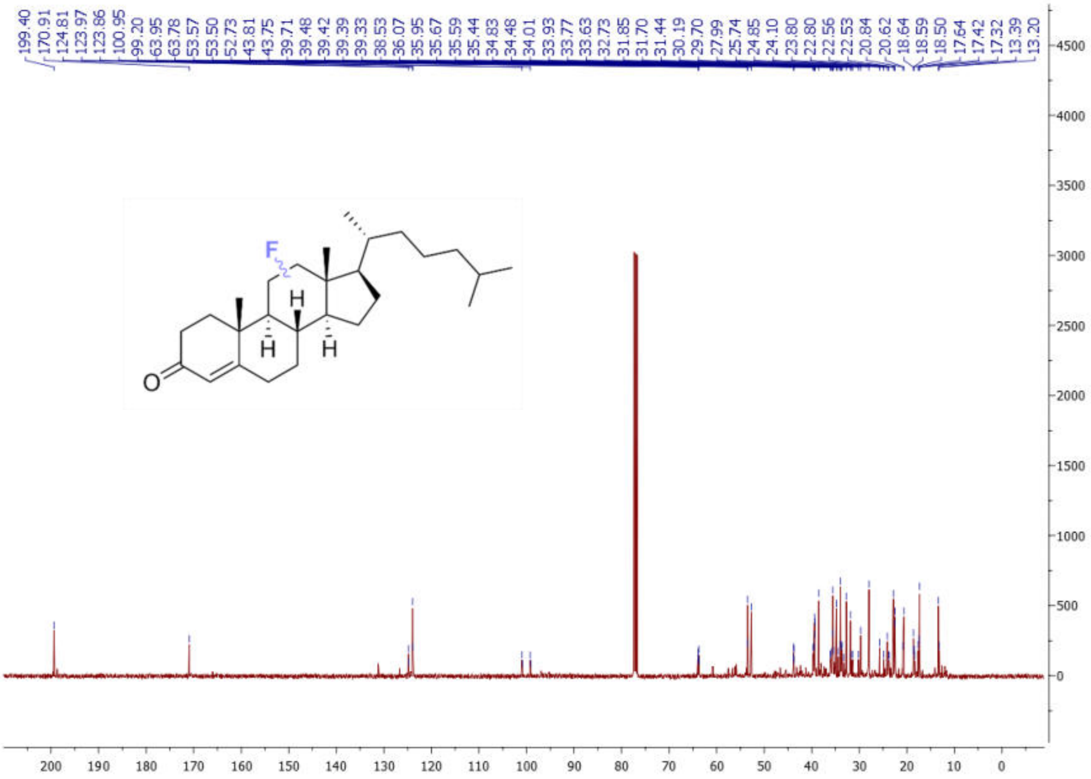

^{19}F NMR of compound **2p** in $CDCl_3$

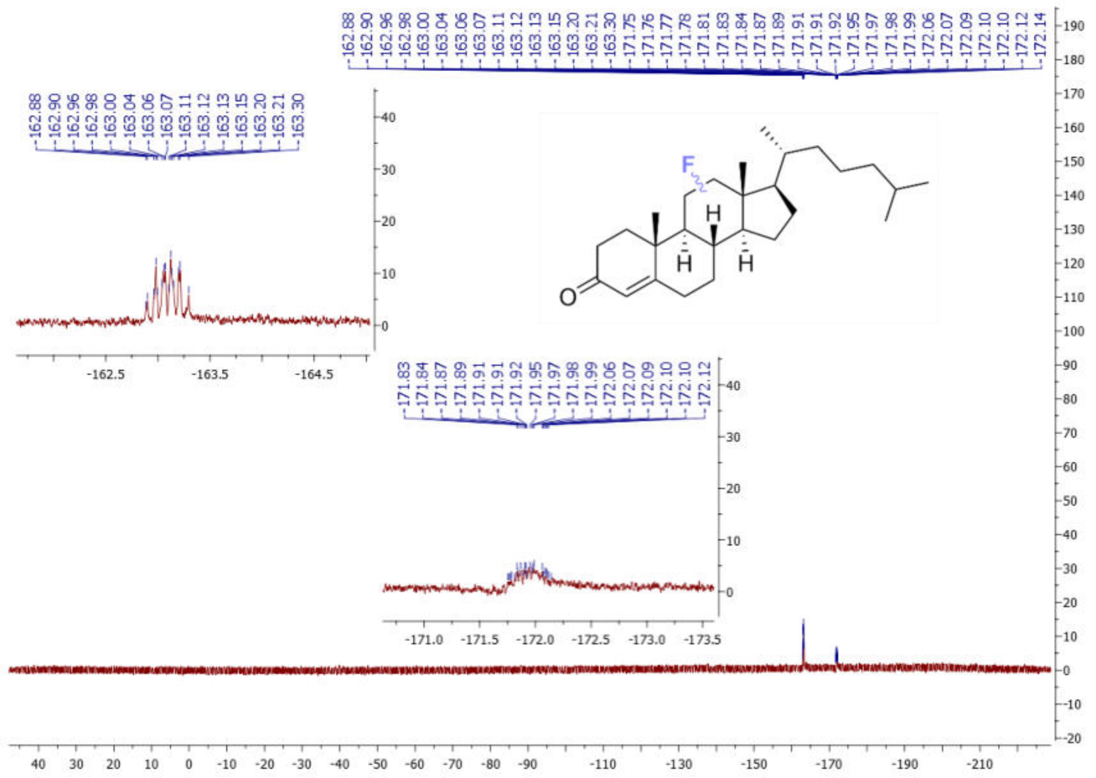

COSY NMR of compound **2p** in $CDCl_3$

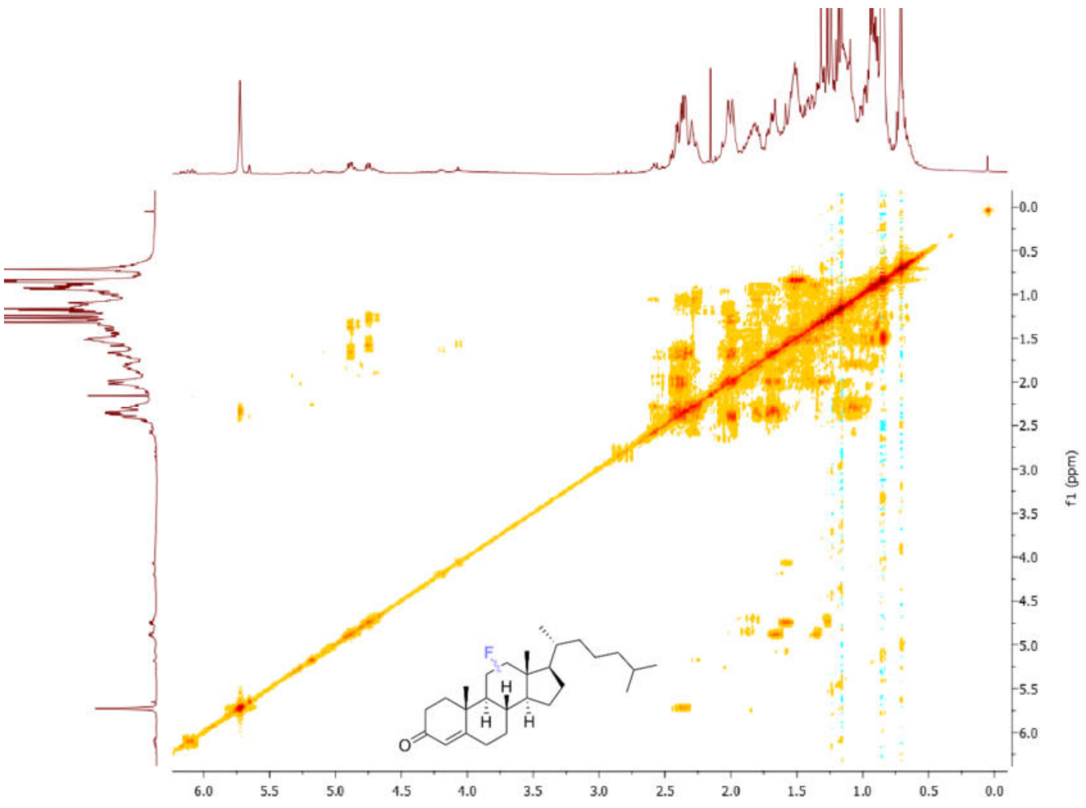

TOCSY NMR of compound **2p** in $CDCl_3$

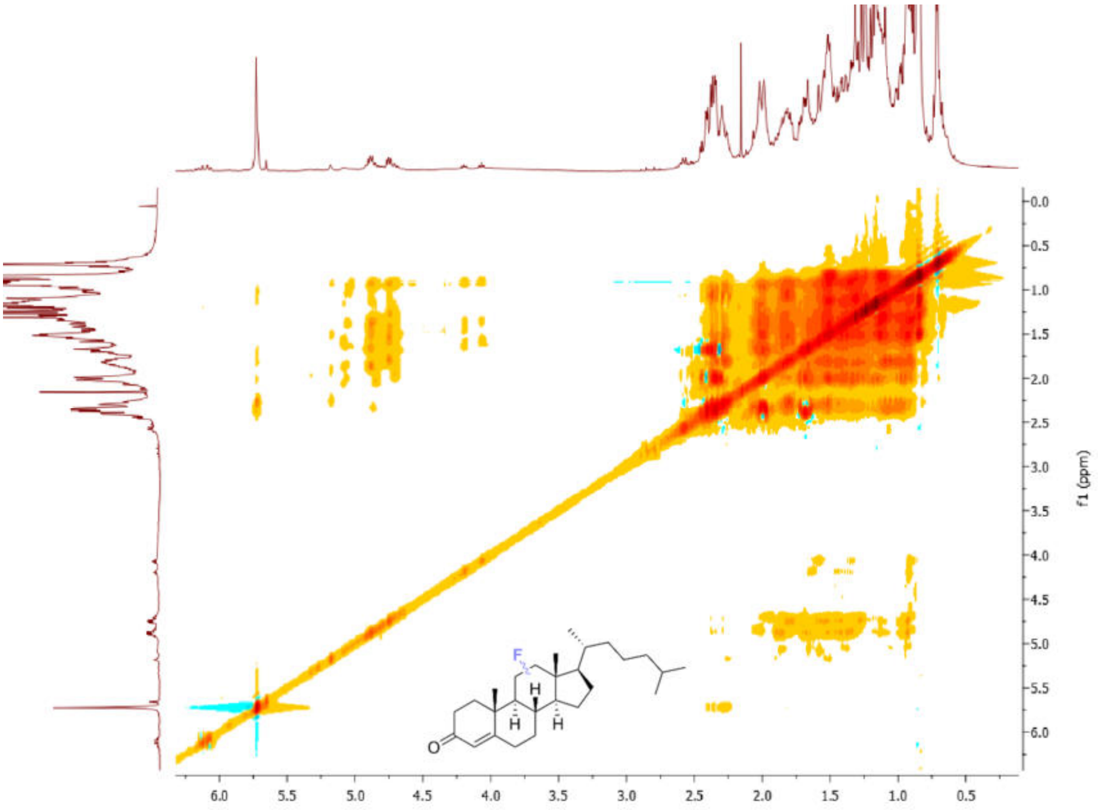

HSQC NMR of compound **2p** in $CDCl_3$

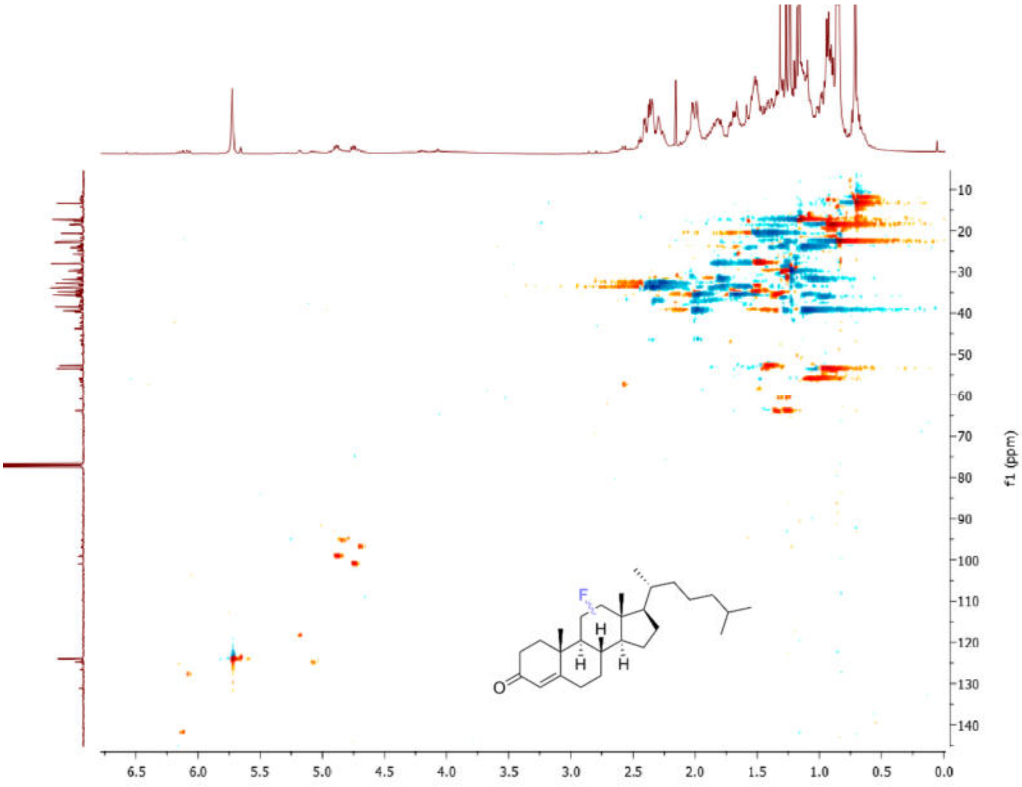

HMBC NMR of compound **2p** in $CDCl_3$

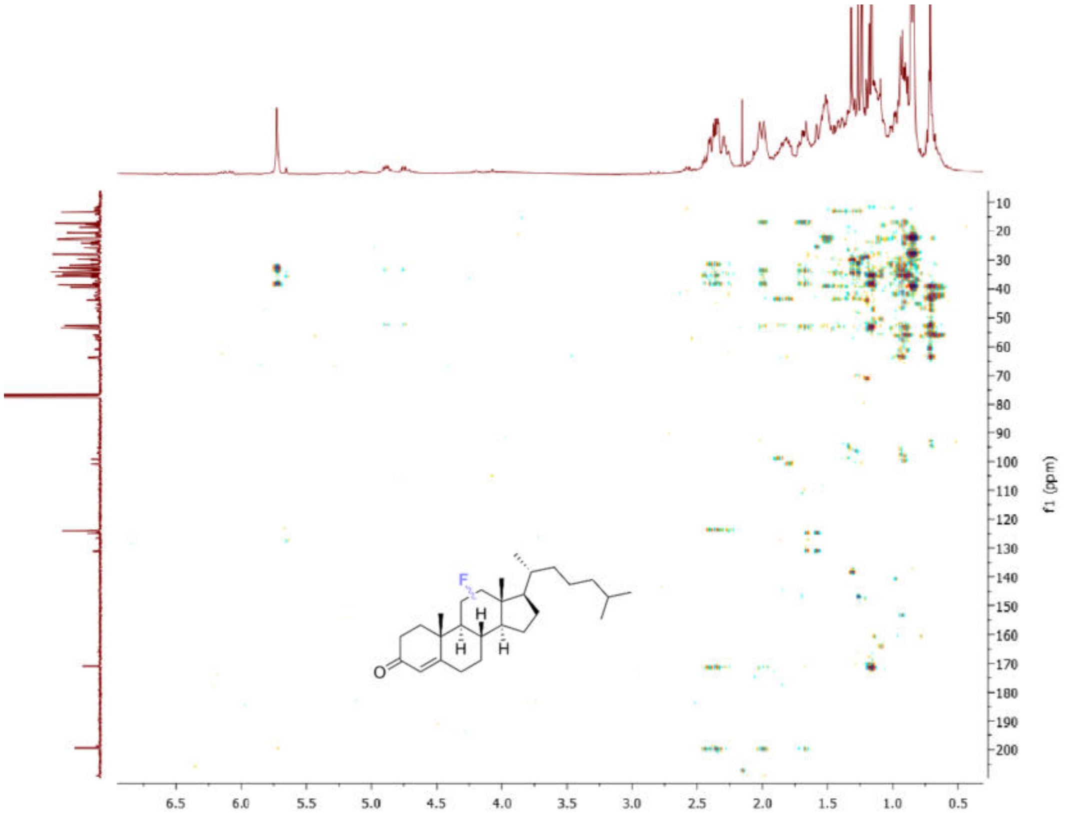

^{1}H NMR of compound **2y** in $CDCl_3$

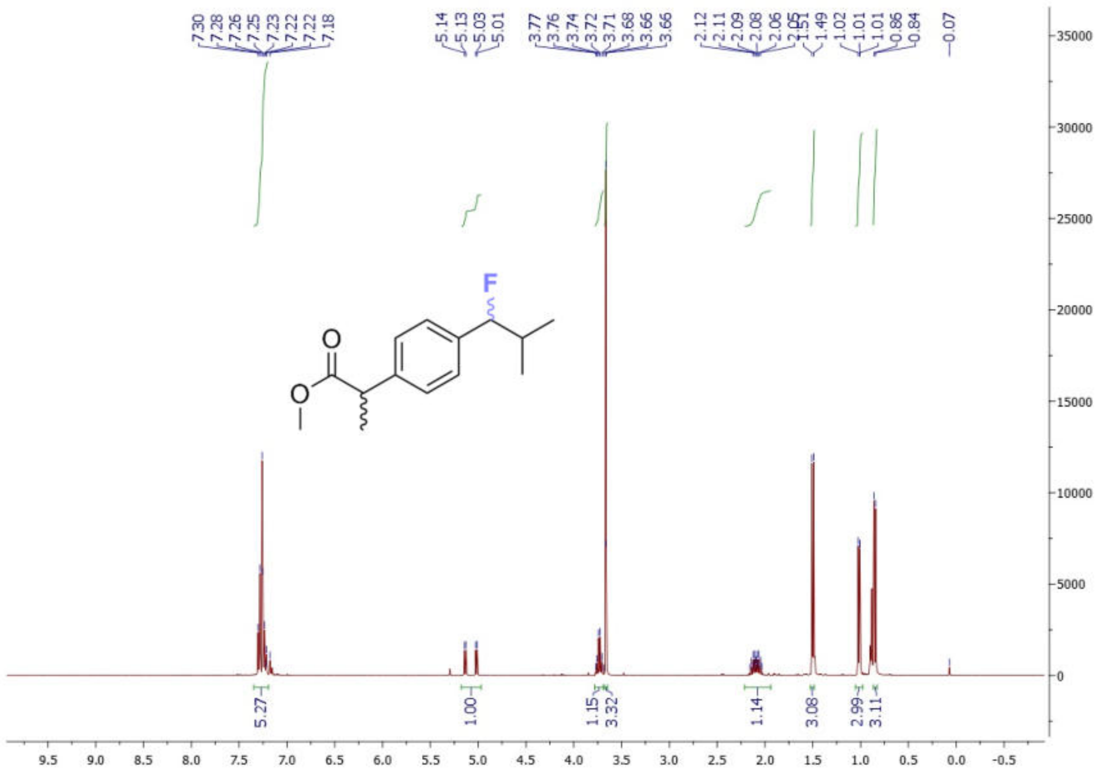

^{13}C NMR of compound **2y** in $CDCl_3$

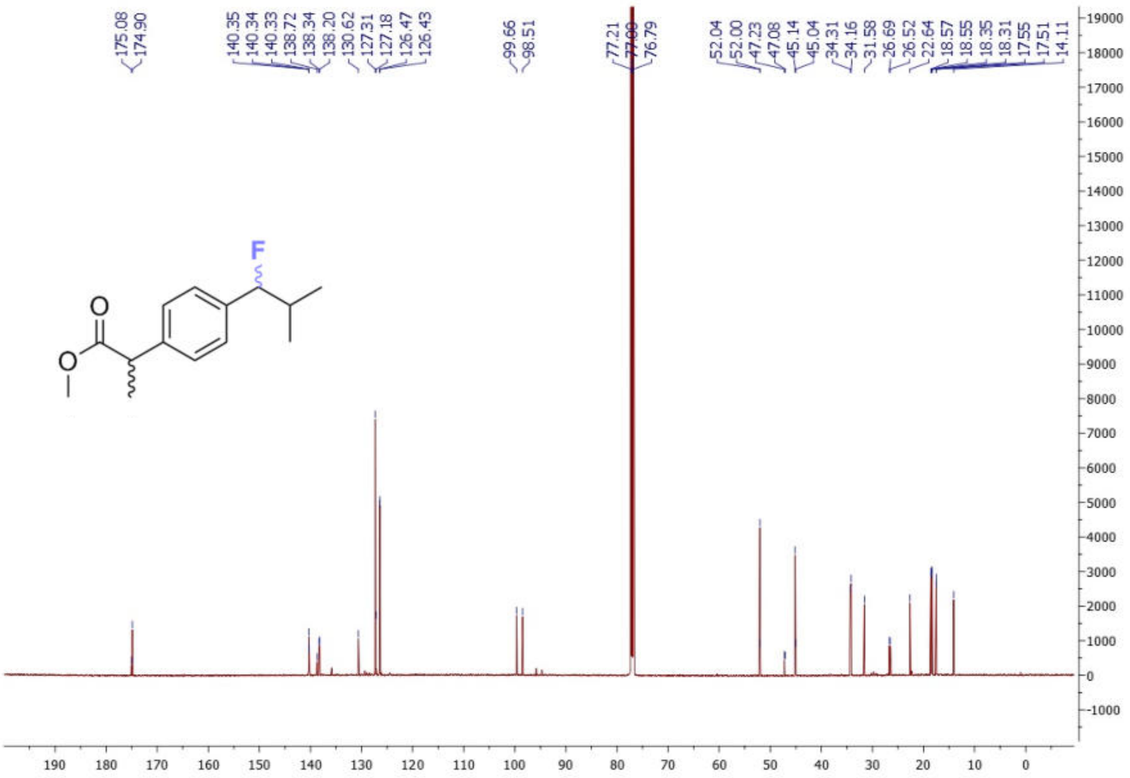

^{19}F NMR of compound **2y** in $CDCl_3$

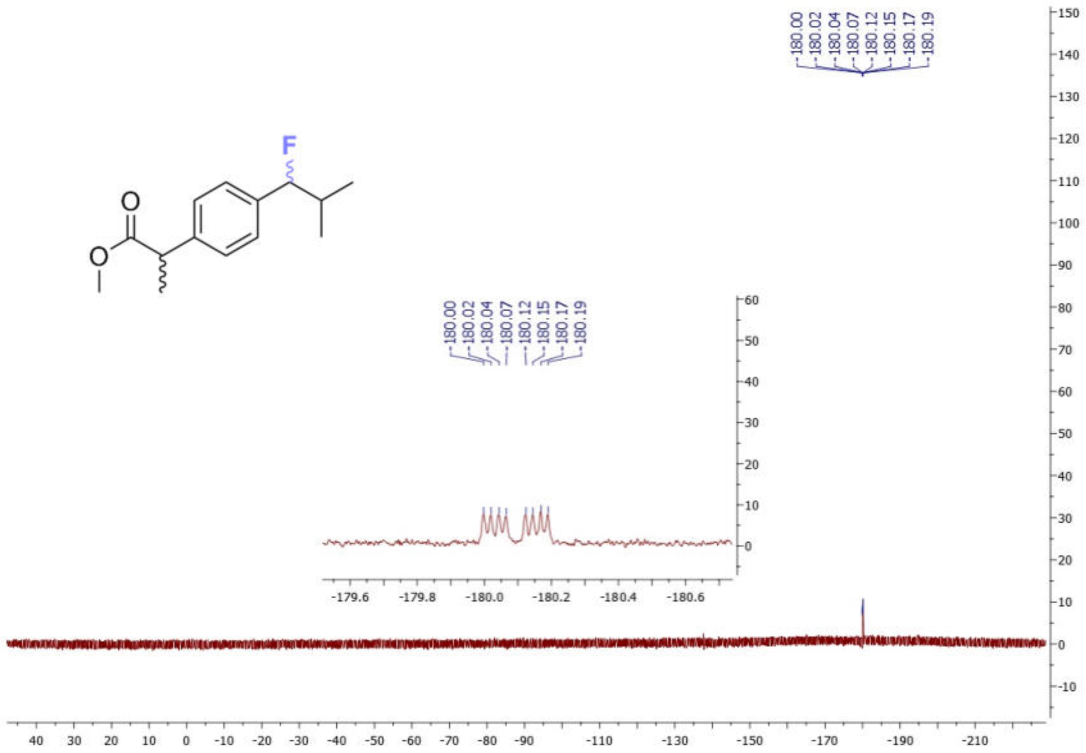

^{1}H NMR of compound **20** in $CDCl_3$

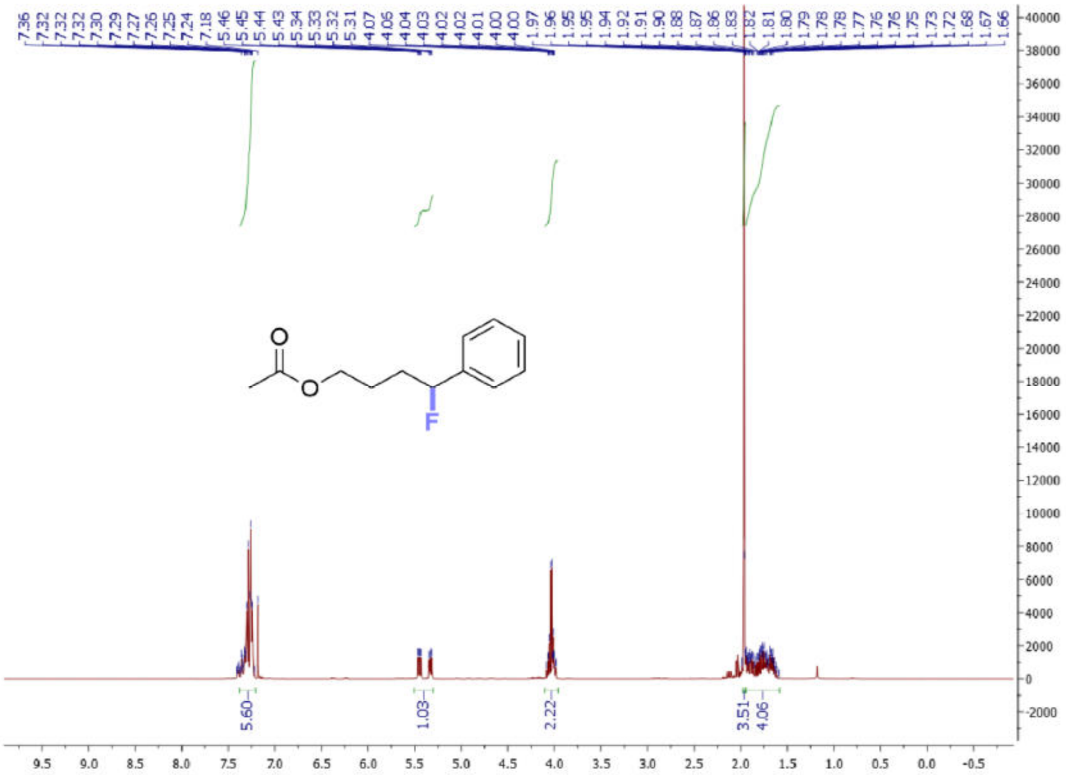

^{13}C NMR of compound **20** in $CDCl_3$

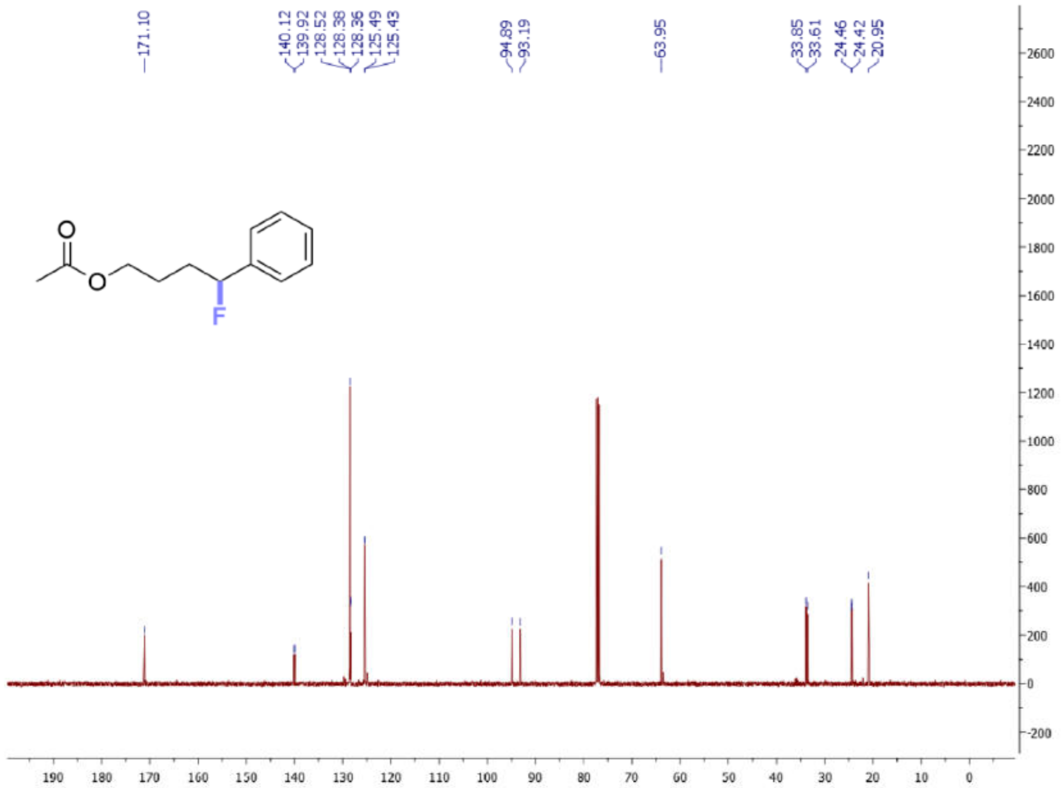

^{19}F NMR of compound **20** in $CDCl_3$

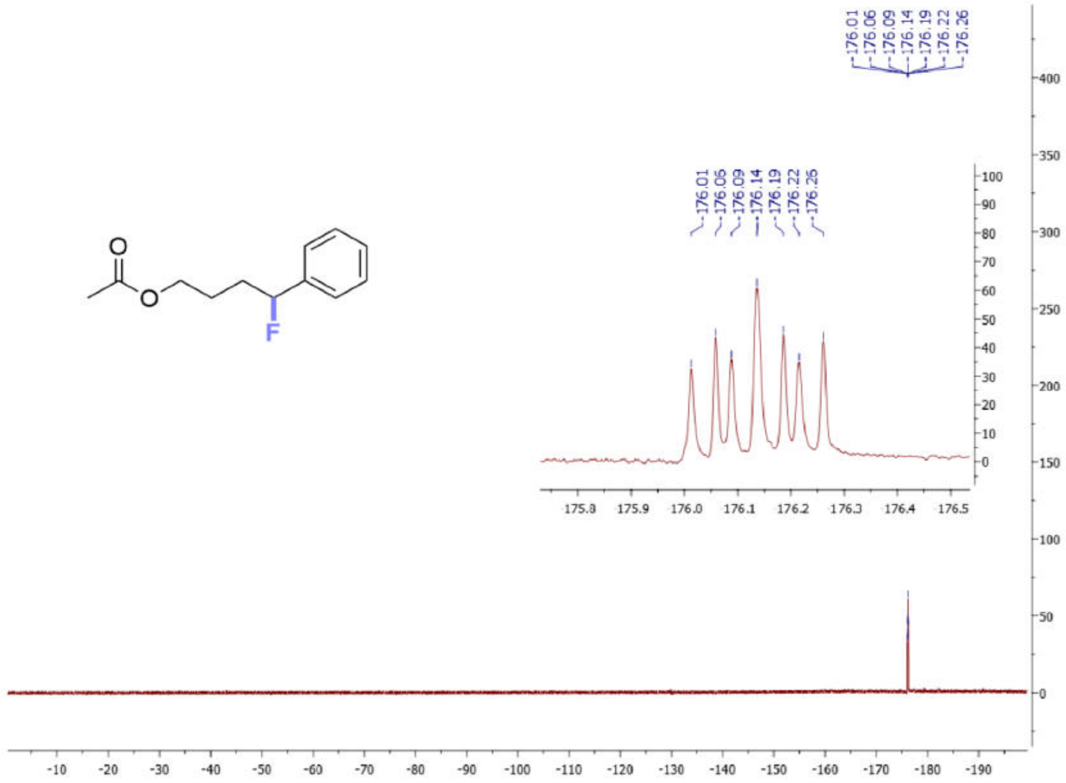

^{1}H NMR of compound **2aa** in $CDCl_3$

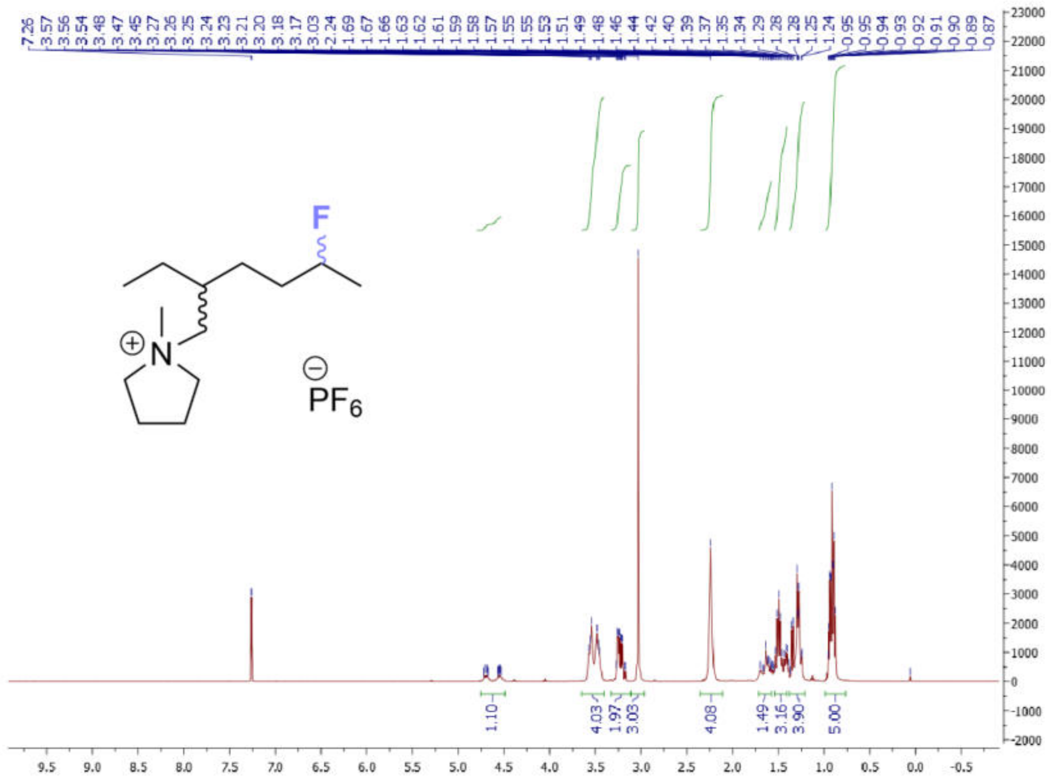

[13] C NMR of compound **2aa** in $CDCl_3$

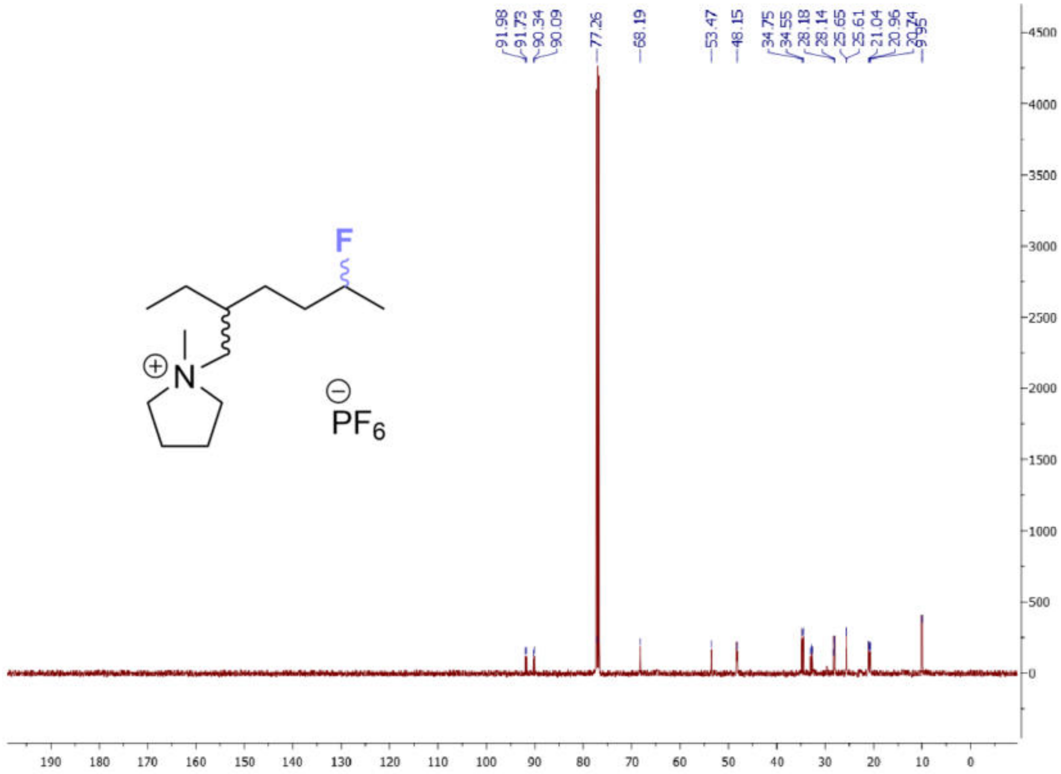

[19] F NMR of compound **2aa** in $CDCl_3$

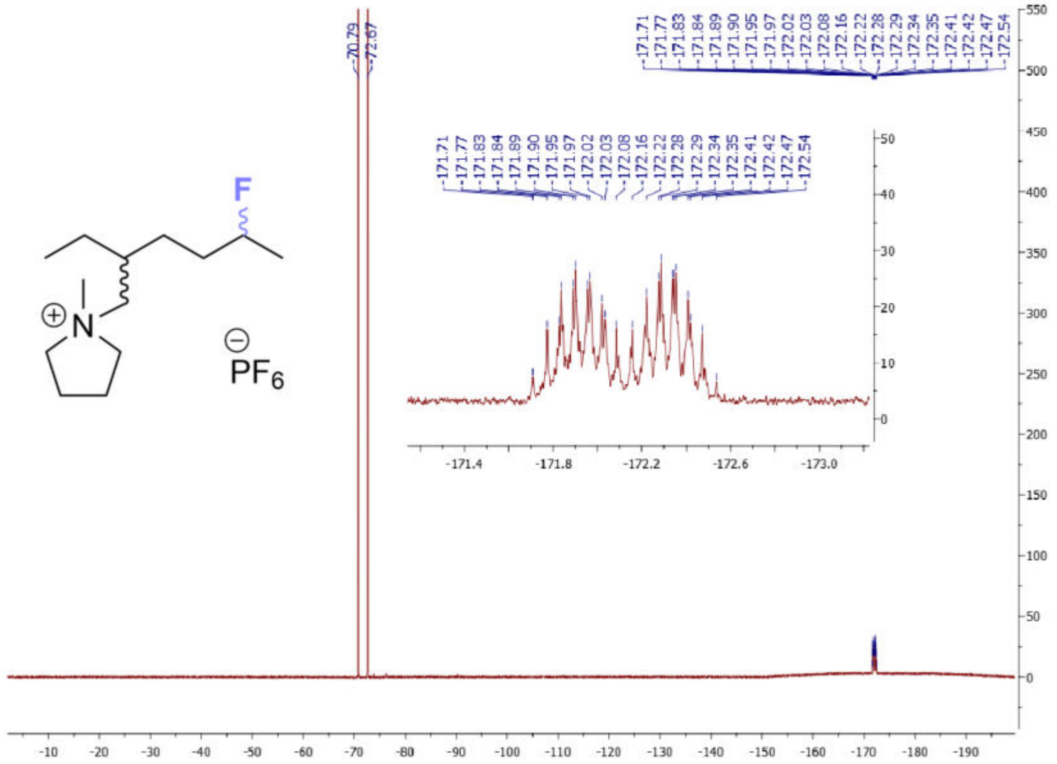

^{1}H NMR of compound **2ae** in $CDCl_3$

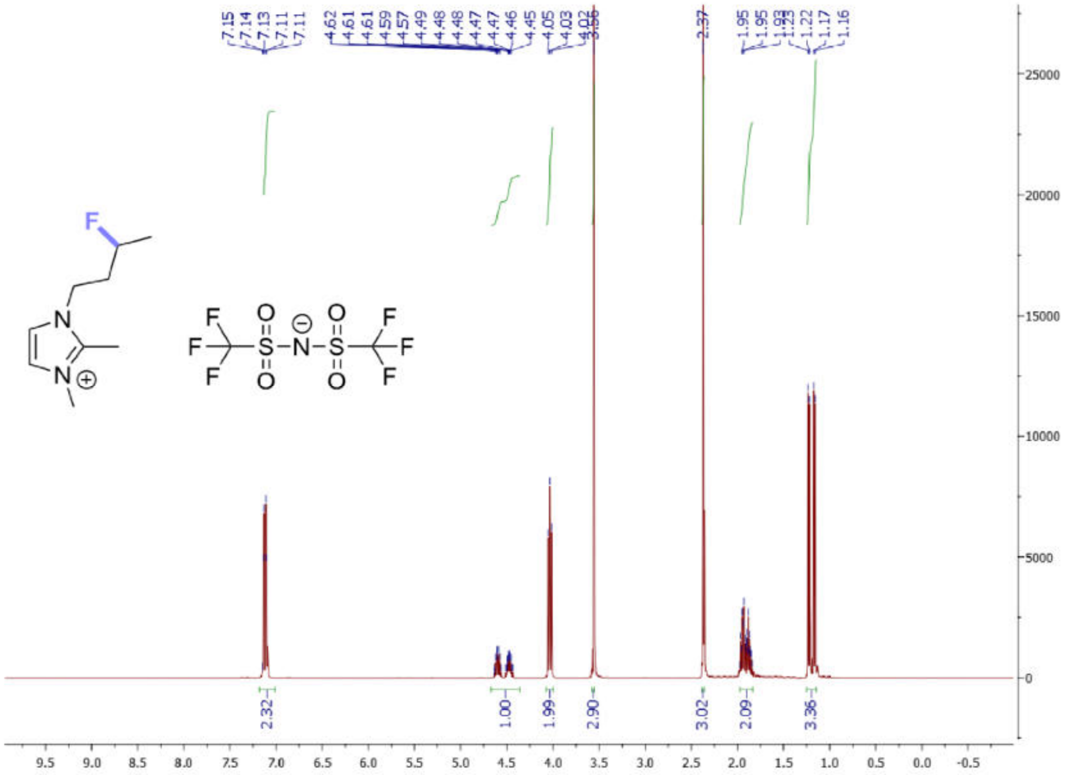

^{13}C NMR of compound **2ae** in $CDCl_3$

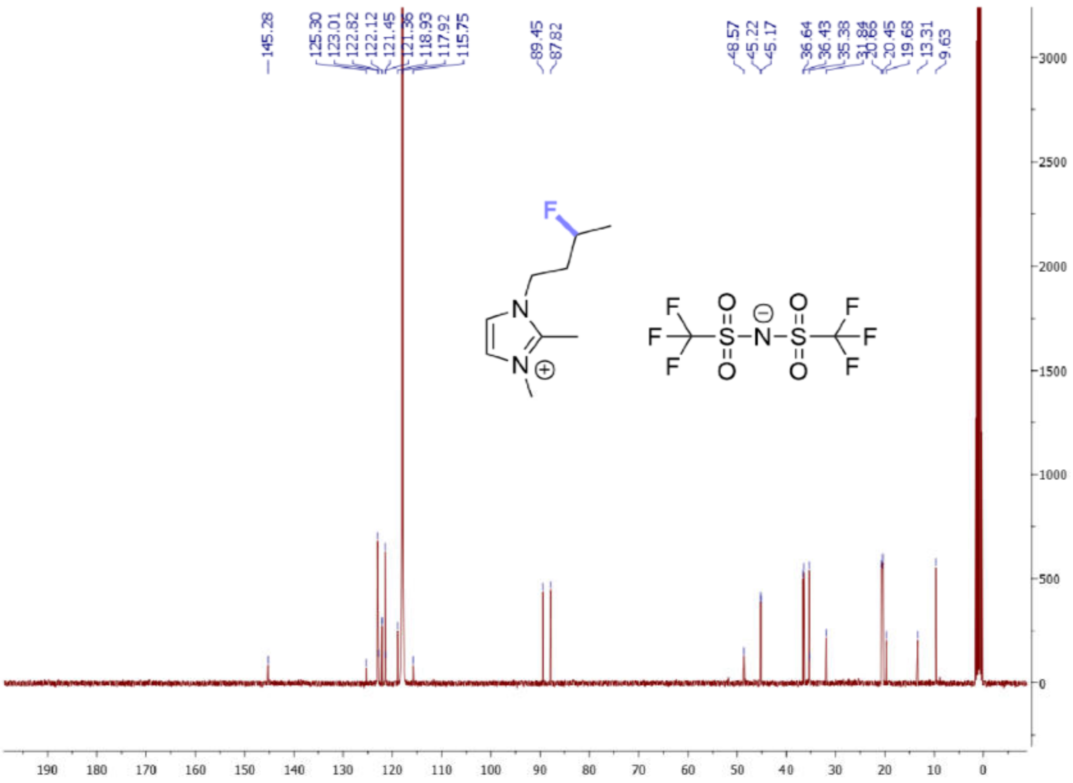

^{19}F NMR of compound **2ae** in $CDCl_3$

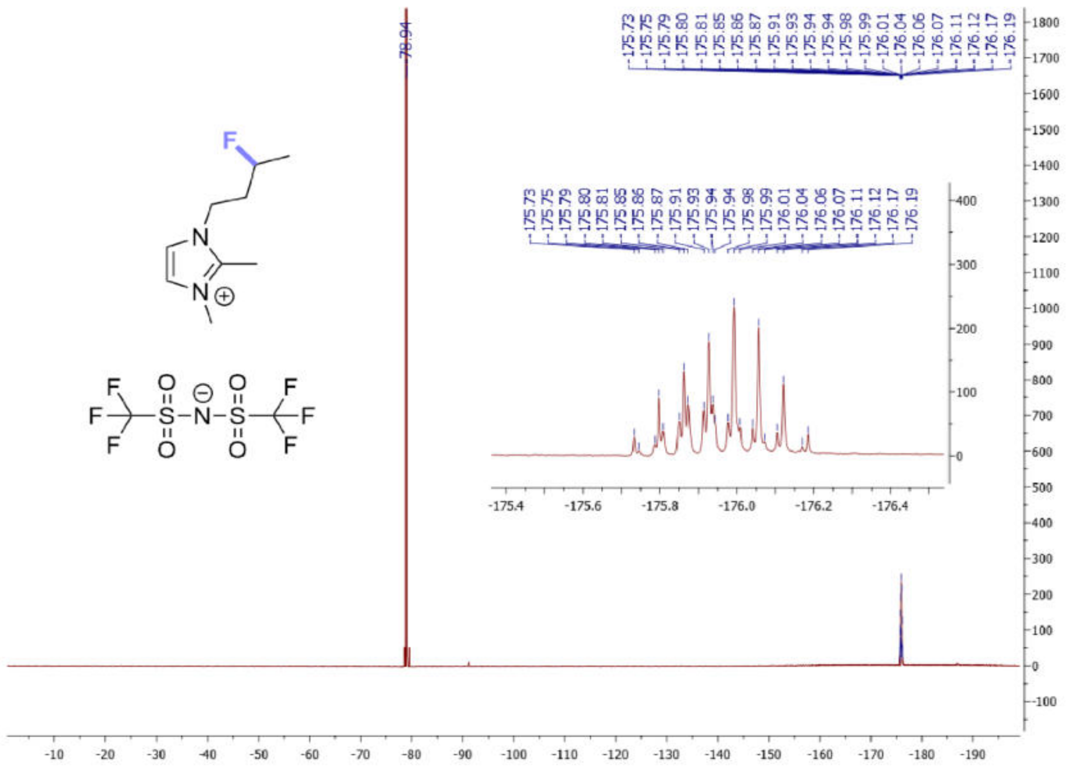

^{1}H NMR of compound **2ad** in $CDCl_3$

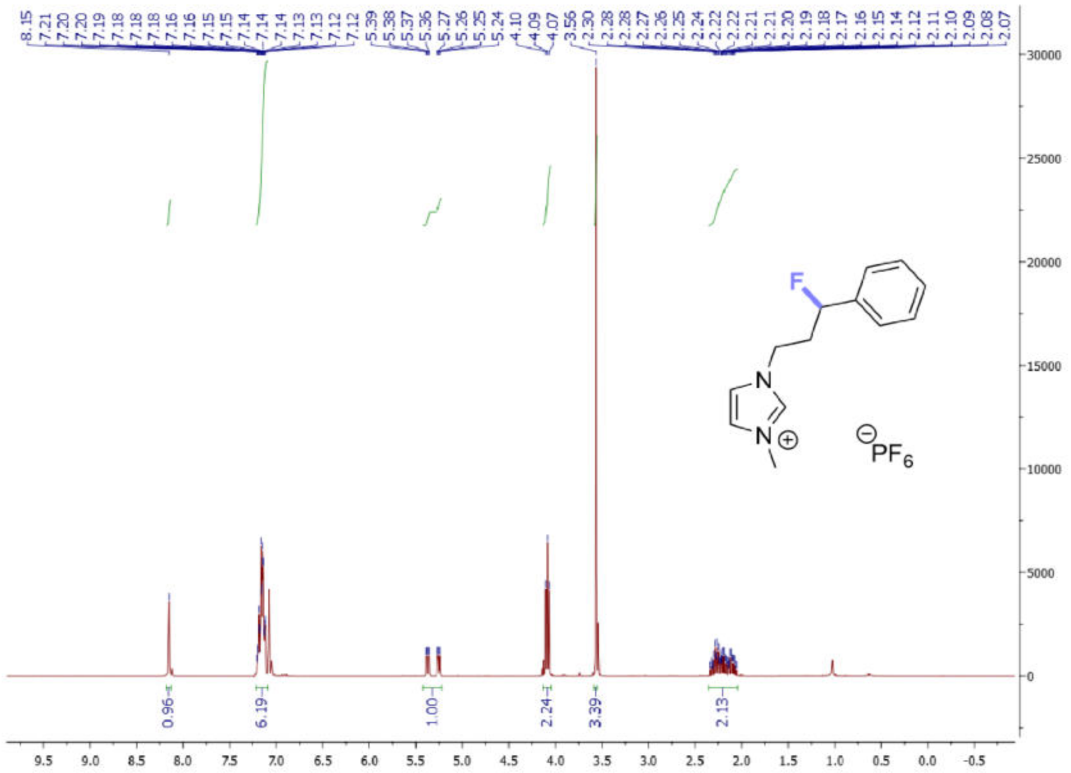

^{13}C NMR of compound **2ad** in $CDCl_3$

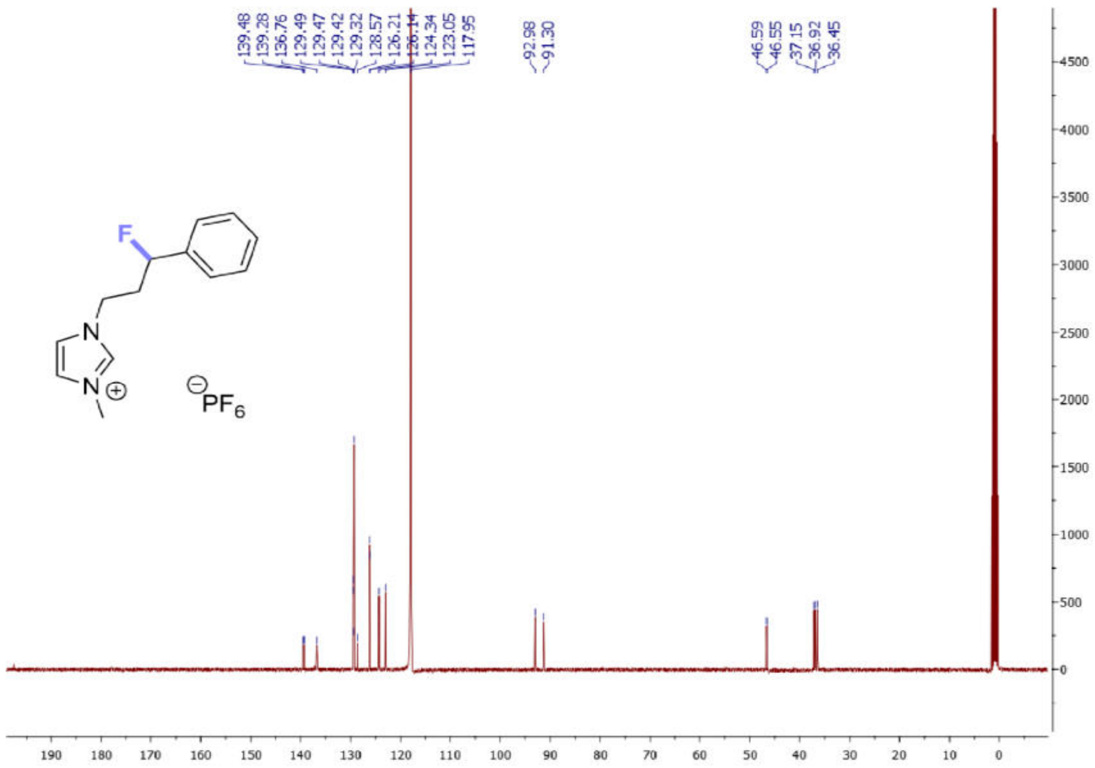

^{19}F NMR of compound **2ad** in $CDCl_3$

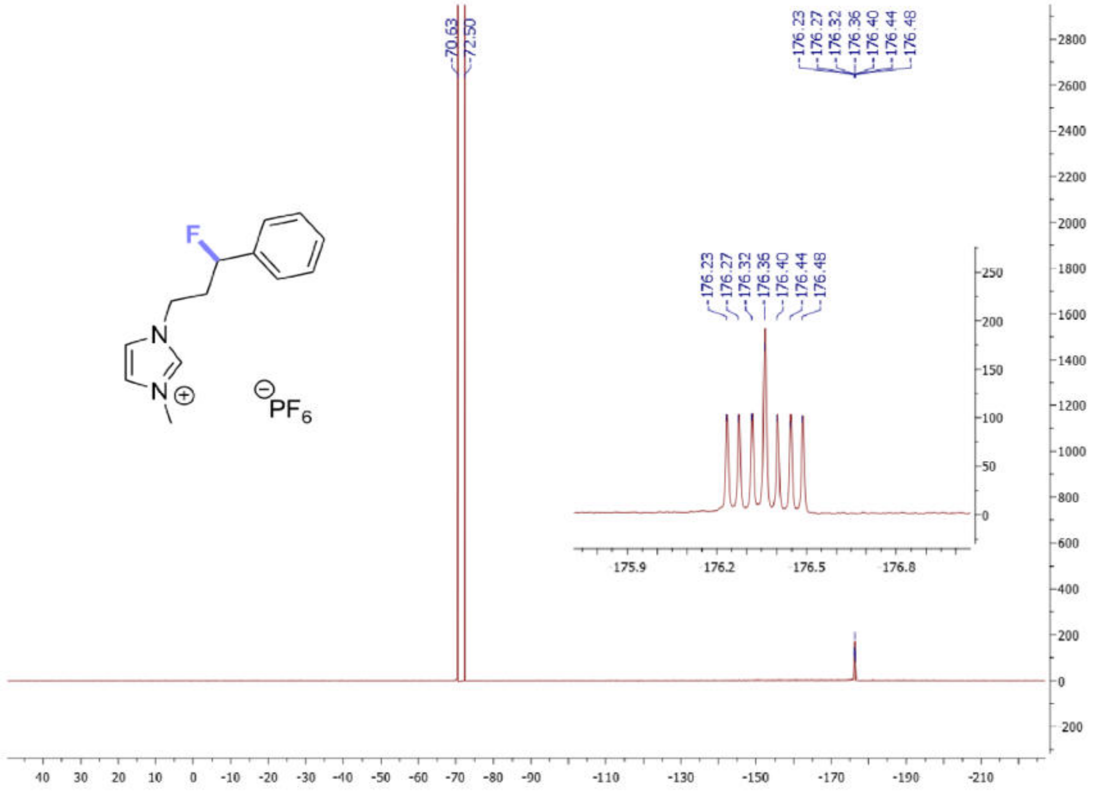

^{1}H NMR of compound **2ac** in $CDCl_3$

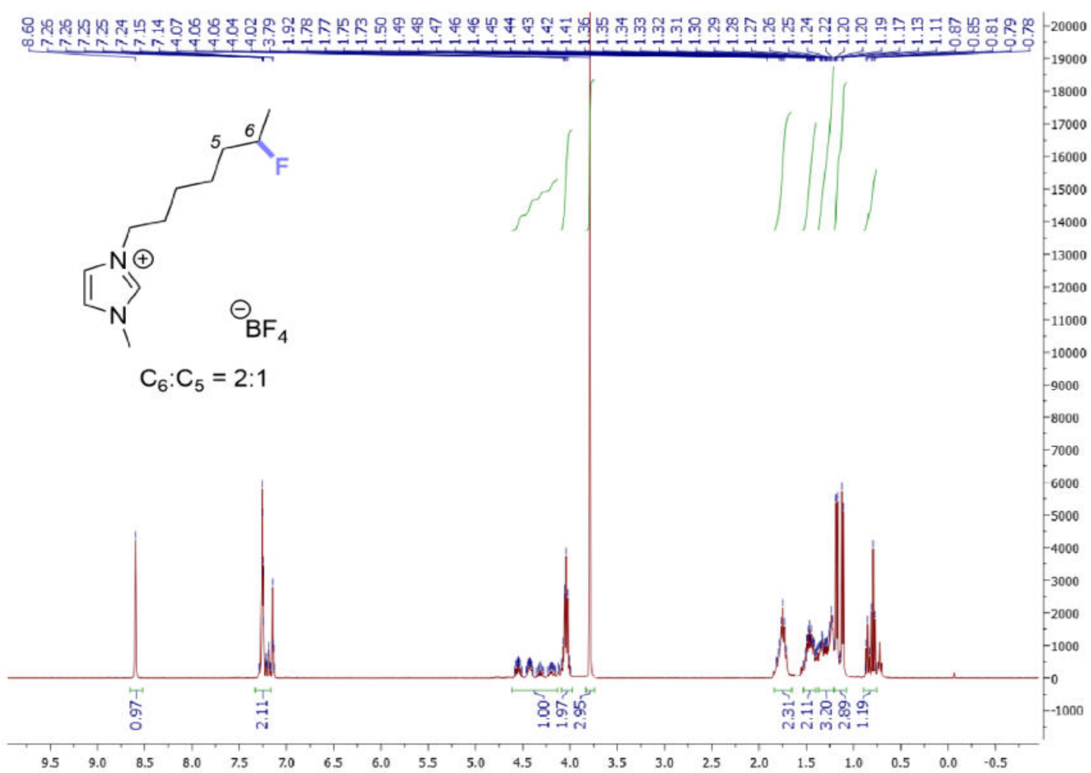

^{13}C NMR of compound **2ac** in $CDCl_3$

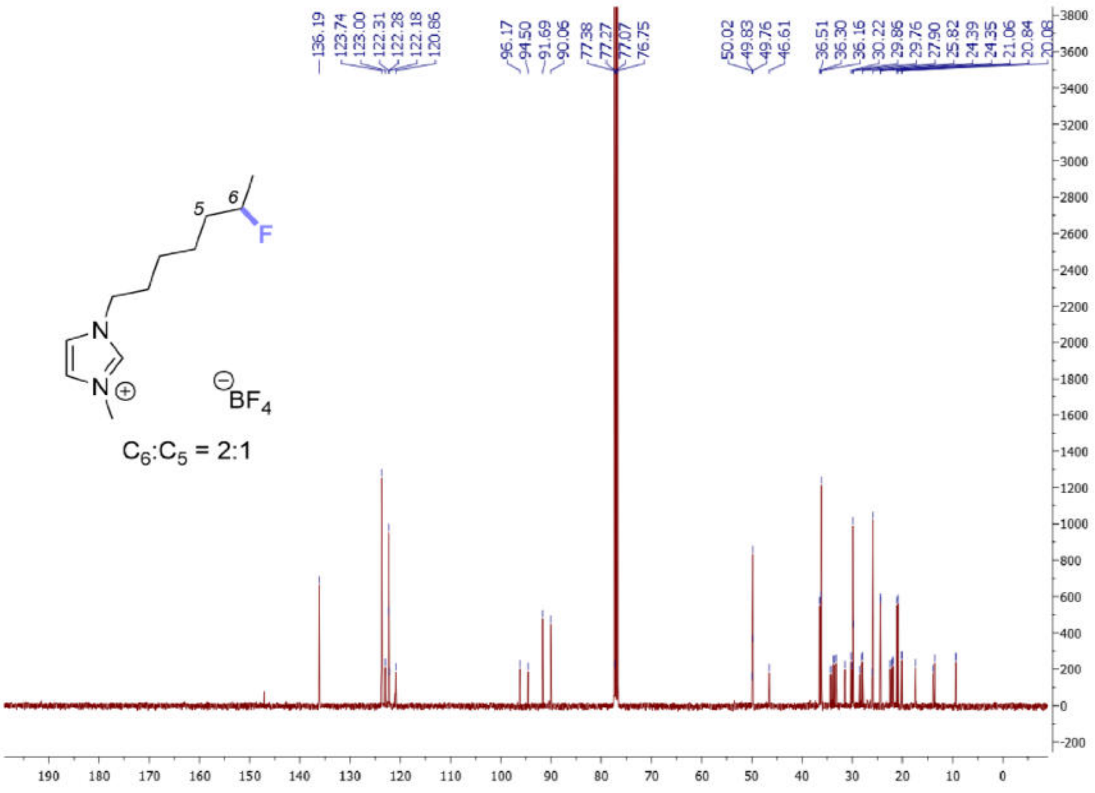

^{19}F NMR of compound **2ac** in $CDCl_3$

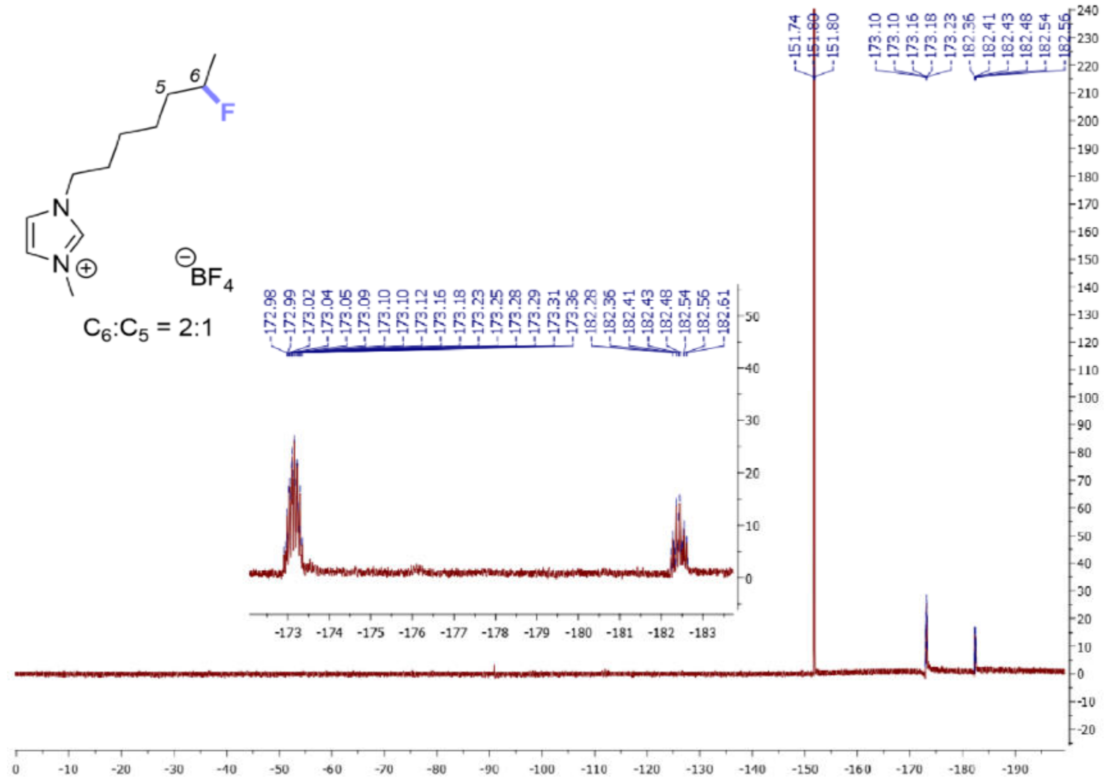

^{1}H NMR of compound **2ab** in $CDCl_3$

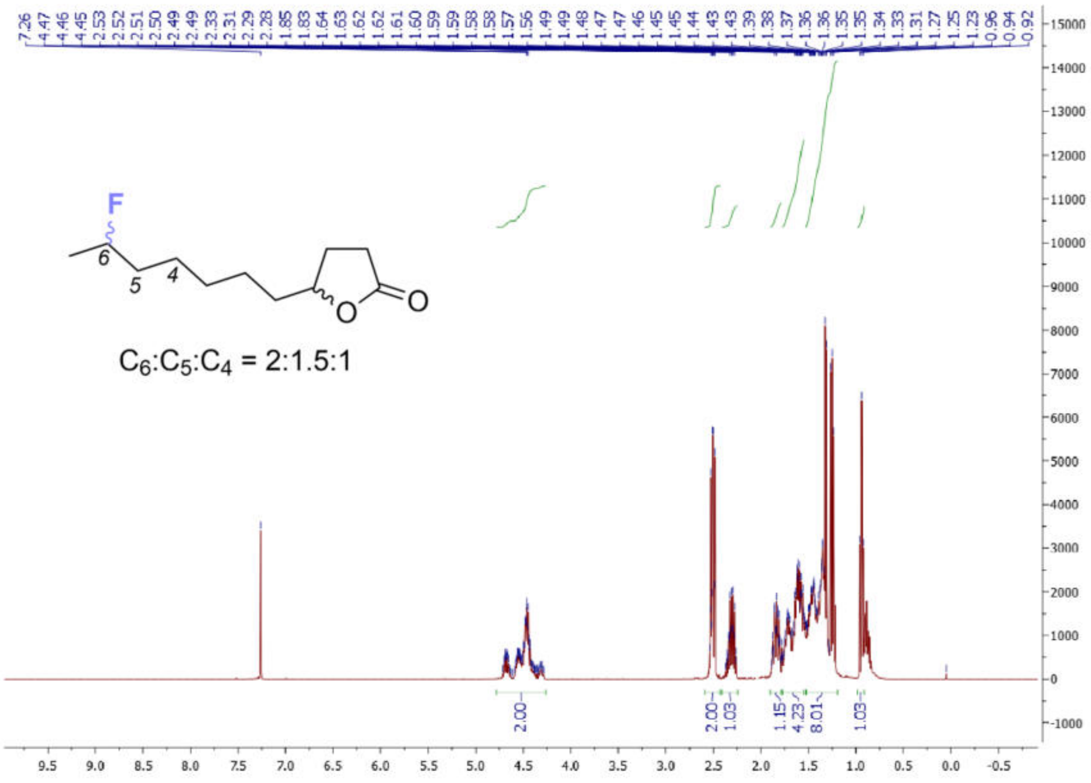

^{13}C NMR of compound **2ab** in $CDCl_3$

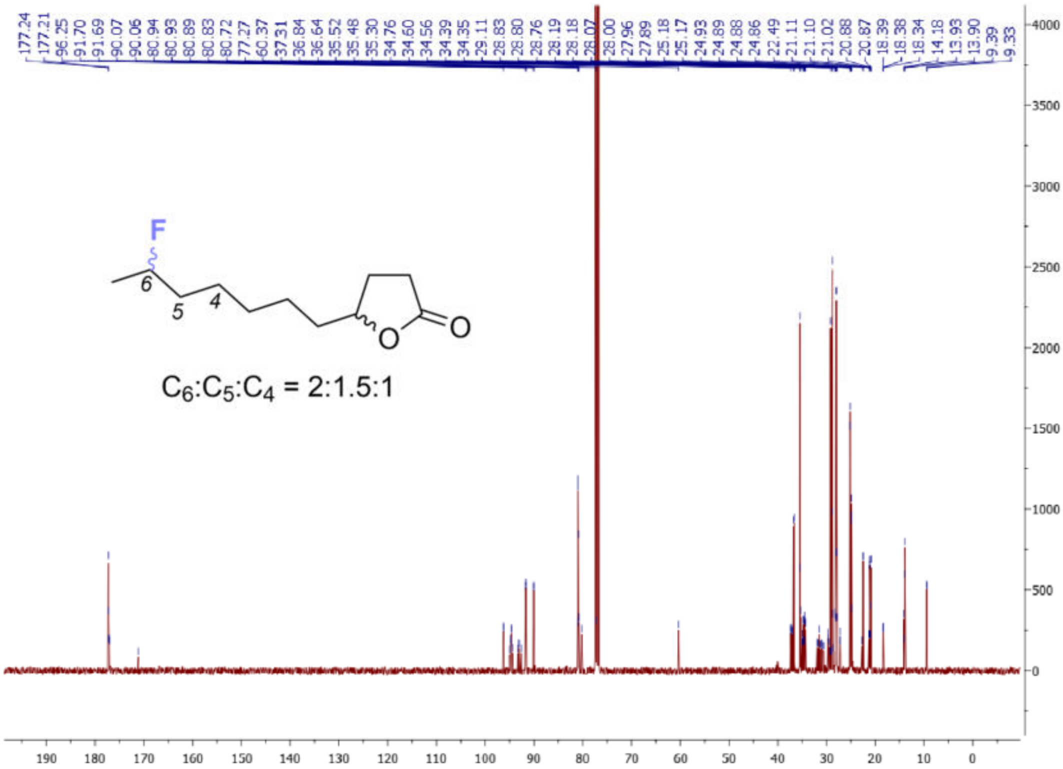

^{19}F NMR of compound **2ab** in $CDCl_3$

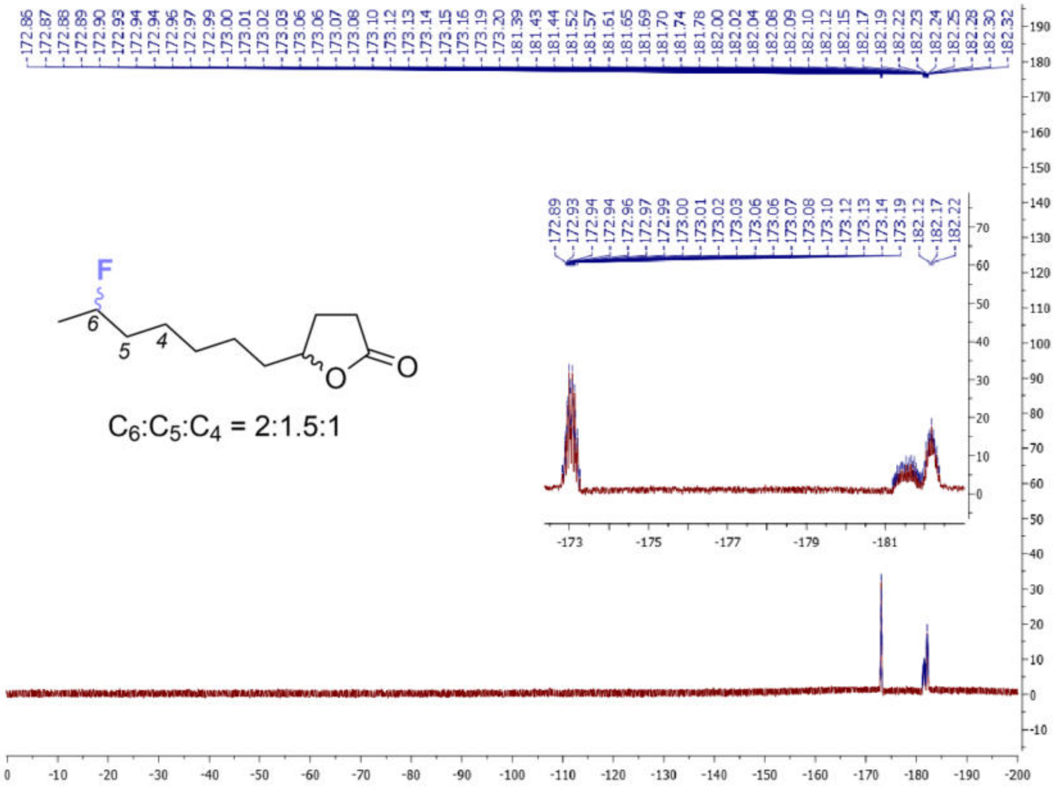

^{1}H NMR of compound **9i** in $CDCl_3$

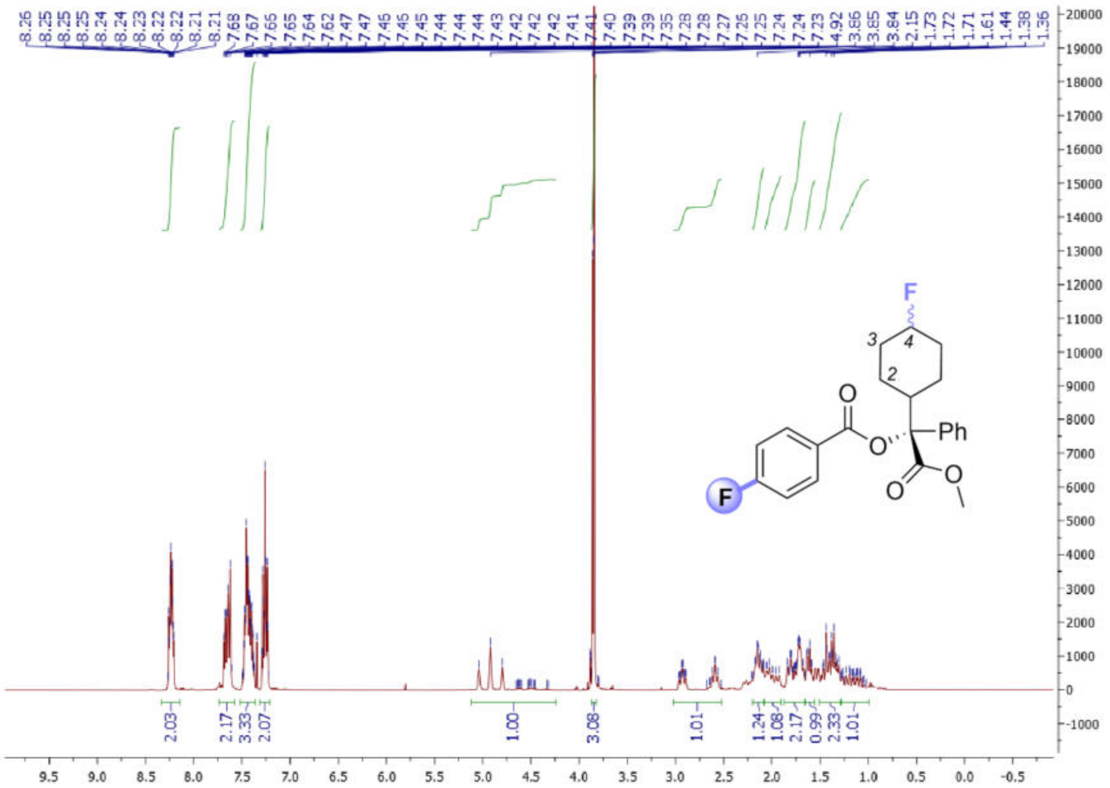

^{13}C NMR of compound **9i** in $CDCl_3$

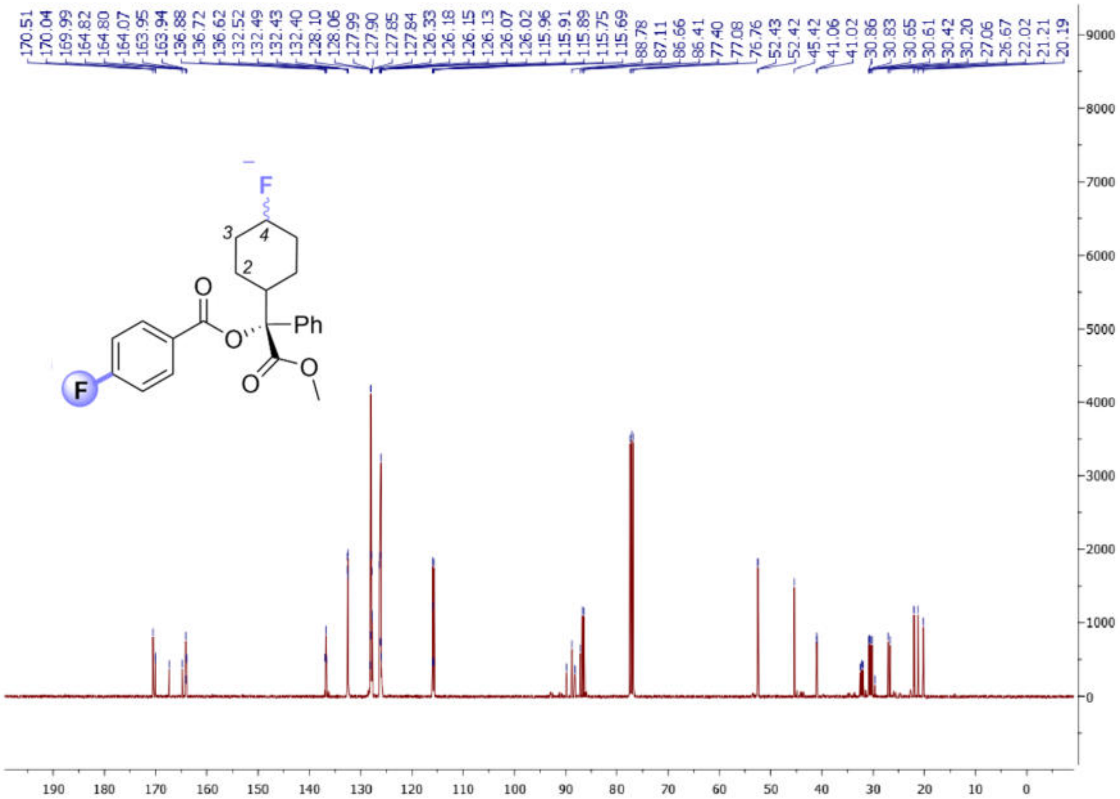

^{19}F NMR of compound **9i** in $CDCl_3$

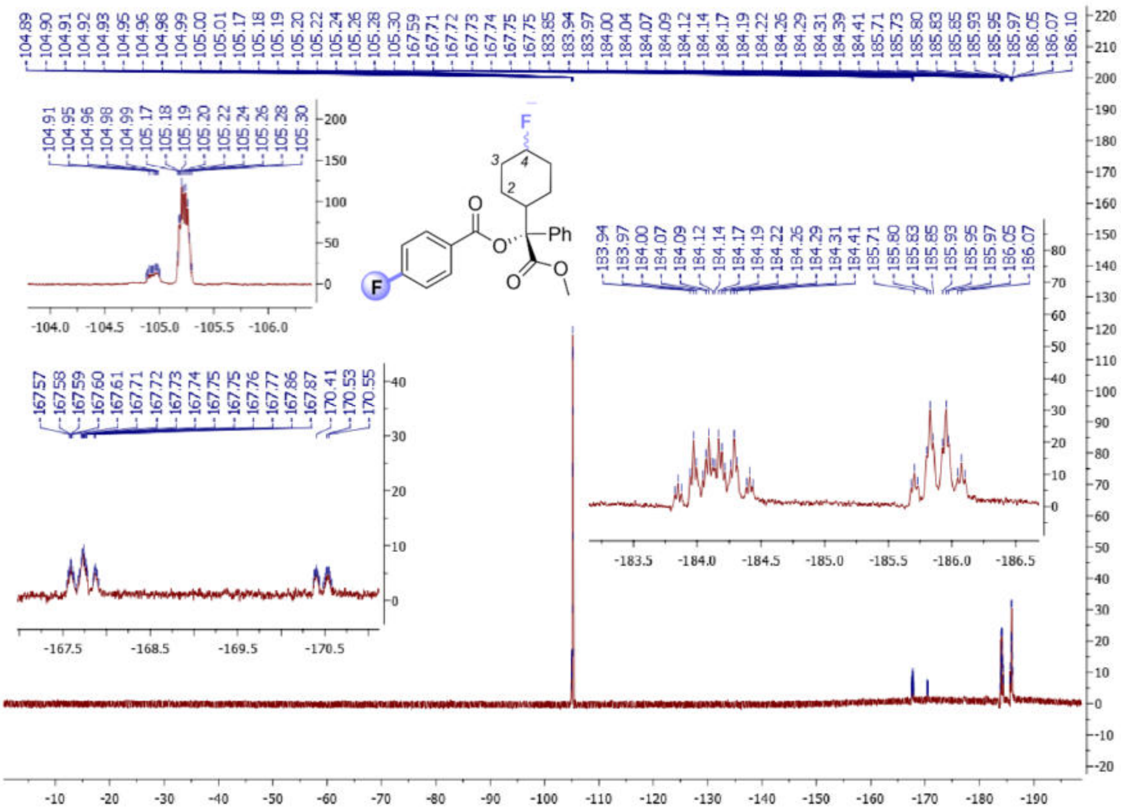

COSY NMR of compound **9i** in $CDCl_3$

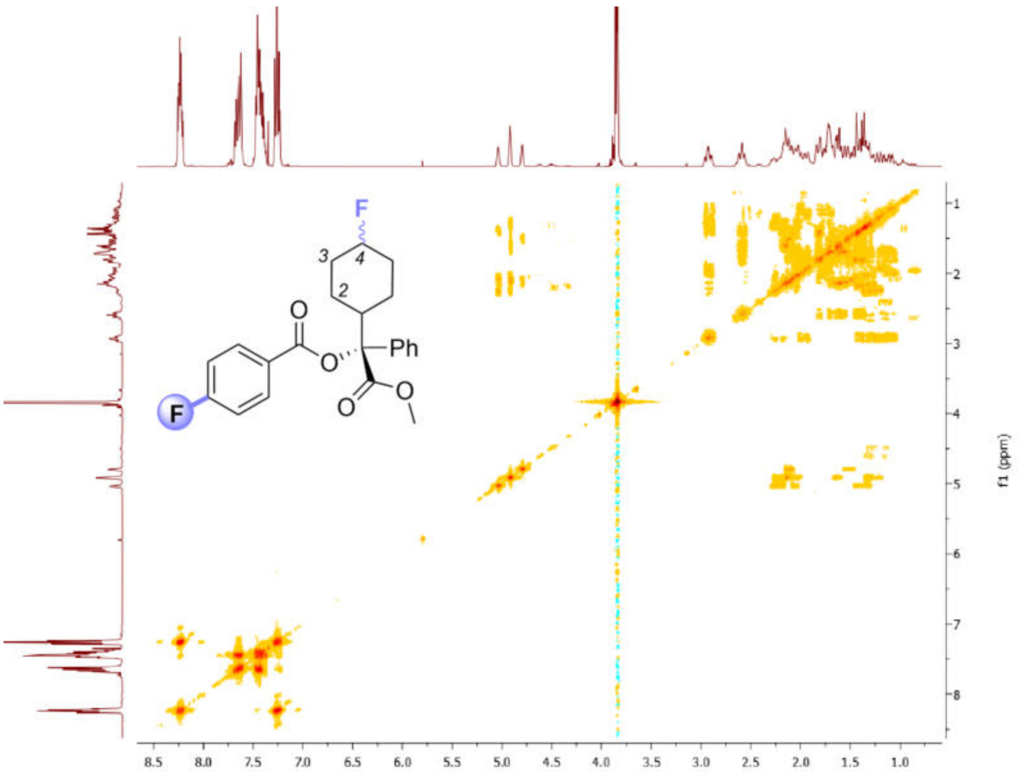

HSQC NMR of compound **9i** in $CDCl_3$

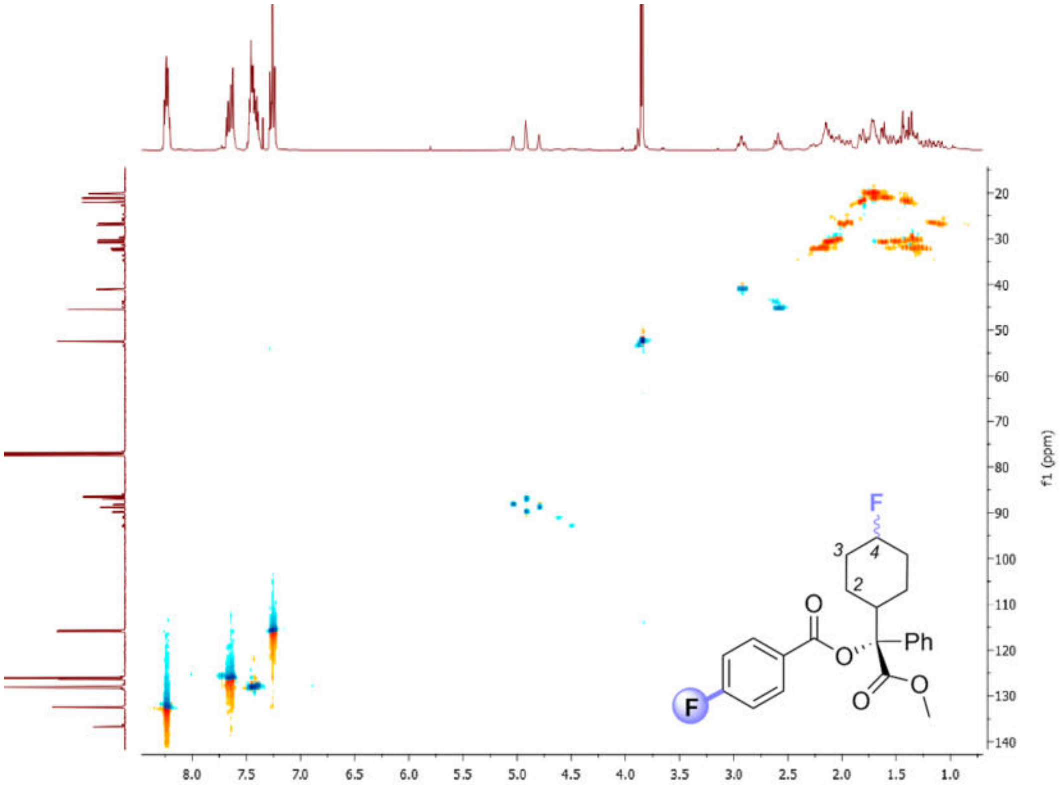

HMBC NMR of compound **9i** in $CDCl_3$

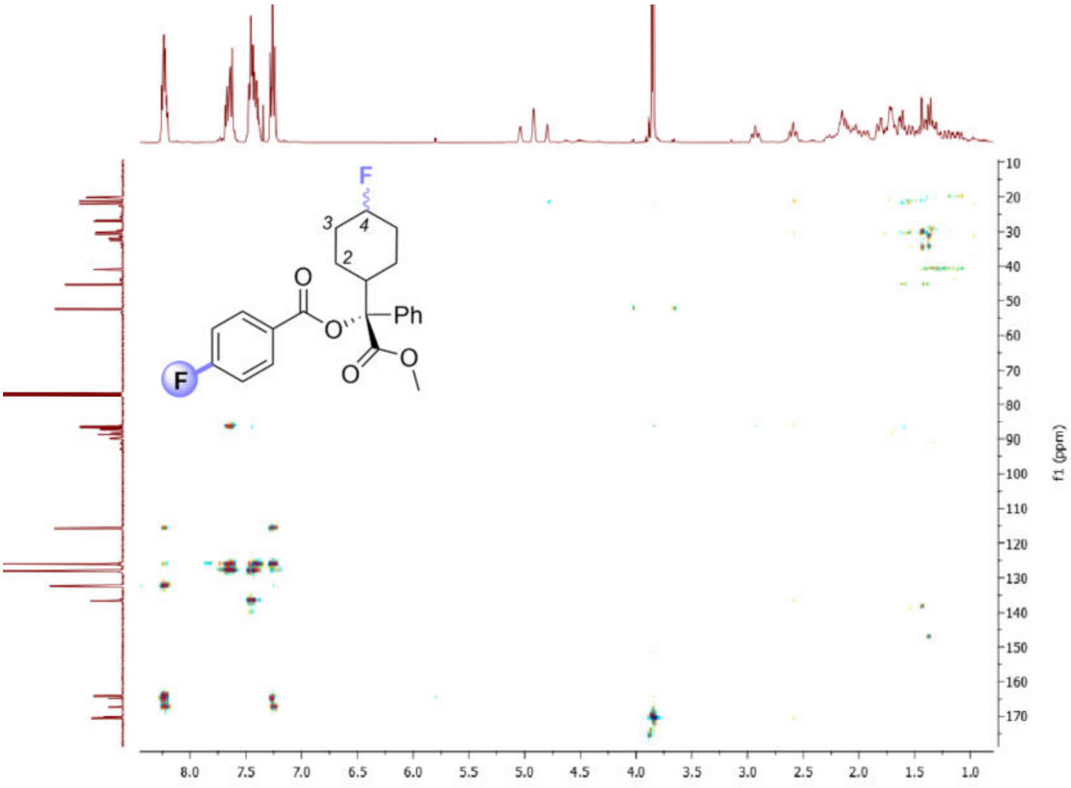

^{1}H NMR of compound **9a** in $CDCl_3$

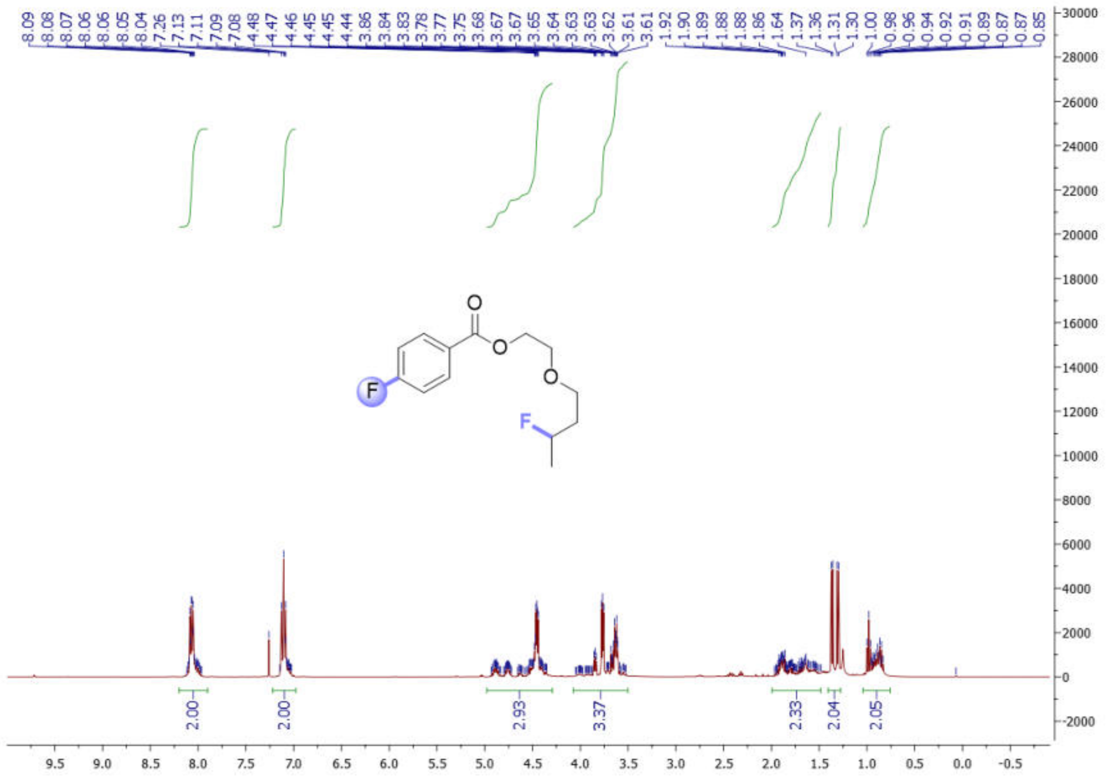

^{13}C NMR of compound **9a** in $CDCl_3$

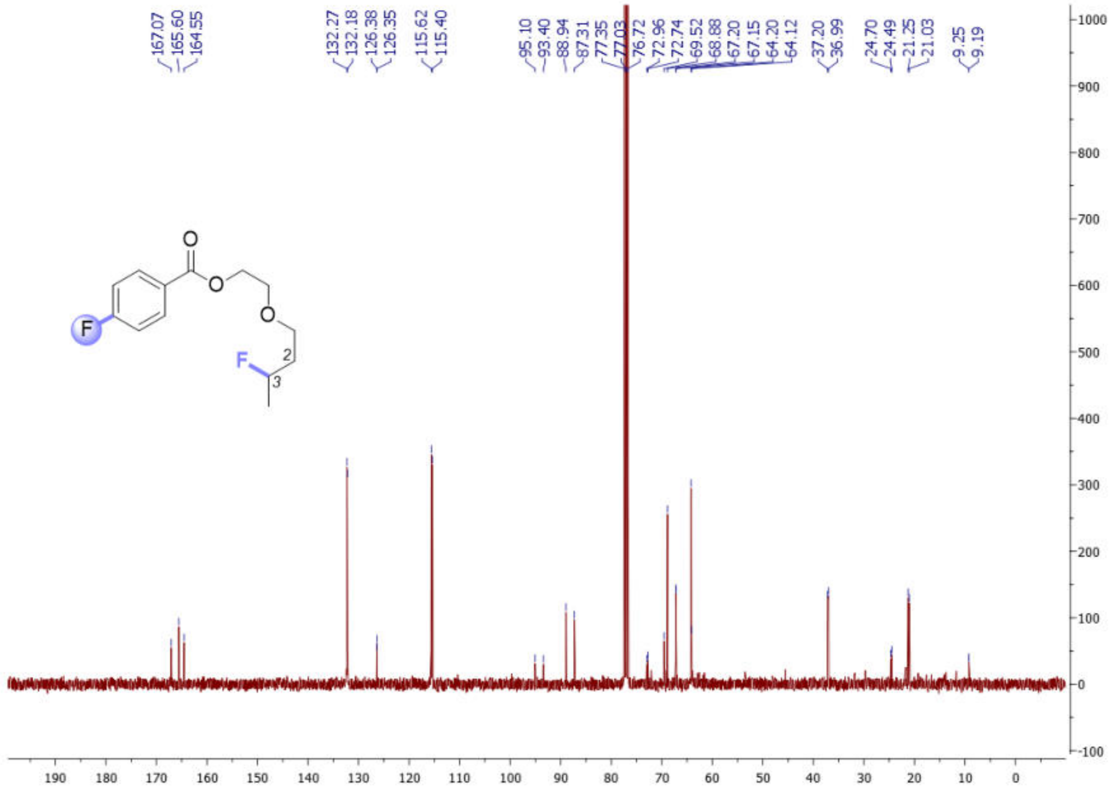

^{19}F NMR of compound **9a** in $CDCl_3$

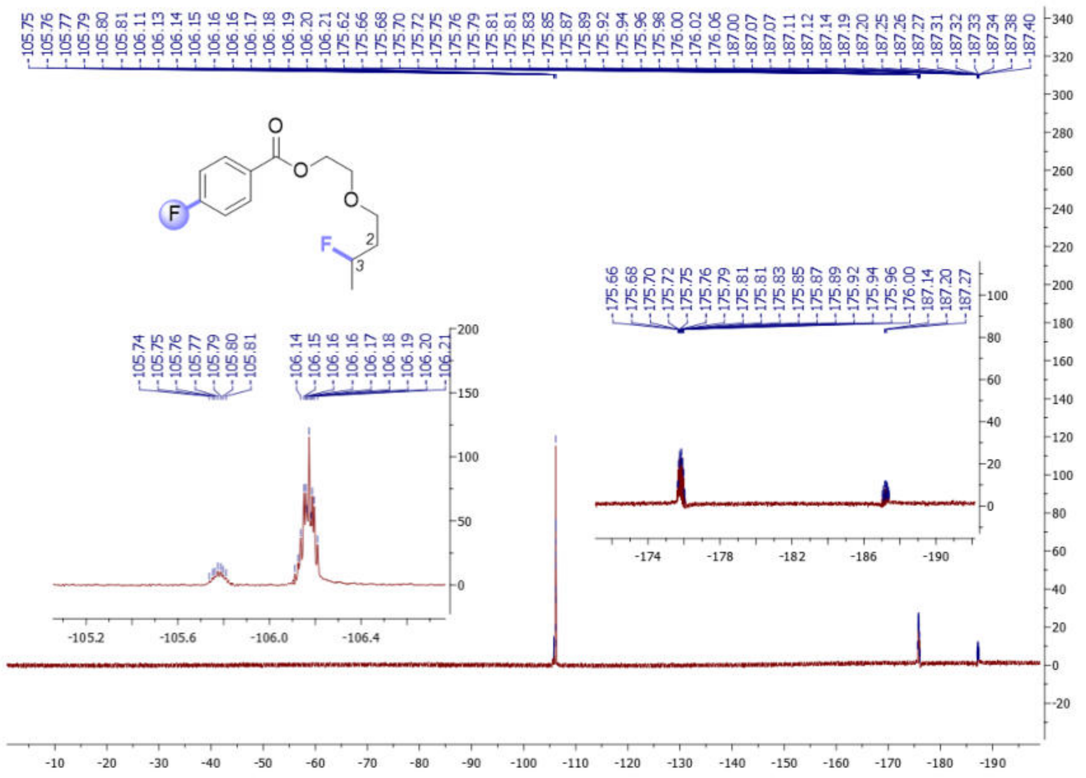

^{1}H NMR of compound **9b** in $CDCl_3$

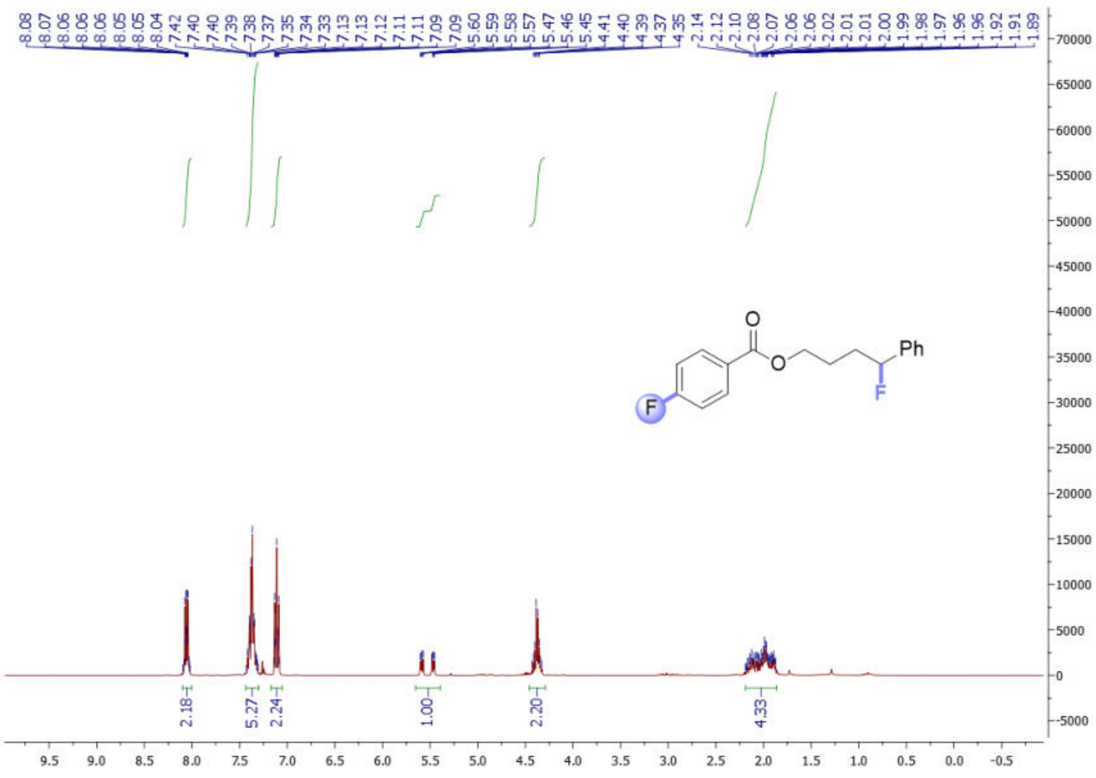

^{13}C NMR of compound **9b** in $CDCl_3$

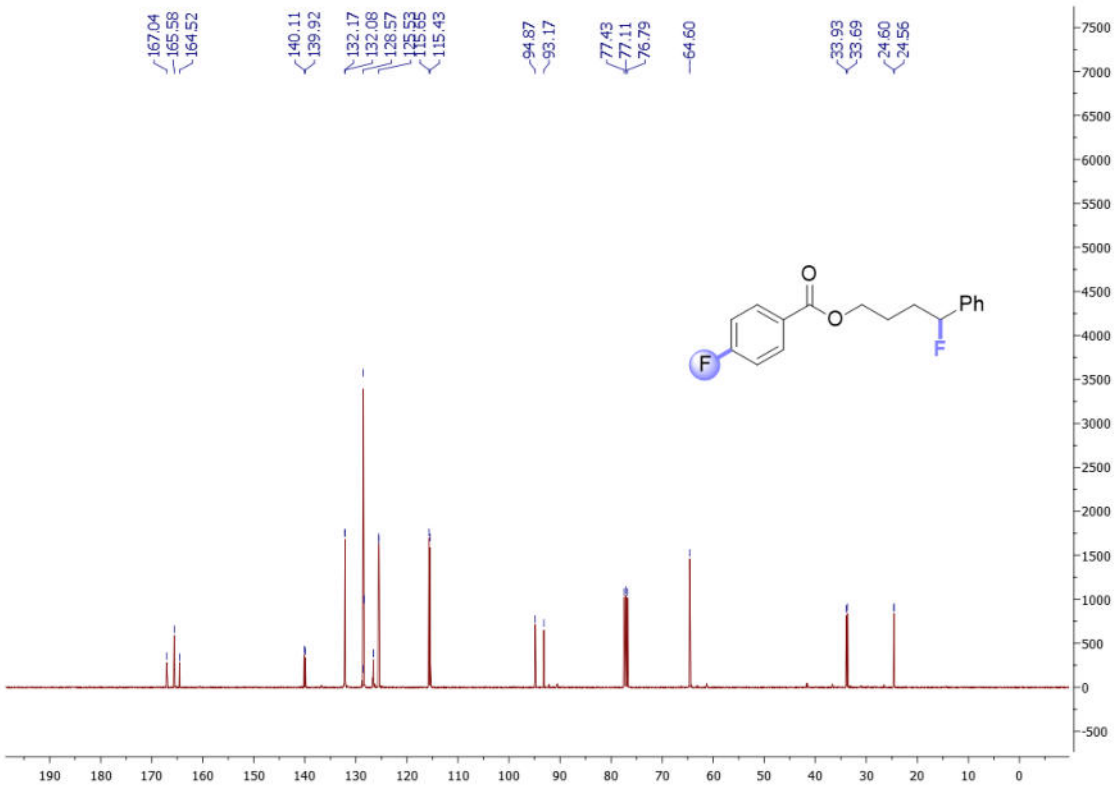

^{19}F NMR of compound **9b** in $CDCl_3$

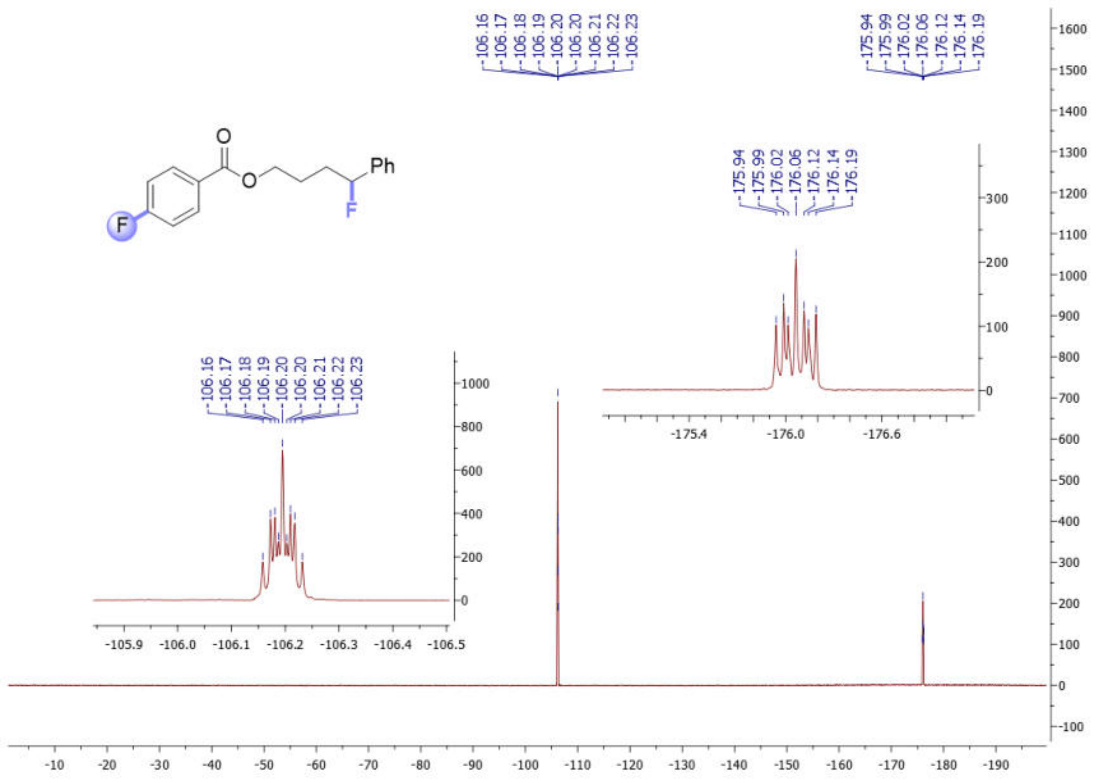

^{1}H NMR of compound **9c** in $CDCl_3$

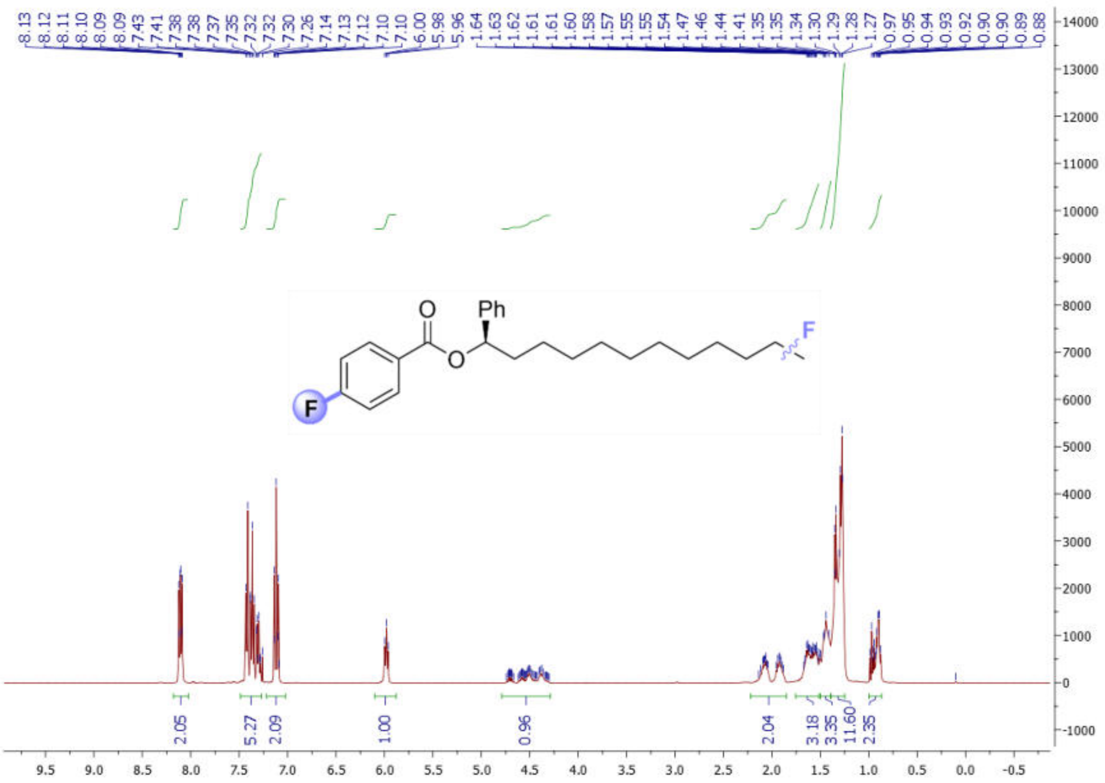

^{13}C NMR of compound **9c** in $CDCl_3$

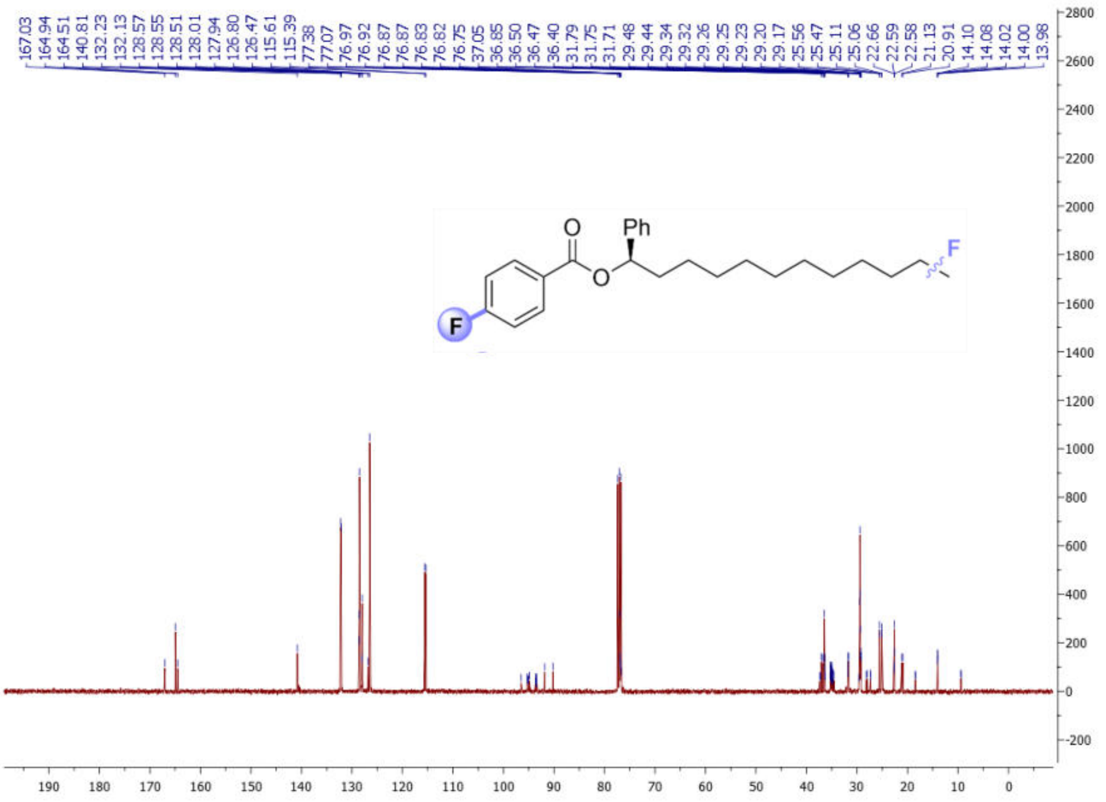

^{19}F NMR of compound **9c** in $CDCl_3$

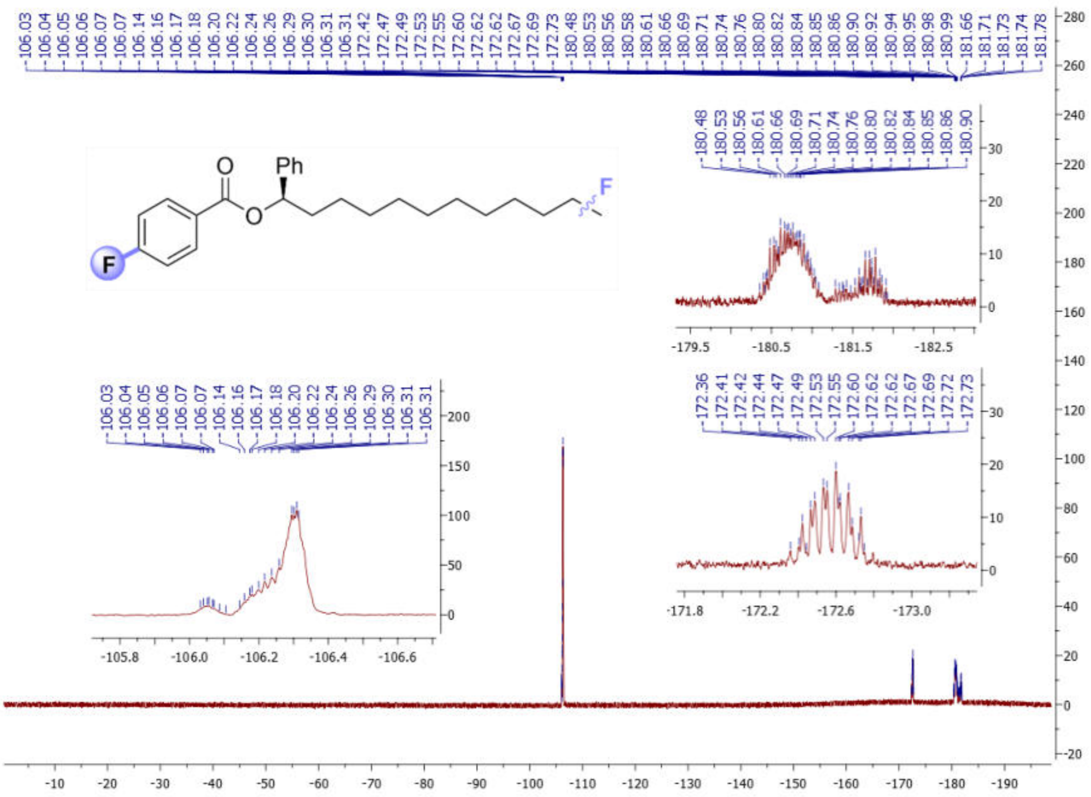

COSY NMR of compound **9c** in $CDCl_3$

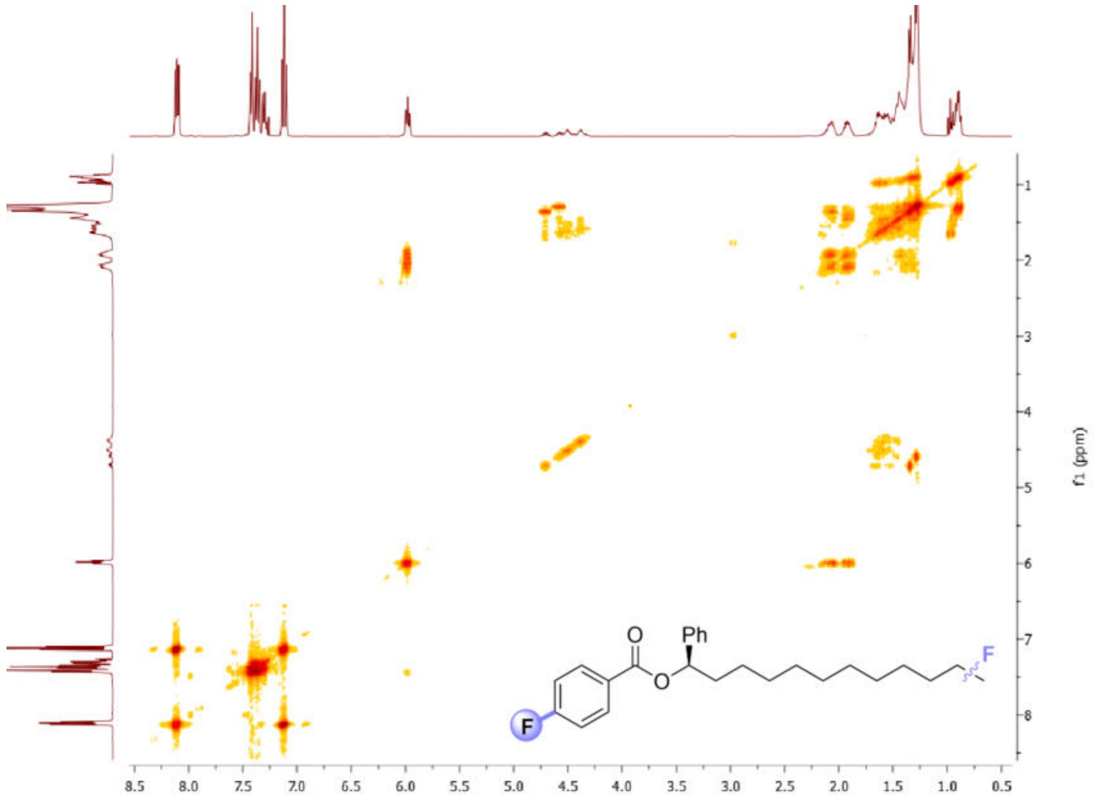

HSQC NMR of compound **9c** in $CDCl_3$

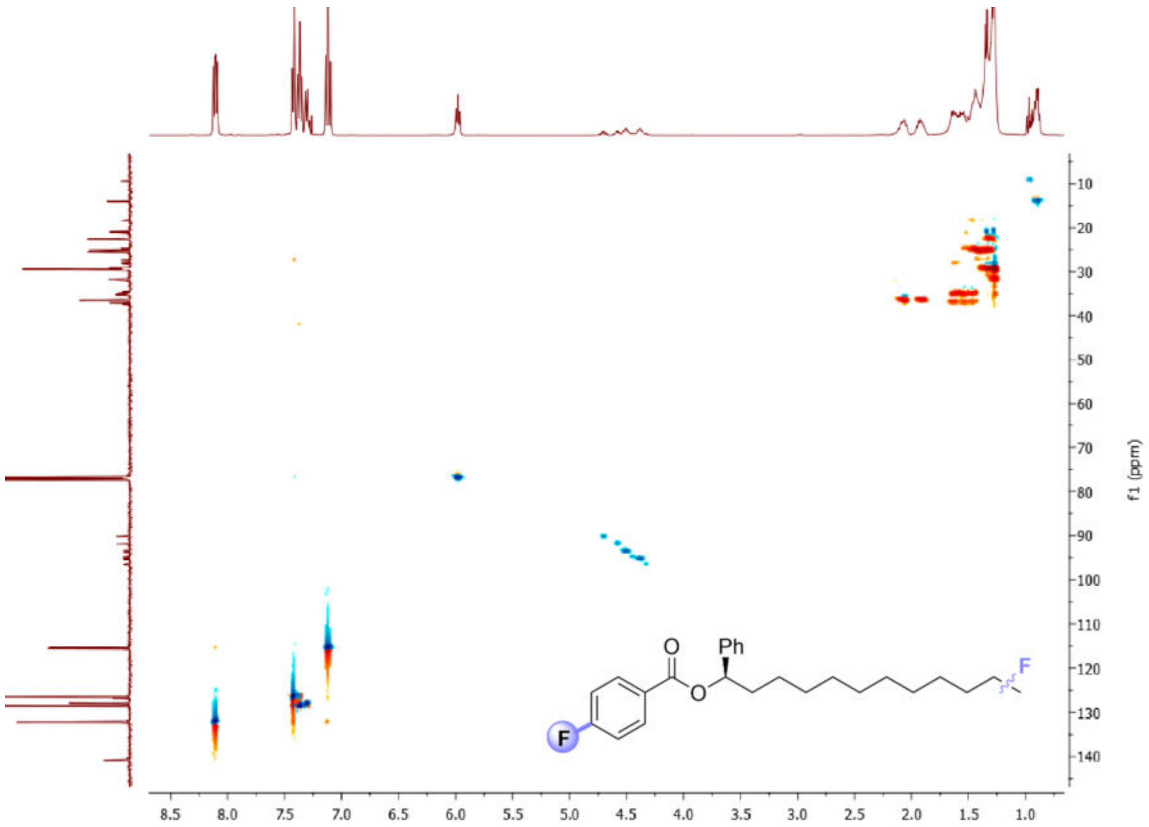

HMBC NMR of compound **9c** in $CDCl_3$

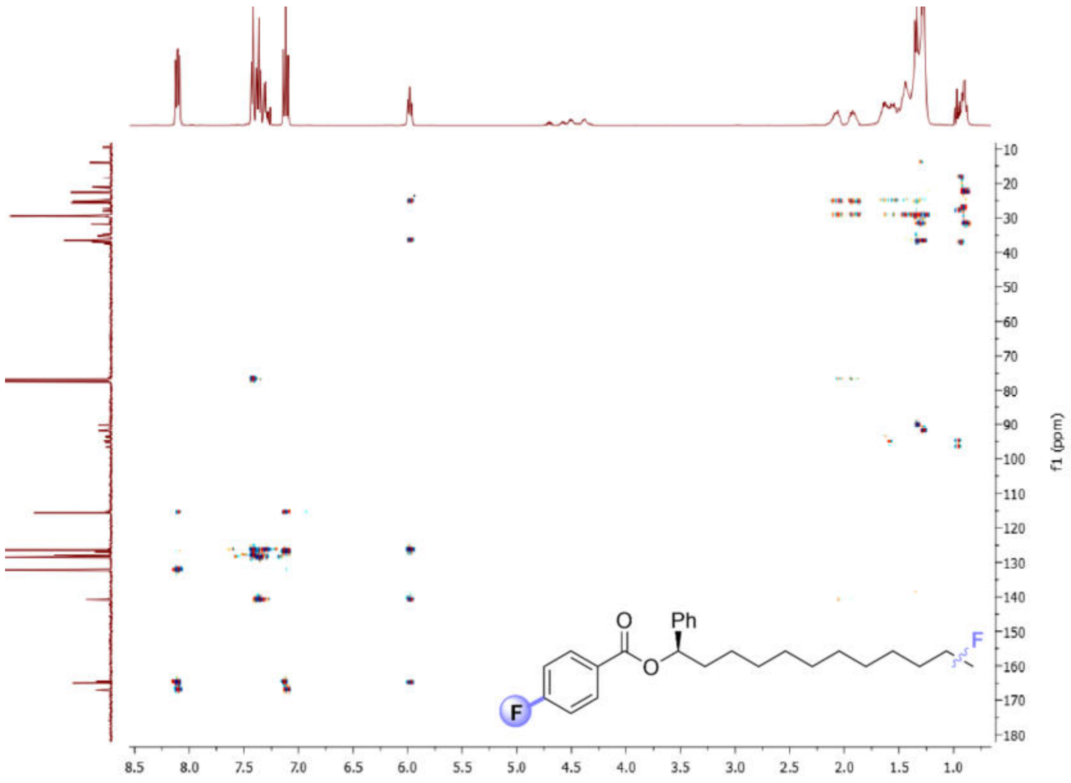

^{1}H NMR of compound **9d** in $CDCl_3$

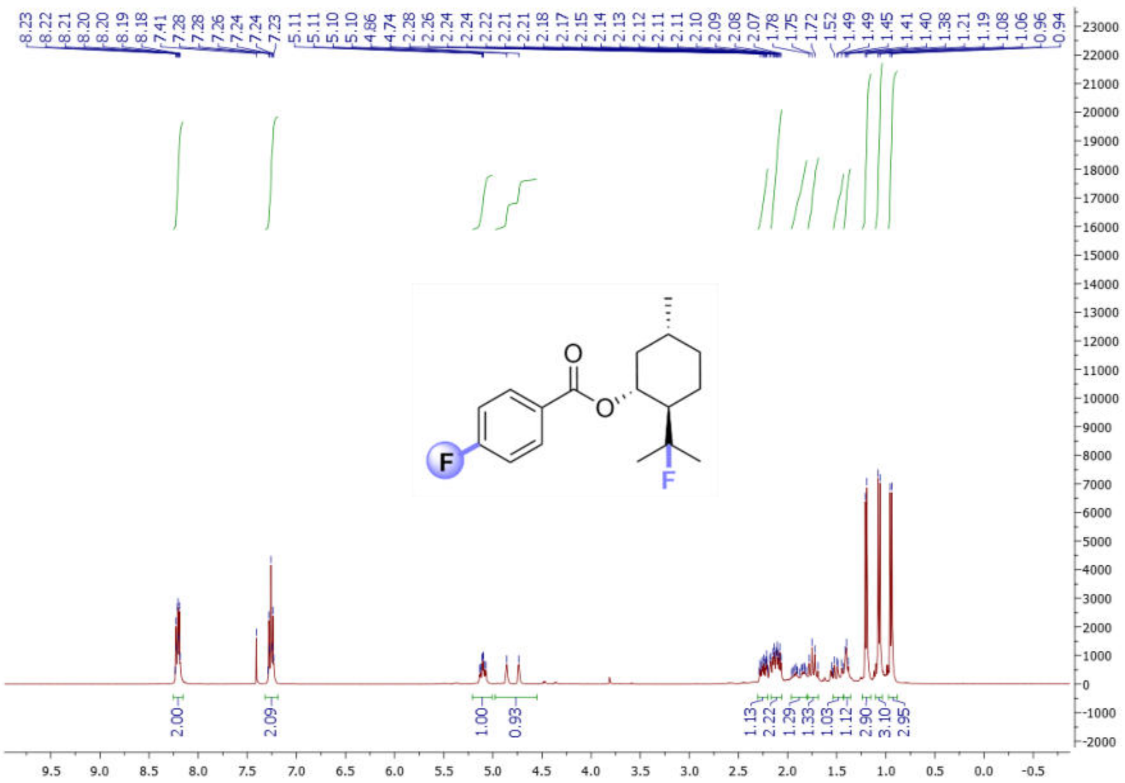

^{13}C NMR of compound **9d** in $CDCl_3$

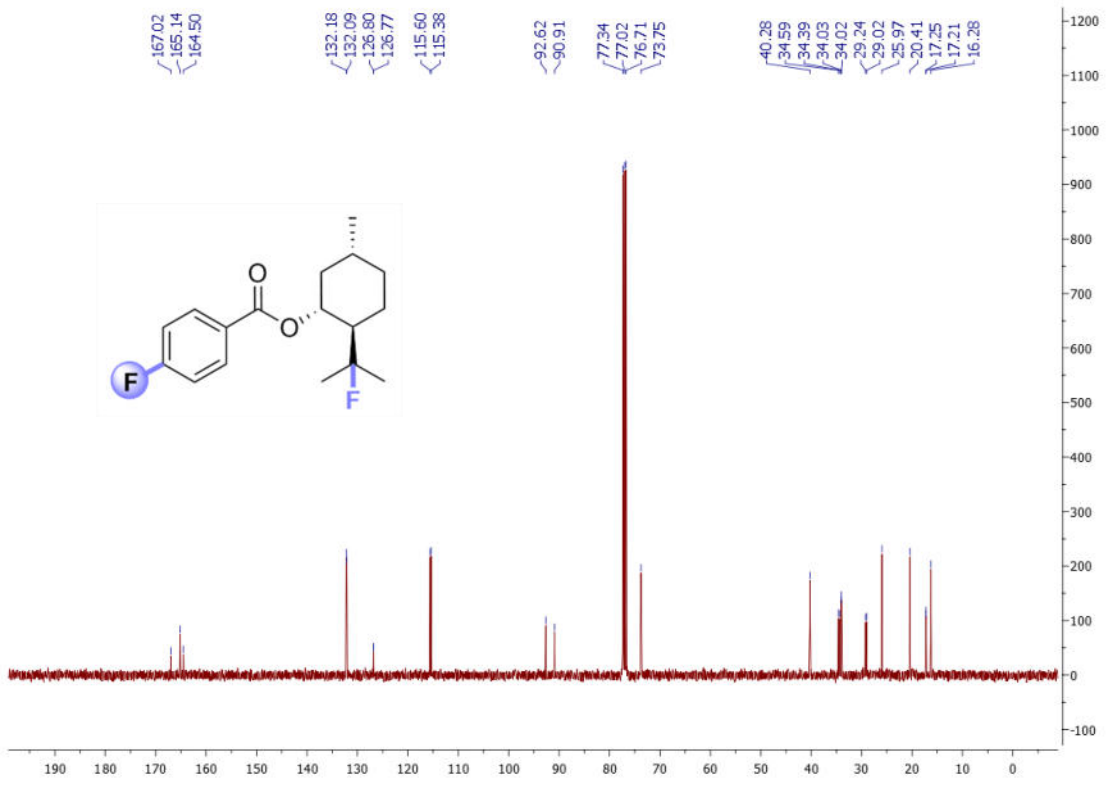

^{19}F NMR of compound **9d** in $CDCl_3$

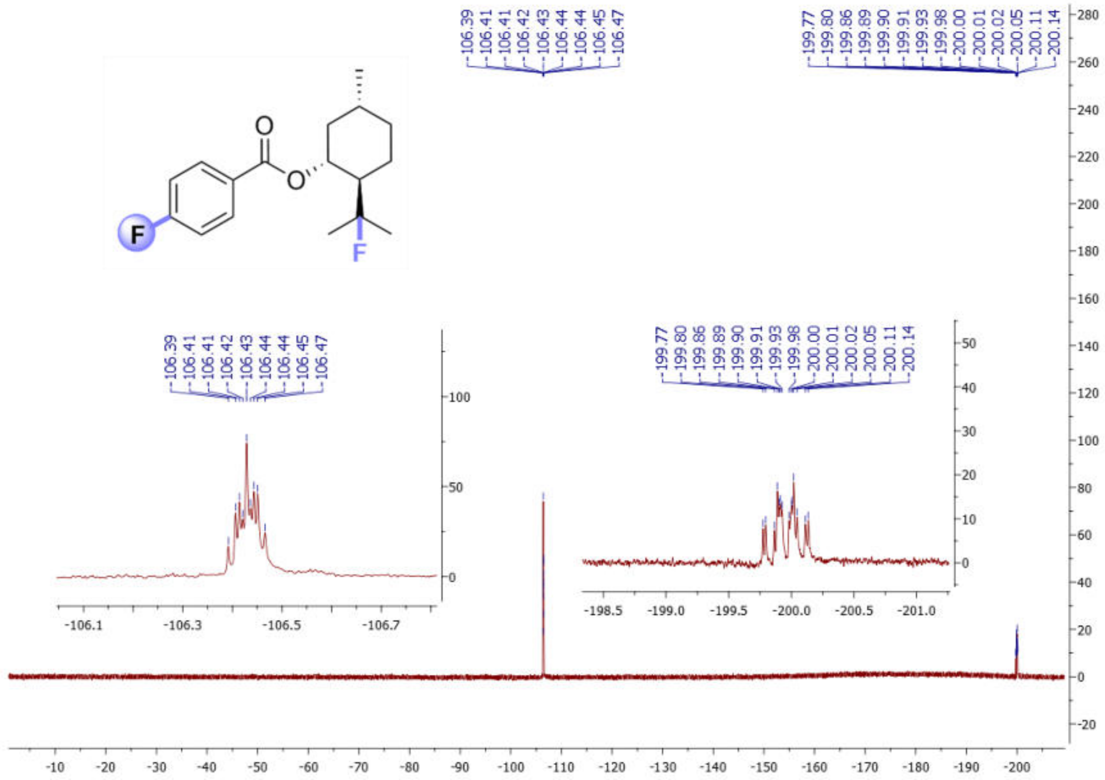

^{1}H NMR of compound **9e** in $CDCl_3$

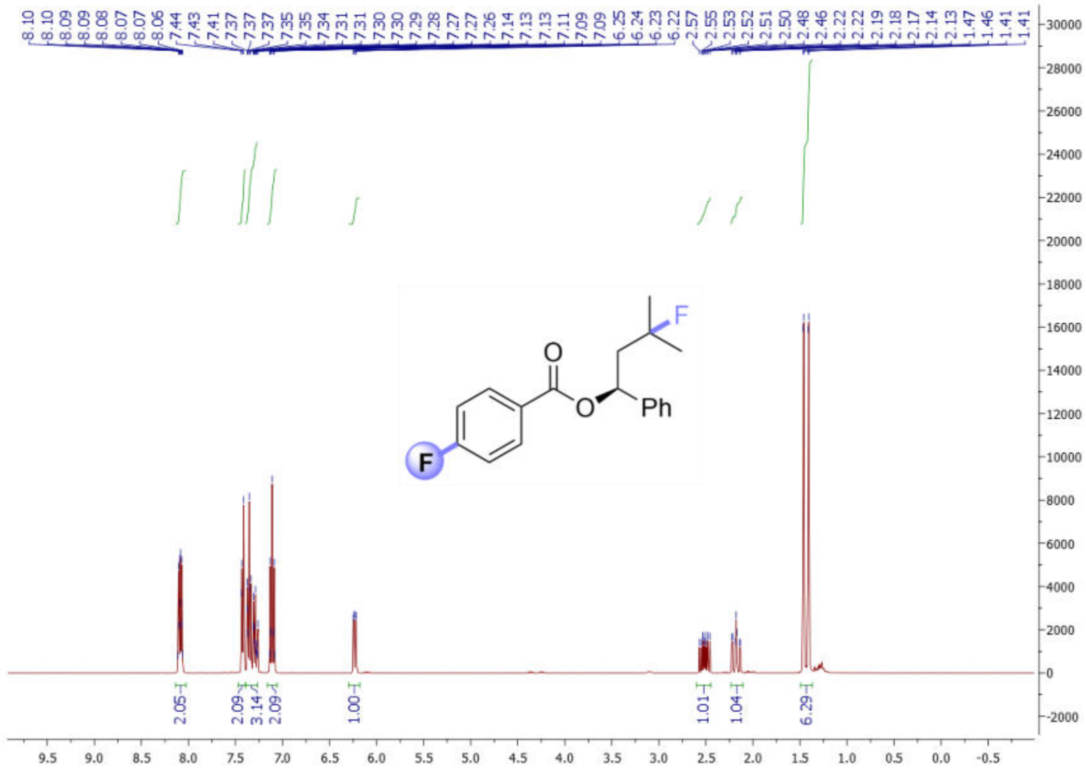

^{13}C NMR of compound **9e** in $CDCl_3$

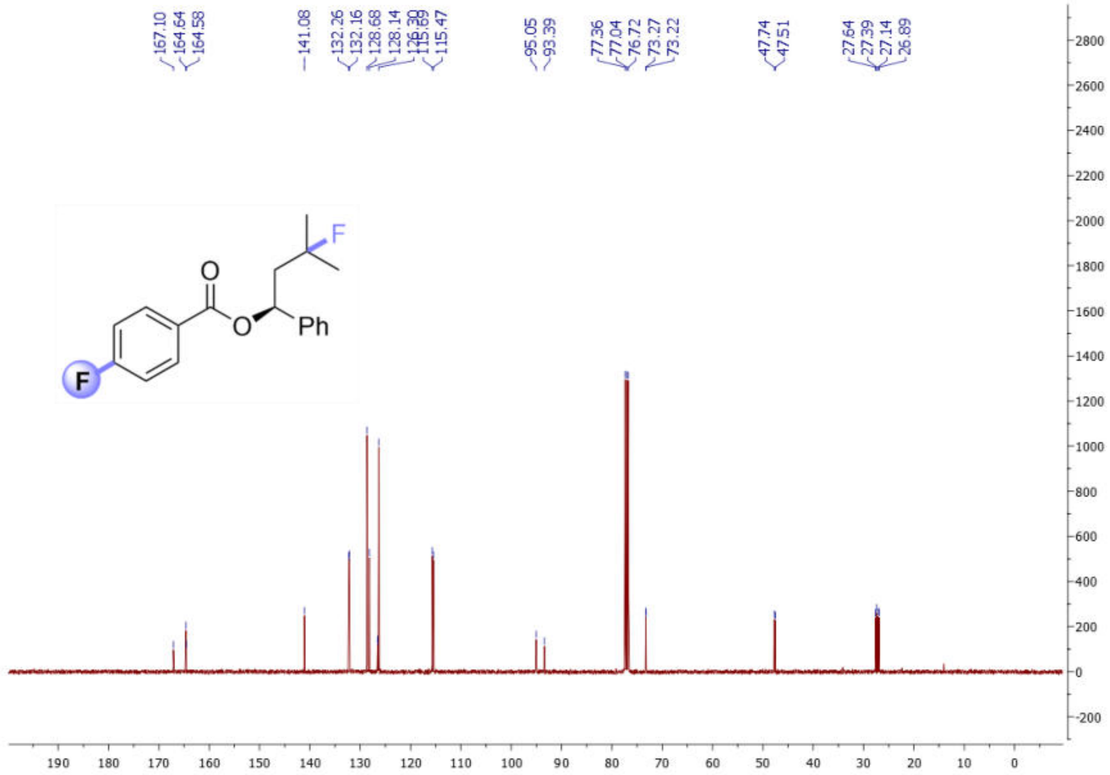

^{19}F NMR of compound **9e** in $CDCl_3$

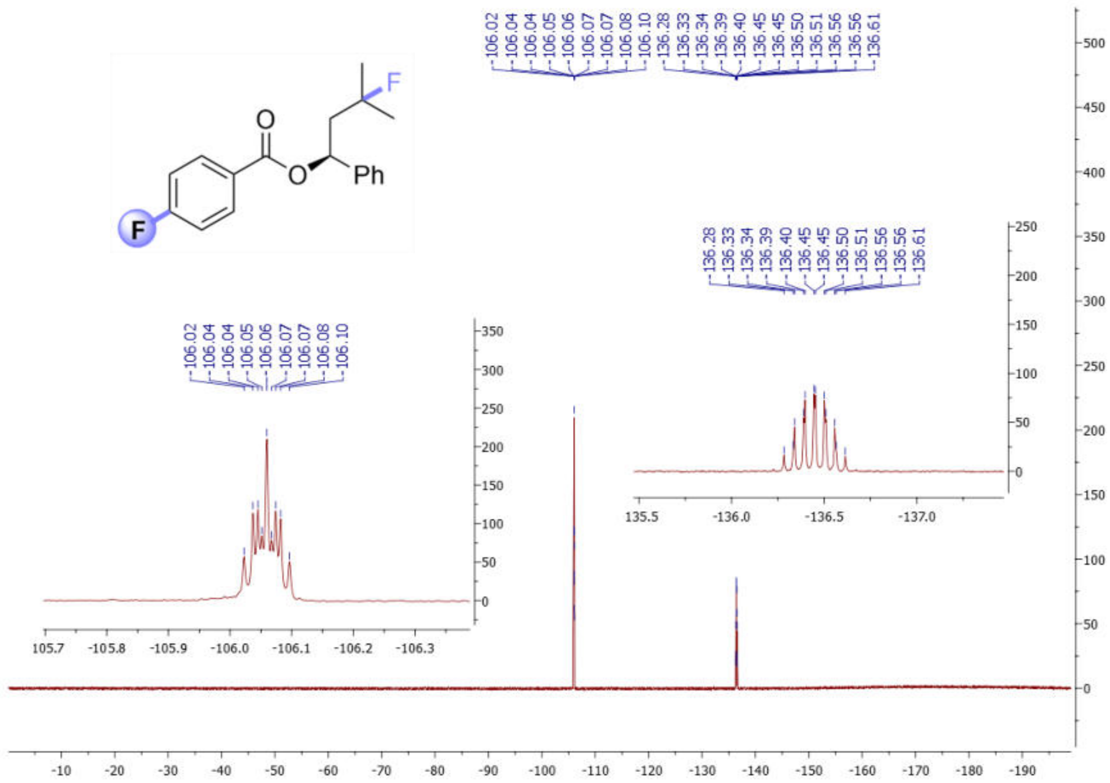

^{1}H NMR of compound **9f** in $CDCl_3$

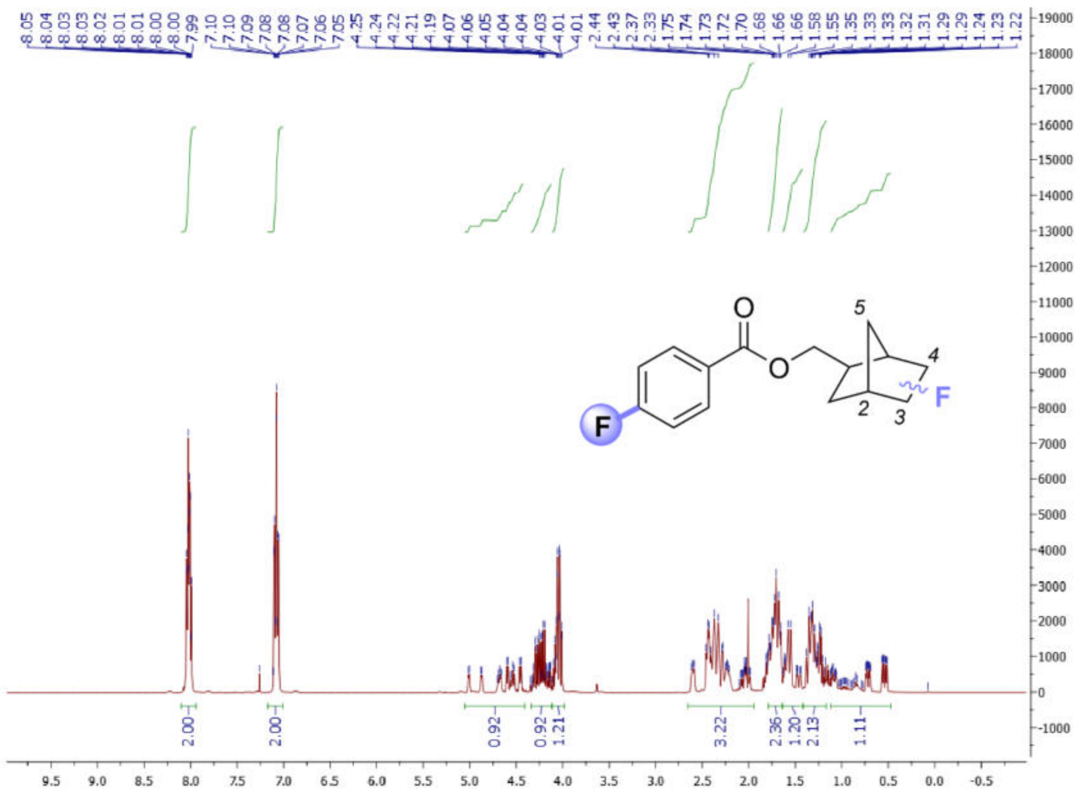

^{13}C NMR of compound **9f** in $CDCl_3$

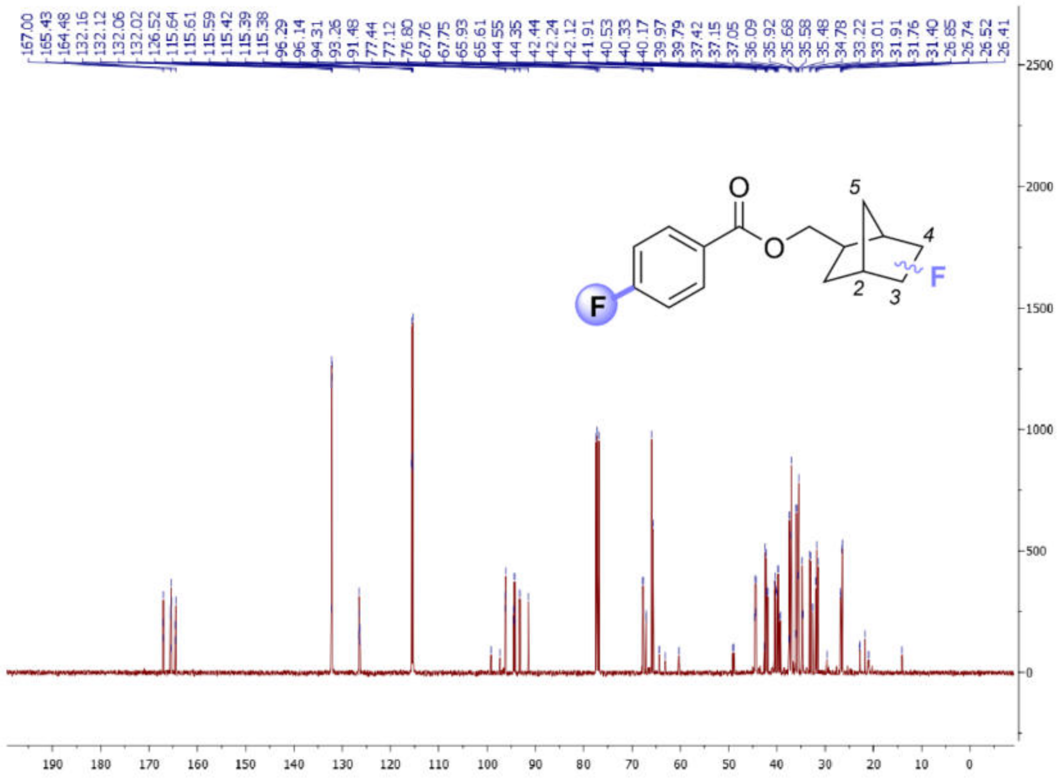

^{19}F NMR of compound **9f** in $CDCl_3$

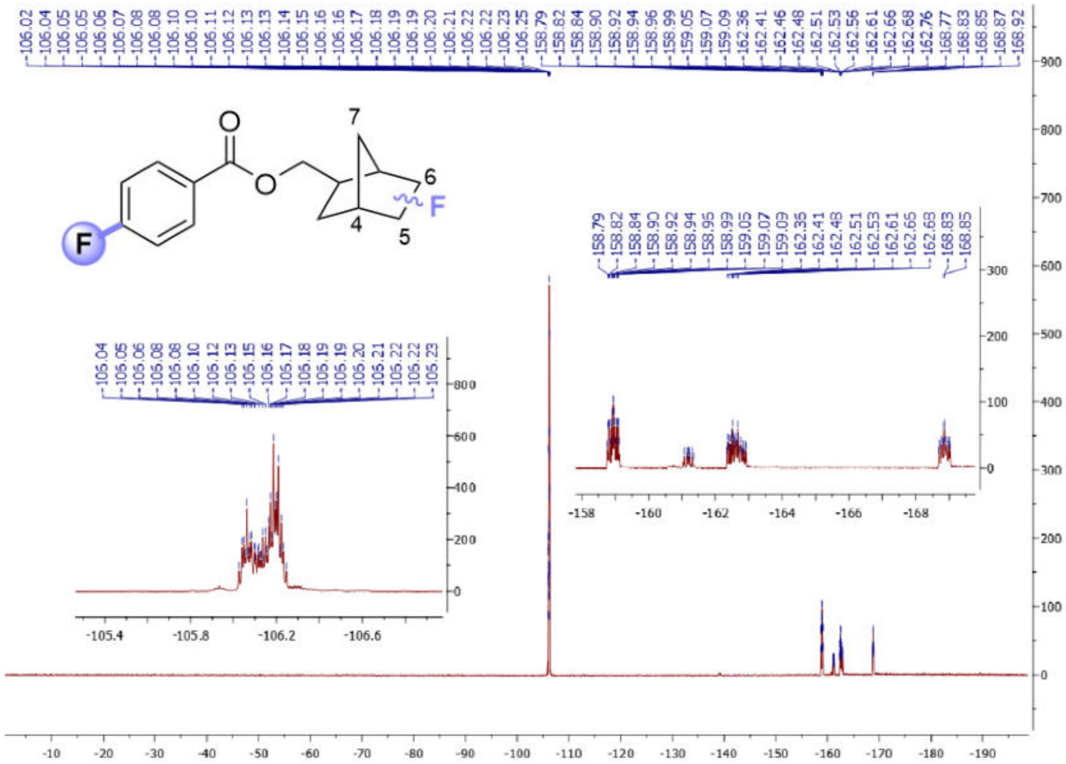

COSY NMR of compound **9f** in $CDCl_3$

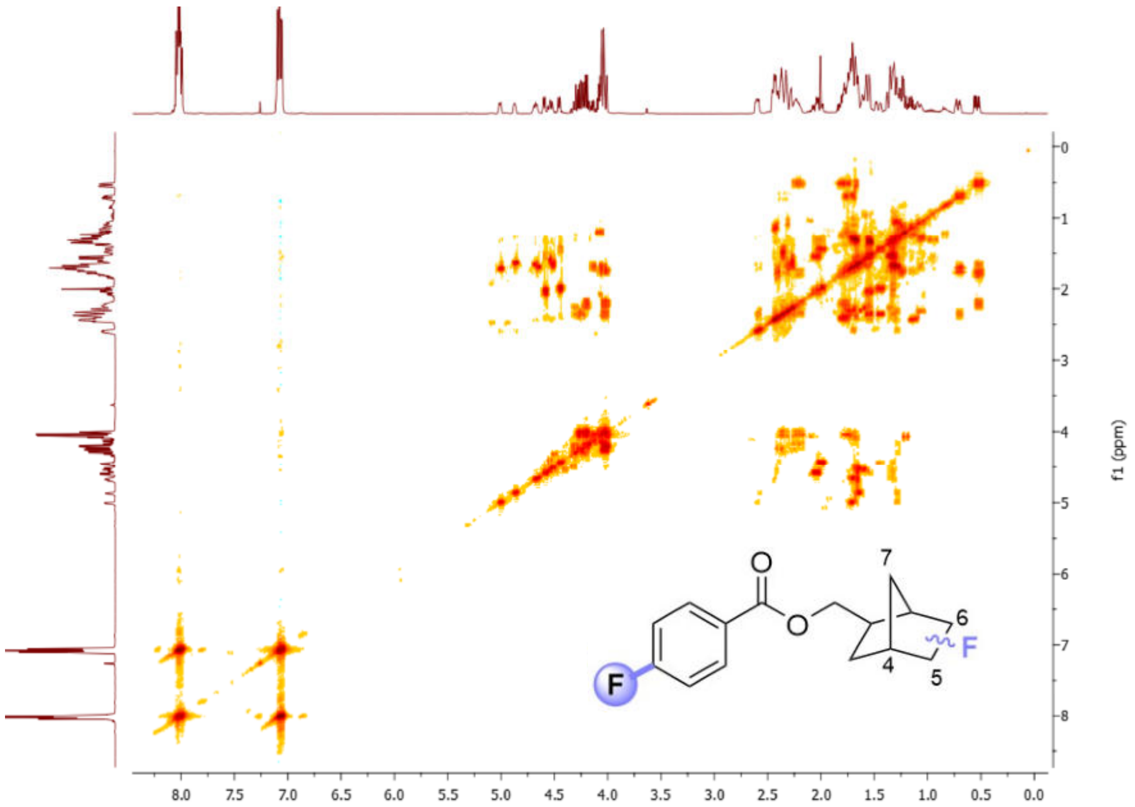

HSQC NMR of compound **9f** in $CDCl_3$

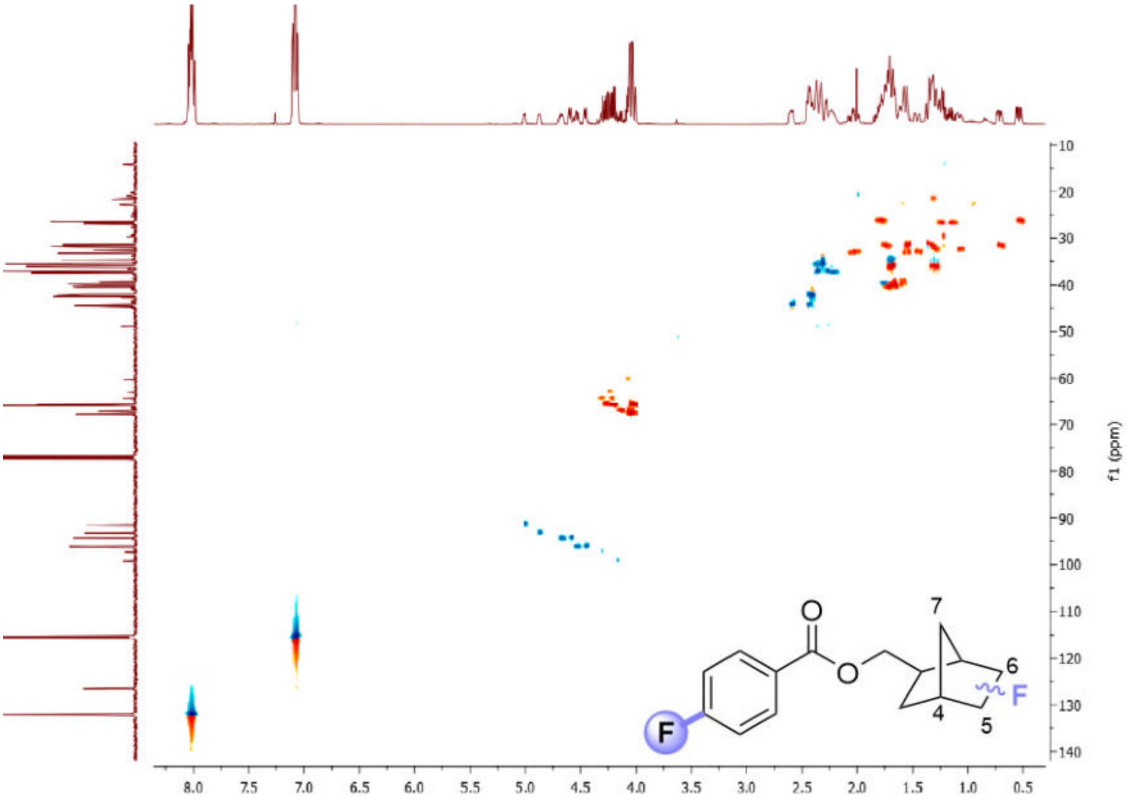

HMBC NMR of compound **9f** in $CDCl_3$

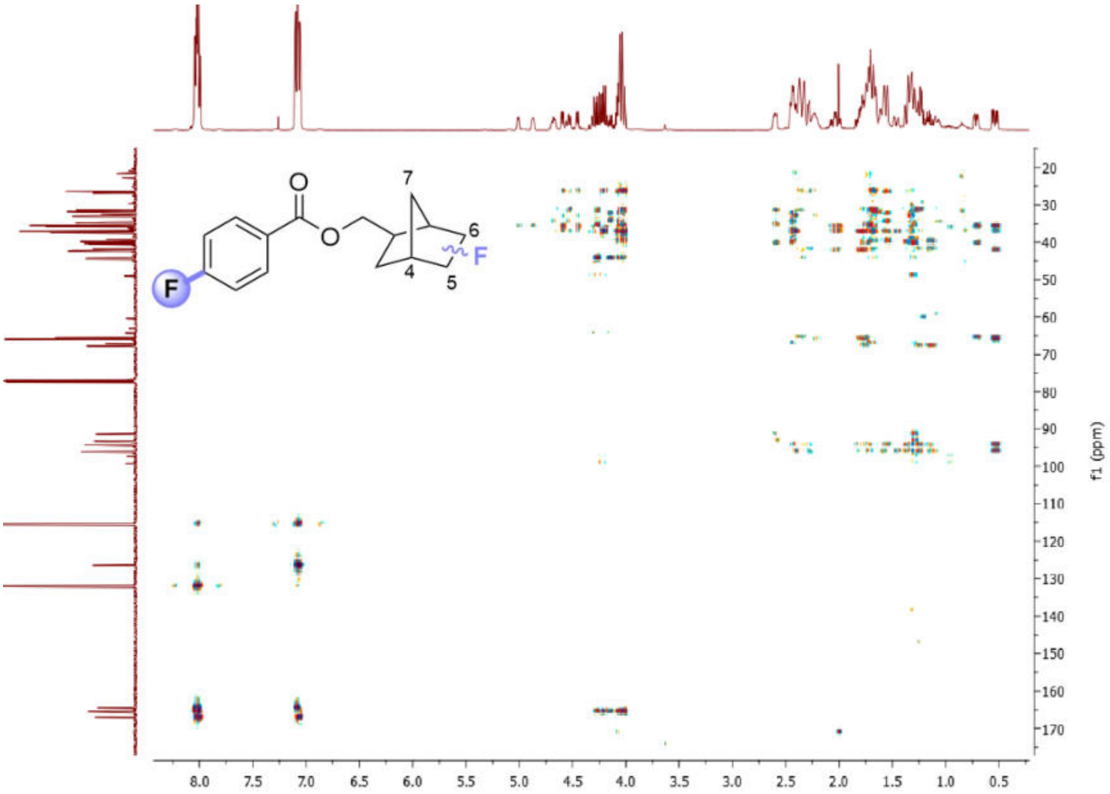

^{1}H NMR of compound **9g** in $CDCl_3$

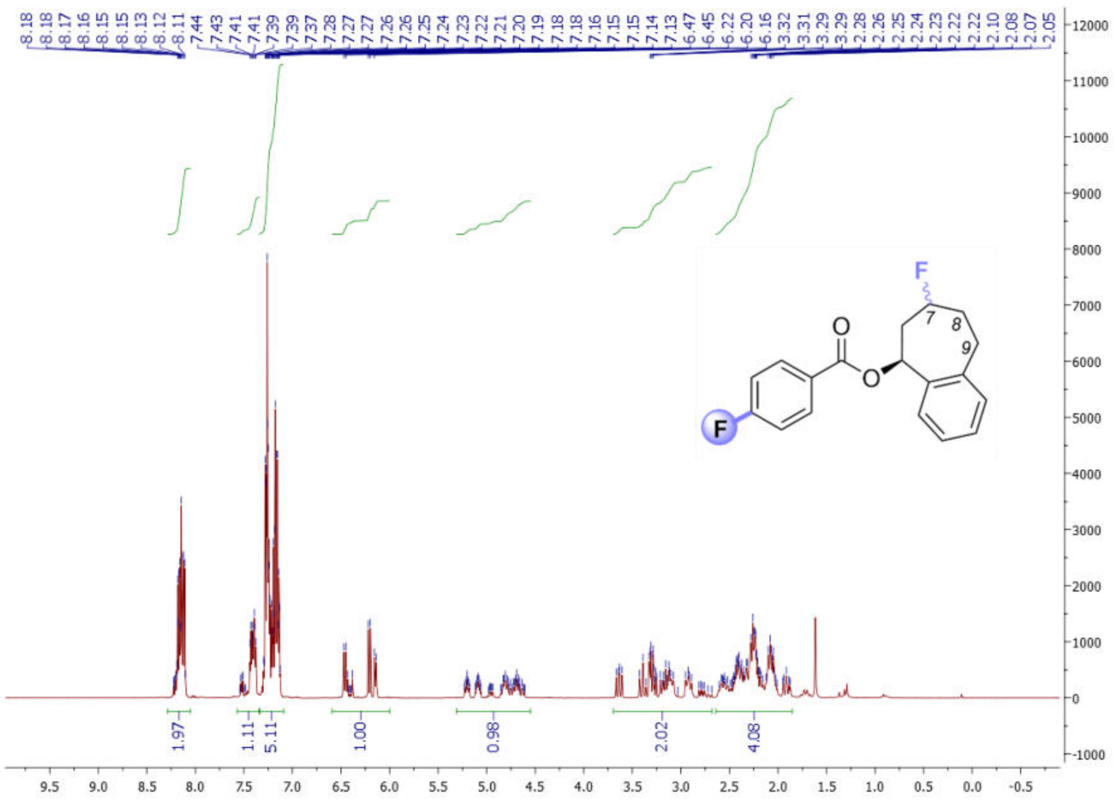

^{13}C NMR of compound **9g** in $CDCl_3$

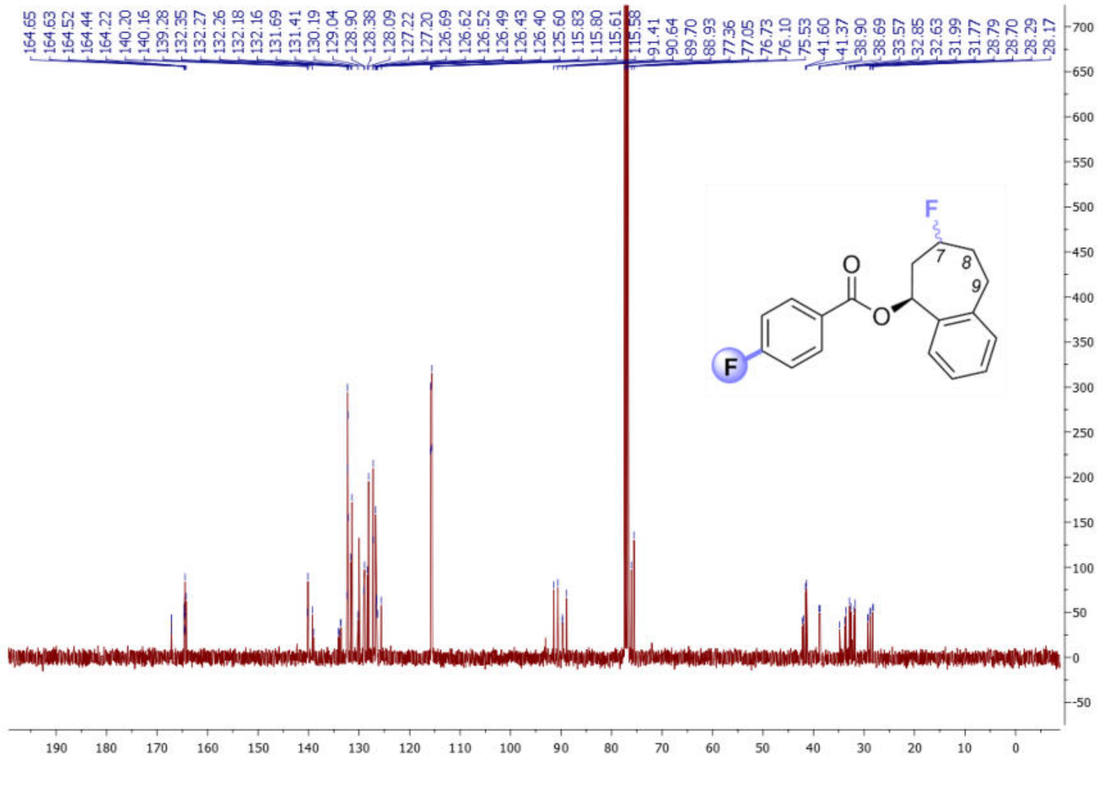

^{1}H NMR of compound **9h** in $CDCl_3$

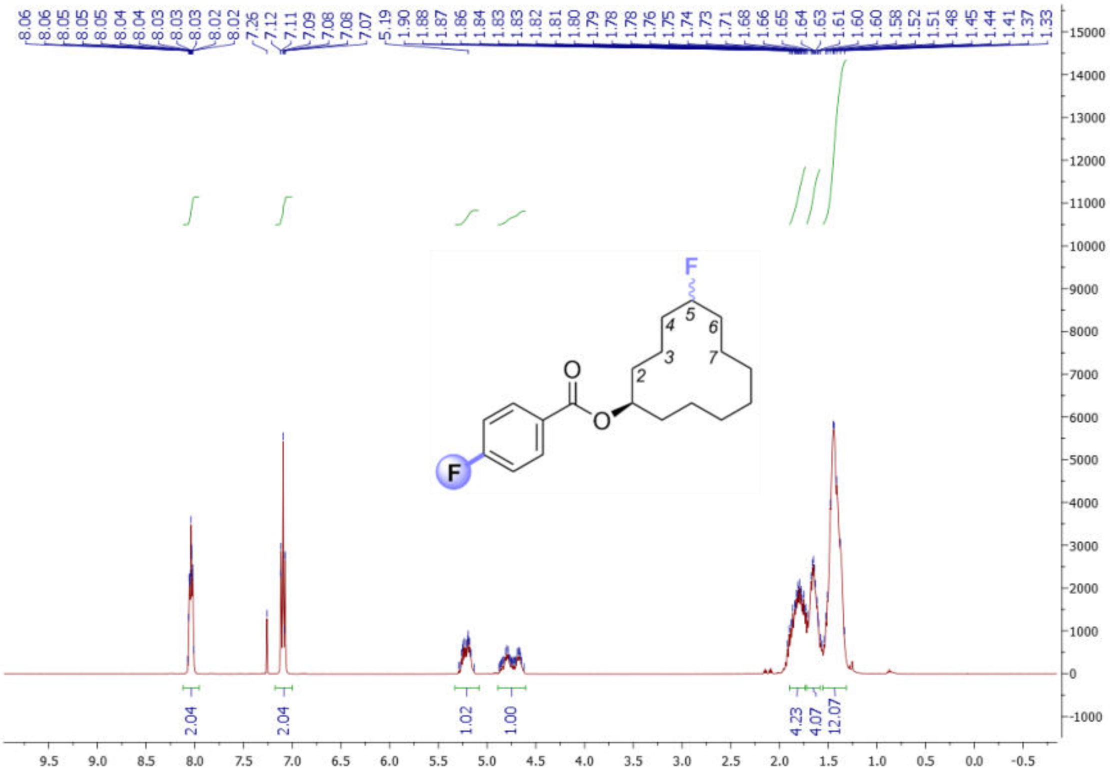

^{13}C NMR of compound **9h** in $CDCl_3$

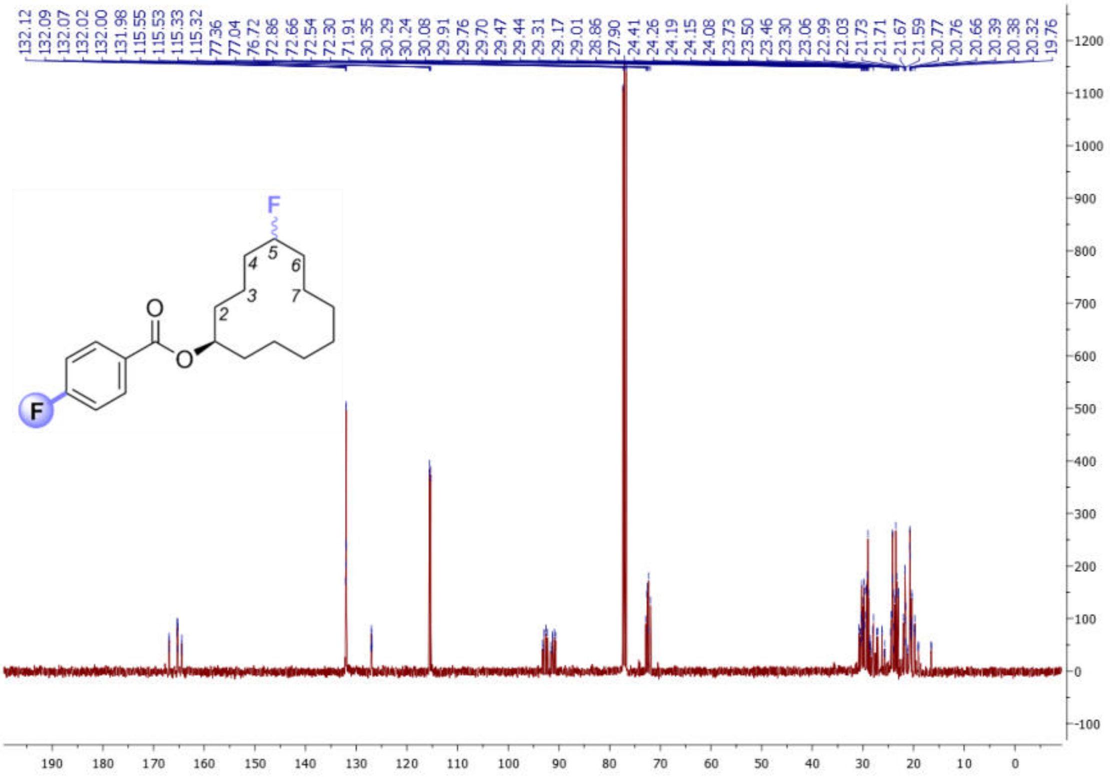

^{19}F NMR of compound **9h** in $CDCl_3$

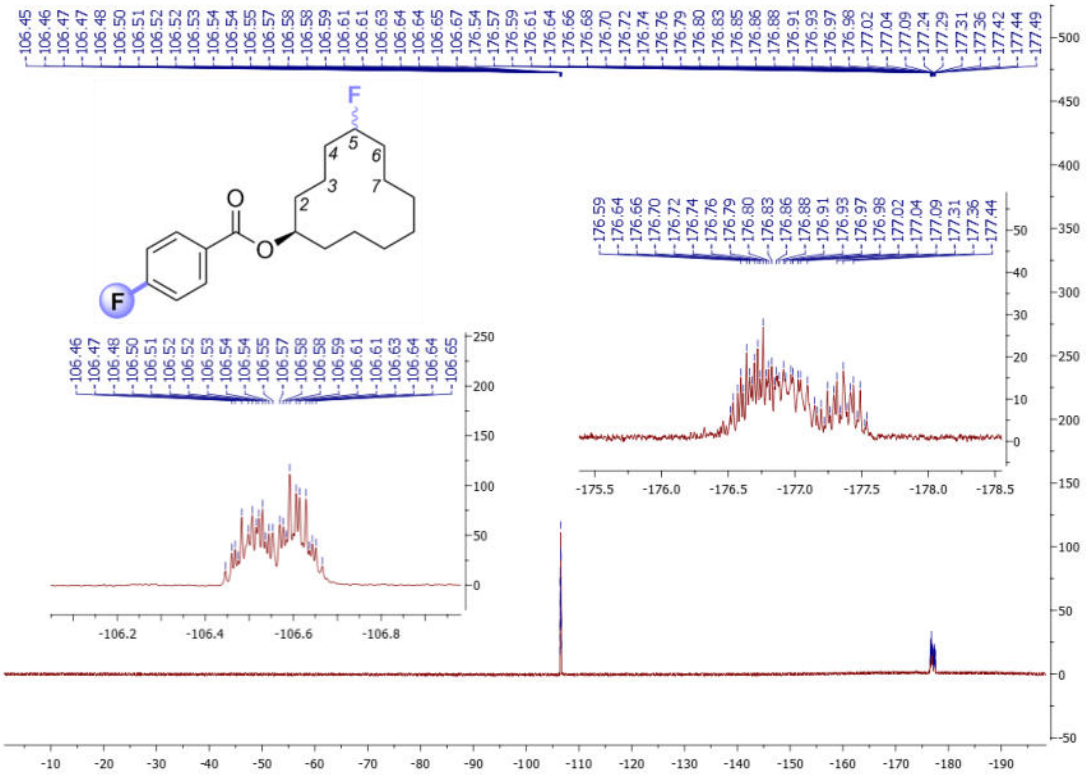

^{19}F{^{1}H} NMR of compound **9h** in $CDCl_3$

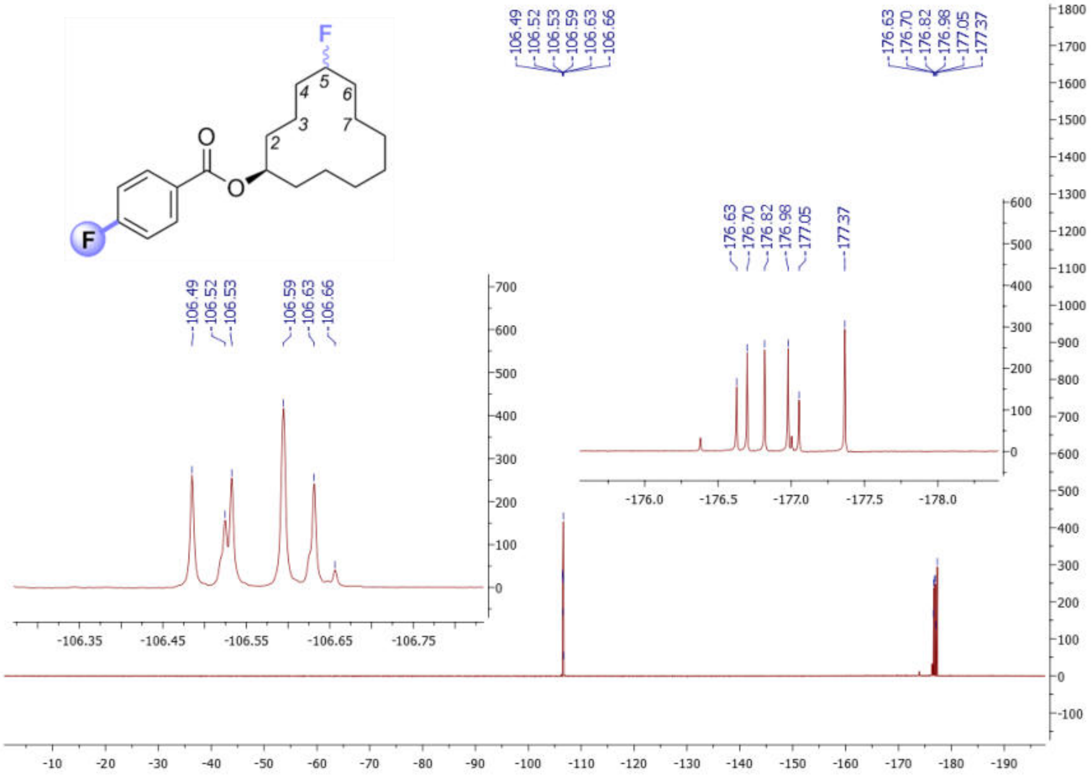

^1H NMR of compound **9j** in $CDCl_3$

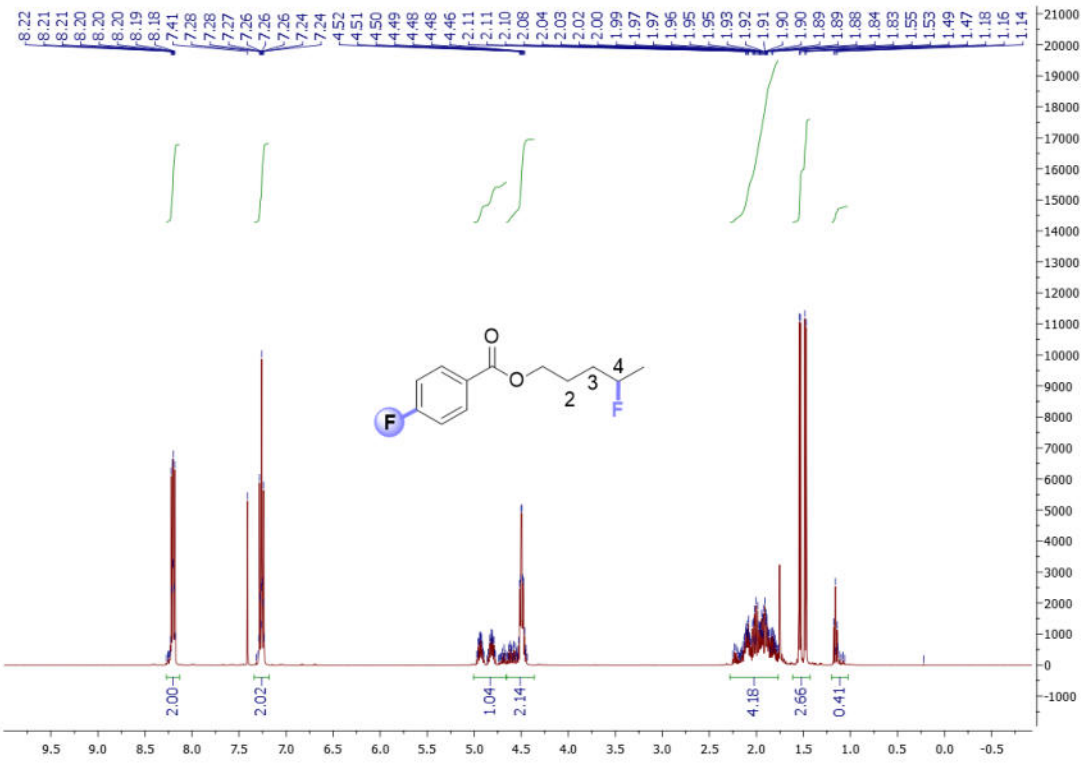

^{13}C NMR of compound **9j** in $CDCl_3$

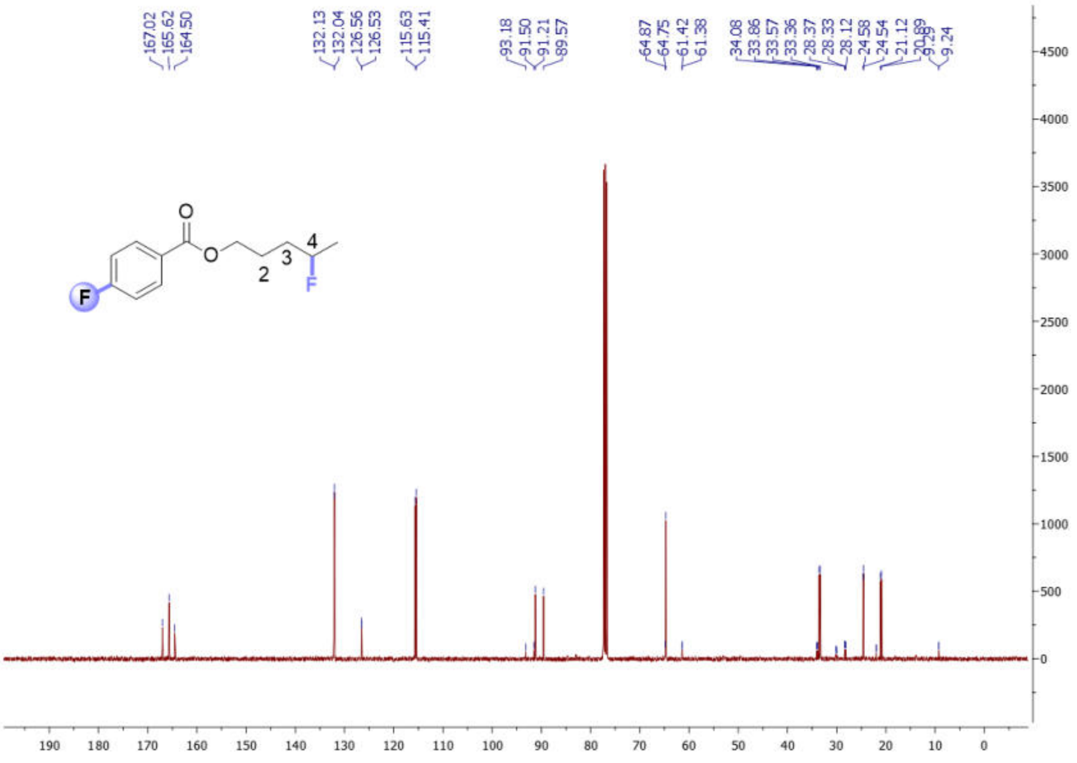

^{19}F NMR of compound **9j** in $CDCl_3$

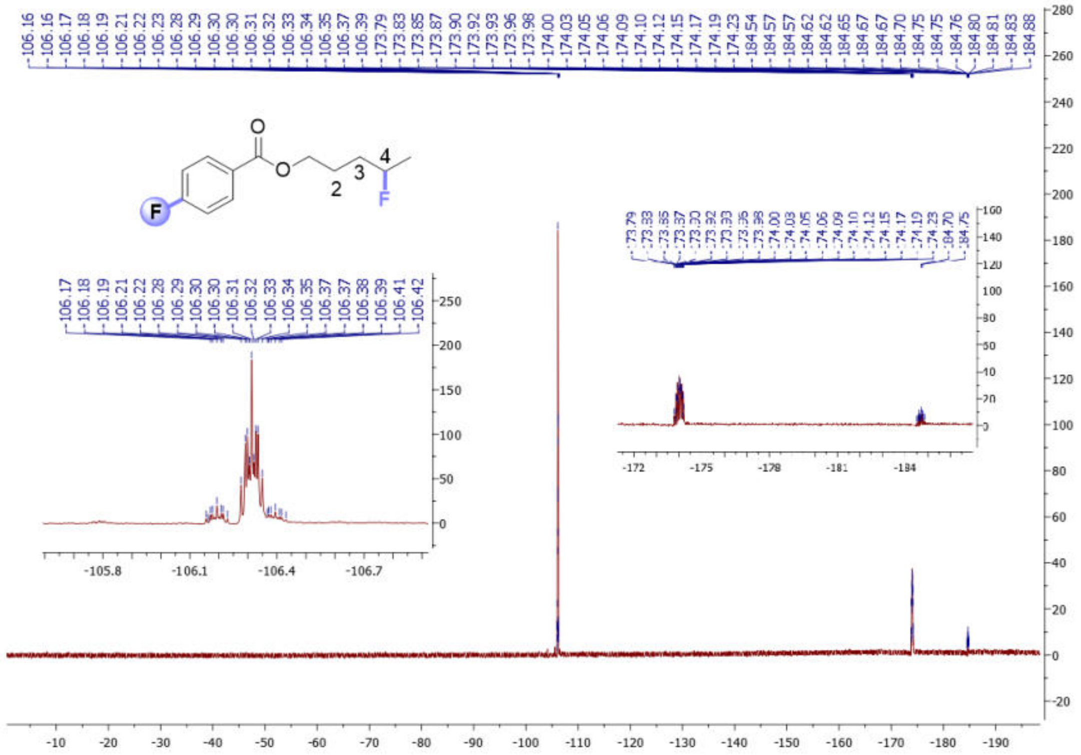

^{1}H NMR of compound **9j-1** in $CDCl_3$

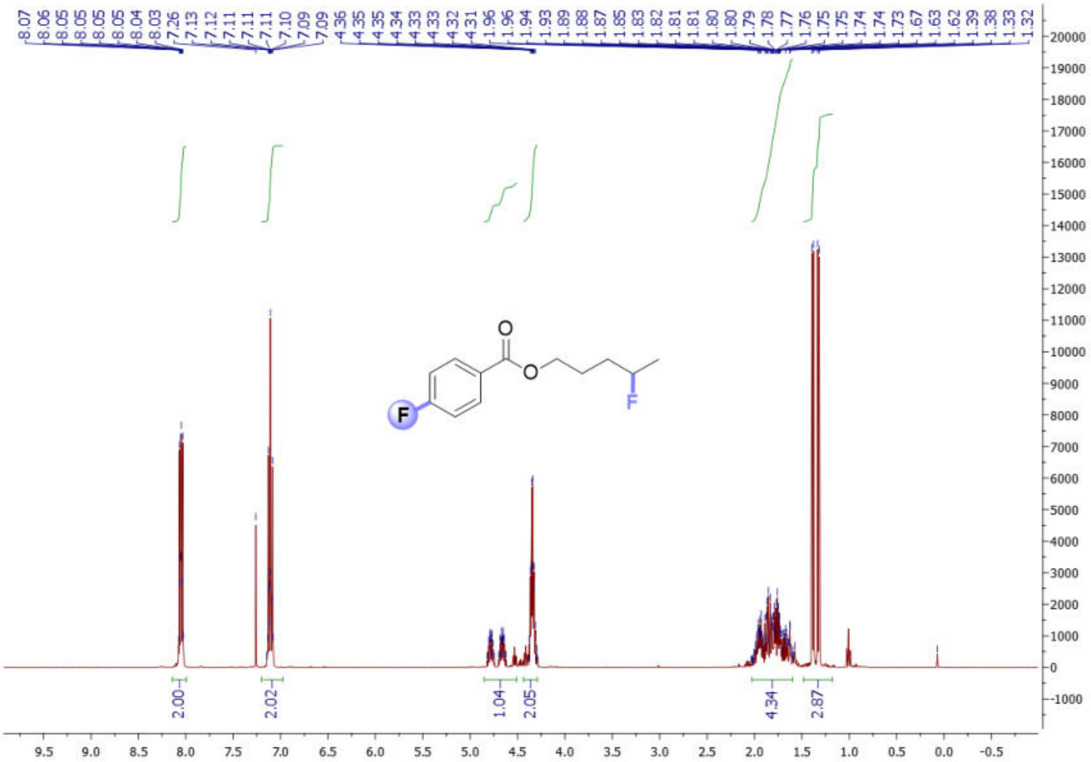

^{13}C NMR of compound **9j-1** in $CDCl_3$

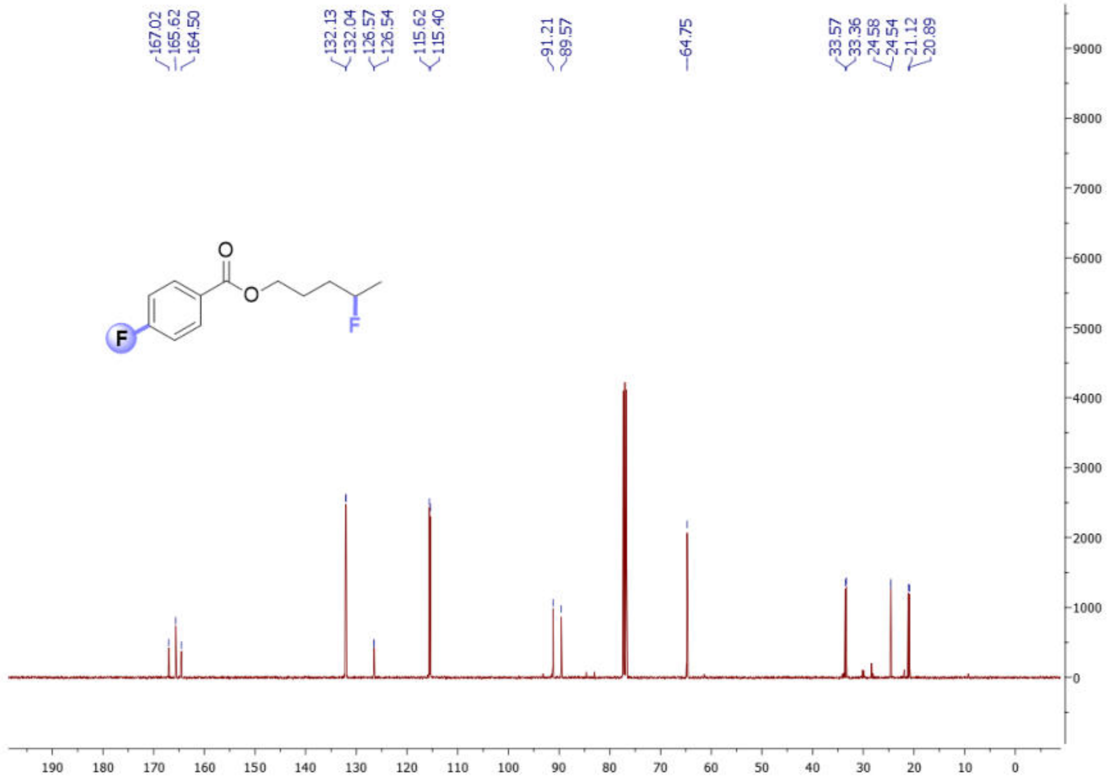

^{19}F NMR of compound **9j-1** in $CDCl_3$

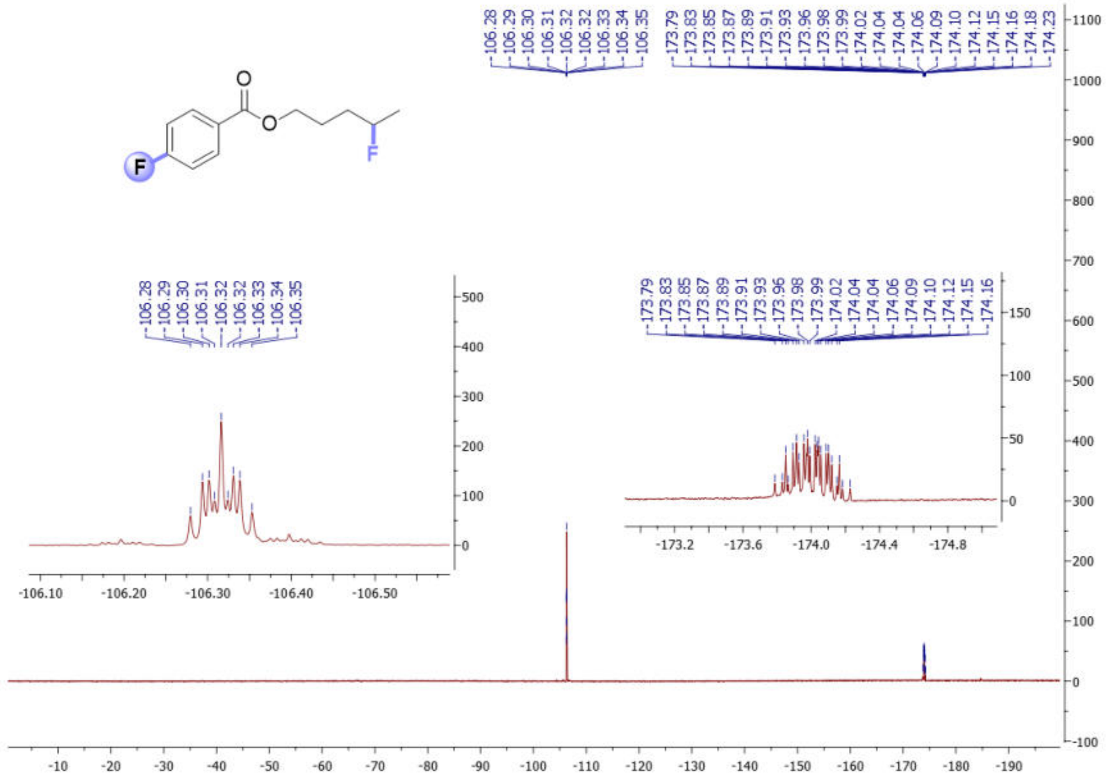

^{1}H NMR of compound **9k** in $CDCl_3$

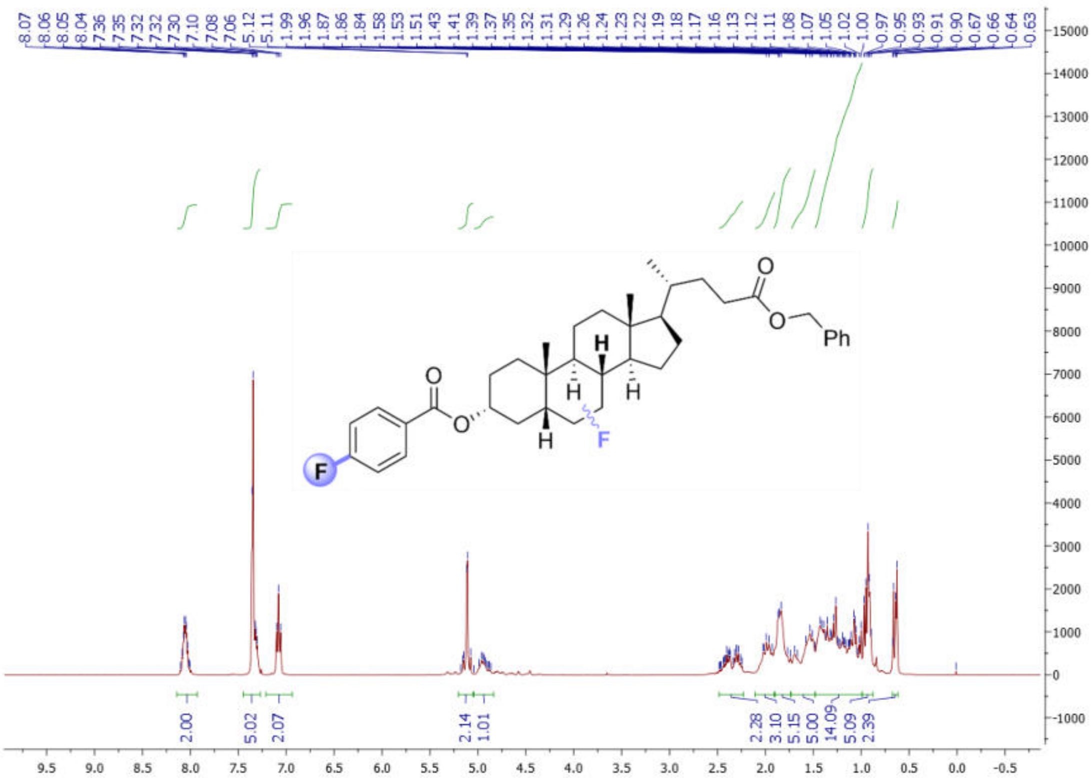

^{13}C NMR of compound **9k** in $CDCl_3$

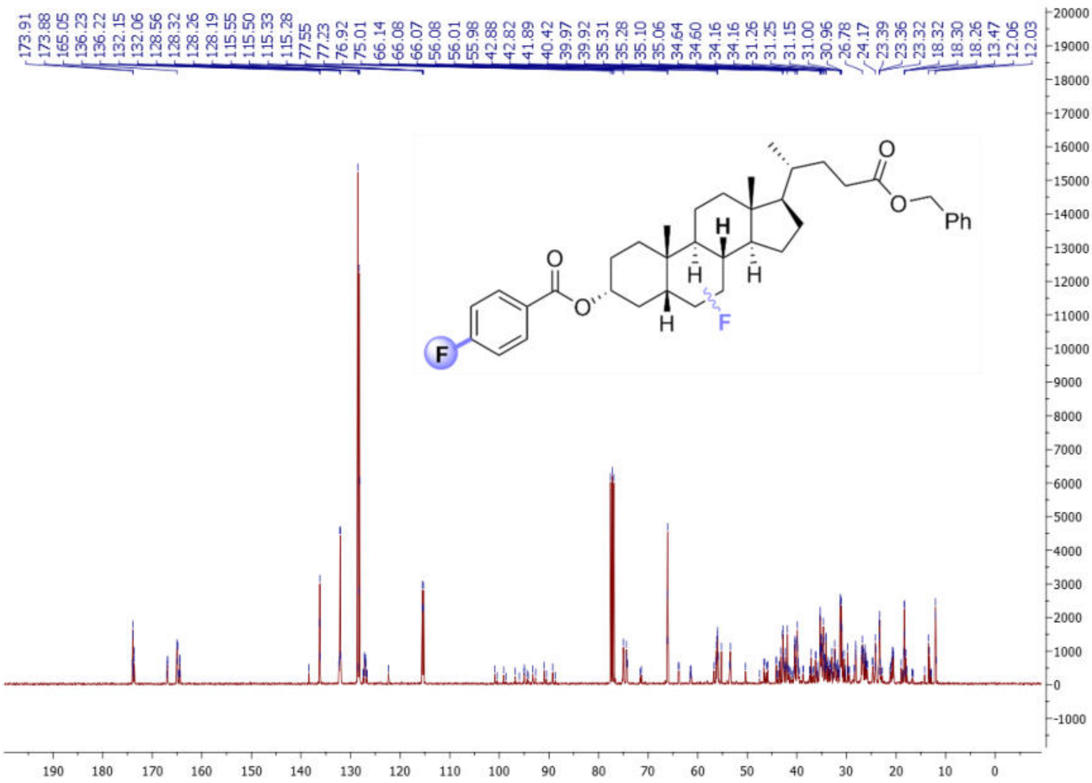

^{19}F{^{1}H} NMR of compound **9k** in $CDCl_3$

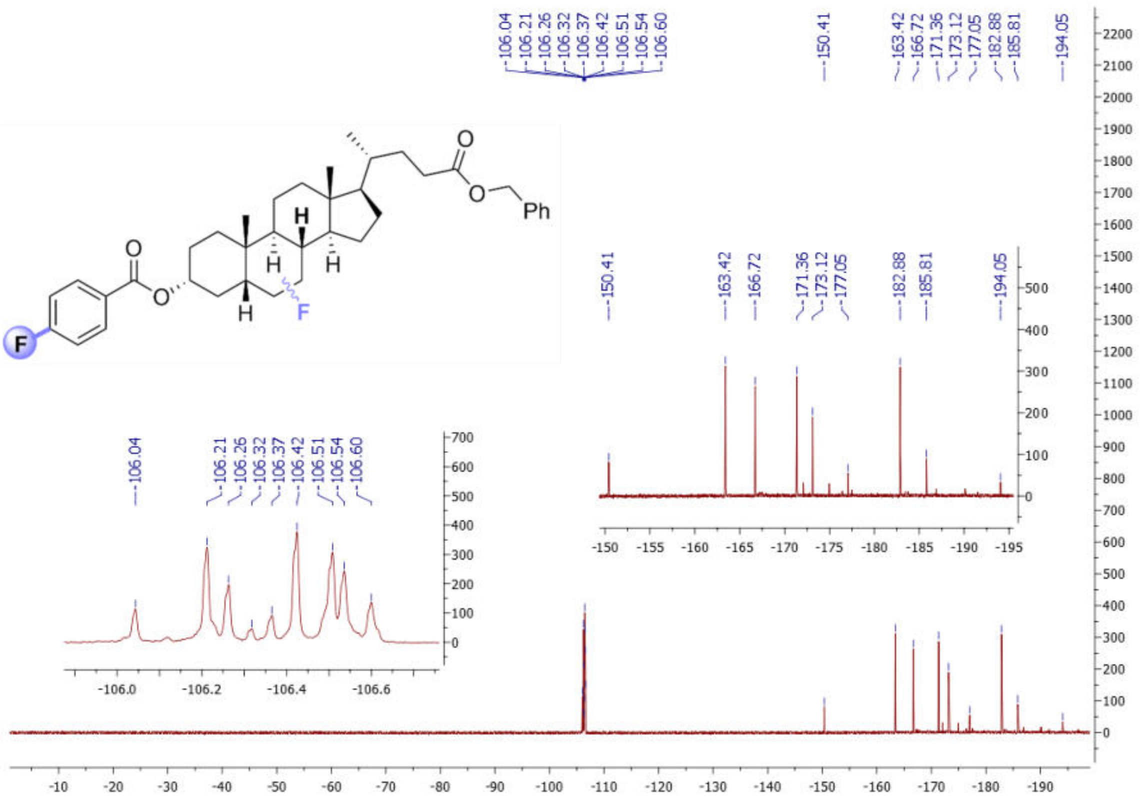

^{1}H NMR of compound **11d-1** in $CDCl_3$

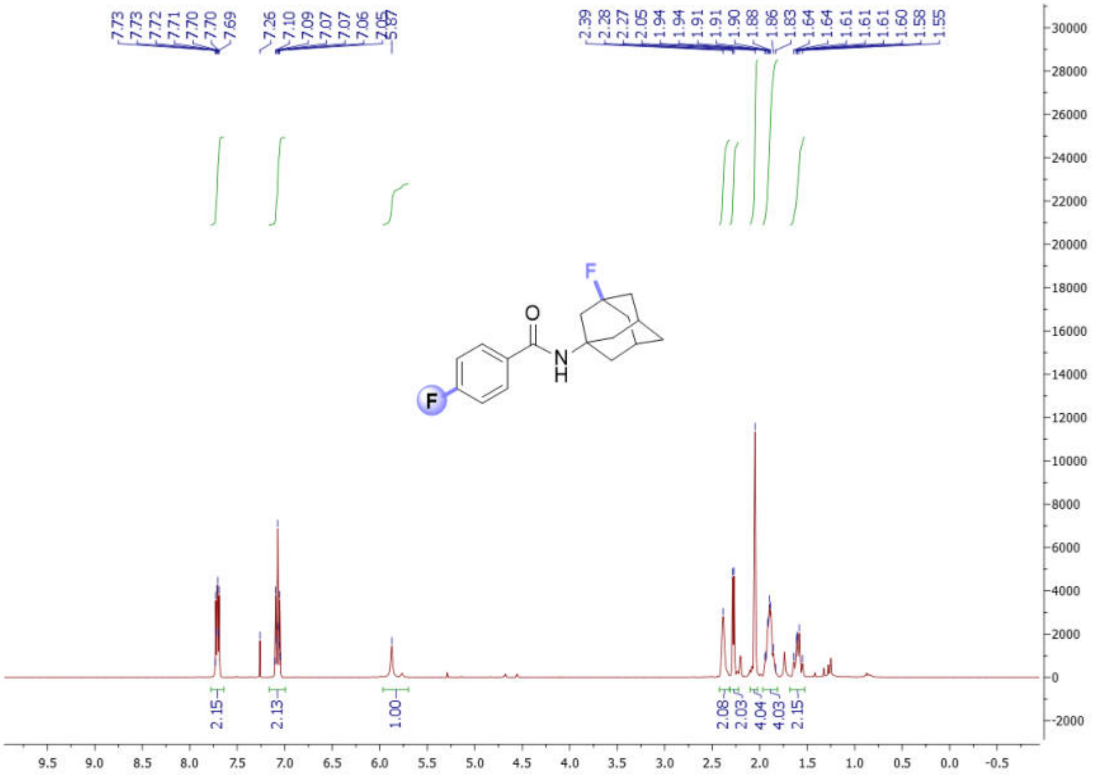

^{13}C NMR of compound **11d-1** in $CDCl_3$

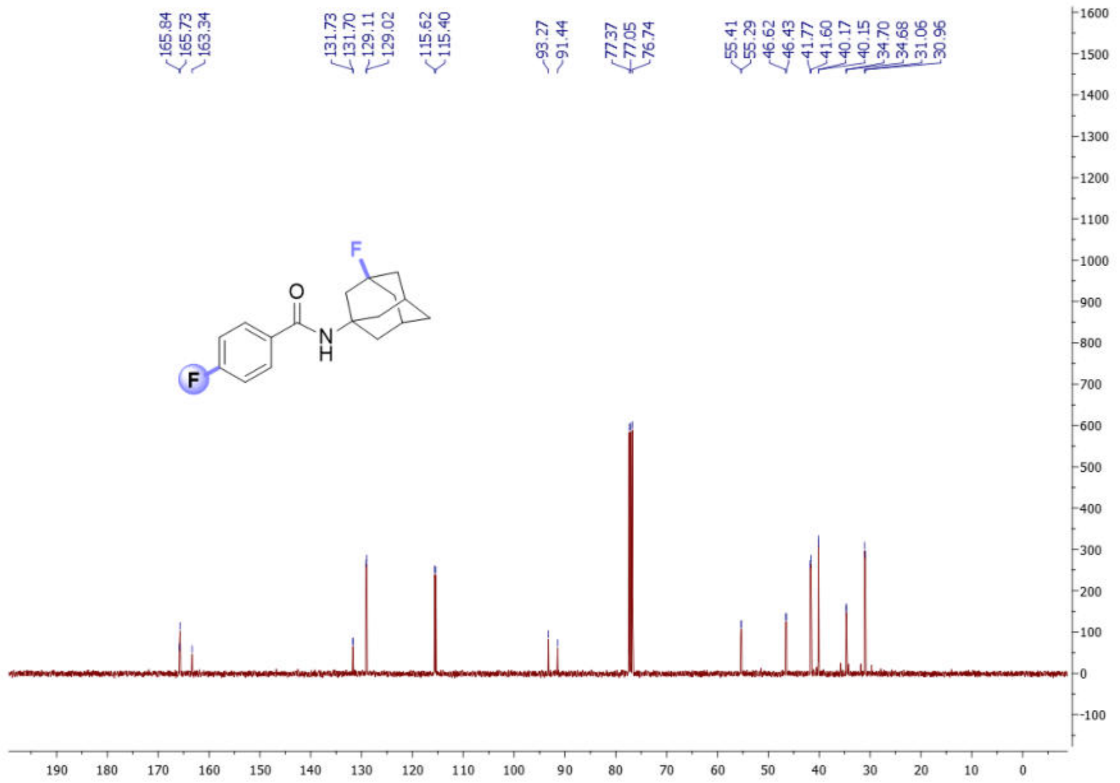

^{19}F NMR of compound **11d-1** in $CDCl_3$

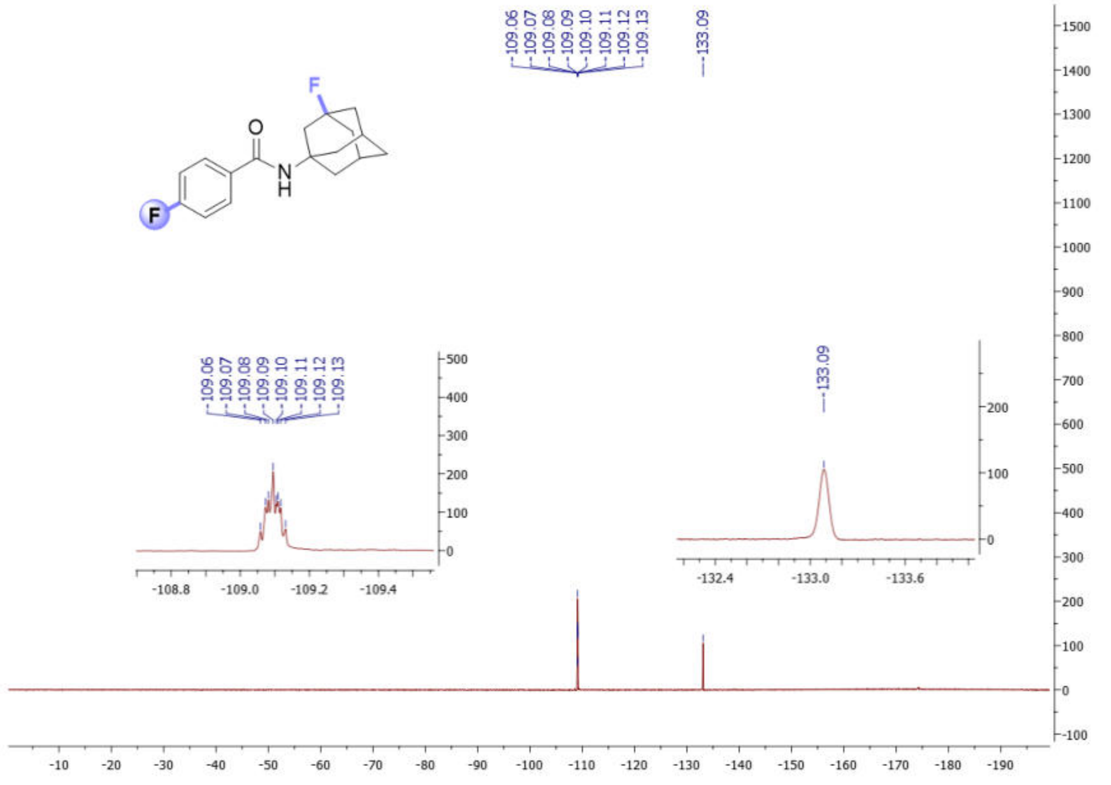

^{1}H NMR of compound **11d-2** in $CDCl_3$

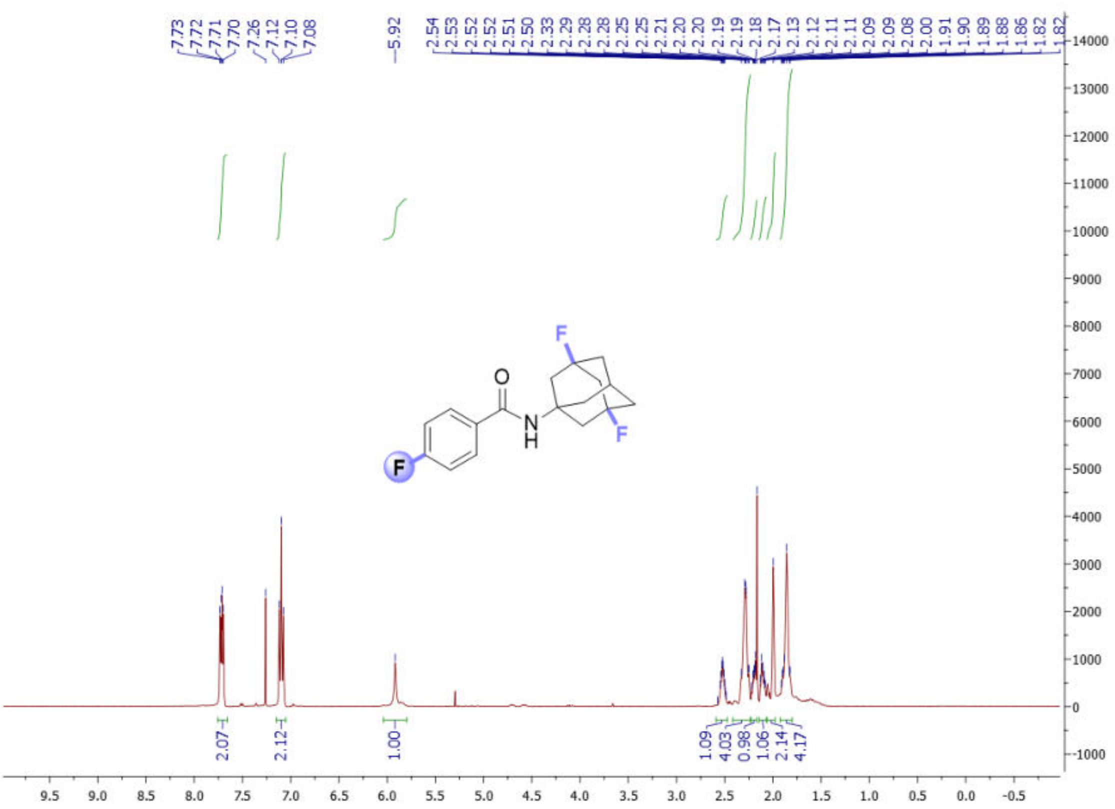

^{13}C NMR of compound **11d-2** in $CDCl_3$

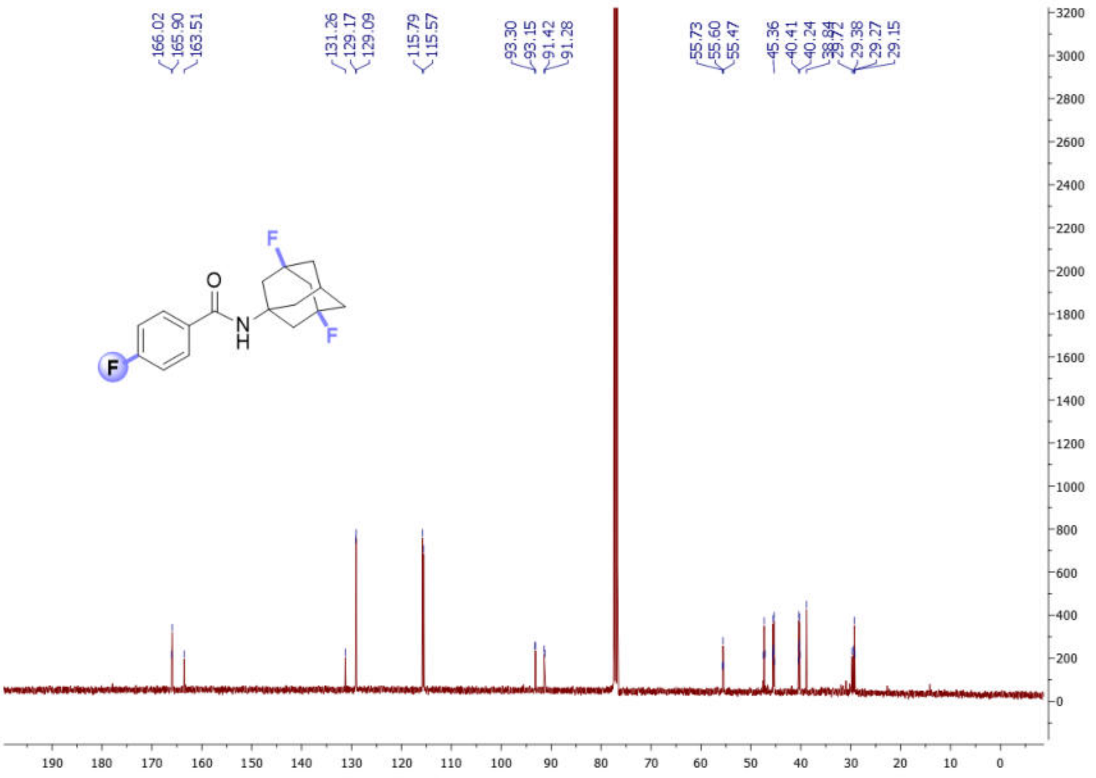

^{19}F NMR of compound **11d-2** in $CDCl_3$

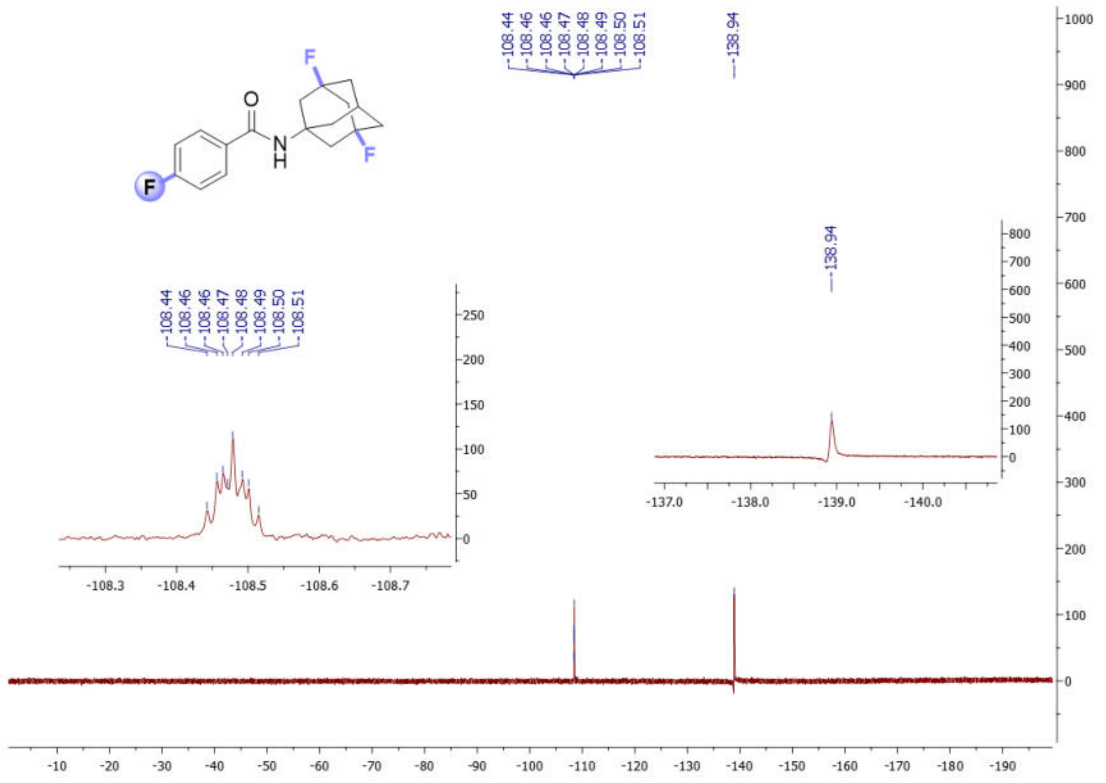

^{1}H NMR of compound **11a** in $CDCl_3$

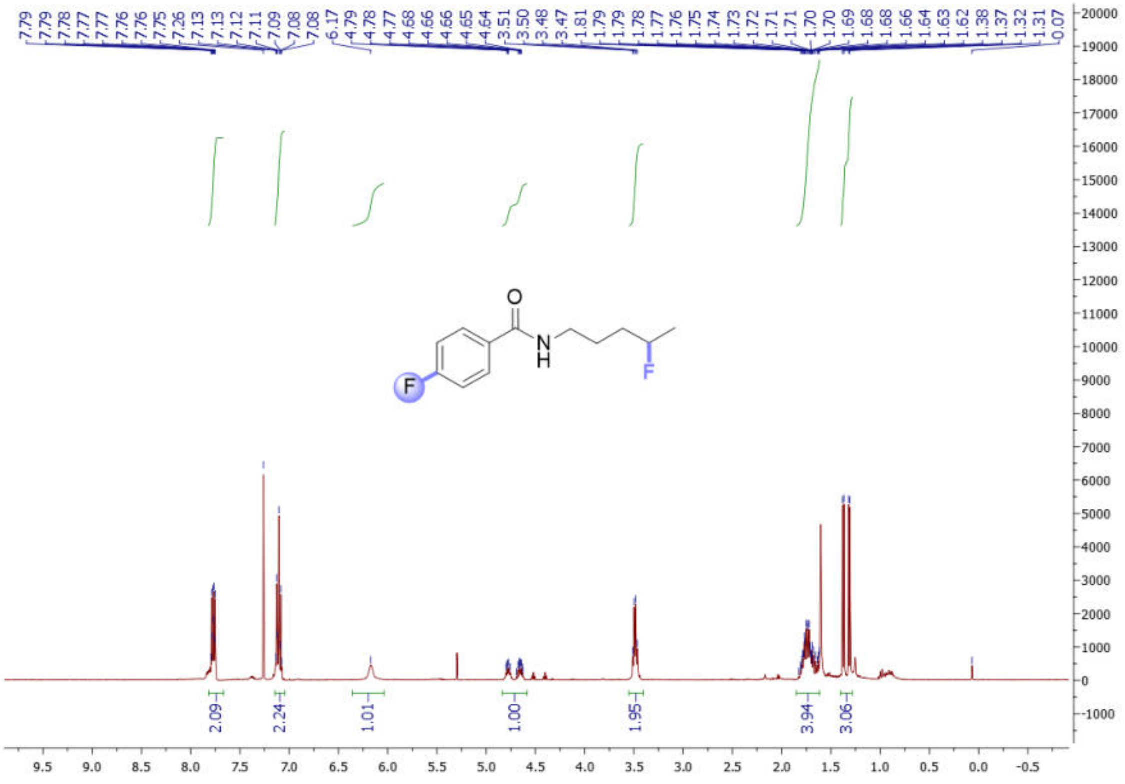

^{13}C NMR of compound **11a** in CDCl$_3$

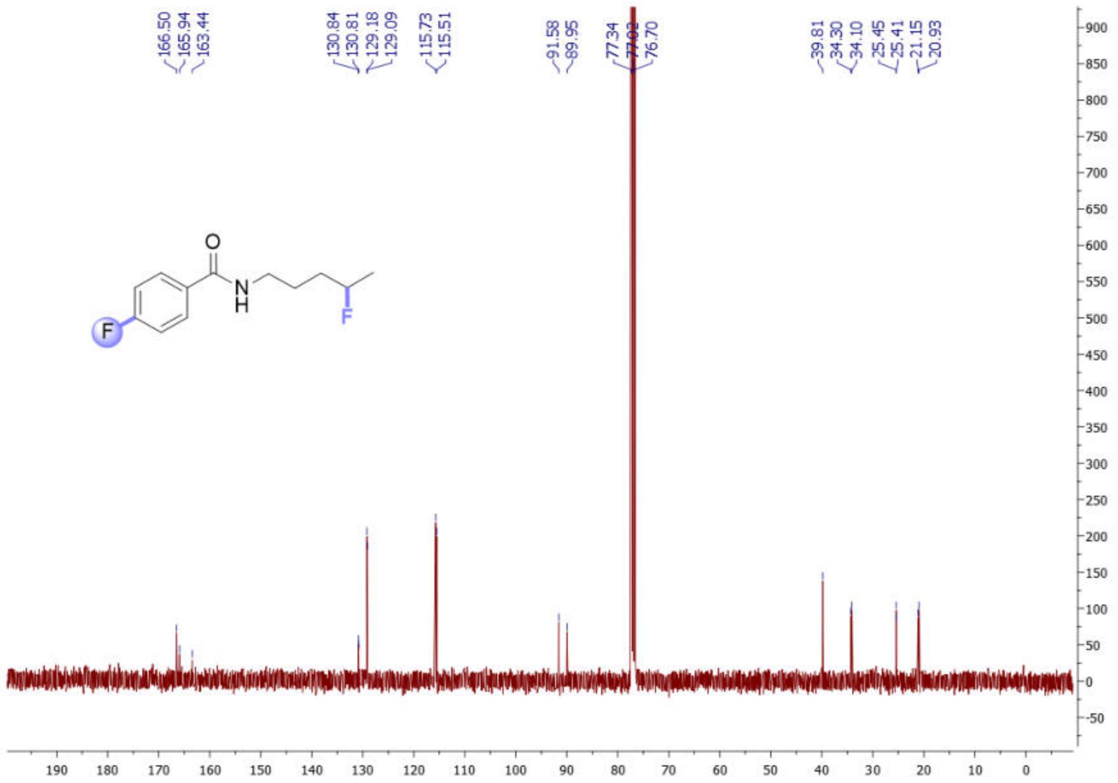

$^{19}F\{^1H\}$ NMR of compound **11a** in CDCl$_3$

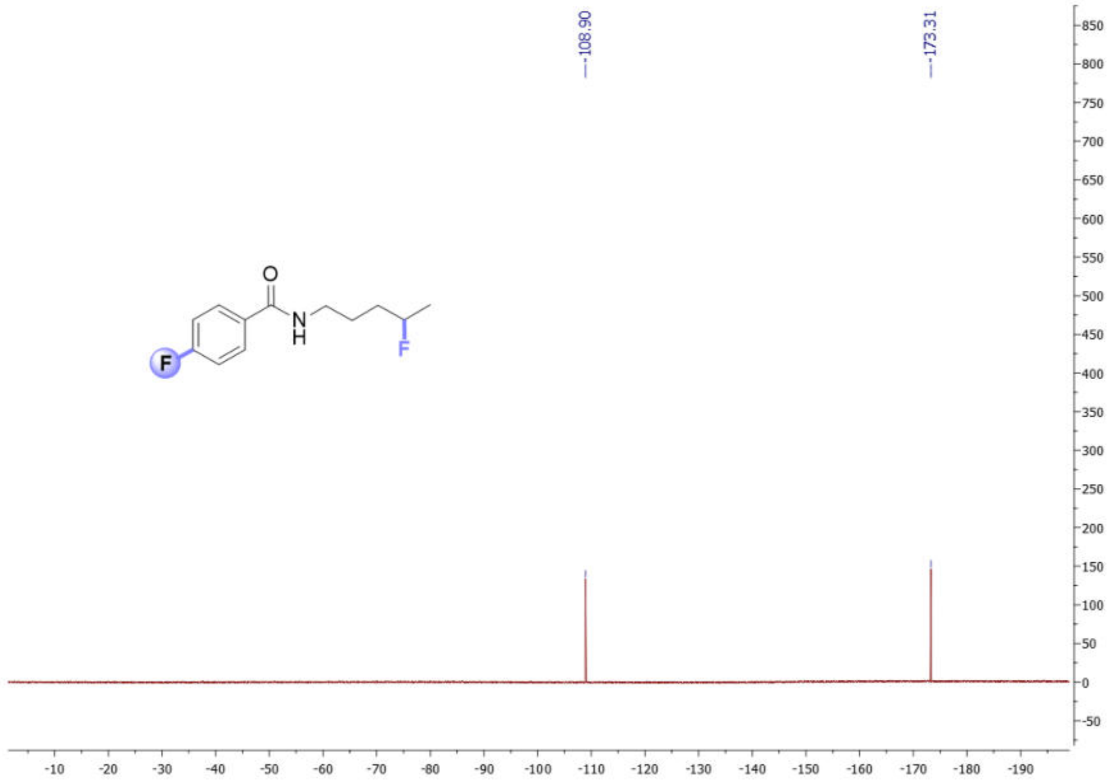

^{1}H NMR of compound **11b** in $CDCl_3$

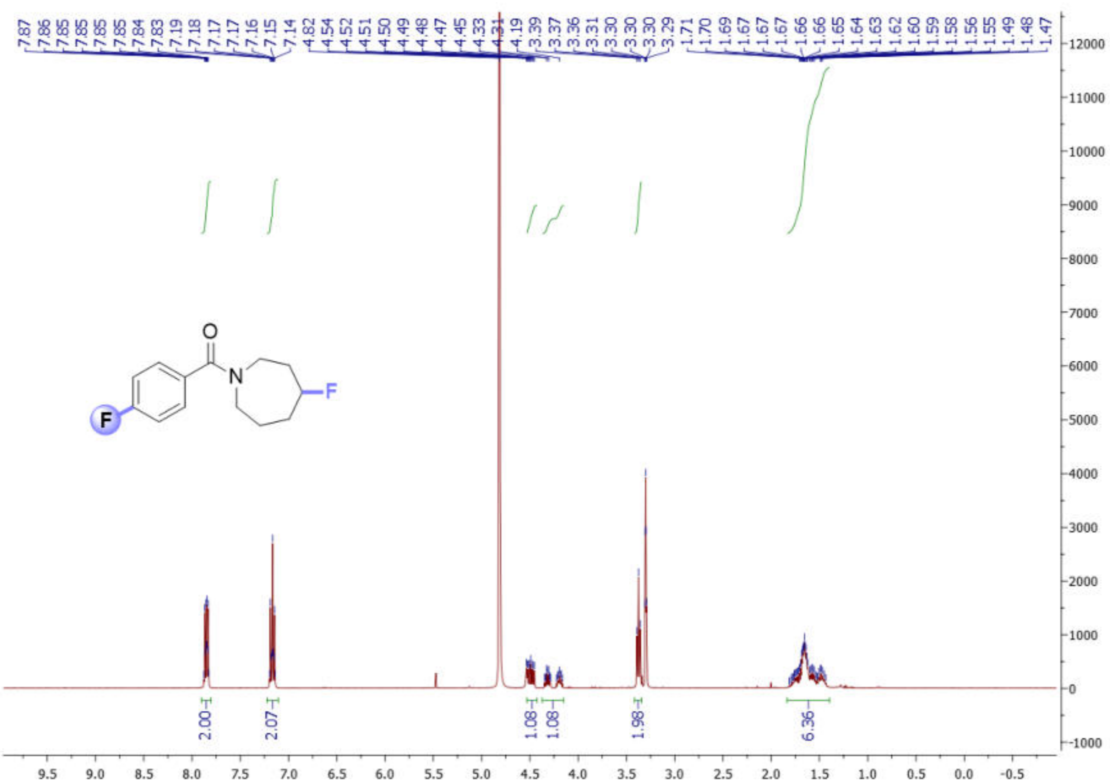

^{13}C NMR of compound **11b** in $CDCl_3$

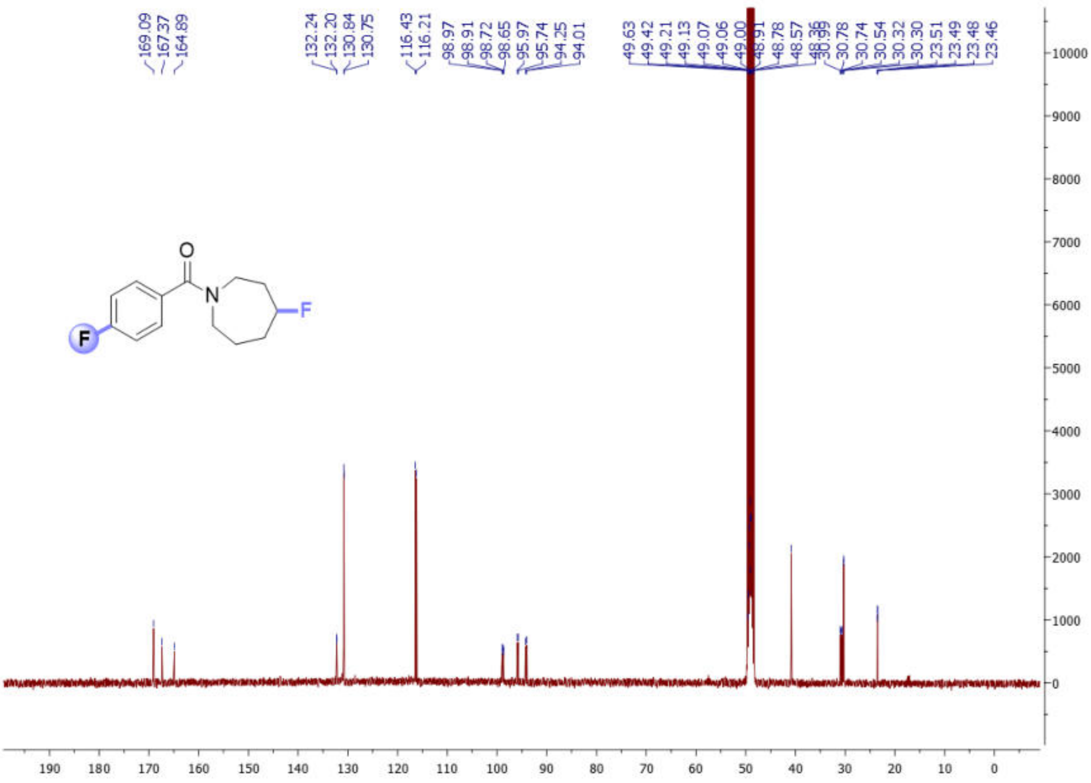

^{19}F NMR of compound **11b** in $CDCl_3$

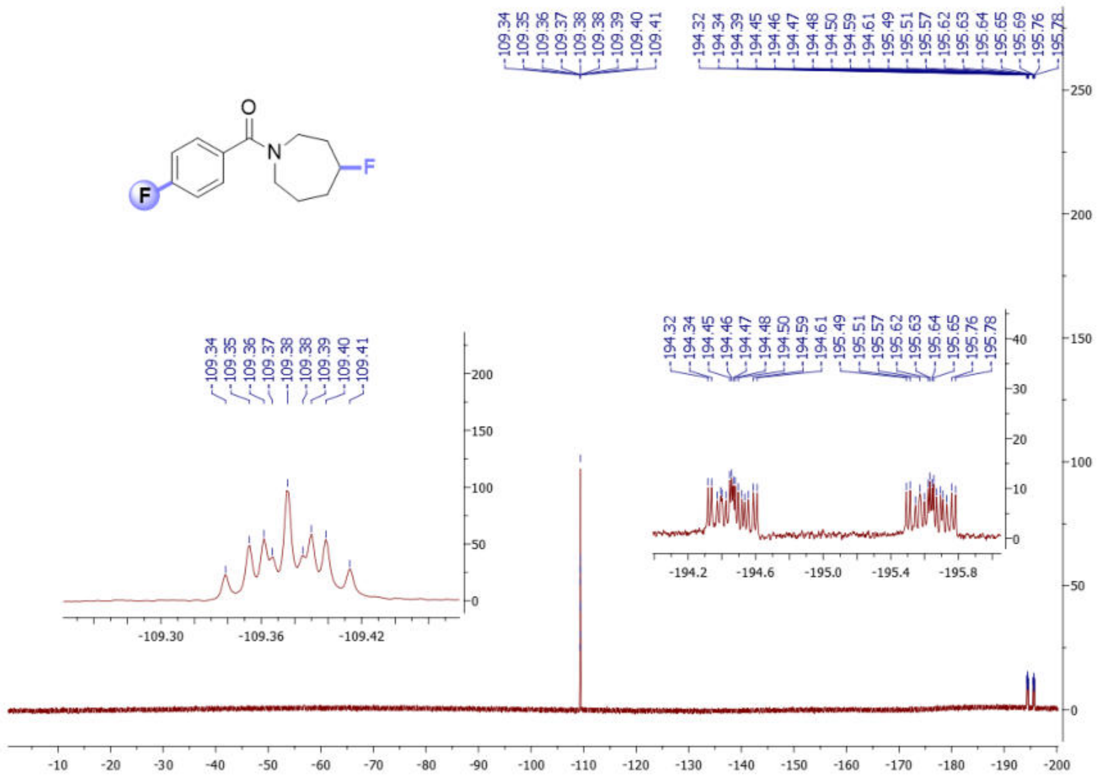

^{19}F NMR{^{1}H} of compound **11b** in $CDCl_3$

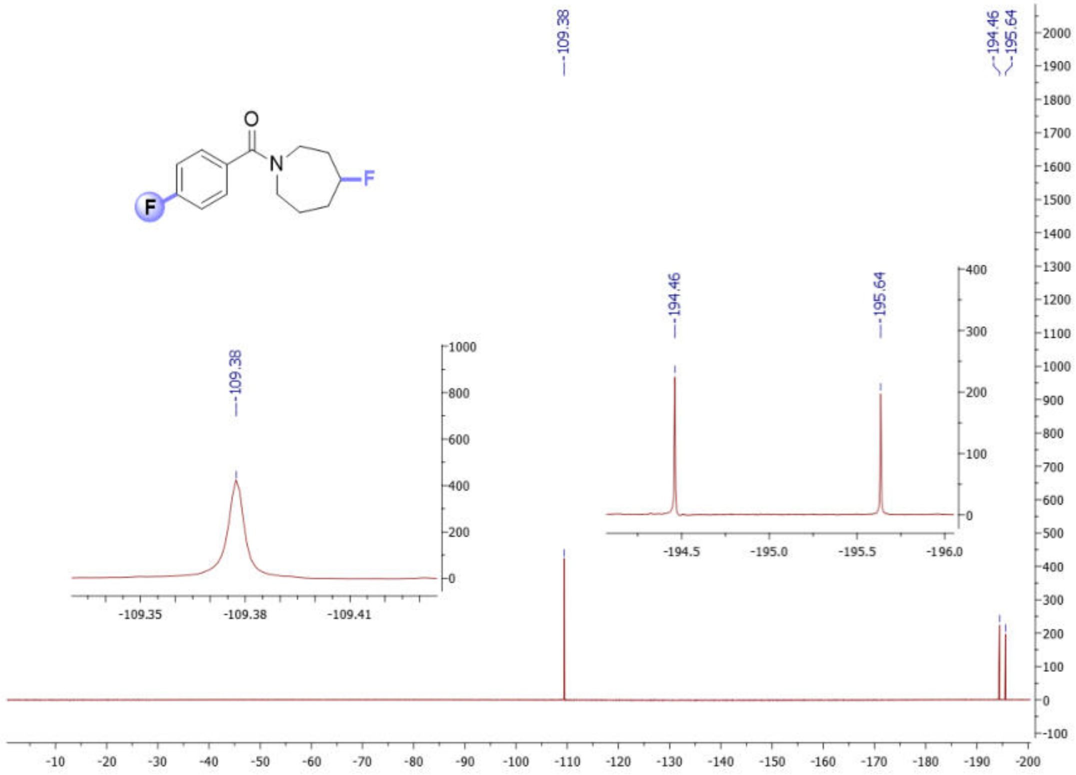

^{1}H NMR of compound **11c** in $CDCl_3$

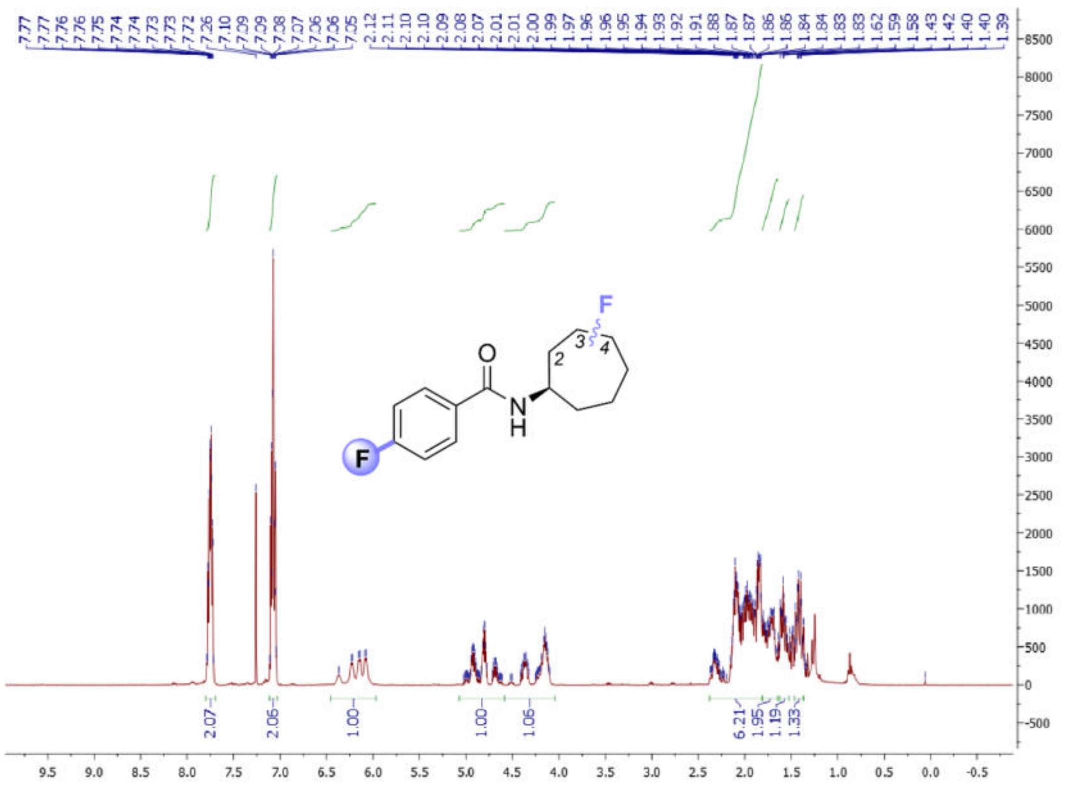

^{13}C NMR of compound **11c** in $CDCl_3$

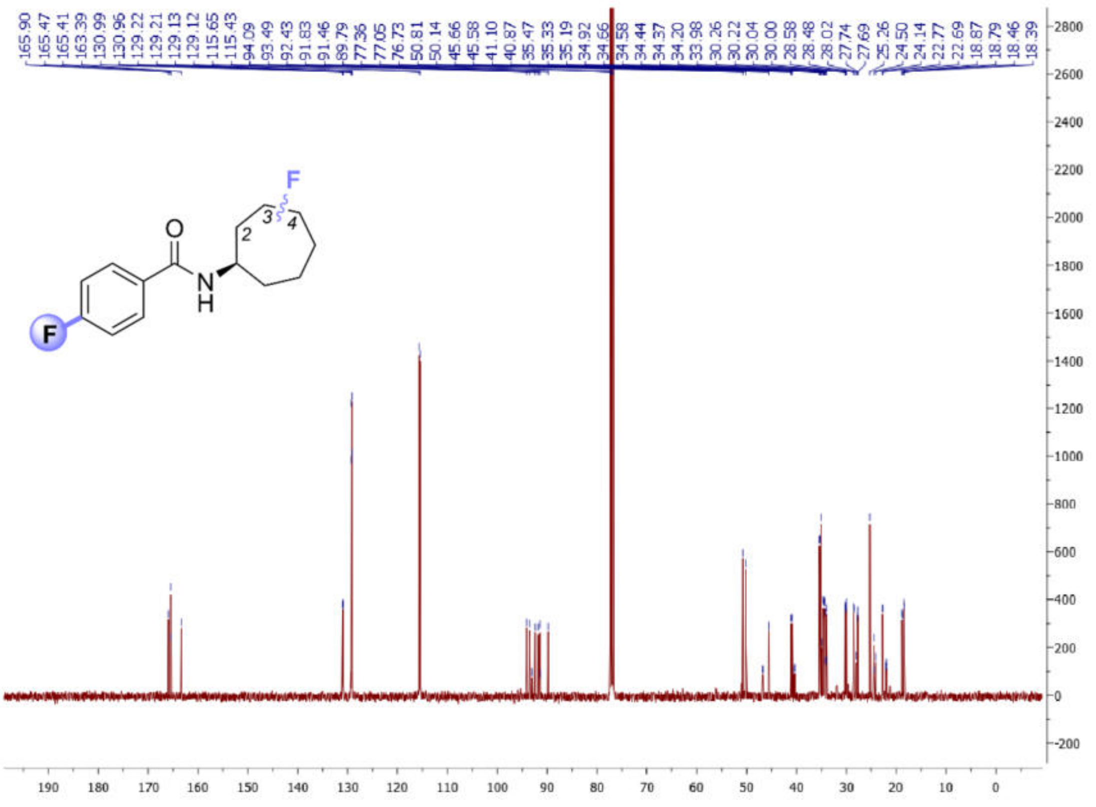

^{19}F NMR of compound **11c** in $CDCl_3$

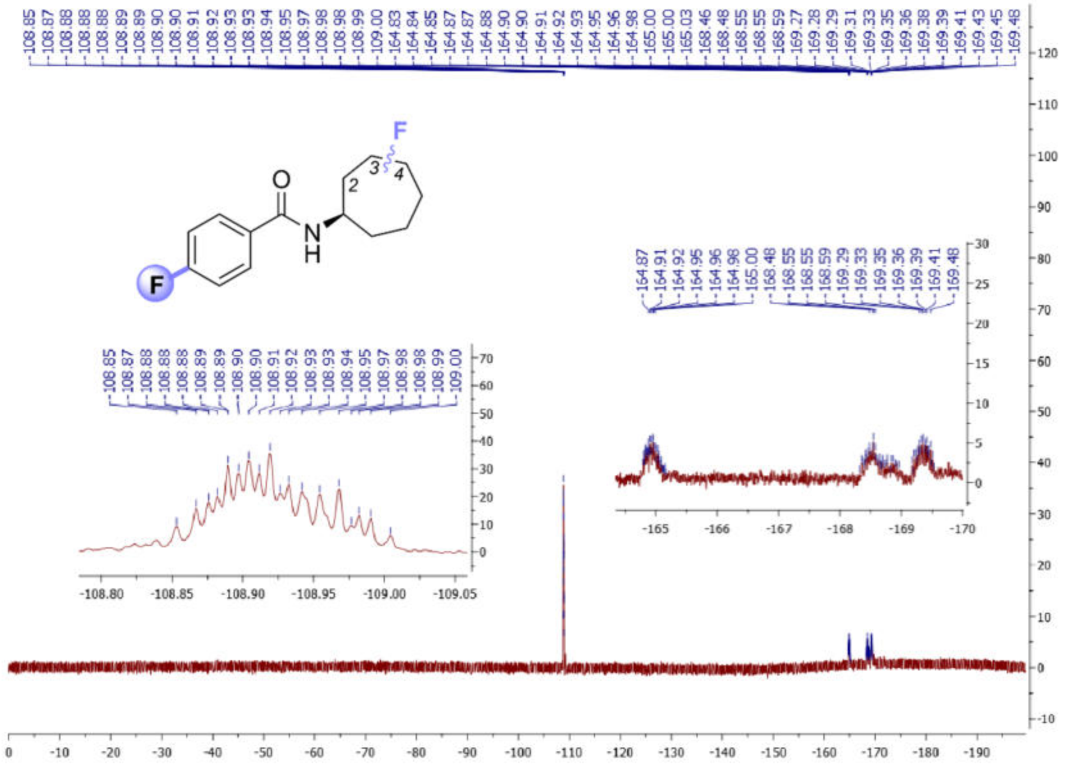

COSY NMR of compound **11c** in $CDCl_3$

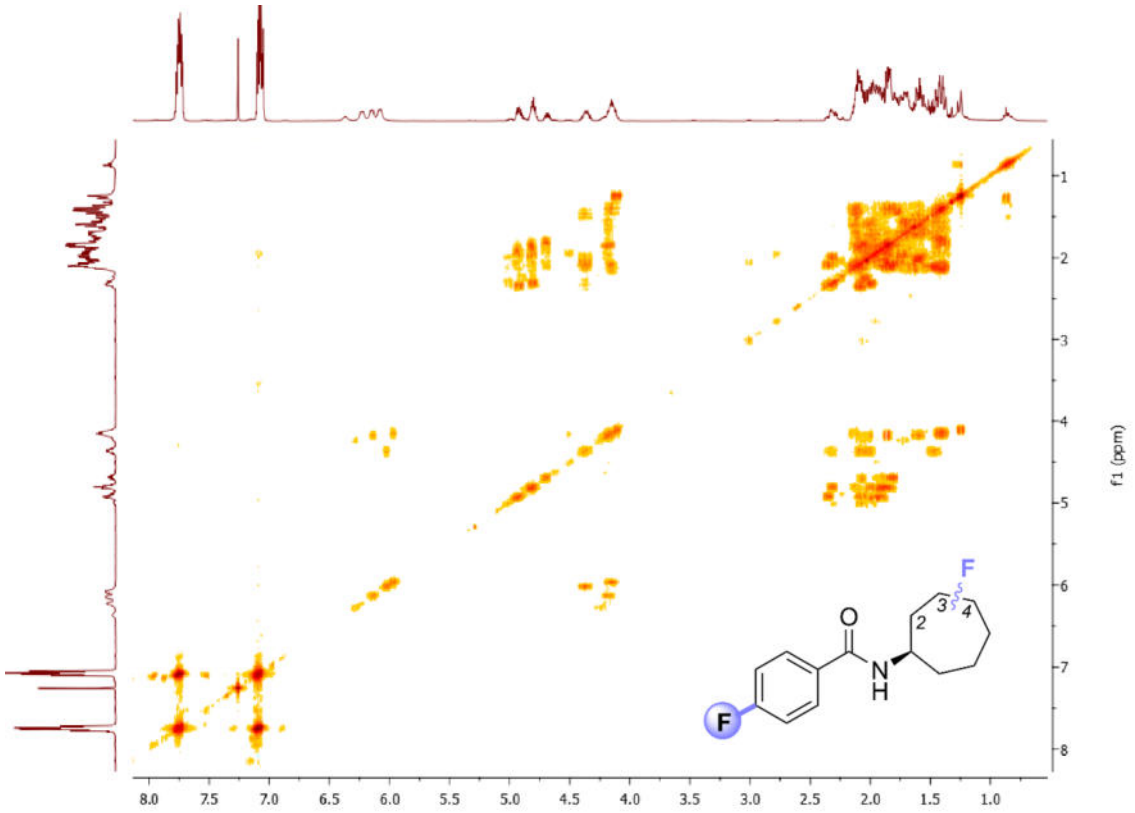

TOCSY NMR of compound **11c** in $CDCl_3$

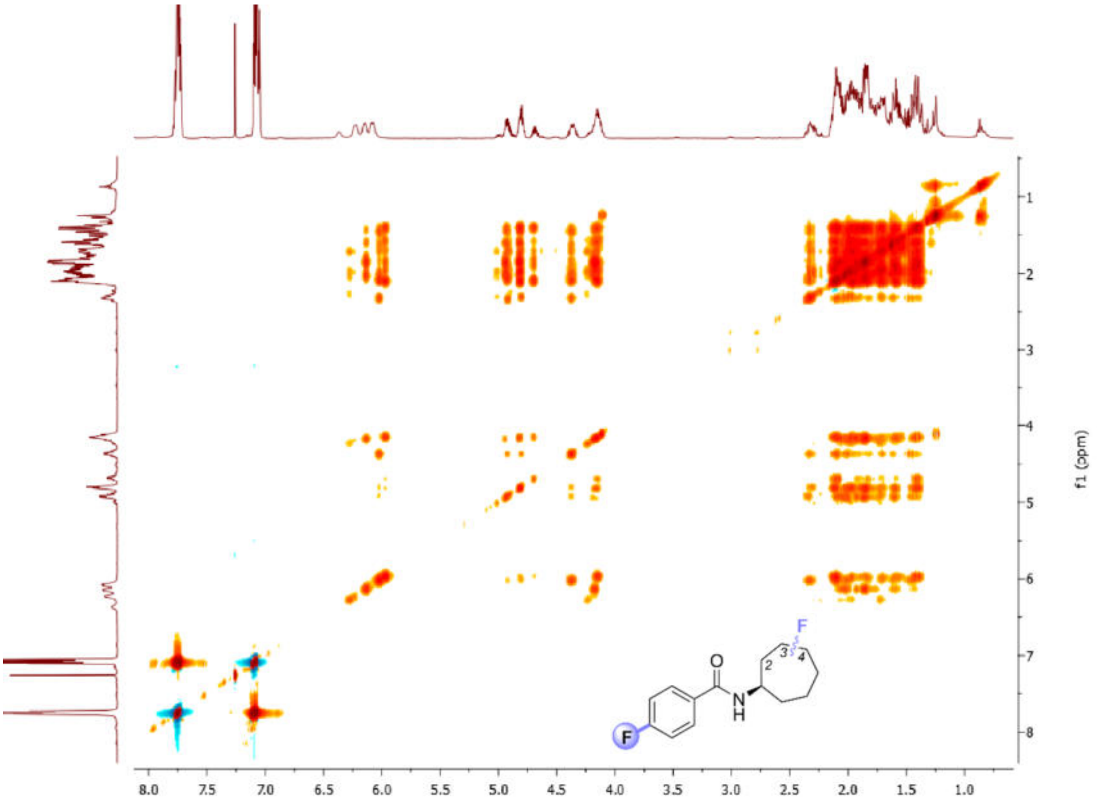

HSQC NMR of compound **11c** in $CDCl_3$

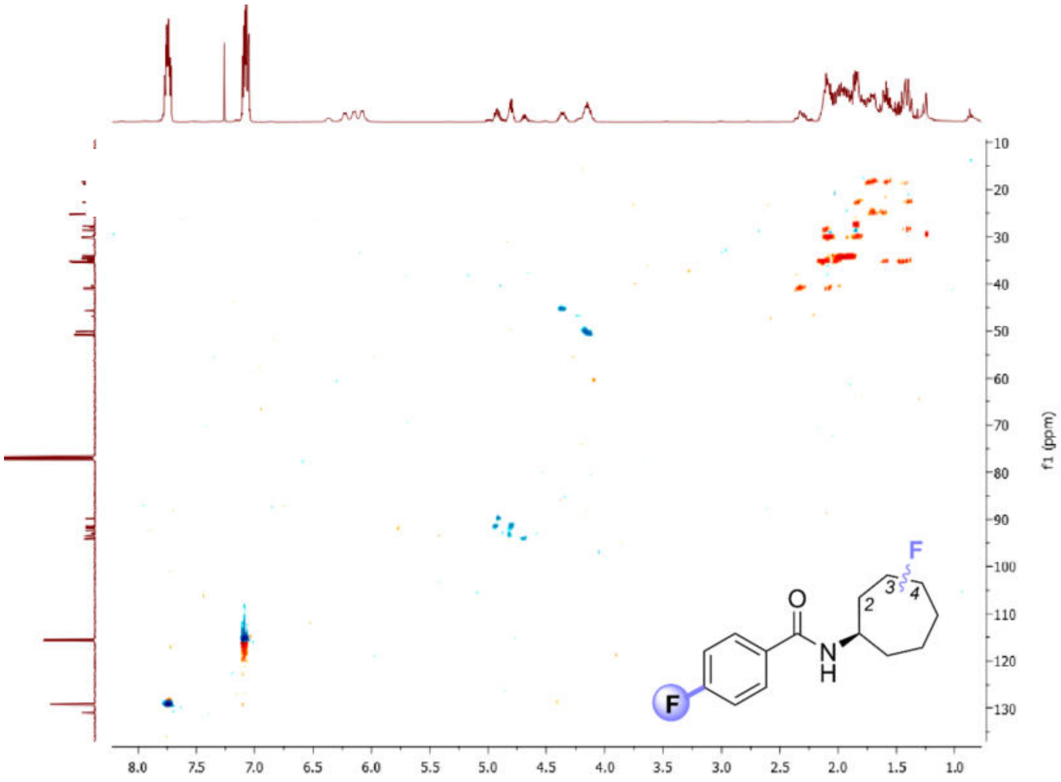

HMBC NMR of compound **11c** in $CDCl_3$

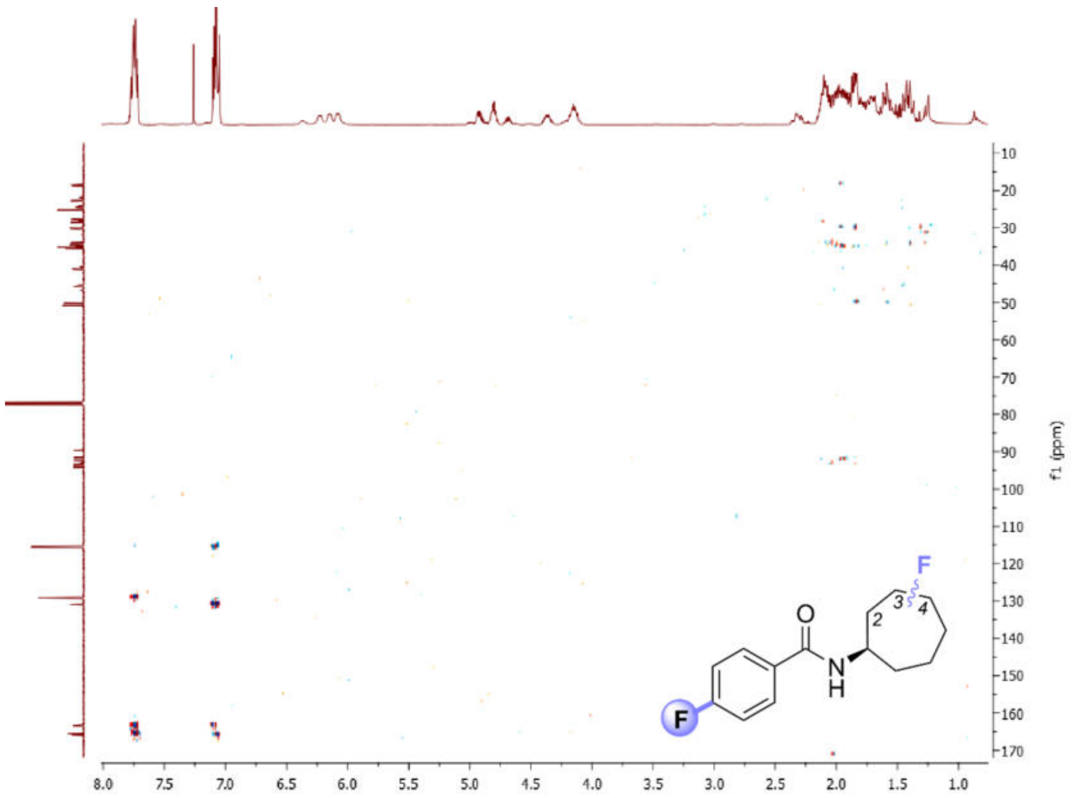

^{1}H NMR of compound **11e** in $CDCl_3$

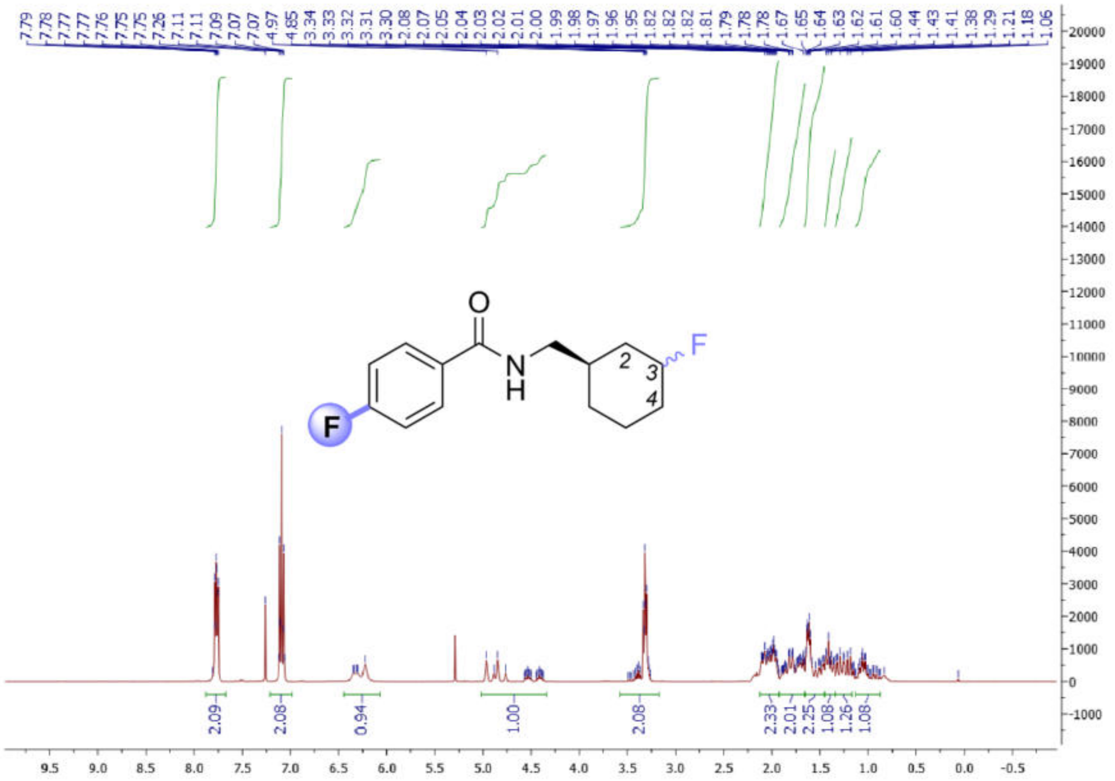

^{13}C NMR of compound **11e** in $CDCl_3$

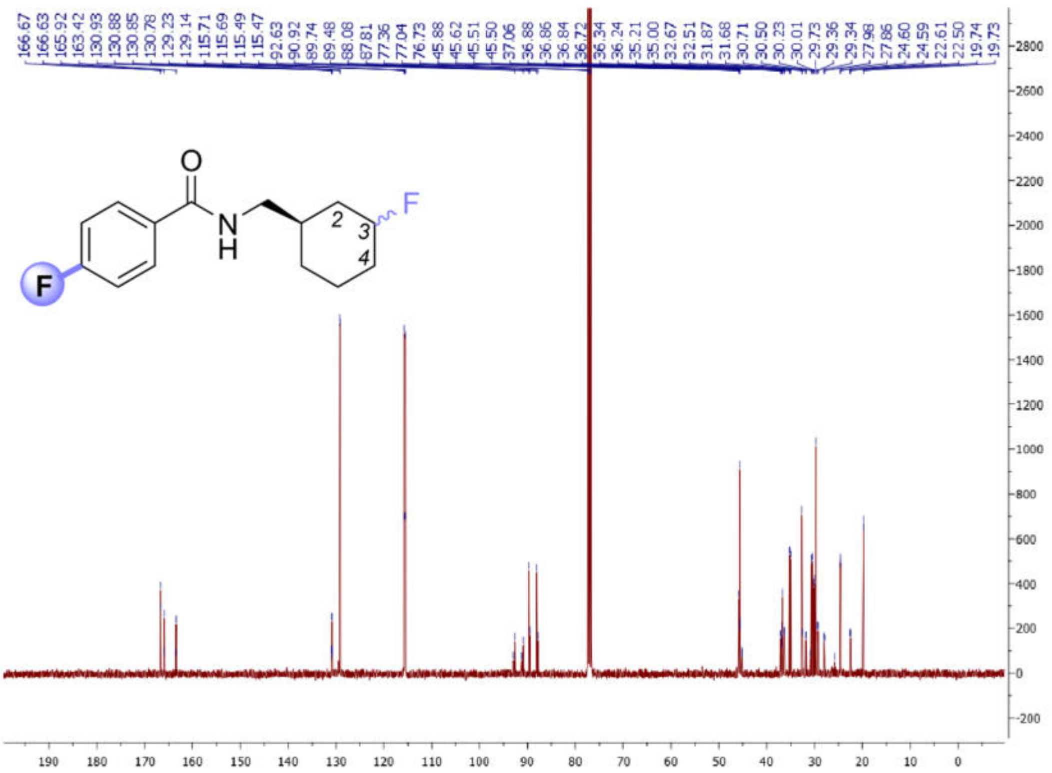

^{19}F NMR of compound **11e** in $CDCl_3$

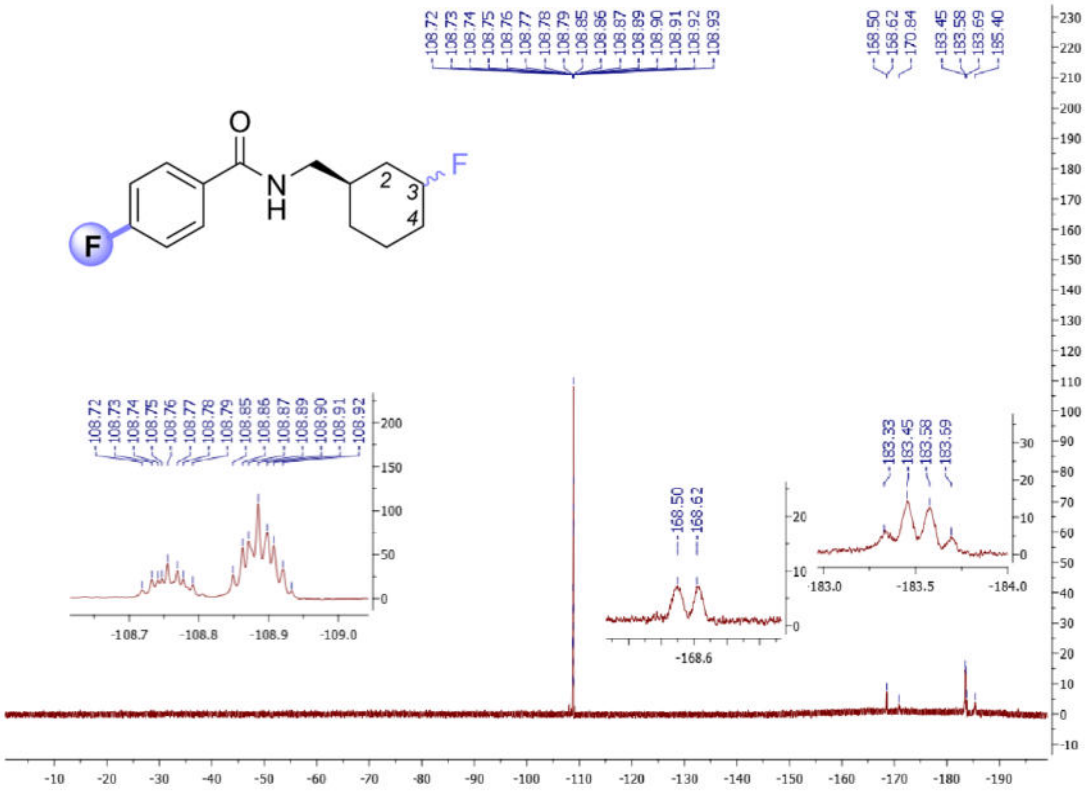

COSY NMR of compound **11e** in $CDCl_3$

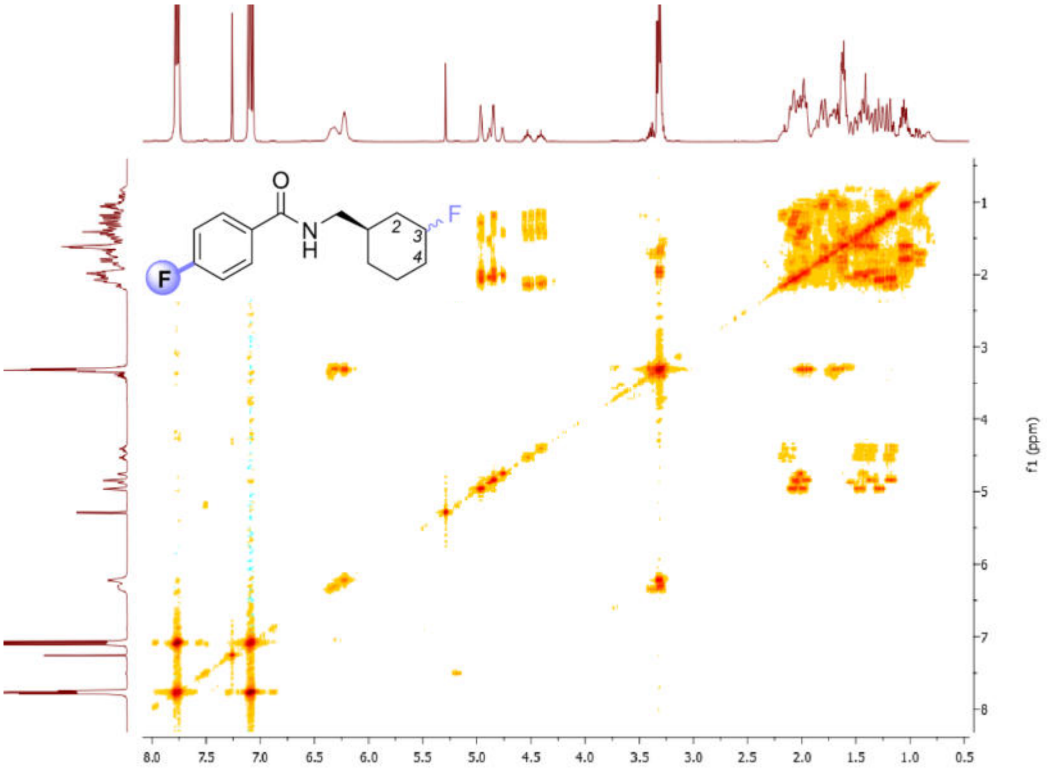

TOCSY NMR of compound **11e** in $CDCl_3$

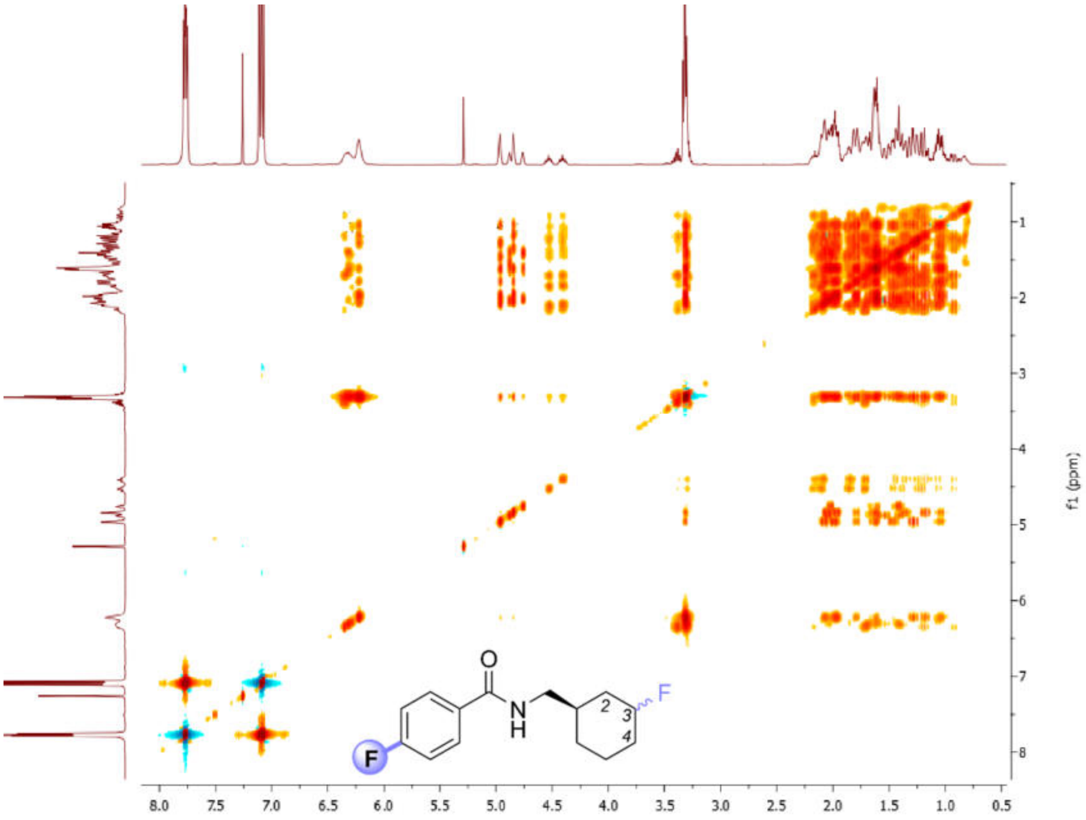

HSQC NMR of compound **11e** in CDCl3

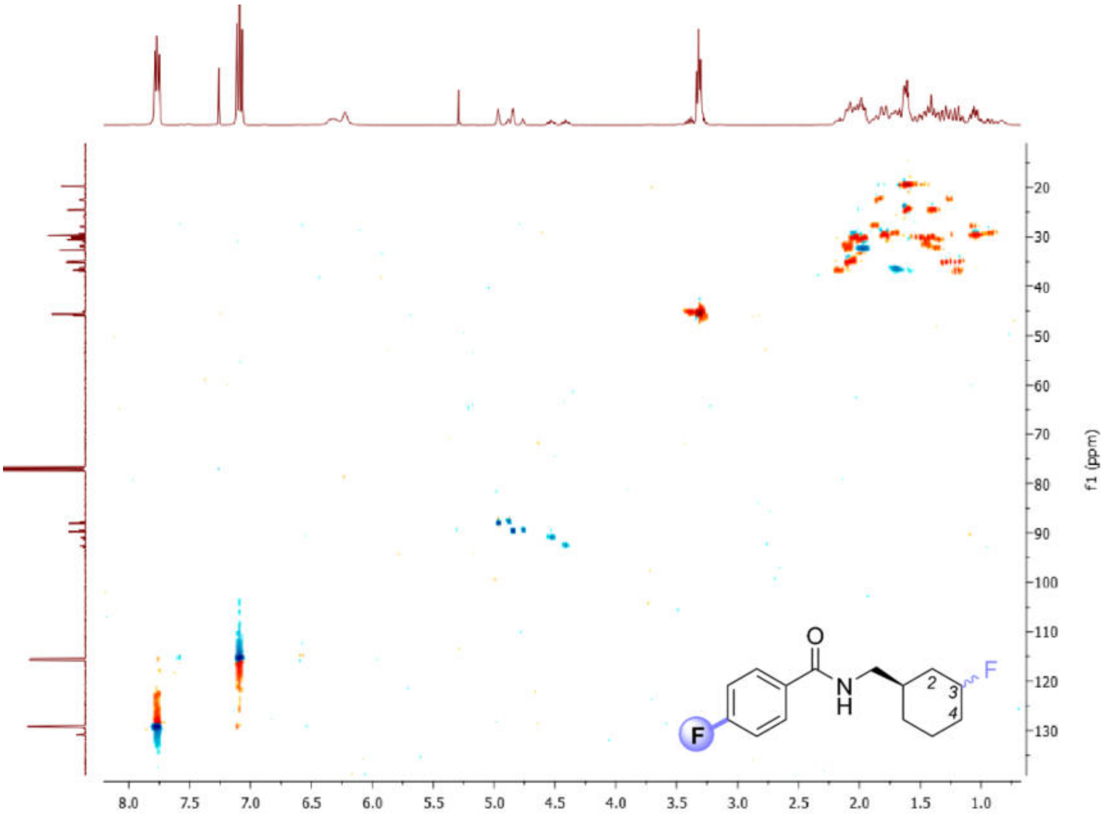

HMBC NMR of compound **11e** in CDCl3

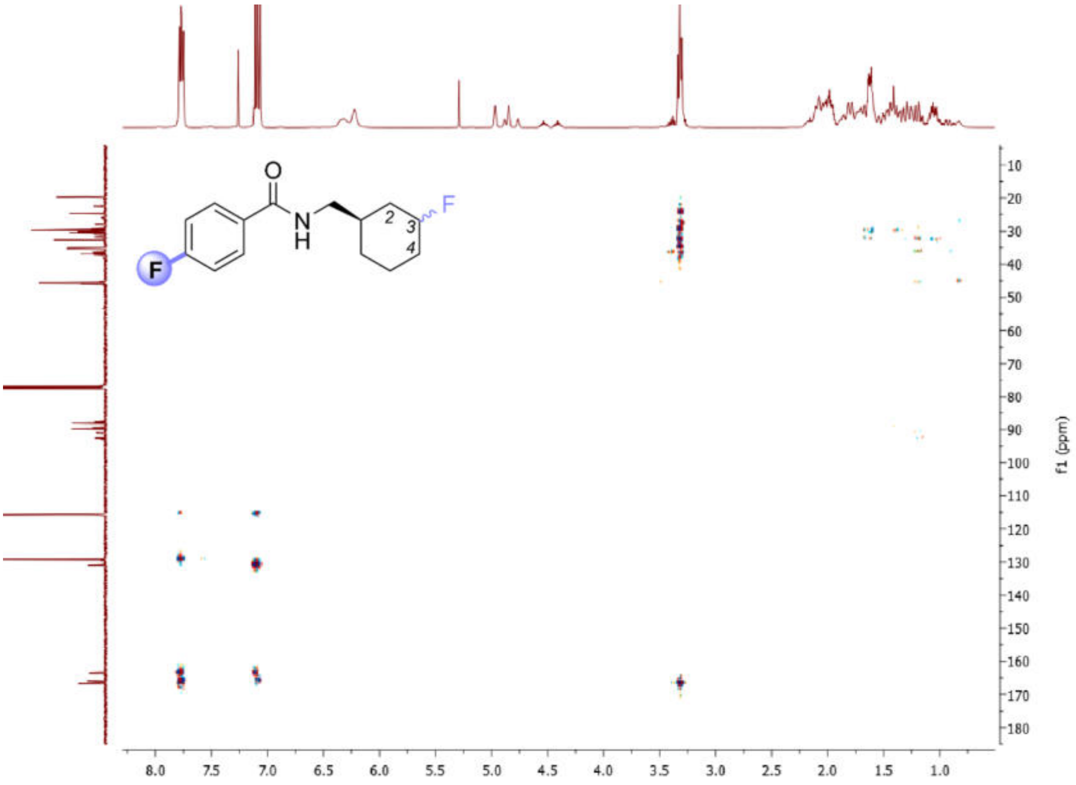

^{1}H NMR of compound **11g** in $CDCl_3$

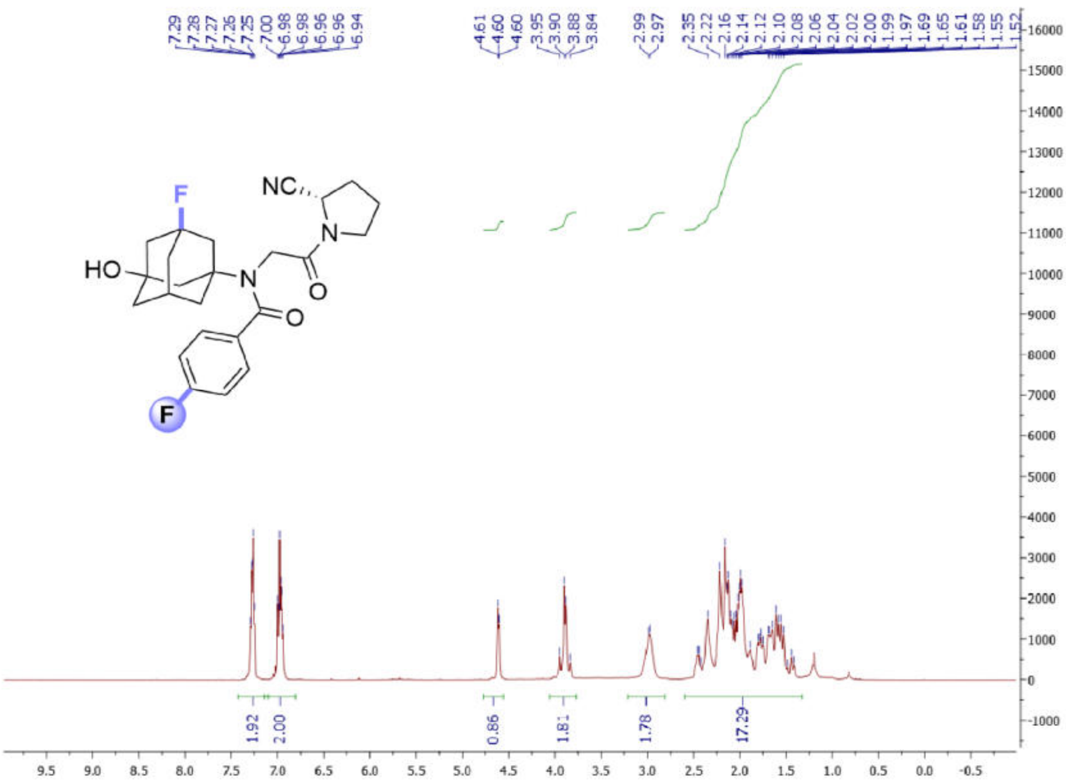

^{13}C NMR of compound **11g** in $CDCl_3$

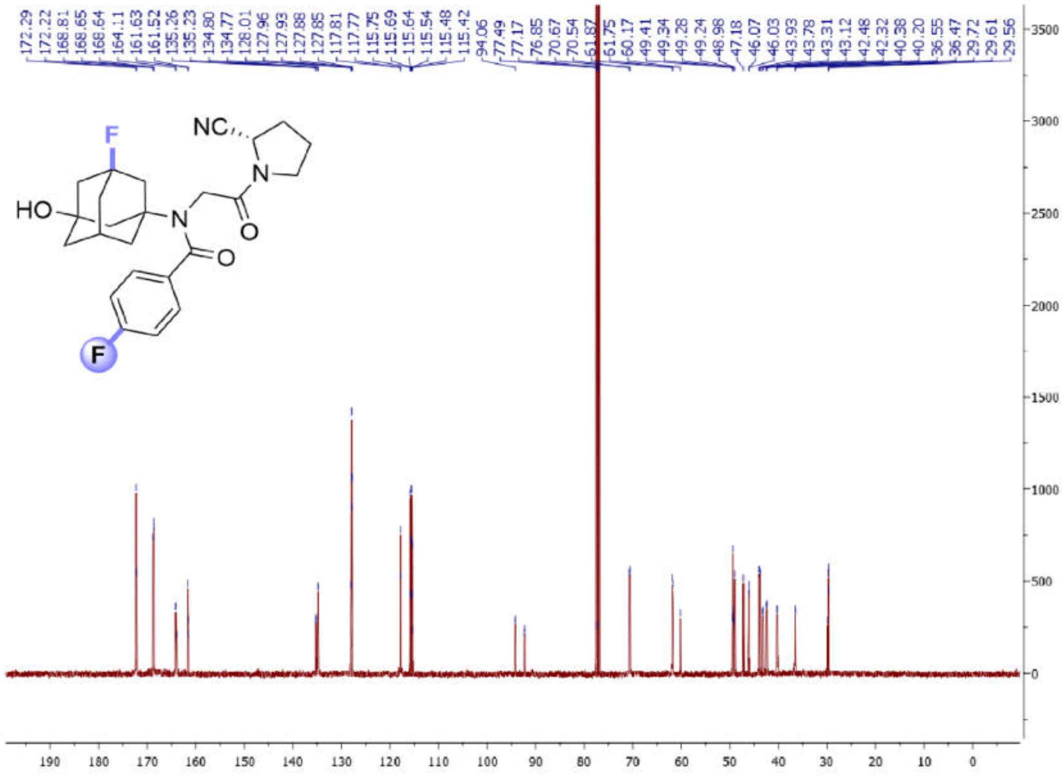

^{19}F NMR of compound **11g** in $CDCl_3$

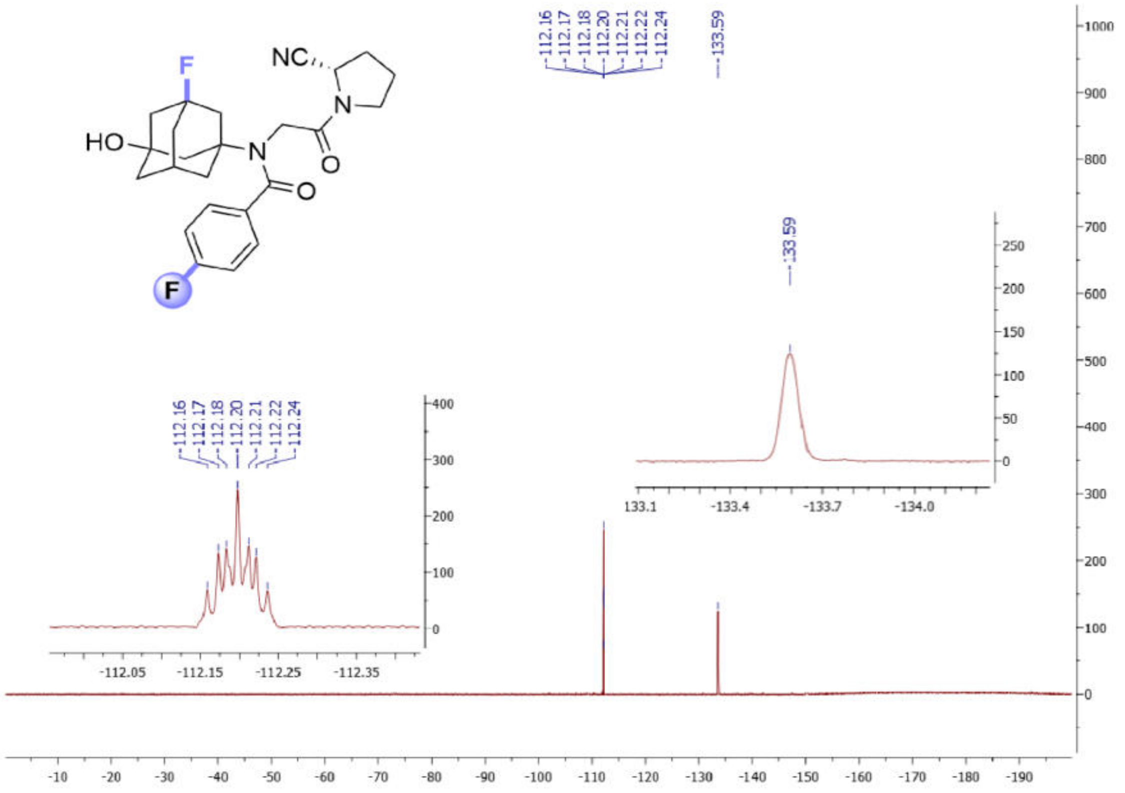

^{1}H NMR of compound **11h** in $CDCl_3$

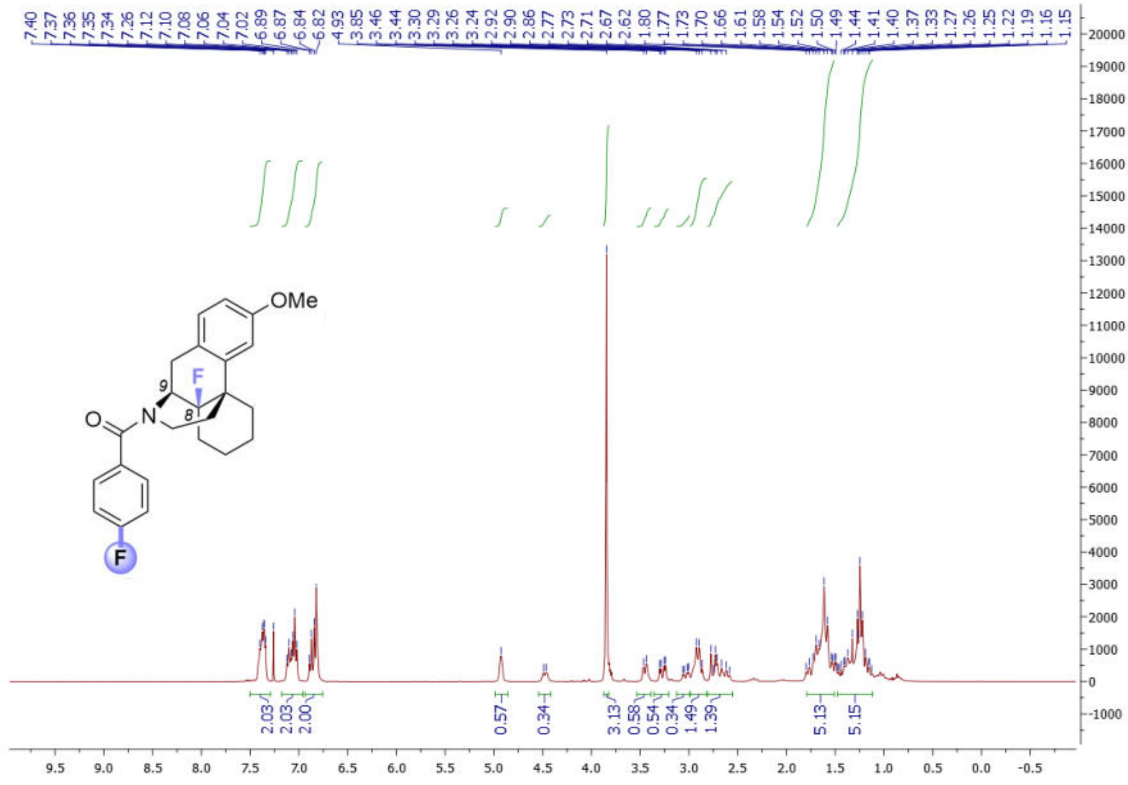

^{13}C NMR of compound **11h** in $CDCl_3$

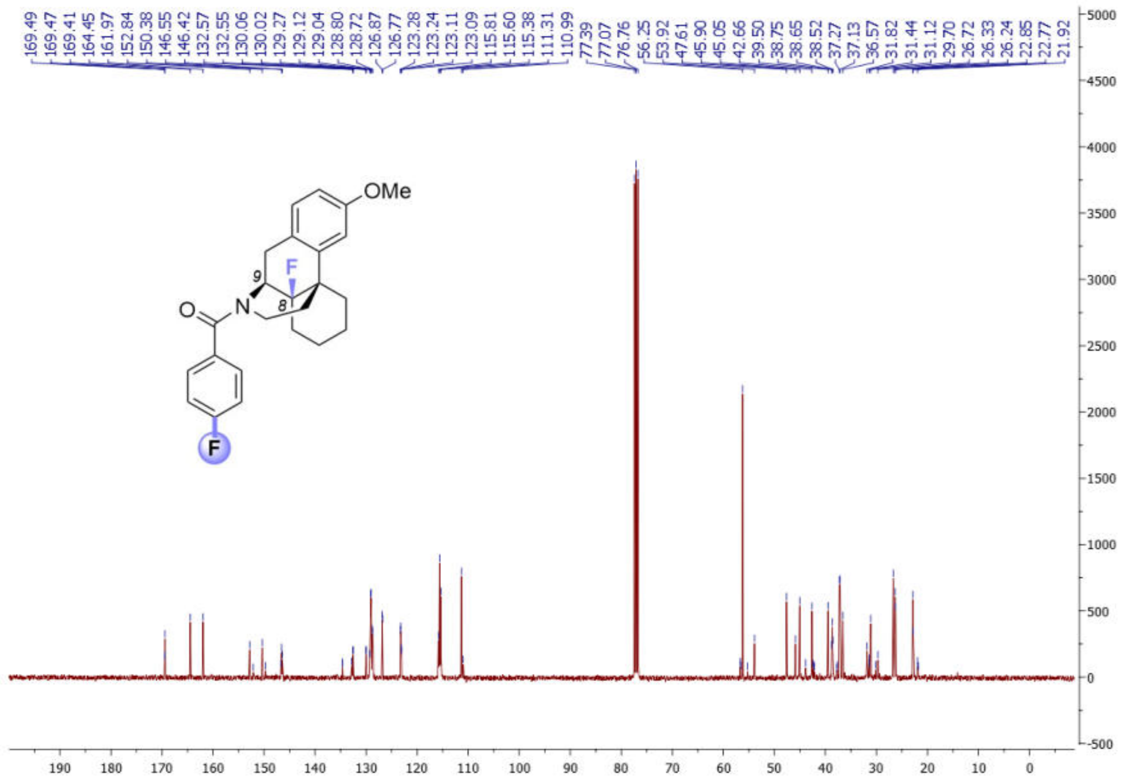

^{19}F NMR of compound **11h** in $CDCl_3$

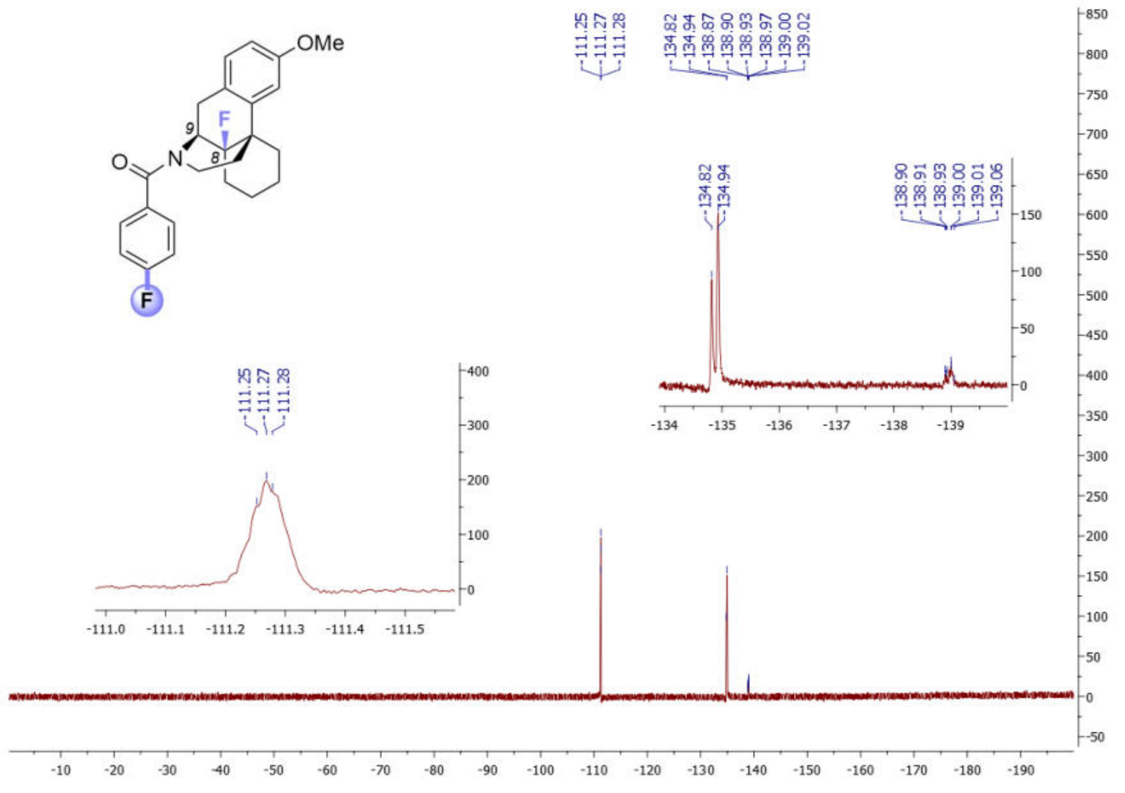

$^{19}F\{^{1}H\}$ NMR of compound **11h** in $CDCl_3$

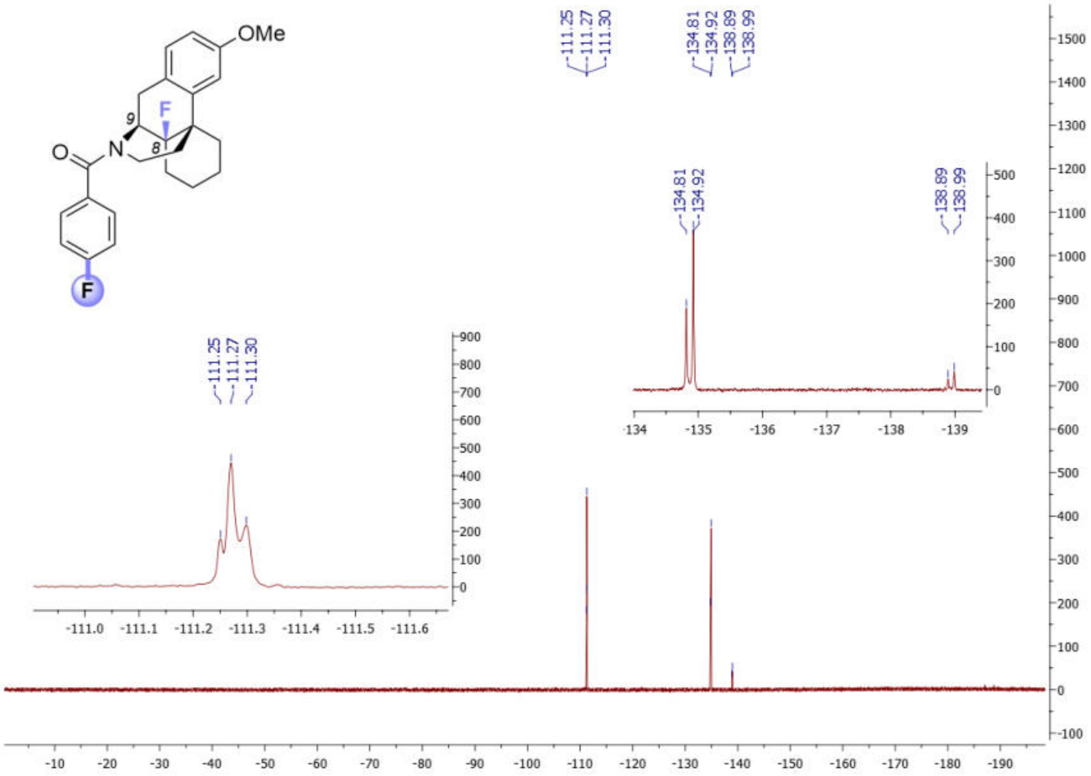

^{1}H NMR of compound **14h** in $CDCl_3$

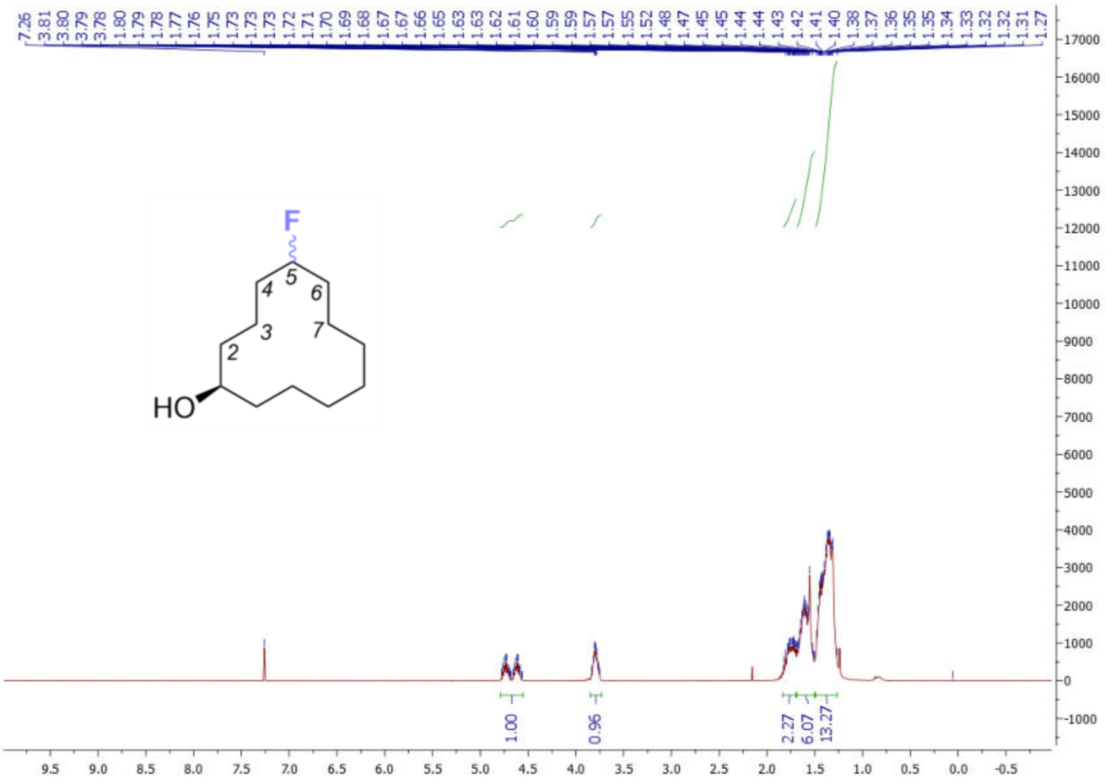

^{13}C NMR of compound **14h** in $CDCl_3$

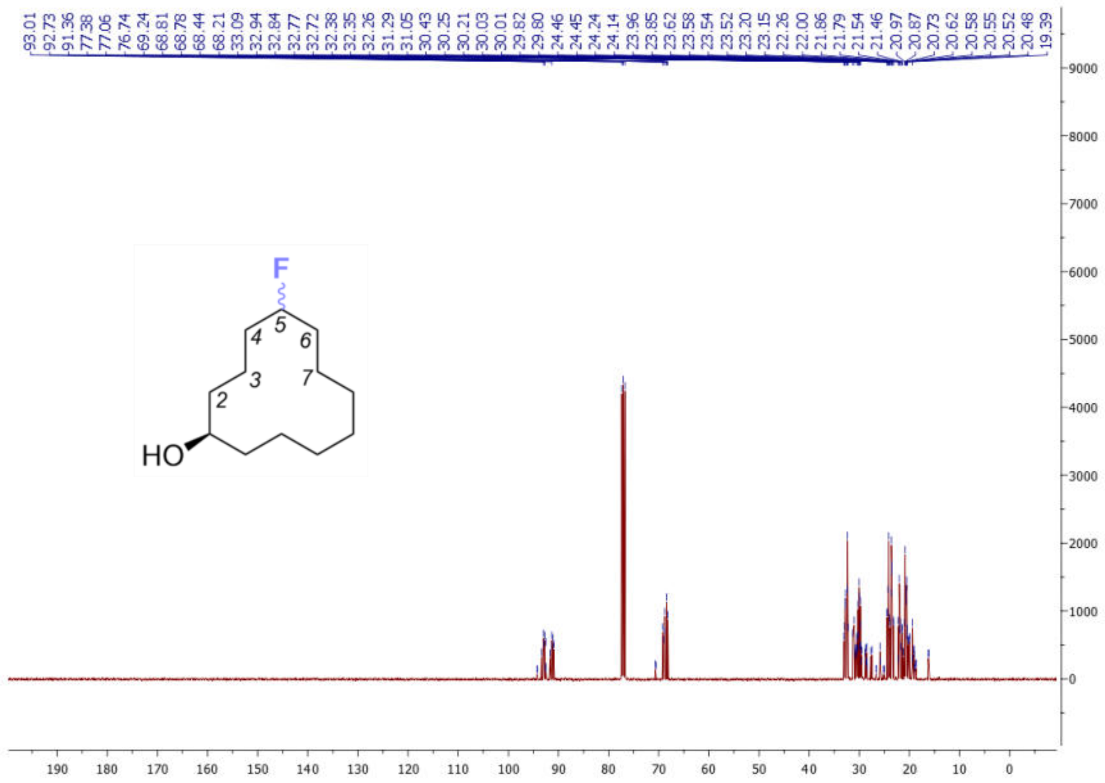

^{19}F NMR of compound **14h** in $CDCl_3$

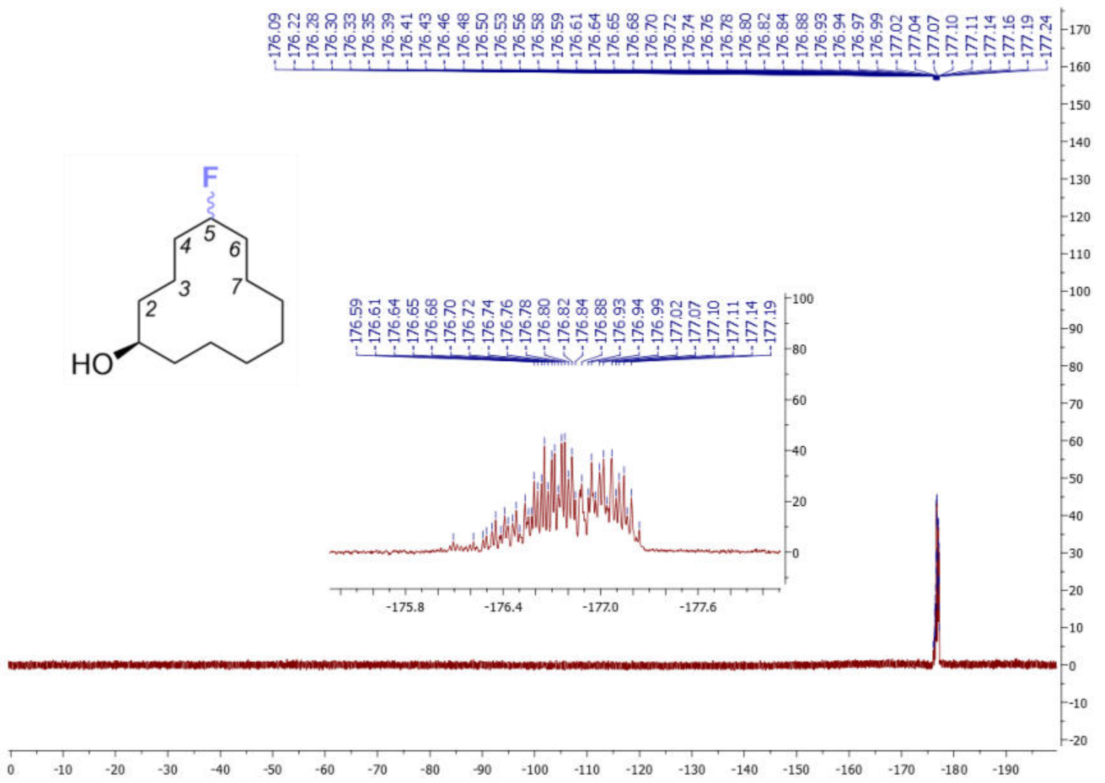

$^{19}F\{^{1}H\}$ NMR of compound **14h** in $CDCl_3$

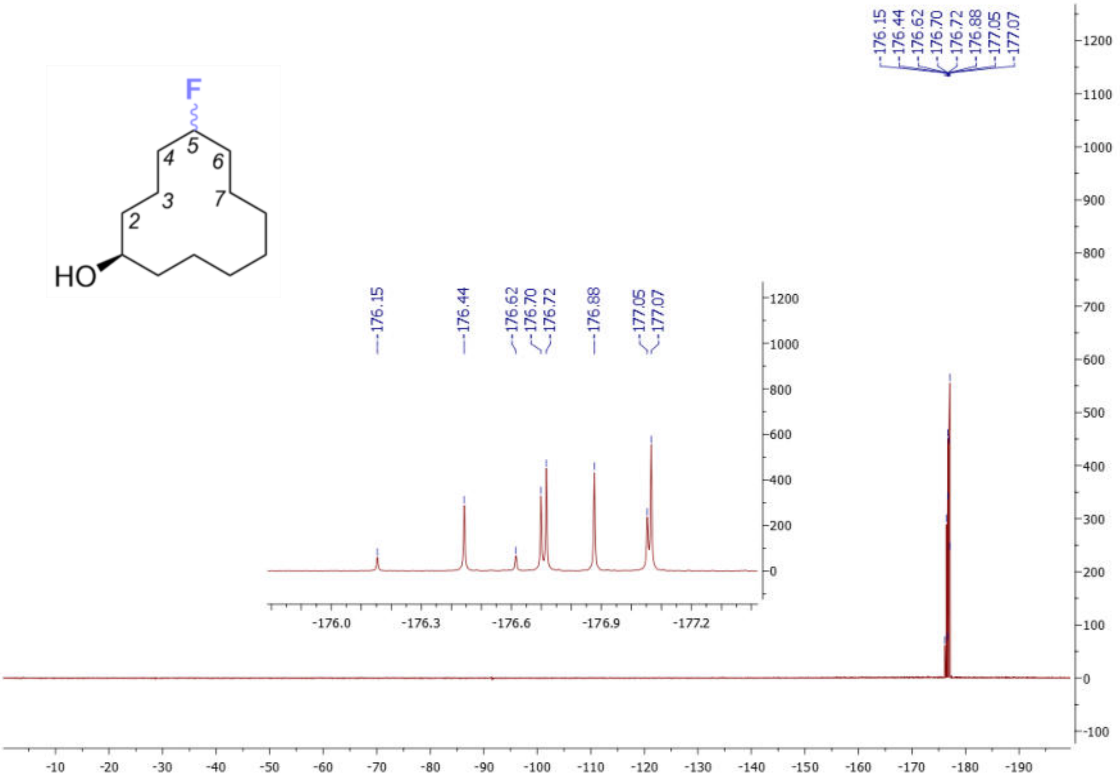

^{1}H NMR of compound **14d** in $CDCl_3$

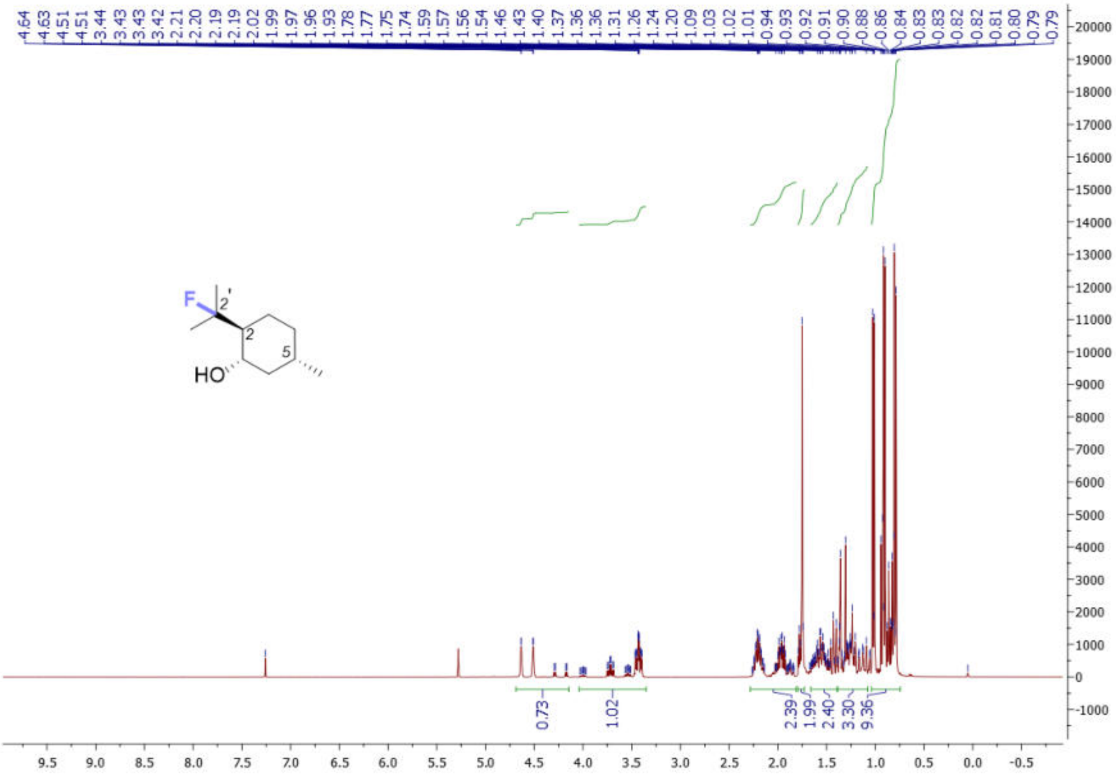

^{13}C NMR of compound **14d** in $CDCl_3$

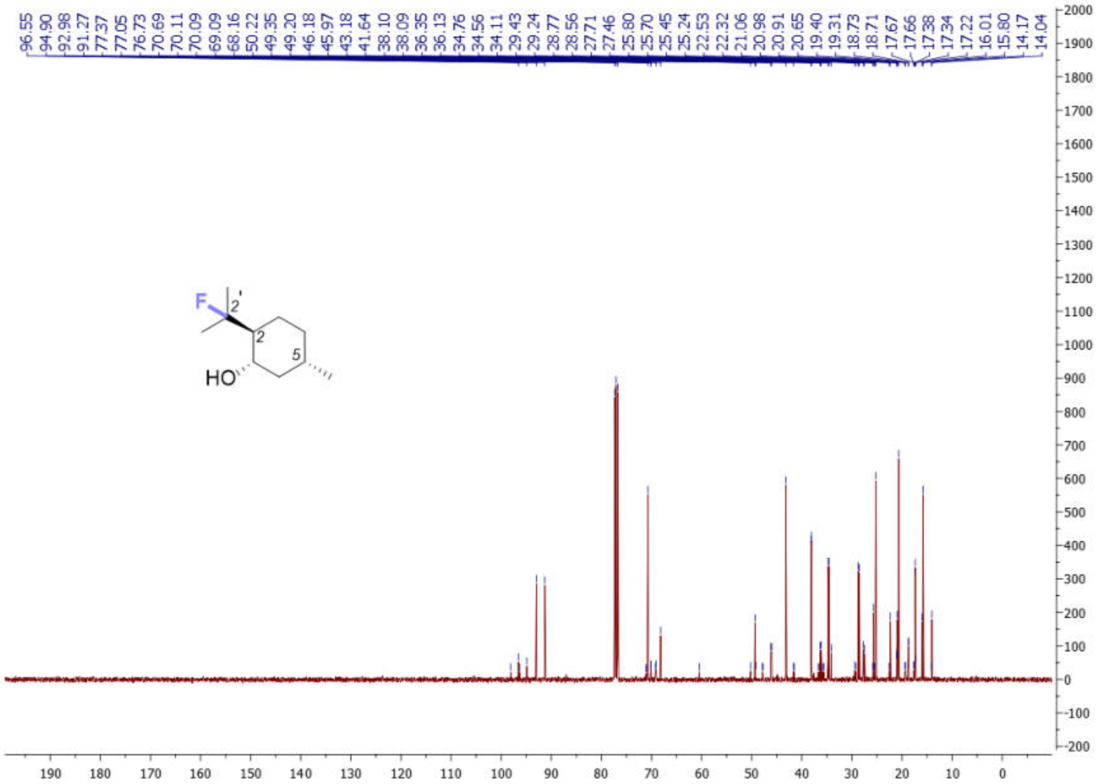

$^{19}F\{^1H\}$ NMR of compound **14d** in $CDCl_3$

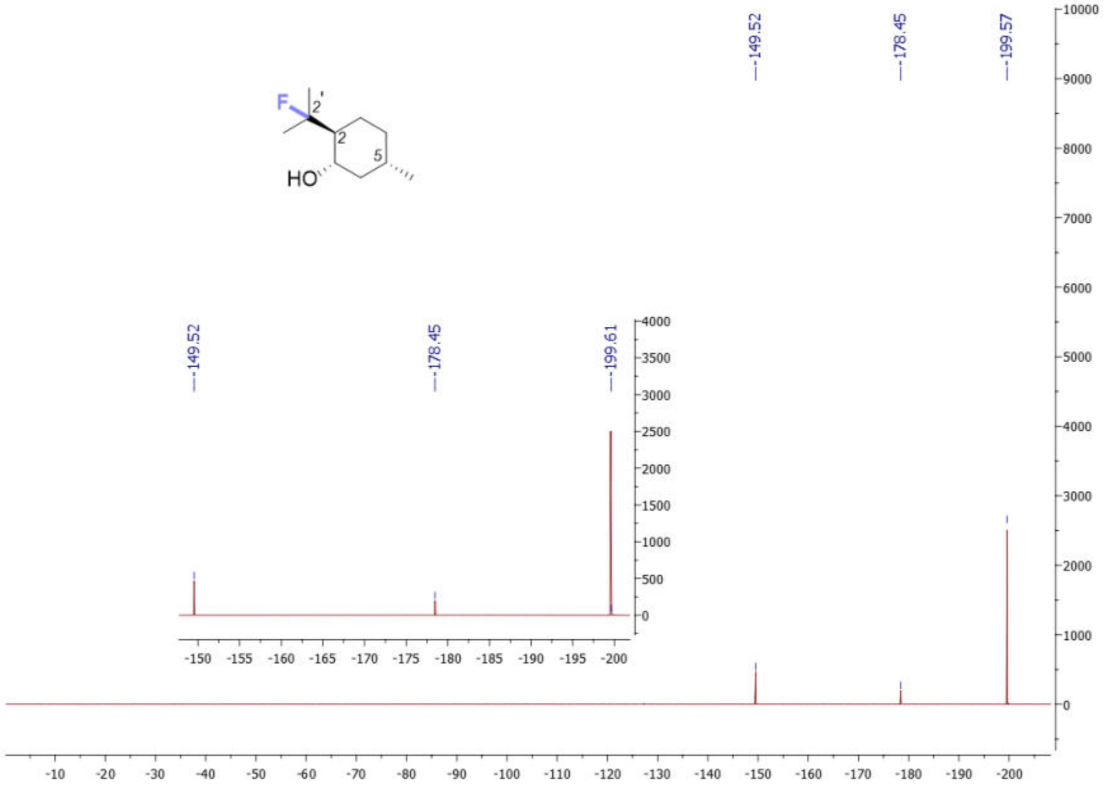

^{1}H NMR of compound **15c** in $CDCl_3$

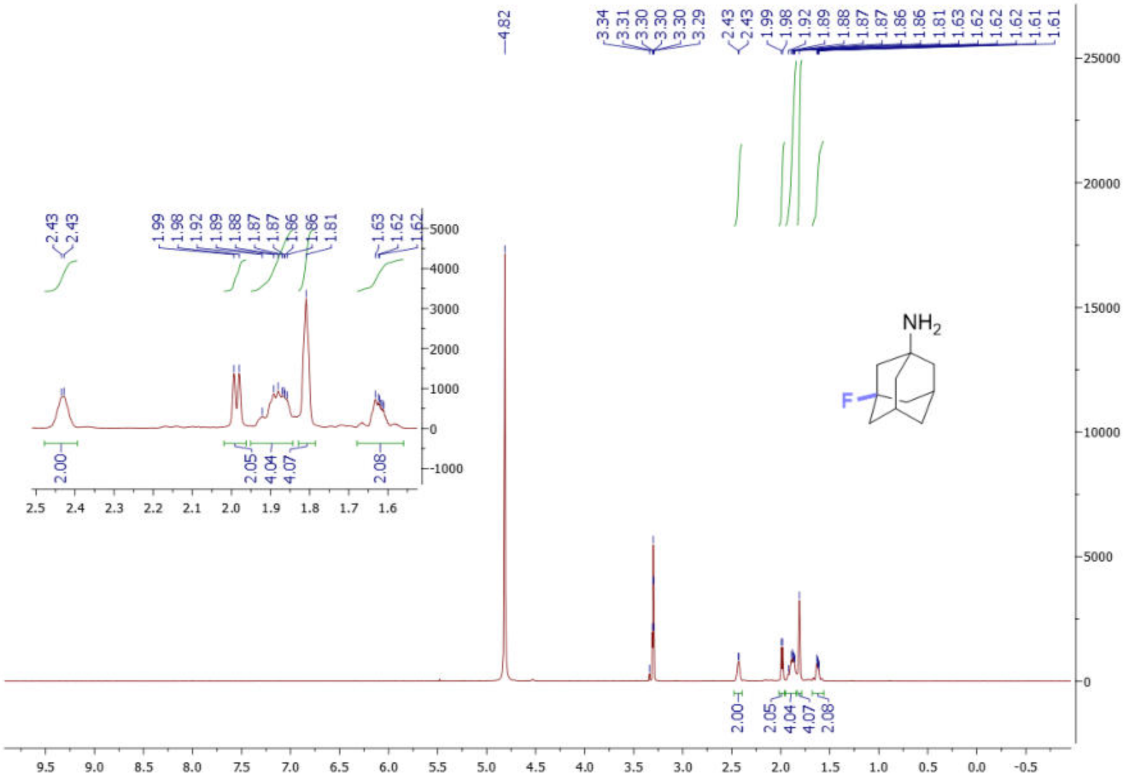

^{13}C NMR of compound **15c** in $CDCl_3$

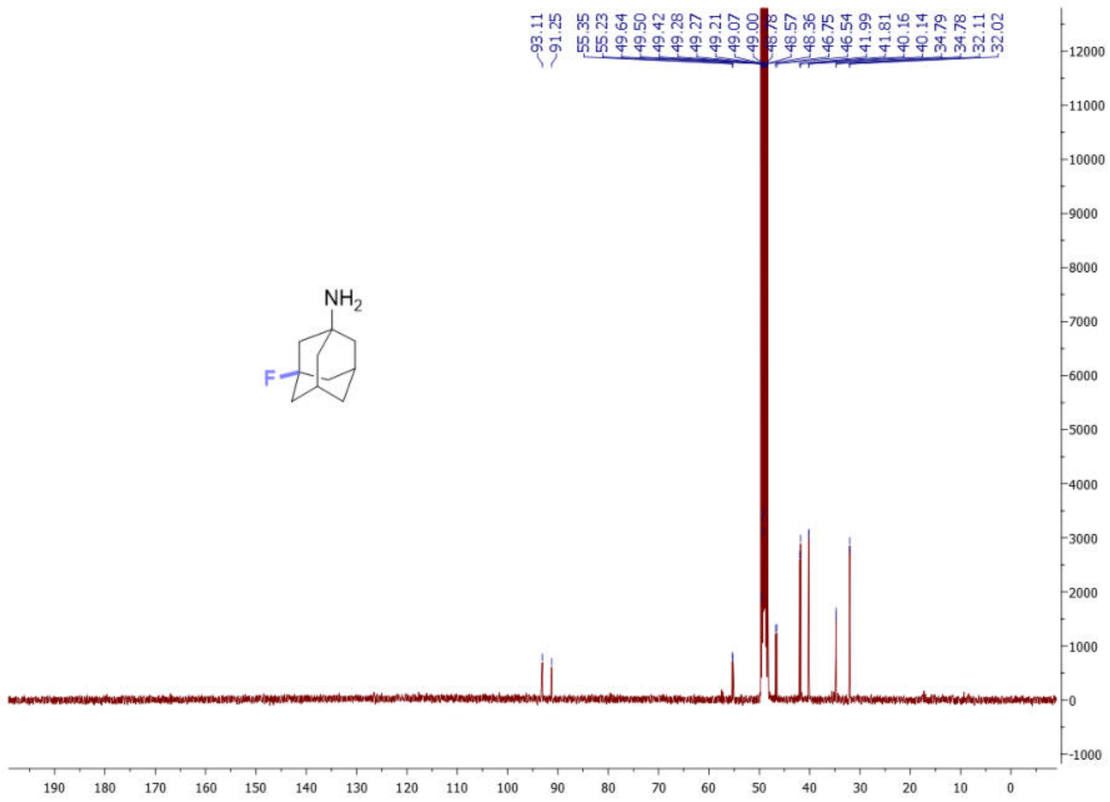

^{19}F NMR of compound **15c** in $CDCl_3$

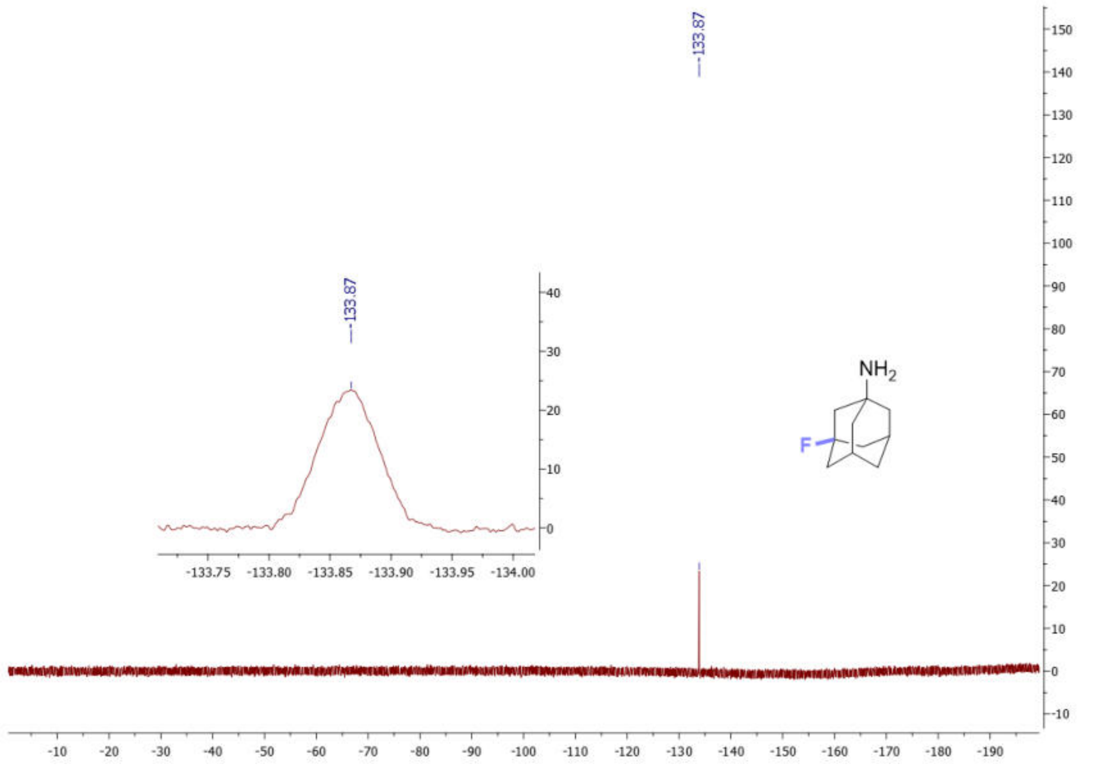

^{1}H NMR of compound **15f** in $CDCl_3$

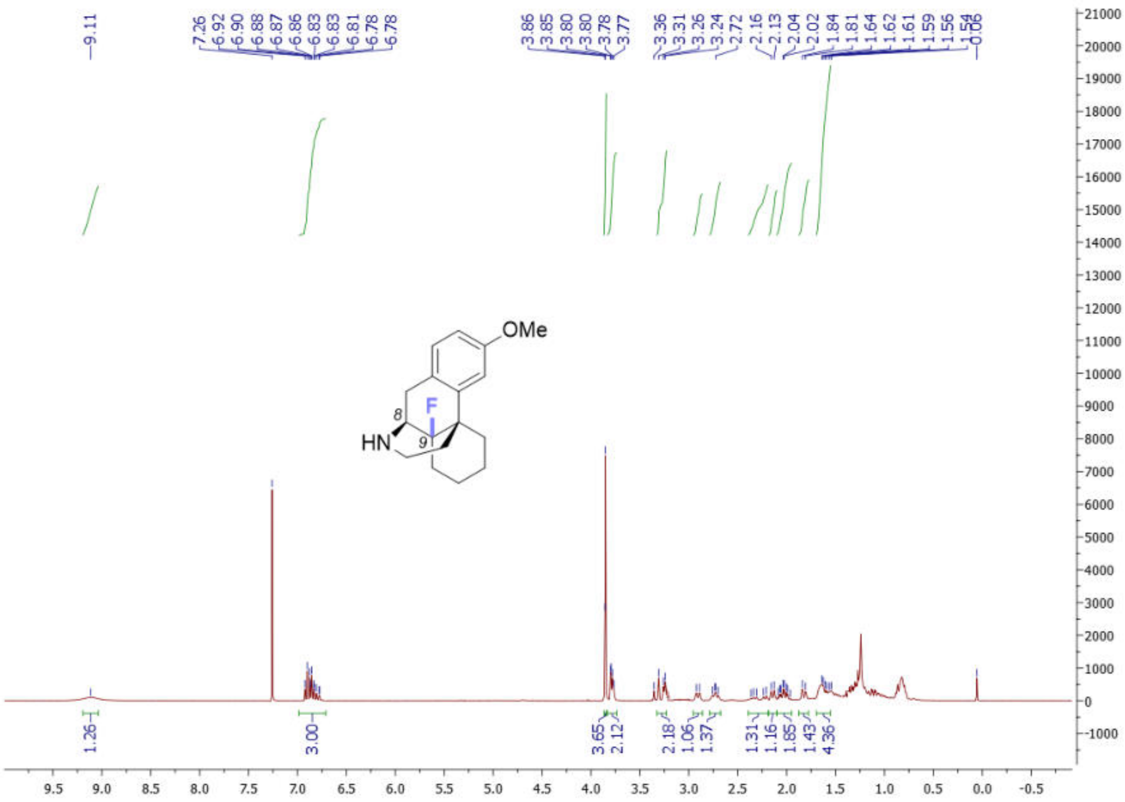

^{13}C NMR of compound **15f** in $CDCl_3$

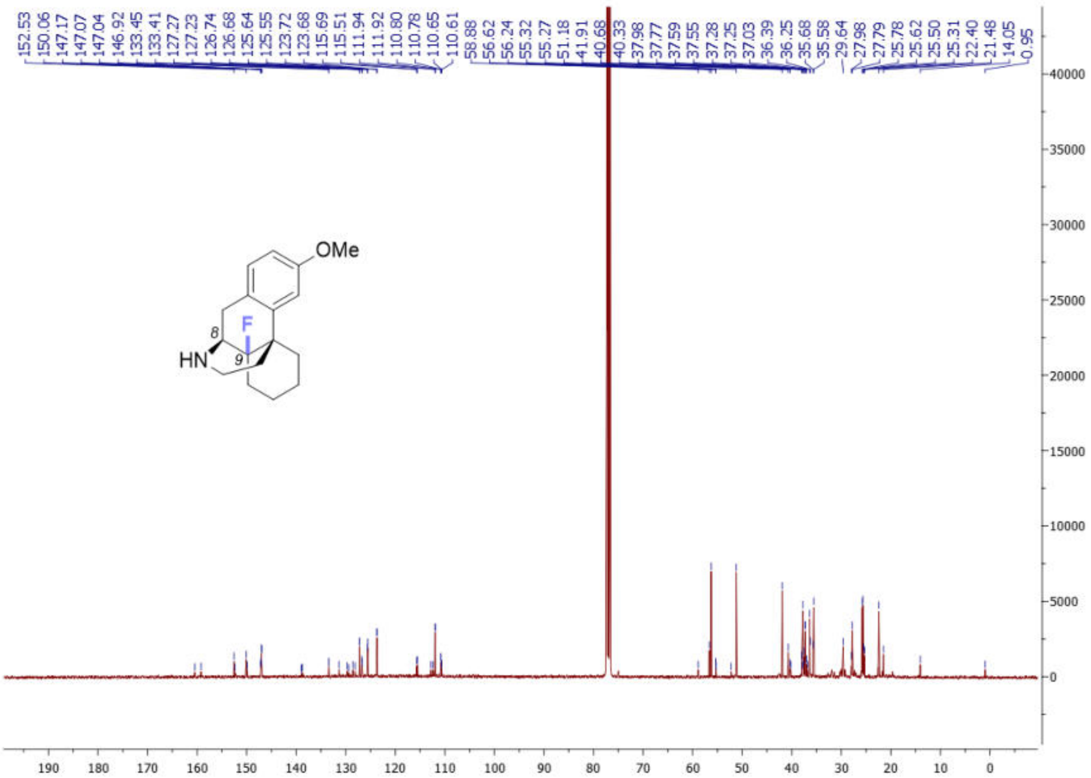

^{19}F NMR of compound **15f** in $CDCl_3$

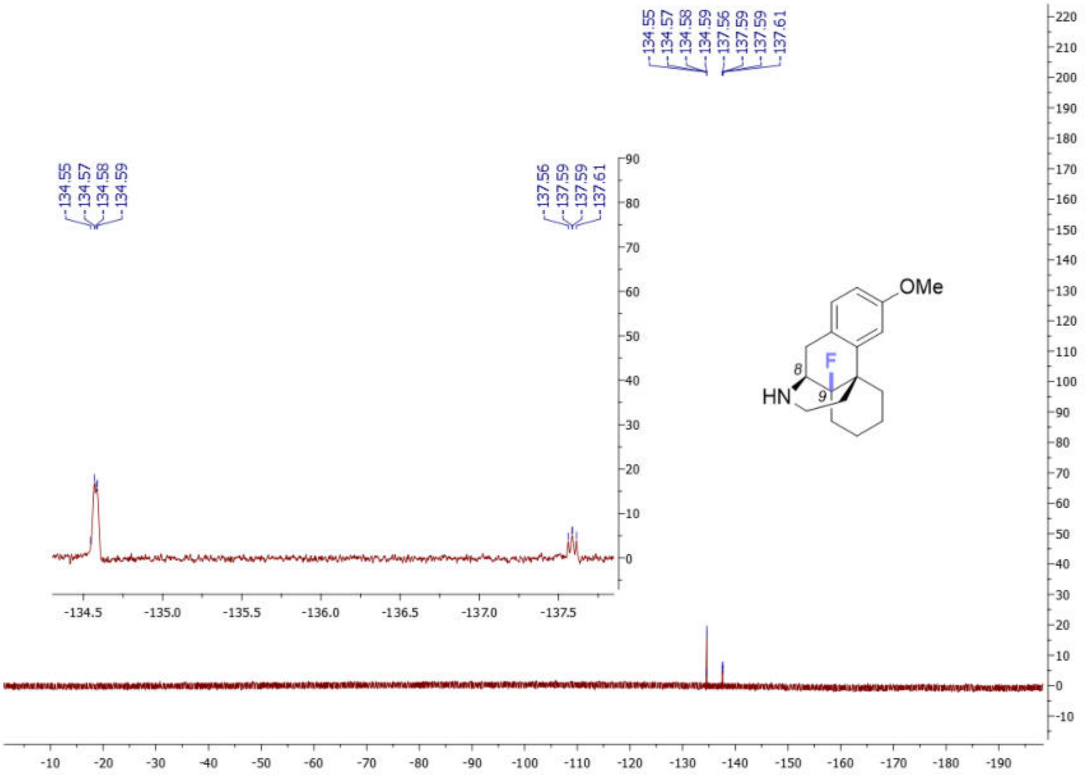

$^{19}F\{^1H\}$ NMR of compound **15f** in $CDCl_3$

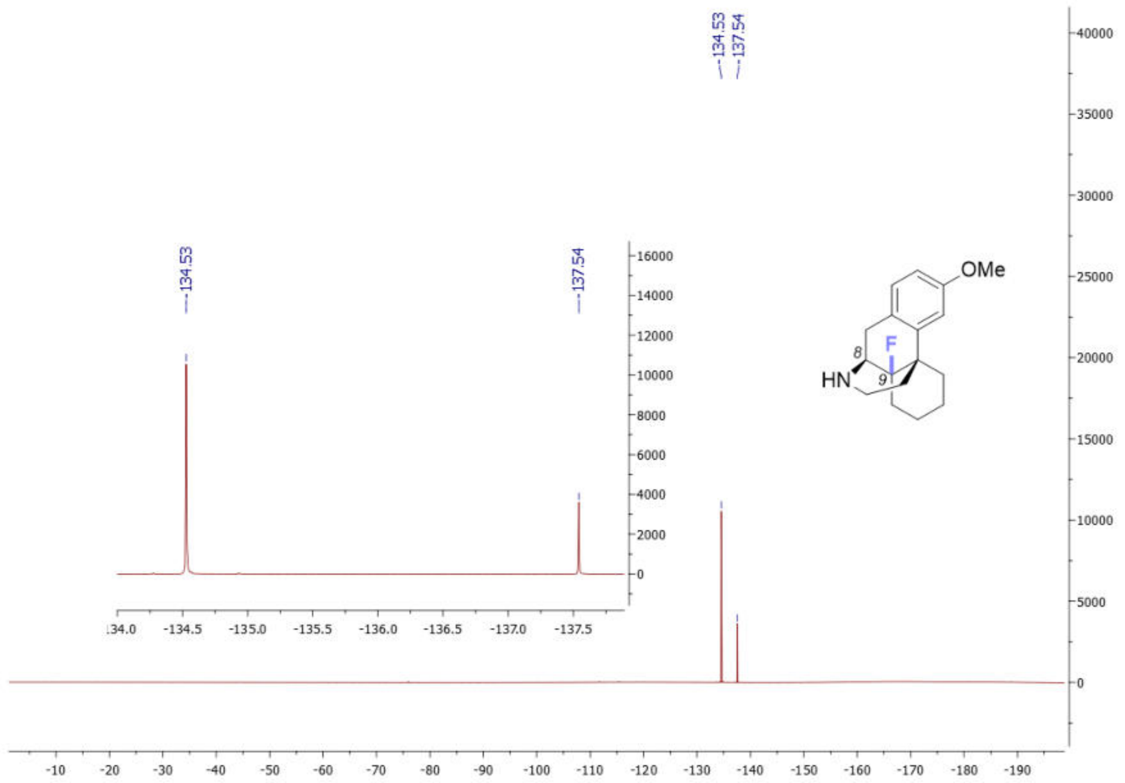

^1H NMR of compound **15b** in $CDCl_3$

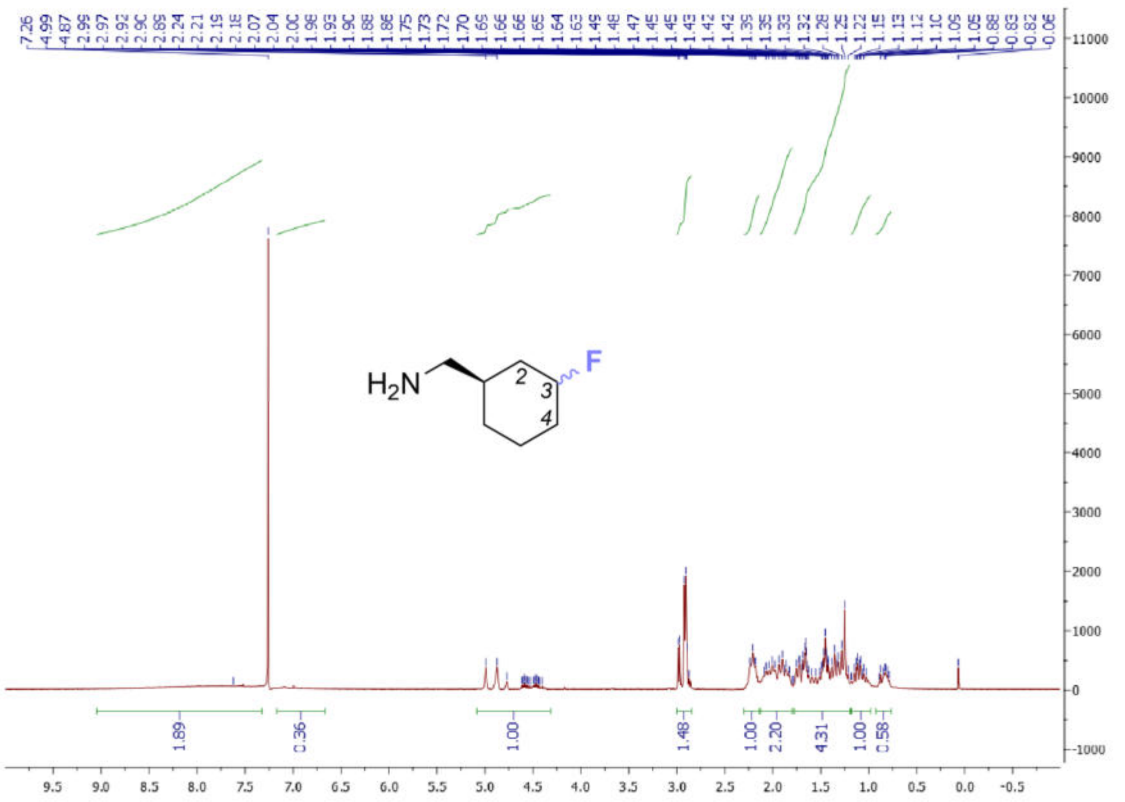

^{13}C NMR of compound **15b** in $CDCl_3$

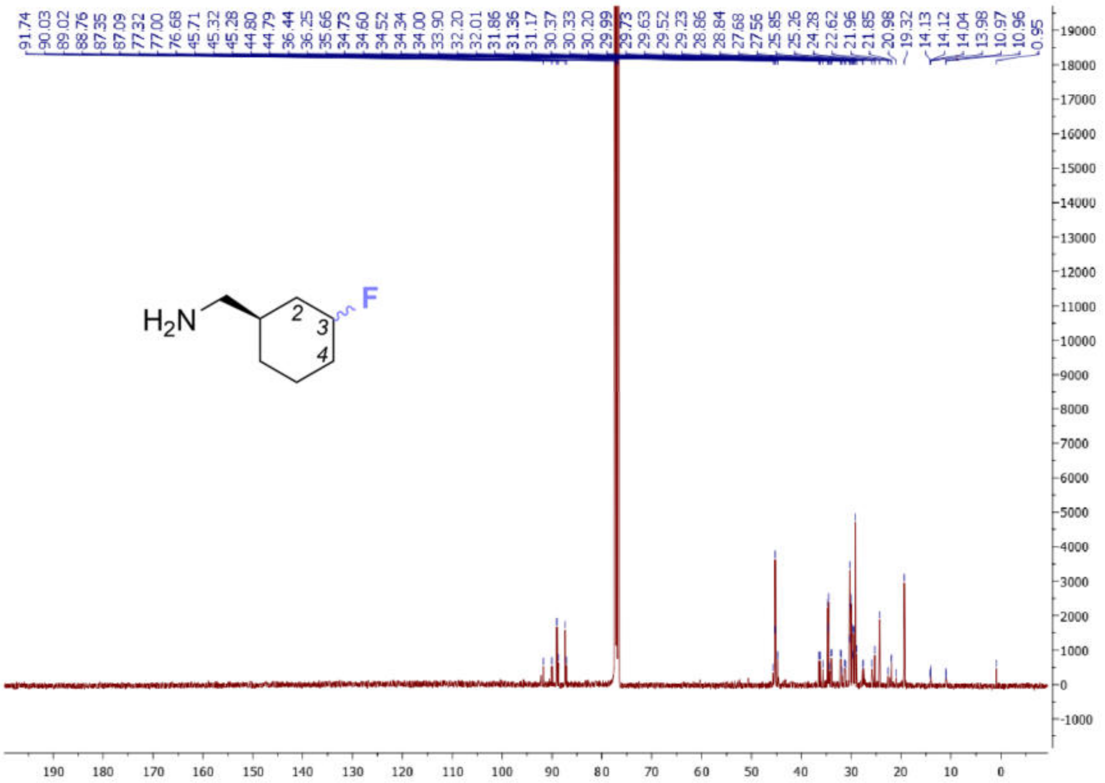

$^{19}F\{^1H\}$ NMR of compound **15b** in $CDCl_3$

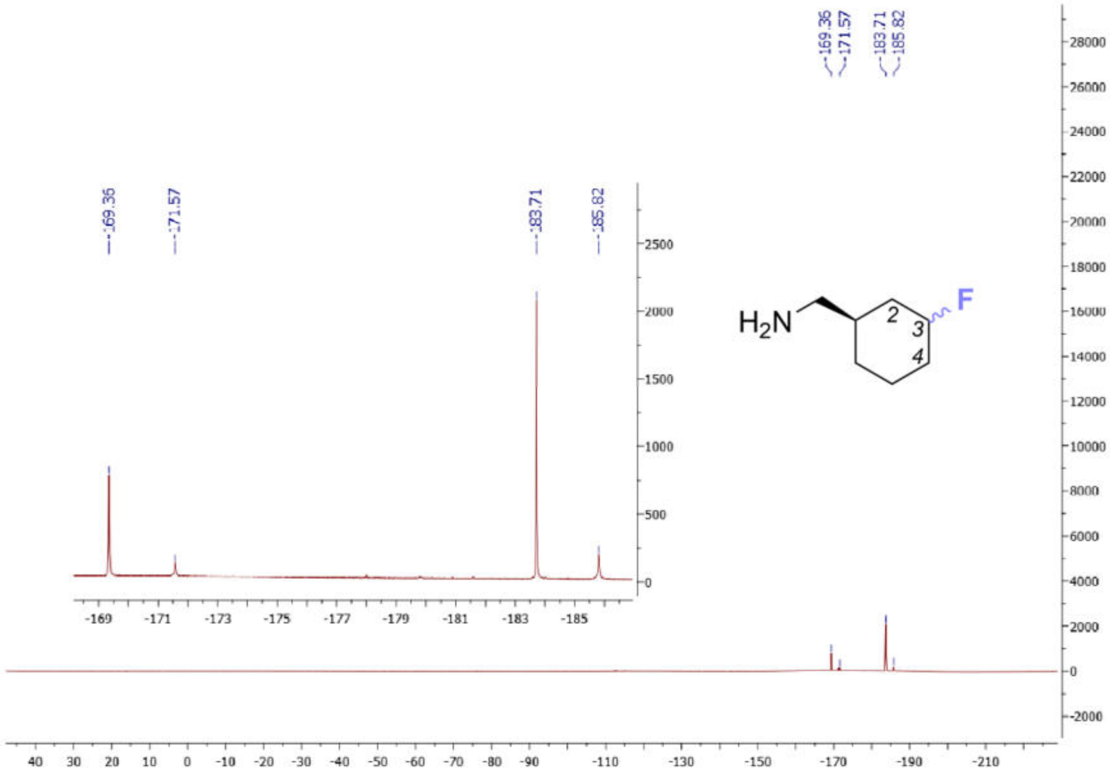

^{1}H NMR of compound **22** in $CDCl_3$

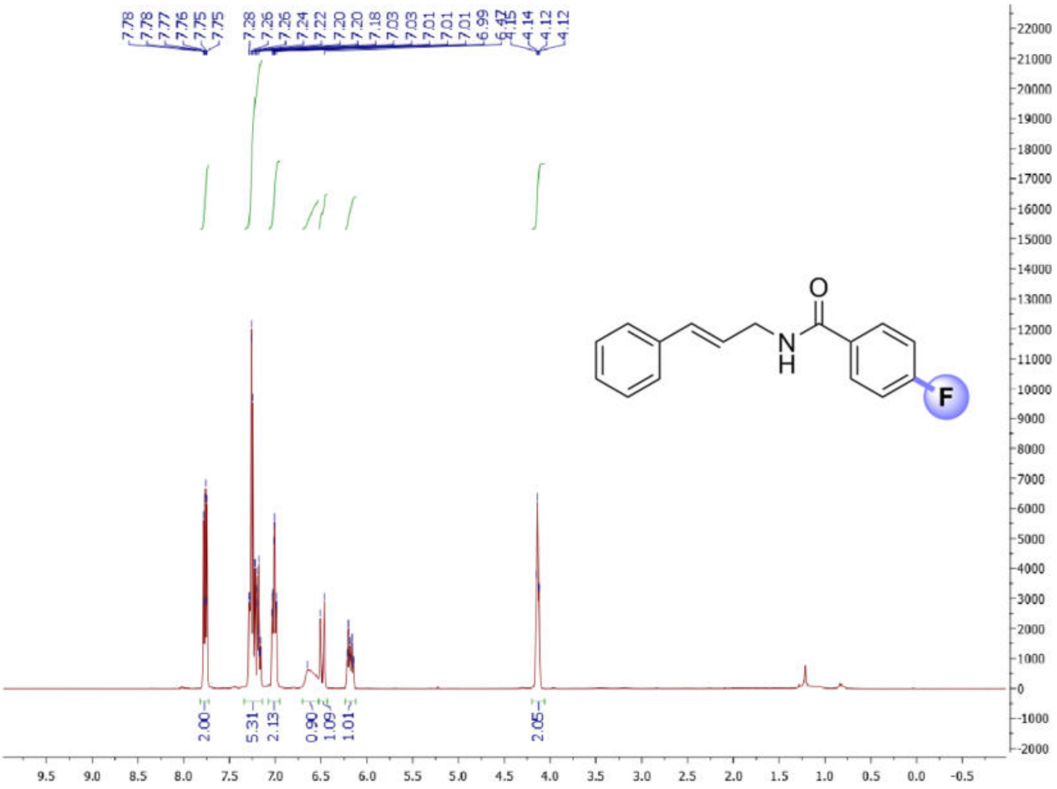

^{13}C NMR of compound **22** in $CDCl_3$

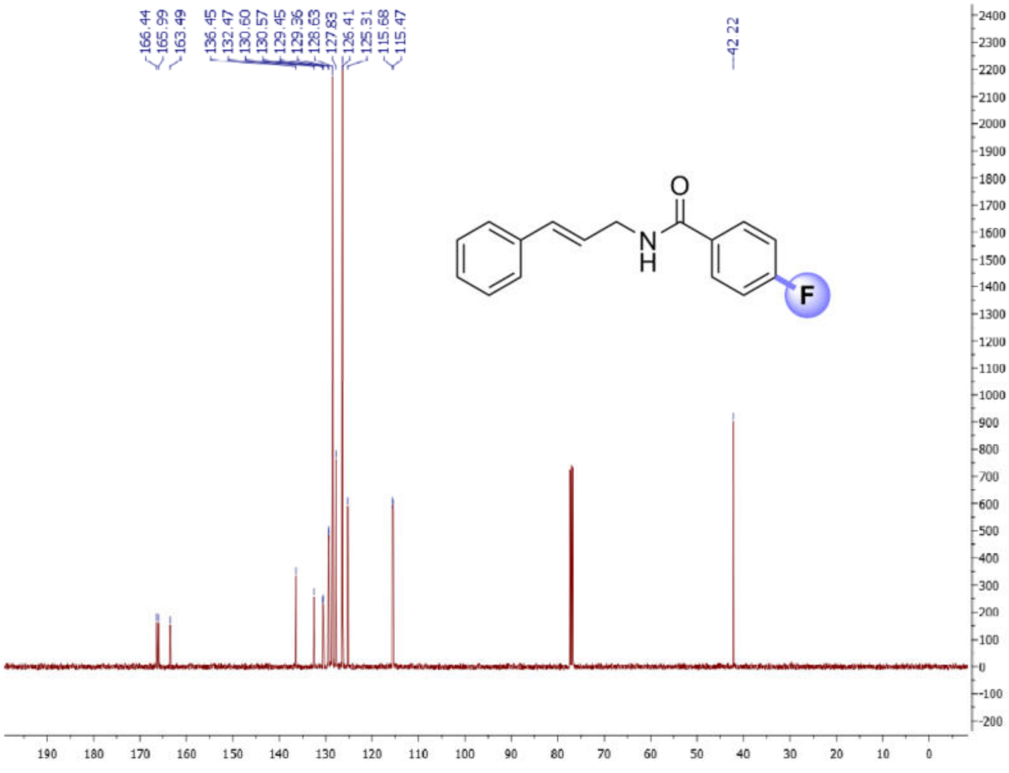

^{19}F NMR of compound **22** in $CDCl_3$

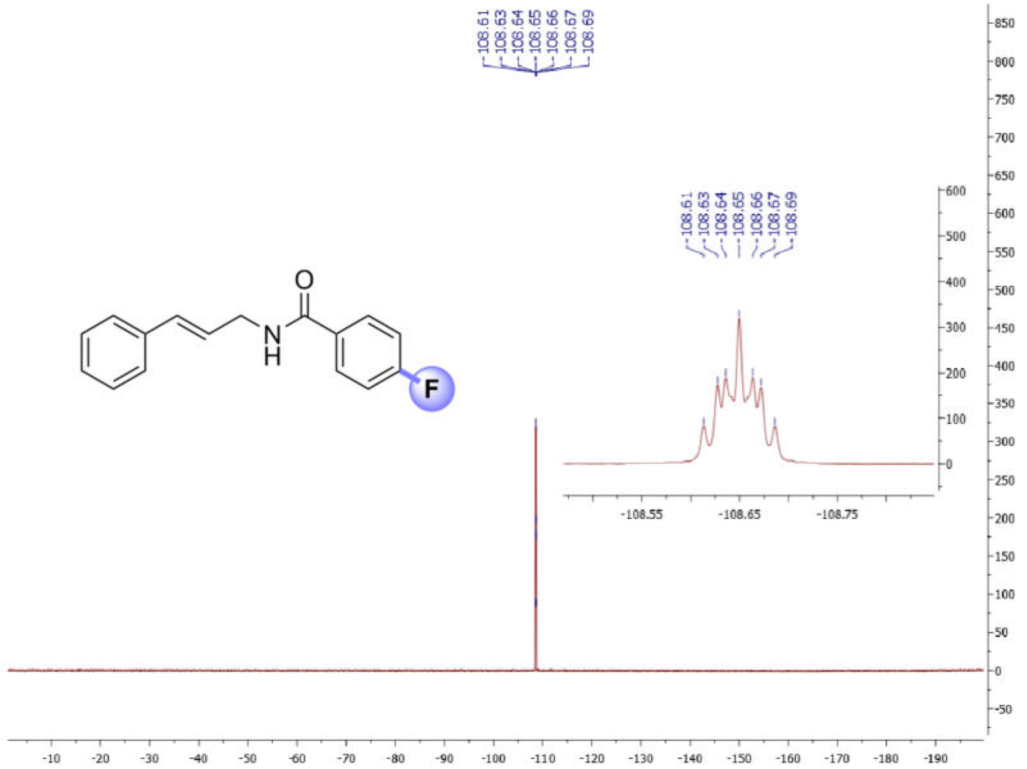

^{1}H NMR of compound **23** in $CDCl_3$

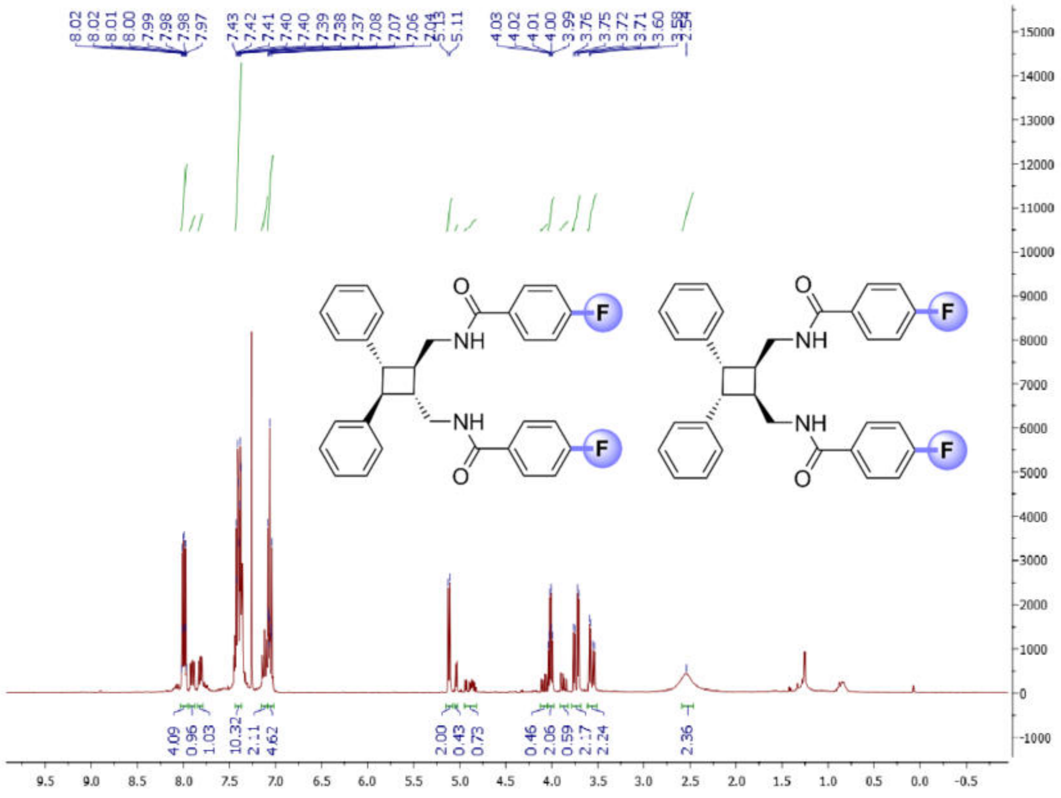

^{13}C NMR of compound **23** in $CDCl_3$

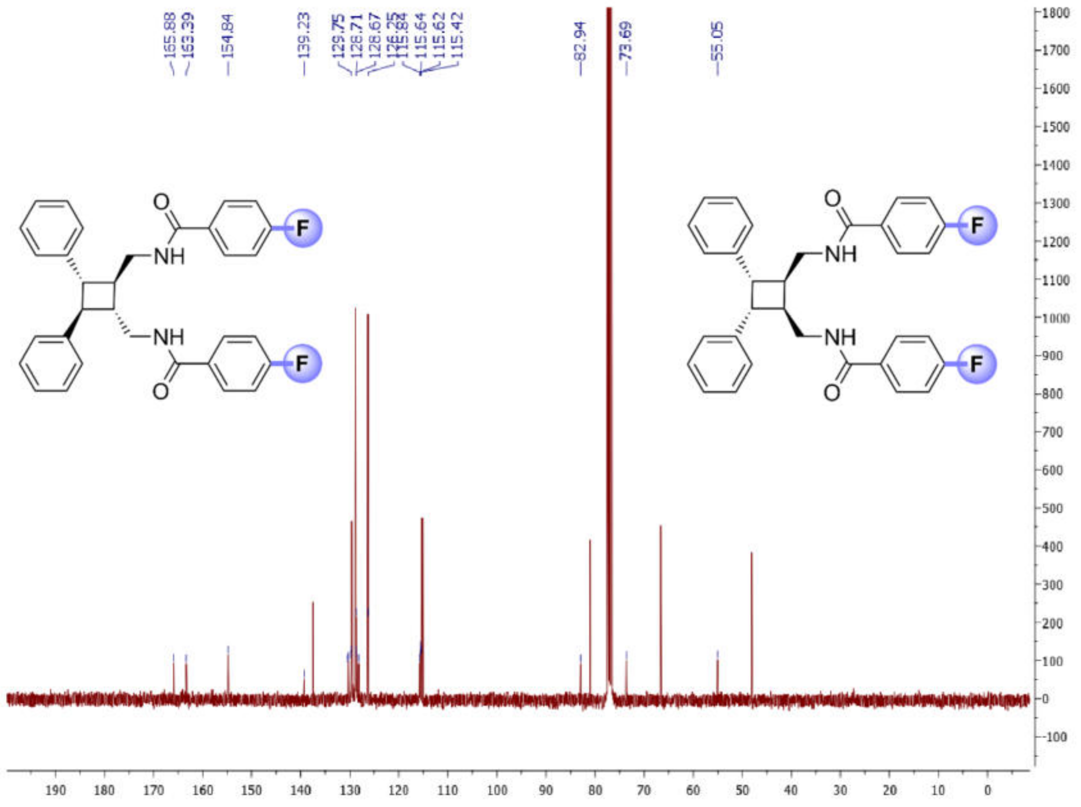

^{19}F NMR of compound **23** in $CDCl_3$

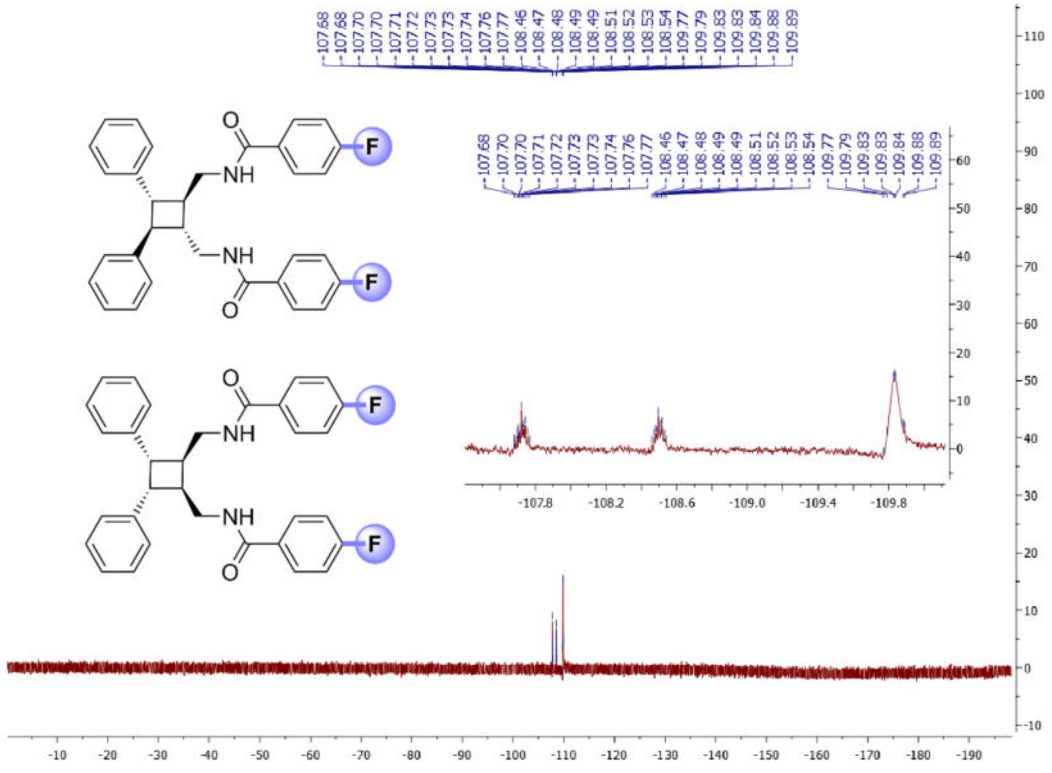

COSY NMR of compound **23** in $CDCl_3$

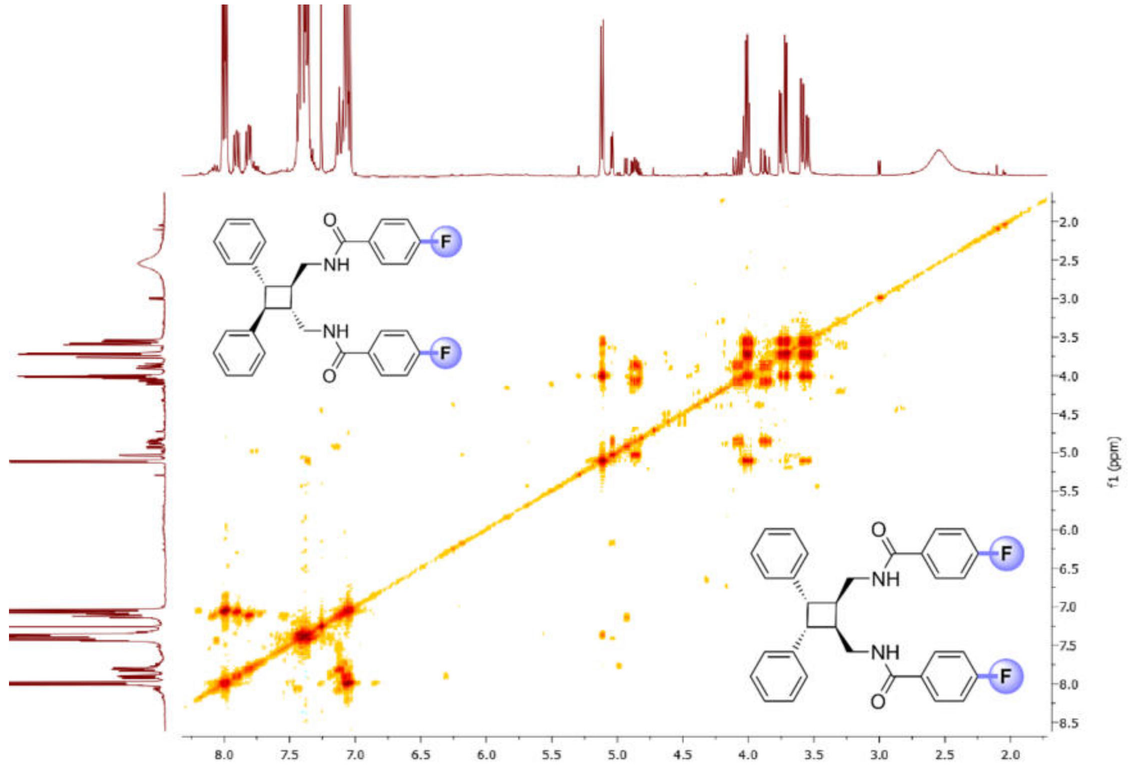

TOCSY NMR of compound **23** in $CDCl_3$

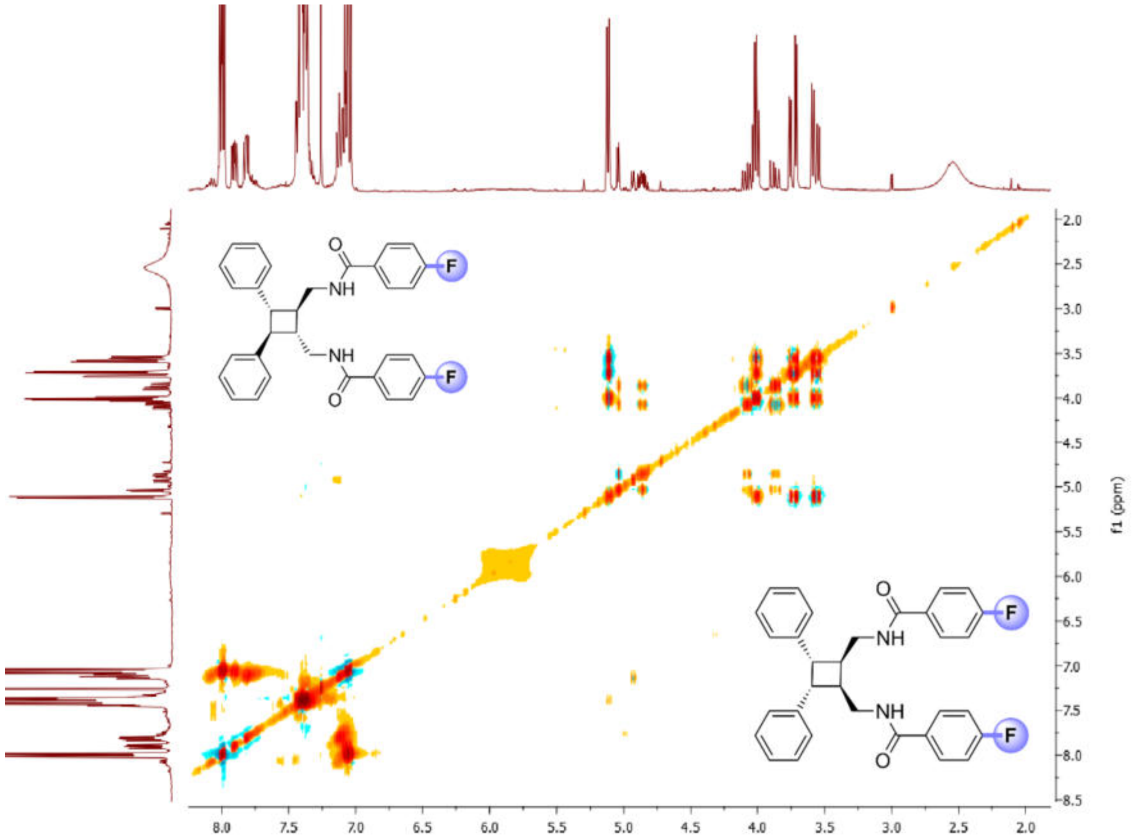

HSQC NMR of compound **23** in $CDCl_3$

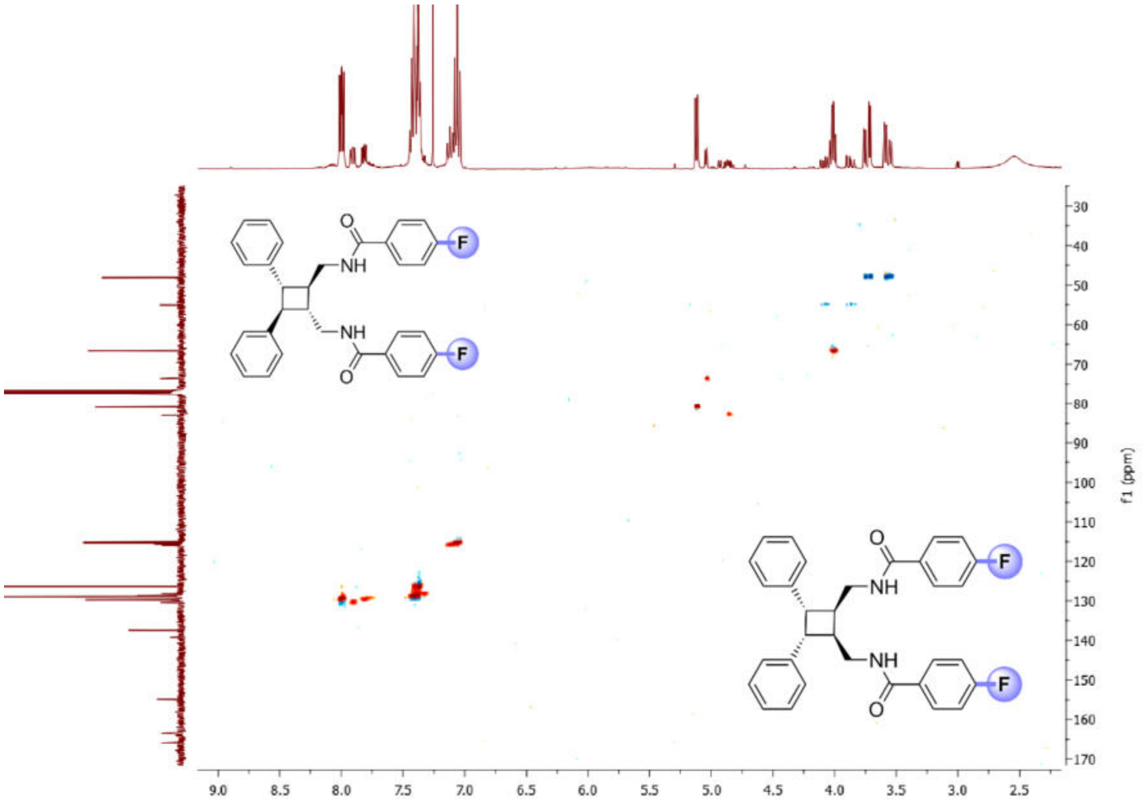

HMBC NMR of compound **23** in $CDCl_3$

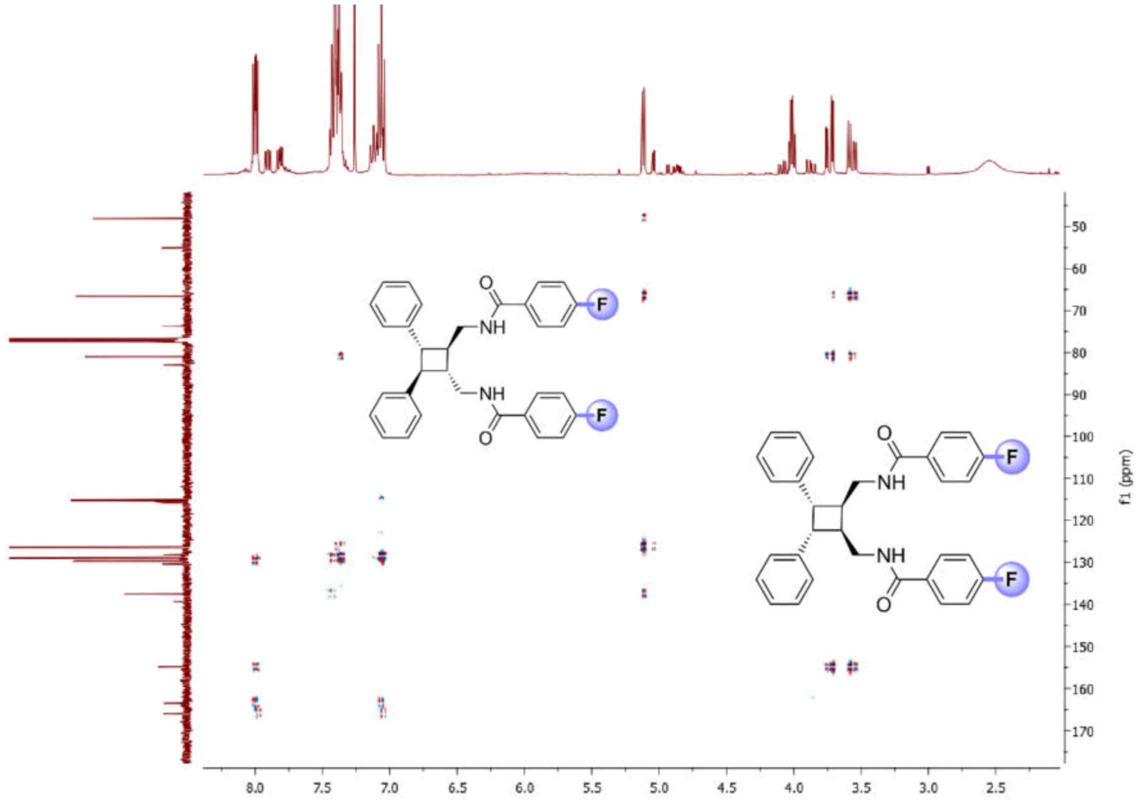

^{1}H NMR of compound **24** in $CDCl_3$

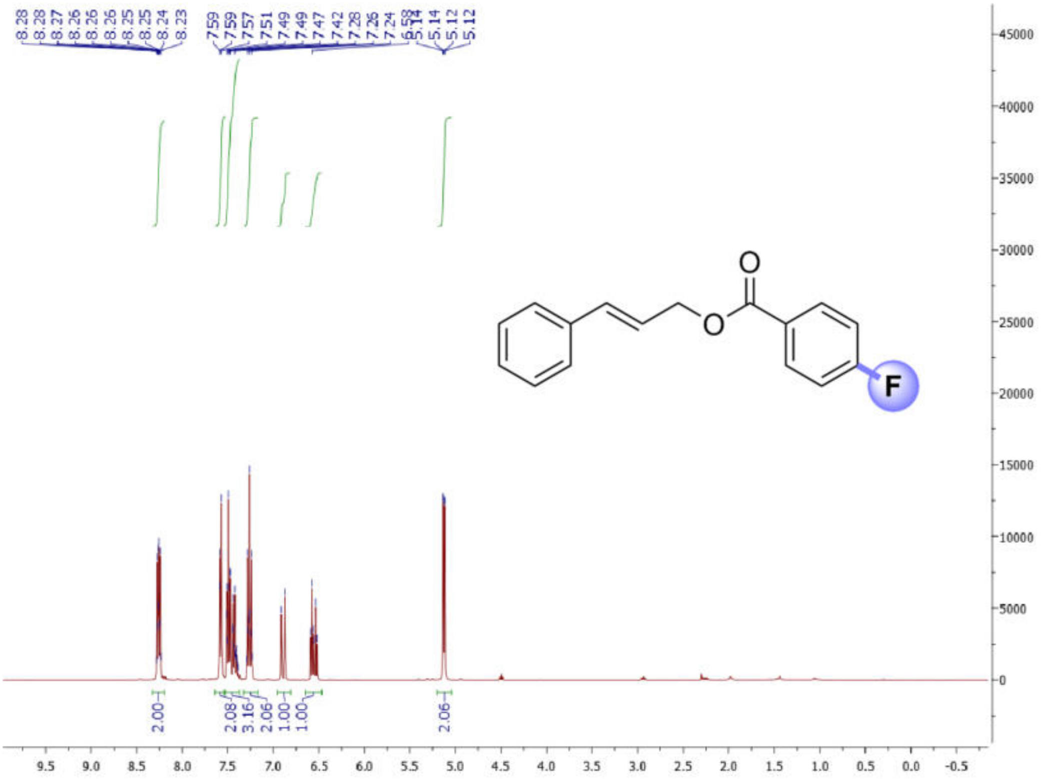

^{13}C NMR of compound **24** in $CDCl_3$

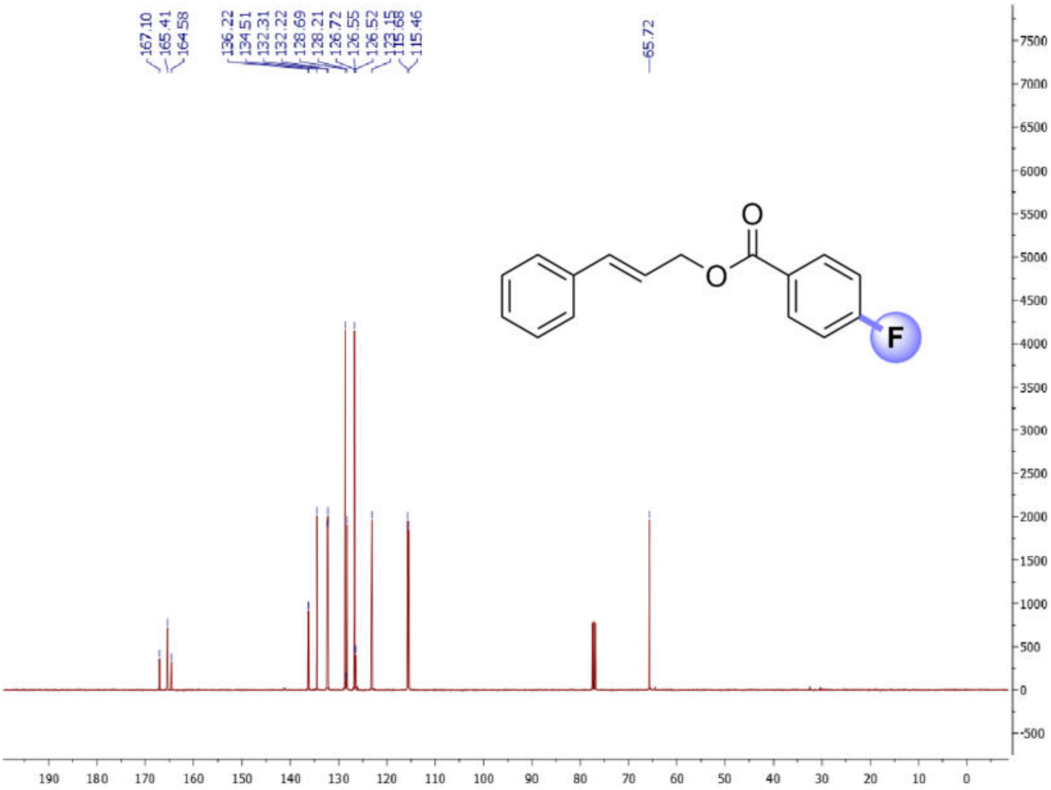

^{19}F NMR of compound **24** in $CDCl_3$

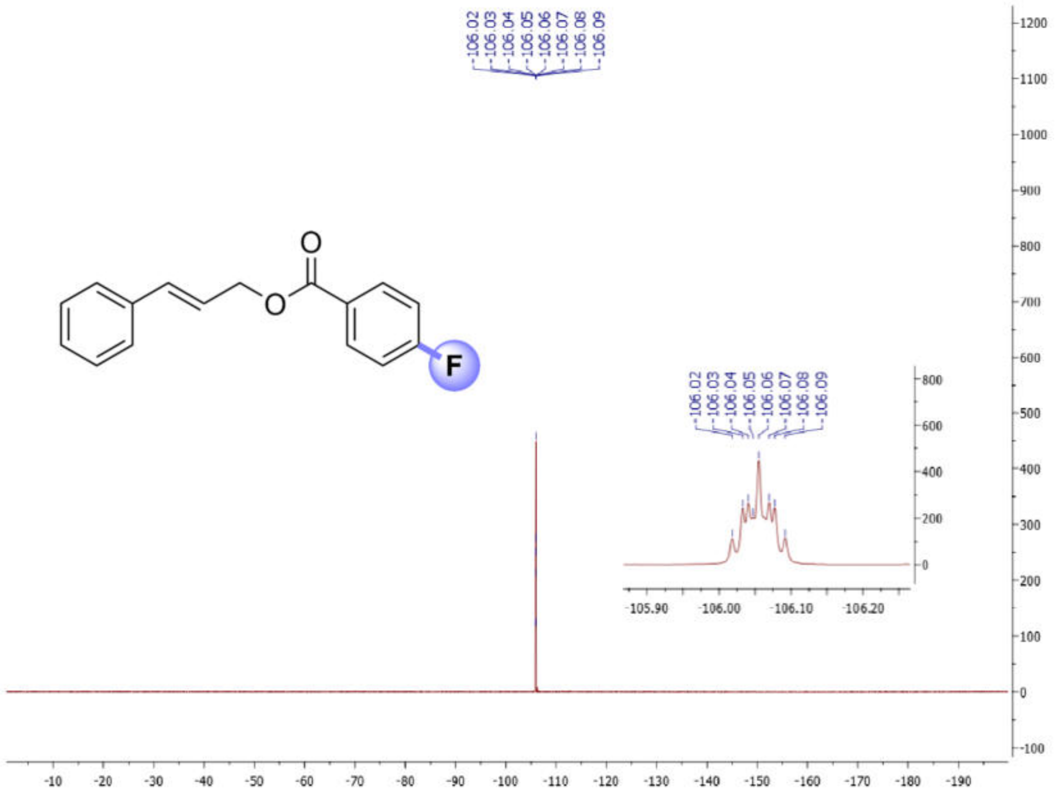

^{1}H NMR of compound **25** in $CDCl_3$

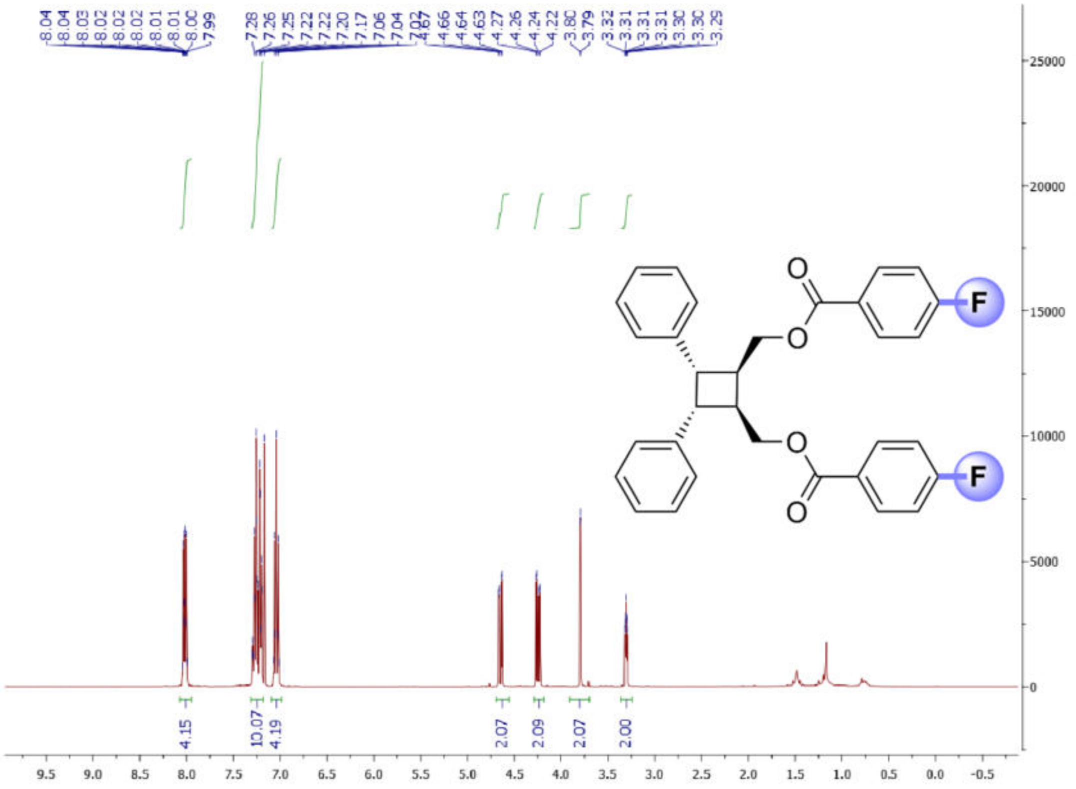

^{13}C NMR of compound **25** in $CDCl_3$

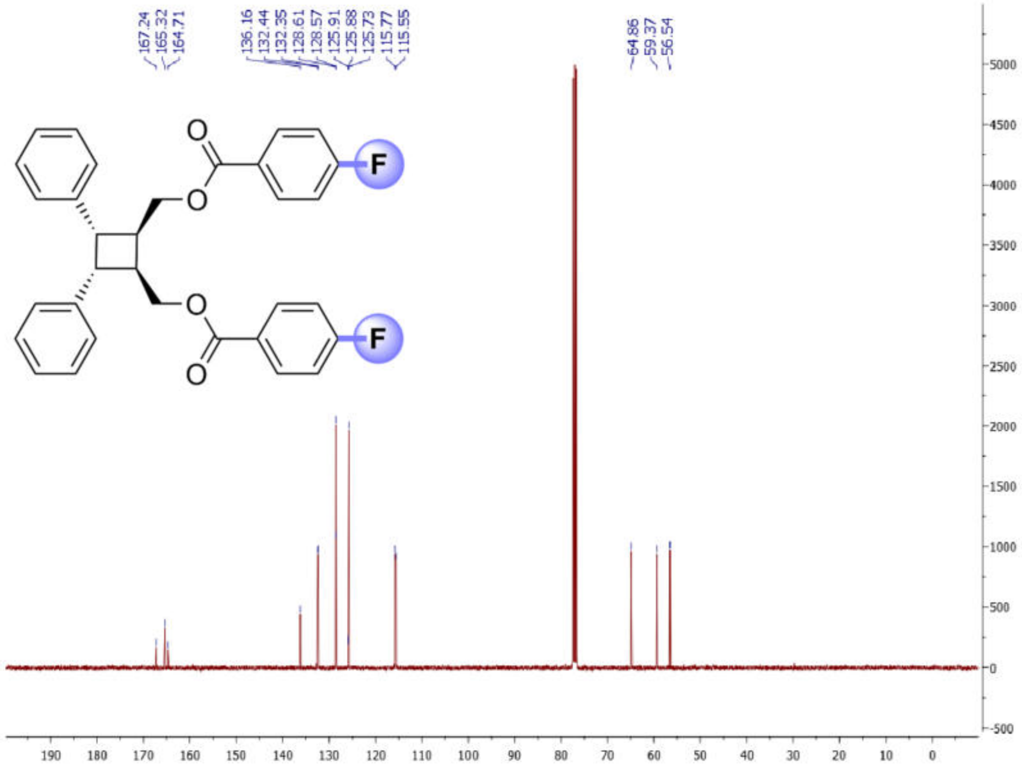

^{19}F NMR of compound **25** in $CDCl_3$

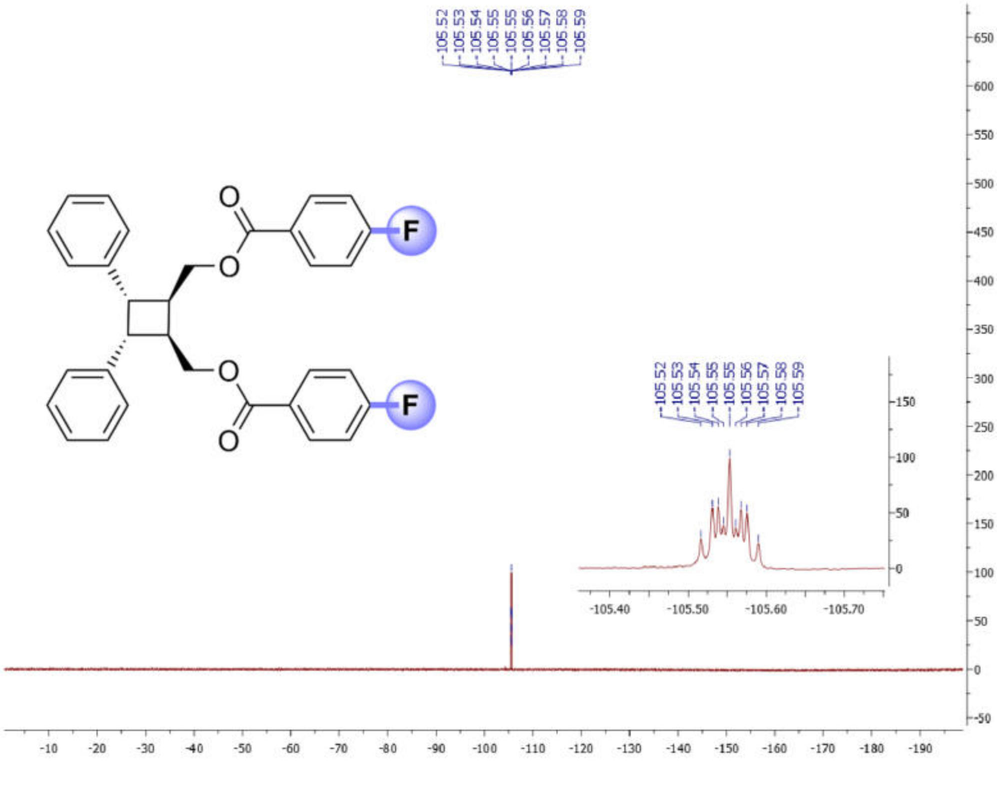

5 X-Ray Crystallography

Single crystal XRD data were recorded for suitable crystals of **11d** and **2o**. Empirical multi-scan[51] and analytical absorption corrections[52] were applied to the data. Experimental details as specified below:

Crystal data for 11d:

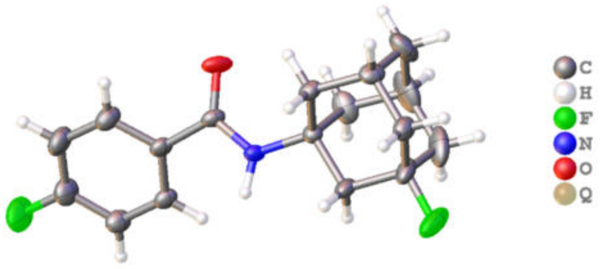

Experimental. Single clear colourless plate-shaped crystals of **11d** (**CCDC 2212436**) were used as supplied. A suitable crystal with dimensions 0.22 × 0.09 × 0.02 mm^3 was selected and mounted on a MITIGEN holder inert oil on a XtaLAB Synergy R, DW system, HyPix-Arc 150 diffractometer. The crystal was kept at a steady T = 123.01(10) K during data collection. The structure was solved with the ShelXT 2018/2 solution program[53] using dual methods and by using Olex2 1.5-alpha[54] as the graphical interface. The model was refined with ShelXL 2018/3[55] using full matrix least squares minimisation on $\boldsymbol{F}^2$.

Crystal Data. $C_{17}H_{19}NOF_2$, M_r = 291.33, onoclinic, *Ia* (No. 9), a = 9.67810(10) Å, b = 11.1140(10) Å, c = 14.2033(2) Å, β = 103.6270(10)°, α = γ = 90°, V = 1484.92(3) Å^3, T = 123.01(10) K, Z = 4, Z' = 1, μ (Cu K$_\alpha$) = 0.812, 14494 reflections measured, 2928 unique (R_{int} = 0.0215) which were used in all calculations. The final wR_2 was 0.0962 (all data) and R_1 was 0.0364 (I≥2 σ(I)).

**Compound**	**11d**
Formula	$C_{17}H_{19}NOF_2$
$D_{calc.}$/ g cm^{-3}	1.303
μ /mm^{-1}	0.812
Formula Weight	291.33
Colour	clear colourless
Shape	plate-shaped
Size/mm^3	0.22×0.09×0.02
T/K	123.01(10)
Crystal System	monoclinic
Flack Parameter	-0.02(4)
Hooft Parameter	-0.01(3)
Space Group	$I\alpha$
a /Å	9.67810(10)
b /Å	11.11540(10)
c /Å	14.2033(2)
α /°	90
β /°	103.6270(10)
γ /°	90
V / Å^3	1484.92(3)
Z	4
Z'	1
Wavelength/Å	1.54184
Radiation type	Cu K$_\alpha$
Θ_{min} /°	5.109
Θ_{max} /°	75.076
Measured Refl's.	14494
Indep't Refl's	2928
Refl's I≥2 σ(I)	2818
R_{int}	0.0215
Parameters	194
Restraints	2
Largest Peak	0.319
Deepest Hole	-0.313
GooF	1.038
wR_2 (all data)	0.0962
wR_2	0.0949
R_1 (all data)	0.0378
R_1	0.0364

Table S17: Fractional Atomic Coordinates (×10^4) and Equivalent Isotropic Displacement Parameters (Å^2×10^3) for **11d.** U_{eq} is defined as 1/3 of the trace of the orthogonalised U_{ij}.

Atom	x	y	z	U_{eq}
O1	2792.6(18)	5387.8(16)	5600.7(15)	36.8(5)
F2	5064(3)	296.4(17)	7072.6(17)	65.9(7)
F1	7195(3)	8366.2(18)	3548.6(19)	70.0(7)
N1	4955(2)	5538.6(17)	5249.1(15)	25.3(4)
C11	3984(2)	4970(2)	5619.6(18)	26.1(5)
C1	4685(2)	6643(2)	4658.4(16)	23.3(5)
C2	4257(3)	7703(2)	5218.1(17)	26.9(5)
C6	6088(3)	6972(2)	4387(2)	32.6(6)
C12	4378(2)	3747(2)	6043.2(18)	25.9(5)
C17	5183(2)	2945(2)	5635.5(18)	27.5(5)
C15	4830(3)	1438(3)	6733(2)	41.9(7)
C13	3807(3)	3360(3)	6804(2)	36.5(6)
C4	5447(3)	9141(2)	4306(2)	34.8(6)
C16	5412(3)	1783(2)	5980(2)	34.5(6)
C3	4039(3)	8832(2)	4576(2)	34.4(6)
C5	5873(3)	8092(3)	3765(2)	40.3(7)
C14	4041(4)	2203(3)	7161(2)	45.8(7)
C7	3536(3)	6417(3)	3731(2)	42.3(7)
C8	2896(4)	8595(3)	3660(3)	54.1(9)
C9	4765(5)	7863(3)	2837(2)	61.7(11)
C10	3361(5)	7552(3)	3101(2)	62.3(10)

Table S18: Anisotropic Displacement Parameters (×10^4) for **11d**. The anisotropic displacement factor exponent takes the form: $-2\pi^2[h^2a^{*2} \times U_{11}+ ... +2hka^* \times b^* \times U_{12}]$

Atom	U_{11}	U_{22}	U_{33}	U_{23}	U_{13}	U_{12}
O1	19.9(8)	27.7(9)	67.1(13)	-3.9(8)	19.2(8)	-1.4(6)
F2	93.9(17)	29.7(9)	87.6(16)	20.2(9)	48.2(14)	5.7(10)
F1	91.3(16)	44.5(11)	101.1(17)	20.0(11)	76.9(15)	12.3(10)
N1	19.6(9)	22.7(9)	36.1(10)	4.0(8)	11.4(8)	2.6(8)
C11	20.3(11)	24.7(11)	36.0(13)	-3.9(9)	12.1(9)	-3.1(9)
C1	24.4(11)	22.5(11)	24.0(11)	0.1(8)	7.9(9)	2.8(8)
C2	29.4(11)	24.7(12)	30.7(12)	-4.7(10)	15.2(9)	-0.9(9)
C6	36.2(13)	26.7(12)	42.9(14)	7.1(11)	25.9(11)	7.2(10)
C12	21.2(10)	24.2(12)	34.7(12)	-1.6(10)	11.3(9)	-4.5(9)
C17	24.4(11)	25.2(11)	35.6(12)	-2.0(10)	12.5(10)	-5.0(9)
C15	49.5(17)	25.6(13)	54.5(18)	6.5(12)	20.1(14)	-3.4(12)
C13	39.6(13)	31.4(13)	44.6(15)	-1.4(11)	22.1(12)	-4.2(11)
C4	43.0(14)	25.0(12)	41.4(14)	4.6(11)	19.9(11)	1.3(11)
C16	36.1(13)	24.2(13)	46.7(15)	-1.9(11)	17.0(12)	-1.7(10)
C3	36.5(14)	22.5(11)	48.7(15)	-1.6(11)	18.7(12)	5.4(10)
C5	59.0(17)	32.2(14)	40.7(15)	8.7(12)	33.9(14)	9.1(12)
C14	57.1(19)	38.6(16)	50.8(17)	7.3(14)	30.7(15)	-5.2(13)
C7	54.7(17)	33.0(15)	31.2(13)	-7.0(11)	-5.9(12)	-4.2(12)
C8	44.6(17)	42.5(18)	67(2)	18.4(15)	-3.1(15)	11.3(13)
C9	116(3)	47.2(18)	24.8(14)	5.8(12)	21.5(17)	10(2)
C10	87(3)	52(2)	30.4(15)	3.1(14)	-22.7(16)	-0.4(18)

Table S19: Bond Lengths in Å for **11d**.

Atom	Atom	Length/Å
O1	C11	1.238(3)
F2	C15	1.357(3)
F1	C5	1.418(4)
N1	C11	1.339(3)
N1	C1	1.475(3)
C11	C12	1.500(3)

Atom	Atom	Length/Å
C1	C2	1.532(3)
C1	C6	1.540(3)
C1	C7	1.531(3)
C2	C3	1.536(3)
C6	C5	1.512(4)
C12	C17	1.395(3)

Atom	Atom	Length/Å
C12	C13	1.393(3)
C17	C16	1.381(4)
C15	C16	1.376(4)
C15	C14	1.376(4)
C13	C14	1.382(4)
C4	C3	1.539(4)

Atom	Atom	Length/Å
C4	C5	1.506(4)
C3	C8	1.520(5)
C5	C9	1.512(5)
C7	C10	1.533(5)
C8	C10	1.531(6)
C9	C10	1.532(6)

Table S20: Bond Angles in ° for **11d**.

Atom	Atom	Atom	Angle/°
C11	N1	C1	124.72(19)
O1	C11	N1	123.5(2)
O1	C11	C12	119.8(2)
N1	C11	C12	116.75(19)
N1	C1	C2	112.23(18)
N1	C1	C6	106.92(18)
N1	C1	C7	110.4(2)
C2	C1	C6	108.1(2)
C7	C1	C2	110.0(2)
C7	C1	C6	109.1(2)
C1	C2	C3	109.67(19)
C5	C6	C1	109.5(2)
C17	C12	C11	121.8(2)
C13	C12	C11	118.7(2)
C13	C12	C17	119.2(2)
C16	C17	C12	120.6(2)
F2	C15	C16	118.2(3)
F2	C15	C14	119.0(3)
C14	C15	C16	122.8(3)
C14	C13	C12	120.7(3)

Atom	Atom	Atom	Angle/°
C5	C4	C3	108.6(2)
C15	C16	C17	118.4(2)
C2	C3	C4	108.9(2)
C8	C3	C2	109.7(2)
C8	C3	C4	109.5(2)
F1	C5	C6	107.3(2)
F1	C5	C4	108.3(2)
F1	C5	C9	109.8(3)
C4	C5	C6	110.9(2)
C4	C5	C9	110.6(3)
C9	C5	C6	110.0(3)
C15	C14	C13	118.3(3)
C1	C7	C10	109.0(2)
C3	C8	C10	109.4(2)
C5	C9	C10	108.2(2)
C8	C10	C7	109.0(3)
C8	C10	C9	110.0(3)
C9	C10	C7	110.3(3)

Table S21: Torsion Angles in ° for **11d**.

Atom	Atom	Atom	Atom	Angle/°
O1	C11	C12	C17	141.4(2)
O1	C11	C12	C13	-32.1(3)
F2	C15	C16	C17	-179.7(2)
F2	C15	C14	C13	-179.7(3)
F1	C5	C9	C10	179.1(3)
N1	C11	C12	C17	-36.5(3)
N1	C11	C12	C13	150.0(2)
N1	C1	C2	C3	178.14(19)
N1	C1	C6	C5	179.2(2)
N1	C1	C7	C10	-175.9(3)
C11	N1	C1	C2	62.0(3)
C11	N1	C1	C6	-179.7(2)
C11	N1	C1	C7	-61.1(3)
C11	C12	C17	C16	-173.5(2)
C11	C12	C13	C14	174.4(3)
C1	N1	C11	O1	-8.7(4)
C1	N1	C11	C12	169.1(2)
C1	C2	C3	C4	-61.1(3)
C1	C2	C3	C8	58.7(3)
C1	C6	C5	F1	179.1(2)
C1	C6	C5	C4	61.1(3)
C1	C6	C5	C9	-61.6(3)
C1	C7	C10	C8	-61.0(4)
C1	C7	C10	C9	59.9(3)
C2	C1	C6	C5	-59.8(3)

Atom	Atom	Atom	Atom	Angle/˚
C2	C1	C7	C10	59.8(3)
C2	C3	C8	C10	-60.4(3)
C6	C1	C2	C3	60.5(3)
C6	C1	C7	C10	-58.6(3)
C6	C5	C9	C10	61.3(3)
C12	C17	C16	C15	-0.1(4)
C12	C13	C14	C15	-1.2(5)
C17	C12	C13	C14	0.6(4)
C13	C12	C17	C16	0.0(4)
C4	C3	C8	C10	59.1(3)
C4	C5	C9	C10	-61.5(3)
C16	C15	C14	C13	1.1(5)
C3	C4	C5	F1	-178.0(2)
C3	C4	C5	C6	-60.6(3)
C3	C4	C5	C9	61.7(3)
C3	C8	C10	C7	61.6(4)
C3	C8	C10	C9	-59.4(3)
C5	C4	C3	C2	59.9(3)
C5	C4	C3	C8	-60.0(3)
C5	C9	C10	C7	-60.6(3)
C5	C9	C10	C8	59.6(3)
C14	C15	C16	C17	-0.5(5)
C7	C1	C2	C3	-58.6(3)
C7	C1	C6	C5	59.8(3)

Table S22: Hydrogen Fractional Atomic Coordinates (×10^4) and Equivalent Isotropic Displacement Parameters (Å^2×10^3) for **11d**. U_{eq} is defined as 1/3 of the trace of the orthogonalised U_{ij}.

Atom	x	y	z	U_{eq}
H2A	5008.81	7853.6	5811.98	32
H2B	3365.46	7508.67	5413.4	32
H6A	6839.88	7114.98	4982.05	39
H6B	6393.93	6298.04	4027.4	39
H17	5576.04	3201.86	5116.19	33
H13	3250.04	3898.37	7081.3	44
H4A	5325.91	9870.51	3894.49	42
H4B	6195.63	9303.03	4899.33	42
H16	5958.26	1235.15	5703	41
H3	3743.75	9520.06	4938.81	41
H14	3667.38	1941.49	7687.54	55
H7A	2624.49	6217.37	3896.52	51
H7B	3813.08	5729.5	3372.14	51
H8A	2742.78	9325.89	3250.44	65
H8B	1989.4	8390.56	3830.34	65
H9A	5066.87	7189.32	2476.38	74
H9B	4643.49	8588.89	2421.46	74
H10	2613.2	7406.2	2493.79	75
H1	5890(30)	5240(30)	5400(20)	31(7)

Crystal data for 2o:

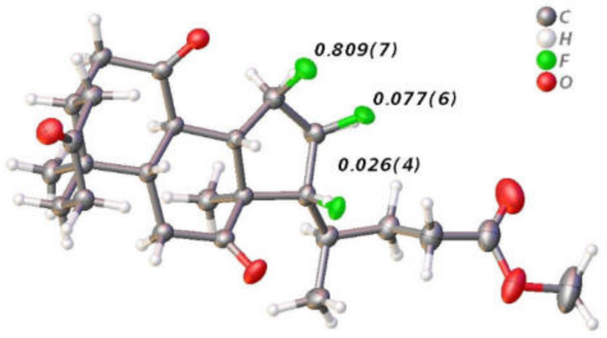

Experimental. Single clear colourless needle-shaped crystals of **2o** (**CCDC 2212438**) were used as supplied. A suitable crystal with dimensions 0.26 × 0.03 × 0.03 mm^3 was selected and mounted on a MITIGEN holder with mineral oil on a XtaLAB Synergy R, DW system, HyPix-Arc 150 diffractometer. The crystal was kept at a steady T = 123.00(10) K during data collection. The structure was solved with the ShelXT 2018/2 solution program[53] using dual methods and by using Olex2 1.5-alpha[54] as the graphical interface. The model was refined with olex2.refine 1.5-alpha[56] using full matrix least squares minimisation on $\boldsymbol{F}^2$.

Crystal Data. $C_{25}H_{35.09}F_{0.92}O_5$, M_r = 433.023, monoclinic, $P2_1$ (No. 4), a = 10.4636(4) Å, b = 6.90652(18) Å, c = 16.1323(6) Å, β = 108.458(4)°, $\alpha = \gamma$ = 90°, V = 1105.87(7) Å^3, T = 123.00(10) K, Z = 2, Z' = 1, μ(Cu K$_\alpha$) = 0.768, 11859 reflections measured, 3857 unique (R_{int} = 0.0407) which were used in all calculations. The final wR_2 was 0.1052 (all data) and R_1 was 0.0443 (I≥2 σ(I)).

**Compound**	**2o**
Formula	$C_{25}H_{35.09}F_{0.92}O_5$
$D_{calc.}$/ g cm^{-3}	1.300
μ /mm^{-1}	0.768
Formula Weight	433.023
Colour	clear colourless
Shape	needle-shaped
Size/mm^3	0.26×0.03×0.03
T/K	123.00(10)
Crystal System	monoclinic
Flack Parameter	0.12(12)
Hooft Parameter	0.12(12)
Space Group	$P2_1$
a/Å	10.4636(4)
b/Å	6.90652(18)
c/Å	16.1323(6)
α/°	90
β/°	108.458(4)
γ/°	90
V/Å^3	1105.87(7)
Z	2
Z'	1
Wavelength/Å	1.54184
Radiation type	Cu K$_\alpha$
Θ_{min}/°	2.89
Θ_{max}/°	75.16
Measured Refl's.	11859
Indep't Refl's	3857
Refl's I≥2 σ(I)	2981
R_{int}	0.0407
Parameters	413
Restraints	13
Largest Peak	0.2077
Deepest Hole	-0.2155
GooF	1.0399
wR_2 (all data)	0.1052
wR_2	0.0947
R_1 (all data)	0.0666
R_1	0.0443

Table S23: Fractional Atomic Coordinates (×10^4) and Equivalent Isotropic Displacement Parameters (Å^2×10^3) for **2o**. U_{eq} is defined as 1/3 of the trace of the orthogonalised U_{ij}.

Atom	x	y	z	U_{eq}
O^{001}	5737.0(18)	619(3)	5435.2(12)	28.6(5)
F^{002}	8303.5(18)	2380(3)	6229.2(12)	29.9(7)
O^{003}	6025.8(19)	7502(3)	7479.8(14)	32.7(5)
O^{004}	255(2)	3564(3)	3842.6(14)	35.6(5)
O^{005}	12631(2)	9942(4)	9138.7(15)	51.6(7)
C^{006}	5029(3)	689(4)	5902.4(17)	20.9(6)
C^{007}	6906(3)	4253(4)	7796.3(18)	20.6(6)
O^{008}	13300(2)	7021(5)	8822.9(18)	66.8(9)
C^{009}	4128(3)	3428(4)	6541.0(18)	20.6(6)
C^{00A}	5805(3)	5773(4)	7537.4(17)	23.6(6)
C^{00B}	6643(2)	3089(4)	6923.5(17)	20.8(6)
C^{00C}	1198(3)	3501(4)	4516.9(18)	25.3(6)
C^{00D}	1393(3)	4916(5)	5253(2)	27.2(6)
C^{00E}	8379(2)	4943(4)	7975.8(18)	22.7(6)
C^{00F}	2753(2)	2368(5)	6354.6(17)	22.0(6)
C^{00G}	2543(3)	956(4)	5580.8(18)	22.5(6)
C^{00H}	5327(3)	1960(4)	6695.7(17)	20.4(6)
C^{00I}	6778(3)	2984(5)	8548.9(19)	26.9(7)
C^{00J}	1578(3)	3834(5)	6110(2)	26.2(7)
C^{00K}	9122(3)	5699(5)	8899.2(19)	26.6(6)
C^{00L}	3741(3)	-446(4)	5711(2)	25.0(6)
C^{00M}	4389(3)	4964(5)	7267.6(19)	23.8(6)
C^{00N}	2678(3)	1257(5)	7162(2)	29.4(7)
C^{00O}	12406(3)	8146(6)	8810(2)	43.2(9)
C^{00P}	2262(3)	1949(5)	4685.8(18)	25.3(6)
C^{00Q}	7983(3)	2059(5)	7021.5(18)	29.0(7)
C^{00R}	9051(3)	3120(5)	7746(2)	28.4(7)
C^{00S}	10622(3)	6089(5)	9047.4(19)	28.9(7)
C^{00T}	10932(3)	7692(5)	8482(2)	38.3(8)
C^{00U}	8448(3)	7451(6)	9164(2)	37.2(8)
C^{00V}	14037(4)	10514(7)	9487(3)	76.3(16)
F^{4}	9904(19)	3696(8)	7299(14)	32(7)
F^{1}	8360(50)	6555(8)	7360(30)	23(6)

Table S24: Anisotropic Displacement Parameters (×10^4) for **2o**. The anisotropic displacement factor exponent takes the form: $-2\pi^2[h^2a^{*2} \times U_{11} + ... +2hka^* \times b^* \times U_{12}]$

Atom	U_{11}	U_{22}	U_{33}	U_{23}	U_{13}	U_{12}
O^{001}	29.1(10)	26.0(12)	30.3(11)	-1.0(9)	8.8(9)	-5.6(10)
F^{002}	24.7(11)	38.7(14)	27.3(12)	0.9(9)	9.9(8)	-5.7(11)
O^{003}	26.8(10)	16.5(11)	45.8(13)	-0.6(9)	-1.2(9)	0.8(11)
O^{004}	31.1(11)	35.7(14)	30.6(11)	2.2(10)	-3.5(9)	2.2(11)
O^{005}	53.4(15)	53.7(18)	40.9(14)	-27.2(13)	5.2(12)	2.9(14)
C^{006}	21.6(13)	14.9(14)	22.1(13)	4.4(11)	1.2(11)	-0.6(12)
C^{007}	20.1(14)	17.4(15)	21.3(13)	2.1(10)	2.4(11)	-0.1(12)
O^{008}	35.7(14)	89(2)	72.7(19)	0.5(15)	12.9(14)	-4.3(19)
C^{009}	19.6(13)	20.0(15)	20.7(14)	-0.4(11)	4.2(11)	0.6(13)
C^{00A}	24.7(14)	24.6(16)	19.8(14)	0.4(12)	4.6(11)	-4.3(13)
C^{00B}	18.2(12)	21.4(15)	20.2(14)	0.4(11)	2.2(11)	-1.5(12)
C^{00C}	23.4(14)	24.8(17)	26.2(15)	-1.9(12)	5.5(13)	5.7(13)
C^{00D}	22.6(15)	24.2(16)	32.4(16)	4.1(13)	5.0(12)	2.6(15)
C^{00E}	17.8(13)	20.9(15)	26.7(14)	-2.5(11)	3.3(11)	1.1(13)
C^{00F}	19.8(13)	23.6(16)	21.0(13)	-2.8(12)	4.4(10)	-0.0(13)
C^{00G}	20.4(14)	21.0(16)	23.0(14)	-2.4(12)	2.6(11)	1.3(13)
C^{00H}	20.9(13)	17.5(15)	19.4(13)	-0.5(11)	1.8(11)	-0.3(12)
C^{00I}	27.2(16)	28.6(18)	22.3(15)	-1.5(13)	4.3(13)	-0.2(13)
C^{00J}	20.6(14)	28.7(18)	29.5(16)	-1.0(12)	8.3(13)	-4.8(14)
C^{00K}	26.6(14)	24.3(16)	23.7(14)	-2.0(12)	0.4(12)	-0.3(14)
C^{00L}	26.7(15)	18.4(16)	24.6(15)	-1.5(12)	0.8(12)	0.7(14)

Atom	U_{11}	U_{22}	U_{33}	U_{23}	U_{13}	U_{12}
C^{00M}	22.1(14)	21.2(16)	26.2(15)	3.1(12)	5.0(12)	-0.9(14)
C^{00N}	24.1(15)	35(2)	26.5(16)	-8.1(14)	5.1(13)	1.1(15)
C^{00O}	39.4(19)	55(3)	33.7(18)	-14.3(18)	9.4(15)	4.8(18)
C^{00P}	27.1(15)	24.6(17)	22.3(15)	0.5(12)	5.2(12)	2.0(13)
C^{00Q}	23.8(14)	29.4(18)	32.5(16)	0.3(12)	7.2(12)	-4.7(15)
C^{00R}	21.3(14)	28.8(17)	32.4(16)	2.1(12)	4.7(12)	-3.3(14)
C^{00S}	26.3(15)	30.1(19)	23.7(15)	-2.7(13)	-1.3(12)	1.2(14)
C^{00T}	33.5(17)	39(2)	37.3(18)	-6.7(15)	3.9(15)	8.8(17)
C^{00U}	31.9(17)	37(2)	38.7(19)	-4.2(15)	5.1(15)	-15.3(18)
C^{00V}	59(3)	101(4)	57(3)	-46(3)	1(2)	11(3)
F^{4}	28(9)	40(11)	34(9)	-3(4)	18(4)	-11(5)
F^{1}	17(10)	19(10)	28(10)	-6(5)	1(5)	2(5)

Table S25: Bond Lengths in Å for **2o**.

Atom	Atom	Length/Å
O^{001}	C^{006}	1.213(3)
F^{002}	C^{00Q}	1.437(3)
O^{003}	C^{00A}	1.225(4)
O^{004}	C^{00C}	1.216(3)
O^{005}	C^{00O}	1.340(4)
O^{005}	C^{00V}	1.453(4)
C^{006}	C^{00H}	1.501(4)
C^{006}	C^{00L}	1.505(4)
C^{007}	C^{00A}	1.516(4)
C^{007}	C^{00B}	1.568(4)
C^{007}	C^{00E}	1.551(4)
C^{007}	C^{00I}	1.537(4)
O^{008}	C^{00O}	1.211(4)
C^{009}	C^{00F}	1.557(4)
C^{009}	C^{00H}	1.570(4)
C^{009}	C^{00M}	1.540(4)
C^{00A}	C^{00M}	1.513(4)
C^{00B}	C^{00H}	1.523(4)
C^{00B}	C^{00Q}	1.535(4)
C^{00C}	C^{00D}	1.501(4)
C^{00C}	C^{00P}	1.507(4)
C^{00D}	C^{00J}	1.529(4)
C^{00E}	C^{00K}	1.537(4)
C^{00E}	C^{00R}	1.544(4)
C^{00E}	F^{1}	1.49(3)
C^{00F}	C^{00G}	1.544(4)
C^{00F}	C^{00J}	1.545(4)
C^{00F}	C^{00N}	1.535(4)
C^{00G}	C^{00L}	1.545(4)
C^{00G}	C^{00P}	1.540(4)
C^{00K}	C^{00S}	1.535(4)
C^{00K}	C^{00U}	1.528(5)
C^{00O}	C^{00T}	1.497(4)
C^{00Q}	C^{00R}	1.524(4)
C^{00R}	F^{4}	1.372(16)
C^{00S}	C^{00T}	1.533(4)

Table S26: Bond Angles in ° for **2o**.

Atom	Atom	Atom	Angle/°
C^{00V}	O^{005}	C^{00O}	115.8(3)
C^{00H}	C^{006}	O^{001}	123.4(3)
C^{00L}	C^{006}	O^{001}	122.4(3)
C^{00L}	C^{006}	C^{00H}	114.2(2)
C^{00B}	C^{007}	C^{00A}	101.9(2)
C^{00E}	C^{007}	C^{00A}	117.5(2)
C^{00E}	C^{007}	C^{00B}	101.9(2)
C^{00I}	C^{007}	C^{00A}	111.4(2)
C^{00I}	C^{007}	C^{00B}	112.5(2)
C^{00I}	C^{007}	C^{00E}	110.9(2)
C^{00H}	C^{009}	C^{00F}	111.7(2)
C^{00M}	C^{009}	C^{00F}	113.7(2)
C^{00M}	C^{009}	C^{00H}	112.6(2)
C^{007}	C^{00A}	O^{003}	123.4(2)
C^{00M}	C^{00A}	O^{003}	121.9(3)
C^{00M}	C^{00A}	C^{007}	114.4(3)
C^{00H}	C^{00B}	C^{007}	111.6(2)
C^{00Q}	C^{00B}	C^{007}	104.5(2)
C^{00Q}	C^{00B}	C^{00H}	121.0(2)
C^{00D}	C^{00C}	O^{004}	123.6(3)
C^{00P}	C^{00C}	O^{004}	122.0(3)
C^{00P}	C^{00C}	C^{00D}	114.4(2)
C^{00J}	C^{00D}	C^{00C}	110.1(3)
C^{00K}	C^{00E}	C^{007}	116.9(2)
C^{00R}	C^{00E}	C^{007}	101.9(2)
C^{00R}	C^{00E}	C^{00K}	112.4(2)
F^{1}	C^{00E}	C^{007}	107.6(19)
F^{1}	C^{00E}	C^{00K}	106.9(17)
F^{1}	C^{00E}	C^{00R}	111.1(16)
C^{00G}	C^{00F}	C^{009}	109.8(2)
C^{00J}	C^{00F}	C^{009}	110.7(2)
C^{00J}	C^{00F}	C^{00G}	108.0(2)
C^{00N}	C^{00F}	C^{009}	111.3(2)
C^{00N}	C^{00F}	C^{00G}	109.8(3)
C^{00N}	C^{00F}	C^{00J}	107.1(2)
C^{00L}	C^{00G}	C^{00F}	112.6(2)
C^{00P}	C^{00G}	C^{00F}	114.4(2)
C^{00P}	C^{00G}	C^{00L}	108.2(2)
C^{009}	C^{00H}	C^{006}	107.6(2)
C^{00B}	C^{00H}	C^{006}	115.6(2)
C^{00B}	C^{00H}	C^{009}	108.9(2)
C^{00F}	C^{00J}	C^{00D}	114.4(2)

Atom	Atom	Atom	Angle/°
C^{00S}	C^{00K}	C^{00E}	112.9(2)
C^{00U}	C^{00K}	C^{00E}	113.8(3)
C^{00U}	C^{00K}	C^{00S}	110.9(3)
C^{00G}	C^{00L}	C^{006}	109.7(2)
C^{00A}	C^{00M}	C^{009}	113.3(2)
O^{008}	C^{00O}	O^{005}	123.3(3)
C^{00T}	C^{00O}	O^{005}	111.5(3)
C^{00T}	C^{00O}	O^{008}	125.1(4)
C^{00G}	C^{00P}	C^{00C}	113.4(2)
C^{00B}	C^{00Q}	F^{002}	107.4(2)
C^{00R}	C^{00Q}	F^{002}	106.7(2)
C^{00R}	C^{00Q}	C^{00B}	106.3(2)
C^{00Q}	C^{00R}	C^{00E}	107.3(2)
F^{4}	C^{00R}	C^{00E}	108.1(4)
F^{4}	C^{00R}	C^{00Q}	100.2(9)
C^{00T}	C^{00S}	C^{00K}	115.6(3)
C^{00S}	C^{00T}	C^{00O}	109.2(3)

Table S27: Torsion Angles in ° for **2o**.

Atom	Atom	Atom	Atom	Angle/°
O^{001}	C^{006}	C^{00H}	C^{009}	118.0(3)
O^{001}	C^{006}	C^{00H}	C^{00B}	-3.9(3)
O^{001}	C^{006}	C^{00L}	C^{00G}	-119.3(3)
F^{002}	C^{00Q}	C^{00B}	C^{007}	-132.2(2)
F^{002}	C^{00Q}	C^{00B}	C^{00H}	101.1(2)
F^{002}	C^{00Q}	C^{00R}	C^{00E}	106.2(2)
F^{002}	C^{00Q}	C^{00R}	F^{4}	-6.6(5)
O^{003}	C^{00A}	C^{007}	C^{00B}	-111.7(3)
O^{003}	C^{00A}	C^{007}	C^{00E}	-1.4(3)
O^{003}	C^{00A}	C^{007}	C^{00I}	128.2(3)
O^{003}	C^{00A}	C^{00M}	C^{009}	120.6(3)
O^{004}	C^{00C}	C^{00D}	C^{00J}	127.0(3)
O^{004}	C^{00C}	C^{00P}	C^{00G}	-130.4(3)
O^{005}	C^{00O}	C^{00T}	C^{00S}	-114.0(3)
C^{006}	C^{00H}	C^{009}	C^{00F}	57.1(2)
C^{006}	C^{00H}	C^{009}	C^{00M}	-173.6(2)
C^{006}	C^{00H}	C^{00B}	C^{007}	-176.0(2)
C^{006}	C^{00H}	C^{00B}	C^{00Q}	-52.6(3)
C^{006}	C^{00L}	C^{00G}	C^{00F}	-53.8(3)
C^{006}	C^{00L}	C^{00G}	C^{00P}	73.6(3)
C^{007}	C^{00A}	C^{00M}	C^{009}	-53.8(2)
C^{007}	C^{00B}	C^{00H}	C^{009}	62.7(2)
C^{007}	C^{00B}	C^{00Q}	C^{00R}	-18.3(2)
C^{007}	C^{00E}	C^{00K}	C^{00S}	172.3(3)
C^{007}	C^{00E}	C^{00K}	C^{00U}	-60.1(3)
C^{007}	C^{00E}	C^{00R}	C^{00Q}	31.6(2)
C^{007}	C^{00E}	C^{00R}	F^{4}	138.9(10)
O^{008}	C^{00O}	C^{00T}	C^{00S}	62.7(4)
C^{009}	C^{00F}	C^{00G}	C^{00L}	53.0(3)
C^{009}	C^{00F}	C^{00G}	C^{00P}	-71.1(2)
C^{009}	C^{00F}	C^{00J}	C^{00D}	65.2(2)
C^{009}	C^{00H}	C^{00B}	C^{00Q}	-173.8(2)
C^{00B}	C^{00Q}	C^{00R}	C^{00E}	-8.2(2)
C^{00B}	C^{00Q}	C^{00R}	F^{4}	-121.0(5)
C^{00C}	C^{00D}	C^{00J}	C^{00F}	56.7(3)
C^{00C}	C^{00P}	C^{00G}	C^{00F}	-48.0(3)
C^{00C}	C^{00P}	C^{00G}	C^{00L}	-174.4(2)
C^{00D}	C^{00J}	C^{00F}	C^{00G}	-55.0(3)
C^{00D}	C^{00J}	C^{00F}	C^{00N}	-173.2(3)
C^{00E}	C^{00K}	C^{00S}	C^{00T}	64.1(3)
C^{00K}	C^{00S}	C^{00T}	C^{00O}	169.8(3)

Table S28: Hydrogen Fractional Atomic Coordinates (×10[4]) and Equivalent Isotropic Displacement Parameters (Å[2]×10[3]) for **2o**. U_{eq} is defined as 1/3 of the trace of the orthogonalised U_{ij}.

Atom	x	y	z	U_{eq}
H^{009}	4090(30)	4040(50)	6026(19)	24(8)
H^{00B}	6570(20)	4150(40)	6443(17)	15(7)
H^{00a}	2170(30)	5730(50)	5278(18)	28(8)
H^{00c}	610(20)	5870(40)	5130(16)	13(6)
H^{00E}	8386(2)	5983(4)	7546.0(18)	27.2(7)
H^{00G}	1710(30)	230(50)	5557(19)	31(8)
H^{00H}	5300(30)	1120(50)	7174(19)	25(8)
H^{00d}	6950(30)	3710(60)	9080(20)	47(10)
H^{00f}	5880(30)	2440(50)	8430(19)	32(8)
H^{00i}	7480(30)	1840(60)	8670(20)	40(9)
H^{00j}	1700(30)	4700(60)	6600(20)	42(10)
H^{00k}	730(30)	3110(50)	6040(20)	39(9)
H^{00l}	9140(30)	4600(50)	9310(20)	33(8)
H^{00m}	3560(30)	-1230(60)	5140(20)	44(10)
H^{00n}	3840(30)	-1250(50)	6207(19)	22(8)
H^{00o}	3740(30)	6040(50)	7074(18)	22(7)
H^{00p}	4300(30)	4480(50)	7820(20)	36(9)
H^{00q}	3440(30)	390(50)	7430(20)	37(9)
H^{00r}	2600(30)	2190(50)	7660(20)	35(8)
H^{00s}	1830(30)	630(50)	6989(19)	30(8)
H^{00t}	1910(30)	950(50)	4168(19)	24(7)
H^{00u}	3060(30)	2580(50)	4607(18)	28(8)
H^{00v}	7933(3)	683(5)	7179.7(18)	34.8(8)
H^{00w}	8200(3)	2121(5)	6467.5(18)	34.8(8)
H^{00y}	9818(3)	3491(5)	7544(2)	34.1(8)
H^{00x}	9392(3)	2276(5)	8265(2)	34.1(8)
H^{00z}	11050(30)	6470(50)	9700(20)	34.6(8)
H	11170(30)	4810(50)	8960(19)	34.6(8)
H^{00}	10380(30)	9010(60)	8470(20)	56(11)
H^{a}	10660(30)	7170(50)	7840(20)	33(8)
H^{1}	8520(40)	8530(70)	8800(30)	62(13)
H^{b}	7400(30)	7200(60)	9160(20)	41(9)
H^{c}	9020(30)	7780(60)	9810(20)	51(10)
H^{2}	14602(16)	9880(40)	9088(16)	114(2)
H^{d}	14111(5)	12090(40)	9475(17)	114(2)
H^{e}	14455(15)	10000(40)	10160(18)	114(2)

Table S29: Atomic Occupancies for all atoms that are not fully occupied in **2o**.

Atom	Occupancy
F^{002}	0.808(6)
H^{00E}	0.973(4)
H^{00w}	0.192(6)
H^{00y}	0.922(6)
F^{4}	0.078(6)
F^{1}	0.027(4)

6 References

[1] C. Liu, J. Wang, L. Meng, Y. Deng, Y. Li and A. Lei, *Angew. Chem. Int. Ed.,* 2011, **51**, 5144.

[2] T. Mukaiyama, T. Shintou and K. Fukumoto, *J. Am. Chem. Soc.,* 2003, **125**, 10538.

[3] V. Valerio, D. Petkova, C. Madelaine and N. Maulide, *Chem. Eur. J.,* 2013, **19**, 2606.

[4] K. Asano and S. Matsubara, *Org. Lett.,* 2009*,* **11**, 1757.

[5] S. Guo, X. Zhang and P. Tang, *Angew. Chem. Int. Ed.,* 2015*,* 54, 4065.

[6] X. F. Wu and C. Darcel, *Eur. J. Org. Chem.,* 2009, **2009**, 1144.

[7] S. Kamijo, Y. Amaoka and M. Inoue, *Tetrahedron Lett.,* 2011*,* **52**, 4654.

[8] a) I. Pravst, M. Zupan, S. Stavber, *Tetrahedron*, 2008, **64**, 5191; b) J. C. Lee, Y. H. Bae and S. K. Chang, *Bull. Korean Chem. Soc.,* 2003, **24**, 407.

[9] X. Tian, T. A. Karl, S. Reiter, S. Yakubov, R. de Vivie-Riedle, B. König and J. P. Barham, *Angew. Chem. Int. Ed.,* 2021, **60**, 20817.

[10] S. Ikonen, Nonappa, A. Valkonen, R. Juvonen, H. Salo and E. Kolehmainen, *Org. Biomol. Chem.,* 2010, **8**, 2784.

[11] M. A. Maslov, N. G. Morozova, T. V. Solomatina, N. G. Shaforostova and G. A. Serebrennikova, *Russ. J. Bioorg. Chem.,* 2011, **37**, 507.

[12] H. Fujioka, A. Goto, K. Otake, O. Kubo, Y. Sawama and T. Maegawa, *Chem. Commun.,* 2011, **47**, 9894.

[13] A. Dasgupta, R. Babaahmadi, S. Pahar, K. Stefkova, L. Gierlichs, B. F. Yates, A. Ariafard and R. L. Melen, *Angew. Chem. Int. Ed.,* 2021, **60**, 24395.

[14] Y. Peng, Y. Y. Jiang, X. J. Du, D. Y. Ma and L. Yang, *Org. Chem. Front.,* 2019, **6**, 3065.

[15] C. H. Xing and Q. S. Hu, *Tetrahedron Lett.,* 2010, **51**, 924.

[16] H. Masuno, Y. Kazui, A. Tanatani, S. Fujii, E. Kawachi, T. Ikura, N. Ito, K. Yamamoto and H. Kagechika, *Bioorg. Med. Chem.,* 2019, **27**, 3674.

[17] P. Mampuys, E. Ruijter, R. V. Orru and B. U. Maes, *Org. Lett.,* 2018, **20**, 4235.

[18] Y. Zhu, J. Shi and W. Yu, *Org. Lett.,* 2020, **22**, 8899.

[19] A. Schuhmacher, T. Shiro, S. J. Ryan and J. W. Bode, *Chem. Sci.,* 2020, **11**, 7609.

[20] Z. L. Li, K. K. Sun and C. Cai, *Org. Lett.,* 2018, **20**, 6420.

[21] R. A. Olofson, J. T. Martz, J. P. Senet, M. Piteau and T. Malfroot, *J. Org. Chem.,* 1984, **49**, 2081.

[22] a) S. G. Senderoff, S. W. Landvatter and J. R. Heys, *J. Labelled Compd. Radiopharm.,* 2000, **43**, 1283; b) Y. F.Liang, N. Jiao, *Angew. Chem. Int. Ed.* 2014, **53**, 548.

[23] a) M. Domański, J. Žurauskas, J. P. Barham, *Org. Process Res. Dev.* 2022, **26**, 2498; b) T. Zeng, G. Song, C. J. Li, *Chem. Comm.* 2009, 6249; c) M. Szostak, M. Spain, A. J. Eberhart and D. J. Procter, *J. Am. Chem. Soc.,* 2014, **136**, 2268.

[24] N. Ilayaraja and M. Noel, *J. Electroanalytic Chem.,* 2010, **638**, 39.

[25] J. B. Xia, C. Zhu and C. Chen, *Chem. Comm.,* 2014, **50**, 11701.

[26] S. Bloom, C. R. Pitts, R. Woltornist, A. Griswold, M. G. Holl and T. Lectka, *Org. Lett.,* 2013, **15**, 1722.

[27] S. Bloom, M. McCann and T. Lectka, *Org. Lett.,* 2014, **16**, 6338.

[28] a) C. W. Kee, K. F. Chin, M. W. Wong and C. H. Tan, *Chem. Comm.,* 2014, **50**, 8211; b) J. W. Kee, H. Shao, C. W. Kee, Y. Lu, H. S. Soo and C. H. Tan, *Catal. Sci. Technol.,* 2017, **7**, 848.

[29] W. Knoll, J. L. Mieusset, V. B. Arion, L. Brecker and U. H. Brinker, *Org. Lett.,* 2010, **12**, 2366.

[30] M. Aoyama, T. Fukuhara and S. Hara, *J. Org. Chem.,* 2008, **73**, 4186.

[31] a) Z. Lu, X. Zeng, G. B. Hammond and B. Xu, *J. Am. Chem. Soc.,* 2017, **139**, 18202; b) Y. Amaoka, M. Nagatomo, M. Inoue, *Org. lett.* **2013**, *15*, 2160.

[32] D. R. Arnold, J. R. Bolton, G. E. Palmer and K. V. Prabhu, *Canad. J. Chem*., 1977, **55**, 2728.

[33] Y. Kanda, R. Shimada and Y. Takenoshita, *Spectrochimica Acta,* 1963, **19**, 1249.

[34] F. J. Aguilar Troyano, K. Merkens and A. Gómez-Suárez, *Asian J. Org. Chem.,* 2020, **9**, 992.

[35] H. Egami, S. Masuda, Y. Kawato and Y. Hamashima, *Org. Lett.,* 2018, **20**, 1367.

[36] N. J. Green, S. M. Pimblott and M. Tachiya, *J. Phys. Chem.,* 1993, **97**, 196.

[37] C. Feldmeier, H. Bartling, E. Riedle and R. M. Gschwind, *J. Magn. Reson.,* 2013, **232**, 39.

[38] A. Jerschow and N. Müller, *J. Magn. Reson.,* 1997, **125**, 372.

[39] A. Bondi, *J. Phys. Chem.,* 1964, **68**, 441.

[40] M. Mantina, A. C. Chamberlin, R. Valero, C. J. Cramer and D. G. Truhlar, *J. Phys. Chem. A,* 2009, **113**, 5806.

[41] a) V. Barone and M. Cossi, *J. Phys. Chem. A,* 1998, **102**, 1995; b) W. Kohn and L. J. Sham. *Phys. Rev.,* 1965, **140**, A1133.

[42] Gaussian 09, Revision D.01, M. J. Frisch, G. W. Trucks, H. B. Schlegel, G. E. Scuseria, M. A. Robb, J. R. Cheeseman, G. Scalmani, V. Barone, G. A. Petersson, H. Nakatsuji, X. Li, M. Caricato, A. Marenich, J. Bloino, B. G. Janesko, R. Gomperts, B. Mennucci, H. P. Hratchian, J. V. Ortiz, A. F. Izmaylov, J. L. Sonnenberg, D. Williams-Young, Young, F. Ding, F. Lipparini, F. Egidi, J. Goings, B. Peng, A. Petrone, T. Henderson, D. Ranasinghe, V. G. Zakrzewski, J. Gao, N. Rega, G. Zheng, W. Liang, M. Hada, M. Ehara, K. Toyota, R. Fukuda, J. Hasegawa, M. Ishida, T. Nakajima, Y. Honda, O. Kitao, H. Nakai, T. Vreven, K. Throssell, J. A. Montgomery, Jr., J. E. Peralta, F. Ogliaro, M. Bearpark, J. J. Heyd, E. Brothers, K. N. Kudin, V. N. Staroverov, T. Keith, R. Kobayashi, J. Normand, K. Raghavachari, A. Rendell, J. C. Burant, S. S. Iyengar, J. Tomasi, M. Cossi, J. M. Millam, M. Klene, C. Adamo, R. Cammi, J. W. Ochterski, R. L. Martin, K. Morokuma, O. Farkas, J. B. Foresman, and D. J. Fox, Gaussian Inc., Wallingford CT, 2, 2016.

[43] T. Yanai, D. P. Tew and N. C. Handy, *Chem. Phys. Lett.,* 2004, **393**, 51.

[44] a) J. D. Chai and M. H. Gordon, *Phys. Chem. Chem. Phys.,* 2008, **10**, 6615; b) Y. Zhao and D.G. Truhlar, *J. Chem. Phys.,* 2006, 194101; c) Y. Zhao and D. G. Truhlar, *Acc. Chem. Res.,* 2008, **41**, 157.

[45] a) R. Krishnan, J. S. Binkley, R. Seeger and J. A. Pople, *J. Chem. Phys.,* 1980, **72,** 650; b) M. J. Frisch, J. A. People and J. S. Binkley, *J. Chem. Phys.,* 1984, **80**, 3265; c) A. D. McLean and G. S. Chandler, *J. Chem. Phys.,* 1980, **72**, 5639.

[46] M. Cossi, N. Rega, G. Scalmani and V. Barone, *J. Comput. Chem.,* 2003, **24**. 669.

[47] C. Adamo and D. Jacquemin, *Chem. Soc. Rev.,* 2013, **42**, 845.
[48] E. Runge and E. K. U. Gross, *Phys. Rev. Lett.,* 1984, **52**, 997.
[49] M. A. Cismesia and T. P. Yoon, *Chem. Sci*., 2015, **6**, 5426.
[50] U. Megerle, R. Lechner, B. König and E. Riedle, *Photochem. Photobiol. Sci.,* 2010, **9**, 1400.
[51] a) *SCALE3ABS, CrysAlisPro, Agilent Technologies Inc.*, Oxford, U.K., 2015; b) G. M. Sheldrick, SADABS, *Bruker AXS*, Madison, U.S.A., 2007.
[52] R. C. Clark, J. S. Reid, *Acta Crystallogr. A. Found Crystallogr.* 1995, **51**, 887.
[53] G. M. Sheldrick, *Acta Cryst.*, 2015, **C71**, 3.
[54] O. V. Dolomanov, L. J. Bourhis, R. J. Gildea, J. A. K. Howard, H. Puschmann, *J. Appl. Cryst.*, 2009, **42**, 339-341.
[55] G. M. Sheldrick, *Acta Cryst.* 2015, **A71**, 3.
[56] L. J. Bourhis, O. V. Dolomanov, R. J. Gildea, J. A. K. Howard, H. Puschmann, *Acta Cryst. A*, 2015, *A71*, 59.
